# Effects of tirzepatide on circulatory overload and end-organ damage in heart failure with preserved ejection fraction and obesity: a secondary analysis of the SUMMIT trial

**DOI:** 10.1038/s41591-024-03374-z

**Published:** 2024-11-17

**Authors:** Barry A. Borlaug, Michael R. Zile, Christopher M. Kramer, Seth J. Baum, Karla Hurt, Sheldon E. Litwin, Masahiro Murakami, Yang Ou, Navneet Upadhyay, Milton Packer

**Affiliations:** 1 Department of Cardiovascular Diseases, Mayo Clinic, Rochester, MN USA; 2 Division of Cardiology, Medical University of South Carolina and the Ralph H. Johnson Veterans Affairs Medical Center, Charleston, SC USA; 3 Cardiovascular Division, Department of Medicine, Department of Radiology and Medical Imaging, University of Virginia Health, Charlottesville, VA USA; 4 Flourish Research, Boca Raton, FL USA; 5 Eli Lilly and Company, Indianapolis, IN USA; 6 Baylor University Medical Center, Dallas, TX USA; 7 Imperial College, London, UK

**Keywords:** Outcomes research, Translational research

## Abstract

Patients with obesity-related heart failure with preserved ejection fraction (HFpEF) display circulatory volume expansion and pressure overload contributing to cardiovascular–kidney end-organ damage. In the SUMMIT trial, patients with HFpEF and obesity were randomized to the long-acting glucose-dependent insulinotropic polypeptide receptor and glucagon-like peptide-1 receptor agonist tirzepatide (*n* = 364, 200 women) or placebo (*n* = 367, 193 women). As reported separately, tirzepatide decreased cardiovascular death or worsening heart failure. Here, in this mechanistic secondary analysis of the SUMMIT trial, tirzepatide treatment at 52 weeks, as compared with placebo, reduced systolic blood pressure (estimated treatment difference (ETD) −5 mmHg, 95% confidence interval (CI) −7 to −3; *P* < 0.001), decreased estimated blood volume (ETD −0.58 l, 95% CI −0.63 to −0.52; *P* < 0.001) and reduced C-reactive protein levels (ETD −37.2%, 95% CI −45.7 to −27.3; *P* < 0.001). These changes were coupled with an increase in estimated glomerular filtration rate (ETD 2.90 ml min^−1^ 1.73 m^−2^ yr^−1^, 95% CI 0.94 to 4.86; *P* = 0.004), a decrease in urine albumin–creatinine ratio (ETD 24 weeks, −25.0%, 95% CI −36 to −13%; *P* < 0.001; 52 weeks, −15%, 95% CI −28 to 0.1; *P* = 0.051), a reduction in N-terminal prohormone B-type natriuretic peptide levels (ETD 52 weeks −10.5%, 95% CI −20.7 to 1.0%; *P* = 0.07) and a reduction in troponin T levels (ETD 52 weeks −10.4%, 95% CI −16.7 to −3.6; *P* = 0.003). In post hoc exploratory analyses, decreased estimated blood volume with tirzepatide treatment was significantly correlated with decreased blood pressure, reduced microalbuminuria, improved Kansas City Cardiomyopathy Questionnaire Clinical Summary Score and increased 6-min walk distance. Moreover, decreased C-reactive protein levels were correlated with reduced troponin T levels and improved 6-min walk distance. In conclusion, tirzepatide reduced circulatory volume–pressure overload and systemic inflammation and mitigated cardiovascular–kidney end-organ injury in patients with HFpEF and obesity, providing new insights into the mechanisms of benefit from tirzepatide. ClinicalTrials.gov registration: NCT04847557.

## Abstract (web summary)

In a mechanistic analysis of the SUMMIT trial that tested tirzepatide in patients with heart failure with preserved ejection fraction and obesity, treatment with this dual glucagon-like peptide-1 receptor and gastric inhibitory polypeptide agonist, as compared with placebo, reduced blood pressure and estimated circulatory volume–pressure overload, reduced systemic inflammation and mitigated cardiovascular and kidney injury.

## Main

Over half of patients with heart failure (HF) have a preserved ejection fraction (HF with preserved ejection fraction, HFpEF), and roughly two-thirds of these patients have obesity^1–3^. Obesity-related HFpEF represents the most advanced form of cardiovascular–kidney–metabolic disease, a syndrome recognized by the American Heart Association^4^. Individuals with obesity-related HFpEF have a high burden of hypertension and chronic kidney disease (CKD), which develops in response to marked blood volume (BV) expansion and chronic systemic inflammation^5–8^. Indeed, hypertension, CKD and BV expansion are the three features that best distinguish obesity-related HFpEF from asymptomatic individuals with obesity but no HF^9^. Endothelial dysfunction, inflammation and CKD in HFpEF can underlie the development of microalbuminuria, which is correlated with greater cardiac dysfunction and congestion, along with higher risk for HF hospitalization or death^10–12^. Patients with HFpEF also frequently display chronic low-grade myocardial injury as reflected by elevated levels of troponin^13,14^, and like albuminuria, this degree of myocardial injury in HFpEF is correlated with the severity of cardiac dysfunction, hemodynamic congestion, impairment in functional capacity and risk for HF hospitalization^13,14^. Notably, cardiac injury is greater in patients with obesity-related HFpEF compared with those without obesity^15^.

The Study of Tirzepatide in Participants with Heart Failure with Preserved Ejection Fraction and Obesity (SUMMIT) trial showed that the long-acting glucose-dependent insulinotropic polypeptide receptor and glucagon-like peptide-1 receptor agonist tirzepatide reduced the risk of worsening HF compared with placebo in patients with obesity-related HFpEF, while reducing HF symptom severity and improving exercise tolerance^16^. The mechanisms underlying these favorable effects of tirzepatide remain unclear. Here, we tested the hypothesis that tirzepatide may reduce cardiovascular–kidney end-organ damage while exerting favorable effects on circulatory volume–pressure overload and systemic inflammation in patients with obesity-related HFpEF participating in the SUMMIT trial.

## Results

### Patient characteristics

A total of 731 patients with obesity-related HFpEF were randomly assigned to receive tirzepatide (*n* = 364) or placebo (*n* = 367) at 129 centers in 9 countries between 20 April 2021 and 30 June 2023, with data collection through the final study visit on 2 July 2024. Participants displayed typical characteristics of obesity-related HFpEF, with mean body mass index (BMI) exceeding 38 kg m^−2^, increased waist circumference and high prevalence of comorbidities including coronary disease and atrial fibrillation, with no baseline differences in those randomized to tirzepatide or placebo (Table 1). Patient-reported health status and exercise capacity were severely impaired (mean Kansas City Cardiomyopathy Questionnaire Clinical Summary Score (KCCQ-CSS), 54; mean 6-min walk distance (6MWD), 303 m), and nearly half the participants had experienced a hospitalization or urgent care visit for worsening HF in the preceding 12 months. Chronic low-grade myocardial injury was common, with 41% of participants having elevated levels of high-sensitivity troponin T (Table 1).

**Table 1:** Baseline characteristics

Characteristic	Tirzepatide (*n* = 364)	Placebo (*n* = 367)
Age (years)	65.5 ± 10.5	65.0 ± 10.9
Women	200 (54.9%)	193 (52.6%)
Race		
American Indian, Alaska Native or Pacific Islanders	26 (7.1%)	24 (6.5%)
Asian	58 (15.9%)	73 (19.9)
Black or African American	22 (6.0%)	14 (3.8%)
White	256 (70.3%)	256 (69.8%)
Other or mixed race	2 (0.5%)	0 (0.0%)
Ethnicity		
Hispanic or Latino	195 (53.6%)	205 (55.9%)
Not Hispanic or Latino	164 (45.1%)	159 (43.3%)
Region		
United States	83 (22.8%)	68 (18.5%)
Latin America	193 (53.0%)	197 (53.7%)
Asia	58 (15.9%)	73 (19.9%)
Other	30 (8.2%)	29 (7.9%)
Measures of adiposity		
Body weight (kg)	102.9 ± 21.7	103.1 ± 22.7
BMI (kg m^−2^)	38.3 ± 6.4	38.2 ± 7.0
Waist circumference (cm)	120 ± 15	120 ± 14
HFpEF severity and phenotyping		
NYHA Class II	262 (72.0%)	268 (73.0%)
NYHA Class III–IV	102 (28.0%)	99 (27.0%)
NT-proBNP, median (IQR), ng l^−1^	196 (56, 488)	169 (64, 476)
KCCQ-CSS score	53.9 ± 17.9	53.2 ± 19.0
6MWD (m)	305.0 ± 80.0	300.6 ± 83.5
Left ventricular ejection fraction (%)	61.0 ± 6.5	60.6 ± 6.2
High-sensitivity troponin T, median (IQR), (ng l^−1^)	11 (7–19)	12 (8–19)
Elevated troponin T (>14 ng l^−1^), *n* (%)	151 (42.2%)	147 (40.6%)
Current atrial fibrillation, *n* (%)	95 (26.1%)	91 (24.8%)
Cardiovascular history		
Hospitalization for HF within 12 months	171 (47.0%)	172 (46.9%)
Diabetes mellitus	174 (47.8%)	178 (48.5%)
Coronary artery disease	111 (30.9%)	106 (29.1%)
Cardiovascular medications		
Diuretics	267 (73.4%)	271 (73.8%)
RAS and neprilysin inhibitors	293 (80.5%)	295 (80.4%)
Beta blocker	245 (67.3%)	263 (71.7%)
Mineralocorticoid receptor antagonist	131 (36.0%)	125 (34.1%)
Sodium–glucose cotransporter 2 inhibitor	69 (19.0%)	57 (15.5%)

Plus–minus values are mean ± s.d. The numbers in parentheses are percentages or IQR. Race was self-reported; patients who identified with ≥1 race or with no race were classified as other. Renin–angiotensin system (RAS) inhibitors include angiotensin-converting enzyme inhibitors, angiotensin receptor blockers and angiotensin receptor neprilysin inhibitors.

Participants displayed borderline hypertension at baseline, with increased estimated BV and plasma volume (PV) in keeping with the diagnosis of obesity-related HFpEF (Table 2). Kidney dysfunction was common, with a baseline mean estimated glomerular filtration rate (eGFR) of 55.3 ml min^−1^ 1.73 m^−2^. Over half of the participants had stage 3 or greater CKD, over one-quarter had microalbuminuria and a smaller proportion had macroalbuminuria. Patients with HFpEF also displayed systemic inflammation, with median C-reactive protein (CRP) of 3.2 mg l^−1^, and 66% had elevated CRP (>2.0 mg l^−1^).

**Table 2:** Baseline circulatory pressure and volume status, kidney function and systemic inflammation

Characteristic	Tirzepatide (*n* = 364)	Placebo (*n* = 367)
**Baseline hemodynamics and volume status**		
Systolic BP (mmHg)	127.9 ± 13.1	128.2 ± 13.7
Diastolic BP (mmHg)	75.7 ± 9.6	76.9 ± 10.0
Pulse pressure (mmHg)	52.1 ± 12.7	51.2 ± 12.4
Estimated total BV (l)	5.77 ± 0.96	5.74 ± 0.94
Estimated PV (l)	3.34 ± 0.58	3.32 ± 0.57
**Baseline kidney indices**		
eGFR, ml min^−1^ 1.73 m^−2^	55.5 ± 23.1	55.1 ± 21.7
Stage 3 or greater CKD, *n* (%)	212 (58.2%)	229 (62.4%)
Cystatin C, median (IQR) (mg l^−1^)	1.26 (1.04–1.63)	1.29 (1.07–1.60)
UACR, median (IQR), (g kg^−1^)	17 (8–47)	20 (8–65)
Microalbuminuria (UACR 30–300), *n* (%)	96 (26.5%)	105 (28.9%)
Macroalbuminuria (UACR >300), *n* (%)	22 (6.1%)	39 (10.7%)
**Systemic inflammation**		
High-sensitivity CRP, median (IQR), mg l^−1^	3.1 (1.6–6.5)	3.4 (1.4–6.4)
Elevated CRP (>2 mg l^−1^), *n* (%)	233 (66.6%)	227 (65.6%)

Plus–minus values are mean ± s.d., median (IQR) or number (percentage).

### Tirzepatide effects on hemodynamic load and inflammation

**Fig. 1:**
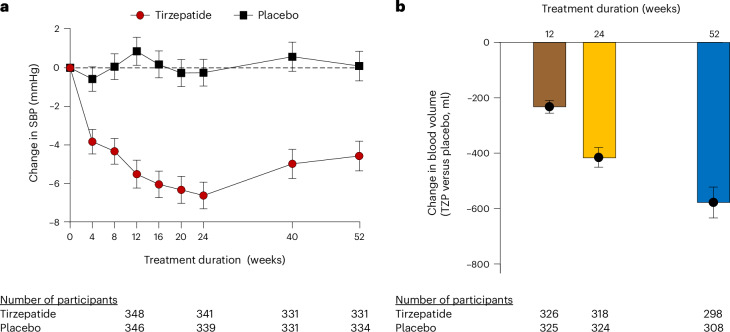
Effects of tirzepatide on pressure overload and volume expansion. **a**, Least-squares mean changes from baseline in systolic BP (SBP) plotted as a function of duration of treatment in participants randomized to tirzepatide (red circles) or placebo (black squares). **b**, The estimated mean treatment difference in the change in BV versus baseline with tirzepatide (TZP) compared with placebo plotted as a function of duration of treatment. The error bars represent s.e.m. for both panels; two-sided *P* < 0.001 for each time point comparison for **a** and **b** based upon comparing the least-squares means calculated from a mixed-model repeated-measures model incorporating baseline value, sex, history of HF decompensation within 12 months, diabetes status, baseline BMI group (<35, ≥35 kg m^−2^), treatment assignment, time, and treatment × time interaction. No correction was made for multiple hypothesis testing.

As compared with placebo, treatment with tirzepatide reduced systolic blood pressure (BP), an effect that was evident after 4 weeks, with a progressive decline up to 24 weeks, which was maintained after 52 weeks (Table 3 and Fig. 1a). In contrast, the effects on diastolic BP were minimal and not statistically significant at 24 or 52 weeks of treatment. Pulse pressure, an indirect reflection of aortic stiffness, was significantly reduced by tirzepatide as compared with placebo at each time point (Table 3).

**Table 3:** Effects of tirzepatide on circulatory pressure–volume characteristics, inflammation and kidney function

Variable and duration of treatment	Change with tirzepatide from baseline	Change with placebo from baseline	ETD	95% CI	*P*
Systolic BP (mmHg)					
12 weeks	−5.5	0.9	−6.4	−8.4 to −4.4	<0.001
24 weeks	−6.6	−0.3	−6.4	−8.3 to −4.5	<0.001
52 weeks	−4.6	0.1	−4.7	−6.8 to −2.5	<0.001
Diastolic BP (mmHg)					
12 weeks	−1.1	−0.5	−0.7	−1.9 to −0.6	0.313
24 weeks	−1.7	−0.5	−1.3	−2.5 to 0.0	0.054
52 weeks	−1.2	−0.3	−0.9	−2.3 to 0.5	0.189
Pulse pressure (mmHg)					
12 weeks	−4.4	1.4	−5.8	−7.4 to −4.1	<0.001
24 weeks	−4.9	0.3	−5.2	−6.8 to −3.7	<0.001
52 weeks	−3.5	0.4	−3.9	−5.6 to −2.2	<0.001
Estimated BV (l)					
12 weeks	−0.27	−0.04	−0.23	−0.26 to −0.21	<0.001
24 weeks	−0.49	−0.07	−0.42	−0.45 to −0.38	<0.001
52 weeks	−0.68	−0.11	−0.58	−0.63 to −0.52	<0.001
Estimated PV (l)					
12 weeks	−0.17	−0.03	−0.13	−0.16 to −0.11	<0.001
24 weeks	−0.27	−0.04	−0.23	−0.26 to −0.19	<0.001
52 weeks	−0.39	−0.08	−0.32	−0.36 to −0.27	<0.001
High-sensitivity CRP (% change from baseline)					
12 weeks	−4.7	1.9	−6.5	−18.0 to 6.7	0.318
24 weeks	−21.5	−6.7	−15.9	−26.4 to −4.0	0.010
52 weeks	−39.8	−4.1	−37.2	−45.7 to −27.3	<0.001
eGFR (ml min^−1^ 1.73 m^−2^)					
12 weeks	−0.6	0.5	−1.1	−2.7 to 0.4	0.148
24 weeks	0.3	0.1	0.2	−1.4 to 1.8	0.818
52 weeks	2.6	−0.3	2.9	0.9 to 4.9	0.004
UACR (% change from baseline)					
24 weeks	−26.4	−1.9	−25.0	−35.5 to −12.7	<0.001
52 weeks	−14.7	0.4	−15.1	−28.0 to 0.1	0.051
NT-proBNP level (% change from baseline)					
12 weeks	−8.9	2.0	−10.8	−19.1 to −1.5	0.024
24 weeks	−10.2	−0.6	−9.7	−18.9 to 0.5	0.063
52 weeks	−7.2	3.7	−10.5	−20.7 to 1.0	0.072
Troponin T level (% change from baseline)					
12 weeks	−1.3	−0.8	−0.6	−7.5 to 7.0	0.881
24 weeks	−8.6	1.4	−9.8	−16.0 to −3.3	0.004
52 weeks	−9.6	0.9	−10.4	−16.7 to −3.6	0.003

*P* values are two-sided for each time point comparison based upon comparing the least-squares means calculated from a mixed-model repeated-measures model incorporating baseline value, history of HF decompensation within 12 months, diabetes status, baseline BMI group (<35, ≥35 kg m^−2^), treatment assignment, time, and treatment × time interaction. No correction was made for multiple hypothesis testing.

As compared with placebo, tirzepatide resulted in a reduction in estimated BV at 12 weeks and 24 weeks, which further decreased at 52 weeks (estimated treatment difference (ETD) −0.58 l, 95% confidence interval (CI) −0.63 to −0.52, *P* < 0.001; Table 3 and Fig. 1b). Tirzepatide resulted in similar reductions in estimated PV at each time point (Table 3).

**Fig. 2:**
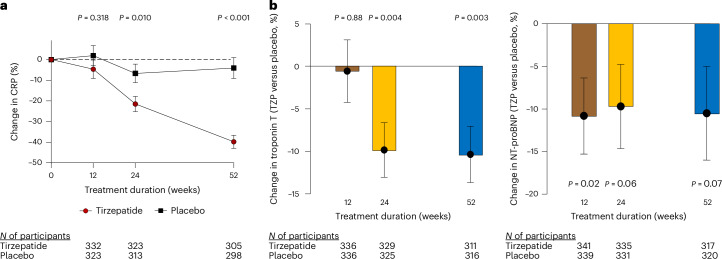
Effects of tirzepatide on systemic inflammation, cardiac injury and natriuretic peptide levels. **a**, Least-squares mean changes from baseline in CRP plotted as a function of duration of treatment in participants randomized to tirzepatide (red circles) or placebo (black squares). **b**, The estimated mean treatment difference in the change in troponin T and NT-proBNP with tirzepatide (TZP) to baseline compared with placebo plotted as a function of duration of treatment. The error bars represent the s.e.m. for all panels; *P* values are two-sided for each time point comparison for **a** and **b** based upon comparing the least-squares means calculated from a mixed-model repeated-measures model incorporating baseline value, history of HF decompensation within 12 months, diabetes status, baseline BMI group (<35, ≥35 kg m^−2^), treatment assignment, time, and treatment × time interaction. No correction was made for multiple hypothesis testing.

Tirzepatide reduced systemic inflammation as measured by high-sensitivity CRP at 24 weeks and to greater extent at 52 weeks, but with no significant effect at 12 weeks (Table 3 and Fig. 2a). Among patients with elevated CRP at baseline, those treated with tirzepatide had greater likelihood of normalization of CRP (to <2 mg l^−1^) at 52 weeks compared with placebo (40.4% versus 19.4%, odds ratio 2.90, 95% CI 1.82 to 4.64; *P* < 0.001).

### Effects of tirzepatide on cardiac injury and kidney function

Treatment with tirzepatide resulted in a significant reduction in myocardial injury reflected by high-sensitivity troponin T as compared with placebo at both 24 and 52 weeks (ETD 24 weeks −9.8% (95% CI −16.0 to −3.3), *P* = 0.004, 52 weeks −10.4% (95% CI −16.7 to −3.6), *P* = 0.003), with no significant effect at 12 weeks (Fig. 2b). Tirzepatide also reduced N-terminal prohormone B-type natriuretic peptide (NT-proBNP) levels compared with placebo, *P* = 0.02–0.07 (Fig. 2), even as baseline levels were near normal on average (Table 1).

**Fig. 3:**
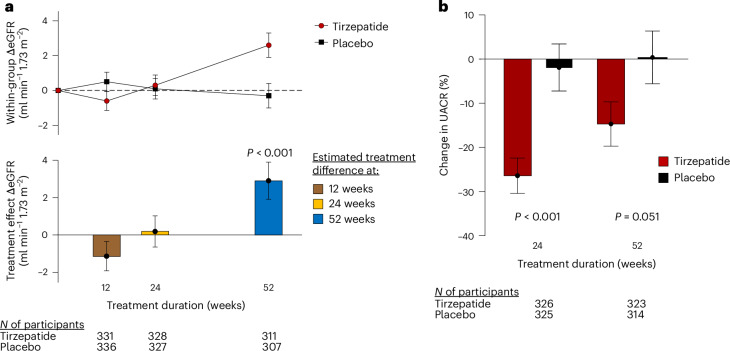
Effects of tirzepatide on kidney function and albuminuria. **a**, Top: least-squares mean changes from baseline in eGFR plotted as a function of duration of treatment in participants randomized to tirzepatide (red circles) or placebo (black squares). Bottom: the estimated mean treatment difference in the change in eGFR with tirzepatide versus baseline compared with placebo plotted as a function of duration of treatment. **b**, The least-squares mean change in UACR in participants treated with tirzepatide (red) and placebo (black) as a function of duration of treatment. The error bars represent the s.e.m. for all panels; *P* values are two-sided for each time point comparison for **a** and **b** based upon comparing the least-squares means calculated from a mixed-model repeated-measures model incorporating baseline value, sex, history of HF decompensation within 12 months, diabetes status, baseline BMI group (<35, ≥35 kg m^−2^), treatment assignment, time, and treatment × time interaction. No correction was made for multiple hypothesis testing.

As compared with placebo, treatment with tirzepatide was associated with a numerical decrease in eGFR at 12 weeks that was not statistically significant (ETD −1.1 ml min^−1^ 1.73 m^−2^, 95% CI −2.7 to 0.4; *P* = 0.148), followed by a significant improvement in eGFR at 52 weeks with tirzepatide compared with placebo (ETD 2.9 ml min^−1^ 1.73 m^−2^, 95% CI 0.9 to 4.9; *P* = 0.004) (Table 3 and Fig. 3a). The improvement in kidney function was consistently observed in sensitivity analyses using alternative equations to estimate eGFR, but using the creatinine-based formulas, there was a statistically significant decrease in eGFR versus placebo at 12 weeks, which again reversed by 52 weeks, showing an improvement in eGFR with tirzepatide across all estimates (Extended Data Table 1). As compared with placebo, tirzepatide decreased the urine albumin–creatinine ratio (UACR) at 24 weeks (ETD −25%, 95% CI −35.5 to −12.7; *P* < 0.001) and at 52 weeks (ETD −15.1%, 95% CI −28.0 to 0.1; *P* = 0.051) (Table 3 and Fig. 3b).

### Changes in circulatory load, inflammation and end-organ damage

In a linear regression model adjusted for baseline value, treatment allocation and interaction between treatment and covariates, at 52 weeks, the reductions in estimated BV with tirzepatide were correlated with decreases in systolic BP (slope 6.12, 95% CI 3.47 to 8.78; *P* < 0.001) and decreases in albuminuria (slope 46.7, 95% CI 11.48 to 81.87; *P* = 0.009), but there was no correlation with changes in eGFR (Extended Data Table 2). These relationships were not observed in patients randomized to placebo. There was a modest direct correlation between changes in BV and eGFR at 12 weeks (Extended Data Table 3).

There was a direct correlation between changes in systolic BP and changes in eGFR on treatment with tirzepatide at 52 weeks (slope 0.256, 95% CI 0.127 to 0.386; *P* < 0.001) (Extended Data Table 2). The direct relationship between change in eGFR and change in BP was also observed at 12 weeks (Extended Data Table 3). There was no significant relationship between changes in systolic BP and changes in log UACR at 52 weeks on treatment with tirzepatide.

There were no significant correlations between changes in CRP with tirzepatide and changes in systolic BP, eGFR or log UACR (Extended Data Table 2). Changes in systolic BP and BV with tirzepatide were not correlated with changes in troponin T. However, the decrease in CRP with tirzepatide at 52 weeks was correlated with reduction in log troponin T (slope 0.239, 95% CI 0.029 to 0.450; *P* = 0.026).

### Correlations with clinical outcomes

As previously reported^16^, treatment with tirzepatide improved KCCQ-CSS score at 52 weeks compared with placebo (ETD 6.9 points, 95% CI 3.3 to 10.6; *P* < 0.001), reduced body weight (ETD 11.6%, 95% CI −12.9% to −10.4%; *P* < 0.001) and increased 6MWD (ETD 18.3 m, 95% CI 9.9 to 26.7; *P* < 0.001). In the tirzepatide group, reductions in systolic BP had no significant relationship with clinical outcomes, but decreases in estimated BV were correlated with improvements in KCCQ-CSS (slope −6.287, 95% CI −8.091 to −4.482; *P* < 0.001) and increases in 6MWD (slope −1.175, 95% CI −1.810 to −0.539; *P* < 0.001) (Extended Data Table 4). Decreases in CRP with tirzepatide were associated with improvement in 6MWD (slope −0.002, 95% CI −0.004 to 0.000; *P* = 0.016), but not changes in KCCQ-CSS.

## Discussion

In this mechanistic analysis of the SUMMIT trial, we found that, compared with placebo, treatment with the long-acting glucose-dependent insulinotropic polypeptide receptor and glucagon-like peptide-1 receptor agonist tirzepatide reduced BP and estimates of circulatory volume expansion in patients with obesity-related HFpEF. These effects were observed early and were sustained with longer duration of treatment. Tirzepatide reduced systemic inflammation, but this effect was observed later than the hemodynamic effects. Tirzepatide resulted in an early trend to reduction in eGFR at 12 weeks, but this was followed by an improvement in eGFR compared with placebo with longer duration treatment, and reduction in microalbuminuria. Tirzepatide reduced myocardial injury compared with placebo, reflected by a significant decrease in troponin T, along with a reduction in NT-proBNP. Reductions in estimated BV with tirzepatide were correlated with decreases in systolic BP, reduction in UACR and improvements in both KCCQ-CSS score and 6MWD, suggesting that mitigation of circulatory volume expansion (or factors responsible for volume expansion) may play a central role in mediating the benefits of tirzepatide. Conversely, decreases in systemic inflammation with tirzepatide were correlated with reductions in cardiac injury with tirzepatide, along with a modest correlation with improvements in 6MWD. These findings provide new insights into the mechanisms by which tirzepatide leads to clinical improvement in HFpEF.

Obesity-related HFpEF is the most common phenotype of this disorder^3,9^, and its emergence is causally related to the growing burden of cardiovascular–kidney–metabolic disorders observed worldwide^4^. As compared with nonobese HFpEF, patients with obesity-related HFpEF display distinct pathophysiologic features, including greater volume expansion^5,17^, more severe inflammation^6,7^, increases in visceral and paracardiac adipose tissue^5,15,18,19^ and more severe myocardial injury^15^. Indeed, volume expansion, CKD and greater burden of hypertension best distinguish individuals from obesity-related HFpEF from age, sex and BMI-matched patients with obesity but no HF^9^. Collectively, these observations suggest that novel treatments that reduce circulatory volume–pressure overload (or its determinants), improve kidney function, reduce inflammation and mitigate cardiac injury may improve clinical status in people with obesity-related HFpEF.

In the SUMMIT trial, tirzepatide reduced the combined risk of cardiovascular death or worsening HF, and improved health status and exercise tolerance in patients with obesity-related HFpEF^16^. These results are consistent with findings observed in the STEP-HFpEF program, where the glucagon-like peptide receptor-1 agonist semaglutide was also shown to improve health status and exercise capacity^20,21^. The magnitude of improvements in health status and exercise tolerance with semaglutide were correlated with the degree of body weight reduction^22^, but the mechanisms underlying clinical improvement with incretins such as tirzepatide and semaglutide in patients with obesity-related HFpEF have remained unclear.

Here, we show that tirzepatide reduced circulatory volume expansion using estimates of BV and PV, an effect that was evident after just 12 weeks of treatment and that further increased at 24 and 52 weeks. It is noteworthy that adipocytes release factors that promote sodium retention and volume expansion, including leptin and aldosterone, and the reduction in adipocyte mass with tirzepatide may reduce these influences^23^. Volume expansion in obesity-related HFpEF exacerbates elevation in cardiac filling pressures that develop due to impairments in left ventricular distensibility^3^, and thus, reducing circulating volume may decrease cardiac filling pressures. This finding could partly explain the direct correlation observed between reductions in BV and improvements in KCCQ-CSS score and exercise capacity.

Tirzepatide also decreased systolic BP, an effect that was evident after just 4 weeks, and was also associated with reductions in estimated BV and PV. Effects on diastolic BP were not apparent, and thus, there was a significant reduction in pulse pressure. Pulse pressure is inversely correlated with arterial compliance, which is frequently impaired in patients with HFpEF, where increases in aortic stiffness contribute to elevation in cardiac filling pressures, especially during exercise^24^. Treatment with the sodium–glucose cotransporter 2 inhibitor dapagliflozin was recently shown to reduce aortic stiffness, systolic BP and pulse pressure during exercise after just 24 weeks, and these changes were correlated with reductions in left ventricular filling pressures^25^. The magnitude of improvement in arterial compliance with dapagliflozin was correlated with the magnitude of weight loss. Further study using more direct measures is indicated to determine whether tirzepatide also improves arterial compliance, which would be consistent with the effects on systolic and pulse pressure observed here.

Obesity can contribute to CKD through multiple mechanisms including volume expansion (leading to glomerular hyperfiltration) and the effect of adipocytokines (leptin and aldosterone) to cause renal injury and fibrosis^23,26^. Leptin is an important determinant of the decline in eGFR in the general population, and mineralocorticoid receptor antagonists ameliorate the progression of CKD^23,27^. In the SUMMIT trial, decreases in estimated BV at 52 weeks (potentially related to the suppression of adipocytokines) were associated with reduction in albuminuria, but we observed no relationship with the improvements in eGFR. Tirzepatide tended to worsen eGFR estimated using cystatin C compared with placebo at 12 weeks, but this was reversed by 52 weeks, an effect that was consistently observed across all formulas used to determine eGFR. This pattern of an early worsening of eGFR followed by favorable improvements with longer-term follow-up was also observed in the tirzepatide versus insulin glargine in type 2 diabetes and increased cardiovascular risk (SURPASS-4) trial with tirzepatide^28^ and the Evaluate Renal Function with Semaglutide Once Weekly (FLOW) trial with semaglutide^29^, as well as other renoprotective drugs such as sodium–glucose cotransporter 2 inhibitors^30^. The present study suggests that the bimodal relationship may be explained by a relatively early-onset reduction in systolic BP and estimated BV, resulting in short-term decline in eGFR at 12 weeks. This is then superseded by a favorable effect on eGFR and UACR, potentially mediated by long-term decrease in fat mass and the suppression of adipocytokines.

Improvements in kidney function with incretins have been postulated to be related to a decrease in systemic inflammation, but we observed no correlation between changes in CRP and changes in eGFR. Systemic hypertension has been linked to the development of obesity-related CKD^26^, but we observed a direct relationship between decreases in systolic BP and decreases in eGFR at multiple time points. This may reflect the impact of reductions in renal perfusion pressure that have been associated with reduction in eGFR in both acute and chronic HF^30,31^. Tirzepatide reduced albuminuria compared with placebo, with a significant effect at 24 weeks, preceding the improvement in eGFR, which is also consistent with a decrease in glomerular hypertension as a key mechanism.

Tirzepatide reduced cardiac injury, reflected by decreases in troponin T, and reduced wall stress, reflected by decreases in NT-proBNP. The latter is notable because weight loss in the absence of HF leads to increases in NT-proBNP^32,33^. We did not require elevated NT-proBNP as an eligibility criterion, and thus, NT-proBNP levels in SUMMIT were only slightly elevated on average (median 175 ng l^−1^), and accordingly, there was little margin for further reduction. Circulating troponin levels are commonly elevated in HFpEF, where they are associated with greater risk for HF hospitalization or death^14^. The low-grade myocardial injury in HFpEF as observed at baseline in SUMMIT is believed to be related to cardiomyocyte loss due to repetitive elevations in cardiac filling pressures during stress, along with adverse loading conditions caused by ventricular–vascular stiffening^13,34^. Patients with HFpEF and elevated troponin have more severe elevation in filling pressures, reduced myocardial stress reserve and poorer exercise capacity compared with those without injury^13^, and individuals with the obesity phenotype have higher troponin levels than those without obesity^15^. We observed a modest but significant correlation between decreases in CRP and reductions in troponin with tirzepatide, suggesting that anti-inflammatory effects may contribute to cardiac benefits. It is noteworthy that the temporal sequence of anti-inflammatory effects and the changes in troponin were similar, and these were delayed compared with effects on circulatory volume and pressure, further supporting a distinct mechanism. The favorable effects of tirzepatide on myocardial injury may contribute to reduced risk for worsening HF seen in the SUMMIT trial^16^. Reduction in CRP with tirzepatide was also associated with greater improvement in 6MWD. This may relate in part to myocardial benefits, as noted above, or potentially improvements in skeletal muscle function and peripheral oxygen uptake, which are more dramatically impaired in patients with HFpEF and more severe inflammation^35^.

The major limitation of this analysis centers on the fact that we estimated PV using a weight-based formula (the Kaplan–Hakim equation)^36^, and according to this formula, the weight loss produced by tirzepatide would be mathematically expected to reduce PV. However, such an effect is physiologically inescapable, since PV/BV is inherently linked to body weight^37^, and thus, PV/BV necessarily declines as weight is lost. The mechanism that mediates this effect is probably due to a decrease in fat mass to reduce the secretion of adipocytokines that have antinatriuretic effects. However, in the case of tirzepatide, it is noteworthy that glucagon-like peptide-1 receptor agonism produces a direct effect itself to promote urinary sodium excretion, independent of changes in adipose tissue volume^38,39^. We understand that weight-independent formulas to estimate changes in PV have been proposed^40^, but these are relevant only for short-term interventions and are not applicable when there are meaningful changes in weight over months. Nevertheless, it is possible that we may have overestimated the magnitude of BV and PV reduction with tirzepatide in our analysis, as the relationship between BV and body weight may change as people lose weight. An alternative and more quantitative approach would be to directly measure PV/BV using the indicator-dilution method, but that would not be practical in a large-scale, international, multicenter randomized trial, leaving us no viable alternatives to explore this potential mechanism. All outcomes and analyses were prespecified, with the exception of the estimated BV and PV, as well as the regression analyses used to explore potential mechanisms, so these data should be treated as hypothesis-generating. Patients with active chronic noninflammatory myopathy and myositis may have mild elevation in troponin T independent of myocardial injury^41^. However, patients with significant musculoskeletal disease were excluded from SUMMIT by protocol. Reductions in body weight with tirzepatide would be expected to reduce skeletal muscle to some extent, which could influence creatinine dependent of changes in kidney function, and for this reason, we calculated eGFR using cystatin C in place of creatinine. Importantly, the findings showing kidney benefits were consistently observed across all formulas used to calculate eGFR. The duration of follow-up assessments for most measures was 52 weeks, reducing our ability to reach conclusions about longer-term effects.

In this mechanistic analysis from the SUMMIT trial, we show that tirzepatide reduced circulatory volume–pressure overload and systemic inflammation in patients with obesity-related HFpEF, while mitigating cardiovascular–kidney end-organ injury and loss of function. These data provide new insights into the mechanisms of benefit from tirzepatide in patients with obesity-related HFpEF.

## Methods

The SUMMIT primary results, trial protocol and statistical analysis plan have been published, and the protocol and statistical analysis plan are included in Supplementary Information^16^. The ethics committee at each investigative site approved the trial, and all patients provided written informed consent. The registration identifier on clinicaltrials.gov is NCT04847557. The sponsor was Eli Lilly and Company. In collaboration with the sponsor, the steering committee developed and amended the protocol, oversaw the recruitment of patients and the quality of follow-up, and supervised the analysis of data; the academic members provided an independent interpretation of the results.

### Study patients

Participants with HFpEF aged ≥40 years with chronic class II–IV HF, left ventricular ejection fraction ≥50% and a BMI ≥30 kg m^−2^ were enrolled. Patients were required to have exercise limitation and high symptom severity due to HFpEF reflected by a 6MWD of 100–425 m and a KCCQ-CSS ≤80 at baseline, along with objective evidence of HF, as demonstrated by at least one of the following: (1) elevated NT-proBNP (≥200 pg ml^−1^ in sinus rhythm or ≥600 pg ml^−1^ in atrial fibrillation); (2) structural heart disease, defined by left atrial enlargement by two-dimensional echocardiography; or (3) elevated filling pressures at rest or with exercise (by invasive hemodynamic measurement or echocardiography). To enrich for greater risk of HF events, participants were also required to have either (1) HF decompensation in the preceding 12 months or (2) eGFR <70 ml min^−1^ 1.73 m^−2^. Full inclusion and exclusion criteria are summarized in Supplementary Appendix 1.

### Study procedures

Following screening, eligible patients were randomized double blind (1:1) to receive placebo or tirzepatide 2.5 mg per week subcutaneously, in addition to usual therapy. Randomization was stratified by (1) HF decompensation within 12 months; (2) type 2 diabetes; (3) BMI ≥35 kg m^−^^2^ or <35 kg m^−2^. The dose of the double-blind study medication was increased by 2.5 mg every 4 weeks as tolerated until a dose of 15.0 mg per week of tirzepatide or matching placebo could be achieved after 20 weeks, which was maintained until the end of the trial. Double-blind treatment was to be continued until the last randomized patient was followed for 52 weeks.

The outcomes for this secondary mechanistic analysis from SUMMIT were identified on the basis of pathophysiologic understanding of obesity-related HFpEF as variables that we hypothesized would change with tirzepatide treatment, including measures of BP, estimated BV and PV, kidney function, albuminuria, cardiac injury, inflammation and cardiac distention (reflected by NT-proBNP). These variables were obtained on the basis of either physical examination (for BP) or laboratory tests (from blood and urine samples). All of the outcomes assessed were prespecified as exploratory outcomes, with the exception of estimated BV and PV, which were post hoc and not prespecified.

Patients were evaluated in a clinical setting at prespecified intervals to assess body weight, BP, New York Heart Association (NYHA) class, worsening HF events, KCCQ-CSS, 6MWD and biomarkers of end-organ function or injury. Cystatin C was determined using a turbidimetric assay (Roche Cobas Chemistry Analyzer). Because weight loss with tirzepatide is expected to reduce both fat and skeletal muscle mass, creatinine could be less accurate as a measure of kidney function. Therefore, for the primary analysis, eGFR was calculated from cystatin C level alone, using the 2012 chronic kidney disease-epidemiology (CKD-EPI) cystatin C formula, at baseline and 12, 24 and 52 weeks^42^. Sensitivity analyses were also performed using eGFR calculated determined using the 2021 creatinine-based CKD-EPI formula^43^, and separately using the combined creatinine-cystatin C CKD-EPI formula (Supplementary Appendix 2)^19^. Urine albumin was measured using a turbidimetric assay and urine creatinine by colorimetric assay (both Roche Cobas Chemistry Analyzer), and UACR was calculated at baseline, 24 weeks and 52 weeks. Systemic inflammation was assessed by high-sensitivity CRP, measured by turbidimetric assay (Roche Cobas Chemistry Analyzer) at baseline and 12, 24 and 52 weeks. Myocardial injury was assessed by high-sensitivity troponin T, measured by chemiluminescent immunoassay (Roche Cobas Elecsys) at baseline, 12 weeks, 24 weeks and 52 weeks. Hematocrit was measured at baseline, 24 weeks and 52 weeks. Estimated PV was calculated from these variables as (1 − hematocrit) × (*a* + (*b* × weight in kg)), where *a* = 1,530 for men and 864 for women and *b* = 41 for men and 47.9 for women^5^. Estimated total BV was then determined by PV/(1 − hematocrit).

### Statistical methods

Data are reported as mean (standard deviation, s.d.), median (interquartile range, IQR) or number (%) unless otherwise specified. All analyses presented were prespecified, with the exception of estimated BV and PV, and the results of linear regression analyses exploring correlations between changes in different variables, which are exploratory and post hoc. Analyses were guided by the treatment policy estimand that represents the efficacy irrespective of adherence to study intervention. A mixed-effects model repeated-measures analysis was used to analyze measurements taken at multiple scheduled visits. The mixed-effects model repeated-measures model included treatment, time, treatment-by-time interaction, stratification factors (HF decompensation within 12 months of screening (yes/no), diagnosed type 2 diabetes (yes/no) and baseline BMI (<35, ≥35 kg m^−2^) as fixed effects, and baseline value as a covariate. Restricted maximum likelihood was used to obtain model parameter estimates, and the Kenward–Roger option was used to estimate the denominator degrees of freedom. An unstructured covariance structure was used to model the within-patient errors.

A linear regression was used to analyze potential relationships between continuous dependent and independent variables, and included baseline value, treatment, specified covariate and treatment-by-covariate interaction. Given that the focus was on mechanistic understanding rather than clinical indications, adjustment for multiple hypothesis testing was not performed. Logistic regression was used to analyze the potential relationship of independent variables with a binary dependent variable, and included baseline value, treatment and stratification factors (HF decompensation within 12 months of screening (yes/no), diagnosed type 2 diabetes (yes/no) and baseline BMI (<35, ≥35 kg m^−2^) as covariates.

### Reporting summary

Further information on research design is available in the Nature Portfolio Reporting Summary linked to this article.

## Online content

Any methods, additional references, Nature Portfolio reporting summaries, source data, extended data, supplementary information, acknowledgements, peer review information; details of author contributions and competing interests; and statements of data and code availability are available at 10.1038/s41591-024-03374-z.

## Supplementary information

Supplementary Information Supplementary Appendices 1 and 2, trial protocol and statistical analysis plan.

Reporting Summary

## Figures and Tables

**Extended Data Table 1:**
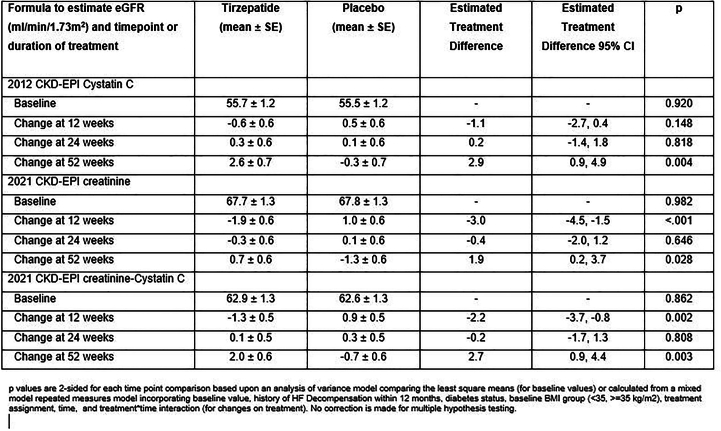
Effects of Tirzepatide on eGFR calculated using alternative formulas

**Extended Data Table 2:**
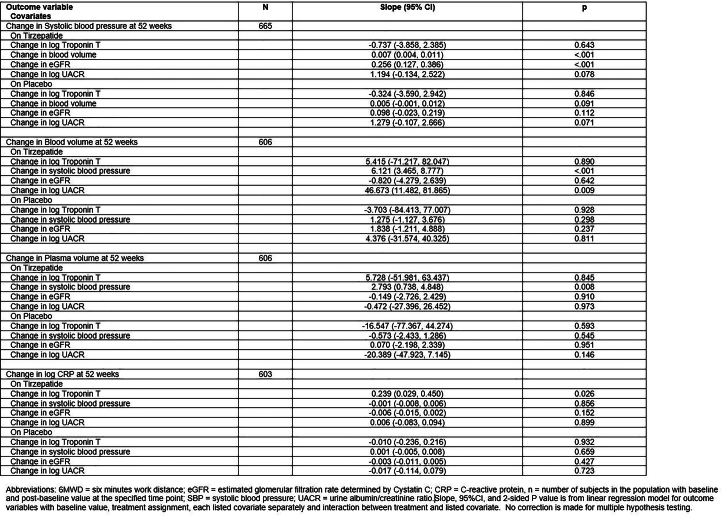
Linear Regression Models Exploring Relationships between Outcome Changes at 52 Weeks

**Extended Data Table 3:**
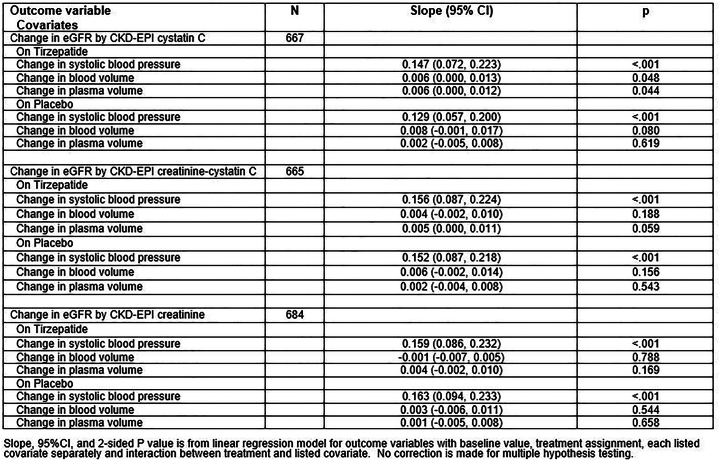
Linear Regression Models Exploring Relationships between Changes in eGFR and Circulatory Volume-Pressure at 12 Weeks

**Extended Data Table 4:**
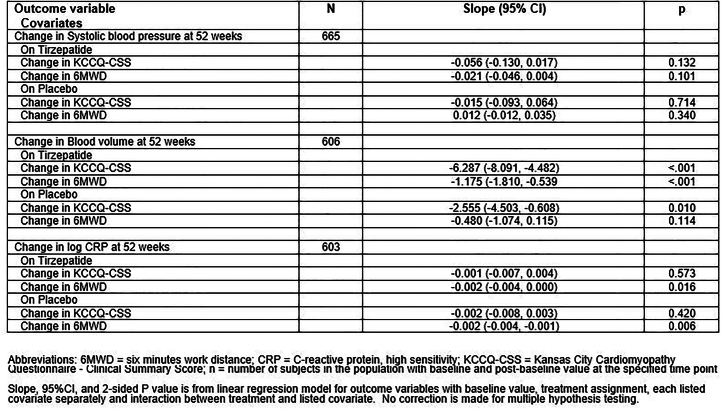
Linear Regression Models Exploring Relationships between Outcome Changes and Clinical Endpoints

## Data Availability

Eli Lilly and Company provides access to all individual participant data collected during the trial, after anonymization, except for pharmacokinetic or genetic data. Data are available to request 6 months after the indication studied has been approved in the United States and European Union and after primary publication acceptance, whichever is later. No expiration date of data requests is currently set once data have been made available. Access is provided after a proposal has been approved by an independent review committee identified for this purpose and after receipt of a signed data-sharing agreement. Data and documents, including the study protocol, statistical analysis plan, clinical study report and blank or annotated case report forms, will be provided in a secure data-sharing environment. The estimated time delay for a researcher requesting access to data to receive a reply from Lilly would be 3–6 months. For details on submitting a request, see the instructions provided at www.vivli.org.
