# Supplementary material for: Effects of tirzepatide on circulatory overload and end-organ damage in heart failure with preserved ejection fraction and obesity: a secondary analysis of the SUMMIT trial
Source: Nat Med. 2024 Nov 17;31(2):544–51. doi: 10.1038/s41591-024-03374-z (PMC11835708; doi:10.1038/s41591-024-03374-z)
Supplement: Supplementary file 1 — Supplementary Appendices 1 and 2, trial protocol and statistical analysis plan. [file 41591_2024_3374_MOESM1_ESM.pdf]

nature medicine

Supplementary information https://doi.org/10.1038/s41591-024-03374-z

Effects of tirzepatide on circulatory overload and end-organ damage in heart failure with preserved ejection fraction and obesity: a secondary analysis of the SUMMIT trial

In the format provided by the authors and unedited

SUPPLEMENTARY APPENDICES

Contents

Supplementary Appendix 1: Inclusion and Exclusion Criteria

Supplementary Appendix 2: Different CKD-EPI formulas used for eGFR estimation

APPENDIX 1: INCLUSION AND EXCLUSION CRITERIA

INCLUSION CRITERIA

Participants are eligible to be included in the study only if all of the following criteria apply:

Age

1. Participant must be at least 40 years of age inclusive at the time of signing the ICF.

Type of Participant and Disease Characteristics

2. 6MWD ≤465 meters at both Visit 1 tests, between ≥100 meters and ≤425 meters at Visit 2 and change from the preceding qualifying 6MWD is <20% and <40 meters.
   - *If* Visit 2a 6MWD is both between 100 and 425 meters and…
     - <20% AND <40 meter change from the higher of the two 6MWDs conducted at Visit 1, *then* participant meets this inclusion criterion.
     - ≥20% OR ≥40 meter change from the higher of the two 6MWDs at Visit 1, *then* participant must attend Visit 2b. If at Visit 2b, the 6MWD is between 100 and 425 meters and <20% AND <40 meter change from preceding (Visit 2a) 6MWD, then participant meets this inclusion criterion.
3. Chronic HF (NYHA Class II-IV) diagnosed for at least 3 months before Visit 1.
4. LVEF ≥50% demonstrated by echocardiogram performed at Visit 1 or within 6 months of Visit 1.
5. At least 1 of the following to document evidence of HF:
   - Elevated NT-proBNP >200 pg/mL for participants without atrial AF or >600 pg/mL for participants with AF, as analyzed at the central laboratory at Visit 1
     OR
   - Evidence of structural heart disease:
     - LA enlargement (any of the following: LAV index ≥29 mL/m^2, or LAV >58 mL in male participants and >52 mL in female participants, or LA area >20 cm^2, or LA diameter >40 mm in male and >38 mm in female participants) determined by echocardiogram at Visit 1 or within 6 months of Visit 1

     OR
   - Evidence of elevated filling pressure:
     - At rest (PCWP ≥15 mmHg or LVEDP ≥15 mmHg) or with exercise (PCWP ≥25 mmHg) (based on historical record, not associated with hospitalization

for decompensation of HF, within 2 years of Visit 1), or

- E/e' ratio >15 (septal) or >13 (average of septal and lateral) determined by echocardiogram at Visit 1 or within 6 months of Visit 1

Note: Supporting medical documentation is required in all instances.

6. Either one of:
   - eGFR <70 mL/min/1.73m^2 at Visit 1, OR
   - HF decompensation within 12 months of Visit 1, defined as hospitalization for HF requiring IV diuretic treatment or urgent HF visit requiring IV diuretic treatment
     Note: Supporting medical documentation is required in all instances.
7. Stable dose of all concomitant HF medications (these may include beta blockers, ACEis, ARBs, MRAs, ARNI, and/or SGLT2is), except for oral diuretics, for at least 4 weeks prior to Visit 1 and throughout the screening period.
8. If treated with oral diuretics, dose must be stable for at least 2 weeks prior to Visit 1 and throughout the screening period; volume control must be optimally achieved in the opinion of the investigator.
9. KCCQ-CSS ≤80 at Visit 1.

Weight

10. BMI ≥30.0 kg/m^2 at Visit 1.

Sex

11. At the time of signing the ICF:
    a. **Male participants**: Male participants with partners of childbearing potential should be willing to use reliable contraceptive methods for the duration of the trial and for 4 months thereafter.
    b. **Female participants:**
       - Female participants not of childbearing potential may participate and include those who are infertile due to surgical sterilization and/or postmenopausal.
       - Female participants of childbearing potential (not surgically sterilized and between menarche and 1-year postmenopausal) must:
         - test negative for pregnancy at Visit 1 based on a serum pregnancy test

followed by a negative urine pregnancy test within 24 hours prior to exposure and agree to use 2 forms of effective contraception, if sexually active, where at least 1 form is highly effective, for the duration of the trial and for 2 months after the last injection, and

- not be breastfeeding.

Contraceptive use by men or women of childbearing potential should be consistent with local regulations regarding the methods of contraception for those participating in clinical trials.

Informed Consent

12. Capable of giving signed informed consent, which includes compliance with the requirements and restrictions listed in the ICF and in the protocol.

EXCLUSION CRITERIA

Participants are excluded from the study if any of the following criteria apply:

Medical Conditions

13. Myocardial infarction, coronary artery bypass graft surgery, or other major CV surgery/intervention, stroke or transient ischemic attack in past 90 days, or unstable angina pectoris in past 30 days, prior to Visit 1 or during screening; Have NYHA Class I heart failure at either Visit 1 or Visit 2.
14. Dominant contribution of noncardiac causes to exercise impairment or symptoms
    - Lung disease: pulmonary arterial hypertension, chronic thromboembolic pulmonary hypertension (CTEPH), or severe pulmonary disease including (COPD)
    - Other medical conditions: severe anemia (hemoglobin level <9 g/dL) at Visit 1, untreated thyroid disease or TSH >4.78 mU/L at Visit 1, or significant musculoskeletal disease
    - Orthopedic conditions that limit the ability to walk, such as severe arthritis in the leg,

knee, hip injuries, hemiplegia, or amputation with artificial limb without stable prosthesis function for the past 3 months

- Any condition that in the opinion of the investigator would interfere with the assessment of 6MWT

15. LVEF <40% by local echocardiography, MRI or other modalities documented any time within 2 years of Visit 1.
16. Acute decompensated HF (exacerbation of HF) requiring IV diuretics, IV inotropes, or IV vasodilators, or left ventricular assist device (LVAD) within 4 weeks prior to Visit 1, and/or during the screening period until randomization.
17. Impaired renal function, defined as eGFR <15 mL/min/1.73m^2 (CKD-EPI) or requiring dialysis at Visit 1.
18. Any one of the following:
    - Systolic blood pressure (SBP) ≥180 mmHg at Visit 1
    - SBP >160 mmHg both at Visit 1 and at Visit 2
    - Have symptomatic hypotension or SBP <100 mmHg at Visit 1 or Visit 2
19. Resting heart rate (sinus rhythm) ≥100 bpm at either Visit 1 or Visit 2.
20. Atrial fibrillation or atrial flutter with a resting heart rate >110 bpm documented by ECG at either Visit 1 or Visit 2.
21. Cardiac amyloidosis or cardiomyopathy based on accumulation disease (for example, haemochromatosis, Fabry disease), muscular dystrophy, cardiomyopathy with reversible causes (for example, stress cardiomyopathy), hypertrophic cardiomyopathy, Chagas cardiomyopathy or known pericardial constriction, or any severe (obstructive or regurgitant) valvular heart disease likely to lead to surgery during the study period.
22. Completed prior surgical treatment for obesity or had liposuction or abdominoplasty within 1 year prior to Visit 1. Participants who plan to have surgical treatment for obesity

or liposuction or abdominoplasty during the duration of the study are excluded.

23. Participation in a structured exercise training program in the 1 month prior to Visit 1 or planning to start a program during the study.
24. Have T1DM.
25. For participants with T2DM:
    - Have uncontrolled diabetes requiring immediate therapy (such as diabetic ketoacidosis) at Visit 1 or Visit 2, in the judgement of the physician
    - Have had 1 or more events of severe hypoglycemia and/or 1 or more events of hypoglycemia unawareness within 6 months prior to Visit 1
    - Have HbA1c ≥9.5% (80 mmol/mol) at Visit 1, as analyzed at the central laboratory
    - Have a history of proliferative diabetic retinopathy or diabetic maculopathy. Patients with severe nonproliferative diabetic retinopathy that requires acute treatment are also excluded.
    - Treated with premix or prandial insulins or intensified insulin regimens (multiple daily injection with basal and prandial insulins or insulin pump therapy) at Visit 1.
26. History of acute or chronic pancreatitis or at high risk for acute pancreatitis (for example, serum triglyceride level >500 mg/dL [5.65 mmol/L]).
27. Have acute or chronic hepatitis, signs and symptoms of any other liver disease other than nonalcoholic fatty liver disease, or any of the following, as determined by the central laboratory during Visit 1:
    - ALT or AST levels >2.5X the ULN for the reference range.

    Note: Participants with nonalcoholic fatty liver disease are eligible to participate in this trial if their ALT level is ≤3.0X the ULN for the reference range.
28. Have a calcitonin level at Visit 1 of:
    - ≥20 ng/L, if eGFR is ≥60 mL/min/1.73m^2

- ≥35 ng/L, if eGFR is <60 mL/min/1.73m^2

29. Have a family or personal history of medullary thyroid carcinoma (MTC) or Multiple Endocrine Neoplasia (MEN) Syndrome type 2.
30. Have a history of an active or untreated malignancy or are in remission from a clinically significant malignancy (other than basal- or squamous-cell skin cancer, in situ carcinoma of the cervix, or in situ prostate cancer) for less than 5 years.
31. Have a history of any other condition (such as known drug or alcohol abuse, diagnosed eating disorder, or other psychiatric disorder) that, in the opinion of the investigator, may preclude the participant from following and completing the protocol.
32. Have a known clinically significant gastric emptying abnormality (for example, severe diabetic gastroparesis or gastric outlet obstruction) or chronically take drugs that directly affect GI motility.

Prior/Concomitant Therapy

33. Treatment with any incretin, GLP-1 RA, or pramlintide in the 3 months prior to Visit 1.
34. Discontinuation of any incretin, GLP-1 RA, or pramlintide due to intolerability any time prior to Visit 1.
35. Have any other condition not listed in this section (for example, hypersensitivity or intolerance) that is a contraindication to GLP-1 RA.
36. Implantable cardioverter defibrillator (ICD) implantation within 1 month prior to Visit 1 or planned implantation during the course of the study.
37. Currently implanted left ventricular assist device (LVAD).
38. Cardiac resynchronization therapy (CRT) implanted within 6 months prior to Visit 1 or planned implantation during the course of the trial.
39. Current use of medication associated with weight gain or weight loss, except when on stable dose for at least 3 months prior to Visit 1, and expected to be stable during the study period.

Prior/Concurrent Clinical Study Experience

40. Have participated within the last 6 months in a clinical study involving an investigational product.

Other Exclusions

41. Investigator site personnel directly affiliated with this study and/or their immediate families. Immediate family is defined as a spouse, parent, child, or sibling, whether biological or legally adopted.
42. Lilly employees.

APPENDIX 2: Formulas used for estimated glomerular filtration rate (eGFR)

1. *2012 CKD-EPI Cystatin C Equation*

eGFR = 133 x $\min(Scys/0.8, 1)^{-0.499}$ x $\max(Scys/0.8, 1)^{-1.328}$ x 0.996^{Age} x 0.932 [if female]

Abbreviations/units

- eGFR = estimated GFR in $mL/min/1.73m^2$
- Scys = standardized serum cystatin C in mg/L
- min = indicates the minimum of Scys/0.8 or 1
- max = indicates the maximum of Scys/0.8 or 1
- age = years

2. *2021 CKD-EPI Creatinine-cystatin C Equation*

eGFR = 135 × $\min(SCr/\kappa,1)^{\alpha}$ × $\max(SCr/\kappa,1)^{-0.544}$ × $\min(Scys/0.8,1)^{-0.323}$ × $\max(Scys/0.8,1)^{-0.778}$ × 0.9961^{Age} × 0.963 [if female]

Abbreviations/units

- eGFR = estimated GFR in $mL/min/1.73m^2$
- SCr = standardized serum creatinine in mg/dL
- Scys = standardized serum cystatin C in mg/L
- κ = 0.7 (females) or 0.9 (males)
- α = -0.219 (females) or -0.144 (males)
- min = indicates the minimum of SCr/κ or 1
- max = indicates the maximum of SCr/κ or 1
- age = years

3. *2021 CKD-EPI Creatinine Equation*

$$eGFR = 142 \times \min(SCr/\kappa,1)^{\alpha} \times \max(SCr/\kappa,1)^{-1.200} \times 0.9938^{Age} \times 1.012 \text{ [if female]}$$

Abbreviations/units

- eGFR = estimated GFR in mL/min/1.73m^2
- SCr = standardized serum creatinine in mg/dL
- κ = 0.7 (females) or 0.9 (males)
- α = -0.241 (females) or -0.302 (males)
- min = indicates the minimum of SCr/κ or 1
- max = indicates the maximum of SCr/κ or 1
- age = years

SUMMIT Protocol and Statistical Analysis Plan

This supplement contains the following items:

1. Protocol
   a. Original Protocol, dated 13 January 2021
   b. Protocol with amendments, version (a), dated 15 December 2021
   c. Protocol with amendments, version (b), dated 21 January 2022
   d. Final protocol with amendments, version (c), dated 14 February 2024
2. Statistical analysis plan:
   a. Original statistical analysis plan (v1), dated 22 November 2021
   b. Final statistical analysis plan (v2), dated 26 June 2024
3. FDA Briefing Document (inclusive of the target number of events and power calculation)

Title Page

Confidential Information

The information contained in this document is confidential and is intended for the use of clinical investigators. It is the property of Eli Lilly and Company or its subsidiaries and should not be copied by or distributed to persons not involved in the clinical investigation of tirzepatide (LY3298176), unless such persons are bound by a confidentiality agreement with Eli Lilly and Company or its subsidiaries.

Note to Regulatory Authorities: This document may contain protected personal data and/or commercially confidential information exempt from public disclosure. Eli Lilly and Company requests consultation regarding release/redaction prior to any public release. In the United States, this document is subject to Freedom of Information Act (FOIA) Exemption 4 and may not be reproduced or otherwise disseminated without the written approval of Eli Lilly and Company or its subsidiaries.

Protocol Title: A Randomized, Double-Blind, Placebo-Controlled, Phase 3 Study Comparing the Efficacy and Safety of Tirzepatide versus Placebo in Patients with Heart Failure with Preserved Ejection Fraction and Obesity (SUMMIT)

Protocol Number: I8F-MC-GPID

Amendment Number: This is the initial protocol

Compound: LY3298176

Study Phase: 3

Short Title: Tirzepatide vs Placebo in Obesity-related HFpEF

Sponsor Name: Eli Lilly and Company

Legal Registered Address: Indianapolis, Indiana USA 46285

Manufacturer: Eli Lilly and Company

Regulatory Agency Identifier Number(s)

IND: 147352

Approval Date: Protocol Electronically Signed and Approved by Lilly on date provided below.

Approval Date: 13-Jan-2021 GMT

Medical Monitor Name and Contact Information will be provided separately.

Table of Contents

1. Protocol Summary 6
1.1. Synopsis 6
1.2. Schema 9
1.3. Schedule of Activities (SoA) 10

2. Introduction 22
2.1. Study Rationale 22
2.2. Background 22
2.3. Benefit/Risk Assessment 23

3. Objectives and Endpoints 23

4. Study Design 25
4.1. Overall Design 25
4.2. Scientific Rationale for Study Design 27
4.2.1. Patient Input into Design 28
4.3. Justification for Dose 28
4.4. End of Study Definition 29

5. Study Population 30
5.1. Inclusion Criteria 30
5.2. Exclusion Criteria 32
5.3. Lifestyle Considerations 34
5.4. Screen Failures 34

6. Study Intervention 35
6.1. Study Interventions Administered 35
6.1.1. Medical Devices 36
6.2. Preparation/Handling/Storage/Accountability 36
6.3. Measures to Minimize Bias: Randomization and Blinding 37
6.4. Study Intervention Compliance 37
6.5. Concomitant Therapy 38
6.6. Dose Modification 40
6.7. Intervention after the End of the Study 41

7. Discontinuation of Study Intervention and Participant Discontinuation/Withdrawal 41
7.1. Discontinuation of Study Intervention 41
7.1.1. Permanent Discontinuation from Study Drug 41
7.1.2. Temporary Interruption 43
7.1.3. Restarting Study Drug after Interruption 43
7.2. Participant Discontinuation/Withdrawal from the Study 44
7.2.1. Inadvertently Enrolled Participants 44
7.3. Lost to Follow up 45

8. Study Assessments and Procedures 45
8.1. Efficacy Assessments 45
8.1.1. Primary Efficacy Assessment 45
8.1.2. Additional Secondary Efficacy Assessments 47

8.1.3. Exploratory Efficacy Assessments 47
8.2. Safety Assessments 48
8.2.1. Physical Examinations 48
8.2.2. Vital Signs 48
8.2.3. Electrocardiograms 49
8.2.4. Clinical Safety Laboratory Assessments 49
8.2.5. Safety Monitoring 50
8.3. Adverse Events, Serious Adverse Events, and Product Complaints 52
8.3.1. Timing and Mechanism for Collecting Events 53
8.3.2. Primary, Secondary, and Additional Study Endpoint Reporting 56
8.3.3. Adverse Events of Special Interest 56
8.4. Treatment of Overdose 57
8.5. Pharmacokinetics 57
8.6. Pharmacodynamics 57
8.7. Genetics 57
8.8. Biomarkers 57
8.9. Immunogenicity Assessments 58
8.10. Medical Resource Utilization and Health Economics 58

9. Statistical Considerations 58
9.1. Statistical Hypotheses 58
9.2. Sample Size Determination 59
9.3. Populations for Analyses 60
9.4. Statistical Analyses 60
9.4.1. General Considerations 60
9.4.2. Primary Endpoint(s) 61
9.4.3. Key Secondary Endpoint(s) 62
9.4.4. Tertiary/Exploratory Endpoint(s) 62
9.4.5. Other Safety Analyses 62
9.4.6. Subgroup Analyses 63
9.5. Interim Analyses 63
9.6. Data Monitoring Committee 63

10. Supporting Documentation and Operational Considerations 63
10.1. Appendix 1: Regulatory, Ethical, and Study Oversight Considerations 63
10.1.1. Regulatory and Ethical Considerations 63
10.1.2. Financial Disclosure 64
10.1.3. Informed Consent Process 64
10.1.4. Data Protection 65
10.1.5. Dissemination of Clinical Study Data 65
10.1.6. Data Quality Assurance 66
10.1.7. Source Documents 67
10.1.8. Study and Site Start and Closure 67
10.1.9. Publication Policy 68
10.1.10. Long-Term Sample Retention 68
10.2. Appendix 2: Clinical Laboratory Tests 69

10.3. Appendix 3: Adverse Events: Definitions and Procedures for Recording, Evaluating, Follow-up, and Reporting....72
10.3.1. Definition of AE....72
10.3.2. Definition of SAE....73
10.3.3. Definition of Product Complaints....75
10.3.4. Recording and Follow-Up of AE and/or SAE and Product Complaints....75
10.3.5. Reporting of SAEs....77
10.3.6. Regulatory Reporting Requirements....77
10.4. Appendix 4: Contraceptive Guidance and Collection of Pregnancy Information....78
10.4.1. Male participants:....78
10.4.2. Female participants:....78
10.5. Appendix 5: Adverse Events of Special Interest: Definitions and Procedures for Recording, Evaluating, Follow-Up, and Reporting....82
10.5.1. Special Safety Topics....82
10.6. Appendix 6: Genetics....87
10.7. Appendix 7: Recommended Laboratory Testing for Hypersensitivity Events....88
10.8. Appendix 8: Liver Safety: Suggested Actions and Follow-Up Assessments....90
10.9. Appendix 9: Medical Device Adverse Events (AEs), Adverse Device Effects (ADEs), Serious Adverse Events (SAEs) and Device Deficiencies: Definition and Procedures for Recording, Evaluating, Follow-Up, and Reporting....92
10.10. Appendix 10: Six-Minute Walk Test....93
10.10.1. Screening Procedures and Flow Diagram....93
10.11. Appendix 11: New York Heart Association Classification of Heart Failure....94
10.12. Appendix 12: Abbreviations....95
11. References....100

1. Protocol Summary

1.1. Synopsis

Protocol Title: A Randomized, Double-Blind, Placebo-Controlled, Phase 3 Study Comparing the Efficacy and Safety of Tirzepatide versus Placebo in Patients with Heart Failure with Preserved Ejection Fraction and Obesity (SUMMIT)

Short Title: Tirzepatide vs Placebo in Obesity-related HFpEF

Rationale:

Heart failure with preserved ejection fraction is a heterogenous clinical syndrome resulting from various pathophysiological processes. Among the broad spectrum of HFpEF clinical presentation, obesity-related HFpEF displays a distinct phenotype where increased visceral and ectopic adiposity as well as volume expansion plays a causal role (Kitzman and Shah 2016; Packer 2018; Miller and Borlaug 2020). Given tirzepatide's anti-inflammatory and antifibrotic effects and a reduction in circulating plasma volume as a consequence of the treatment of obesity, tirzepatide may provide clinical benefit to patients with HFpEF and BMI ≥30 kg/m^2.

Study I8F-MC-GPID, also known as SUMMIT, is a Phase 3, randomized, multicenter, international, placebo-controlled, double-blinded, parallel-arm study. This study will evaluate the effect of SC QW injection of tirzepatide, MTD up to 15 mg, on the risk of death, hospitalization or emergency care due to HF, exercise capacity, and health status in participants with HFpEF and BMI ≥30 kg/m^2.

Objectives and Endpoints

Objectives	Endpoints
Primary	
To demonstrate that a maximally tolerated tirzepatide dose up to 15 mg administered subcutaneously once weekly (SC QW) is superior to placebo based on the hierarchal composite endpoint in participants with HFpEF and BMI ≥30 kg/m^2.	A hierarchical composite of the following:<br>1. Time to all-cause mortality through the end of the treatment period<br>2. Occurrence of heart failure (HF) events through end of the treatment period, where HF events include HF hospitalization OR urgent HF visit (adjudicated)<br>o number of HF events<br>o time to first HF events<br>3. Change from baseline in the 6-minute walk test distance (6MWD) category at Week 52

	4. Change from baseline in the Kansas City Cardiomyopathy Questionnaire (KCCQ) Clinical Summary Scale (CSS) category at Week 52<br><br>The categories for change from baseline in the 6MWD are: 1) ≥30% worsening; 2) ≥20% and <30% worsening; 3) ≥10% and <20% worsening; 4) No change (<10% change); 5) ≥10% and <20% improvement; 6) ≥20% and <30% improvement; and 7) ≥30% improvement.<br><br>The categories for change from baseline in the KCCQ-CSS are: 1) ≥10-point worsening; 2) ≥5- but <10-point worsening; 3) No change (<5-point change); 4) ≥5- but <10-point improvement; and 5) ≥10-point improvement.
To demonstrate that a maximally tolerated tirzepatide dose up to 15 mg administered SC QW is superior to placebo to improve exercise capacity in participants with HFpEF and BMI ≥30 kg/m^2.	Change from baseline to Week 52 in exercise capacity as measured 6MWD
Key Secondary (multiplicity controlled)	
Long-term weight loss	Percent change from baseline to Week 52 in body weight loss
Patient-reported symptoms and physical limitations	Change from baseline to Week 52 in the KCCQ CSS
Exercise capacity	Change from baseline to Week 24 in 6MWD
New York Heart Association (NYHA) Class	Proportion of participants with NYHA Class change at Week 52

Abbreviations: BMI = body mass index; HFpEF = heart failure with preserved ejection fraction.

Overall Design

Study GPID is a randomized, outpatient, multicenter, international, placebo-controlled, double-blind, parallel-arm, Phase 3 study with 2 study periods. The study is designed to evaluate the efficacy and safety of SC QW tirzepatide, MTD up to 15 mg, compared to placebo, in participants with HFpEF and obesity.

Two intervention groups will be studied:

- Tirzepatide, MTD up to 15 mg, SC QW
- Placebo

The starting dose of tirzepatide is 2.5 mg QW, which is to be escalated at 4-week intervals to a maximum of 15 mg QW or to the highest maintenance dose tolerated by the participant (see Section 6).

The maximum duration of participation depends on when the last participant completes 52 weeks of treatment.

Disclosure Statement: This is a parallel-treatment study with 2 intervention groups that is double blinded.

Number of Participants:

Approximately 700 participants will be randomly assigned to study drug with approximately 350 participants per intervention group.

Intervention Groups and Duration:

The study will compare treatment with tirzepatide and treatment with placebo. Assignment to tirzepatide or placebo groups will be randomly allocated in a 1:1 ratio.

The starting dose of tirzepatide 2.5 mg QW is to be escalated to 15 mg QW or the highest maintenance dose tolerated by the participant (5 mg, 10 mg, or 15 mg).

Study participation for each participant is sectioned into the following study periods:

- Study Period 1: screening period, approximately 6 weeks
- Study Period 2: treatment period, at least 52 weeks

The study will continue until approximately 52 weeks after the last participant is randomized. The maximum duration of an individual's participation is estimated to be 120 weeks and will depend on duration of study enrollment.

Data Monitoring Committee: Yes

1.2. Schema

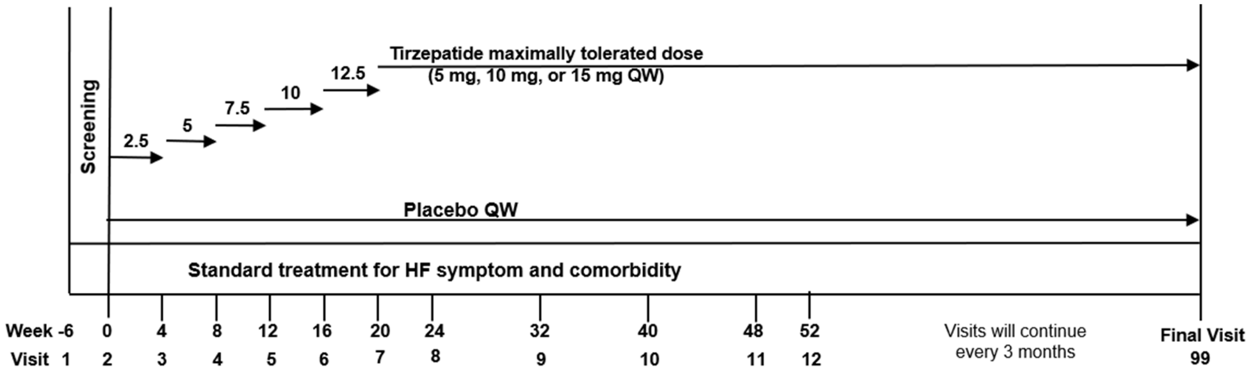

Abbreviations: HF = heart failure; QW = weekly.

Note: Screening procedures may take longer or shorter than 6 weeks and variation in screening procedures will not be considered a protocol deviation.

1.3. Schedule of Activities (SoA)

Visit 1 and 2 procedures may be conducted over more than 1 day each as long as all activities are completed within the allowable visit tolerance for each visit.

For early terminations (ET) from the study that occur before the last visit in treatment period, see the activities listed for ET in this table.

Study I8F-MC-GPID	Study Period I Screening	Study Period II - Treatment period															
Visit number	1	2	3	4	5	6	7	8	9	10	11	12	EVa, EVb, EVc (13, 14, 15, 17, 18, 19, etc.)	EVd (16, 20, etc.)	ET	99	Comment
Weeks from randomization	-6	0	4	8	12	16	20	24	32	40	48	52	(+3, 6, 9, 15, 18, 21 months)	(+12, 24 months)	—	Final Visit	See footnote a
Visit interval tolerance (days)			±3	±3	±3	±3	±3	±3	±7	±7	±7	±7	±15	±15	—		
Telephone Visit									X		X		X				See footnote b.
Informed consent	X																
Inclusion and exclusion criteria, review and confirm	X	X															
Demographics	X																
Preexisting conditions and medical history, including relevant surgical history	X																
Prespecified medical history (indication and history of interest)	X																Includes HF history, hospitalization for HF, CVD, MI, atrial fibrillation, stroke, CV risk (T2DM, HTN, dyslipidemia, metabolic syndrome)
Prior treatments for HFpEF	X																

Study I8F-MC-GPID	Study Period I Screening	Study Period II - Treatment period															
Visit number	1	2	3	4	5	6	7	8	9	10	11	12	EVa, EVb, EVc (13, 14, 15, 17, 18, 19, etc.)	EVd (16, 20, etc.)	ET	99	Comment
Weeks from randomization	-6	0	4	8	12	16	20	24	32	40	48	52	(+3, 6, 9, 15, 18, 21 months)	(+12, 24 months)	—	Final Visit	See footnote a
Visit interval tolerance (days)			±3	±3	±3	±3	±3	±3	±7	±7	±7	±7	±15	±15	—		
Telephone Visit									X		X		X				See footnote b.
Substance use (alcohol, tobacco use)	X																
Concomitant medications	X	X	X	X	X	X	X	X	X	X	X	X	X	X	X	X	
Adverse events (AEs)	X	X	X	X	X	X	X	X	X	X	X	X	X	X	X	X	Including Product Complaints
**Physical Evaluation**																	
Height	X																
Weight	X	X	X	X	X	X	X	X		X		X		X	X	X	
Waist circumference	X	X						X				X		X	X	X	
Vital Signs	X	X	X	X	X	X	X	X		X		X		X	X	X	Include 2 Sitting BP and HR. HR to be performed by apical auscultation. Vital signs should be collected prior to the first 6MWT of the day and before ECG.
Physical examination	X											X		X	X		

Study I8F-MC-GPID	Study Period I Screening	Study Period II - Treatment period														
Visit number	1	2	3	4	5	6	7	8	9	10	11	12	EVa, EVb, EVc (13, 14, 15, 17, 18, 19, etc.)	EVd (16, 20, etc.)	ET	99	Comment
Weeks from randomization	-6	0	4	8	12	16	20	24	32	40	48	52	(+3, 6, 9, 15, 18, 21 months)	(+12, 24 months)	—	Final Visit	See footnote a
Visit interval tolerance (days)			±3	±3	±3	±3	±3	±3	±7	±7	±7	±7	±15	±15	—		
Telephone Visit									X		X		X				See footnote b.
Symptom-directed physical examination		X	X	X	X	X	X	X		X						X	As indicated based on participant status and standard of care, including dyspnea, orthopnea, paroxysmal nocturnal dyspnea (PND), edema, jugular venous distension (JVD), rales
NYHA class assessment	X	X						X				X		X	X*		NYHA class assessment must be performed by an independent assessor.<br>*Perform at ET only if participant early terminates at or prior to Week 52.
HF events		X	X	X	X	X	X	X	X	X	X	X	X	X	X	X	
Evaluation of injection site reactions		X	X	X	X	X	X	X		X		X		X	X	X	
Single-read 12-lead ECG	X	X						X				X			X*		Collect locally. Report atrial fibrillation or other abnormalities on the eCRF. Optional ECG is allowable if indicated.

Study I8F-MC-GPID	Study Period I Screening	Study Period II - Treatment period														
Visit number	1	2	3	4	5	6	7	8	9	10	11	12	EVa, EVb, EVc (13, 14, 15, 17, 18, 19, etc.)	EVd (16, 20, etc.)	ET	99	Comment
Weeks from randomization	-6	0	4	8	12	16	20	24	32	40	48	52	(+3, 6, 9, 15, 18, 21 months)	(+12, 24 months)	—	Final Visit	See footnote a
Visit interval tolerance (days)			±3	±3	±3	±3	±3	±3	±7	±7	±7	±7	±15	±15	—		
Telephone Visit									X		X		X				See footnote b.
																	*Perform at ET only if participant early terminates at or prior to Week 52.
Echocardiography	X																For those required to complete the ECHO examination;<br>Perform after results received for NT-proBNP, if applicable.
Dilated fundoscopic examination	X																Perform for participants with T2DM who have not had an evaluable dilated fundoscopic examination in the last 12 months. See exclusion criterion 25 (Section 5.2)<br>Follow-up dilated fundoscopic examination should be performed when clinically indicated by any AE suspected of worsening retinopathy.
6MWT	X*	X **						X				X			X***		Ensure that participant completes the associated Borg Questionnaire prior to and after the 6MWT.

Study I8F-MC-GPID	Study Period I Screening	Study Period II - Treatment period															
Visit number	1	2	3	4	5	6	7	8	9	10	11	12	EVa, EVb, EVc (13, 14, 15, 17, 18, 19, etc.)	EVd (16, 20, etc.)	ET	99	Comment
Weeks from randomization	-6	0	4	8	12	16	20	24	32	40	48	52	(+3, 6, 9, 15, 18, 21 months)	(+12, 24 months)	—	Final Visit	See footnote a
Visit interval tolerance (days)			±3	±3	±3	±3	±3	±3	±7	±7	±7	±7	±15	±15	—		
Telephone Visit									X		X		X				See footnote b.
																	*Two 6MWTs conducted at screening visit.<br>**See Section 10.10.1 to determine if the 6MWT needs to be repeated for Visit 2.<br>***Perform at ET only if participant early terminates at or prior to Week 52.
**Participant Education**																	
Diary instruction		X															
**Participant Diary**																	
Participant diary dispensed		X	X	X	X	X	X	X		X		X		X			
Diary compliance check			X	X	X	X	X	X	X	X	X	X	X	X	X	X	
Diary return			X	X	X	X	X	X		X		X		X	X	X	
**Patient-Reported Outcomes (PROs) (Electronic)***																	*Perform PRO at ET only if participant early terminates at or prior to Week 52.

Study I8F-MC-GPID	Study Period I Screening	Study Period II - Treatment period															
Visit number	1	2	3	4	5	6	7	8	9	10	11	12	EVa, EVb, EVc (13, 14, 15, 17, 18, 19, etc.)	EVd (16, 20, etc.)	ET	99	Comment
Weeks from randomization	-6	0	4	8	12	16	20	24	32	40	48	52	(+3, 6, 9, 15, 18, 21 months)	(+12, 24 months)	—	Final Visit	See footnote a
Visit interval tolerance (days)			±3	±3	±3	±3	±3	±3	±7	±7	±7	±7	±15	±15	—		
Telephone Visit									X		X		X				See footnote b.
Kansas City Cardiomyopathy Questionnaire (KCCQ)	X	X						X				X			X		
EQ-5D-5L		X						X				X			X		
Patient Global Impression of Status – Overall (PGIS-Overall)	X	X						X				X			X		
Patient Global Impression of Status – Physical Function (PGIS-Physical Function)	X	X						X				X			X		
Patient Global Impression of Status – Symptom Severity (PGIS-Symptom Severity)	X	X						X				X			X		
**Laboratory Tests and Sample Collections**																	
Hematology	X	X			X			X				X		X	X	X	

Study I8F-MC-GPID	Study Period I Screening	Study Period II - Treatment period														
Visit number	1	2	3	4	5	6	7	8	9	10	11	12	EVa, EVb, EVc (13, 14, 15, 17, 18, 19, etc.)	EVd (16, 20, etc.)	ET	99	Comment
Weeks from randomization	-6	0	4	8	12	16	20	24	32	40	48	52	(+3, 6, 9, 15, 18, 21 months)	(+12, 24 months)	—	Final Visit	See footnote a
Visit interval tolerance (days)			±3	±3	±3	±3	±3	±3	±7	±7	±7	±7	±15	±15	—		
Telephone Visit									X		X		X				See footnote b.
Hemoglobin A1c (HbA1c)	X	X	X	X	X	X	X	X		X		X		X	X	X	
Clinical chemistry (with glucose)	X	X *			X			X				X		X	X	X	*Required at Visit 2 randomization if screening labs are older than 28 days for chemistry, lipid, and pancreatic lipase/amylase
Lipid panel	X	X *			X			X				X		X	X	X	*Required at Visit 2 randomization if screening labs are older than 28 days for chemistry, lipid, and pancreatic lipase/amylase
Thyroid-stimulating hormone (TSH)	X																
Serum pregnancy	X	X *															For women of childbearing potential only. See Appendix 4 (Section 10.4). *Collect serum pregnancy at Visit 2 only if Visit 1 serum was ≥28 days prior.

Study I8F-MC-GPID	Study Period I Screening	Study Period II - Treatment period															
Visit number	1	2	3	4	5	6	7	8	9	10	11	12	EVa, EVb, EVc (13, 14, 15, 17, 18, 19, etc.)	EVd (16, 20, etc.)	ET	99	Comment
Weeks from randomization	-6	0	4	8	12	16	20	24	32	40	48	52	(+3, 6, 9, 15, 18, 21 months)	(+12, 24 months)	—	Final Visit	See footnote a
Visit interval tolerance (days)			±3	±3	±3	±3	±3	±3	±7	±7	±7	±7	±15	±15	—		
Telephone Visit									X		X		X				See footnote b.
Urine pregnancy (local)		X						X				X		X	X	X	A local urine pregnancy test must be performed at Visit 2 after patient eligibility has been confirmed with the result available prior to randomization and first injection of study drug(s) for WOCBP only. Additional local urine pregnancy tests may be performed at the investigator's discretion during the study. If required per local regulations and/or institutional guidelines, pregnancy testing can also occur at other times during the study treatment period. See Appendix 4 (Section 10.4).
Follicle-stimulating hormone (FSH)	X																Collect FSH only in women whose menopausal status needs to be determined. For participants known to be either premenopausal or postmenopausal, these tests do not need to be collected

Study I8F-MC-GPID	Study Period I Screening	Study Period II - Treatment period														
Visit number	1	2	3	4	5	6	7	8	9	10	11	12	EVa, EVb, EVc (13, 14, 15, 17, 18, 19, etc.)	EVd (16, 20, etc.)	ET	99	Comment
Weeks from randomization	-6	0	4	8	12	16	20	24	32	40	48	52	(+3, 6, 9, 15, 18, 21 months)	(+12, 24 months)	—	Final Visit	See footnote a
Visit interval tolerance (days)			±3	±3	±3	±3	±3	±3	±7	±7	±7	±7	±15	±15	—		
Telephone Visit									X		X		X				See footnote b.
NT-proBNP	X	X			X			X				X			X*		*Perform at ET only if participant early terminates at or prior to Week 52.
Cardiac troponin T (cTnT)		X			X			X				X			X*		*Perform at ET only if participant early terminates at or prior to Week 52.
Calcitonin	X							X				X		X	X	X	
Cystatin C		X			X			X				X			X*		*Perform at ET only if participant early terminates at or prior to Week 52.
C-reactive protein, high-sensitivity (hs-CRP)		X			X			X				X			X*		*Perform at ET only if participant early terminates at or prior to Week 52.
Pancreatic amylase	X	X *			X			X		X		X		X	X	X	*Required at Visit 2 randomization if screening labs are older than 28 days for chemistry, lipid, and pancreatic lipase/amylase
Lipase	X	X *			X			X		X		X		X	X	X	*Required at Visit 2 randomization if screening labs are older than 28 days for chemistry, lipid, and pancreatic lipase/amylase

Study I8F-MC-GPID	Study Period I Screening	Study Period II - Treatment period															
Visit number	1	2	3	4	5	6	7	8	9	10	11	12	EVa, EVb, EVc (13, 14, 15, 17, 18, 19, etc.)	EVd (16, 20, etc.)	ET	99	Comment
Weeks from randomization	-6	0	4	8	12	16	20	24	32	40	48	52	(+3, 6, 9, 15, 18, 21 months)	(+12, 24 months)	—	Final Visit	See footnote a
Visit interval tolerance (days)			±3	±3	±3	±3	±3	±3	±7	±7	±7	±7	±15	±15	—		
Telephone Visit									X		X		X				See footnote b.
eGFR (CKD-EPI)	X	X			X			X				X		X	X	X	The CKD-EPI equation will be used by the central lab to estimate and report eGFR.
Urinary albumin/creatinine ratio (UACR)	X							X				X		X	X	X	
Pharmacokinetic (PK) samples		X	X		X			X				X		X	X	X	PK samples should be taken prior to dose administration at the visit and at the same time as immunogenicity samples.
Immunogenicity samples		X	X		X			X				X		X	X	X	
**Stored Samples**																	
Genetics sample		X															
Exploratory biomarker samples		X			X			X				X					
**Randomization and Dosing**																	
Randomization		X															

Study I8F-MC-GPID	Study Period I Screening	Study Period II - Treatment period														
Visit number	1	2	3	4	5	6	7	8	9	10	11	12	EVa, EVb, EVc (13, 14, 15, 17, 18, 19, etc.)	EVd (16, 20, etc.)	ET	99	Comment
Weeks from randomization	-6	0	4	8	12	16	20	24	32	40	48	52	(+3, 6, 9, 15, 18, 21 months)	(+12, 24 months)	—	Final Visit	See footnote a
Visit interval tolerance (days)			±3	±3	±3	±3	±3	±3	±7	±7	±7	±7	±15	±15	—		
Telephone Visit									X		X		X				See footnote b.
Dispense study drug		X	X	X	X	X	X	X		X		X	X	X			For EVa, EVb, and EVc, the participant receives study drug via carrier.
Injection training with autoinjector demonstration device		X															
Observe participant administer study drug		X															Participants should administer the first dose of study drug at Visit 2 after study procedures and randomization have been completed. Sites should coach and oversee if participants self-administer study drug at a scheduled visit.
Dispense ancillary supplies to participant		X	X	X	X	X	X	X		X				X			
Participant returns study drugs and injection supplies			X	X	X	X	X	X		X		X		X	X	X	
Assess study drug compliance			X	X	X	X	X	X	X	X	X	X	X	X	X	X	

Abbreviations: 6MWT = 6-minute walk test; BP = blood pressure; CKD-EPI = Chronic Kidney Disease Epidemiology Collaboration; CVD = cardiovascular disease; ECG = electrocardiogram; ECHO = echocardiography; eCRF = electronic case report form; eGFR = estimated glomerular filtration rate; EQ-5D-5L = 5-Level European Quality of Life Questionnaire; ET = Early Termination; EV = Extended Visit; HF = heart failure; HFpEF = heart failure with preserved ejection fraction; HR = heart rate; HTN = hypertension; MI = myocardial infarction; NT-proBNP = N-terminal pro b-type natriuretic peptide; NYHA = New York Heart Association; T2DM = type 2 diabetes mellitus; WOCBP = women of childbearing potential.

a At least 52 weeks (Visit 12) of treatment are planned. Additional visits after Visit 12 will occur every 3 months. The visits occurring after Visit 12 will follow the Extended Maintenance Visit schedule in sequence (EVa, EVb, EVc, and EVd) then repeat.

b Telephone visits can become office visits. Site documentation will serve as the source for telephone visits. Additional, optional telephone visits may be conducted at investigator discretion.

2. Introduction

2.1. Study Rationale

Heart failure with preserved ejection fraction is a heterogenous clinical syndrome resulting from various pathophysiological processes. Among the broad spectrum of HFpEF clinical presentation, obesity-related HFpEF displays a distinct phenotype where increased visceral and ectopic adiposity as well as volume expansion plays a causal role (Kitzman and Shah 2016; Packer 2018; Miller and Borlaug 2020). Given tirzepatide's potential to decrease inflammation and fibrosis and a to reduce circulating plasma volume as a consequence of the treatment of obesity, tirzepatide may provide clinical benefit to patients with HFpEF and BMI ≥30 kg/m^2.

Study I8F-MC-GPID, also known as SUMMIT, is a Phase 3, randomized, multicenter, international, placebo-controlled, double-blinded, parallel-arm study. This study will evaluate the effect of SC QW injection of tirzepatide, MTD up to 15 mg, on the risk of death, hospitalization or emergency care due to HF, exercise capacity, and health status in participants with HFpEF and BMI ≥30 kg/m^2.

2.2. Background

There is a significant unmet need in treatment of patients with HFpEF. Tirzepatide, a GIP and GLP-1 dual agonist, has the potential to provide benefit to patients with HFpEF and BMI ≥30 kg/m^2 with not only improvements in exercise capacity and symptoms but also a reduction in HF events and/or increased survival. Supporting a causal association between obesity and HFpEF, bariatric surgery improved NYHA class, patient-reported outcomes, and echo parameters (LV wall thickness, LV relaxation) in patients with HFpEF and obesity (Mikhalkova et al. 2018). A meta-analysis of bariatric surgery also showed improvement in functional capacity 6 to 12 months after surgery in patients with obesity (Herring et al. 2016). In patients with HFpEF and obesity, diet-induced weight loss (Δ=−7 kg, 20 weeks) significantly improved symptoms (KCCQ) and exercise capacity (6MWD and peak oxygen uptake) (Kitzman et al. 2016). Furthermore, weight reduction is proven to be effective in reducing HF risk and HF hospitalizations. A large observational study has demonstrated a 62% decrease (over 8 years) of HF incidence after bariatric surgery in patients with T2DM (Aminian et al. 2019). The reduction of HF risk after bariatric surgery has been consistently demonstrated in broader patient populations and considered to be mediated by weight loss with a hazard ratio for a 10-kg weight loss being 0.77 (Sundström et al. 2017; Jamaly et al. 2019). Moreover, a self-controlled case study showed a 29% (0 to 12 months) and a 43% (13 to 24 months) risk reduction of HF events in patients with HFpEF after bariatric surgery (Shimada et al. 2016).

It has been demonstrated that tirzepatide can provide significant body weight loss and improvement of lipid and glucose metabolism in patients with T2DM (Frias et al. 2018; Wilson et al. 2020). It is known that the body weight reduction with GLP-1 RAs in patients without T2DM is higher than in patients with T2DM (Davies et al. 2015; Pi-Sunyer et al. 2015; Lingvay et al. 2018). If Study GPID is assumed to include 40% to 50% of patients with T2DM, the mean placebo-adjusted body weight percent reduction that tirzepatide can provide in 52 weeks in this study is estimated to be 15% to 16%. This is based on tirzepatide clinical data and the

understanding of body weight loss differences between patients with and without T2DM treated with GLP-1 RAs. Thus, the predicted body weight loss with treatment with tirzepatide is close to that shown with bariatric surgery. In addition, tirzepatide may provide benefit to patients with HFpEF and obesity by virtue of cardiometabolic improvements (Wilson et al. 2020). Given the wide distribution of GIP receptor in the adipose tissue, GIP is thought to be actively involved in lipid and glucose metabolism.

Therefore, it will be meaningful to assess HF clinical outcomes in addition to the functional and symptomatic endpoints in this study, thereby facilitating the understanding of the clinical benefit of tirzepatide treatment in patients with HFpEF and obesity.

2.3. Benefit/Risk Assessment

More detailed information about the known and expected benefits and risks and reasonably expected AEs of tirzepatide may be found in the IB.

3. Objectives and Endpoints

Objectives	Endpoints
Primary	
To demonstrate that a maximally tolerated tirzepatide dose up to 15 mg administered subcutaneously once weekly (SC QW) is superior to placebo based on the hierarchal composite endpoint in participants with HFpEF and BMI ≥30 kg/m2.	A hierarchical composite of the following:<br>1. Time to all-cause mortality through the end of the treatment period<br>2. Occurrence of heart failure (HF) events through end of the treatment period, where HF events include HF hospitalization OR urgent HF visit (adjudicated)<br>o number of HF events<br>o time to first HF event<br>3. Change from baseline in the 6-minute walk test distance (6MWD) category at Week 52<br>4. Change from baseline in the Kansas City Cardiomyopathy Questionnaire (KCCQ) Clinical Summary Score (CSS) category at Week 52<br><br>The categories for change from baseline in the 6MWD score are: 1) ≥30% worsening; 2) ≥20% and <30% worsening; 3) ≥10% and <20% worsening; 4) No change (<10% change); 5) ≥10% and <20% improvement;

	6) ≥20% and <30% improvement; and<br>7) ≥30% improvement.<br>The categories for change from baseline in the KCCQ-CSS are: 1) ≥10-point worsening;<br>2) ≥5- but <10-point worsening; 3) No change (<5-point change); 4) ≥5- but <10-point improvement; and 5) ≥10-point improvement.
To demonstrate that a maximally tolerated tirzepatide dose up to 15 mg administered SC QW is superior to placebo to improve exercise capacity in participants with HFpEF and BMI ≥30 kg/m^2.	Change from baseline to Week 52 in exercise capacity as measured by 6MWD
Key Secondary (multiplicity controlled)	
Long-term weight loss	Percent change from baseline to Week 52 in body weight loss
Patient-reported symptoms and physical limitations	Change from baseline to Week 52 in the KCCQ CSS
Exercise capacity	Change from baseline to Week 24 in 6MWD
New York Heart Association (NYHA) Class	Proportion of participants with NYHA Class change at Week 52
Exploratory	
HF medication use	Change in the HF concomitant medication net use
Clinical outcome events of HF	Incidence of<br>• HF events<br>• Death
Atrial fibrillation	Change in proportion of participants with atrial fibrillation from baseline
Waist circumference	Change from baseline (centimeters)
Patient-reported health-related quality of life	Change from baseline in KCCQ:<br>• Total Symptom Score (TSS)<br>• Overall Summary Score (OSS)

Patient-reported health status	Change from baseline in EQ-5D-5L:<br>o Index Score<br>o VAS Score
Patient-reported global health status	Proportion of participants with improvements in global health status from baseline as assessed by the PGIS-Overall
Patient-reported global impression of physical function	Proportion of participants with improvements in physical function from baseline as assessed by the PGIS-Physical Function
Patient-reported global symptom severity	Proportion of participants with improvements in symptom severity from baseline as assessed by the PGIS-Symptom Severity
Evaluation of prespecified biomarkers	• NT-proBNP<br>• cTnT<br>• hs-CRP

Abbreviations: BMI = body mass index; cTnT = cardiac troponin T; EQ-5D-5L = 5-Level European Quality of Life Questionnaire; HFpEF = heart failure with preserved ejection fraction; hsCRP = C-reactive protein, high-sensitivity; NT-proBNP = N-terminal pro b-type natriuretic peptide; PGIS = Patient-Reported Global Impression of Status; VAS = visual analog scale.

4. Study Design

4.1. Overall Design

Study GPID is a randomized, outpatient, multicenter, international, placebo-controlled, double-blinded, parallel-arm, Phase 3 study with 2 study periods. The study is designed to evaluate the efficacy and safety of SC QW tirzepatide, MTD up to 15 mg, compared to placebo, in participants with HFpEF and obesity.

Two intervention groups will be studied:

- Tirzepatide MTD up to 15 mg SC QW
- Placebo

The study will compare treatment with tirzepatide and treatment with placebo. Assignment to tirzepatide or placebo groups will be randomly allocated in a 1:1 ratio.

The starting dose of tirzepatide is 2.5 mg QW, which is to be escalated at 4-week intervals to a maximum of 15 mg QW or to the highest maintenance dose tolerated by the participant (see Section 6).

The study will consist of 2 periods:

- Study Period 1: screening period, up to approximately 6 weeks
- Study Period 2: treatment period, with a 20-week escalation followed by at least a 32-week maintenance period

The study will continue until approximately 52 weeks after the last participant is randomized. The maximum duration of an individual's participation is estimated to be 120 weeks and will depend on duration of study enrollment.

Participant Visit Scheme

Study participants will undergo screening assessments and procedures, randomization, and double-blinded treatment with tirzepatide or placebo. Assessments and procedures to be conducted in each treatment period are described in the SoA (Section 1.3) and in Study Assessments and Procedures (Section 8).

Screening

Screening procedures will be performed at Visit 1. Visit 1 procedures may be conducted over more than 1 day as long as all activities are completed within the allowable visit tolerance for each visit.

At Visit 1, two 6MWTs will be conducted. The investigator must ensure that the participant is recovered from completion of the first 6MWT prior to conducting the second 6MWT (at least 1 hour between each test). The screening 6MWTs may be conducted over more than 1 day.

Randomization

At Visit 2, prior to randomization, the participant needs to complete the 6MWT. Visit 2 may need to be conducted over 2 in-clinic visits (considered Visit 2a and Visit 2b) if a repeat 6MWT is necessary to assess participant eligibility. If the participant is required to return to repeat the 6MWT, the remaining procedures should be conducted at the second in-clinic visit for randomization (Visit 2b), more than 10 days from Visit 2a. See Section 10.10.1 for details on when a participant must return for a second Visit 2 6MWT.

Participants will be randomized and receive study drug at the end of Visit 2 after all screening procedures are completed. The participant must not receive study drug until all eligibility criteria, including the 6MWT, are met.

Treatment

Starting from randomization, the participant receives study drug and procedures are conducted as described in the SoA (Section 1.3).

Participants will continue into the extended maintenance period starting with Visit 13. Extended visits (EV) continue until criteria for study discontinuation is met or study ends (see Study Closeout and Final Visit below).

Tirzepatide dose will be escalated as illustrated in the study schema (Section 1.2).

If a participant discontinues study drug (Section 7.1), all study procedures will continue to be performed as described in the SoA (Section 1.3). Participants who discontinue or withdraw from the study (see Section 7.2) will undergo an ET visit as described in the SoA (Section 1.3).

Study Closeout and Final Visit

The study will continue until approximately 52 weeks after the final participant is randomized. A study duration of approximately 30 months is planned.

Approximately 3 months prior to the anticipated end of study, a study closeout will be declared. During the study closeout, a final visit (Visit 99) will be planned for each participant, with the exception of those who have died or prematurely discontinued from the study (Section 7.2). Study procedures for the final visit will be performed as outlined in the SoA (Section 1.3).

All unused study drug (unused single-dose pens) must be returned for compliance and final drug accountability. The sharp items container should also be returned to site or disposed of per local regulations.

4.2. Scientific Rationale for Study Design

Study GPID is a Phase 3 study designed to examine the efficacy and safety of SC QW tirzepatide MTD compared with placebo in participants with HFpEF and BMI ≥30kg/m^2.

A placebo comparator was selected for this trial in accordance with regulatory guidance (FDA 2007; EMA 2016). Inclusion of a placebo comparator in Study GPID will allow for a direct assessment of the safety and efficacy of tirzepatide in participants with HFpEF and obesity.

Additionally, there is currently no approved therapy to be used as an active comparator in this population.

An endpoint assessment at 52 weeks of treatment is considered appropriate to assess the improvement of symptom and functional capacity. An extended maintenance treatment period increases the opportunity to evaluate HF events and outcomes.

The parallel-group design for treatment comparison was chosen to avoid any interaction between treatments that may interfere with the interpretation of the trial outcome. To minimize potential confounding effect of changes to concomitant medications, participants will be permitted to use the stable dose of concomitant medications that they require during the study. Medications that may interfere with the assessment of efficacy and safety characteristics of the study drug will not be allowed (see Section 6.5).

The primary endpoints were selected to assess the clinical benefit that tirzepatide could provide to patients in a holistic manner.

Assessment of HF events is relevant to HFpEF, which is characterized by a high frequency of recurrent HF hospitalizations. Moreover, hospitalization events reflect disease progression and high subsequent risk and predisposition, both of readmission and death (Solomon et al. 2007).

The conventional reporting of HF outcomes uses CV death and hospitalization for HF as a composite endpoint. Limitations of this approach include handling of each endpoint with equal importance irrespective of clinical significance, and ignorance of symptomatic and functional endpoints that are also relevant to patients from a clinical standpoint. To overcome this problem, the Win ratio approach was selected using 4 clinically meaningful endpoints as a hierarchical composite (Pocock et al. 2012; Ferreira 2020). This approach has several advantages over the conventional outcome assessment: 1) it prioritizes the more significant component of the

composite (for example, death); 2) more of major component events are included in the analysis; and 3) it allows holistic evaluation of drug effects in a complex disorder such as HF.

Obesity is associated with cardiometabolic derangement and depleted myocardial energetics. During exercise in those with obesity, the heart does not increase antitachycardia pacing delivery, leading to cardiac function impairment and exercise intolerance. It has been shown that weight loss reverses the energetic changes (Rayner et al. 2020). In patients with HFpEF, due to increased filling pressures, functional capacity is severely impaired and patients can develop symptoms with light exercise. As a result, the ability to perform activities of daily living is deteriorated. Obesity is one of the main attributes to worsen quality of life in patients with HFpEF (Reddy et al. 2020). Therefore, 6MWD and KCCQ are meaningful endpoints to evaluate the clinical benefit of tirzepatide in patients with HFpEF and obesity.

For the primary composite endpoint, participants will be classified based on 6MWD categories of change from baseline with 10% incremental for improvement or worsening and KCCQ-CSS categories of change from baseline with 5- or 10-point improvements or worsening. It was estimated that the minimal important difference in 6MWD is approximately 30 to 37 meters in individuals with chronic HF. Relative to the mean 6MWD in these study populations (approximately 350 meters and 480 meters), the 30 to 37 meters is approximately 8% to 9% (Shoemaker et al. 2013; Täger et al. 2014).

For KCCQ-CSS, the categories were specified based on the evidence in the literature suggesting that 5- and 10-point changes in KCCQ summary scores represent small and moderate-to-large clinical changes, respectively. Each 5-point improvement in KCCQ scores has been demonstrated to be associated with improvements in CV mortality and all-cause hospitalization rates, while each 5-point decrement in KCCQ scores was associated with increased risk of CV death and the combined endpoint of CV death and hospitalization, in a linear fashion (Spertus et al. 2020).

4.2.1. Patient Input into Design

The sponsor involved patients in the design of this study by engaging patients in virtual collaborative events. The insights gained from these events were used to ensure that the study design is supportive of the well-being of the study participants and that the study procedures can be implemented effectively at the investigative sites.

4.3. Justification for Dose

Tirzepatide doses of up to 15 mg administered SC QW will be evaluated in this study.

Participants may be treated with lower maintenance doses of 5 mg or 10 mg if they do not achieve full dose escalation to 15 mg and/or do not tolerate 15 mg.

These doses and associated escalation schemes were selected based on assessment of safety, efficacy (glycemic and weight loss benefit), and GI tolerability data followed by exposure response modeling of data in participants with T2DM in Phase 1 and Phase 2 studies. Dosing algorithms starting at a low dose of 2.5 mg accompanied by dose escalation of 2.5 mg increments

every 4 weeks should permit time for development of tolerance to GI events and are predicted to minimize GI tolerability concerns.

The dose selection of tirzepatide is based on the findings of the Phase 2 study results. Tirzepatide doses of 5 mg, 10 mg, and 15 mg QW have been tested and compared with dulaglutide 1.5 mg QW or placebo in a Phase 2 study (Frias et al. 2018). While all 3 doses of tirzepatide significantly improved the glycemic control versus dulaglutide, the largest difference was observed in the 15-mg tirzepatide treatment group. Moreover, the reduction in body weight on tirzepatide was also dose-dependent and greatest in the 15 mg QW treatment group.

4.4. End of Study Definition

The end of the study is defined as the date of the last visit or last scheduled procedure shown in the SoA for the last participant in the trial globally.

The criteria used to determine if a participant has completed the study will be described in the SAP.

5. Study Population

Prospective approval of protocol deviations to recruitment and enrollment criteria, also known as protocol waivers or exemptions, is not permitted.

5.1. Inclusion Criteria

Participants are eligible to be included in the study only if all of the following criteria apply:

Age

1. Participant must be at least 40 years of age inclusive, at the time of signing the ICF.

Type of Participant and Disease Characteristics

2. 6MWD ≤465 meters at both Visit 1 tests, between ≥100 meters and ≤425 meters at Visit 2, and change from the preceding qualifying 6MWD is <20% and <40 meters. See Section 10.10.1 for the flow diagram of the below qualifiers.
   - *If* Visit 2a 6MWD is both between 100 and 425 meters and…
     - <20% AND <40 meter change from the higher of the two 6MWDs conducted at Visit 1, *then* participant meets this inclusion criterion.
     - ≥20% OR ≥40 meter change from the higher of the two 6MWDs at Visit 1, *then* participant must attend Visit 2b. If at Visit 2b, the 6MWD is between 100 and 425 meters and <20% AND <40 meter change from preceding (Visit 2a) 6MWD, then participant meets this inclusion criterion.
3. Chronic HF (NYHA Class II-IV) diagnosed for at least 3 months before Visit 1
4. LVEF ≥50% demonstrated by echocardiogram performed at Visit 1 or within 6 months of Visit 1
5. At least 1 of the following to document evidence of HF:
   - Elevated NT-proBNP >200 pg/mL for participants without atrial AF or >600 pg/mL for participants with AF, as analyzed at the central laboratory at Visit 1
     OR
   - Evidence of structural heart disease:
     - LA enlargement (any of the following: LAV index ≥29 mL/m^2, or LAV >58 mL in male participants and >52 mL in female participants, or LA area >20 cm^2, or LA diameter >40 mm in male and >38 mm in female participants) determined by echocardiogram at Visit 1 or within 6 months of Visit 1

     OR
   - Evidence of elevated filling pressure:
     - At rest (PCWP ≥15 mmHg or LVEDP ≥15 mmHg) or with exercise (PCWP ≥25 mmHg) (based on historical record, not associated with hospitalization for decompensation of HF, within 2 years of Visit 1), or
     - E/e' ratio >15 (septal) or >13 (average of septal and lateral) determined by echocardiogram at Visit 1 or within 6 months of Visit 1

Note: Supporting medical documentation is required in all instances

6. Either one of:
   - eGFR <70 mL/min/1.73m^2 at Visit 1, OR
   - HF decompensation within 12 months of Visit 1, defined as hospitalization for HF requiring IV diuretic treatment or urgent HF visit requiring IV diuretic treatment
     Note: Supporting medical documentation is required in all instances
7. Stable dose of all concomitant HF medications (that is, beta blockers, ACEis, ARBs, and MRAs), except for oral diuretics, for at least 4 weeks prior to Visit 1 and throughout the screening period
8. If treated with oral diuretics, dose must be stable for at least 2 weeks prior to Visit 1 and throughout the screening period; volume control must be optimally achieved in the opinion of the investigator
9. KCCQ-CSS ≤80 at Visit 1

Weight

10. BMI ≥30.0 kg/m^2 at Visit 1

Sex

11. At the time of signing the ICF:
    a. **Male participants**: Male participants with partners of childbearing potential should be willing to use reliable contraceptive methods for the duration of the trial and for 4 months thereafter (see Appendix 4 [Section 10.4]).
    b. Female participants:
    - Female participants not of childbearing potential may participate and include those who are infertile due to surgical sterilization and/or postmenopausal. Please refer to Appendix 4 (Section 10.4) for definitions.
    - Female participants of childbearing potential (not surgically sterilized and between menarche and 1-year postmenopausal) must:
      - test negative for pregnancy at Visit 1 based on a serum pregnancy test followed by a negative urine pregnancy test within 24 hours prior to exposure and agree to use 2 forms of effective contraception, if sexually active, where at least 1 form is highly effective, for the duration of the trial and for 2 months after the last injection, and
      - not be breastfeeding.

Contraceptive use by men or women of childbearing potential should be consistent with local regulations regarding the methods of contraception for those participating in clinical trials. See Appendix 4 (Section 10.4) for guidance.

Informed Consent

12. Capable of giving signed informed consent as described in Appendix 1 (Section 10.1), which includes compliance with the requirements and restrictions listed in the ICF and in this protocol.

5.2. Exclusion Criteria

Participants are excluded from the study if any of the following criteria apply:

Medical Conditions

13. Myocardial infarction, coronary artery bypass graft surgery, or other major CV surgery/intervention, stroke or transient ischemic attack in past 90 days, or unstable angina pectoris in past 30 days, prior to Visit 1 or during screening
14. Dominant contribution of noncardiac causes to exercise impairment or symptoms
    - Lung disease: pulmonary arterial hypertension, chronic thromboembolic pulmonary hypertension (CTEPH), or severe pulmonary disease including (COPD)
    - Other medical conditions: severe anemia (hemoglobin level <9 g/dL) at Visit 1, untreated thyroid disease or TSH >4.0 mU/L at Visit 1, or significant musculoskeletal disease
    - Orthopedic conditions that limit the ability to walk, such as severe arthritis in the leg, knee, hip injuries, hemiplegia, or amputation with artificial limb without stable prosthesis function for the past 3 months
    - Any condition that in the opinion of the investigator would interfere with the assessment of 6MWT
15. LVEF <40% by local echocardiography documented any time within 2 years of Visit 1
16. Acute decompensated HF (exacerbation of HF) requiring IV diuretics, IV inotropes, or IV vasodilators, or left ventricular assist device (LVAD) within 4 weeks prior to Visit 1, and/or during the screening period until randomization
17. Impaired renal function, defined as eGFR <15 mL/min/1.73 m^2 (CKD-EPI) or requiring dialysis at Visit 1
18. Any one of the following:
    - Systolic blood pressure (SBP) ≥180 mmHg at Visit 1
    - SBP >160 mmHg both at Visit 1 and at Visit 2
    - Have symptomatic hypotension or SBP <100 mmHg at Visit 1 or Visit 2
19. Resting heart rate (sinus rhythm) ≥100 bpm at either Visit 1 or Visit 2
20. Atrial fibrillation or atrial flutter with a resting heart rate >110 bpm documented by ECG at Visit 1
21. Cardiac amyloidosis or cardiomyopathy based on accumulation disease (for example, haemochromatosis, Fabry disease), muscular dystrophy, cardiomyopathy with reversible causes (for example, stress cardiomyopathy), hypertrophic obstructive cardiomyopathy or known pericardial constriction, or any severe (obstructive or regurgitant) valvular heart disease likely to lead to surgery during the study period
22. Completed prior surgical treatment for obesity or had liposuction or abdominoplasty within 1 year prior to Visit 1. Participants who plan to have surgical treatment for obesity or liposuction or abdominoplasty during the duration of the study are excluded.
23. Participation in a structured exercise training program in the 1 month prior to Visit 1 or planning to start a program during the study
24. Have T1DM

25. For participants with T2DM:
- Have uncontrolled diabetes requiring immediate therapy (such as diabetic ketoacidosis) at Visit 1 or Visit 2, in the judgement of the physician
- Have had 1 or more events of severe hypoglycemia and/or 1 or more events of hypoglycemia unawareness within 6 months prior to Visit 1 (see Section 10.5.1.1 for definition of hypoglycemia)
- Have HbA1c ≥9.5% (80 mmol/mol) at Visit 1, as analyzed at the central laboratory
- Have a history of proliferative diabetic retinopathy, diabetic maculopathy, or severe nonproliferative diabetic retinopathy that requires acute treatment. Patients with T2DM should have had a dilated fundoscopic examination, performed by an ophthalmologist or optometrist, within 12 months of Visit 1 or prior to randomization
- Treated with premix or prandial insulins or intensified insulin regimens (multiple daily injection with basal and prandial insulins or insulin pump therapy) at Visit 1

26. History of acute or chronic pancreatitis or at high risk for acute pancreatitis (for example, serum triglyceride level >500 mg/dL [5.65 mmol/L])
27. Have acute or chronic hepatitis, signs and symptoms of any other liver disease other than nonalcoholic fatty liver disease, or any of the following, as determined by the central laboratory during Visit 1:

- ALT or AST levels >2.5X the ULN for the reference range.

Note: Participants with nonalcoholic fatty liver disease are eligible to participate in this trial if their ALT level is ≤3.0X the ULN for the reference range.

28. Have a calcitonin level at Visit 1 of:
    - ≥20 ng/L, if eGFR is ≥60 mL/min/1.73 m^2
    - ≥35 ng/L, if eGFR is <60 mL/min/1.73 m^2
29. Have a family or personal history of medullary thyroid carcinoma (MTC) or Multiple Endocrine Neoplasia (MEN) Syndrome type 2
30. Have a history of an active or untreated malignancy or are in remission from a clinically significant malignancy (other than basal- or squamous-cell skin cancer, in situ carcinoma of the cervix, or in situ prostate cancer) for less than 5 years
31. Have a history of any other condition (such as known drug or alcohol abuse, diagnosed eating disorder, or other psychiatric disorder) that, in the opinion of the investigator, may preclude the participant from following and completing the protocol
32. Have a known clinically significant gastric emptying abnormality (for example, severe diabetic gastroparesis or gastric outlet obstruction) or chronically take drugs that directly affect GI motility

Prior/Concomitant Therapy

33. Treatment with any incretin, GLP-1 RA, or pramlintide in the 3 months prior to Visit 1
34. Discontinuation of any incretin, GLP-1 RA, or pramlintide due to intolerability any time prior to Visit 1
35. Have any other condition not listed in this section (for example, hypersensitivity or intolerance) that is a contraindication to GLP-1 RA

36. Implantable cardioverter defibrillator (ICD) implantation within 1 month prior to Visit 1 or planned implantation during the course of the study
37. Currently implanted left ventricular assist device (LVAD)
38. Cardiac resynchronization therapy (CRT) implanted within 6 months prior to Visit 1 or planned implantation during the course of the trial
39. Current use of medication associated with weight gain or weight loss, except when on stable dose for at least 3 months prior to Visit 1, and expected to be stable during the study period

Prior/Concurrent Clinical Study Experience

40. Have participated within the last 6 months in a clinical study involving an investigational product

Other Exclusions

41. Investigator site personnel directly affiliated with this study and/or their immediate families. Immediate family is defined as a spouse, parent, child, or sibling, whether biological or legally adopted
42. Lilly employees

5.3. Lifestyle Considerations

Study participants should be instructed not to donate blood or blood products during the study and for 8 weeks following the study.

5.4. Screen Failures

Screen failures are defined as participants who consent to participate in the clinical study but are not subsequently randomly assigned to study drug. A minimal set of screen failure information is required to ensure transparent reporting of screen failure participants to meet the CONSORT publishing requirements and to respond to queries from regulatory authorities. Minimal information includes demography, screen failure details, eligibility criteria, and any SAE.

Individuals who do not meet certain criteria for participation in this study (screen failure) may be rescreened once. Rescreened participants should be assigned a new participant number. The interval between rescreenings should be at least 2 weeks. For participants who may have screen failed due to HbA1c criterion not met, the time to permit rescreening is at least 8 weeks. If rescreening is performed, the individual must sign a new ICF. If, in the opinion of the investigator, an ineligible laboratory test result is the result of an error or extenuating circumstance, then that parameter can be retested once without the participant having to be rescreened. For rescreened patients, a repeat echocardiogram is not permitted.

Participants may be rescreened for the following reasons:

- Have become eligible to enroll in the study as the result of a protocol amendment
- Status has changed such that the eligibility criterion that caused the participant to screen fail would not cause the participant to screen fail again

- Completed screening and met all inclusion and exclusion requirements but are unable to be enrolled due to extenuating circumstances (such as severe weather, death in family, or child illness)

6. Study Intervention

Study drug is defined as any investigational intervention(s), marketed product(s), placebo, or medical device(s) intended to be administered to/used by a study participant according to the study protocol. For this study, 'study intervention' and 'study drug' are equivalent.

6.1. Study Interventions Administered

**Intervention Name**	Placebo	Tirzepatide (LY3298176)
**Type**	Drug (placebo)	Drug
**Dose Formulation**	Single-dose pen	Single-dose pen
**Unit Dose Strengths**	Not applicable	2.5 mg, 5 mg, 7.5 mg, 10 mg, 12.5 mg, 15 mg
**Dosage Levels**	Not applicable	15 mg QW<br>(or maximum tolerated dose of 5 mg QW or 10 mg QW)
**Route of Administration**	Subcutaneous	Subcutaneous
**Use**	Placebo	Experimental
**IMP and NIMP**	IMP	IMP
**Sourcing**	Provided centrally by the sponsor and dispensed via IWRS	
**Packaging and Labeling**	Study drug will be provided in autoinjectors (single-dose pens), packaged in cartons to be dispensed.<br>Clinical study materials will be labeled according to country regulatory requirements.	

Abbreviations: QW = weekly; IMP = investigational medicinal product; IWRS = interactive web-response system; NIMP = non-investigational medicinal product.

The following table shows the randomized study drugs for the entire study.

Treatment Group	Treatment Period Interval					
	Weeks 0 to 3	Weeks 4 to 7	Weeks 8 to 11	Weeks 12 to 15	Weeks 16 to 19	Weeks 20 to End of Treatment Period
Tirzepatide	2.5 mg	5 mg	7.5 mg	10 mg	12.5 mg	15 mg or MTD
Placebo	→					

Abbreviation: MTD = maximum tolerated dose.

There are no restrictions on the time of day each weekly dose of study drug is given, but it is advisable to administer the SC injections on the same day and same time each week. The actual date, time, and injection site location of all dose administrations will be recorded in the diary by the participant. If a dose of study drug is missed, the participant should take it as soon as possible, unless it is within 72 hours of the next dose, in which case that dose should be skipped, and the next dose should be taken at the appropriate time. The day of weekly administration can be changed if necessary, as long as the last dose was administered 72 or more hours previously.

All participants will inject study drug subcutaneously in the abdomen or thigh using the injection supplies provided; a caregiver may also administer the injection in the participant's upper arm. The injection site location of all dose administrations will be recorded by the participant. A new autoinjector will be used for each injection. If study drug is to always be injected in the same body region, participants should be advised to rotate injection sites each week.

6.1.1. Medical Devices

The combination product provided for use in the study is a tirzepatide or matching placebo autoinjector.

6.2. Preparation/Handling/Storage/Accountability

- The investigator or designee must confirm appropriate storage conditions have been maintained during transit for all study drug received and any discrepancies are reported and resolved before use of the study drug.
- Only participants enrolled in the study may receive study drug. Only study personnel may supply study drug.
- All study drug must be stored in a secure, environmentally controlled, and monitored (manual or automated) area in accordance with the labeled storage conditions with access limited to the investigator and authorized study personnel.
- The investigator, institution, or the head of the medical institution (where applicable) is responsible for study drug (includes study drug and autoinjector or single-dose pen) accountability, reconciliation, and record maintenance (that is, receipt, reconciliation, and final disposition of records).
- Study site staff must regularly assess whether the participant is correctly administering the assigned study drug and storing study drug according to the provided instructions.

Further guidance and information for the final disposition of unused study drugs are provided in the study training materials.

The investigator or designee is responsible for the following:

- Explaining the correct use of the study drug to the participant
- Verifying that instructions are followed properly
- Maintaining accurate records of study drug dispensing and collection as well as records of interruptions in study drug administration
- Instructing the participant to discard all used autoinjectors for study drug in a closeable, puncture-resistant container and dispose according to local regulations, and
- Considering dose adjustment of antihyperglycemic medications (see Section 6.5) at Visit 2 from first administration of study drug.

6.3. Measures to Minimize Bias: Randomization and Blinding

This is a double-blind study.

Participants who meet all criteria for enrollment will be randomized to one of the study treatment groups at the end of Visit 2. Assignment to treatment groups will be determined by a computer-generated, random sequence using an IWRS. Participants will be randomized in a 1:1 ratio to receive tirzepatide or placebo. The randomization will be stratified by HF decompensation (hospitalization for HF requiring IV diuretic treatment or urgent HF visit requiring IV diuretic treatment) within 12 months of screening (Y/N), diagnosed T2DM (Y/N), and BMI ≥35 kg/m^2 (Y/N).

Before the study is initiated, the log in information and directions for the IWRS will be provided to each site.

Study drug will be dispensed at the study visits shown in the SoA.

Returned study drug should not be re-dispensed to the participants.

The IWRS will be programmed with blind-breaking instructions. In case of an emergency, the investigator has the sole responsibility for determining if unblinding of a participant's drug assignment is warranted. Participant safety must always be the first consideration in making such a determination. If a participant's drug assignment is unblinded, the sponsor must be notified immediately after breaking the blind. The date and reason that the blind was broken must be recorded in the source documentation and CRF, as applicable.

If an investigator, site personnel performing assessments, or participant is unblinded, the participant must be discontinued from the study. In cases where there are ethical reasons to have the participant remain in the study, the investigator must obtain specific approval from a sponsor CRP for the participant to continue in the study.

6.4. Study Intervention Compliance

Study drug compliance will be determined by the following:

- Study drug administration data will be recorded by the participants in the participant study diary and reviewed by the investigator at each study visit.

- The participants will be instructed to return any unused study drug and/or empty cartons at the next visit to the study site for the purpose of performing drug accountability.

Treatment compliance for each visit interval is defined as taking at least 75% of the required doses of study drug. Similarly, a participant will be considered significantly noncompliant if he or she is judged by the investigator to have intentionally or repeatedly taken more than the prescribed amount of medication (more than 125%).

In addition to the assessment of a participant's compliance with the study drug administration, other aspects of compliance with the study drug will be assessed at each visit based on the participant's adherence to the visit schedule, completion of study diaries, and any other parameters the investigator considers necessary.

Participants considered to be poorly compliant with their medication and/or the study procedures will receive additional training and instruction, as required, and will be reminded of the importance of complying with the protocol.

6.5. Concomitant Therapy

Any medication or vaccine (including over-the-counter or prescription medicines, vitamins, and/or herbal supplements) or other specific categories of interest that the participant is receiving at the time of enrollment or receives during the study must be recorded along with:

- Reason for use
- Dates of administration including start and end dates
- Dosage information including dose and frequency for concomitant therapy of special interest

The sponsor should be contacted if there are any questions regarding concomitant or prior therapy.

Initial doses of tirzepatide delay gastric emptying and have the potential to transiently impact the rate of absorption of concomitantly administered oral medicinal products. Tirzepatide should be used with caution in participants receiving oral medicinal products that require rapid GI absorption following the initial doses of tirzepatide, as exposure to oral medications may be increased.

Prevention of Hypoglycemia

Similar to GLP-1 RAs, tirzepatide does not generally cause hypoglycemia, but it is recommended to decrease the dose of concomitant sulfonylurea or insulin to reduce the risk of hypoglycemic episodes in patients with T2DM. For participants with T2DM, specific, individually tailored adjustments of the respective antihyperglycemic medications should be considered during the entire study. At Visit 2, with the initiation of study drug, the dose adjustments to the following concomitant glucose lowering medications are recommended.

Sulfonylureas: Sulfonylurea dose is recommended to be reduced at least 50% or discontinued, especially if the participant is receiving a low dose at randomization.

Insulins: For participants on basal insulin and with screening HbA1c ≤8.5%, the daily dose is recommended to be reduced by at least 20%.

During the dose escalation period, consider adjusting the total daily dose of insulin, if required for controlling worsening hyperglycemia and its acute complications.

During the maintenance period, further insulin dose reduction for the prevention of hypoglycemia is to be considered at the investigator’s discretion.

Standard of Care for T2DM

The standard of care for diabetes may be adjusted at the discretion of the investigator as clinically indicated in accordance with local standard of care and professional society guidelines.

Participants will be permitted to use concomitant medications that they require during the study, except certain medications that may interfere with the assessment of efficacy and safety characteristics of the study drug. Prohibited medications include all GLP-1 RAs and pramlintide. Discontinuation of dipeptidyl peptidase 4 inhibitors at randomization is recommended in line with guidelines. Similarly, the use of dipeptidyl peptidase 4 inhibitor therapies during the study is also discouraged (Davies et al. 2018).

Hyperglycemia Rescue

Other medications for glycemic control for participants with T2DM meeting severe, persistent hyperglycemia criteria for rescue may be added during the study at the investigator’s discretion.

Rescue therapy with glucose-lowering agents, including basal and prandial insulins, may be medically indicated in situations after randomization due to severe, persistent hyperglycemia or early discontinuation of study drug.

Hyperglycemia rescue criteria will be determined from values recorded in T2DM participant diaries. If a diary value equal to or greater than the glycemic threshold for rescue (see definitions below) is recorded, that value should be confirmed by a repeat fasting glucose text within 48 hours (for example, local laboratory). Intensification of T2DM therapy should be initiated if confirmed fasting glucose values are:

- ≥15.0 mmol/L (270 mg/dL) from baseline to Week 6 over at least a 2-week period (at least 2 consecutive values) after randomization
- ≥13.3 mmol/L (240 mg/dL) from Week 6 to Week 12 over at least a 2-week period (at least 2 consecutive values)
- ≥11.1 mmol/L (200 mg/dL) from Week 12 to end of trial over at least a 2-week period (at least 2 consecutive values)

In addition, if HbA1c is >9.0% at Week 12 or >8.0% at Week 26 or later in the study, glucose-lowering therapy should be adjusted to improve glycemic control as outlined above. In the event a participant’s HbA1c values are less than these thresholds but are higher than what the investigator feels comfortable leaving untreated, glucose-lowering medication can be adjusted. In addition, if participants develop symptoms of hyperglycemia (for example, polyuria and polydipsia), the investigator should implement measures to determine glycemic control and adjust as necessary. For participants newly diagnosed with T2DM during the trial, appropriate glucose-lowering therapy should be initiated per standard of care.

Standard of Care for Heart Failure

Both American College of Cardiology/American Heart Association and European Society of Cardiology guidelines recommend symptom management with diuretic agents in patients with excess volume, as well as aggressive risk factor management for comorbidities for the treatment of HFpEF (van der Meer JACC 2019). Optimization of volume status and proactive adjustment of diuretic doses will help control symptoms and volume overload.

Participants should remain on stable doses of medications to treat comorbidities such as hypertension. Dose reduction or discontinuation of such background therapies should be avoided unless all other measures fail to improve the participant's condition. However, if the participant's condition warrants a change in any of these medications, it will be allowed at the discretion of the investigator.

Management of Participants with Gastrointestinal Symptoms

In the Phase 2 program, the most commonly reported TEAEs for participants receiving tirzepatide were nausea, vomiting, and diarrhea. To mitigate GI symptoms and manage participants with intolerable GI AEs, the investigator should:

- Advise participants to eat smaller meals, for example, splitting 3 daily meals into 4, or more smaller meals, and to stop eating when they feel full. Also, participants may be informed that lower-fat meals could be better tolerated.
- Prescribe symptomatic medication (for example, anti-emetic or antidiarrheal medication) per local country availability and individual participant needs. Use of symptomatic medication should be captured as concomitant medication in the eCRF.
- Temporarily interrupt study drug (omit 1 dose; the participant will take 3 of 4 doses at that dose level). The data related to temporary interruption of study drug should be documented in source documents and entered on the eCRF.
- After the interruption, restart at the same dose with the participant taking medication to alleviate their GI symptoms.

If intolerable GI symptoms or events persist despite the above measures, see Section 6.6.

6.6. Dose Modification

Participants who do not tolerate the first dose escalation (that is, from 2.5 mg to 5 mg [or placebo equivalent]) will need to discontinue from study drug. Interventions to optimize study drug tolerance and adherence may be employed throughout the study and include, but are not limited to, brief temporary interruptions and use of additional medications to manage GI symptoms.

- If a participant does not tolerate a dose level higher than 5 mg for 2 weeks (for example, moderate-to-severe nausea, vomiting, or diarrhea) and the investigator does not believe that the participant will tolerate the dose with further exposure, then the investigator may reduce the dose to the next lower 5-mg incremental dose (for example, 5 mg or 10 mg).
    - If this dose is tolerated after 4 weeks, the dose should be increased by 2.5 mg every 4 weeks until the target dose is achieved.

  - If this dose escalation is not tolerated, the dose should be reduced to the next lower 5-mg incremental dose that was tolerated (for example, 5 mg or 10 mg). The participant will remain at that dose level for the duration of the study. Maintenance doses of 2.5 mg, 7.5 mg, or 12.5 mg will not be allowed.

After the dose escalation period and at the investigator's discretion, when excessive weight reduction is not warranted due to safety concerns, study drug dose can be adjusted as described above. Participants on 5 mg will have study drug temporarily interrupted (Section 7.1.2). Participants on 10 mg or 15 mg of tirzepatide will be decreased to 5 mg. If continued weight loss concerns persist, study drug could be temporarily interrupted.

In order to maintain blinding of the investigator and participant, the investigator should call the IWRS to explain that the participant needs the dose reduced, and the IWRS will provide dispensing information. Dose reductions may occur at scheduled and unscheduled visits.

6.7. Intervention after the End of the Study

Tirzepatide will not be made available to participants after conclusion of the study.

7. Discontinuation of Study Intervention and Participant Discontinuation/Withdrawal

7.1. Discontinuation of Study Intervention

7.1.1. Permanent Discontinuation from Study Drug

In rare instances, it may be necessary for a participant to permanently discontinue study drug. If study drug is definitively discontinued, the participant will remain in the study and attend all scheduled visits to be evaluated for safety and efficacy and any further evaluations as described in the SoA.

Possible reasons leading to permanent discontinuation of study drug:

- **Participant decision**
  - The participant or the participant's designee (for example, legal guardian) requests to discontinue study drug
- **Investigator Decision**
  - The investigator decides that the participant should be discontinued from study drug
- **Discontinuation due to a hepatic event or liver test abnormality**
  Participants who are interrupted/discontinued from study drug due to a hepatic event or liver test abnormality should have additional hepatic safety data collected via CRF.

  Interruption/discontinuation of the study drug for abnormal liver test results should be considered by the investigator when a participant meets one of the following conditions after consultation with the sponsor-designated medical monitor. These criteria differ based on the baseline liver test findings and are summarized below (Regev et al. 2019). If

study drug is interrupted, it can be restarted only if another etiology is identified and liver enzymes return to baseline.

For participants with baseline ALT <1.5X ULN and normal baseline bilirubin:

- ALT or AST >8X ULN
- ALT or AST >5X ULN for more than 2 weeks
- ALT or AST >3X ULN and TBL >2X ULN or INR >1.5
- ALT or AST >5X ULN with the appearance of fatigue, nausea, vomiting, right upper-quadrant pain or tenderness, fever, rash, and/or eosinophilia (>5%)
- ALP >3X ULN
- ALP >2.5X ULN and TBL >2X ULN
- ALP >2.5X ULN with the appearance of fatigue, nausea, vomiting, right quadrant pain or tenderness, fever, rash, and/or eosinophilia (>5%)

For participants with baseline ALT ≥1.5X ULN and normal baseline bilirubin:

- ALT >5X baseline or ≥500 U/L (whichever occurs first)
- ALT >2X baseline or ≥300 U/L (whichever occurs first) and TBL >2X ULN
- ALT >3X baseline or ≥300 U/L (whichever occurs first) with the appearance of fatigue, nausea, vomiting, right upper-quadrant pain or tenderness, fever, rash, and/or eosinophilia (>5%)
- ALP >3X ULN
- ALP >2.5X ULN and TBL >2X ULN
- ALP >2.5X ULN with the appearance of fatigue, nausea, vomiting, right quadrant pain or tenderness, fever, rash, and/or eosinophilia (>5%)

- In addition, participants will be permanently discontinued from the study drug in the following circumstances:
  - acute or chronic pancreatitis (see Section 10.5.1.2)
  - if a participant is diagnosed with thyroid C-cell hyperplasia, MEN-2, or MTC after randomization
  - if a participant is diagnosed with an active or untreated malignancy (other than basal or squamous cell skin cancer, in situ carcinoma of the cervix, or in situ prostate cancer) after randomization
  - if any other TEAE, SAE, or clinically significant laboratory value for which the investigator believes that permanent study drug discontinuation is the appropriate measure to be taken
  - if a participant is diagnosed with T1DM
  - if a female participant becomes pregnant
  - if an investigator, site personnel performing assessments, or participant is unblinded, or
  - if the investigator, after consultation with the sponsor-designated medical resource, determines that a clinically significant systemic hypersensitivity reaction has occurred. A clinically significant systemic hypersensitivity reaction is one that occurs

after administration of the study drug (for example, drug-related symptomatic bronchospasm, allergy-related edema/angioedema, or hypotension) and requires parenteral medication, does not respond to symptomatic medication, results in clinical sequelae, or is an anaphylactic reaction.

7.1.2. Temporary Interruption

After randomization, the investigator may interrupt study drug, for example, due to an AE (such as nausea or vomiting) or a clinically significant laboratory value. If study drug interruption is due to an AE, the event is to be followed and documented.

Every effort should be made by the investigator to maintain participants in the study and to restart study drug promptly after any interruption, as soon as it is safe to do so (see Section 7.1.3 for restarting study drug). The dates of study drug interruption and restart must be documented. The data related to interruption of study drug will be documented in source documents and entered on the eCRF. Participant noncompliance should not be recorded as interruption of study drug on the eCRF.

Participants who have a temporary interruption of the study drug will continue participating in the trial according to the protocol to collect all planned efficacy and safety measurements (Section 1.3).

Participants temporarily interrupting study drug for any reason should complete AE and other follow-up procedures per Section 1.3 (SoA), Section 8.3 (Adverse Events), and Section 8.2 (Safety) of this protocol.

7.1.3. Restarting Study Drug after Interruption

In certain situations, after randomization, the investigator may need to temporarily interrupt study drug. Every effort should be made by the investigator to maintain participants on study drug and to restart study drug after any temporary interruption, as soon as it is safe to do so. Distribution of study drug at the correct dose will be per IWRS instructions.

If study drug interruption is…	then…
1 or 2 consecutive doses	Participant restarts study drug at last administered dose, per escalation schedule.
3 or more consecutive doses	Participant restarts study drug (at 5 mg, managed by IWRS) and repeats dose escalation scheme.
Due to an AE	The event is to be documented and followed according to the procedures in Section 8.3.
Due to intolerable persistent GI AE	Participants should be treated as suggested in Section 6.5.

Abbreviations: AE = adverse event; GI = gastrointestinal; IWRS = interactive web response service.

Investigators should inform the sponsor that study drug has been temporarily interrupted.

In the event that a participant has a temporary interruption that requires extending the escalation beyond Visit 8, unscheduled visits are allowed in the IWRS to facilitate a 4-week dispensing schedule to complete the escalation.

7.2. Participant Discontinuation/Withdrawal from the Study

In order to minimize the amount of missing data and to enable assessment of study objectives as planned in the study protocol, every attempt will be made to keep participants in the study, irrespective of the following:

- compliance to study drug
- adherence to visit schedule
- missing assessments
- study drug discontinuation due to AE (Section 7)
- development of comorbidities, and
- development of clinical outcomes.

The circumstances listed above are ***not*** valid reasons for participant discontinuation from the study.

Participant will be discontinued from study in the following circumstances:

- enrollment in any other clinical study involving a study drug or enrollment in any other type of medical research judged not to be scientifically or medically compatible with this study
- participation in the study needs to be stopped for medical, safety, regulatory, or other reasons consistent with applicable laws, regulations, and GCP, and
- if the participant becomes pregnant.

A participant may withdraw from the study:

- at any time at his/her own request, or
- at the request of his/her designee (for example, legal guardian).

Discontinuation from the study is expected to be rare.

At the time of discontinuing from the study, if possible, an ET visit should be conducted, as shown in the SoA. See the SoA for data to be collected at the time of study discontinuation for any further evaluations that need to be completed. The participant will be permanently discontinued from the study at that time.

If the participant withdraws consent for disclosure of future information, the sponsor may retain and continue to use any data collected before such a withdrawal of consent. If a participant withdraws from the study, he/she may request destruction of any samples taken and not tested, and the investigator must document this in the site study records.

7.2.1. Inadvertently Enrolled Participants

If the sponsor or investigator identifies a participant who did not meet enrollment criteria and was inadvertently enrolled, then the participant should remain in the study and be discontinued

from study drug unless there are extenuating circumstances that make it medically necessary for the participant to continue on study drug. If the investigator and the sponsor CRP agree it is medically appropriate to continue the study drug, the investigator must obtain documented approval from the sponsor CRP to allow the inadvertently enrolled participant to continue study drug. Safety follow-up should be performed as outlined in Section 8.2 (Safety Assessments) and Section 8.3 (Adverse Events and Serious Adverse Events) of the protocol.

7.3. Lost to Follow up

A participant will be considered lost to follow-up if he or she repeatedly fails to return for scheduled visits and is unable to be contacted by the study site (≥2 consecutive visits). Site personnel or designee are expected to make diligent attempts to contact participants who fail to return for a scheduled visit or were otherwise unable to be followed up by the site.

Site personnel, or an independent third party, will attempt to collect the vital status of the participant within legal and ethical boundaries for all participants randomized, including those who did not get study drug. Public sources may be searched for vital status information. If vital status is determined to be deceased, this will be documented and the participant will not be considered lost to follow-up. Sponsor personnel will not be involved in any attempts to collect vital status information.

Discontinuation of specific sites or of the study as a whole are handled as part of Appendix 1 (Section 10.1).

8. Study Assessments and Procedures

Study procedures and their timing are summarized in the SoA.

Immediate safety concerns should be discussed with the sponsor immediately upon occurrence or awareness to determine if the participant should continue or discontinue study drug.

Adherence to the study design requirements, including those specified in the SoA, is essential and required for study conduct.

All screening evaluations must be completed and reviewed to confirm that potential participants meet all eligibility criteria. The investigator will maintain a screening log to record details of all participants screened and to confirm eligibility or record reasons for screening failure, as applicable.

Repeat or unscheduled samples may be taken for safety reasons or for technical issues with the samples.

8.1. Efficacy Assessments

8.1.1. Primary Efficacy Assessment

The primary efficacy endpoints are

- the hierarchal composite of time to all-cause mortality, occurrence of HF events through the end of the treatment period, the change from baseline in 6MWD, and the change from baseline in KCCQ-CSS at 52 weeks, and

- change from baseline in 6MWD at 52 weeks.

The primary efficacy assessments include

- time to all-cause mortality
- occurrence of HF events
- 6MWT, and
- KCCQ.

An independent CEC will adjudicate death and HF events. The CEC charter will contain the final detailed event definitions for all adjudicated events.

8.1.1.1. Time to All-Cause Mortality

All-cause mortality will be recorded by the investigator at the time of death.

8.1.1.2. Occurrence of Heart Failure Events

HF events will be recorded when the investigator becomes aware of any

- hospitalization for HF, or
- urgent HF visit.

8.1.1.3. Six-Minute Walk Test

Participants will perform an exercise capacity assessment using the 6MWT. Testing of the 6MWT should be performed as directed in the SoA (Section 1.3). The 6MWT is to be performed indoors on a straight, flat, hard surface that is at least 30 meters in length.

The 6MWTs at Visits 1 and 2 will be performed to assess participant eligibility. The Visit 1 tests will also serve as the training test to familiarize participants with the procedure. Additional details can be found in Section 10.10.1.

Prior to and at the end of each 6MWT, participants will be asked to rate their breathing discomfort and overall fatigue using the Borg Scale.

8.1.1.4. Kansas City Cardiomyopathy Questionnaire

The KCCQ is a 23-item, participant self-administered questionnaire that assesses impacts of HF “over the past 2 weeks” on the following 7 domains (Green et al. 2000; Joseph et al. 2013):

- Physical Limitation (6 items)
- Symptom Stability (1 item)
- Symptom Frequency (4 items)
- Symptom Burden (3 items)
- Self-Efficacy (2 items)
- Quality of Life (3 items), and
- Social Limitation (4 items).

Each of the 23 individual items are answered on Likert scales of varying lengths (5-point, 6-point, or 7-point scales). Domain scores are obtained by averaging the associated individual

items and transforming the score to a 0 to 100 range. Higher scores indicate better health status. Summary scores are obtained by combining select domain scores:

- Total Symptom Score: mean of the Symptom Frequency and Symptom Burden scores
- Clinical Summary Score: mean of the Physical Limitation and Total Symptom scores, and
- Overall Summary Score: mean of the Physical Limitation, Total Symptom, Quality of Life, and Social Limitation scores.

The Clinical Summary Score will be used for the primary and key secondary endpoints.

8.1.2. Additional Secondary Efficacy Assessments

Body weight will be assessed as described in Section 8.2.1.

The NYHA classification will be assessed and recorded at the time points indicated in the SoA (Section 1.3) by an independent, blinded assessor. The NYHA classification is provided in Appendix 11 (Section 10.11).

8.1.3. Exploratory Efficacy Assessments

8.1.3.1. Patient-Reported Outcomes Assessments

The self-reported questionnaires will be translated into the native language of the region, linguistically validated, and administered according to the SoA (Section 1.3). At these visits, the questionnaires should be completed before the participant has discussed their medical condition or progress in the study with the investigator and/or site staff.

8.1.3.1.1. Patient Global Impression of Status – Overall

Study participants will be asked to complete a Patient Global Impression of Status – Overall item specifically developed for this study. This is a participant-rated assessment of their overall health "in the past 2 weeks" and is rated on a 5-point scale ranging from "1-Excellent" to "5-Poor."

8.1.3.1.2. Patient Global Impression of Status – Physical Function

Study participants will be asked to complete a Patient Global Impression of Status – Physical Function item specifically developed for this study. This is a participant-rated assessment of the overall impact of HF symptoms on their ability to perform physical activities "in the past 2 weeks" and is rated on a 5-point scale ranging from "1- Not impacted" to "5- Extremely impacted, cannot perform physical activities."

8.1.3.1.3. Patient Global Impression of Status – Symptom Severity

Study participants will be asked to complete a Patient Global Impression of Status – Symptom Severity item specifically developed for this study. This is a participant-rated assessment of the overall severity of their HF symptoms "in the past 2 weeks" and is rated on a 5-point scale ranging from "1- No symptoms" to "5- Very severe."

8.1.3.1.4. EQ-5D-5L

Generic health-related quality of life will be assessed using the EQ-5D-5L (EuroQoL Research Foundation 2019). The EQ-5D-5L is a standardized 5-item instrument for use as a measure of

health outcome. It provides a simple descriptive profile and a single index value for health status that can be used in the clinical and economic evaluation of health care as well as population health surveys. The EQ-5D-5L comprises 5 dimensions of health (mobility, self-care, usual activities, pain/discomfort, and anxiety/depression). The 5L version, introduced in 2005, scores each dimension at 5 levels (no problems, slight problems, moderate problems, severe problems, and unable to perform/extreme problems), for a total of 3125 possible health states. In addition to the health profile, a single health state index value can be derived based on a formula that attaches weights to each of the levels in each dimension. This index value ranges between less than 0 (where 0 is a health state equivalent to death; negative values are valued as worse than dead) to 1 (perfect health). In addition, the EQ Visual Analog Scale records the respondent's self-rated health status on a vertical graduated (0 to 100) visual analog scale. In conjunction with the health state data, it provides a composite picture of the respondent's health status.

The EQ-5D-5L is used worldwide and is available in more than 170 languages. Details on the instrument, scoring, organizing, and presenting the data collected can be found in the EQ-5D-5L User Guide (EuroQoL Research Foundation 2019).

8.2. Safety Assessments

Planned time points for all safety assessments are provided in the SoA (Section 1.3).

8.2.1. Physical Examinations

- A complete physical examination will include, at a minimum, assessments of the CV, respiratory, GI and neurological systems, as well as a thyroid examination.
  - Body weight, waist circumference, and height should be measured. All weights for a given participant should be measured in a consistent manner using a calibrated scale (mechanical or digital scales are acceptable), using the same scale whenever possible, and after the participant has emptied their bladder. Participants should be lightly clothed but not wearing shoes while their weight is measured.
- Symptom-directed physical examinations will be conducted as described in the SoA.
  - Investigators should pay special attention to clinical signs and symptoms related to HF as well as related to previous serious illnesses. Particular interest would include dyspnea, orthopnea, paroxysmal nocturnal dyspnea, edema, jugular venous distension, and rales.

The physical examination should be performed before the first 6MWT, if more than one 6MWTs are done.

8.2.2. Vital Signs

For each participant, vital signs measurements should be conducted according to the SoA (Section 1.3).

Any clinically significant findings from vital signs measurements that result in a diagnosis and that occur after the participant receives the first dose of study drug should be reported to the sponsor or its designee as an AE via the eCRF.

8.2.3. Electrocardiograms

Single 12-lead ECGs will be obtained locally as outlined in the SoA (see Section 1.3) using an ECG machine that automatically calculates the heart rate and measures PR, QRS, QT, and QTc intervals.

All ECGs should be recorded after the participant has been supine for 5 minutes in a quiet room.

The ECGs must be interpreted by a qualified physician (the investigator or designee) at the site as soon after the time of ECG collection as possible, and ideally while the participant is still present, for immediate participant management, if needed. The investigator (or qualified designee) is responsible for determining if any change in participant management is needed, and must document his/her review of the ECG printed at the time of evaluation. If a clinically relevant abnormality is observed on the participant's ECG, then the investigator should assess the participant for symptoms (such as palpitations, near syncope, syncope, or chest pain). The investigator must report the presence of AF on the eCRF.

The original ECG must be retained at the investigative site.

The investigator or qualified designee's interpretation will prevail for immediate participant management purposes.

8.2.4. Clinical Safety Laboratory Assessments

With the exception of laboratory test results that may unblind the study, the sponsor or its designee will provide the investigator with the results of laboratory tests analyzed by a central vendor, if a central vendor is used for the clinical trial.

See Appendix 2 (Section 10.2) for the list of clinical laboratory tests to be performed. The SoA describes the timing and frequency.

The investigator must review the laboratory results, document this review, and report any clinically relevant changes occurring during the study as an AE. The laboratory results must be retained with source documents unless a Source Document Agreement or comparable document cites an electronic location that accommodates the expected retention duration. Clinically significant abnormal laboratory findings are those that are not associated with the underlying disease, unless judged by the investigator to be more severe than expected for the participant's condition.

All laboratory tests with values considered clinically significantly abnormal during participation in the study should be repeated until the values return to normal or baseline levels or are no longer considered clinically significant by the investigator or medical monitor.

- If such values do not return to normal/baseline levels within a period of time judged reasonable by the investigator, the etiology should be identified and the sponsor notified.
- All protocol-required laboratory assessments, as defined in Appendix 2 (Section 10.2), must be conducted in accordance with the SoA, standard collection requirements, and laboratory manual.

If laboratory values from non-protocol specified laboratory assessments performed at an investigator-designated local laboratory require a change in participant management or are considered clinically significant by the investigator (for example, SAE or AE or dose modification), then report the information as an AE.

8.2.5. Safety Monitoring

The sponsor will periodically review evolving aggregate safety data within the study by appropriate methods. The study team will review safety reports in a blinded fashion according to the schedule provided in the Trial-Level Safety Review Plan. The sponsor will also review SAEs within time frames mandated by company procedures. The Sponsor CRP will, as appropriate, consult with the functionally independent Global Patient Safety therapeutic area physician or clinical scientist.

8.2.5.1. Hepatic Safety Monitoring

Close Hepatic Monitoring

Laboratory tests (Appendix 8 [Section 10.8]), including ALT, AST, ALP, TBL, D Bil, GGT, and creatine kinase, should be repeated within 48 to 72 hours to confirm the abnormality and to determine if it is increasing or decreasing, if one or more of these conditions occur:

If a participant with baseline results of …	develops the following elevations:
ALT or AST <1.5X ULN	ALT or AST ≥3X ULN
ALP <1.5X ULN	ALP ≥2X ULN
TBL <1.5X ULN	TBL ≥2X ULN (except for participants with Gilbert's syndrome)
ALT or AST ≥1.5X ULN	ALT or AST ≥2X baseline
ALP ≥1.5X ULN	ALP ≥2X baseline
TBL ≥1.5x ULN	TBL ≥1.5X baseline (except for participants with Gilbert's syndrome)

Abbreviations: ALP = alkaline phosphatase; ALT = alanine aminotransferase; AST = aspartate aminotransferase; TBL = total bilirubin; ULN = upper limit of normal.

If the abnormality persists or worsens, clinical and laboratory monitoring and evaluation for possible causes of abnormal liver tests should be initiated by the investigator in consultation with the sponsor-designated medical monitor. At a minimum, this evaluation should include physical examination and a thorough medical history, including symptoms, recent illnesses (for example, HF, systemic infection, hypotension, or seizures), recent travel, history of concomitant medications (including over-the-counter), herbal and dietary supplements, history of alcohol use, and other substance abuse.

Initially, monitoring of symptoms and hepatic biochemical tests should be done at a frequency of 1 to 3 times weekly, based on the participant's clinical condition and hepatic biochemical tests. Subsequently, the frequency of monitoring may be lowered to once every 1 to 2 weeks, if the

participant's clinical condition and laboratory results stabilize. Monitoring of ALT, AST, ALP, and TBL should continue until levels normalize or return to approximate baseline levels.

Comprehensive Hepatic Evaluation

A comprehensive evaluation should be performed to search for possible causes of liver injury if one or more of these conditions occur:

If a participant with baseline results of …	develops the following elevations:
ALT or AST <1.5X ULN	ALT or AST ≥3X ULN with hepatic signs/symptoms* OR<br>ALT or AST ≥5X ULN
ALP <1.5X ULN	ALP ≥3X ULN
TBL <1.5X ULN	TBL ≥2X ULN (except for participants with Gilbert's syndrome)
ALT or AST ≥1.5X ULN	ALT or AST ≥2X baseline with hepatic signs/symptoms* OR<br>ALT or AST ≥3X baseline
ALP ≥1.5X ULN	ALP ≥2X baseline
TBL ≥1.5X ULN	TBL ≥2X baseline (except for participants with Gilbert's syndrome)

Abbreviations: ALP = alkaline phosphatase; ALT = alanine aminotransferase; AST = aspartate aminotransferase; TBL = total bilirubin; ULN = upper limit of normal.

* Hepatic signs/symptoms are severe fatigue, nausea, vomiting, right upper quadrant abdominal pain, jaundice, fever, rash, and/or eosinophilia >5%.

At a minimum, this evaluation should include physical examination and a thorough medical history, as outlined above, as well as tests for PT-INR; tests for viral hepatitis A, B, C, or E; tests for autoimmune hepatitis; and an abdominal imaging study (for example, ultrasound or CT scan).

Based on the participant's history and initial results, further testing should be considered in consultation with the sponsor-designated medical monitor, including tests for hepatitis D virus (HDV), cytomegalovirus (CMV), Epstein-Barr virus (EBV), acetaminophen levels, acetaminophen protein adducts, urine toxicology screen, Wilson's disease, blood alcohol levels, urinary ethyl glucuronide, and blood phosphatidylethanol (see Appendix 8 [Section 10.8]). Based on the circumstances and the investigator's assessment of the participant's clinical condition, the investigator should consider referring the participant for a hepatologist or gastroenterologist consultation, magnetic resonance cholangiopancreatography (MRCP), endoscopic retrograde cholangiopancreatography (ERCP), cardiac echocardiogram, or a liver biopsy.

Additional hepatic data collection (hepatic safety CRF) in study participants who have abnormal liver tests during the study

Additional hepatic safety data collection in hepatic safety CRFs should be performed in study participants who meet 1 or more of the following 5 conditions:

1. Elevation of serum ALT to ≥5X ULN on 2 or more consecutive blood tests (if baseline ALT <1.5X ULN)
   - In participants with baseline ALT ≥1.5X ULN, the threshold is ALT ≥3X baseline on 2 or more consecutive tests
2. Elevated TBL to ≥2X ULN (if baseline TBL <1.5X ULN) (except for cases of known Gilbert's syndrome)
   - In participants with baseline TBL ≥1.5X ULN, the threshold should be TBL ≥2X baseline
3. Elevation of serum ALP to ≥2X ULN on 2 or more consecutive blood tests (if baseline ALP <1.5X ULN)
   - In participants with baseline ALP ≥1.5X ULN, the threshold is ALP ≥2X baseline on 2 or more consecutive blood tests
4. Hepatic event considered to be an SAE
5. Discontinuation of study drug due to a hepatic event

Note: the interval between the two consecutive blood tests should be at least 2 days.

8.3. Adverse Events, Serious Adverse Events, and Product Complaints

The definitions of the following events can be found in Appendix 3 (Section 10.3):

- Adverse events (AEs)
- Serious adverse events (SAEs)
- Product complaints (PCs)

These events will be reported by the participant (or, when appropriate, by a caregiver, surrogate, or the participant's legally authorized representative).

The investigator and any qualified designees are responsible for detecting, documenting, and recording events that meet these definitions and remain responsible for following up events that are serious, considered related to the study drug or study procedures, or that caused the participant to discontinue the study drug or study (see Section 7).

Care will be taken not to introduce bias when detecting events. Open-ended and non-leading verbal questioning of the participant is the preferred method to inquire about event occurrences.

After the initial report, the investigator is required to proactively follow each participant at subsequent visits/contacts. All SAEs will be followed until resolution, stabilization, the event is otherwise explained, or the participant is lost to follow-up (as defined in Section 7.3).

For product complaints, the investigator is responsible for ensuring that follow-up includes any supplemental investigations as indicated to elucidate the nature and/or causality. Further information on follow-up procedures is provided in Appendix 3 (Section 10.3).

Suspected Unexpected Serious Adverse Reactions

Suspected unexpected serious adverse reactions (SUSARs) are serious events that are not listed in the IB and that the investigator identifies as related to study drug or procedure. United States 21 CFR 312.32, European Union Clinical Trial Directive 2001/20/EC, and the associated detailed guidance or national regulatory requirements in participating countries require the reporting of SUSARs. The sponsor has procedures that will be followed for the identification, recording, and expedited reporting of SUSARs that are consistent with global regulations and the associated detailed guidance.

8.3.1. Timing and Mechanism for Collecting Events

This table describes the timing, deadlines, and mechanism for collecting events.

**Event**	**Collection Start**	**Collection Stop**	**Timing for Reporting to Sponsor or Designee**	**Mechanism for Reporting**	**Back-up Method of Reporting**
**Adverse Event**					
AE	Signing of the informed consent form (ICF)	Participation in study has ended	As soon as possible upon site awareness	AE eCRF	N/A
**Serious Adverse Event**					
SAE and SAE updates – prior to start of study drug **and** deemed reasonably possibly related with study procedures	Signing of the informed consent form (ICF)	Start of study drug	Within 24 hours of awareness	SAE eCRF	SAE paper form
SAE* and SAE updates – after start of study drug	Start of study drug	Participation in study has ended	Within 24 hours of awareness	SAE eCRF	SAE paper form
SAE* – after participant's study participation has ended **and** the investigator becomes aware	After participant's study participation has ended	N/A	Promptly	SAE paper form	N/A

Event	Collection Start	Collection Stop	Timing for Reporting to Sponsor or Designee	Mechanism for Reporting	Back-up Method of Reporting
**Pregnancy**					
Pregnancy in female participants and female partners of male participants	After the start of study drug	Four months after the last injection for female partners of male participants and 2 months after the last injection for female participants	Within 24 hours of learning of the pregnancy	SAE eCRF	SAE paper form
**Product Complaints**					
PC associated with an SAE or might have led to an SAE	Start of study drug	End of study drug	Within 24 hours of awareness	Product Complaint form	N/A
PC not associated with an SAE	Start of study drug	End of study drug	Within 1 business day of awareness	Product Complaint form	N/A
Updated PC information	—	—	As soon as possible upon site awareness	Originally completed Product Complaint form with all changes signed and dated by the investigator	N/A
PC (if investigator becomes aware)	Participation in study has ended	N/A	Promptly	Product Complaint form	N/A

Abbreviations: eCRF = electronic case report form; N/A = not applicable; PC = product complaint; SAE = serious adverse event.

*Serious adverse events, including death, caused by disease progression as described in Section 8.3.2 should not be reported unless the investigator deems them to be possibly related to study drug.

8.3.2. Primary, Secondary, and Additional Study Endpoint Reporting

The following investigator-reported events are considered potential endpoints and must be reported first as an AE on the AE eCRF (with the appropriate designation for seriousness). They must then be reported as an endpoint on the eCRF with all required source documents provided for adjudication to the CEC. These potential endpoints (even if they meet criteria for a serious event) are not to be reported on the SAE eCRF unless considered as possibly related to study drug, the drug delivery system, or study procedure. Potential endpoints that are serious and considered as possibly related to study drug, the drug delivery system, or study procedure must also be reported as an SAE using the SAE eCRF:

- all-cause mortality (death), and
- hospitalization for HF or an urgent HF visit.

In the case where 1 of the above endpoint events is reported but does not meet a prespecified event definition detailed in the CEC charter, as reviewed by the independent CEC, no further action will be taken by the study site.

8.3.3. Adverse Events of Special Interest

The following are AESI and will be adjudicated by an external adjudication committee. This committee will be blinded to treatment assignment.

- pancreatitis
- major adverse CV events (see Section 10.5.1.5), and
- deaths

The following are additional AESI for this program that will not be adjudicated by an external committee:

- hepatobiliary disorders
- severe hypoglycemia
- thyroid malignancies and C-cell hyperplasia
- supraventricular arrhythmias and cardiac conduction disorders
- allergic/hypersensitivity reactions, including injection site reactions and ADA formation
- severe GI AEs, and
- acute renal events.

Sites should collect additional details and data regarding AESI, as instructed on the applicable eCRFs, and detailed in Section 10.5.

The details on the definition of AESI will be provided in SAP.

8.4. Treatment of Overdose

Considering the mechanism of action of tirzepatide, potential overdose effects can be GI disorders and hypoglycemia. In the event of overdose, appropriate supportive treatment should be initiated according to the participant's clinical signs and symptoms. In the event of overdose, refer to the IB for tirzepatide.

8.5. Pharmacokinetics

Pharmacokinetic samples will be collected from all participants in this study.

Tirzepatide plasma concentrations will be determined from blood samples obtained from participants receiving tirzepatide treatment. Blood samples collected from participants assigned to the placebo arm will not be included in the bioanalysis of drug concentrations.

Blood samples for PK assessment will be collected prior to the dose administration and at the same time as the planned immunogenicity samples (that is, at Week 0 and then at Weeks 4, 12, 24, and 52 per the Study Schedule or additionally at follow-up and ET (reference SoA).

Samples will be analyzed at a laboratory approved by the sponsor and stored at a facility designated by the sponsor.

Concentrations of tirzepatide will be assayed using a validated liquid chromatography mass spectrometry method. Bioanalytical samples collected to measure tirzepatide concentrations will be retained for a maximum of 1 year following last participant visit for the study (Section 10.1.10). During this time, samples remaining after the bioanalyses may be used for exploratory analyses such as metabolism work, protein binding, and/or bioanalytical method cross-validation.

8.6. Pharmacodynamics

Pharmacodynamic parameters will not be evaluated in this study.

8.7. Genetics

A whole blood sample will be collected for pharmacogenetic analysis where local regulations allow.

See Appendix 2, Clinical Laboratory Tests (Section 10.2), and Section 1.3 (SoA) for sample collection information.

See Section 10.6 for genetic research, custody, and sample retention information.

8.8. Biomarkers

Biomarker research is performed to address questions of relevance to drug disposition, target engagement, pharmacodynamics, mechanism of action, variability of participant response (including safety), and clinical outcome. Sample collection is incorporated into clinical studies to enable examination of these questions through measurement of biomolecules including DNA, RNA, proteins, lipids, and other cellular elements.

Serum and plasma for exploratory biomarker research will be collected at the time specified in the SoA (Section 1.3) where local regulations allow.

All samples will be coded with the participant number. These samples and any data generated can be linked back to the participant only by the investigator site personnel.

Samples will be stored and analysis may be performed on biomarkers thought to play a role in disease processes, mechanism of action of tirzepatide, pathways associated with HFpEF, and/or research methods validating diagnostic tools or assay(s) related to HFpEF and associated diseases. Biomarkers may be evaluated to determine their association with observed clinical responses to tirzepatide and the disease state.

Samples will be retained at a facility selected by the sponsor or its designee for the duration detailed in Section 10.1.10, or for a shorter period if local regulations and ERBs impose shorter time limits. This retention period enables use of new technologies, response to regulatory questions, and investigation of variable response that may not be observed until later in the development of tirzepatide or after tirzepatide becomes commercially available.

8.9. Immunogenicity Assessments

At the visits and times specified in the SoA (Section 1.3), venous blood samples will be collected to determine antibody production against tirzepatide. Antibodies may be further characterized for cross-reactive binding to endogenous counterparts (native GIP and GLP-1) and their ability to neutralize the activity of tirzepatide and endogenous counterparts. To interpret the results of immunogenicity, a venous blood sample will be collected at the same time points to determine the plasma concentrations of tirzepatide. All samples for immunogenicity should be taken predose when applicable and possible.

Samples will be retained for a maximum of 15 years after the last participant visit, or for a shorter period if local regulations and ERBs allow, at a facility selected by the sponsor. The duration allows the sponsor to respond to future regulatory requests related to tirzepatide. Any samples remaining after 15 years will be destroyed.

8.10. Medical Resource Utilization and Health Economics

Not applicable.

9. Statistical Considerations

9.1. Statistical Hypotheses

Two primary hypotheses will be tested in this study:

- Tirzepatide MTD is superior to placebo for the hierarchical composite endpoint of all-cause mortality, occurrence of adjudicated HF events (including HF hospitalization or urgent HF visit) through the end of the treatment period, the change from baseline in 6MWD, and the change from baseline in KCCQ-CSS at Week 52.
- Tirzepatide MTD is superior to placebo for the change from baseline in 6MWD at Week 52.

Key secondary hypotheses (all under multiplicity control) are that tirzepatide MTD is superior to placebo with regards to

- percent change from baseline in body weight at Week 52
- change from baseline in KCCQ-CSS at Week 52
- change from baseline in 6MWD at Week 24, and
- proportion of participants with NYHA class change at Week 52.

All primary and key secondary hypotheses will be tested with the overall family-wise type I error rate at a 2-sided alpha level of 0.05 through the multiplicity control approach based on the graphical multiple testing procedure. For the 2 primary hypotheses, the hierarchical composite endpoint will be tested at a 2-sided alpha level of 0.04, and change in 6MWD will be tested at a 2-sided alpha level of 0.01 in parallel for statistical significance. If either is significant, the corresponding assigned alpha will be propagated to test the other primary efficacy endpoint at a 2-side alpha level of 0.05. The detailed graphical testing scheme will be outlined in the SAP.

9.2. Sample Size Determination

Approximately 700 participants will be randomized to either tirzepatide or placebo in a 1:1 ratio (approximately 350 participants per treatment arm), and the statistical powers are evaluated for each of the 2 primary efficacy endpoints at a 2-sided significance level of 0.01 to ensure that the sample size and power are sufficient for registration purposes. This sample size will provide

- approximately 85% power or higher to demonstrate superiority of tirzepatide MTD to placebo for the hierarchical composite endpoint using Finkelstein-Schoenfeld method (Finkelstein and Schoenfeld 1999)
- approximately 95% power for the change from baseline in 6MWD using Wilcoxon rank sum test, and
- more than 95% power to demonstrate superiority for at least one of above.

The sample size and power are estimated through simulations under the following assumptions:

Variable	Placebo	Tirzepatide MTD	Treatment Difference
All-cause mortality<br>(n per 100 yr)	5	5 or 4.75	0 or -0.25<br>(0 or 5% RRR)
First HF events<br>(n per 100 yr)	10	8 or 7	-2 or -3<br>(20% or 30% RRR)
6MWD – change from baseline (mean±SD)	0±75	30±85	30
KCCQ-CSS – change from baseline (mean±SD)	5±19	10±19	5

Abbreviations: 6MWD = 6-minute walk test distance; HF = heart failure; KCCQ-CSS = Kansas City Cardiomyopathy Questionnaire – Clinical Summary Scale; MTD = maximum tolerated dose; n = number; RRR = relative risk reduction; SD = standard deviation.

In addition, the following imputation rules for 6MWD and KCCQ-CSS at Week 52 are also built into the simulations: worst case imputation (assign 0 steps or a score of 0) for missing due to death (assume 5% death rate) and placebo imputation for 10% participants who discontinue study treatment due to AE.

9.3. Populations for Analyses

The following populations are defined:

Analysis Population	Description
Entered	All participants who sign the informed consent form
Randomized/Intent-to-Treat (ITT) Population	All participants assigned to treatment, regardless of whether they take any doses of study treatment, or if they took the correct treatment. Participants will be analyzed according to the treatment group to which they were assigned.
Safety Population	All participants in ITT population who take at least 1 dose of study treatment. Participants will be analyzed according to the treatment group to which they were assigned.

9.4. Statistical Analyses

9.4.1. General Considerations

Statistical analysis of this study will be the responsibility of sponsor or its designee.

Unless specified otherwise, all tests of treatment effects will be conducted at a 2-sided alpha level of 0.05 and all confidence intervals will be given at a 2-sided 95% level. Efficacy will be assessed using ITT Population and safety will be assessed using the Safety Population.

Any change to the data analysis methods described in the protocol will require an amendment only if it changes a principal feature of the protocol. Any other change to the data analysis methods described in the protocol, and the justification for making the change, will be described in the SAP and the CSR. Additional exploratory analyses of the data will be conducted as deemed appropriate.

The SAP will be completed prior to first unblinding and any subsequent amendments will be documented, with final amendments finalized prior to final database lock. The SAP will include a more technical and detailed description of the statistical analyses described in this section. This section is a summary of the planned statistical analyses of the most important endpoints including primary and key secondary endpoints.

9.4.2. Primary Endpoint(s)

The primary estimand for primary endpoints is to assess the treatment difference between tirzepatide and placebo relative to the efficacy measures for all randomized participants, and treatment policy strategy will be used to handle all intercurrent events, which is, all the observed values for the variable of interest are used regardless of whether the intercurrent event occurs. The endpoint and population-level summary for the estimand is described in Section 9.4.2.1 and Section 9.4.2.2 for each primary endpoint.

9.4.2.1. The Hierarchical Composite Endpoint

The analysis of the primary hierarchical composite endpoint will be performed with the Finkelstein-Schoenfeld method, and the win ratio (Pocock et al. 2012) will be reported as the measure of treatment effect. The population-level summary of win ratio will be calculated as number of pairs of tirzepatide-treated participant "wins" divided by number of pairs of placebo-treated participant "wins."

The Finkelstein-Schoenfeld method is based on the principle that each participant is compared with every other participant within each stratum in a pairwise manner that proceeds in a hierarchical fashion. Participants will be stratified according to HF decompensation within 12 months of screening (Y/N), diagnosed T2DM (Y/N), and baseline BMI ≥35 kg/m^2 (Y/N), yielding 8 stratification pools. For the primary composite endpoint, each pairwise comparison will proceed in the following order and a winner has:

- A delayed first occurrence of all-cause death;
- If the pair cannot be differentiated based on mortality, a winner has fewer HF events;
- If the pair cannot be differentiated by number of HF events, a winner has delayed time to the occurrence of first HF event;
- If the pair still cannot be differentiated, a winner has a more favorable category for change from baseline in 6MWD at Week 52;
- If the pair still cannot be differentiated, a winner has a more favorable category for change from baseline in KCCQ-CSS at Week 52;
- Otherwise the pair will be recorded as tied.

The categories for change from baseline in 6MWD are: 1) ≥30% worsening; 2) ≥20% and <30% worsening; 3) ≥10% and <20% worsening; 4) No change (less than 10% change); 5) ≥10% and <20% improvement; 6) ≥20% and <30% improvement; and 7) ≥30% improvement.

The categories for change from baseline in KCCQ-CSS are: 1) ≥10-point worsening; 2) ≥5- but <10-point worsening; 3) No change (<5-point change); 4) ≥5- but <10-point improvement; 5) ≥10-point improvement.

In the pairwise comparison for all-cause mortality and HF events, the censoring for death and HF events will be handled based on the win ratio method (Pocock et al. 2012). When 2 participants have different follow-up times, the shorter follow-up time will be used to compare the clinical outcome measure. Only adjudicated and confirmed endpoint events are included in the primary analysis.

The last measurement prior to randomization for 6MWD and KCCQ-CSS will be used as baseline. Missing 6MWD and KCCQ-CSS values at Week 52 will be imputed through multiple

imputations based on the reason of missingness with details described in the SAP; the statistical inference over multiple imputations will be guided by the method proposed by Rubin (1987).

9.4.2.2. Change from Baseline in 6-Minute Walk Test Distance

For the primary endpoint of change from baseline to Week 52 in 6MWD, a stratified Wilcoxon (Van Elteren) test will be used as the primary analysis method, controlling for the stratification factors of HF decompensation within 12 months of screening (Y/N), diagnosed T2DM (Y/N), and baseline BMI ≥35 kg/m^2 (Y/N). Population-level summary of Hodges-Lehmann estimate for the median difference and corresponding confidence interval will be reported.

Missing 6MWD at Week 52 will be imputed through multiple imputations based on the reason of missingness with details described in the SAP. The statistical inference over multiple imputations will be guided by the method proposed by Rubin (1987).

9.4.3. Key Secondary Endpoint(s)

Analyses for the key secondary endpoints will also be guided by the treatment policy strategy.

Change from baseline in KCCQ-CSS at Week 52 and change from baseline in 6MWD at Week 24 will be analyzed using the same nonparametric approach as described in Section 9.4.2.2.

Percent change from baseline in body weight will be analyzed using an MMRM analysis. The MMRM model will include the categorical effect of treatment, time, treatment-by-time interaction, stratification factors, and the continuous covariate of baseline body weight value. Missing data will be imputed through multiple imputations based on the reason of missingness with details described in the SAP.

Change in NYHA class (improved, no change, or worsened) from baseline will be analyzed using a longitudinal proportional odds model. Missing data will be imputed based on the reason of missingness as described in the SAP.

9.4.4. Tertiary/Exploratory Endpoint(s)

The analyses for exploratory endpoints will be described in the SAP. Statistical tests will be performed at the two-sided significance level of 0.05. There will be no multiplicity adjustment for any analysis of exploratory variables unless specified otherwise. Missing values will not be explicitly imputed unless specified otherwise.

9.4.5. Other Safety Analyses

Safety will be assessed by summarizing and analyzing AEs, special safety topics, laboratory analytes, and vital signs. All safety analyses will be made on the Safety Population. Unless specified otherwise, all data obtained during study period from Safety Population, regardless of adherence to study drug, will be used for safety analyses. The details for safety analysis will be described in the SAP.

Adverse events will be coded from the actual term using MedDRA and reported with preferred terms and system organ class.

9.4.5.1. Evaluation of Immunogenicity

The frequency and percentage of participants with preexisting ADAs and with treatment-emergent ADAs to tirzepatide will be tabulated. Treatment-emergent ADAs are defined as those with a titer 2-fold (1 dilution) greater than the minimum required dilution of the ADA assay if no ADAs were detected at baseline (treatment-induced ADA), or those with a 4-fold (2 dilutions) increase in titer compared with baseline if ADAs were detected at baseline (treatment-boosted ADA). The details of analyses for immunogenicity will be specified in SAP.

9.4.6. Subgroup Analyses

Subgroup variables to be evaluated for the primary efficacy endpoint may include demography (for example, race, ethnicity), baseline disease characteristics (for example, diagnosed T2DM) and others. Subgroup analyses may also be performed for selected key secondary efficacy endpoints. Details for the subgroup analyses will be provided in the SAP.

9.5. Interim Analyses

Based on the projected enrollment, approximately 4 interim analyses of safety will be conducted. The first interim analysis is planned to occur when approximately 20% of the anticipated number of participants are randomized or 6 months after the first participant is randomized, whichever occurs later, followed by subsequent reviews approximately every 6 months throughout the study.

The DMC is authorized to evaluate unblinded interim analyses. Study sites will receive information about interim results only if they need to know for the safety of their participants.

Unblinding details are specified in a separate unblinding plan document.

The DMC charter will describe the planned interim analyses in detail.

9.6. Data Monitoring Committee

An independent DMC with members all external to the sponsor will be used to monitor participant safety in an unblinded fashion. For details on the DMC, refer to the DMC charter.

10. Supporting Documentation and Operational Considerations

10.1. Appendix 1: Regulatory, Ethical, and Study Oversight Considerations

10.1.1. Regulatory and Ethical Considerations

- This study will be conducted in accordance with the protocol and with the following:
  - Consensus ethical principles derived from international guidelines including the Declaration of Helsinki and CIOMS International Ethical Guidelines
  - Applicable ICH GCP guidelines

  - Applicable laws and regulations

- The protocol, protocol amendments, ICF, IB, and other relevant documents (for example, advertisements) must be submitted to an IRB/IEC by the investigator and reviewed and approved by the IRB/IEC before the study is initiated.
- Any amendments to the protocol will require IRB/IEC approval before implementation of changes made to the study design, except for changes necessary to eliminate an immediate hazard to study participants.
- The investigator will be responsible for the following:
  - Providing written summaries of the status of the study to the IRB/IEC annually or more frequently in accordance with the requirements, policies, and procedures established by the IRB/IEC
  - Notifying the IRB/IEC of SAEs or other significant safety findings as required by IRB/IEC procedures
  - Providing oversight of study conduct for participants under their responsibility and adherence to requirements of 21 CFR, ICH guidelines, the IRB/IEC, European regulation 536/2014 for clinical studies (if applicable), and all other applicable local regulations
- Investigator sites are compensated for participation in the study as detailed in the clinical trial agreement.

10.1.2. Financial Disclosure

Investigators and sub-investigators will provide the sponsor with sufficient, accurate financial information as requested to allow the sponsor to submit complete and accurate financial certification or disclosure statements to the appropriate regulatory authorities. Investigators are responsible for providing information on financial interests during the course of the study and for 1 year after completion of the study.

10.1.3. Informed Consent Process

The investigator or his/her representative will explain the nature of the study, including the risks and benefits, to the participant or his/her legally authorized representative and answer all questions regarding the study.

Participants must be informed that their participation is voluntary. Participants or their legally authorized representative will be required to sign a statement of informed consent that meets the requirements of 21 CFR 50, local regulations, ICH guidelines, HIPAA requirements, where applicable, and the IRB/IEC or study center.

The medical record must include a statement that written informed consent was obtained before the participant was entered in the study and the date the written consent was obtained. The authorized person obtaining the informed consent must also sign the ICF.

Participants must be re-consented to the most current version of the ICF(s) during their participation in the study.

A copy of the ICF(s) must be provided to the participant or the participant's legally authorized representative and is kept on file.

Participants who are rescreened are required to sign a new ICF.

10.1.4. Data Protection

Participants will be assigned a unique identifier by the sponsor. Any participant records, datasets, or tissue samples that are transferred to the sponsor will contain the identifier only; participant names or any information which would make the participant identifiable will not be transferred.

The participant must be informed that his/her personal, study-related data will be used by the sponsor in accordance with local data protection law. The level of disclosure must also be explained to the participant who will be required to give consent for his/her data to be used as described in the informed consent.

The participant must be informed that his/her medical records may be examined by Clinical Quality Assurance auditors or other authorized personnel appointed by the sponsor, by appropriate IRB/IEC members, and by inspectors from regulatory authorities.

The sponsor has processes in place to ensure data protection, information security, and data integrity. These processes include appropriate contingency plan(s) for appropriate and timely response in the event of a data security breach.

10.1.5. Dissemination of Clinical Study Data

Report Preparation

An investigator will sign the final CSR for this study, indicating agreement that, to the best of his or her knowledge, the report accurately describes the conduct and results of the study.

Public Access to Reports and Data

Reports

The sponsor will disclose a summary of study information, including tabular study results, on publicly available websites where required by local law or regulation.

Data

The sponsor provides access to all individual participant data collected during the trial, after anonymization, with the exception of PK, immunogenicity, or genetic data. Data are available to request 6 months after the indication studied has been approved in the United States and after primary publication acceptance, whichever is later. No expiration date of data requests is currently set once they are made available. Access is provided after a proposal has been approved by an independent review committee identified for this purpose and after receipt of a signed data sharing agreement. Data and documents, including the study protocol, SAP, CSR, and blank or annotated CRFs, will be provided in a secure data sharing environment for up to 2 years per proposal. For details on submitting a request, see the instructions provided at www.clinicalstudydatarequest.com.

Publications/Publication Policy

The publication policy is described in Section 10.1.9.

10.1.6. Data Quality Assurance

All participant data relating to the study will be recorded on printed or electronic CRF unless transmitted to the sponsor or designee electronically (for example, laboratory data or an electronic source, such as eCOA). The investigator is responsible for verifying that data entries are accurate and correct by physically or electronically signing the CRF.

The investigator must maintain accurate documentation (source data) that supports the information entered in the CRF.

The investigator must permit study-related monitoring, audits, IRB/IEC review, and regulatory agency inspections and provide direct access to source data documents.

Monitoring details describing strategy (for example, risk-based initiatives in operations and quality such as risk management and mitigation strategies and analytical risk-based monitoring), methods, responsibilities, and requirements, including handling of noncompliance issues and monitoring techniques, are provided in the Monitoring Plan.

The sponsor or designee is responsible for the data management of this study including quality checking of the data.

The sponsor assumes accountability for actions delegated to other individuals (for example, contract research organizations).

Study monitors will perform ongoing source data verification to confirm that data transcribed into the CRF by authorized site personnel are accurate, complete, and verifiable from source documents; that the safety and rights of participants are being protected; and that the study is being conducted in accordance with the currently approved protocol and any other study agreements, ICH GCP, and all applicable regulatory requirements.

Records and documents, including signed ICFs, pertaining to the conduct of this study must be retained by the investigator for the time period outlined in the clinical trial agreement unless local regulations or institutional policies require a longer retention period. No records may be destroyed during the retention period without the written approval of the sponsor. No records may be transferred to another location or party without written notification to the sponsor.

In addition, the sponsor or its representatives will periodically check a sample of the participant data recorded against source documents at the study site. The study may be audited by the sponsor or its representatives, and/or regulatory agencies at any time. Investigators will be given notice before an audit occurs.

Data Capture System

The investigator is responsible for ensuring the accuracy, completeness, legibility, and timeliness of the data reported to the sponsor.

An EDC system will be used in this study for the collection of CRF data. The investigator maintains a separate source for the data entered by the investigator or designee into the sponsor-provided EDC system. The investigator is responsible for the identification of any data to be considered source and for the confirmation that data reported are accurate and complete by signing the CRF.

Additionally, eCOA data (patient-reported outcomes instruments) will be directly recorded by the participant, into a device (for example, hand-held smart phone or tablet). The eCOA data will serve as the source documentation, and the investigator does not maintain a separate, written or electronic record of these data.

Data collected via the sponsor-provided data capture systems will be stored at third parties. The investigator will have continuous access to the data during the study and until decommissioning of the data capture system(s). Prior to decommissioning, the investigator will receive an archival copy of pertinent data for retention.

Data managed by a central vendor, such as laboratory test data, will be stored electronically in the central vendor's database system, and reports (as applicable) will be provided to the investigator for review and retention. Data will subsequently be transferred from the central vendor to the sponsor data warehouse.

Data from complaint forms submitted to the sponsor will be encoded and stored in the global product complaint management system.

10.1.7. Source Documents

Source documents provide evidence for the existence of the participant and substantiate the integrity of the data collected. Source documents are filed at the investigator's site.

Data reported on the CRF or entered in the eCRF that are transcribed from source documents must be consistent with the source documents, or the discrepancies must be explained. The investigator may need to request previous medical records or transfer records, depending on the study. Also, current medical records must be available.

Definition of what constitutes source data can be found in Section 10.1.6.

10.1.8. Study and Site Start and Closure

The study start date is the date on which the clinical study will be open for recruitment of participants.

The first act of recruitment is the first site open and will be the study start date.

The sponsor designee reserves the right to close the study site or terminate the study at any time for any reason at the sole discretion of the sponsor. Study sites will be closed upon study completion. A study site is considered closed when all required documents and study supplies have been collected and a study-site closure visit has been performed.

The investigator may initiate study-site closure at any time, provided there is reasonable cause and sufficient notice is given in advance of the intended termination.

Reasons for the early closure of a study site by the sponsor or investigator may include but are not limited to:

- Failure of the investigator to comply with the protocol, the requirements of the IRB/IEC or local health authorities, the sponsor's procedures, or GCP guidelines
- Inadequate recruitment of participants by the investigator
- Discontinuation of further study drug development.

If the study is prematurely terminated or suspended, the sponsor shall promptly inform the investigators, the IECs/IRBs, the regulatory authorities, and any contract research organization(s) used in the study of the reason for termination or suspension, as specified by the applicable regulatory requirements. The investigator shall promptly inform the participant and assures appropriate participant therapy and/or follow-up.

10.1.9. Publication Policy

In accordance with the sponsor's publication policy, the results of this study will be submitted for publication by a peer-reviewed journal.

10.1.10. Long-Term Sample Retention

Sample retention enables use of new technologies, response to regulatory questions, and investigation of variable response that may not be observed until later in the development of the intervention or after the intervention becomes commercially available.

This table describes the retention period for potential sample types.

Sample Type	Custodian	Retention Period After Last Participant Visit
Genetics sample	Sponsor or designee	up to 15 years
Exploratory biomarker sample	Sponsor or designee	up to15 years
Immunogenicity (antidrug antibody) sample	Sponsor or designee	up to 15 years
Pharmacokinetic sample	Sponsor or designee	up to 1 years

10.2. Appendix 2: Clinical Laboratory Tests

- Clinical laboratory testing will be performed according to the SoA (Section 1.3).
- Central and local laboratories will be used. The table below describes when the local or central laboratory will be used
- In circumstances where the sponsor approves local laboratory testing in lieu of central laboratory testing (in the table below), the local laboratory must be qualified in accordance with applicable local regulations.
- Protocol-specific requirements for inclusion or exclusion of participants are detailed in Section 5 of the protocol.
- Additional tests may be performed at any time during the study as determined necessary by the investigator or required by local regulations.
- Pregnancy testing will be performed according to the SoA.
- Investigators must document their review of the laboratory safety results. Laboratory results that will not be reported to investigative sites or other blinded personnel are noted in the table below.

Refer to Section 10.7 for recommended laboratory testing for hypersensitivity events.

Clinical Laboratory Tests	Comments
**Hematology**	Assayed by Lilly-designated laboratory.
Hemoglobin	
Hematocrit	
Erythrocyte count (RBCs [red blood cells])	
Mean cell volume	
Mean cell hemoglobin concentration	
Leukocytes (WBCs [white blood cells])	
Differential	
Neutrophils, segmented	
Lymphocytes	
Monocytes	
Eosinophils	
Basophils	
Platelets	
**Clinical Chemistry**	Assayed by Lilly-designated laboratory.
Sodium	
Potassium	
Chloride	
Bicarbonate	
Total bilirubin	
Direct bilirubin	
Alkaline phosphatase (ALP)	
Alanine aminotransferase (ALT)	

Clinical Laboratory Tests	Comments
Aspartate aminotransferase (AST)	
Gamma-glutamyl transferase (GGT)	
Blood urea nitrogen (BUN)	
Creatinine	
Creatine kinase (CK)	
Uric acid	
Total protein	
Albumin	
Calcium	
Phosphorus	
Glucose	
Lactate dehydrogenase (LDH)	
**Lipids**	
Total cholesterol	
Direct LDL-C	
HDL-C	
VLDL-C	
Triglycerides	
**Pancreas (Exocrine)**	Assayed by Lilly-designated laboratory.
Pancreatic amylase	
Lipase	
**Special Chemistry**	Assayed by Lilly-designated laboratory.
Hemoglobin A1c (HbA1c)	
Calcitonin	
Cystatin C	
N-terminal pro b-type natriuretic peptide (NT-proBNP)	
Cardiac troponin T (cTnT)	
C-reactive protein, high-sensitivity (hsCRP)	
Thyroid stimulating hormone	
**Urine Chemistry**	Assayed by Lilly-designated laboratory.
Albumin	
Creatinine	
**Calculation**	
eGFR (calculated by CKD-EPI equation)	Will be calculated by the Lilly-designated laboratory at all visits.
Urine albumin, creatinine, UACR	
**Hormones (female)**	
Urine Pregnancy	Local laboratory
Serum Pregnancy	Assayed by Lilly-designated laboratory.
Follicle Stimulating Hormone (FSH)	Assayed by Lilly-designated laboratory.
**Pharmacokinetic Samples**	• Assayed by Lilly-designated laboratory.<br>• Results will not be provided to the investigative sites.

Clinical Laboratory Tests	Comments
	• In the event of systemic drug hypersensitivity reactions (immediate or nonimmediate), additional blood samples will be collected including ADA, PK, and exploratory biomarker samples. PK samples for immunogenicity must be taken prior to drug administration.
**Genetics sample**<br>Whole blood (EDTA)	• Assayed by Lilly-designated laboratory.<br>• Results will not be provided to the investigative sites.
**Exploratory Biomarker Samples**	• Assayed by Lilly-designated laboratory.<br>• Results will not be provided to the investigative sites.
Serum	
EDTA Plasma	
P800 Plasma	
**Immunogenicity Samples**	
Anti-tirzepatide antibodies<br>Anti-tirzepatide neutralizing antibodies	• Assayed by Lilly-designated laboratory.<br>• Results will not be provided to the investigative sites.<br>• In the event of systemic drug hypersensitivity reactions (immediate or nonimmediate), additional blood samples will be collected including ADA, PK, and exploratory biomarker samples. PK samples for immunogenicity must be taken prior to drug administration.

Abbreviations: ADA = antidrug antibody; CKD-EPI = Chronic Kidney Disease-Epidemiology Collaboration; EDTA = ethylenediaminetetraacetic acid; eGFR = estimated glomerular filtration rate; HDL-C = high-density lipoprotein cholesterol; LDL-C = low-density lipoprotein cholesterol; Lilly = Eli Lilly and company; PK = pharmacokinetic; UACR = urine albumin/creatinine ratio; VLDL-C = very low-density lipoprotein cholesterol.

10.3. Appendix 3: Adverse Events: Definitions and Procedures for Recording, Evaluating, Follow-up, and Reporting

The definitions and procedures detailed in this appendix are in accordance with ISO 14155.

Both the investigator and the sponsor will comply with all local medical device reporting requirements.

The detection and documentation procedures described in this protocol apply to all sponsor medical devices provided for use in the study. See Section 6.1.1 for the list of sponsor medical devices).

10.3.1. Definition of AE

**AE Definition**
• An AE is any untoward medical occurrence in a participant administered a pharmaceutical product and which does not necessarily have a causal relationship with the study drug. An AE can therefore be any unfavourable and unintended sign (including an abnormal laboratory finding), symptom, or disease (new or exacerbated) temporally associated with the use of a medicinal (investigational) product, whether or not related to the medicinal (investigational) product.<br>• An AE is any untoward medical occurrence, unintended disease or injury, or untoward clinical signs (including abnormal laboratory finding) in study participants, users, or other persons, whether or not related to the investigational medical device. This definition includes events related to the investigational medical device or comparator and events related to the procedures involved except for events in users or other persons, which only include events related to investigational devices.
**Events Meeting the AE Definition**
• Any abnormal laboratory test results (hematology, clinical chemistry, or urinalysis) or other safety assessments (for example, ECG, radiological scans, vital signs measurements), including those that worsen from baseline, considered clinically significant in the medical and scientific judgment of the investigator (that is, not related to progression of underlying disease).<br>• Exacerbation of a chronic or intermittent preexisting condition including either an increase in frequency and/or intensity of the condition.<br>• New conditions detected or diagnosed after study drug administration even though they may have been present before the start of the study.<br>• Signs, symptoms, or the clinical sequelae of a suspected drug-drug interaction.<br>• Signs, symptoms, or the clinical sequelae of a suspected overdose of either study drug or a concomitant medication. Overdose per se will not be reported as an AE/SAE unless it is an intentional overdose taken with possible suicidal/self-harming intent. Such an overdose should be reported regardless of sequelae.<br>• "Lack of efficacy" or "failure of expected pharmacological action" per se will not be reported as an AE or SAE. Such instances will be captured in the efficacy assessments.

However, the signs, symptoms, and/or clinical sequelae resulting from lack of efficacy will be reported as an AE or SAE if they fulfill the definition of an AE or SAE.
**Events NOT Meeting the AE Definition**
• Any clinically significant abnormal laboratory findings or other abnormal safety assessments which are associated with the underlying disease, unless judged by the investigator to be more severe than expected for the participant's condition.<br>• The disease/disorder being studied or expected progression, signs, or symptoms of the disease/disorder being studied, unless more severe than expected for the participant's condition.<br>• Medical or surgical procedure (for example, endoscopy, appendectomy): the condition that leads to the procedure is the AE.<br>• Situations in which an untoward medical occurrence did not occur (social and/or convenience admission to a hospital).<br>• Anticipated day-to-day fluctuations of preexisting disease(s) or condition(s) present or detected at the start of the study that do not worsen.

10.3.2. Definition of SAE

If an event is not an AE per the definition above, then it cannot be an SAE even if serious conditions are met (for example, hospitalization for signs/symptoms of the disease under study, death due to progression of disease).

**SAE is defined as any untoward medical occurrence that, at any dose:**
**a. Results in death**
**b. Is life-threatening**<br>The term 'life-threatening' in the definition of 'serious' refers to an event in which the participant was at risk of death at the time of the event. It does not refer to an event, which hypothetically might have caused death, if it were more severe.
**c. Requires inpatient hospitalization or prolongation of existing hospitalization**<br>• In general, hospitalization signifies that the participant has been admitted to the hospital for observation and/or treatment that would not have been appropriate in the physician's office or outpatient setting. Complications that occur during hospitalization are AEs. If a complication prolongs hospitalization or fulfills any other serious criteria, the event is serious. When in doubt as to whether "hospitalization" occurred or was necessary, the AE should be considered serious.<br>• Hospitalization for elective treatment of a preexisting condition that did not worsen from baseline is not considered an AE.
**d. Results in persistent disability/incapacity**

- The term disability means a substantial disruption of a person's ability to conduct normal life functions.
- This definition is not intended to include experiences of relatively minor medical significance such as uncomplicated headache, nausea, vomiting, diarrhea, influenza, and accidental trauma (for example, sprained ankle) which may interfere with or prevent everyday life functions but do not constitute a substantial disruption.

e. Is a congenital anomaly/birth defect

- Abnormal pregnancy outcomes (for example, spontaneous abortion, fetal death, stillbirth, congenital anomalies, ectopic pregnancy) are considered SAEs.

f. Other situations:

- Medical or scientific judgment should be exercised in deciding whether SAE reporting is appropriate in other situations such as important medical events that may not be immediately life-threatening or result in death or hospitalization but may jeopardize the participant or may require medical or surgical intervention to prevent one of the other outcomes listed in the above definition. These events should usually be considered serious.
- Examples of such events include invasive or malignant cancers; intensive treatment in an emergency room or at home for allergic bronchospasm, blood dyscrasias, or convulsions that do not result in hospitalization; or development of drug dependency or drug abuse.

g. Resulted in medical or surgical intervention to prevent life-threatening illness or injury or permanent impairment to a body structure or a body function.

Definition of Serious Adverse Device Effect (SADE)

An SADE is defined as an adverse device effect that has resulted in any of the consequences characteristic of a serious adverse event.

Definition of Unanticipated Adverse Device Effect (UADE)

A UADE is a serious adverse effect on health or safety or any life-threatening problem or death caused by or associated with a device, if that effect, problem, or death was not previously identified in nature, severity, or degree of incidence in the investigational plan or application (including a supplementary plan or application) or any other unanticipated serious problem associated with a device that relates to the rights, safety, or welfare of the participant.

10.3.3. Definition of Product Complaints

Product Complaint
• A product complaint is any written, electronic, or oral communication that alleges deficiencies related to the identity, quality, durability, reliability, safety, effectiveness, or performance of a study drug. When the ability to use the study drug safely is impacted, the following are also product complaints:<br>o Deficiencies in labeling information, and<br>o Use errors for device or drug-device combination products due to ergonomic design elements of the product.<br>• Product complaints related to study drugs used in clinical trials are collected in order to ensure the safety of participants, monitor quality, and to facilitate process and product improvements.<br>• Investigators will instruct participants to contact the site as soon as possible if he or she has a product complaint or problem with the study drug so that the situation can be assessed.<br>• An event may meet the definition of both a product complaint and an AE/SAE. In such cases, it should be reported as both a product complaint and as an AE/SAE.

10.3.4. Recording and Follow-Up of AE and/or SAE and Product Complaints

AE, SAE, and Product Complaint Recording
When an AE/SAE/product complaint occurs, it is the responsibility of the investigator to review all documentation (for example, hospital progress notes, laboratory reports, and diagnostics reports) related to the event.<br>The investigator will then record all relevant AE/SAE/product complaint information in the participant's medical records, in accordance with the investigator's normal clinical practice. AE/SAE information is reported on the appropriate eCRF page and product complaint information is reported on the Product Complaint Form.<br>Note: An event may meet the definition of both a product complaint and an AE/SAE. In such cases, it should be reported as both a product complaint and as an AE/SAE.<br>It is **not** acceptable for the investigator to send photocopies of the participant's medical records to sponsor or designee in lieu of completion of the eCRF page for AE/SAE and the Product Complaint Form for product complaints.<br>There may be instances when copies of medical records for certain cases are requested by sponsor or designee. In this case, all participant identifiers, with the exception of the participant number, will be redacted on the copies of the medical records before submission to sponsor or designee.<br>The investigator will attempt to establish a diagnosis of the event based on signs, symptoms, and/or other clinical information. Whenever possible, the diagnosis (not the individual signs/symptoms) will be documented as the AE/SAE.

Assessment of Intensity
The investigator will make an assessment of intensity for each AE and SAE reported during the study and assign it to one of the following categories:<br>• Mild: A type of adverse event that is usually transient and may require only minimal treatment or therapeutic intervention. The event does not generally interfere with usual activities of daily living.<br>• Moderate: A type of adverse event that is usually alleviated with additional specific therapeutic intervention. The event interferes with usual activities of daily living, causing discomfort but poses no significant or permanent risk of harm to the research participant.<br>• Severe: A type of adverse event that interrupts usual activities of daily living, or significantly affects clinical status, or may require intensive therapeutic intervention. An AE that is assessed as severe should not be confused with an SAE. Severe is a category utilized for rating the intensity of an event; and both AEs and SAEs can be assessed as severe.<br>An event is defined as 'serious' when it meets at least 1 of the predefined outcomes as described in the definition of an SAE, NOT when it is rated as severe.
**Assessment of Causality**
• The investigator is obligated to assess the relationship between study drug and each occurrence of each AE/SAE.<br>• A "reasonable possibility" of a relationship conveys that there are facts, evidence, and/or arguments to suggest a causal relationship, rather than a relationship cannot be ruled out.<br>• The investigator will use clinical judgment to determine the relationship.<br>• Alternative causes, such as underlying disease(s), concomitant therapy, and other risk factors, as well as the temporal relationship of the event to study drug administration will be considered and investigated.<br>• The investigator will also consult the IB in his/her assessment.<br>• For each AE/SAE, the investigator **must** document in the medical notes that he/she has reviewed the AE/SAE and has provided an assessment of causality.<br>• There may be situations in which an SAE has occurred, and the investigator has minimal information to include in the initial report to sponsor or designee. However, it is very important that the investigator always make an assessment of causality for every event before the initial transmission of the SAE data to sponsor or designee.<br>• The investigator may change his/her opinion of causality in light of follow-up information and send an SAE follow-up report with the updated causality assessment.<br>• The causality assessment is one of the criteria used when determining regulatory reporting requirements.

Follow-up of AEs and SAEs
• The investigator is obligated to perform or arrange for the conduct of supplemental measurements and/or evaluations as medically indicated or as requested by sponsor or designee to elucidate the nature and/or causality of the AE or SAE as fully as possible. This may include additional laboratory tests or investigations, histopathological examinations, or consultation with other health care professionals.

10.3.5. Reporting of SAEs

SAE Reporting via an Electronic Data Collection Tool
• The primary mechanism for reporting an SAE will be the electronic data collection tool.<br>• If the electronic system is unavailable, then the site will use the paper SAE data collection tool (see next section) in order to report the event within 24 hours.<br>• The site will enter the SAE data into the electronic system as soon as it becomes available.<br>• After the study is completed at a given site, the electronic data collection tool will be taken offline to prevent the entry of new data or changes to existing data.<br>• If a site receives a report of a new SAE from a study participant or receives updated data on a previously reported SAE after the electronic data collection tool has been taken off-line, then the site can report this information on a paper SAE form (see next section) or to the sponsor by telephone.<br>• Contacts for SAE reporting can be found in the study training.

10.3.6. Regulatory Reporting Requirements

SAE Regulatory Reporting
• Prompt notification by the investigator to the sponsor of an SAE is essential so that legal obligations and ethical responsibilities towards the safety of participants and the safety of a study drug under clinical investigation are met.<br>• The sponsor has a legal responsibility to notify both the local regulatory authority and other regulatory agencies about the safety of a study drug under clinical investigation. The sponsor will comply with country-specific regulatory requirements relating to safety reporting to the regulatory authority, Institutional Review Boards (IRB)/Independent Ethics Committees (IEC), and investigators.<br>• An investigator who receives an investigator safety report describing an SAE or other specific safety information (for example, summary or listing of SAEs) from the sponsor will review and then file it along with the IB and will notify the IRB/IEC, if appropriate according to local requirements.<br>• As required by local regulations, the investigator will report to their IRB/IEC any UADE (unanticipated problem that resulted in an SAE), or any product complaint that could have led to an SAE had precautions not been taken.

10.4. Appendix 4: Contraceptive Guidance and Collection of Pregnancy Information

10.4.1. Male participants:

Men, regardless of their fertility status, with nonpregnant WOCBP partners must agree to either remain abstinent (if this is their preferred and usual lifestyle) or use condoms, as well as 1 additional highly effective (<1% failure rate) method of contraception (such as combination oral contraceptives, implanted contraceptives, or intrauterine devices) or effective method of contraception (such as diaphragms with spermicide or cervical sponges) for the duration of the study and until their plasma concentrations are below the level that could result in a relevant potential exposure to a possible fetus, predicted to be 90 days plus 5 half-lives following the last dose of study drug, which is approximately 4 months after the last injection.

a) Men and their partners may choose to use a double-barrier method of contraception. (Barrier protection methods without concomitant use of a spermicide are not an effective or acceptable method of contraception. Thus, each barrier method must include use of a spermicide. It should be noted, however, that the use of male and female condoms as a double-barrier method is not considered acceptable due to the high failure rate when these barrier methods are combined.)

b) Periodic abstinence (for example, calendar, ovulation, symptothermal, or postovulation methods), declaration of abstinence just for the duration of a trial, and withdrawal are not acceptable methods of contraception.

Men with pregnant partners should use condoms during intercourse for the duration of the study and until the end of the estimated, relevant potential exposure in WOCBP (4 months).

Men should refrain from sperm donation for the duration of the study and until their plasma concentrations are below the level that could result in a relevant potential exposure to a possible fetus, predicted to be 90 days plus 5 half-lives following the last dose of study drug, which is approximately 4 months.

Men who are in exclusively same-sex relationships (as their preferred and usual lifestyle) are not required to use contraception.

10.4.2. Female participants:

Women of childbearing potential who are abstinent (if this is complete abstinence, as their preferred and usual lifestyle) or in a same-sex relationship (as part of their preferred and usual lifestyle) must agree to either remain abstinent or stay in a same-sex relationship without sexual relationships with males. Periodic abstinence (for example, calendar, ovulation, symptothermal, or postovulation methods), declaration of abstinence just for the duration of a trial, and withdrawal are not acceptable methods of contraception.

Otherwise, WOCBP participating must agree to use 2 forms of effective contraception, where at least 1 form is highly effective (<than 1% failure rate), for the entirety of the study. Contraception must continue following completion of study drug administration for the entirety of the study and for 2 months after the last injection.

a) WOCBP participating must test negative for pregnancy prior to initiation of treatment as indicated by a negative serum pregnancy test at the screening visit followed by a negative urine pregnancy test within 24 hours prior to exposure.

b) Two forms of effective contraception, where at least 1 form is highly effective (such as combination oral contraceptives, implanted contraceptives, or intrauterine devices) will be used for the duration of the trial and for 2 months after the last injection. Effective contraception (such as male or female condoms with spermicide, diaphragms with spermicide, or cervical sponges) may be used as the second therapy. Barrier protection methods without concomitant use of a spermicide are not a reliable or acceptable method. Thus, each barrier method must include use of a spermicide (that is, condom with spermicide, diaphragm with spermicide, or female condom with spermicide). It should be noted that the use of male and female condoms as a double-barrier method is not considered acceptable due to the high failure rate when these methods are combined.

c) Not be breastfeeding.

Women not of childbearing potential may participate and include those who are:

a) Infertile due to surgical sterilization, or

b) Postmenopausal.

Definitions:

Woman of Childbearing Potential (WOCBP)

A woman is considered fertile following menarche and until becoming postmenopausal unless permanently sterile (see below).

If fertility is unclear (for example, amenorrhea in adolescents or athletes) and a menstrual cycle cannot be confirmed before first dose of study drug, additional evaluation should be considered.

Women in the following categories are not considered WOCBP

1. Premenarchal
2. Premenopausal female with 1 of the following:
   - Documented hysterectomy
   - Documented bilateral salpingectomy
   - Documented bilateral oophorectomy

For individuals with permanent infertility due to an alternate medical cause other than the above, (for example, mullerian agenesis, androgen insensitivity), investigator discretion should be applied to determining study entry.

Note: Determination can come from the site personnel's review of the participant's medical records, medical examination, or medical history interview.

3. Postmenopausal female is defined as follows:
   a. A woman at any age at least 6 weeks post-surgical bilateral oophorectomy with or without hysterectomy, confirmed by operative note
   b. A woman at least 40 years of age and up to 55 years old with an intact uterus, not on hormone therapy*, who has had cessation of menses for at least 12 consecutive

months without an alternative medical cause, AND with a follicle-stimulating hormone >40 mIU/mL; or

c. A woman 55 or older not on hormone therapy, who has had at least 12 months of spontaneous amenorrhea, or
d. A woman at least 55 years of age with a diagnosis of menopause prior to starting hormone replacement therapy

* Women should not be taking medications during amenorrhea such as oral contraceptives, hormones, gonadotropin-releasing hormone, anti-estrogens, selective estrogen receptor modulators (SERMs), or chemotherapy that could induce transient amenorrhea.

Contraception Guidance:

Highly Effective Methods of Contraception:

- Combined oral contraceptive pill and mini pill
- NuvaRing®
- Implantable contraceptives
- Injectable contraceptives (such as Depo-Provera®)
- Intrauterine device (such as Mirena® and ParaGard®)
- Contraceptive patch – ONLY women <198 pounds or 90 kg
- Total abstinence (if this is their preferred and usual lifestyle) or in a same-sex relationship with no sexual relationship with males (as part of their preferred and usual lifestyle).

Note: periodic abstinence (for example, calendar, ovulation, symptothermal, postovulation methods), declaration of abstinence just for the duration of a trial, and withdrawal are not acceptable methods of contraception.

Note: Implantable contraceptives and injectable contraceptives (such as Depo-Provera) are only permitted if started prior to screening. Participants should not start these methods of contraception after being enrolled in the study.

- Vasectomy - for men in clinical trials

Effective Methods of Contraception (must use combination of 2 methods):

- Male condom with spermicide
- Female condom with spermicide
- Diaphragm with spermicide
- Cervical sponge
- Cervical cap with spermicide

Collection of Pregnancy Information

Male participants with partners who become pregnant

- The investigator will attempt to collect pregnancy information on any male participant's female partner who becomes pregnant while the male participant is in this study.
- After obtaining the necessary signed informed consent from the pregnant female partner directly, the investigator will record pregnancy information on the appropriate form and

submit it to the sponsor within 24 hours of learning of the partner's pregnancy. The female partner will also be followed to determine the outcome of the pregnancy. Information on the status of the mother and child will be forwarded to the sponsor. Generally, the follow-up will be no longer than 6 to 8 weeks following the estimated delivery date. Any termination of the pregnancy will be reported regardless of fetal status (presence or absence of anomalies) or indication for the procedure.

Female Participants who become pregnant

- The investigator will collect pregnancy information on any female participant who becomes pregnant while participating in this study. The initial information will be recorded on the appropriate form and submitted to the sponsor within 24 hours of learning of a participant's pregnancy.
- The participant will be followed to determine the outcome of the pregnancy. The investigator will collect follow-up information on the participant and the neonate, and the information will be forwarded to the sponsor. Generally, follow-up will not be required for longer than 6 to 8 weeks beyond the estimated delivery date. Any termination of pregnancy will be reported, regardless of fetal status (presence or absence of anomalies) or indication for the procedure.
- While pregnancy itself is not considered to be an AE or SAE, any pregnancy complication or elective termination of a pregnancy for medical reasons will be reported as an AE or SAE.
- A spontaneous abortion (occurring at <20 weeks gestational age) or still birth (occurring at >20 weeks gestational age) is always considered to be an SAE and will be reported as such.
- Any poststudy, pregnancy-related SAE considered reasonably related to the study drug by the investigator will be reported to the sponsor as described in protocol Section 8.3.1. While the investigator is not obligated to actively seek this information in former study participants, he or she may learn of an SAE through spontaneous reporting.
- Any female participant who becomes pregnant while participating in the study will discontinue study drug and be withdrawn from the study.

10.5. Appendix 5: Adverse Events of Special Interest: Definitions and Procedures for Recording, Evaluating, Follow-Up, and Reporting.

10.5.1. Special Safety Topics

10.5.1.1. Hypoglycemia

Upon ICF signing, all participants will be educated about signs and symptoms of hypoglycemia, how to treat hypoglycemia, and how to collect appropriate information for each episode of hypoglycemia.

Hypoglycemia may be identified by spontaneous reporting of symptoms from participants (whether confirmed or unconfirmed by simultaneous glucose values) or by BG samples collected during study visits.

All participants with T2DM and who develop diabetes during the study will be provided with glucometers.

Participants with T2DM will be provided a diary to record relevant information (for example, glucose values, symptoms).

All hypoglycemic episodes are to be recorded on a specific eCRF and should not be otherwise recorded as AEs unless the event meets severe criteria. If a hypoglycemic event meets severe criteria (see definition below), it should be recorded as serious on the AE eCRFs, and reported to the sponsor as an SAE. To avoid duplicate reporting, all consecutive BG values <70 mg/dL (<3.9 mmol/L) occurring within a 1-hour period may be considered to be a single hypoglycemic event (Weinberg et al. 2010; Danne et al. 2013).

Investigators should use the following definitions and criteria when diagnosing and categorizing an episode considered to be related to hypoglycemia (the BG values in this section refer to values determined by a laboratory or International Federation of Clinical Chemistry and Laboratory Medicine blood-equivalent glucose meters and strips) in accordance with the 2020 American Diabetes Association position statement on glycemic targets (ADA 2020):

Glucose Alert Value (Level 1):

- Documented symptomatic hypoglycemia is defined as any time a participant feels that he or she is experiencing symptoms and/or signs associated with hypoglycemia and has a BG level of <70 mg/dL (<3.9 mmol/L).
- Documented asymptomatic hypoglycemia is defined as any event not accompanied by typical symptoms of hypoglycemia, but with a measured BG <70 mg/dL (<3.9 mmol/L).
- Documented unspecified hypoglycemia is defined as any event with no information about symptoms of hypoglycemia available, but with a measured BG <70 mg/dL (<3.9 mmol/L).

Clinically Significant Hypoglycemia (Level 2):

- Documented symptomatic hypoglycemia is defined as any time a participant feels that he or she is experiencing symptoms and/or signs associated with hypoglycemia and has a BG level of <54 mg/dL (<3.0 mmol/L).

- Documented asymptomatic hypoglycemia is defined as any event not accompanied by typical symptoms of hypoglycemia but with a measured BG <54 mg/dL (<3.0 mmol/L).
- Documented unspecified hypoglycemia is defined as any event with no information about symptoms of hypoglycemia available but with a measured BG <54 mg/dL (<3.0 mmol/L).

Severe Hypoglycemia (Level 3):

- Severe hypoglycemia is defined as an episode with severe cognitive impairment requiring the assistance of another person to actively administer carbohydrate, glucagon, or other resuscitative actions. These episodes may be associated with sufficient neuroglycopenia to induce seizure or coma. Blood glucose measurements may not be available during such an event, but neurological recovery attributable to the restoration of BG to normal is considered sufficient evidence that the event was induced by a low BG concentration.

Nocturnal Hypoglycemia:

Nocturnal hypoglycemia is a hypoglycemia event (including severe hypoglycemia) that occurs at night, presumably during sleep.

10.5.1.2. Pancreatitis

Acute pancreatitis is defined as an AE of interest in all trials with tirzepatide, including this trial.

Acute pancreatitis is an acute inflammatory process of the pancreas that may also involve peripancreatic tissues and/or remote organ systems (Banks and Freeman 2006). The diagnosis of acute pancreatitis requires 2 of the following 3 features:

- abdominal pain, characteristic of acute pancreatitis (generally located in the epigastrium and radiates to the back in approximately half the cases) (Banks and Freeman 2006; Koizumi et al. 2006); the pain is often associated with nausea and vomiting
- serum amylase (total and/or pancreatic) and/or lipase ≥3X ULN, and
- characteristic findings of acute pancreatitis on CT scan or MRI.

If acute pancreatitis is suspected, appropriate laboratory tests (including levels of pancreatic amylase and lipase) should be obtained via the central laboratory (and locally, if needed).

Imaging studies, such as abdominal CT scan with or without contrast, MRI, or gallbladder ultrasound, should be performed. Abdominal ultrasound may be used as an alternative method only if CT and MRI cannot be performed. If laboratory values and/or abdominal imaging support the diagnosis of acute pancreatitis, the participant must discontinue therapy with tirzepatide but will continue in the study. A review of the participant's concomitant medications should be conducted to assess any potential causal relationship with pancreatitis.

Each AE of pancreatitis must be reported. If typical signs and/or symptoms of pancreatitis are present and confirmed by laboratory values (lipase or amylase [total and/or pancreatic]) and imaging studies, the event must be reported as an SAE. For a potential case that does not meet all of these criteria, it is up to the investigator to determine the seriousness of the case (AE or SAE) and the relatedness of the event to study drug(s).

Each participant will have measurements of p-amylase and lipase (assessed at the central laboratory) as shown on the SoA (Section 1.3) to assess the effects of the investigational doses of tirzepatide on pancreatic enzyme levels. Serial measurements of pancreatic enzymes have limited clinical value for predicting episodes of acute pancreatitis in asymptomatic participants (Nauck et al. 2017; Steinberg et al. 2017a, 2017b). Thus, further diagnostic follow-up of cases of asymptomatic pancreatic hyperenzymemia (lipase and/or pancreatic amylase ≥3X ULN) is not mandated but may be performed based on the investigator's clinical judgment and assessment of the participant's overall clinical condition. Only cases of pancreatic hyperenzymemia that undergo additional diagnostic follow-up and/or are accompanied by symptoms suggestive of pancreatitis will be submitted for adjudication.

All suspected cases of acute or chronic pancreatitis will be adjudicated by an independent clinical endpoint committee. In addition, AEs of severe or serious abdominal pain of unknown etiology will also be submitted to the adjudication committee to assess for possible pancreatitis or other pancreatic disease. Relevant data from participants with acute or chronic pancreatitis and those with severe or serious abdominal pain will be entered into a specifically designed eCRF page. The adjudication committee representative will enter the results of adjudication in a corresponding eCRF page.

10.5.1.3. Thyroid Malignancies and C-Cell Hyperplasia

Individuals with personal or family history of MTC and/or MEN-2 will be excluded from the study. Participants who are diagnosed with MTC and/or MEN-2 during the study will have study drug stopped and should continue follow-up with an endocrinologist.

The assessment of thyroid safety during the trial will include reporting of any case of thyroid malignancy (including MTC and papillary carcinoma) and measurements of calcitonin. This data will be captured in specific eCRFs. The purpose of calcitonin measurements is to assess the potential of tirzepatide to affect thyroid C-cell function, which may indicate development of C-cell hyperplasia and neoplasms.

10.5.1.4. Calcitonin Measurements

If an increased calcitonin value (see definitions below) is observed in a participant who has been administered a medication that is known to increase serum calcitonin, then this medication should be stopped, and calcitonin levels should be measured after an appropriate washout period.

For participants who require additional endocrine assessment because of increased calcitonin concentration as defined in this section, data from the follow-up assessment will be collected in the specific section of the eCRF.

Calcitonin Measurements in Participants with eGFR ≥60 mL/min/1.73m^2

A significant increase in calcitonin for participants with eGFR ≥60 mL/min is defined below. If a participant's laboratory results meet these criteria, these clinically significant laboratory results should be recorded as an AE.

- calcitonin value ≥20 ng/L and <35 ng/L AND ≥50% increase from the screening value.
    - These participants will be asked to repeat the measurement within 1 month. If this repeat value is increasing (≥10% increase), study drug should be stopped, and the

participant encouraged to undergo additional endocrine assessment and longer-term follow-up by an endocrinologist to exclude any serious adverse effect on the thyroid.

- calcitonin value ≥35 ng/L AND ≥50% over the screening value.
    - In these participants, study drug should be stopped, and the participant recommended to immediately undergo additional endocrine assessments and longer-term follow-up by an endocrinologist.

Calcitonin Measurement in Participants with eGFR <60 mL/min/1.73m^2

A significant increase in calcitonin for participants with eGFR <60 mL/min/1.73m^2 is defined as a calcitonin value ≥35 ng/L AND ≥50% over the screening value. If a participant's labs meet these criteria, these clinically significant labs should be recorded as an AE.

In these participants, study drug should be discontinued (after first confirming the value), and the participant recommended to immediately undergo additional endocrine assessments and longer-term follow-up by an endocrinologist to exclude any serious adverse effect on the thyroid.

10.5.1.5. Major Adverse Cardiovascular Events

Deaths and nonfatal CV AEs will be adjudicated by a committee of physicians external to the sponsor with cardiology expertise. This committee will be blinded to treatment assignment. The nonfatal CV AEs to be adjudicated include:

- myocardial infarction
- hospitalization for unstable angina
- hospitalization for HF
- coronary interventions (such as coronary artery bypass graft or percutaneous coronary intervention), and
- cerebrovascular events, including cerebrovascular accident (stroke) and transient ischemic attack.

10.5.1.6. Supraventricular Arrhythmias and Cardiac Conduction Disorders

Participants who develop any event from these groups of disorders should undergo an ECG, which will be retained at the site as a source document. Additional diagnostic tests to determine exact diagnosis should be performed, as needed. The specific diagnosis will be recorded as an AE. Events that meet criteria for serious conditions as described in Section 10.3.2 must be reported as SAEs. If a clinically significant finding is identified by ECG (including, but not limited to, AF or changes from baseline in corrected QT interval), the investigator or qualified designee will determine if any change in study participant management is needed. This review of the ECG printed at the time of collection must be documented. Any new clinically relevant finding should be reported as an AE.

10.5.1.7. Hypersensitivity Events

All allergic or hypersensitivity reactions will be reported by the investigator as either AEs, or if any serious criterion is met, as SAEs.

In the event of suspected drug hypersensitivity reactions (immediate or nonimmediate) in subjects who experience moderate-to-severe reactions as assessed by the investigator, unscheduled blood samples will be collected as outlined in Appendix 7 (Section 10.7).

Additional data, such as type of reaction and treatment received, will be collected on any AEs or SAEs that the investigator deems related to study drug via the eCRF created for this purpose.

Study drug should be temporarily interrupted in any individual suspected of having a severe or serious allergic reaction to study drug. Study drug may be restarted when/if it is safe to do so, in the opinion of the investigator.

10.5.1.8. Injection Site Reactions

Injection site reactions will be collected on the eCRF separate from the hypersensitivity reaction eCRF. At the time of AE occurrence, samples will be collected for measurement of tirzepatide ADA and tirzepatide concentration.

10.5.1.9. Antidrug Antibodies

The occurrence of ADA formation will be assessed as outlined in Section 8.9.

10.5.1.10. Hepatobiliary Disorders

All events of treatment-emergent biliary colic, cholecystitis, or other suspected events related to gallbladder disease should be evaluated and additional diagnostic tests performed, as needed. In cases of elevated liver markers, hepatic monitoring should be initiated as outlined in Appendix 8 (Section 10.8).

10.5.1.11. Severe Gastrointestinal Adverse Events

Tirzepatide may cause severe GI AEs such as nausea, vomiting, and diarrhea. Information about severe GI AEs as well as antiemetic/antidiarrheal use will be collected in the eCRF/AE form. For detailed information concerning the management of GI AEs, please refer to Section 6.5.

10.5.1.12. Acute Renal Events

Renal safety will be assessed based on repeated renal function assessment as well as assessment of AEs suggestive of acute or worsening of chronic renal failure. Gastrointestinal AEs have been reported with tirzepatide, including nausea, diarrhea, and vomiting. This is consistent with other GLP-1 RAs (Aroda and Ratner 2011). The events may lead to dehydration, which could cause a deterioration in renal function, including acute renal failure.

Participants should be advised to notify investigators in case of severe nausea, frequent vomiting, or symptoms of dehydration.

10.6. Appendix 6: Genetics

Use/Analysis of DNA

- Genetic variation may impact a participant's response to study drug, susceptibility to, severity, and progression of disease. Variable response to study drug may be due to genetic determinants that impact drug absorption, distribution, metabolism, and excretion; mechanism of action of the drug; disease etiology; and/or molecular subtype of the disease being treated. Therefore, where local regulations and IRB/IEC allow, a blood sample will be collected for DNA analysis from consenting participants.
- DNA samples will be used for research related to tirzepatide or HF and related diseases. They may also be used to develop tests/assays including diagnostic tests related to tirzepatide or HF. Genetic research may consist of the analysis of one or more candidate genes, the analysis of genetic markers throughout the genome, or analysis of the entire genome (as appropriate).
- DNA sample analysis may be performed on pharmacogenetic variants thought to play a role in T2DM or CV disease to evaluate their association with observed clinical outcomes to tirzepatide in this study. In the event the observation of a study drug response, the samples may be genotyped, and analysis may be performed to evaluate a genetic association with response to tirzepatide. These investigations may be limited to a focused, candidate-gene study or, if appropriate, genome-wide association studies may be performed to identify regions of the genome associated with the variability observed in drug response. Samples may be used for investigations related to the disease, drug, or class of drugs under study in the context of this clinical program; however, samples may not be used for broad, exploratory, unspecified disease or population genetic analysis. Additional analyses may be conducted if it is hypothesized that this may help further understand the clinical data.
- The samples may be analyzed as part of a multi-study assessment of genetic factors involved in the response to tirzepatide or study drugs of this class to understand the study disease or related conditions.
- The results of genetic analyses may be reported in the CSR or in a separate study summary.
- The sponsor will store the DNA samples in a secure storage space with adequate measures to protect confidentiality.
- The samples will be retained while research on tirzepatide continues but no longer than 15 years or another period as per local requirements (see Section 10.1.10).

10.7. Appendix 7: Recommended Laboratory Testing for Hypersensitivity Events

Laboratory testing should be performed at the time of a systemic hypersensitivity event. The management of the AE may warrant lab testing beyond that described below and should be performed as clinically indicated. Laboratory assessments should be performed if the participant experiences generalized urticaria or if anaphylaxis is suspected.

- Collect the sample after the participant has been stabilized and within 1 to 2 hours of the event; however, samples may be obtained as late as 12 hours after the event as analytes can remain altered for an extended period of time. Record the time at which the sample was collected.
- Obtain a follow-up sample at the next regularly scheduled visit or after approximately 4 weeks, whichever is later.

Clinical Laboratory Tests for Hypersensitivity Events

**Hypersensitivity Tests**	**Notes**<br>Selected test may be obtained in the event of anaphylaxis or systemic allergic/hypersensitivity reactions.
Tirzepatide antidrug antibodies (immunogenicity/ADA)	Assayed by Lilly-designated laboratory.<br>Results will not be provided to the investigative sites.
Tirzepatide concentrations (PK)	Assayed by Lilly-designated laboratory.<br>Results will not be provided to the investigative sites.
Tryptase	Assayed by Lilly-designated laboratory.<br>Results will not be provided to the investigative sites.<br>Urine N-methylhistamine testing is performed in addition to tryptase testing. Collect the first void urine sample following the event. Collect a follow-up urine sample after approximately 4 weeks.<br>**Note:** If a tryptase sample is obtained more than 2 hours after the event (that is, within 2-12 hours), or is not obtained because more than 12 hours have lapsed since the event, collect a urine sample for N-methylhistamine testing.
N-methylhistamine	Will be performed if validated assay is available.<br>Assayed by Lilly-designated laboratory.<br>Results will not be provided to the investigative sites.
Drug-specific IgE	Will be performed if a validated assay is available.<br>Assayed by Lilly-designated laboratory.<br>Results will not be provided to the investigative sites.
Basophil activation test	Will be performed if a validated assay is available.<br>Assayed by Lilly-designated laboratory.<br>Results will not be provided to the investigative sites.<br>**NOTE:** The basophil activation test is an in vitro, cell-based assay that only requires a serum sample. It is a surrogate assay for drug specific-IgE but is not specific for IgE.
Complement (C3, C3a and C5a)	Assayed by Lilly-designated laboratory.<br>Results will not be provided to the investigative sites.
Cytokine panel: IL-6, IL-1β, IL-10	Assayed by Lilly-designated laboratory.<br>Results will not be provided to the investigative sites.

Abbreviations: ADA = antidrug antibody; IgE = immunoglobulin E; IL = interleukin; PK = pharmacokinetic.

10.8. Appendix 8: Liver Safety: Suggested Actions and Follow-Up Assessments

Hepatic Evaluation Testing

See Section 8.2.5.1 for guidance on appropriate test selection.

The Lilly-designated central laboratory must complete the analysis of all selected testing except for microbiology testing.

Local testing may be performed in addition to central testing when necessary for immediate participant management.

Results will be reported if a validated test or calculation is available.

**Hematology**	**Clinical Chemistry**
Hemoglobin	Total bilirubin
Hematocrit	Direct bilirubin
Erythrocytes (red blood cells [RBCs])	Alkaline phosphatase (ALP)
Leukocytes (white blood cells [WBCs])	Alanine aminotransferase (ALT)
Differential:	Aspartate aminotransferase (AST)
Neutrophils, segmented	Gamma-glutamyl transferase (GGT)
Lymphocytes	Creatine kinase (CK)
Monocytes	**Other Chemistry**
Basophils	Acetaminophen
Eosinophils	Acetaminophen protein adducts
Platelets	Alkaline phosphatase isoenzymes
Cell morphology (RBC and WBC)	Ceruloplasmin
**Coagulation**	Copper
	Ethyl alcohol (EtOH)
Prothrombin time, INR (PT-INR)	Haptoglobin
**Serology**	Immunoglobulin IgA (quantitative)
Hepatitis A virus (HAV) testing:	Immunoglobulin IgG (quantitative)
HAV total antibody	Immunoglobulin IgM (quantitative)
HAV IgM antibody	Phosphatidylethanol (PEth)
Hepatis B virus (HBV) testing:	**Urine Chemistry**
Hepatitis B surface antigen (HBsAg)	Drug screen
Hepatitis B surface antibody (anti-HBs)	Ethyl glucuronide (EtG)
Hepatitis B core total antibody (anti-HBc)	**Other Serology**
Hepatitis B core IgM antibody	Antinuclear antibody (ANA)
Hepatitis B core IgG antibody	Anti-smooth muscle antibody (ASMA)[a]

HBV DNA[b]	Anti-actin antibody[c]
Hepatis C virus (HCV) testing:	Epstein-Barr virus (EBV) testing:
HCV antibody	EBV antibody
HCV RNA[b]	EBV DNA[b]
Hepatitis D virus (HDV) testing:	Cytomegalovirus (CMV) testing:
HDV antibody	CMV antibody
Hepatitis E virus (HEV) testing:	CMV DNA[b]
HEV IgG antibody	Herpes simplex virus (HSV) testing:
HEV IgM antibody	HSV (Type 1 and 2) antibody
HEV RNA[b]	HSV (Type 1 and 2) DNA[b]
**Microbiology**[d]	Liver kidney microsomal type 1 (LKM-1) antibody
Culture:	
Blood	
Urine	

Abbreviations: Ig = immunoglobulin; INR = international normalized ratio; PT = prothrombin time.

[a] Not required if anti-actin antibody is tested.

[b] Reflex/confirmation dependent on regulatory requirements, testing availability, or both.

[c] Not required if anti-smooth muscle antibody (ASMA) is tested.

[d] Assayed ONLY by investigator-designated local laboratory; no central testing available.

10.9. Appendix 9: Medical Device Adverse Events (AEs), Adverse Device Effects (ADEs), Serious Adverse Events (SAEs) and Device Deficiencies: Definition and Procedures for Recording, Evaluating, Follow-Up, and Reporting

Refer to Section 10.3 for definitions and procedures for recording, evaluating, follow-up, and reporting of all events.

10.10. Appendix 10: Six-Minute Walk Test

10.10.1. Screening Procedures and Flow Diagram

The flow diagram below details the participant flow and eligibility with the 6MWT.

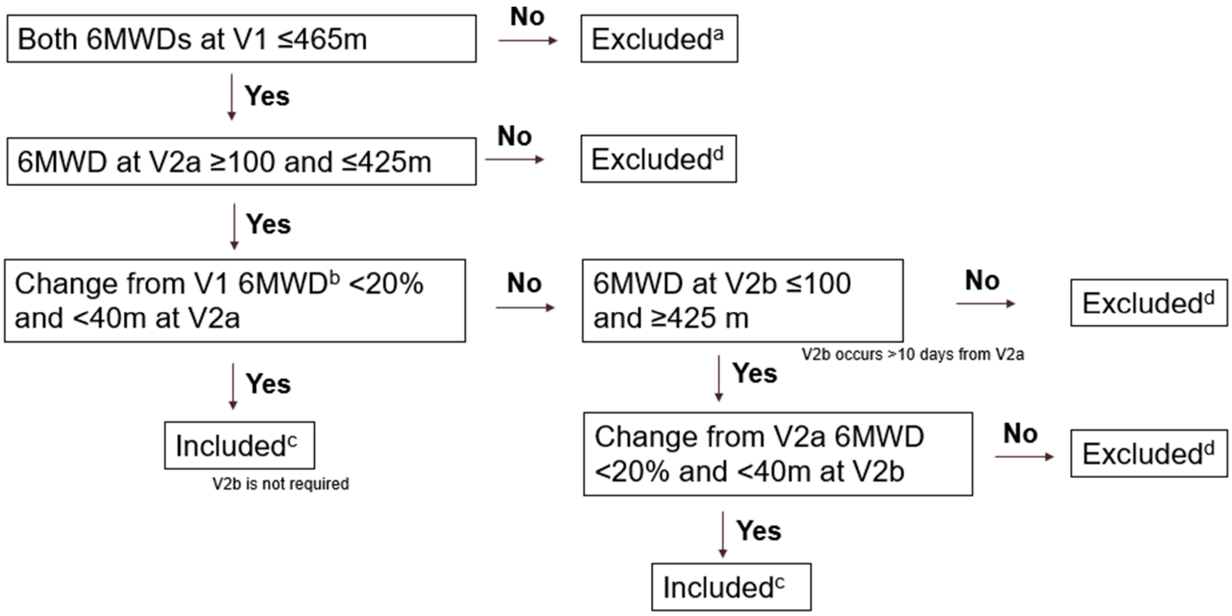

Abbreviations: 6MWD = 6-minute walk test distance; 6MWT = 6-minute walk test; V1 = Visit 1; V2a = Visit 2a; V2b = Visit 2b.

[a] Rescreening is not allowed

[b] Use the higher value of the two Visit 1 6MWD as a reference for Visit 2a

[c] Continue with other Visit 2 assessments according to the SoA

[d] Patients excluded on 6MWT may be re-screened after a minimum of 2 weeks

10.11. Appendix 11: New York Heart Association Classification of Heart Failure

Class	Symptomatology
**I**	No symptoms. Ordinary physical activity such as walking and climbing stairs does not cause fatigue or dyspnea.
**II**	Symptoms with ordinary physical activity. Walking or climbing stairs rapidly, walking uphill, walking or stair climbing after meals, in cold weather, in wind or when under emotional stress causes undue fatigue or dyspnea.
**III**	Symptoms with less than ordinary physical activity. Walking 1-2 blocks on the level and climbing more than one flight of stairs in normal conditions causes undue fatigue or dyspnea.
**IV**	Symptoms at rest. Inability to carry on any physical activity without fatigue or dyspnea.

10.12. Appendix 12: Abbreviations

Term	Definition
**6MWD**	6-minute walk test distance
**6MWT**	6-minute walk test
**ADA**	antidrug antibody
**AE**	adverse event
**AESI**	adverse events of special interest
**AF**	atrial fibrillation
**ALP**	alkaline phosphatase
**ALT**	alanine aminotransferase
**AST**	aspartate aminotransferase
**blinding/masking**	A single-blind study is one in which the investigator and/or his staff are aware of the treatment but the participant is not, or vice versa, or when the sponsor is aware of the treatment but the investigator and/his staff and the participant are not.<br>A double-blind study is one in which neither the participant nor any of the investigator or sponsor staff who are involved in the treatment or clinical evaluation of the subjects are aware of the treatment received.
**BG**	blood glucose
**BMI**	body mass index
**CEC**	clinical endpoint committee
**CFR**	Code of Federal Regulations
**CIOMS**	Council for International Organizations of Medical Sciences
**complaint**	A complaint is any written, electronic, or oral communication that alleges deficiencies related to the identity, quality, purity, durability, reliability, safety or effectiveness, or performance of a drug or drug delivery system.
**compliance**	Adherence to all study-related, good clinical practice (GCP), and applicable regulatory requirements.
**CONSORT**	Consolidated Standards of Reporting Trials
**CRF**	case report form
**CRP**	clinical research physician: Individual responsible for the medical conduct of the study. Responsibilities of the CRP may be performed by a physician, clinical research scientist, global safety physician or other medical officer.

Term	Definition
**CSR**	clinical study report
**CT**	computed tomography
**CV**	cardiovascular
**D Bil**	direct bilirubin
**DMC**	data monitoring committee
**Device Deficiencies**	Equivalent to product complaint
**ECG**	electrocardiogram
**eCOA**	electronic clinical outcome assessment
**eCRF**	electronic case report form
**EDC**	electronic data capture
**eGFR**	estimated glomerular filtration rate
**enroll**	The act of assigning a participant to a treatment. Participants who are enrolled in the study are those who have been assigned to a treatment.
**enter**	Participants entered into a study are those who sign the informed consent form directly or through their legally acceptable representatives.
**ERB**	ethical review board
**ET**	early termination
**EV**	extended visit
**GCP**	good clinical practice
**GGT**	gamma-glutamyl transferase
**GI**	gastrointestinal
**GIP**	glucose-dependent insulinotropic polypeptide
**GLP-1**	glucagon-like peptide-1
**HbA1c**	hemoglobin A1c
**HF**	heart failure
**HFpEF**	heart failure with preserved ejection fraction
**HIPAA**	Health Insurance Portability and Accountability Act

Term	Definition
**IB**	Investigator's Brochure
**ICF**	informed consent form
**ICH**	International Council for Harmonisation
**IEC**	independent ethics committee
**informed consent**	A process by which a participant voluntarily confirms his or her willingness to participate in a particular study, after having been informed of all aspects of the study that are relevant to the participant's decision to participate. Informed consent is documented by means of a written, signed and dated informed consent form.
**INR**	international normalized ratio
**interim analysis**	An interim analysis is an analysis of clinical study data, separated into treatment groups, that is conducted before the final reporting database is created/locked.
**IRB**	institutional review board
**ISO**	International Organization for Standardization
**ITT**	intention to treat: The principle that asserts that the effect of a treatment policy can be best assessed by evaluating on the basis of the intention to treat a participant (that is, the planned treatment regimen) rather than the actual treatment given. It has the consequence that participant allocated to a treatment group should be followed up, assessed, and analyzed as members of that group irrespective of their compliance to the planned course of treatment.
**IV**	intravenous
**IWRS**	interactive web-response system
**KCCQ**	Kansas City Cardiomyopathy Questionnaire
**KCCQ-CSS**	Kansas City Cardiomyopathy Questionnaire – Clinical Summary Scale
**LA**	left atrial
**LAV**	left atrial volume
**LVDEP**	left ventricular end-diastolic pressure
**LVEF**	left ventricular ejection fraction
**MedDRA**	Medical Dictionary for Regulatory Activities
**MEN-2**	multiple endocrine neoplasia type 2
**MMRM**	mixed model repeated measures
**MRI**	magnetic resonance imaging

Term	Definition
**MTC**	medullary thyroid cancer
**MTD**	maximum tolerated dose
**NT-proBNP**	N-terminal pro b-type natriuretic peptide
**NYHA**	New York Heart Association
**participant**	Equivalent to CDISC term "subject": an individual who participates in a clinical trial, either as recipient of an investigational medicinal product or as a control
**PC**	product complaint
**PCWP**	pulmonary capillary wedge pressure
**PK**	pharmacokinetics
**PT**	prothrombin time
**QTc**	corrected QT interval
**QW**	weekly
**RA**	receptor agonist
**SADE**	serious adverse device effect
**SAE**	serious adverse event
**SAP**	statistical analysis plan
**SBP**	systolic blood pressure
**SC**	subcutaneous
**screen**	The act of determining if an individual meets minimum requirements to become part of a pool of potential candidates for participation in a clinical study.
**SoA**	schedule of activities
**study intervention**	for this study, study intervention may be interpreted/synonymous with study drug
**SUSAR**	suspected unexpected serious adverse reaction
**T1DM**	type 1 diabetes mellitus
**T2DM**	type 2 diabetes mellitus
**TBL**	total bilirubin

Term	Definition
**TEAE**	Treatment-emergent adverse event: An untoward medical occurrence that emerges during a defined treatment period, having been absent pretreatment, or worsens relative to the pretreatment state, and does not necessarily have to have a causal relationship with this treatment.
**UADE**	unanticipated adverse device effect
**ULN**	upper limit of normal
**WOCBP**	women of childbearing potential

11. References

[ADA] American Diabetes Association. Glycemic targets: standards of medical care in diabetes – 2020. *Diabetes Care*. 2020;43(suppl 1):S66-S76. https://doi.org/10.2337/dc20-S006

Aminian A, Zajichek A, Arterburn DE, et al. Association of metabolic surgery with major adverse cardiovascular outcomes in patients with type 2 diabetes and obesity. *JAMA*. 2019;322(13):1271-1282. https://doi.org/10.1001/jama.2019.14231

Aroda VR, Ratner R. The safety and tolerability of GLP-1 receptor agonists in the treatment of type 2 diabetes: a review. *Diabetes Metab Res Rev*. 2011;27(6):528-542. https://doi.org/10.1002/dmrr.1202

Banks PA, Freeman ML.; Practice Parameters Committee of the American College of Gastroenterology. Practice guidelines in acute pancreatitis. *Am J Gastroenterol*. 2006;101(10):2379-2400. https://doi.org/10.1111/j.1572-0241.2006.00856.x

Danne T, Philotheou A, Goldman D, et al. A randomized trial comparing the rate of hypoglycemia – assessed using continuous glucose monitoring – in 125 preschool children with type 1 diabetes treated with insulin glargine or NPH insulin (the PRESCHOOL study). *Pediatr Diabetes*. 2013;14(8):593-601. https://doi.org/10.1111/pedi.12051

Davies MJ, Bergenstal R, Bode B, et al. Efficacy of liraglutide for weight loss among patients with type 2 diabetes: The SCALE Diabetes Randomized Clinical Trial. *JAMA*. 2015;314(7):687-699. https://doi.org/10.1001/jama.2015.9676 Erratum in: JAMA. 2016 Jan 5;315(1):90. PMID: 26284720.

Davies MJ, D'Alessio DA, Fradkin J, et al. Management of hyperglycemia in type 2 diabetes, 2018. A consensus report by the American Diabetes Association (ADA) and the European Association for the Study of Diabetes (EASD). *Diabetes Care*. 2018;41(12):2669-2701. https://doi.org/10.2337/dci18-0033

[EMA] European Medicines Agency. Guideline on clinical evaluation of medicinal products used in weight management. EMA/CHMP/311805/2014. Published June 23, 2016. Accessed December 23, 2020. https://www.ema.europa.eu/en/documents/scientific-guideline/guideline-clinical-evaluation-medicinal-products-used-weight-management-revision-1_en.pdf

EuroQol Research Foundation. EQ-5D-5L User Guide, Version 3.0. Updated September 2019. Accessed March 13, 2020. https://euroqol.org/publications/user-guides

[FDA] United States Food and Drug Administration. Guidance for industry: developing products for weight management. Published February 2007. Accessed December 23, 2020. https://www.fda.gov/media/71252/download

Ferreira JP, Jhund PS, Duarte K, et al. Use of the win ratio in cardiovascular trials. *JACC Heart Fail*. 2020;8(6):441-450. https://doi.org/10.1016/j.jchf.2020.02.010

Finkelstein DM, Schoenfeld DA. Combining mortality and longitudinal measures in clinical trials. *Stat Med*. 1999;18(11):1341-1354. https://doi.org/10.1002/(sici)1097-0258(19990615)18:11<1341::aid-sim129>3.0.co;2-7

Frias JP, Nauck MA, Van J, et al. Efficacy and safety of LY3298176, a novel dual GIP and GLP-1 receptor agonist, in patients with type 2 diabetes: a randomised, placebo-controlled and active comparator-controlled phase 2 trial. *Lancet*. 2018;392(10160):2180-2193. https://doi.org/10.1016/s0140-6736(18)32260-8

Green CP, Porter CB, Bresnahan DR, Spertus JA. Development and evaluation of the Kansas City Cardiomyopathy Questionnaire: a new health status measure for heart failure. *J Am Coll Cardiol*. 2000;35(5):1245-1255. https://doi.org/10.1016/S0735-1097(00)00531-3

Herring LY, Stevinson C, Davies MJ, et al. Changes in physical activity behaviour and physical function after bariatric surgery: a systematic review and meta-analysis. *Obes Rev*. 2016;17(3):250-261. https://doi.org/10.1111/obr.12361

Jamaly S, Carlsson L, Peltonen M, et al. Surgical obesity treatment and the risk of heart failure. *Eur Heart J*. 2019;40(26):2131-2138. https://doi.org/10.1093/eurheartj/ehz295

Joseph SM, Novak E, Arnold SV, et al. Comparable performance of the Kansas City Cardiomyopathy Questionnaire in patients with heart failure with preserved and reduced ejection fraction. *Circ Heart Fail*. 2013;6(6):1139-1146. https://doi.org/10.1161/CIRCHEARTFAILURE.113.000359

Kitzman DW, Brubaker P, Morgan T, et al. Effect of caloric restriction or aerobic exercise training on peak oxygen consumption and quality of life in obese older patients with heart failure with preserved ejection fraction: a randomized clinical trial. *JAMA*. 2016;315(1):36-46. https://doi.org/10.1001/jama.2015.17346

Kitzman DW, Shah SJ. The HFpEF obesity phenotype: the elephant in the room. *J Am Coll Cardiol*. 2016;68(2):200-203. https://doi.org/10.1016/j.jacc.2016.05.019

Koizumi M, Takada T, Kawarada Y, et al. JPN Guidelines for the management of acute pancreatitis: diagnostic criteria for acute pancreatitis. *J Hepatobiliary Pancreat Surg*. 2006;13(1):25-32. https://doi.org/10.1007/s00534-005-1048-2

Lingvay I, Desouza CV, Lalic KS, et al. A 26-week randomized controlled trial of semaglutide once daily versus liraglutide and placebo in patients with type 2 diabetes suboptimally controlled on diet and exercise with or without metformin. *Diabetes Care*. 2018;41(9):1926-1937. https://doi.org/10.2337/dc17-2381

Mikhalkova D, Holman SR, Jiang H, et al. Bariatric surgery-induced cardiac and lipidomic changes in obesity-related heart failure with preserved ejection fraction. *Obesity (Silver Spring)*. 2018;26(2):284-290. https://doi.org/10.1002/oby.22038

Miller WL, Borlaug BA. Impact of obesity on volume status in patients with ambulatory chronic heart failure. *J Card Fail*. 2020;26(2):112-117. https://doi.org/10.1016/j.cardfail.2019.09.010

Nauck MA, Frossard JL, Barkin JS, et al. Assessment of pancreas safety in the development program of once-weekly GLP-1 receptor agonist dulaglutide. *Diabetes Care*. 2017;40(5):647-654. https://doi.org/10.2337/dc16-0984

Packer M. Epicardial adipose tissue may mediate deleterious effects of obesity and inflammation on the myocardium. *J Am Coll Cardiol*. 2018;71(20):2360-2372. https://doi.org/10.1016/j.jacc.2018.03.509

Pi-Sunyer X, Astrup A, Fujioka K, et al; SCALE Obesity and Prediabetes NN8022-1839 Study Group. A randomized, controlled trial of 3.0 mg of liraglutide in weight management. *N Engl J Med*. 2015;373(1):11-22. https://doi.org/10.1056/NEJMoa1411892

Pocock SJ, Ariti CA, Collier TJ, Wang D. The win ratio: a new approach to the analysis of composite endpoints in clinical trials based on clinical priorities. *Eur Heart J*. 2012;33(2):176-182. https://doi.org/10.1093/eurheartj/ehr352

Rayner JJ, Peterzan MA, Watson WD, et al. Myocardial energetics in obesity: enhanced ATP delivery through creatine kinase with blunted stress response. *Circulation*. 2020;141(14):1152-1163. https://doi.org/10.1161/CIRCULATIONAHA.119.042770

Reddy YN, Rikhi A, Obokata M, et al. Quality of life in heart failure with preserved ejection fraction: importance of obesity, functional capacity, and physical inactivity. *Eur J Heart Fail*. 2020;22(6):1009-1018. https://doi.org/10.1002/ejhf.1788

Regev A, Palmer M, Avigan MI, et al. Consensus: guidelines: best practices for detection, assessment and management of suspected acute drug-induced liver injury during clinical trials in patients with nonalcoholic steatohepatitis. *Aliment Pharmacol Ther*. 2019;49(6):702-713.

Rubin DB. *Multiple Imputation for Nonresponse in Surveys*. John Wiley & Sons, Inc.; 1987.

Shimada YJ, Tsugawa Y, Brown DFM, Hasegawa K. Bariatric surgery and emergency department visits and hospitalizations for heart failure exacerbation: population-based, self-controlled series. *J Am Coll Cardiol*. 2016;67(8):895-903. https://doi.org/10.1016/j.jacc.2015.12.016

Shoemaker MJ, Curtis AB, Vangsnes E, Dickinson MG. Clinically meaningful change estimates for the six-minute walk test and daily activity in individuals with chronic heart failure. *Cardiopulm Phys Ther J*. 2013;24(3):21-29.

Solomon SD, Dobson J, Pocock S, et al.; Candesartan in Heart Failure: Assessment of Reduction in Mortality and Morbidity (CHARM) Investigators. *Circulation*. 2007;116(13):1482-1487. https://doi.org/10.1161/CIRCULATIONAHA.107.696906

Spertus JA, Jones PG, Sandhu AT, Arnold SV. Interpreting the Kansas City Cardiomyopathy Questionnaire in clinical trials and clinical care: JACC state-of-the-art review. *J Am Coll Cardiol*. 2020;76(20):2379-2390. https://doi.org/10.1016/j.jacc.2020.09.542

Steinberg WM, Buse JB, Ghorbani MLM, et al.; LEADER Steering Committee; LEADER Trial Investigators. Amylase, lipase, and acute pancreatitis in people with type 2 diabetes treated with liraglutide: results from the LEADER randomized trial. *Diabetes Care*. 2017a;40(7):966-972. https://doi.org/10.2337/dc16-2747

Steinberg WM, Rosenstock J, Wadden TA, et al. Impact of liraglutide on amylase, lipase, and acute pancreatitis in participants with overweight/obesity and normoglycemia, prediabetes, or type 2 diabetes: secondary analyses of pooled data from the SCALE clinical development program. *Diabetes Care*. 2017b;40(7):839-848. https://doi.org/10.2337/dc16-2684

Sundström J, Bruze G, Ottosson J, et al. Weight loss and heart failure: a nationwide study of gastric bypass surgery versus intensive lifestyle treatment. *Circulation*. 2017;135(17):1577-1585. https://doi.org/10.1161/CIRCULATIONAHA.116.025629

Täger T, Hanholz W, Cebola R, et al. Minimal important difference for 6-minute walk test distances among patients with chronic heart failure. *Int J Cardiol*. 2014;176(1):94-98. https://doi.org/10.1016/j.ijcard.2014.06.035

van der Meer P, Gaggin HK, Dec GW. ACC/AHA versus ESC guidelines on heart failure: JACC guideline comparison. *J Am Coll Cardiol*. 2019;73(21):2756-2768. https://doi.org/10.1016/j.jacc.2019.03.478

Weinberg ME, Bacchetti P, Rushakoff RJ. Frequently repeated glucose measurements overestimate the incidence of inpatient hypoglycemia and severe hyperglycemia. *J Diabetes Sci Technol.* 2010;4(3):577-582. https://doi.org/10.1177/193229681000400311

Wilson JM, Nikooienejad A, Robins DA, et al. The dual glucose-dependent insulinotropic peptide and glucagon-like peptide-1 receptor agonist, tirzepatide, improves lipoprotein biomarkers associated with insulin resistance and cardiovascular risk in patients with type 2 diabetes [published online August 16, 2020]. *Diab Obes and Metab*. https://doi.org/10.1111/dom.14174

Leo Document ID = 9c8244e5-2b39-4e27-8d76-a825193568a2

Approver: PPD

Approval Date & Time: 13-Jan-2021 16:30:02 GMT

Signature meaning: Approved

Approver: PPD

Approval Date & Time: 13-Jan-2021 16:41:02 GMT

Signature meaning: Approved

Title Page

Confidential Information

The information contained in this document is confidential and is intended for the use of clinical investigators. It is the property of Eli Lilly and Company or its subsidiaries and should not be copied by or distributed to persons not involved in the clinical investigation of tirzepatide (LY3298176), unless such persons are bound by a confidentiality agreement with Eli Lilly and Company or its subsidiaries.

Note to Regulatory Authorities: This document may contain protected personal data and/or commercially confidential information exempt from public disclosure. Eli Lilly and Company requests consultation regarding release/redaction prior to any public release. In the United States, this document is subject to Freedom of Information Act (FOIA) Exemption 4 and may not be reproduced or otherwise disseminated without the written approval of Eli Lilly and Company or its subsidiaries.

Protocol Title: A Randomized, Double-Blind, Placebo-Controlled, Phase 3 Study Comparing the Efficacy and Safety of Tirzepatide versus Placebo in Patients with Heart Failure with Preserved Ejection Fraction and Obesity (SUMMIT)

Protocol Number: I8F-MC-GPID

Amendment Number: a

Compound: LY3298176

Study Phase: 3

Short Title: Tirzepatide vs Placebo in Obesity-related HFpEF

Sponsor Name: Eli Lilly and Company

Legal Registered Address: Indianapolis, Indiana USA 46285

Manufacturer: Eli Lilly and Company

Regulatory Agency Identifier Number(s)

IND: 147352

Approval Date: Protocol amendment (a) Electronically Signed and Approved by Lilly on date provided below.

Approval Date: 15-Dec-2021 GMT

Medical Monitor Name and Contact Information will be provided separately.

Protocol Amendment Summary of Changes Table

DOCUMENT HISTORY	
**Document**	**Date**
Original Protocol	*13 January 2021*

Amendment a

Overall Rationale for the Amendment:

The purpose of this protocol amendment is to clarify dosing information to allow for more participant retention and clarity on the dosing regimen, add additional unscheduled visits, and make inclusion/exclusion criteria updates.

Section # and Name	Description of Change	Brief Rationale
Section 1.1 Synopsis	• In Intervention Groups and Duration, changed 15 mg to QW<br>• Added wording to Study Period 1 to specify timing	• To Clarify dose<br>• For Clarification in timing
Section 1.2 Schema	Adjusted wording in Note for screening procedures timing	To Clarify screening period timing
Section 1.3 Schedule of Activities (SoA)	• Added 3 columns to Unscheduled Visits (UV): UV, Dosing UV, and Phone follow up Dosing UV, added Xs for specific tests<br>• Footnote "a" added in regard to Unscheduled Visits; subsequent footnotes adjusted<br>• For Telephone Visit, X added to Phone Follow-Up Dosing UV	• To specify UV<br>• To explain UV; for accuracy<br>• For Accuracy
Section 3 Objectives and Endpoints	• Added Other Secondary title & moved Incidence HF events and CV Death from Exploratory<br>• Added timing to first occurrence and recurrent events for HF events and CV death<br>• The Endpoint wording for the exploratory Objective Atrial fibrillation is updated	• Change in Endpoints<br>• To specify timings<br>• Correction
Section 4.1 Overall Design	• For Study Period 1, timing period adjusted	• Correction

	• For Screening, sentence added to clarify time period for V1<br><br>• For Treatment, dose escalation described; paragraphs added to specify that participant be maintained on study drug; some sentences regarding dose and discontinuation removed	• Clarification for timing for duration of V1<br><br>• New information added to clarify dose escalation, maintenance on study drug; To reduce redundancy
Section 5.1 Inclusion Criteria	• For Inclusion Criterion #7 added additional HF medications	• Clarification for Concomitant Medication use
Section 5.2 Exclusion Criteria	• For Exclusion Criterion #13, NYHA Class information added<br>• For Exclusion Criterion #15 changed wording to include MRI or other local modalities<br>• For Exclusion Criterion #21 added sentence on participants with Chagas disease<br>• For Exclusion Criterion #25 removed sentence regarding fundoscopic examination and clarified definition of nonproliferative diabetic retinopathy	• Additional information<br>• For Clarification<br><br>• For Clarification<br><br>• Correction and Clarification
Section 5.4 Screen Failures	Added upper limit of screening period to clarify screen failure	For Clarification
Section 6.4 Study Intervention Compliance	Wording added to clarify participant compliance	For Clarification
Section 6.5 Concomitant Therapy	• Added information on ARNI, SGLT2i, and GLP-1/GIPR use in the study<br>• Changed Hyperglycemia Rescue at Week 26 to Week 24<br>• Wording added in Standard of Care for Heart Failure	• Clarification of Concomitant Medication Use<br>• Correction<br><br>• For Clarification

	• Wording changed to Clarify dosing modification for GI Symptoms	• For Clarification
Section 6.6 Dose Modification	Information changed to clarify dose modification	For Clarification
Section 6.7 Intervention after the End of the Study	Wording added in regard to unblinding information	For Clarification
Section 7.1.1 Permanent Discontinuation from Study Drug	• Wording added to clarify permanent discontinuation of study drug<br>• Wording changed to specific criteria when participant has Pancreatic Cancer	• For Clarification<br>• For Clarification
Section 7.1.2 Temporary Interruption	Wording changed to clarify that study interruption, is not study discontinuation; Moved to Section 6.6.1	For Clarification
Section 7.1.3 Restarting Study Drug after Interruption	Added wording on Maintenance dose levels; Moved to Section 6.6.2	For Clarification
Section 7.2 Participant Discontinuation/Withdrawal from the Study	Wording changed to clarify participant retention in the study	For Clarification
Section 7.2.1 Inadvertently Enrolled Participants	Wording Changed to clarify medical appropriateness of treatment	For Clarification
Section 8 Study Assessments and Procedures	Information added regarding remote visits	For Clarification
Section 8.1.1.3 Six-Minute Walk Test	Clarified wording on Borg Scale	For Clarification
Section 8.2.3 Electrocardiograms	Clarified on use of ECG machine type	For Clarification
Section 8.2.4 Clinical Safety Laboratory Assessments	Urine pregnancy wording added	For Clarification

Section 10.1.1 Regulatory and Ethical Considerations	Added bullet point, International Organization for Standardization (ISO) 14155 and wording on substantiality of amendments	To align with current Harmonized Protocol Template v10
Section 10.1.11 Investigator Information	Added Investigator Information Section	To align with current Harmonized Protocol Template v10
Section 10.2 Appendix 2: Clinical Laboratory Tests	Removed Lactate dehydrogenase	Error Correction
Section 10.5.1.5 Major Adverse Cardiovascular Events	Removed hospitalization for HF	This is an efficacy point
Section 10.10 Appendix 10: Six Minute Walk Test Screening Procedures	Flow Diagram corrected for 6MWD	Error Correction

Table of Contents

1. Protocol Summary 10
1.1. Synopsis 10
1.2. Schema 13
1.3. Schedule of Activities (SoA) 14

2. Introduction 31
2.1. Study Rationale 31
2.2. Background 31
2.3. Benefit/Risk Assessment 32

3. Objectives and Endpoints 33

4. Study Design 36
4.1. Overall Design 36
4.2. Scientific Rationale for Study Design 38
4.2.1. Patient Input into Design 39
4.3. Justification for Dose 39
4.4. End of Study Definition 39

5. Study Population 41
5.1. Inclusion Criteria 41
5.2. Exclusion Criteria 43
5.3. Lifestyle Considerations 45
5.4. Screen Failures 45

6. Study Intervention 47
6.1. Study Interventions Administered 47
6.1.1. Medical Devices 48
6.2. Preparation/Handling/Storage/Accountability 48
6.3. Measures to Minimize Bias: Randomization and Blinding 49
6.4. Study Intervention Compliance 49
6.5. Concomitant Therapy 50
6.6. Dose Modification 52
6.6.1. Temporary Interruption 53
6.6.2. Restarting Study Drug after Interruption 54
6.7. Intervention after the End of the Study 55

7. Discontinuation of Study Intervention and Participant Discontinuation/Withdrawal 56
7.1. Discontinuation of Study Intervention 56
7.1.1. Permanent Discontinuation from Study Drug 56
7.2. Participant Discontinuation/Withdrawal from the Study 57
7.2.1. Inadvertently Enrolled Participants 59
7.3. Lost to Follow up 59

8. Study Assessments and Procedures 60
8.1. Efficacy Assessments 60
8.1.1. Primary Efficacy Assessment 60

8.1.2. Additional Secondary Efficacy Assessments 61
8.1.3. Exploratory Efficacy Assessments 62
8.2. Safety Assessments 63
8.2.1. Physical Examinations 63
8.2.2. Vital Signs 63
8.2.3. Electrocardiograms 63
8.2.4. Clinical Safety Laboratory Assessments 64
8.2.5. Safety Monitoring 64
8.3. Adverse Events, Serious Adverse Events, and Product Complaints 67
8.3.1. Timing and Mechanism for Collecting Events 68
8.3.2. Primary, Secondary, and Additional Study Endpoint Reporting 70
8.3.3. Adverse Events of Special Interest 70
8.4. Treatment of Overdose 71
8.5. Pharmacokinetics 71
8.6. Pharmacodynamics 71
8.7. Genetics 72
8.8. Biomarkers 72
8.9. Immunogenicity Assessments 72
8.10. Medical Resource Utilization and Health Economics 73

9. Statistical Considerations 74
9.1. Statistical Hypotheses 74
9.2. Sample Size Determination 74
9.3. Populations for Analyses 75
9.4. Statistical Analyses 75
9.4.1. General Considerations 75
9.4.2. Primary Endpoint(s) 76
9.4.3. Key Secondary Endpoint(s) 77
9.4.4. Tertiary/Exploratory Endpoint(s) 78
9.4.5. Other Safety Analyses 78
9.4.6. Subgroup Analyses 78
9.5. Interim Analyses 78
9.6. Data Monitoring Committee 79

10. Supporting Documentation and Operational Considerations 80
10.1. Appendix 1: Regulatory, Ethical, and Study Oversight Considerations 80
10.1.1. Regulatory and Ethical Considerations 80
10.1.2. Financial Disclosure 80
10.1.3. Informed Consent Process 81
10.1.4. Data Protection 81
10.1.5. Dissemination of Clinical Study Data 81
10.1.6. Data Quality Assurance 82
10.1.7. Source Documents 83
10.1.8. Study and Site Start and Closure 84
10.1.9. Publication Policy 84
10.1.10. Long-Term Sample Retention 84

10.1.11. Investigator Information 85
10.2. Appendix 2: Clinical Laboratory Tests....... 85
10.3. Appendix 3: Adverse Events: Definitions and Procedures for Recording, Evaluating, Follow-up, and Reporting....... 88
10.3.1. Definition of AE 88
10.3.2. Definition of SAE....... 89
10.3.3. Definition of Product Complaints 91
10.3.4. Recording and Follow-Up of AE and/or SAE and Product Complaints 91
10.3.5. Reporting of SAEs....... 93
10.3.6. Regulatory Reporting Requirements 93
10.4. Appendix 4: Contraceptive Guidance and Collection of Pregnancy Information 94
10.4.1. Male participants: 94
10.4.2. Female participants:....... 94
10.5. Appendix 5: Adverse Events of Special Interest: Definitions and Procedures for Recording, Evaluating, Follow-Up, and Reporting....... 98
10.5.1. Special Safety Topics 98
10.6. Appendix 6: Genetics 103
10.7. Appendix 7: Recommended Laboratory Testing for Hypersensitivity Events....... 104
10.8. Appendix 8: Liver Safety: Suggested Actions and Follow-Up Assessments 106
10.9. Appendix 9: Medical Device Adverse Events (AEs), Adverse Device Effects (ADEs), Serious Adverse Events (SAEs) and Device Deficiencies: Definition and Procedures for Recording, Evaluating, Follow-Up, and Reporting....... 108
10.10. Appendix 10: Six-Minute Walk Test Screening Procedures 109
10.10.1. Screening Procedures and Flow Diagram....... 109
10.11. Appendix 11: New York Heart Association Classification of Heart Failure....... 110
10.12. Appendix 12: Abbreviations 111
11. References 116

1. Protocol Summary

1.1. Synopsis

Protocol Title: A Randomized, Double-Blind, Placebo-Controlled, Phase 3 Study Comparing the Efficacy and Safety of Tirzepatide versus Placebo in Patients with Heart Failure with Preserved Ejection Fraction and Obesity (SUMMIT)

Short Title: Tirzepatide vs Placebo in Obesity-related HFpEF

Rationale:

Heart failure with preserved ejection fraction is a heterogenous clinical syndrome resulting from various pathophysiological processes. Among the broad spectrum of HFpEF clinical presentation, obesity-related HFpEF displays a distinct phenotype where increased visceral and ectopic adiposity as well as volume expansion plays a causal role (Kitzman and Shah 2016; Packer 2018; Miller and Borlaug 2020). Given tirzepatide's anti-inflammatory and antifibrotic effects and a reduction in circulating plasma volume as a consequence of the treatment of obesity, tirzepatide may provide clinical benefit to patients with HFpEF and BMI ≥30 kg/m^2.

Study I8F-MC-GPID, also known as SUMMIT, is a Phase 3, randomized, multicenter, international, placebo-controlled, double-blinded, parallel-arm study. This study will evaluate the effect of SC QW injection of tirzepatide, MTD up to 15 mg, on the risk of death, hospitalization or emergency care due to HF, exercise capacity, and health status in participants with HFpEF and BMI ≥30 kg/m^2.

Objectives and Endpoints

Objectives	Endpoints
Primary	
To demonstrate that a maximally tolerated tirzepatide dose up to 15 mg administered subcutaneously once weekly (SC QW) is superior to placebo based on the hierarchal composite endpoint in participants with HFpEF and BMI ≥30 kg/m^2.	A hierarchical composite of the following:<br>1. Time to all-cause mortality through the end of the treatment period<br>2. Occurrence of heart failure (HF) events through end of the treatment period, where HF events include HF hospitalization OR urgent HF visit (adjudicated)<br>o number of HF events<br>o time to first HF events<br>3. Change from baseline in the 6-minute walk test distance (6MWD) category at Week 52

	4. Change from baseline in the Kansas City Cardiomyopathy Questionnaire (KCCQ) Clinical Summary Scale (CSS) category at Week 52<br><br>The categories for change from baseline in the 6MWD are: 1) ≥30% worsening; 2) ≥20% and <30% worsening; 3) ≥10% and <20% worsening; 4) No change (<10% change); 5) ≥10% and <20% improvement; 6) ≥20% and <30% improvement; and 7) ≥30% improvement.<br><br>The categories for change from baseline in the KCCQ-CSS are: 1) ≥10-point worsening; 2) ≥5- but <10-point worsening; 3) No change (<5-point change); 4) ≥5- but <10-point improvement; and 5) ≥10-point improvement.
To demonstrate that a maximally tolerated tirzepatide dose up to 15 mg administered SC QW is superior to placebo to improve exercise capacity in participants with HFpEF and BMI ≥30 kg/m^2.	Change from baseline to Week 52 in exercise capacity as measured 6MWD
Key Secondary (multiplicity controlled)	
Long-term weight loss	Percent change from baseline to Week 52 in body weight loss
Patient-reported symptoms and physical limitations	Change from baseline to Week 52 in the KCCQ CSS
Exercise capacity	Change from baseline to Week 24 in 6MWD
New York Heart Association (NYHA) Class	Proportion of participants with NYHA Class change at Week 52

Abbreviations: BMI = body mass index; HFpEF = heart failure with preserved ejection fraction.

Overall Design

Study GPID is a randomized, outpatient, multicenter, international, placebo-controlled, double-blind, parallel-arm, Phase 3 study with 2 study periods. The study is designed to evaluate the efficacy and safety of SC QW tirzepatide, MTD up to 15 mg, compared to placebo, in participants with HFpEF and obesity.

Two intervention groups will be studied:

- Tirzepatide, MTD up to 15 mg, SC QW
- Placebo

The starting dose of tirzepatide is 2.5 mg QW, which is to be escalated at 4-week intervals to a maximum of 15 mg QW or to the highest maintenance dose tolerated by the participant (see Section 6).

The maximum duration of participation depends on when the last participant completes 52 weeks of treatment.

Disclosure Statement: This is a parallel-treatment study with 2 intervention groups that is double blinded.

Number of Participants:

Approximately 700 participants will be randomly assigned to study drug with approximately 350 participants per intervention group.

Intervention Groups and Duration:

The study will compare treatment with tirzepatide and treatment with placebo. Assignment to tirzepatide or placebo groups will be randomly allocated in a 1:1 ratio.

The starting dose of tirzepatide 2.5 mg QW is to be escalated to 15 mg QW or the highest maintenance dose tolerated by the participant (5 mg, 10 mg, QW).

Study participation for each participant is sectioned into the following study periods:

- Study Period 1: screening period, approximately 6 weeks and not more than 12 weeks
- Study Period 2: treatment period, at least 52 weeks

The study will continue until approximately 52 weeks after the last participant is randomized. The maximum duration of an individual's participation is estimated to be 120 weeks and will depend on duration of study enrollment.

Data Monitoring Committee: Yes

1.2. Schema

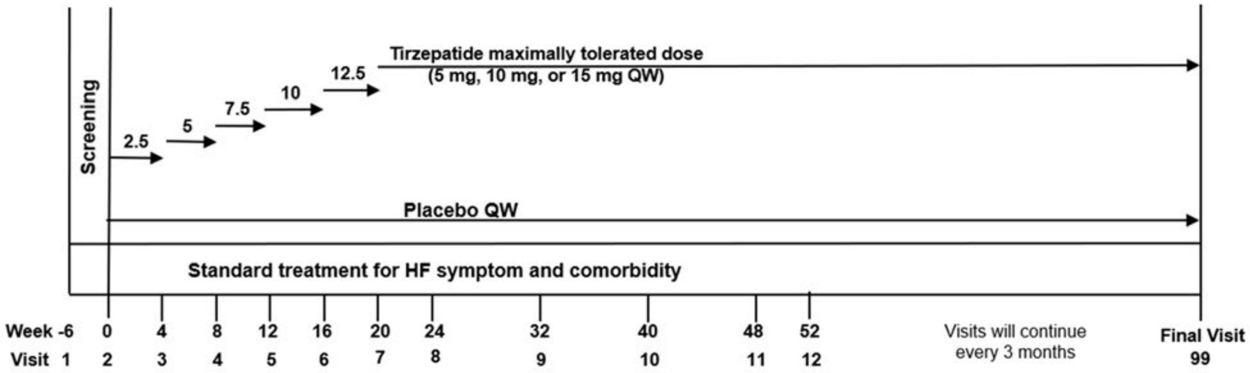

Abbreviations: HF = heart failure; QW = weekly.

Note: Screening procedures may take longer or shorter than 6 weeks but no more than 12 weeks.

1.3. Schedule of Activities (SoA)

Visit 1 and 2 procedures may be conducted over more than 1 day each as long as all activities are completed within the allowable visit interval tolerance for each visit.

For early terminations (ET) from the study that occur before the final visit (V99) in treatment period, see the activities listed for ET in this table.

Study I8F-MC-GPID	Study Period I Screening	Study Period II - Treatment period																			
														Unscheduled Visit (UV)[a]							
Visit number	1	2	3	4	5	6	7	8	9	10	11	12	EVa, EVb, EVc (13, 14, 15, 17, 18, 19, etc.)	EVd (16, 20, etc.)	UV	Dosing UV	Phone Follow-Up Dosing UV	ET	99		Comment
Weeks from randomization	-6	0	4	8	12	16	20	24	32	40	48	52	(+3, 6, 9, 15, 18, 21 months)	(+12, 24 months)				—	Final Visit		See footnote b
Visit interval tolerance (days)			±3	±3	±3	±3	±3	±3	±7	±7	±7	±7	±15	±15				—			
Telephone Visit									X		X		X				X				See footnote c
Informed consent	X																				
Inclusion and exclusion criteria, review and confirm	X	X																			
Demographics	X																				
Preexisting conditions and medical history, including relevant surgical history	X																				

Study I8F-MC-GPID	Study Period I Screening	Study Period II - Treatment period																		
															Unscheduled Visit (UV)[a]					
Visit number	1	2	3	4	5	6	7	8	9	10	11	12	EVa, EVb, EVc (13, 14, 15, 17, 18, 19, etc.)	EVd (16, 20, etc.)	UV	Dosing UV	Phone Follow-Up Dosing UV	ET	99	Comment
Weeks from randomization	-6	0	4	8	12	16	20	24	32	40	48	52	(+3, 6, 9, 15, 18, 21 months)	(+12, 24 months)				—	Final Visit	See footnote b
Visit interval tolerance (days)			±3	±3	±3	±3	±3	±3	±7	±7	±7	±7	±15	±15				—		
Telephone Visit									X		X		X				X			See footnote c
Prespecified medical history (indication and history of interest)	X																			Includes HF history, hospitalization for HF, CVD, MI, atrial fibrillation, stroke, CV risk (T2DM, HTN, dyslipidemia, metabolic syndrome)
Prior treatments for HFpEF	X																			
Substance use (alcohol, tobacco use)	X																			
Concomitant medications	X	X	X	X	X	X	X	X	X	X	X	X	X	X	X	X	X	X	X	
Adverse events (AEs)	X	X	X	X	X	X	X	X	X	X	X	X	X	X	X	X	X	X	X	Including Product Complaints
**Physical Evaluation**																				
Height	X																			

Study I8F-MC-GPID	Study Period I Screening	Study Period II - Treatment period																		
														Unscheduled Visit (UV)[a]						
Visit number	1	2	3	4	5	6	7	8	9	10	11	12	EVa, EVb, EVc (13, 14, 15, 17, 18, 19, etc.)	EVd (16, 20, etc.)	UV	Dosing UV	Phone Follow-Up Dosing UV	ET	99	Comment
Weeks from randomization	-6	0	4	8	12	16	20	24	32	40	48	52	(+3, 6, 9, 15, 18, 21 months)	(+12, 24 months)				—	Final Visit	See footnote b
Visit interval tolerance (days)			±3	±3	±3	±3	±3	±3	±7	±7	±7	±7	±15	±15				—		
Telephone Visit									X		X		X				X			See footnote c
Weight	X	X	X	X	X	X	X	X		X		X		X	X	X		X	X	
Waist circumference	X	X						X				X		X				X	X	
Vital Signs	X	X	X	X	X	X	X	X		X		X		X	X	X		X	X	Include 2 Sitting BP and HR. HR to be performed by apical auscultation. Vital signs should be collected prior to the first 6MWT of the day and before ECG.
Physical examination	X											X		X				X		
Symptom-directed physical examination		X	X	X	X	X	X	X		X		X		X	X	X			X	As indicated based on participant status and standard of care, including dyspnea, orthopnea, paroxysmal nocturnal dyspnea (PND), edema, jugular venous distension (JVD), rales

Study I8F-MC-GPID	Study Period I Screening	Study Period II - Treatment period																		
														Unscheduled Visit (UV)[a]						
Visit number	1	2	3	4	5	6	7	8	9	10	11	12	EVa, EVb, EVc (13, 14, 15, 17, 18, 19, etc.)	EVd (16, 20, etc.)	UV	Dosing UV	Phone Follow-Up Dosing UV	ET	99	Comment
Weeks from randomization	-6	0	4	8	12	16	20	24	32	40	48	52	(+3, 6, 9, 15, 18, 21 months)	(+12, 24 months)				—	Final Visit	See footnote b
Visit interval tolerance (days)			±3	±3	±3	±3	±3	±3	±7	±7	±7	±7	±15	±15				—		
Telephone Visit									X		X		X				X			See footnote c
NYHA class assessment	X	X						X				X		X				X[d]		NYHA class assessment must be performed by an independent assessor. [d]Perform at ET only if participant early terminates at or prior to Week 52.
HF events		X	X	X	X	X	X	X	X	X	X	X	X	X	X	X		X	X	
Evaluation of injection site reactions		X	X	X	X	X	X	X		X		X		X	X	X		X	X	
Single-read 12-lead ECG	X	X						X				X						X[d]		Collect locally. Report atrial fibrillation or other abnormalities on the eCRF. Optional ECG is allowable if indicated. [d]Perform at ET only if participant early terminates at or prior to Week 52.

Study I8F-MC-GPID	Study Period I Screening	Study Period II - Treatment period																	
														Unscheduled Visit (UV)[a]					
Visit number	1	2	3	4	5	6	7	8	9	10	11	12	EVa, EVb, EVc (13, 14, 15, 17, 18, 19, etc.)	EVd (16, 20, etc.)	UV	Dosing UV	Phone Follow-Up Dosing UV	ET	99	Comment
Weeks from randomization	-6	0	4	8	12	16	20	24	32	40	48	52	(+3, 6, 9, 15, 18, 21 months)	(+12, 24 months)				—	Final Visit	See footnote b
Visit interval tolerance (days)			±3	±3	±3	±3	±3	±3	±7	±7	±7	±7	±15	±15				—		
Telephone Visit									X		X		X				X			See footnote c
Echocardiography	X																			For those required to complete the ECHO examination
Dilated fundoscopic examination	X																			Perform for participants with T2DM who have not had an evaluable dilated fundoscopic examination in the last 12 months. See exclusion criterion 25 (Section 5.2)<br>Follow-up dilated fundoscopic examination should be performed when clinically indicated by any AE suspected of worsening retinopathy.

Study I8F-MC-GPID	Study Period I Screening	Study Period II - Treatment period																		
														Unscheduled Visit (UV)[a]						
Visit number	1	2	3	4	5	6	7	8	9	10	11	12	EVa, EVb, EVc (13, 14, 15, 17, 18, 19, etc.)	EVd (16, 20, etc.)	UV	Dosing UV	Phone Follow-Up Dosing UV	ET	99	Comment
Weeks from randomization	-6	0	4	8	12	16	20	24	32	40	48	52	(+3, 6, 9, 15, 18, 21 months)	(+12, 24 months)				—	Final Visit	See footnote b
Visit interval tolerance (days)			±3	±3	±3	±3	±3	±3	±7	±7	±7	±7	±15	±15				—		
Telephone Visit									X		X		X				X			See footnote c
6MWT	X[e]	X[f]						X				X						X[d]		Ensure that participant completes the associated Borg Questionnaire prior to and after the 6MWT. [e]Two 6MWTs conducted at screening visit. [f]See Section 10.10.1 to determine if the 6MWT needs to be repeated for Visit 2. [d]Perform at ET only if participant early terminates at or prior to Week 52.
**Participant Education**																				
Diary instruction		X																		
**Participant Diary**																				

Study I8F-MC-GPID	Study Period I Screening	Study Period II - Treatment period																		
														Unscheduled Visit (UV)[a]						
Visit number	1	2	3	4	5	6	7	8	9	10	11	12	EVa, EVb, EVc (13, 14, 15, 17, 18, 19, etc.)	EVd (16, 20, etc.)	UV	Dosing UV	Phone Follow-Up Dosing UV	ET	99	Comment
Weeks from randomization	-6	0	4	8	12	16	20	24	32	40	48	52	(+3, 6, 9, 15, 18, 21 months)	(+12, 24 months)				—	Final Visit	See footnote b
Visit interval tolerance (days)			±3	±3	±3	±3	±3	±3	±7	±7	±7	±7	±15	±15				—		
Telephone Visit									X		X		X				X			See footnote c
Participant diary dispensed		X	X	X	X	X	X	X		X		X		X						
Diary compliance check/Assess study drug compliance[g]			X	X	X	X	X	X	X	X	X	X	X	X				X	X	
Diary return			X	X	X	X	X	X		X		X		X				X	X	
**Patient-Reported Outcomes (PROs) (Electronic[h]**																				[h]Perform PRO at ET only if participant early terminates at or prior to Week 52.
Kansas City Cardiomyopathy Questionnaire (KCCQ)	X	X						X				X						X		
EQ-5D-5L		X						X				X						X		

Study I8F-MC-GPID	Study Period I Screening	Study Period II - Treatment period																		
														Unscheduled Visit (UV)[a]						
Visit number	1	2	3	4	5	6	7	8	9	10	11	12	EVa, EVb, EVc (13, 14, 15, 17, 18, 19, etc.)	EVd (16, 20, etc.)	UV	Dosing UV	Phone Follow-Up Dosing UV	ET	99	Comment
Weeks from randomization	-6	0	4	8	12	16	20	24	32	40	48	52	(+3, 6, 9, 15, 18, 21 months)	(+12, 24 months)				—	Final Visit	See footnote b
Visit interval tolerance (days)			±3	±3	±3	±3	±3	±3	±7	±7	±7	±7	±15	±15				—		
Telephone Visit									X		X		X				X			See footnote c
Patient Global Impression of Status – Overall (PGIS-Overall)	X	X						X				X						X		
Patient Global Impression of Status – Physical Function (PGIS-Physical Function)	X	X						X				X						X		
Patient Global Impression of Status – Symptom Severity (PGIS-Symptom Severity)	X	X						X				X						X		
**Laboratory Tests and Sample Collections**																				
Hematology	X	X			X			X				X		X	X[i]	X[i]		X	X	See footnote i
Hemoglobin A1c (HbA1c)	X	X	X	X	X	X	X	X		X		X		X				X	X	

Study I8F-MC-GPID	Study Period I Screening	Study Period II - Treatment period																	
														Unscheduled Visit (UV)[a]					
Visit number	1	2	3	4	5	6	7	8	9	10	11	12	EVa, EVb, EVc (13, 14, 15, 17, 18, 19, etc.)	EVd (16, 20, etc.)	UV	Dosing UV	Phone Follow-Up Dosing UV	ET	99	Comment
Weeks from randomization	-6	0	4	8	12	16	20	24	32	40	48	52	(+3, 6, 9, 15, 18, 21 months)	(+12, 24 months)				—	Final Visit	See footnote b
Visit interval tolerance (days)			±3	±3	±3	±3	±3	±3	±7	±7	±7	±7	±15	±15				—		
Telephone Visit									X		X		X				X			See footnote c
Clinical chemistry (with glucose)	X	X[j]			X			X				X		X	X[i]	X[i]		X	X	[j]Required at Visit 2 randomization if screening labs are older than 28 days for chemistry, lipid, and pancreatic lipase/amylase[i]
Lipid panel	X	X[j]			X			X				X		X	X[i]	X[di]		X	X	[j]Required at Visit 2 randomization if screening labs are older than 28 days for chemistry, lipid, and pancreatic lipase/amylase
Thyroid-stimulating hormone (TSH)	X																			
Serum pregnancy	X	X[k]																		For women of childbearing potential only. See Appendix 4 (Section 10.4). [k]Collect serum pregnancy at Visit 2 only if Visit 1 serum was ≥28 days prior.

Study I8F-MC-GPID	Study Period I Screening	Study Period II - Treatment period																		
														Unscheduled Visit (UV)[a]						
Visit number	1	2	3	4	5	6	7	8	9	10	11	12	EVa, EVb, EVc (13, 14, 15, 17, 18, 19, etc.)	EVd (16, 20, etc.)	UV	Dosing UV	Phone Follow-Up Dosing UV	ET	99	Comment
Weeks from randomization	-6	0	4	8	12	16	20	24	32	40	48	52	(+3, 6, 9, 15, 18, 21 months)	(+12, 24 months)				—	Final Visit	See footnote b
Visit interval tolerance (days)			±3	±3	±3	±3	±3	±3	±7	±7	±7	±7	±15	±15				—		
Telephone Visit									X		X		X				X			See footnote c
Urine pregnancy (local)		X	X	X	X	X	X	X		X		X		X				X	X	A local urine pregnancy test must be performed at Visit 2 after patient eligibility has been confirmed with the result available prior to randomization and first injection of study drug(s) for WOCBP only. Additional local urine pregnancy tests may be performed at the investigator's discretion during the study. If required per local regulations and/or

Study I8F-MC-GPID	Study Period I Screening	Study Period II - Treatment period																		
															Unscheduled Visit (UV)[a]					
Visit number	1	2	3	4	5	6	7	8	9	10	11	12	EVa, EVb, EVc (13, 14, 15, 17, 18, 19, etc.)	EVd (16, 20, etc.)	UV	Dosing UV	Phone Follow-Up Dosing UV	ET	99	Comment
Weeks from randomization	-6	0	4	8	12	16	20	24	32	40	48	52	(+3, 6, 9, 15, 18, 21 months)	(+12, 24 months)				—	Final Visit	See footnote b
Visit interval tolerance (days)			±3	±3	±3	±3	±3	±3	±7	±7	±7	±7	±15	±15				—		
Telephone Visit									X		X		X				X			See footnote c
																				institutional guidelines, pregnancy testing can also occur at other times during the study treatment period. See Appendix 4 (Section 10.4).
Follicle-stimulating hormone (FSH)	X																			Collect FSH only in women whose menopausal status needs to be determined. For participants known to be either premenopausal or postmenopausal, these tests do not need to be collected
NT-proBNP	X	X			X			X				X						X[d]		[d]Perform at ET only if participant early terminates at or prior to Week 52.

Study I8F-MC-GPID	Study Period I Screening	Study Period II - Treatment period																		
														Unscheduled Visit (UV)[a]						
Visit number	1	2	3	4	5	6	7	8	9	10	11	12	EVa, EVb, EVc (13, 14, 15, 17, 18, 19, etc.)	EVd (16, 20, etc.)	UV	Dosing UV	Phone Follow-Up Dosing UV	ET	99	Comment
Weeks from randomization	-6	0	4	8	12	16	20	24	32	40	48	52	(+3, 6, 9, 15, 18, 21 months)	(+12, 24 months)				—	Final Visit	See footnote b
Visit interval tolerance (days)			±3	±3	±3	±3	±3	±3	±7	±7	±7	±7	±15	±15				—		
Telephone Visit									X		X		X				X			See footnote c
Cardiac troponin T (cTnT)		X			X			X				X						X[d]		[d]Perform at ET only if participant early terminates at or prior to Week 52.
Calcitonin	X							X				X		X				X	X	
Cystatin C		X			X			X				X						X[d]		[d]Perform at ET only if participant early terminates at or prior to Week 52.
C-reactive protein, high-sensitivity (hs-CRP)		X			X			X				X						X[d]		[d]Perform at ET only if participant early terminates at or prior to Week 52.
Pancreatic amylase	X	X[j]			X			X		X		X		X				X	X	[j]Required at Visit 2 randomization if screening labs are older than 28 days for chemistry, lipid, and pancreatic lipase/amylase

Study I8F-MC-GPID	Study Period I Screening	Study Period II - Treatment period																		
														Unscheduled Visit (UV)[a]						
Visit number	1	2	3	4	5	6	7	8	9	10	11	12	EVa, EVb, EVc (13, 14, 15, 17, 18, 19, etc.)	EVd (16, 20, etc.)	UV	Dosing UV	Phone Follow-Up Dosing UV	ET	99	Comment
Weeks from randomization	-6	0	4	8	12	16	20	24	32	40	48	52	(+3, 6, 9, 15, 18, 21 months)	(+12, 24 months)				—	Final Visit	See footnote b
Visit interval tolerance (days)			±3	±3	±3	±3	±3	±3	±7	±7	±7	±7	±15	±15				—		
Telephone Visit									X		X		X				X			See footnote c
Lipase	X	X[j]			X			X		X		X		X				X	X	[j]Required at Visit 2 randomization if screening labs are older than 28 days for chemistry, lipid, and pancreatic lipase/amylase
eGFR (CKD-EPI)	X	X[l]			X			X				X		X				X	X	The CKD-EPI equation will be used by the central lab to estimate and report eGFR. [l]Only collect calculated eGFR with Clinical Chemistry sample if screening labs are older than 28 days.
Urinary albumin/creatinine ratio (UACR)	X							X				X		X				X	X	

Study I8F-MC-GPID	Study Period I Screening	Study Period II - Treatment period																		
														Unscheduled Visit (UV)[a]						
Visit number	1	2	3	4	5	6	7	8	9	10	11	12	EVa, EVb, EVc (13, 14, 15, 17, 18, 19, etc.)	EVd (16, 20, etc.)	UV	Dosing UV	Phone Follow-Up Dosing UV	ET	99	Comment
Weeks from randomization	-6	0	4	8	12	16	20	24	32	40	48	52	(+3, 6, 9, 15, 18, 21 months)	(+12, 24 months)				—	Final Visit	See footnote b
Visit interval tolerance (days)			±3	±3	±3	±3	±3	±3	±7	±7	±7	±7	±15	±15				—		
Telephone Visit									X		X		X				X			See footnote c
Pharmacokinetic (PK) samples		X	X		X			X				X		X				X	X	PK samples should be taken prior to dose administration at the visit and at the same time as immunogenicity samples.
Immunogenicity samples		X	X		X			X				X		X				X	X	
**Stored Samples**																				
Genetics sample		X																		
Exploratory biomarker samples		X			X			X				X								
**Randomization and Dosing**																				
Randomization		X																		
Dispense study drug		X	X	X	X	X	X	X		X		X	X	X		X				

Study I8F-MC-GPID	Study Period I Screening	Study Period II - Treatment period																		
														Unscheduled Visit (UV)[a]						
Visit number	1	2	3	4	5	6	7	8	9	10	11	12	EVa, EVb, EVc (13, 14, 15, 17, 18, 19, etc.)	EVd (16, 20, etc.)	UV	Dosing UV	Phone Follow-Up Dosing UV	ET	99	Comment
Weeks from randomization	-6	0	4	8	12	16	20	24	32	40	48	52	(+3, 6, 9, 15, 18, 21 months)	(+12, 24 months)				—	Final Visit	See footnote b
Visit interval tolerance (days)			±3	±3	±3	±3	±3	±3	±7	±7	±7	±7	±15	±15				—		
Telephone Visit									X		X		X				X			See footnote c
Injection training with autoinjector demonstration device		X																		
Observe participant administer study drug		X	X	X	X	X	X	X		X		X		X		X				Participants should administer the first dose of study drug at Visit 2 after study procedures and randomization have been completed. Review technique with the participant at each in clinic visit.<br>Sites should coach and oversee if participants self-administer study drug at a scheduled visit.

Study I8F-MC-GPID	Study Period I Screening	Study Period II - Treatment period																		
														Unscheduled Visit (UV)[a]						
Visit number	1	2	3	4	5	6	7	8	9	10	11	12	EVa, EVb, EVc (13, 14, 15, 17, 18, 19, etc.)	EVd (16, 20, etc.)	UV	Dosing UV	Phone Follow-Up Dosing UV	ET	99	Comment
Weeks from randomization	-6	0	4	8	12	16	20	24	32	40	48	52	(+3, 6, 9, 15, 18, 21 months)	(+12, 24 months)				—	Final Visit	See footnote b
Visit interval tolerance (days)			±3	±3	±3	±3	±3	±3	±7	±7	±7	±7	±15	±15				—		
Telephone Visit									X		X		X				X			See footnote c
Dispense ancillary supplies to participant		X	X	X	X	X	X	X		X				X						
Participant returns study drugs and injection supplies			X	X	X	X	X	X		X		X		X				X	X	

Abbreviations: 6MWT = 6-minute walk test; BP = blood pressure; CKD-EPI = Chronic Kidney Disease Epidemiology Collaboration; CVD = cardiovascular disease; ECG = electrocardiogram; ECHO = echocardiography; eCRF = electronic case report form; eGFR = estimated glomerular filtration rate; EQ-5D-5L = 5-Level European Quality of Life Questionnaire; ET = Early Termination; EV = Extended Visit; HF = heart failure; HFpEF = heart failure with preserved ejection fraction; HR = heart rate; HTN = hypertension; MI = myocardial infarction; NT-proBNP = N-terminal pro b-type natriuretic peptide; NYHA = New York Heart Association; T2DM = type 2 diabetes mellitus; WOCBP = women of childbearing potential.

[a] There are 3 types of Unscheduled Visits (UV): These visits can occur during the dose-escalation period and maintenance period.

- Unscheduled Visits (UV) – Unscheduled Visit per investigator discretion. All activities with a check mark in UV visit are required.
- Dosing Unscheduled Visit (UV) – these visits will include dose re-escalation. The study drug must be restarted when it is safe to do so and only while at the clinic visit. The subsequent unscheduled dosing visits will be scheduled every 4 weeks (±7 days) until maximum tolerated dose is achieved.
- Phone Follow-up Dosing Unscheduled Visit (UV) – these visits are optional phone visits.

[b] At least 52 weeks (Visit 12) of treatment are planned. Additional visits after Visit 12 will occur every 3 months. The visits occurring after Visit 12 will follow the Extended Maintenance Visit schedule in sequence (EVa, EVb, EVc, and EVd) then repeat.

[c] Telephone visits can become office visits. Site documentation will serve as the source for telephone visits. Additional, optional telephone visits may be conducted at investigator discretion.

[g] This includes glucose monitoring for participants with T2D, weekly study drug injection.

[i] Unscheduled Visits: laboratory tests may be drawn according to investigator discretion. If only re-test labs required, site is not required to complete an unscheduled visit and associated procedures.

2. Introduction

2.1. Study Rationale

Heart failure with preserved ejection fraction is a heterogenous clinical syndrome resulting from various pathophysiological processes. Among the broad spectrum of HFpEF clinical presentation, obesity-related HFpEF displays a distinct phenotype where increased visceral and ectopic adiposity as well as volume expansion plays a causal role (Kitzman and Shah 2016; Packer 2018; Miller and Borlaug 2020). Given tirzepatide's potential to decrease inflammation and fibrosis and a to reduce circulating plasma volume as a consequence of the treatment of obesity, tirzepatide may provide clinical benefit to patients with HFpEF and BMI ≥30 kg/m^2.

Study I8F-MC-GPID, also known as SUMMIT, is a Phase 3, randomized, multicenter, international, placebo-controlled, double-blinded, parallel-arm study. This study will evaluate the effect of SC QW injection of tirzepatide, MTD up to 15 mg, on the risk of death, hospitalization or emergency care due to HF, exercise capacity, and health status in participants with HFpEF and BMI ≥30 kg/m^2.

2.2. Background

There is a significant unmet need in treatment of patients with HFpEF. Tirzepatide, a GIP and GLP-1 dual agonist, has the potential to provide benefit to patients with HFpEF and BMI ≥30 kg/m^2 with not only improvements in exercise capacity and symptoms but also a reduction in HF events and/or increased survival. Supporting a causal association between obesity and HFpEF, bariatric surgery improved NYHA class, patient-reported outcomes, and echo parameters (LV wall thickness, LV relaxation) in patients with HFpEF and obesity (Mikhalkova et al. 2018). A meta-analysis of bariatric surgery also showed improvement in functional capacity 6 to 12 months after surgery in patients with obesity (Herring et al. 2016). In patients with HFpEF and obesity, diet-induced weight loss (Δ=−7 kg, 20 weeks) significantly improved symptoms (KCCQ) and exercise capacity (6MWD and peak oxygen uptake) (Kitzman et al. 2016). Furthermore, weight reduction is proven to be effective in reducing HF risk and HF hospitalizations. A large observational study has demonstrated a 62% decrease (over 8 years) of HF incidence after bariatric surgery in patients with T2DM (Aminian et al. 2019). The reduction of HF risk after bariatric surgery has been consistently demonstrated in broader patient populations and considered to be mediated by weight loss with a hazard ratio for a 10-kg weight loss being 0.77 (Sundström et al. 2017; Jamaly et al. 2019). Moreover, a self-controlled case study showed a 29% (0 to 12 months) and a 43% (13 to 24 months) risk reduction of HF events in patients with HFpEF after bariatric surgery (Shimada et al. 2016).

It has been demonstrated that tirzepatide can provide significant body weight loss and improvement of lipid and glucose metabolism in patients with T2DM (Frias et al. 2018; Wilson et al. 2020). It is known that the body weight reduction with GLP-1 RAs in patients without T2DM is higher than in patients with T2DM (Davies et al. 2015; Pi-Sunyer et al. 2015; Lingvay et al. 2018). If Study GPID is assumed to include 40% to 50% of patients with T2DM, the mean placebo-adjusted body weight percent reduction that tirzepatide can provide in 52 weeks in this study is estimated to be 15% to 16%. This is based on tirzepatide clinical data and the

understanding of body weight loss differences between patients with and without T2DM treated with GLP-1 RAs. Thus, the predicted body weight loss with treatment with tirzepatide is close to that shown with bariatric surgery. In addition, tirzepatide may provide benefit to patients with HFpEF and obesity by virtue of cardiometabolic improvements (Wilson et al. 2020). Given the wide distribution of GIP receptor in the adipose tissue, GIP is thought to be actively involved in lipid and glucose metabolism.

Therefore, it will be meaningful to assess HF clinical outcomes in addition to the functional and symptomatic endpoints in this study, thereby facilitating the understanding of the clinical benefit of tirzepatide treatment in patients with HFpEF and obesity.

2.3. Benefit/Risk Assessment

More detailed information about the known and expected benefits and risks and reasonably expected AEs of tirzepatide may be found in the Investigator’s Brochure.

3. Objectives and Endpoints

Objectives	Endpoints
Primary	
To demonstrate that a maximally tolerated tirzepatide dose up to 15 mg administered subcutaneously once weekly (SC QW) is superior to placebo based on the hierarchal composite endpoint in participants with HFpEF and BMI ≥30 kg/m^2.	A hierarchical composite of the following:<br>1. Time to all-cause mortality through the end of the treatment period<br>2. Occurrence of heart failure (HF) events through end of the treatment period, where HF events include HF hospitalization OR urgent HF visit (adjudicated)<br>o number of HF events<br>o time to first HF event<br>3. Change from baseline in the 6-minute walk test distance (6MWD) category at Week 52<br>4. Change from baseline in the Kansas City Cardiomyopathy Questionnaire (KCCQ) Clinical Summary Score (CSS) category at Week 52<br>The categories for change from baseline in the 6MWD are: 1) ≥30% worsening; 2) ≥20% and <30% worsening; 3) ≥10% and <20% worsening; 4) No change (<10% change); 5) ≥10% and <20% improvement; 6) ≥20% and <30% improvement; and 7) ≥30% improvement.<br>The categories for change from baseline in the KCCQ-CSS are: 1) ≥10-point worsening; 2) ≥5- but <10-point worsening; 3) No change (<5-point change); 4) ≥5- but <10-point improvement; and 5) ≥10-point improvement.
To demonstrate that a maximally tolerated tirzepatide dose up to 15 mg administered SC QW is superior to placebo to improve exercise capacity in participants with HFpEF and BMI ≥30 kg/m^2.	Change from baseline to Week 52 in exercise capacity as measured by 6MWD

Key Secondary (multiplicity controlled)	
Long-term weight loss	Percent change from baseline to Week 52 in body weight loss
Patient-reported symptoms and physical limitations	Change from baseline to Week 52 in the KCCQ CSS
Exercise capacity	Change from baseline to Week 24 in 6MWD
New York Heart Association (NYHA) Class	Proportion of participants with NYHA Class change at Week 52
Other Secondary	
Clinical outcome events of HF	• Time to first occurrence of HF events or CV death<br>• Time to first occurrence of HF events or all-cause death<br>• Time to first occurrence of HF events<br>• Time to recurrent events of HF events and CV death<br>• Time to recurrent events of HF events
Exploratory	
HF medication use	Change in the HF concomitant medication net use
Atrial fibrillation	Proportion of participants with atrial fibrillation
Waist circumference	Change from baseline (centimeters)
Patient-reported health-related quality of life	Change from baseline in KCCQ:<br>• Total Symptom Score (TSS)<br>• Overall Summary Score (OSS)
Patient-reported health status	Change from baseline in EQ-5D-5L:<br>o Index Score<br>o VAS Score

Patient-reported global health status	Proportion of participants with improvements in global health status from baseline as assessed by the PGIS-Overall
Patient-reported global impression of physical function	Proportion of participants with improvements in physical function from baseline as assessed by the PGIS-Physical Function
Patient-reported global symptom severity	Proportion of participants with improvements in symptom severity from baseline as assessed by the PGIS-Symptom Severity
Evaluation of prespecified biomarkers	• NT-proBNP<br>• cTnT<br>• hs-CRP

Abbreviations: BMI = body mass index; cTnT = cardiac troponin T; HFpEF = heart failure with preserved ejection fraction; hsCRP = C-reactive protein, high-sensitivity; NT-proBNP = N-terminal pro b-type natriuretic peptide; PGIS = Patient-Reported Global Impression of Status; VAS = visual analog scale.

4. Study Design

4.1. Overall Design

Study GPID is a randomized, outpatient, multicenter, international, placebo-controlled, double-blinded, parallel-arm, Phase 3 study with 2 study periods. The study is designed to evaluate the efficacy and safety of SC QW tirzepatide, MTD up to 15 mg, compared to placebo, in participants with HFpEF and obesity.

Two intervention groups will be studied:

- Tirzepatide MTD up to 15 mg SC QW
- Placebo

The study will compare treatment with tirzepatide and treatment with placebo. Assignment to tirzepatide or placebo groups will be randomly allocated in a 1:1 ratio

The starting dose of tirzepatide or placebo is 2.5 mg QW, which is to be escalated at 4-week intervals to a maximum of 15 mg QW or to the highest maintenance dose tolerated by the participant (see Section 6).

The study will consist of 2 periods:

- Study Period 1: screening period, lasting no more than 12 weeks.
- Study Period 2: treatment period, with a 20-week escalation followed by at least a 32-week maintenance period

The study will continue until approximately 52 weeks after the last participant is randomized. The maximum duration of an individual's participation is estimated to be 120 weeks and will depend on duration of study enrollment.

Participant Visit Scheme

Study participants will undergo screening assessments and procedures, randomization, and double-blinded treatment with tirzepatide or placebo. Assessments and procedures to be conducted in each treatment period are described in the SoA (Section 1.3) and in Study Assessments and Procedures (Section 8).

<u>Screening</u>

Screening procedures will be performed at Visit 1. Visit 1 procedures may be conducted over more than 1 day as long as all activities are completed within the allowable visit tolerance for each visit. The duration of Visit 1 is anticipated to be <6 weeks but may be longer. Visit 1 should no more than 12 weeks from date of informed consent.

At Visit 1, two 6MWTs will be conducted. The investigator must ensure that the participant is recovered from completion of the first 6MWT prior to conducting the second 6MWT (at least 1 hour between each test). The screening 6MWTs may be conducted over more than 1 day.

<u>Randomization</u>

At Visit 2, prior to randomization, the participant needs to complete the 6MWT. Visit 2 may need to be conducted over 2 in-clinic visits (considered Visit 2a and Visit 2b) if a repeat 6MWT is necessary to assess participant eligibility. If the participant is required to return to repeat the 6MWT, the remaining procedures should be conducted at the second in-clinic visit for randomization (Visit 2b), more than 10 days from Visit 2a. See Section 10.10.1 for details on when a participant must return for a second Visit 2 6MWT.

Participants will be randomized and receive blinded study drug at the end of Visit 2 after all screening procedures are completed. The participant must not receive study drug until all eligibility criteria, including the 6MWT, are met.

Treatment

Starting from randomization, the participant receives study drug and procedures are conducted as described in the SoA (Section 1.3). Every effort should be made by the investigator to maintain participants on study drug.

Study drug dose will be escalated as illustrated in the study schema (Section 1.2). Dose escalation will continue until the participant reaches the maintenance dose of either 5 mg, 10 mg, or 15 mg. Participants who do not tolerate the first dose escalation (that is, from 2.5 mg to 5 mg [or placebo equivalent]) will need to discontinue from study drug.

Participants begin treatment with either tirzepatide or placebo starting at 2.5 mg given as a subcutaneous (SC) injection every week (QW). The dose level is increased by 2.5 mg increments approximately every 4 weeks for the first 20 weeks according to the participant's tolerability, reaching a MTD up to 15 mg. Once a MTD has been achieved, participants will continue at this MTD until study end, treatment discontinuation, or study discontinuation. Dose modifications will be permitted during the study under the circumstances specified in Section 6.6.

During the study treatment period, if an unscheduled visit or telephone visit is deemed necessary to support participant compliance, this is allowable at the discretion of the investigator site personnel. Optional telephone visits can be performed approximately 2 weeks after starting study drug and after each dose increase.

Participants will continue into the extended maintenance period with the same treatment assignment starting with Visit 13. Extended visits (EV) continue until criteria for study discontinuation is met or study ends (see Study Closeout and Final Visit below).

Study Closeout and Final Visit

The study will continue until approximately 52 weeks after the final participant is randomized. A study duration of approximately 30 months is planned.

Approximately 3 months prior to the anticipated end of study, a study closeout will be declared. During the study closeout, a final visit (Visit 99) will be planned for each participant, with the exception of those who have died or prematurely discontinued from the study (Section 7.2). Study procedures for the final visit will be performed as outlined in the SoA (Section 1.3).

All unused study drug (unused single-dose pens) must be returned for compliance and final drug accountability. The sharp items container should also be returned to site or disposed of per local regulations.

4.2. Scientific Rationale for Study Design

Study GPID is a Phase 3 study designed to examine the efficacy and safety of SC QW tirzepatide MTD compared with placebo in participants with HFpEF and BMI ≥30kg/m^2.

A placebo comparator was selected for this trial in accordance with regulatory guidance (FDA 2007; EMA 2016). Inclusion of a placebo comparator in Study GPID will allow for a direct assessment of the safety and efficacy of tirzepatide in participants with HFpEF and obesity.

Additionally, there is currently no approved therapy to be used as an active comparator in this population.

An endpoint assessment at 52 weeks of treatment is considered appropriate to assess the improvement of symptom and functional capacity. An extended maintenance treatment period increases the opportunity to evaluate HF events and outcomes.

The parallel-group design for treatment comparison was chosen to avoid any interaction between treatments that may interfere with the interpretation of the trial outcome. To minimize potential confounding effect of changes to concomitant medications, participants will be permitted to use the stable dose of concomitant medications that they require during the study. Medications that may interfere with the assessment of efficacy and safety characteristics of the study drug will not be allowed (see Section 6.5).

The primary endpoints were selected to assess the clinical benefit that tirzepatide could provide to patients in a holistic manner.

Assessment of HF events is relevant to HFpEF, which is characterized by a high frequency of recurrent HF hospitalizations. Moreover, hospitalization events reflect disease progression and high subsequent risk and predisposition, both of readmission and death (Solomon et al. 2007).

The conventional reporting of HF outcomes uses CV death and hospitalization for HF as a composite endpoint. Limitations of this approach include handling of each endpoint with equal importance irrespective of clinical significance, and ignorance of symptomatic and functional endpoints that are also relevant to patients from a clinical standpoint. To overcome this problem, the Win ratio approach was selected using 4 clinically meaningful endpoints as a hierarchical composite (Pocock et al. 2012; Ferreira 2020). This approach has several advantages over the conventional outcome assessment: 1) it prioritizes the more significant component of the composite (for example, death); 2) more of major component events are included in the analysis; and 3) it allows holistic evaluation of drug effects in a complex disorder such as HF.

Obesity is associated with cardiometabolic derangement and depleted myocardial energetics. During exercise in those with obesity, the heart does not increase adenosine triphosphate, leading to cardiac function impairment and exercise intolerance. It has been shown that weight loss reverses the energetic changes (Rayner et al. 2020). In patients with HFpEF, due to increased filling pressures, functional capacity is severely impaired, and patients can develop symptoms with light exercise. As a result, the ability to perform activities of daily living is deteriorated. Obesity is one of the main attributes to worsen quality of life in patients with HFpEF (Reddy et al. 2020). Therefore, 6MWD and KCCQ are meaningful endpoints to evaluate the clinical benefit of tirzepatide in patients with HFpEF and obesity.

For the primary composite endpoint, participants will be classified based on 6MWD categories of change from baseline with 10% incremental for improvement or worsening and KCCQ-CSS categories of change from baseline with 5- or 10-point improvements or worsening. It was estimated that the minimal important difference in 6MWD is approximately 30 to 37 meters in individuals with chronic HF. Relative to the mean 6MWD in these study populations (approximately 350 meters and 480 meters), the 30 to 37 meters is approximately 8% to 9% (Shoemaker et al. 2013; Täger et al. 2014).

For KCCQ-CSS, the categories were specified based on the evidence in the literature suggesting that 5- and 10-point changes in KCCQ summary scores represent small and moderate-to-large clinical changes, respectively. Each 5-point improvement in KCCQ scores has been demonstrated to be associated with improvements in CV mortality and all-cause hospitalization rates, while each 5-point decrement in KCCQ scores was associated with increased risk of CV death and the combined endpoint of CV death and hospitalization, in a linear fashion (Spertus et al. 2020).

4.2.1. Patient Input into Design

The sponsor involved patients in the design of this study by engaging patients in virtual collaborative events. The insights gained from these events were used to ensure that the study design is supportive of the well-being of the study participants and that the study procedures can be implemented effectively at the investigative sites.

4.3. Justification for Dose

Tirzepatide doses of up to 15 mg administered SC QW will be evaluated in this study.

Participants may be treated with lower maintenance doses of 5 mg or 10 mg if they do not achieve full dose escalation to 15 mg and/or do not tolerate 15 mg.

These doses and associated escalation schemes were selected based on assessment of safety, efficacy (glycemic and weight loss benefit), and GI tolerability data followed by exposure response modeling of data in participants with T2DM in Phase 1 and Phase 2 studies. Dosing algorithms starting at a low dose of 2.5 mg accompanied by dose escalation of 2.5 mg increments every 4 weeks should permit time for development of tolerance to GI events and are predicted to minimize GI tolerability concerns.

The dose selection of tirzepatide is based on the findings of the Phase 2 study results. Tirzepatide doses of 5 mg, 10 mg, and 15 mg QW have been tested and compared with dulaglutide 1.5 mg QW or placebo in a Phase 2 study (Frias et al. 2018). While all 3 doses of tirzepatide significantly improved the glycemic control versus dulaglutide, the largest difference was observed in the 15-mg tirzepatide treatment group. Moreover, the reduction in body weight on tirzepatide was also dose-dependent and greatest in the 15 mg QW treatment group.

4.4. End of Study Definition

The end of the study is defined as the date of the last visit or last scheduled procedure shown in the SoA for the last participant in the trial globally.

The criteria used to determine if a participant has completed the study will be described in the SAP.

5. Study Population

Prospective approval of protocol deviations to recruitment and enrollment criteria, also known as protocol waivers or exemptions, is not permitted.

5.1. Inclusion Criteria

Participants are eligible to be included in the study only if all of the following criteria apply:

Age

1. Participant must be at least 40 years of age inclusive, at the time of signing the ICF.

Type of Participant and Disease Characteristics

2. 6MWD ≤465 meters at both Visit 1 tests, between ≥100 meters and ≤425 meters at Visit 2 and change from the preceding qualifying 6MWD is <20% and <40 meters. See Section 10.10.1 for the flow diagram of the below qualifiers.
   - *If* Visit 2a 6MWD is both between 100 and 425 meters and…
     - <20% AND <40 meter change from the higher of the two 6MWDs conducted at Visit 1, *then* participant meets this inclusion criterion.
     - ≥20% OR ≥40 meter change from the higher of the two 6MWDs at Visit 1, *then* participant must attend Visit 2b. If at Visit 2b, the 6MWD is between 100 and 425 meters and <20% AND <40 meter change from preceding (Visit 2a) 6MWD, then participant meets this inclusion criterion.
3. Chronic HF (NYHA Class II-IV) diagnosed for at least 3 months before Visit 1
4. LVEF ≥50% demonstrated by echocardiogram performed at Visit 1 or within 6 months of Visit 1
5. At least 1 of the following to document evidence of HF:
   - Elevated NT-proBNP >200 pg/mL for participants without atrial AF or >600 pg/mL for participants with AF, as analyzed at the central laboratory at Visit 1
     OR
   - Evidence of structural heart disease:
     - LA enlargement (any of the following: LAV index ≥29 mL/m^2, or LAV >58 mL in male participants and >52 mL in female participants, or LA area >20 cm^2, or LA diameter >40 mm in male and >38 mm in female participants) determined by echocardiogram at Visit 1 or within 6 months of Visit 1

     OR
   - Evidence of elevated filling pressure:
     - At rest (PCWP ≥15 mmHg or LVEDP ≥15 mmHg) or with exercise (PCWP ≥25 mmHg) (based on historical record, not associated with hospitalization for decompensation of HF, within 2 years of Visit 1), or
     - E/e' ratio >15 (septal) or >13 (average of septal and lateral) determined by echocardiogram at Visit 1 or within 6 months of Visit 1

Note: Supporting medical documentation is required in all instances

6. Either one of:
    - eGFR <70 mL/min/1.73m^2 at Visit 1, OR
    - HF decompensation within 12 months of Visit 1, defined as hospitalization for HF requiring IV diuretic treatment or urgent HF visit requiring IV diuretic treatment
    Note: Supporting medical documentation is required in all instances
7. Stable dose of all concomitant HF medications (these may include beta blockers, ACEis, ARBs, and/or MRAs, ARNI, and SGLT2is), except for oral diuretics, for at least 4 weeks prior to Visit 1 and throughout the screening period
8. If treated with oral diuretics, dose must be stable for at least 2 weeks prior to Visit 1 and throughout the screening period; volume control must be optimally achieved in the opinion of the investigator
9. KCCQ-CSS ≤80 at Visit 1

Weight

10. BMI ≥30.0 kg/m^2 at Visit 1

Sex

11. At the time of signing the ICF:
    a. **Male participants**: Male participants with partners of childbearing potential should be willing to use reliable contraceptive methods for the duration of the trial and for 4 months thereafter (see Appendix 4 [Section 10.4]).
    b. Female participants:
    - Female participants not of childbearing potential may participate and include those who are infertile due to surgical sterilization and/or postmenopausal. Please refer to Appendix 4 (Section 10.4) for definitions.
    - Female participants of childbearing potential (not surgically sterilized and between menarche and 1-year postmenopausal) must:
        - test negative for pregnancy at Visit 1 based on a serum pregnancy test followed by a negative urine pregnancy test within 24 hours prior to exposure and agree to use 2 forms of effective contraception, if sexually active, where at least 1 form is highly effective, for the duration of the trial and for 2 months after the last injection, and
        - not be breastfeeding.

Contraceptive use by men or women of childbearing potential should be consistent with local regulations regarding the methods of contraception for those participating in clinical trials. See Appendix 4 (Section 10.4) for guidance.

Informed Consent

12. Capable of giving signed informed consent as described in Appendix 1 (Section 10.1), which includes compliance with the requirements and restrictions listed in the ICF and in this protocol.

5.2. Exclusion Criteria

Participants are excluded from the study if any of the following criteria apply:

Medical Conditions

13. Myocardial infarction, coronary artery bypass graft surgery, or other major CV surgery/intervention, stroke or transient ischemic attack in past 90 days, or unstable angina pectoris in past 30 days, prior to Visit 1 or during screening; Have NYHA Class I heart failure at Visit 1 and Visit 2

14. Dominant contribution of noncardiac causes to exercise impairment or symptoms
    - Lung disease: pulmonary arterial hypertension, chronic thromboembolic pulmonary hypertension (CTEPH), or severe pulmonary disease including (COPD)
    - Other medical conditions: severe anemia (hemoglobin level <9 g/dL) at Visit 1, untreated thyroid disease or TSH >4.78 mU/L at Visit 1, or significant musculoskeletal disease
    - Orthopedic conditions that limit the ability to walk, such as severe arthritis in the leg, knee, hip injuries, hemiplegia, or amputation with artificial limb without stable prosthesis function for the past 3 months
    - Any condition that in the opinion of the investigator would interfere with the assessment of 6MWT

15. LVEF <40% by local echocardiography, MRI or other modalities documented any time within 2 years of Visit 1
16. Acute decompensated HF (exacerbation of HF) requiring IV diuretics, IV inotropes, or IV vasodilators, or left ventricular assist device (LVAD) within 4 weeks prior to Visit 1, and/or during the screening period until randomization
17. Impaired renal function, defined as eGFR <15 mL/min/1.73 m^2 (CKD-EPI) or requiring dialysis at Visit 1
18. Any one of the following:
    - Systolic blood pressure (SBP) ≥180 mmHg at Visit 1
    - SBP >160 mmHg both at Visit 1 and at Visit 2
    - Have symptomatic hypotension or SBP <100 mmHg at Visit 1 or Visit 2
19. Resting heart rate (sinus rhythm) ≥100 bpm at either Visit 1 or Visit 2
20. Atrial fibrillation or atrial flutter with a resting heart rate >110 bpm documented by ECG at Visit 1 and Visit 2
21. Cardiac amyloidosis or cardiomyopathy based on accumulation disease (for example, haemochromatosis, Fabry disease), muscular dystrophy, cardiomyopathy with reversible causes (for example, stress cardiomyopathy), hypertrophic cardiomyopathy, Chagas cardiomyopathy or known pericardial constriction, or any severe (obstructive or regurgitant) valvular heart disease likely to lead to surgery during the study period
22. Completed prior surgical treatment for obesity or had liposuction or abdominoplasty within 1 year prior to Visit 1. Participants who plan to have surgical treatment for obesity or liposuction or abdominoplasty during the duration of the study are excluded.

23. Participation in a structured exercise training program in the 1 month prior to Visit 1 or planning to start a program during the study
24. Have T1DM
25. For participants with T2DM:
- Have uncontrolled diabetes requiring immediate therapy (such as diabetic ketoacidosis) at Visit 1 or Visit 2, in the judgement of the physician
- Have had 1 or more events of severe hypoglycemia and/or 1 or more events of hypoglycemia unawareness within 6 months prior to Visit 1 (see Section 10.5.1.1 for definition of hypoglycemia)
- Have HbA1c ≥9.5% (80 mmol/mol) at Visit 1, as analyzed at the central laboratory
- Have a history of proliferative diabetic retinopathy or diabetic maculopathy. Patients with severe nonproliferative diabetic retinopathy that requires acute treatment are also excluded.
- Treated with premix or prandial insulins or intensified insulin regimens (multiple daily injection with basal and prandial insulins or insulin pump therapy) at Visit 1

26. History of acute or chronic pancreatitis or at high risk for acute pancreatitis (for example, serum triglyceride level >500 mg/dL [5.65 mmol/L])
27. Have acute or chronic hepatitis, signs and symptoms of any other liver disease other than nonalcoholic fatty liver disease, or any of the following, as determined by the central laboratory during Visit 1:
- ALT or AST levels >2.5X the ULN for the reference range.

Note: Participants with nonalcoholic fatty liver disease are eligible to participate in this trial if their ALT level is ≤3.0X the ULN for the reference range.

28. Have a calcitonin level at Visit 1 of:
    - ≥20 ng/L, if eGFR is ≥60 mL/min/1.73 m^2
    - ≥35 ng/L, if eGFR is <60 mL/min/1.73 m^2
29. Have a family or personal history of medullary thyroid carcinoma (MTC) or Multiple Endocrine Neoplasia (MEN) Syndrome type 2
30. Have a history of an active or untreated malignancy or are in remission from a clinically significant malignancy (other than basal- or squamous-cell skin cancer, in situ carcinoma of the cervix, or in situ prostate cancer) for less than 5 years
31. Have a history of any other condition (such as known drug or alcohol abuse, diagnosed eating disorder, or other psychiatric disorder) that, in the opinion of the investigator, may preclude the participant from following and completing the protocol
32. Have a known clinically significant gastric emptying abnormality (for example, severe diabetic gastroparesis or gastric outlet obstruction) or chronically take drugs that directly affect GI motility

Prior/Concomitant Therapy

33. Treatment with any incretin, GLP-1 RA, or pramlintide in the 3 months prior to Visit 1
34. Discontinuation of any incretin, GLP-1 RA, or pramlintide due to intolerability any time prior to Visit 1

35. Have any other condition not listed in this section (for example, hypersensitivity or intolerance) that is a contraindication to GLP-1 RA
36. Implantable cardioverter defibrillator (ICD) implantation within 1 month prior to Visit 1 or planned implantation during the course of the study
37. Currently implanted left ventricular assist device (LVAD)
38. Cardiac resynchronization therapy (CRT) implanted within 6 months prior to Visit 1 or planned implantation during the course of the trial
39. Current use of medication associated with weight gain or weight loss, except when on stable dose for at least 3 months prior to Visit 1, and expected to be stable during the study period

Prior/Concurrent Clinical Study Experience

40. Have participated within the last 6 months in a clinical study involving an investigational product

Other Exclusions

41. Investigator site personnel directly affiliated with this study and/or their immediate families. Immediate family is defined as a spouse, parent, child, or sibling, whether biological or legally adopted
42. Lilly employees

5.3. Lifestyle Considerations

Study participants should be instructed not to donate blood or blood products during the study and for 8 weeks following the study.

5.4. Screen Failures

Screen failures are defined as participants who consent to participate in the clinical study but are not subsequently randomly assigned to study drug. A minimal set of screen failure information is required to ensure transparent reporting of screen failure participants to meet the CONSORT publishing requirements and to respond to queries from regulatory authorities. Minimal information includes demography, screen failure details, eligibility criteria, and any SAE.

Individuals who do not meet certain criteria for participation in this study (screen failure) may be rescreened once. Additionally, individuals who do not complete screening within 12 weeks from ICF date should be screen failed and may be rescreened. Rescreened participants should sign a new ICF and be assigned a new participant number. The interval between rescreenings should be at least 2 weeks. For participants who may have screen failed due to HbA1c criterion not met, the time to permit rescreening is at least 8 weeks. If, in the opinion of the investigator, an ineligible laboratory test result is the result of an error or extenuating circumstance, then that parameter can be retested once without the participant having to be rescreened. For rescreened participants, a repeat echocardiogram is not permitted.

Participants may be rescreened for the following reasons:

- Have become eligible to enroll in the study as the result of a protocol amendment

- Status has changed such that the eligibility criterion that caused the participant to screen fail would not cause the participant to screen fail again
- Completed screening and met all inclusion and exclusion requirements but are unable to be enrolled due to extenuating circumstances (such as severe weather, death in family, or child illness)

6. Study Intervention

Study drug is defined as any investigational intervention(s), marketed product(s), placebo, or medical device(s) intended to be administered to/used by a study participant according to the study protocol. For this study, 'study intervention' and 'study drug' are equivalent.

6.1. Study Interventions Administered

**Intervention Name**	Placebo	Tirzepatide (LY3298176)
**Type**	Drug (placebo)	Drug
**Dose Formulation**	Single-dose pen	Single-dose pen
**Unit Dose Strengths**	Not applicable	2.5 mg, 5 mg, 7.5 mg, 10 mg, 12.5 mg, 15 mg
**Dosage Levels**	Not applicable	15 mg QW<br>(or maximum tolerated dose of 5 mg QW or 10 mg QW)
**Route of Administration**	Subcutaneous	Subcutaneous
**Use**	Placebo	Experimental
**IMP and NIMP**	IMP	IMP
**Sourcing**	Provided centrally by the sponsor and dispensed via IWRS	
**Packaging and Labeling**	Study drug will be provided in autoinjectors (single-dose pens), packaged in cartons to be dispensed.<br>Clinical study materials will be labeled according to country regulatory requirements.	

Abbreviations: QW = weekly; IMP = investigational medicinal product; IWRS = interactive web-response system; NIMP = non-investigational medicinal product.

The following table shows the randomized study drugs for the entire study.

Treatment Group	Treatment Period Interval					
	**Weeks 0 to 3**	**Weeks 4 to 7**	**Weeks 8 to 11**	**Weeks 12 to 15**	**Weeks 16 to 19**	**Weeks 20 to End of Treatment Period**
Tirzepatide	2.5 mg	5 mg	7.5 mg	10 mg	12.5 mg	15 mg or MTD
Placebo	→					

Abbreviation: MTD = maximum tolerated dose.

There are no restrictions on the time of day each weekly dose of study drug is given, but it is advisable to administer the SC injections on the same day and same time each week. The actual date, time, and injection site location of all dose administrations will be recorded in the diary by the participant. If a dose of study drug is missed, the participant should take it as soon as possible, unless it is within 72 hours of the next dose, in which case that dose should be skipped, and the next dose should be taken at the appropriate time. The day of weekly administration can be changed if necessary, as long as the last dose was administered 72 or more hours previously.

All participants will inject study drug subcutaneously in the abdomen or thigh using the injection supplies provided; a caregiver may also administer the injection in the participant's upper arm. The injection site location of all dose administrations will be recorded by the participant. A new autoinjector will be used for each injection. If study drug is to always be injected in the same body region, participants should be advised to rotate injection sites each week.

6.1.1. Medical Devices

The combination product provided for use in the study is a tirzepatide or matching placebo autoinjector.

6.2. Preparation/Handling/Storage/Accountability

- The investigator or designee must confirm appropriate storage conditions have been maintained during transit for all study drug received and any discrepancies are reported and resolved before use of the study drug.
- Only participants enrolled in the study may receive study drug. Only study personnel may supply study drug.
- All study drug must be stored in a secure, environmentally controlled, and monitored (manual or automated) area in accordance with the labeled storage conditions with access limited to the investigator and authorized study personnel.
- The investigator, institution, or the head of the medical institution (where applicable) is responsible for study drug (includes study drug and autoinjector or single-dose pen) accountability, reconciliation, and record maintenance (that is, receipt, reconciliation, and final disposition of records).
- Study site staff must regularly assess whether the participant is correctly administering the assigned study drug and storing study drug according to the provided instructions.

Further guidance and information for the final disposition of unused study drugs are provided in the study training materials.

The investigator or designee is responsible for the following:

- Explaining the correct use of the study drug to the participant
- Verifying that instructions are followed properly
- Maintaining accurate records of study drug dispensing and collection as well as records of interruptions in study drug administration
- Instructing the participant to discard all used autoinjectors for study drug in a closeable, puncture-resistant container and dispose according to local regulations, and
- Considering dose adjustment of antihyperglycemic medications (see Section 6.5) at Visit 2 from first administration of study drug.

6.3. Measures to Minimize Bias: Randomization and Blinding

This is a double-blind study.

Participants who meet all criteria for enrollment will be randomized to one of the study treatment groups at the end of Visit 2. Assignment to treatment groups will be determined by a computer-generated, random sequence using an IWRS. Participants will be randomized in a 1:1 ratio to receive tirzepatide or placebo. The randomization will be stratified by HF decompensation (hospitalization for HF requiring IV diuretic treatment or urgent HF visit requiring IV diuretic treatment) within 12 months of screening (Y/N), diagnosed T2DM (Y/N), and BMI ≥35 kg/m^2 (Y/N).

Before the study is initiated, the log in information and directions for the IWRS will be provided to each site.

Study drug will be dispensed at the study visits shown in the SoA.

Returned unused study drug should not be re-dispensed to the participants.

The IWRS will be programmed with blind-breaking instructions. In case of an emergency, the investigator has the sole responsibility for determining if unblinding of a participant's drug assignment is warranted. Participant safety must always be the first consideration in making such a determination. If a participant's drug assignment is unblinded, the sponsor must be notified immediately after breaking the blind. The date and reason that the blind was broken must be recorded in the source documentation and CRF, as applicable.

If an investigator, site personnel performing assessments, or participant is unblinded, the participant must be discontinued from the study. In cases where there are ethical reasons to have the participant remain in the study, the investigator must obtain specific approval from a sponsor CRP for the participant to continue in the study.

6.4. Study Intervention Compliance

Participant compliance with study drug and adherence to study visits and procedures will be vitally important to meet the study objectives. This will be emphasized through comprehensive site training and consistently monitoring participant retention throughout the duration of the study.

Study drug compliance will be determined by the following:

- Study drug administration data will be recorded by the participants in the participant study diary and reviewed by the investigator at each study visit.
- The participants will be instructed to return any unused study drug and/or empty cartons at the next visit to the study site for the purpose of performing drug accountability.

Treatment compliance for each visit interval is defined as taking at least 75% of the required doses of study drug. Similarly, a participant will be considered significantly noncompliant if he or she is judged by the investigator to have intentionally or repeatedly taken more than the prescribed amount of medication (more than 125%).

In addition to the assessment of a participant's compliance with the study drug administration, other aspects of compliance with the study drug will be assessed at each visit based on the participant's adherence to the visit schedule, completion of study diaries, and any other parameters the investigator considers necessary.

Participants considered to be poorly compliant with their medication and/or the study procedures will receive additional training and instruction, as required, and will be reminded of the importance of complying with the protocol.

6.5. Concomitant Therapy

Any medication or vaccine (including over-the-counter or prescription medicines, vitamins, and/or herbal supplements) or other specific categories of interest that the participant is receiving at the time of enrollment or receives during the study must be recorded along with:

- Reason for use
- Dates of administration including start and end dates
- Dosage information including dose and frequency for concomitant therapy of special interest

The sponsor should be contacted if there are any questions regarding concomitant or prior therapy.

Initial doses of tirzepatide delay gastric emptying and have the potential to transiently impact the rate of absorption of concomitantly administered oral medicinal products. Tirzepatide should be used with caution in participants receiving oral medicinal products that require rapid GI absorption following the initial doses of tirzepatide, as exposure to oral medications may be increased.

<u>Prevention of Hypoglycemia</u>

Similar to GLP-1 RAs, tirzepatide does not generally cause hypoglycemia, but it is recommended to decrease the dose of concomitant sulfonylurea or insulin to reduce the risk of hypoglycemic episodes in patients with T2DM. For participants with T2DM, specific, individually tailored adjustments of the respective antihyperglycemic medications should be considered during the entire study. At Visit 2, with the initiation of study drug, the dose adjustments to the following concomitant glucose lowering medications are recommended.

Sulfonylureas: Sulfonylurea dose is recommended to be reduced at least 50% or discontinued, especially if the participant is receiving a low dose at randomization.

Insulins: For participants on basal insulin and with screening HbA1c ≤8.5%, the daily dose is recommended to be reduced by at least 20%.

During the dose escalation period, consider adjusting the total daily dose of insulin, if required for controlling worsening hyperglycemia and its acute complications.

During the maintenance period, further insulin dose reduction for the prevention of hypoglycemia is to be considered at the investigator's discretion.

Standard of Care for T2DM

The standard of care for diabetes may be adjusted at the discretion of the investigator as clinically indicated in accordance with local standard of care and professional society guidelines.

Participants will be permitted to use concomitant medications that they require during the study, except certain medications that may interfere with the assessment of efficacy and safety characteristics of the study drug. Prohibited medications include all GLP-1 RAs and pramlintide. Discontinuation of dipeptidyl peptidase 4 inhibitors at randomization is recommended in line with guidelines. Similarly, the use of dipeptidyl peptidase 4 inhibitor therapies during the study is also discouraged (Davies et al. 2018). The use of SGLT2i for treatment of T2DM is not prohibited; however, it is preferred that other agents are considered prior to initiating a SGLT2i while participating in study.

Regarding the use of insulin, participants can be included if treated with basal insulin only; and are allowed to use prandial insulin if needed during the study to attain optimal glucose control.

Hyperglycemia Rescue

Other medications for glycemic control for participants with T2DM meeting severe, persistent hyperglycemia criteria for rescue may be added during the study at the investigator's discretion.

Rescue therapy with glucose-lowering agents, including basal and prandial insulins, may be medically indicated in situations after randomization due to severe, persistent hyperglycemia or early discontinuation of study drug.

Hyperglycemia rescue criteria will be determined from values recorded in T2DM participant diaries. If a diary value equal to or greater than the glycemic threshold for rescue (see definitions below) is recorded, that value should be confirmed by a repeat fasting glucose text within 48 hours (for example, local laboratory). Intensification of T2DM therapy should be initiated if confirmed fasting glucose values are:

- ≥15.0 mmol/L (270 mg/dL) from baseline to Week 6 over at least a 2-week period (at least 2 consecutive values) after randomization
- ≥13.3 mmol/L (240 mg/dL) from Week 6 to Week 12 over at least a 2-week period (at least 2 consecutive values)
- ≥11.1 mmol/L (200 mg/dL) from Week 12 to end of trial over at least a 2-week period (at least 2 consecutive values)

In addition, if HbA1c is >9.0% at Week 12 or >8.0% at Week 24 or later in the study, glucose-lowering therapy should be adjusted to improve glycemic control as outlined above. In the event a participant's HbA1c values are less than these thresholds but are higher than what the investigator feels comfortable leaving untreated, glucose-lowering medication can be adjusted. In addition, if participants develop symptoms of hyperglycemia (for example, polyuria and polydipsia), the investigator should implement measures to determine glycemic control and adjust as necessary. For participants newly diagnosed with T2DM during the trial, appropriate glucose-lowering therapy should be initiated per standard of care.

Standard of Care for Heart Failure

Anticipated treatment for heart failure should be decided prior to randomization.

Both American College of Cardiology/American Heart Association and European Society of Cardiology guidelines recommend symptom management with diuretic agents in patients with excess volume, as well as aggressive risk factor management for comorbidities for the treatment of HFpEF (van der Meer JACC 2019). Optimization of volume status and proactive adjustment of diuretic doses will help control symptoms and volume overload.

Participants should remain on stable doses of medications to treat heart failure condition and comorbidities such as hypertension. With the exception of diuretics, dose modification or alteration of such background therapies should be avoided unless all other measures fail to improve the participant's condition. However, if the participant's condition warrants a change in any of these medications, it will be allowed at the discretion of the investigator.

Management of Participants with Gastrointestinal Symptoms

In the Phase 2 program, the most commonly reported TEAEs for participants receiving tirzepatide were nausea, vomiting, and diarrhea. To mitigate GI symptoms and manage participants with intolerable GI AEs, the investigator should:

- Advise participants to eat smaller meals, for example, splitting 3 daily meals into 4, or more smaller meals, and to stop eating when they feel full. Also, participants may be informed that lower-fat meals could be better tolerated.
- Prescribe symptomatic medication (for example, anti-emetic or antidiarrheal medication) per local country availability and individual participant needs. Use of symptomatic medication should be captured as concomitant medication in the eCRF.
- Temporarily interrupt study drug. See Section 6.6.1. The data related to temporary interruption of study drug should be documented in source documents and entered on the eCRF.
- After the interruption, follow the guidance for restarting study drug (Section 6.6.2).

If intolerable GI symptoms or events persist despite the above measures, other dose modifications (see Section 6.6) may be considered.

6.6. Dose Modification

Interventions to optimize study drug tolerance and adherence may be employed throughout the study and include, but are not limited to, brief temporary interruptions and use of additional medications to manage symptoms.

Dose modifications, including temporary interruption and de-escalation may occur to manage issues with tolerability. It is preferred to attempt a temporary dose interruption (at any time) (Section 6.6.1) to manage tolerability issues. After a temporary dose interruption, participants may resume study drug at the same dose, re-escalate to the prior dose level (if re-escalation is desired or required), or resume at a lower MTD dose level, as tolerated. Guidance for resuming study drug after a temporary dose interruption should be followed (Section 6.6.2).

Unwarranted excessive weight reduction: **Dose interruption is preferred over de-escalation for slowing unwarranted excessive weight loss; however, the method used is at the investigator's discretion.** After the dose escalation period and at the investigator's discretion, when excessive weight reduction is not warranted due to safety concerns, the investigator may choose to adjust the study drug dose without first attempting a temporary dose interruption. The participant's study drug dose will be permanently reduced to 5 mg, in a blinded fashion for the remainder of the study and the dose cannot be re-escalated. A dose adjustment for unwarranted excessive weight reduction is completed through the IWRS. Participants on 5 mg will have blinded study drug temporarily interrupted (Section 6.6.1).

Dose reductions for unwarranted excessive weight loss may occur at scheduled and unscheduled visits.

6.6.1. Temporary Interruption

Temporary study drug interruption should be utilized to manage tolerability issues. After randomization, the investigator may interrupt study drug, for example, due to an AE (such as nausea vomiting, or excessive unwarranted weight loss) or a clinically significant laboratory value. Guidance for resuming study drug after a temporary dose interruption should be followed (Section 6.6.2). If study drug interruption is due to an AE, the event is to be followed and documented.

The interruption of study drug is not equivalent to discontinuation from study treatment. Any interruption of study drug does not affect the study visit structure/schedule per the SoA (Section 1.3). Even if study drug is interrupted, study procedures should continue during the dose interruption. Every effort should be made by the investigator to maintain participants in the study and to restart study drug promptly after any interruption, as soon as it is safe to do so (see Section 6.6.2. for restarting study drug). Dose interruptions are managed through the IWRS. The data related to interruption of study drug will be documented in source documents and entered on the eCRF, however participant noncompliance should not be recorded as interruption of study drug on the eCRF.

Participants who have a temporary interruption of the study drug will continue participating in the trial according to the protocol to collect all planned efficacy and safety measurements (Section 1.3).

6.6.2. Restarting Study Drug after Interruption

In certain situations, the investigator may need to temporarily interrupt study drug. Every effort should be made by the investigator to maintain participants on study drug and to restart study drug after any temporary interruption, as soon as it is safe to do so. Distribution of study drug at the correct dose will be per IWRS instructions.

If study drug interruption is…	then…
1 or 2 consecutive doses	Participant restarts study drug at last administered dose, per escalation schedule. If the participant has reached maintenance dose level, the study drug dose level will restart at the same prior achieved maintenance dose level.
3 or more consecutive doses	Participant restarts study drug (at 5 mg, managed by IWRS) and repeats dose escalation scheme until maintenance dose is reached. If maintenance dose has previously been established, the dose escalation cannot not exceed the prior achieved maintenance dose level.
Due to an AE	The event is to be documented and followed according to the procedures in Section 8.3.
Due to intolerable persistent GI AE	Participants should be treated as suggested in Section 6.5.

Abbreviations: AE = adverse event; GI = gastrointestinal; IWRS = interactive web response service.

Investigators should inform the sponsor that study drug has been temporarily interrupted.

Participants are not required to re-escalate to the prior maintenance dose level if a tolerability issue recurs during dose re-escalation.

If this attempted dose re-escalation to the prior maintenance dose level is not tolerated, the dose should be reduced to the next lower 5 mg incremental dose that was tolerated (for example, 5 mg or 10 mg). The participant will remain at that dose level for the duration of the study.

For participants receiving 5mg maintenance dose, no dose de-escalation is permitted. Only dose interruption is permitted to manage tolerability issues (Section 6.6.2). This can be performed through IWRS.

In the event that a participant has a temporary interruption that requires extending the escalation beyond Visit 8, unscheduled visits are allowed in the IWRS to facilitate a 4-week dispensing schedule to complete the escalation. Unscheduled visits either in the clinic or by telephone may be conducted to provide support and guidance to participants as needed.

6.7. Intervention after the End of the Study

Tirzepatide will not be made available to participants after conclusion of the study. Due to the double-blind study drug assignment, it is not known if a participant received active study drug or placebo. Participants will not be unblinded until study end and the final analyses are complete.

7. Discontinuation of Study Intervention and Participant Discontinuation/Withdrawal

7.1. Discontinuation of Study Intervention

7.1.1. Permanent Discontinuation from Study Drug

Before permanently discontinuing study drug, attempts to maintain the participant should be documented (see Dose Modification Section 6.6 and Temporary Dose Interruption, Section 6.6.2). Contact the sponsor before study treatment discontinuation occurs to discuss potential options to maintain the participant on study drug until final study ends. It is the goal for participants to remain on study drug treatment until study ends. It may be necessary for a participant to permanently discontinue study drug. If study drug is definitively discontinued, the participant will remain in the study and attend all scheduled visits to be evaluated for safety and efficacy and any further evaluations as described in the SoA.

Possible reasons leading to permanent discontinuation of study drug:

- **Participant decision**
  - The participant or the participant’s designee (for example, legal guardian) requests to discontinue study drug
- **Investigator Decision**
  - The investigator decides that the participant should be discontinued from study drug
- **Discontinuation due to a hepatic event or liver test abnormality**
  Participants who are interrupted/discontinued from study drug due to a hepatic event or liver test abnormality should have additional hepatic safety data collected via CRF.

  Interruption/discontinuation of the study drug for abnormal liver test results should be considered by the investigator when a participant meets one of the following conditions after consultation with the sponsor-designated medical monitor. These criteria differ based on the baseline liver test findings and are summarized below (Regev et al. 2019). If study drug is interrupted, it can be restarted only if another etiology is identified and liver enzymes return to baseline.

  For participants with baseline ALT <1.5X ULN and normal baseline bilirubin:

  - ALT or AST >8X ULN
  - ALT or AST >5X ULN for more than 2 weeks
  - ALT or AST >3X ULN and TBL >2X ULN or INR >1.5
  - ALT or AST >5X ULN with the appearance of fatigue, nausea, vomiting, right upper-quadrant pain or tenderness, fever, rash, and/or eosinophilia (>5%)
  - ALP >3X ULN
  - ALP >2.5X ULN and TBL >2X ULN

  - ALP >2.5X ULN with the appearance of fatigue, nausea, vomiting, right quadrant pain or tenderness, fever, rash, and/or eosinophilia (>5%)

For participants with baseline ALT ≥1.5X ULN and normal baseline bilirubin:

- ALT >5X baseline or ≥500 U/L (whichever occurs first)
- ALT >2X baseline or ≥300 U/L (whichever occurs first) and TBL >2X ULN
- ALT >3X baseline or ≥300 U/L (whichever occurs first) with the appearance of fatigue, nausea, vomiting, right upper-quadrant pain or tenderness, fever, rash, and/or eosinophilia (>5%)
- ALP >3X ULN
- ALP >2.5X ULN and TBL >2X ULN
- ALP >2.5X ULN with the appearance of fatigue, nausea, vomiting, right quadrant pain or tenderness, fever, rash, and/or eosinophilia (>5%)

- In addition, participants will be permanently discontinued from the study drug in the following circumstances: Investigators will have the discretion to leave participants on study medication, with the exception of pancreatic cancer.
  - acute or chronic pancreatitis (see Section 10.5.1.2)
  - if a participant is diagnosed with thyroid C-cell hyperplasia, MEN-2, or MTC, or pancreatic cancer after randomization
  - if any other TEAE, SAE, or clinically significant laboratory value for which the investigator believes that permanent study drug discontinuation is the appropriate measure to be taken
  - if a participant is diagnosed with T1DM
  - if a female participant becomes pregnant
  - if an investigator, site personnel performing assessments, or participant is unblinded, or
  - if the investigator, after consultation with the sponsor-designated medical resource, determines that a clinically significant systemic hypersensitivity reaction has occurred. A clinically significant systemic hypersensitivity reaction is one that occurs after administration of the study drug (for example, drug-related symptomatic bronchospasm, allergy-related edema/angioedema, or hypotension) and requires parenteral medication, does not respond to symptomatic medication, results in clinical sequelae, or is an anaphylactic reaction.
  - in the opinion of the investigator, the participant should permanently discontinue the study intervention for safety reasons

7.2. Participant Discontinuation/Withdrawal from the Study

In order to minimize the amount of missing data and to enable assessment of study objectives as planned in the study protocol, every attempt will be made to keep participants in the study, irrespective of the following:

- compliance to study drug
- adherence to visit schedule

- missing assessments
- study drug discontinuation due to AE (Section 7)
- development of comorbidities, and
- development of clinical outcomes.

The circumstances listed above are ***not*** valid reasons for participant discontinuation from the study.

Participant will be discontinued from study (early termination, ET) in the following circumstances:

- enrollment in any other clinical study involving a study drug or enrollment in any other type of medical research judged not to be scientifically or medically compatible with this study
- participation in the study needs to be stopped for medical, safety, regulatory, or other reasons consistent with applicable laws, regulations, and GCP, and
- if the participant becomes pregnant.

A participant may withdraw from the study:

- at any time at his/her own request, or
- at the request of his/her designee (for example, legal guardian).
- at the discretion of the investigator for safety, behavioral, compliance, or administrative reasons
- if enrolled in any other clinical study involving an investigational product, or enrolled in any other type of medical research judged not to be scientifically or medically compatible with this study
- if the participant, for any reason, requires treatment with a therapeutic agent that is prohibited by the protocol and has been demonstrated to be effective for treatment of the study indication. In this case, discontinuation from the study occurs prior to introduction of the new agent.

Study discontinuation is expected to be rare and participants should be provided with options for alternative follow-up methods and/or end of study vital status/endpoint ascertainment. At the time of discontinuing from the study, an ET visit should be conducted, as shown in the SoA. If the participant refuses to have an ET visit in the clinic, efforts should be made to collect data via telephone. See the SoA for data to be collected at the time of study discontinuation. If the participant has not already discontinued the study intervention, the participant will be permanently discontinued from the study intervention at the time of the decision to discontinue the study.

If the participant withdraws consent for disclosure of future information, the sponsor may retain and continue to use any data collected before such a withdrawal of consent. If a participant withdraws from the study, he/she may request destruction of any samples taken and not tested, and the investigator must document this in the site study records.

7.2.1. Inadvertently Enrolled Participants

If the sponsor or investigator identifies a participant who did not meet enrollment criteria and was inadvertently enrolled, then the participant should remain in the study and be discontinued from study drug when continued treatment would not be medically appropriate If the investigator and the sponsor CRP agree it is medically appropriate to continue the study drug, the investigator must obtain documented approval from the sponsor CRP to allow the inadvertently enrolled participant to continue study drug. Safety follow-up should be performed as outlined in Section 8.2 (Safety Assessments) and Section 8.3 (Adverse Events and Serious Adverse Events) of the protocol.

7.3. Lost to Follow up

A participant will be considered lost to follow-up if he or she repeatedly fails to return for scheduled visits and is unable to be contacted by the study site (≥2 consecutive visits). Site personnel or designee are expected to make diligent attempts to contact participants who fail to return for a scheduled visit or were otherwise unable to be followed up by the site.

Site personnel, or an independent third party, will attempt to collect the vital status of the participant within legal and ethical boundaries for all participants randomized, including those who did not get study drug. Public sources may be searched for vital status information. If vital status is determined to be deceased, this will be documented and the participant will not be considered lost to follow-up. Sponsor personnel will not be involved in any attempts to collect vital status information.

Discontinuation of specific sites or of the study as a whole are handled as part of Appendix 1 (Section 10.1)

8. Study Assessments and Procedures

Study procedures and their timing are summarized in the SoA.

Immediate safety concerns should be discussed with the sponsor immediately upon occurrence or awareness to determine if the participant should continue or discontinue study drug.

Adherence to the study design requirements, including those specified in the SoA, is essential and required for study conduct.

All screening evaluations must be completed and reviewed to confirm that potential participants meet all eligibility criteria. The investigator will maintain a screening log to record details of all participants screened and to confirm eligibility or record reasons for screening failure, as applicable.

Repeat or unscheduled samples may be taken for safety reasons or for technical issues with the samples.

Other visit methods (i.e. remote, telephone) may be considered at the discretion of the investigator if a participant is unable to come to their scheduled on-site visit. Alternative options to visit procedures must be considered with prior consultation and written approval of the sponsor.

8.1. Efficacy Assessments

8.1.1. Primary Efficacy Assessment

The primary efficacy endpoints are

- the hierarchal composite of time to all-cause mortality, occurrence of HF events through the end of the treatment period, the change from baseline in 6MWD, and the change from baseline in KCCQ-CSS at 52 weeks, and
- change from baseline in 6MWD at 52 weeks.

The primary efficacy assessments include

- time to all-cause mortality
- occurrence of HF events
- 6MWT, and
- KCCQ.

An independent CEC will adjudicate death and HF events. The CEC charter will contain the final detailed event definitions for all adjudicated events.

8.1.1.1. Time to All-Cause Mortality

All-cause mortality will be recorded by the investigator at the time of death.

8.1.1.2. Occurrence of Heart Failure Events

HF events will be recorded when the investigator becomes aware of any

- hospitalization for HF, or
- urgent HF visit.

8.1.1.3. Six-Minute Walk Test

Participants will perform an exercise capacity assessment using the 6MWT. Testing of the 6MWT should be performed as directed in the SoA (Section 1.3). The 6MWT is to be performed indoors on a straight, flat, hard surface that is at least 30 meters in length.

The 6MWTs at Visits 1 and 2 will be performed to assess participant eligibility. The Visit 1 tests will also serve as the training test to familiarize participants with the procedure. Additional details can be found in Section 10.10.1.

Prior to and at the end of each 6MWT, participants will be asked to rate their breathing discomfort and overall fatigue using the Borg Scale, separately, at each timepoint.

8.1.1.4. Kansas City Cardiomyopathy Questionnaire

The KCCQ is a 23-item, participant self-administered questionnaire that assesses impacts of HF "over the past 2 weeks" on the following 7 domains (Green et al. 2000; Joseph et al. 2013):

- Physical Limitation (6 items)
- Symptom Stability (1 item)
- Symptom Frequency (4 items)
- Symptom Burden (3 items)
- Self-Efficacy (2 items)
- Quality of Life (3 items), and
- Social Limitation (4 items).

Each of the 23 individual items are answered on Likert scales of varying lengths (5-point, 6-point, or 7-point scales). Domain scores are obtained by averaging the associated individual items and transforming the score to a 0 to 100 range. Higher scores indicate better health status. Summary scores are obtained by combining select domain scores:

- Total Symptom Score: mean of the Symptom Frequency and Symptom Burden scores
- Clinical Summary Score: mean of the Physical Limitation and Total Symptom scores, and
- Overall Summary Score: mean of the Physical Limitation, Total Symptom, Quality of Life, and Social Limitation scores.

The Clinical Summary Score will be used for the primary and key secondary endpoints.

8.1.2. Additional Secondary Efficacy Assessments

Body weight will be assessed as described in Section 8.2.1.

The NYHA classification will be assessed and recorded at the time points indicated in the SoA (Section 1.3) by an independent, blinded assessor. The NYHA classification is provided in Appendix 11 (Section 10.11).

8.1.3. Exploratory Efficacy Assessments

8.1.3.1. Patient-Reported Outcomes Assessments

The self-reported questionnaires will be translated into the native language of the region, linguistically validated, and administered according to the SoA (Section 1.3). At these visits, the questionnaires should be completed before the participant has discussed their medical condition or progress in the study with the investigator and/or site staff.

8.1.3.1.1. Patient Global Impression of Status – Overall

Study participants will be asked to complete a Patient Global Impression of Status – Overall item specifically developed for this study. This is a participant-rated assessment of their overall health "in the past 2 weeks" and is rated on a 5-point scale ranging from "1-Excellent" to "5-Poor."

8.1.3.1.2. Patient Global Impression of Status – Physical Function

Study participants will be asked to complete a Patient Global Impression of Status – Physical Function item specifically developed for this study. This is a participant-rated assessment of the overall impact of HF symptoms on their ability to perform physical activities "in the past 2 weeks" and is rated on a 5-point scale ranging from "1- Not impacted" to "5- Extremely impacted, cannot perform physical activities."

8.1.3.1.3. Patient Global Impression of Status – Symptom Severity

Study participants will be asked to complete a Patient Global Impression of Status – Symptom Severity item specifically developed for this study. This is a participant-rated assessment of the overall severity of their HF symptoms "in the past 2 weeks" and is rated on a 5-point scale ranging from "1- No symptoms" to "5- Very severe."

8.1.3.1.4. EQ-5D-5L

Generic health-related quality of life will be assessed using the EQ-5D-5L (EuroQoL Research Foundation 2019). The EQ-5D-5L is a standardized 5-item instrument for use as a measure of health outcome. It provides a simple descriptive profile and a single index value for health status that can be used in the clinical and economic evaluation of health care as well as population health surveys. The EQ-5D-5L comprises 5 dimensions of health (mobility, self-care, usual activities, pain/discomfort, and anxiety/depression). The 5L version, introduced in 2005, scores each dimension at 5 levels (no problems, slight problems, moderate problems, severe problems, and unable to perform/extreme problems), for a total of 3125 possible health states. In addition to the health profile, a single health state index value can be derived based on a formula that attaches weights to each of the levels in each dimension. This index value ranges between less than 0 (where 0 is a health state equivalent to death; negative values are valued as worse than dead) to 1 (perfect health). In addition, the EQ Visual Analog Scale records the respondent's self-rated health status on a vertical graduated (0 to 100) visual analog scale. In conjunction with the health state data, it provides a composite picture of the respondent's health status.

The EQ-5D-5L is used worldwide and is available in more than 170 languages. Details on the instrument, scoring, organizing, and presenting the data collected can be found in the EQ-5D-5L User Guide (EuroQoL Research Foundation 2019).

8.2. Safety Assessments

Planned time points for all safety assessments are provided in the SoA (Section 1.3).

8.2.1. Physical Examinations

- A complete physical examination will include, at a minimum, assessments of the CV, respiratory, GI and neurological systems, as well as a thyroid examination.
  - Body weight, waist circumference, and height should be measured. All weights for a given participant should be measured in a consistent manner using a calibrated scale (mechanical or digital scales are acceptable), using the same scale whenever possible, and after the participant has emptied their bladder. Participants should be lightly clothed but not wearing shoes while their weight is measured.
- Symptom-directed physical examinations will be conducted as described in the SoA.
  - Investigators should pay special attention to clinical signs and symptoms related to HF as well as related to previous serious illnesses. Particular interest would include dyspnea, orthopnea, paroxysmal nocturnal dyspnea, edema, jugular venous distension, and rales.

The physical examination should be performed before the first 6MWT, if more than one 6MWTs are done.

8.2.2. Vital Signs

For each participant, vital signs measurements should be conducted according to the SoA (Section 1.3). An apical heart rate should be assessed during the collection of vital signs. The vital signs collection associated with the 6MWT should be separate and may be performed using automated equipment.

Any clinically significant findings from vital signs measurements that result in a diagnosis and that occur after the participant receives the first dose of study drug should be reported to the sponsor or its designee as an AE via the eCRF.

8.2.3. Electrocardiograms

Single 12-lead ECGs will be obtained locally as outlined in the SoA (see Section 1.3).

All ECGs should be recorded after the participant has been supine for 5 minutes in a quiet room.

The ECGs must be interpreted by a qualified physician (the investigator or designee) at the site as soon after the time of ECG collection as possible, and ideally while the participant is still present, for immediate participant management, if needed. The investigator (or qualified designee) is responsible for determining if any change in participant management is needed, and must document his/her review of the ECG printed at the time of evaluation. If a clinically relevant abnormality is observed on the participant's ECG, then the investigator should assess the participant for symptoms (such as palpitations, near syncope, syncope, or chest pain). The investigator must report the presence of AF on the eCRF.

The original ECG must be retained at the investigative site.

The investigator or qualified designee's interpretation will prevail for immediate participant management purposes.

8.2.4. Clinical Safety Laboratory Assessments

With the exception of laboratory test results that may unblind the study, the sponsor or its designee will provide the investigator with the results of laboratory tests analyzed by a central vendor, if a central vendor is used for the clinical trial.

See Appendix 2 (Section 10.2) for the list of clinical laboratory tests to be performed. The SoA describes the timing and frequency.

The investigator must review the laboratory results, document this review, and report any clinically relevant changes occurring during the study as an AE. The laboratory results must be retained with source documents unless a Source Document Agreement or comparable document cites an electronic location that accommodates the expected retention duration. Clinically significant abnormal laboratory findings are those that are not associated with the underlying disease, unless judged by the investigator to be more severe than expected for the participant's condition.

All laboratory tests with values considered clinically significantly abnormal during participation in the study should be repeated until the values return to normal or baseline levels or are no longer considered clinically significant by the investigator or medical monitor.

- If such values do not return to normal/baseline levels within a period of time judged reasonable by the investigator, the etiology should be identified and the sponsor notified.
- All protocol-required laboratory assessments, as defined in Appendix 2 (Section 10.2), must be conducted in accordance with the SoA, standard collection requirements, and laboratory manual.

If laboratory values from non-protocol specified laboratory assessments performed at an investigator-designated local laboratory require a change in participant management or are considered clinically significant by the investigator (for example, SAE or AE or dose modification), then report the information as an AE.

All urine pregnancy tests will be performed locally according to the SoA. Guidance for contraception and definitions are defined in Appendix 4 (Section 10.4).

8.2.5. Safety Monitoring

The sponsor will periodically review evolving aggregate safety data within the study by appropriate methods. The study team will review safety reports in a blinded fashion according to the schedule provided in the Trial-Level Safety Review Plan. The sponsor will also review SAEs within time frames mandated by company procedures. The Sponsor CRP will, as appropriate, consult with the functionally independent Global Patient Safety therapeutic area physician or clinical scientist.

8.2.5.1. Hepatic Safety Monitoring

Close Hepatic Monitoring

Laboratory tests (Appendix 8 [Section 10.8]), including ALT, AST, ALP, TBL, D Bil, GGT, and creatine kinase, should be repeated within 48 to 72 hours to confirm the abnormality and to determine if it is increasing or decreasing, if one or more of these conditions occur:

If a participant with baseline results of …	develops the following elevations:
ALT or AST <1.5X ULN	ALT or AST ≥3X ULN
ALP <1.5X ULN	ALP ≥2X ULN
TBL <1.5X ULN	TBL ≥2X ULN (except for participants with Gilbert's syndrome)
ALT or AST ≥1.5X ULN	ALT or AST ≥2X baseline
ALP ≥1.5X ULN	ALP ≥2X baseline
TBL ≥1.5x ULN	TBL ≥1.5X baseline (except for participants with Gilbert's syndrome)

Abbreviations: ALP = alkaline phosphatase; ALT = alanine aminotransferase; AST = aspartate aminotransferase; TBL = total bilirubin; ULN = upper limit of normal.

If the abnormality persists or worsens, clinical and laboratory monitoring and evaluation for possible causes of abnormal liver tests should be initiated by the investigator in consultation with the sponsor-designated medical monitor. At a minimum, this evaluation should include physical examination and a thorough medical history, including symptoms, recent illnesses (for example, HF, systemic infection, hypotension, or seizures), recent travel, history of concomitant medications (including over-the-counter), herbal and dietary supplements, history of alcohol use, and other substance abuse.

Initially, monitoring of symptoms and hepatic biochemical tests should be done at a frequency of 1 to 3 times weekly, based on the participant's clinical condition and hepatic biochemical tests. Subsequently, the frequency of monitoring may be lowered to once every 1 to 2 weeks, if the participant's clinical condition and laboratory results stabilize. Monitoring of ALT, AST, ALP, and TBL should continue until levels normalize or return to approximate baseline levels.

Comprehensive Hepatic Evaluation

A comprehensive evaluation should be performed to search for possible causes of liver injury if one or more of these conditions occur:

If a participant with baseline results of …	develops the following elevations:
ALT or AST <1.5X ULN	ALT or AST ≥3X ULN with hepatic signs/symptoms* OR<br>ALT or AST ≥5X ULN
ALP <1.5X ULN	ALP ≥3X ULN

TBL <1.5X ULN	TBL ≥2X ULN (except for participants with Gilbert's syndrome)
ALT or AST ≥1.5X ULN	ALT or AST ≥2X baseline with hepatic signs/symptoms* OR<br>ALT or AST ≥3X baseline
ALP ≥1.5X ULN	ALP ≥2X baseline
TBL ≥1.5X ULN	TBL ≥2X baseline (except for participants with Gilbert's syndrome)

Abbreviations: ALP = alkaline phosphatase; ALT = alanine aminotransferase; AST = aspartate aminotransferase; TBL = total bilirubin; ULN = upper limit of normal.

* Hepatic signs/symptoms are severe fatigue, nausea, vomiting, right upper quadrant abdominal pain, jaundice, fever, rash, and/or eosinophilia >5%.

At a minimum, this evaluation should include physical examination and a thorough medical history, as outlined above, as well as tests for PT-INR; tests for viral hepatitis A, B, C, or E; tests for autoimmune hepatitis; and an abdominal imaging study (for example, ultrasound or CT scan).

Based on the participant's history and initial results, further testing should be considered in consultation with the sponsor-designated medical monitor, including tests for hepatitis D virus (HDV), cytomegalovirus (CMV), Epstein-Barr virus (EBV), acetaminophen levels, acetaminophen protein adducts, urine toxicology screen, Wilson's disease, blood alcohol levels, urinary ethyl glucuronide, and blood phosphatidylethanol (see Appendix 8 [Section 10.8]). Based on the circumstances and the investigator's assessment of the participant's clinical condition, the investigator should consider referring the participant for a hepatologist or gastroenterologist consultation, magnetic resonance cholangiopancreatography (MRCP), endoscopic retrograde cholangiopancreatography (ERCP), cardiac echocardiogram, or a liver biopsy.

Additional hepatic data collection (hepatic safety CRF) in study participants who have abnormal liver tests during the study

Additional hepatic safety data collection in hepatic safety CRFs should be performed in study participants who meet 1 or more of the following 5 conditions:

1. Elevation of serum ALT to ≥5X ULN on 2 or more consecutive blood tests (if baseline ALT <1.5X ULN)
   - In participants with baseline ALT ≥1.5X ULN, the threshold is ALT ≥3X baseline on 2 or more consecutive tests
2. Elevated TBL to ≥2X ULN (if baseline TBL <1.5X ULN) (except for cases of known Gilbert's syndrome)
   - In participants with baseline TBL ≥1.5X ULN, the threshold should be TBL ≥2X baseline
3. Elevation of serum ALP to ≥2X ULN on 2 or more consecutive blood tests (if baseline ALP <1.5X ULN)

- In participants with baseline ALP ≥1.5X ULN, the threshold is ALP ≥2X baseline on 2 or more consecutive blood tests

4. Hepatic event considered to be an SAE
5. Discontinuation of study drug due to a hepatic event

Note: the interval between the two consecutive blood tests should be at least 2 days.

8.3. Adverse Events, Serious Adverse Events, and Product Complaints

The definitions of the following events can be found in Appendix 3 (Section 10.3):

- Adverse events (AEs)
- Serious adverse events (SAEs)
- Product complaints (PCs)

These events will be reported by the participant (or, when appropriate, by a caregiver, surrogate, or the participant's legally authorized representative).

The investigator and any qualified designees are responsible for detecting, documenting, and recording events that meet these definitions and remain responsible for following up events that are serious, considered related to the study drug or study procedures, or that caused the participant to discontinue the study drug or study (see Section 7).

Care will be taken not to introduce bias when detecting events. Open-ended and non-leading verbal questioning of the participant is the preferred method to inquire about event occurrences.

After the initial report, the investigator is required to proactively follow each participant at subsequent visits/contacts. All SAEs will be followed until resolution, stabilization, the event is otherwise explained, or the participant is lost to follow-up (as defined in Section 7.3).

For product complaints, the investigator is responsible for ensuring that follow-up includes any supplemental investigations as indicated to elucidate the nature and/or causality. Further information on follow-up procedures is provided in Appendix 3 (Section 10.3).

Suspected Unexpected Serious Adverse Reactions

Suspected unexpected serious adverse reactions (SUSARs) are serious events that are not listed in the IB and that the investigator identifies as related to study drug or procedure. United States 21 CFR 312.32, European Union Clinical Trial Directive 2001/20/EC, and the associated detailed guidance or national regulatory requirements in participating countries require the reporting of SUSARs. The sponsor has procedures that will be followed for the identification, recording, and expedited reporting of SUSARs that are consistent with global regulations and the associated detailed guidance.

8.3.1. Timing and Mechanism for Collecting Events

This table describes the timing, deadlines, and mechanism for collecting events.

Event	Collection Start	Collection Stop	Timing for Reporting to Sponsor or Designee	Mechanism for Reporting	Back-up Method of Reporting
**Adverse Event**					
AE	Signing of the informed consent form (ICF)	Participation in study has ended	As soon as possible upon site awareness	AE eCRF	N/A
**Serious Adverse Event**					
SAE and SAE updates – prior to start of study drug **and** deemed reasonably possibly related with study procedures	Signing of the informed consent form (ICF)	Start of study drug	Within 24 hours of awareness	SAE eCRF	SAE paper form
SAE* and SAE updates – after start of study drug	Start of study drug	Participation in study has ended	Within 24 hours of awareness	SAE eCRF	SAE paper form

Event	Collection Start	Collection Stop	Timing for Reporting to Sponsor or Designee	Mechanism for Reporting	Back-up Method of Reporting
SAE* – after participant's study participation has ended **and** the investigator becomes aware	After participant's study participation has ended	N/A	Promptly	SAE paper form	N/A
**Pregnancy**					
Pregnancy in female participants and female partners of male participants	After the start of study drug	Four months after the last injection for female partners of male participants and 2 months after the last injection for female participants	Within 24 hours of learning of the pregnancy	SAE eCRF	SAE paper form
**Product Complaints**					
PC associated with an SAE or might have led to an SAE	Start of study drug	End of study drug	Within 24 hours of awareness	Product Complaint form	N/A
PC not associated with an SAE	Start of study drug	End of study drug	Within 1 business day of awareness	Product Complaint form	N/A

Event	Collection Start	Collection Stop	Timing for Reporting to Sponsor or Designee	Mechanism for Reporting	Back-up Method of Reporting
Updated PC information	—	—	As soon as possible upon site awareness	Originally completed Product Complaint form with all changes signed and dated by the investigator	N/A
PC (if investigator becomes aware)	Participation in study has ended	N/A	Promptly	Product Complaint form	N/A

Abbreviations: eCRF = electronic case report form; N/A = not applicable; PC = product complaint; SAE = serious adverse event.

*Serious adverse events, including death, caused by disease progression as described in Section 8.3.2 should not be reported unless the investigator deems them to be possibly related to study drug.

8.3.2. Primary, Secondary, and Additional Study Endpoint Reporting

The following investigator-reported events are considered potential endpoints and must be reported first as an AE on the AE eCRF (with the appropriate designation for seriousness). They must then be reported as an endpoint on the eCRF with all required source documents provided for adjudication to the CEC. These potential endpoints (even if they meet criteria for a serious event) are not to be reported on the SAE eCRF unless considered as possibly related to study drug, the drug delivery system, or study procedure. Potential endpoints that are serious and considered as possibly related to study drug, the drug delivery system, or study procedure must also be reported as an SAE using the SAE eCRF:

- all-cause mortality (death), and
- hospitalization for HF or an urgent HF visit.

In the case where 1 of the above endpoint events is reported but does not meet a prespecified event definition detailed in the CEC charter, as reviewed by the independent CEC, no further action will be taken by the study site.

8.3.3. Adverse Events of Special Interest

The following are AESI and will be adjudicated by an external adjudication committee. This committee will be blinded to treatment assignment.

- pancreatitis

- major adverse CV events (see Section 10.5.1.5), and
- deaths

The following are additional AESI for this program that will not be adjudicated by an external committee:

- hepatobiliary disorders
- severe hypoglycemia
- thyroid malignancies and C-cell hyperplasia
- supraventricular arrhythmias and cardiac conduction disorders
- allergic/hypersensitivity reactions, including injection site reactions and ADA formation
- severe GI AEs, and
- acute renal events.

Sites should collect additional details and data regarding AESI, as instructed on the applicable eCRFs, and detailed in Section 10.5.

The details on the definition of AESI will be provided in SAP.

8.4. Treatment of Overdose

Considering the mechanism of action of tirzepatide, potential overdose effects can be GI disorders and hypoglycemia. In the event of overdose, appropriate supportive treatment should be initiated according to the participant's clinical signs and symptoms. In the event of overdose, refer to the IB for tirzepatide.

8.5. Pharmacokinetics

Pharmacokinetic samples will be collected from all participants in this study.

Tirzepatide plasma concentrations will be determined from blood samples obtained from participants receiving tirzepatide treatment. Blood samples collected from participants assigned to the placebo arm will not be included in the bioanalysis of drug concentrations.

Blood samples for PK assessment will be collected prior to the dose administration and at the same time as the planned immunogenicity samples (that is, at Week 0 and then at Weeks 4, 12, 24, and 52 per the Study Schedule or additionally at follow-up and ET (reference SoA).

Samples will be analyzed at a laboratory approved by the sponsor and stored at a facility designated by the sponsor.

Concentrations of tirzepatide will be assayed using a validated liquid chromatography mass spectrometry method. Bioanalytical samples collected to measure tirzepatide concentrations will be retained for a maximum of 1 year following last participant visit for the study (Section 10.1.10). During this time, samples remaining after the bioanalyses may be used for exploratory analyses such as metabolism work, protein binding, and/or bioanalytical method cross-validation.

8.6. Pharmacodynamics

Pharmacodynamic parameters will not be evaluated in this study.

8.7. Genetics

A whole blood sample will be collected for pharmacogenetic analysis where local regulations allow.

See Appendix 2, Clinical Laboratory Tests (Section 10.2), and Section 1.3 (SoA) for sample collection information.

See Section 10.6 for genetic research, custody, and sample retention information.

8.8. Biomarkers

Biomarker research is performed to address questions of relevance to drug disposition, target engagement, pharmacodynamics, mechanism of action, variability of participant response (including safety), and clinical outcome. Sample collection is incorporated into clinical studies to enable examination of these questions through measurement of biomolecules including DNA, RNA, proteins, lipids, and other cellular elements.

Serum and plasma for exploratory biomarker research will be collected at the time specified in the SoA (Section 1.3) where local regulations allow.

All samples will be coded with the participant number. These samples and any data generated can be linked back to the participant only by the investigator site personnel.

Samples will be stored and analysis may be performed on biomarkers thought to play a role in disease processes, mechanism of action of tirzepatide, pathways associated with HFpEF, and/or research methods validating diagnostic tools or assay(s) related to HFpEF and associated diseases. Biomarkers may be evaluated to determine their association with observed clinical responses to tirzepatide and the disease state.

Samples will be retained at a facility selected by the sponsor or its designee for the duration detailed in Section 10.1.10, or for a shorter period if local regulations and ERBs impose shorter time limits. This retention period enables use of new technologies, response to regulatory questions, and investigation of variable response that may not be observed until later in the development of tirzepatide or after tirzepatide becomes commercially available.

8.9. Immunogenicity Assessments

At the visits and times specified in the SoA (Section 1.3), venous blood samples will be collected to determine antibody production against tirzepatide. Antibodies may be further characterized for cross-reactive binding to endogenous counterparts (native GIP and GLP-1) and their ability to neutralize the activity of tirzepatide and endogenous counterparts. To interpret the results of immunogenicity, a venous blood sample will be collected at the same time points to determine the plasma concentrations of tirzepatide. All samples for immunogenicity should be taken predose when applicable and possible.

Samples will be retained for a maximum of 15 years after the last participant visit, or for a shorter period if local regulations and ERBs allow, at a facility selected by the sponsor. The duration allows the sponsor to respond to future regulatory requests related to tirzepatide. Any samples remaining after 15 years will be destroyed.

8.10. Medical Resource Utilization and Health Economics

Not applicable.

9. Statistical Considerations

9.1. Statistical Hypotheses

Two primary hypotheses will be tested in this study:

- Tirzepatide MTD is superior to placebo for the hierarchical composite endpoint of all-cause mortality, occurrence of adjudicated HF events (including HF hospitalization or urgent HF visit) through the end of the treatment period, the change from baseline in 6MWD, and the change from baseline in KCCQ-CSS at Week 52.
- Tirzepatide MTD is superior to placebo for the change from baseline in 6MWD at Week 52.

Key secondary hypotheses (all under multiplicity control) are that tirzepatide MTD is superior to placebo with regards to

- percent change from baseline in body weight at Week 52
- change from baseline in KCCQ-CSS at Week 52
- change from baseline in 6MWD at Week 24, and
- proportion of participants with NYHA class change at Week 52.

All primary and key secondary hypotheses will be tested with the overall family-wise type I error rate at a 2-sided alpha level of 0.05 through the multiplicity control approach based on the graphical multiple testing procedure. For the 2 primary hypotheses, the hierarchical composite endpoint will be tested at a 2-sided alpha level of 0.04, and change in 6MWD will be tested at a 2-sided alpha level of 0.01 in parallel for statistical significance. If either is significant, the corresponding assigned alpha will be propagated to test the other primary efficacy endpoint at a 2-side alpha level of 0.05. The detailed graphical testing scheme will be outlined in the SAP.

9.2. Sample Size Determination

Approximately 700 participants will be randomized to either tirzepatide or placebo in a 1:1 ratio (approximately 350 participants per treatment arm), and the statistical powers are evaluated for each of the 2 primary efficacy endpoints at a 2-sided significance level of 0.01 to ensure that the sample size and power are sufficient for registration purposes. This sample size will provide

- approximately 85% power or higher to demonstrate superiority of tirzepatide MTD to placebo for the hierarchical composite endpoint using Finkelstein-Schoenfeld method (Finkelstein and Schoenfeld 1999)
- approximately 95% power for the change from baseline in 6MWD using Wilcoxon rank sum test, and
- more than 95% power to demonstrate superiority for at least one of above.

The sample size and power are estimated through simulations under the following assumptions:

Variable	Placebo	Tirzepatide MTD	Treatment Difference
All-cause mortality<br>(n per 100 yr)	5	5 or 4.75	0 or -0.25<br>(0 or 5% RRR)
First HF events<br>(n per 100 yr)	10	8 or 7	-2 or -3<br>(20% or 30% RRR)
6MWD – change from baseline (mean±SD)	0±75	30±85	30
KCCQ-CSS – change from baseline (mean±SD)	5±19	10±19	5

Abbreviations: 6MWD = 6-minute walk test distance; HF = heart failure; KCCQ-CSS = Kansas City Cardiomyopathy Questionnaire – Clinical Summary Scale; MTD = maximum tolerated dose; n = number; RRR = relative risk reduction; SD = standard deviation.

In addition, the following imputation rules for 6MWD and KCCQ-CSS at Week 52 are also built into the simulations: worst case imputation (assign 0 steps or a score of 0) for missing due to death (assume 5% death rate) and placebo imputation for 10% participants who discontinue study treatment due to AE.

9.3. Populations for Analyses

The following populations are defined:

Analysis Population	Description
Entered	All participants who sign the informed consent form
Randomized/Intent-to-Treat (ITT) Population	All participants assigned to treatment, regardless of whether they take any doses of study treatment, or if they took the correct treatment. Participants will be analyzed according to the treatment group to which they were assigned.
Safety Population	All participants in ITT population who take at least 1 dose of study treatment. Participants will be analyzed according to the treatment group to which they were assigned.

9.4. Statistical Analyses

9.4.1. General Considerations

Statistical analysis of this study will be the responsibility of sponsor or its designee.

Unless specified otherwise, all tests of treatment effects will be conducted at a 2-sided alpha level of 0.05 and all confidence intervals will be given at a 2-sided 95% level. Efficacy will be assessed using ITT Population and safety will be assessed using the Safety Population.

Any change to the data analysis methods described in the protocol will require an amendment only if it changes a principal feature of the protocol. Any other change to the data analysis methods described in the protocol, and the justification for making the change, will be described in the SAP and the CSR. Additional exploratory analyses of the data will be conducted as deemed appropriate.

The SAP will be completed prior to first unblinding and any subsequent amendments will be documented, with final amendments finalized prior to final database lock. The SAP will include a more technical and detailed description of the statistical analyses described in this section. This section is a summary of the planned statistical analyses of the most important endpoints including primary and key secondary endpoints.

9.4.2. Primary Endpoint(s)

The primary estimand for primary endpoints is to assess the treatment difference between tirzepatide and placebo relative to the efficacy measures for all randomized participants, and treatment policy strategy will be used to handle all intercurrent events, which is, all the observed values for the variable of interest are used regardless of whether the intercurrent event occurs. The endpoint and population-level summary for the estimand is described in Section 9.4.2.1 and Section 9.4.2.2 for each primary endpoint.

9.4.2.1. The Hierarchical Composite Endpoint

The analysis of the primary hierarchical composite endpoint will be performed with the Finkelstein-Schoenfeld method, and the win ratio (Pocock et al. 2012) will be reported as the measure of treatment effect. The population-level summary of win ratio will be calculated as number of pairs of tirzepatide-treated participant "wins" divided by number of pairs of placebo-treated participant "wins."

The Finkelstein-Schoenfeld method is based on the principle that each participant is compared with every other participant within each stratum in a pairwise manner that proceeds in a hierarchical fashion. Participants will be stratified according to HF decompensation within 12 months of screening (Y/N), diagnosed T2DM (Y/N), and baseline BMI $\geq$35 kg/m^2 (Y/N), yielding 8 stratification pools. For the primary composite endpoint, each pairwise comparison will proceed in the following order and a winner has:

- A delayed first occurrence of all-cause death;
- If the pair cannot be differentiated based on mortality, a winner has fewer HF events;
- If the pair cannot be differentiated by number of HF events, a winner has delayed time to the occurrence of first HF event;
- If the pair still cannot be differentiated, a winner has a more favorable category for change from baseline in 6MWD at Week 52;
- If the pair still cannot be differentiated, a winner has a more favorable category for change from baseline in KCCQ-CSS at Week 52;

- Otherwise the pair will be recorded as tied.

The categories for change from baseline in 6MWD are: 1) ≥30% worsening; 2) ≥20% and <30% worsening; 3) ≥10% and <20% worsening; 4) No change (less than 10% change); 5) ≥10% and <20% improvement; 6) ≥20% and <30% improvement; and 7) ≥30% improvement.

The categories for change from baseline in KCCQ-CSS are: 1) ≥10-point worsening; 2) ≥5- but <10-point worsening; 3) No change (<5-point change); 4) ≥5- but <10-point improvement; 5) ≥10-point improvement.

In the pairwise comparison for all-cause mortality and HF events, the censoring for death and HF events will be handled based on the win ratio method (Pocock et al. 2012). When 2 participants have different follow-up times, the shorter follow-up time will be used to compare the clinical outcome measure. Only adjudicated and confirmed endpoint events are included in the primary analysis.

The last measurement prior to randomization for 6MWD and KCCQ-CSS will be used as baseline. Missing 6MWD and KCCQ-CSS values at Week 52 will be imputed through multiple imputations based on the reason of missingness with details described in the SAP; the statistical inference over multiple imputations will be guided by the method proposed by Rubin (1987).

9.4.2.2. Change from Baseline in 6-Minute Walk Test Distance

For the primary endpoint of change from baseline to Week 52 in 6MWD, a stratified Wilcoxon (Van Elteren) test will be used as the primary analysis method, controlling for the stratification factors of HF decompensation within 12 months of screening (Y/N), diagnosed T2DM (Y/N), and baseline BMI ≥35 kg/m^2 (Y/N). Population-level summary of Hodges-Lehmann estimate for the median difference and corresponding confidence interval will be reported.

Missing 6MWD at Week 52 will be imputed through multiple imputations based on the reason of missingness with details described in the SAP. The statistical inference over multiple imputations will be guided by the method proposed by Rubin (1987).

9.4.3. Key Secondary Endpoint(s)

Analyses for the key secondary endpoints will also be guided by the treatment policy strategy.

Change from baseline in KCCQ-CSS at Week 52 and change from baseline in 6MWD at Week 24 will be analyzed using the same nonparametric approach as described in Section 9.4.2.2.

Percent change from baseline in body weight will be analyzed using an MMRM analysis. The MMRM model will include the categorical effect of treatment, time, treatment-by-time interaction, stratification factors, and the continuous covariate of baseline body weight value. Missing data will be imputed through multiple imputations based on the reason of missingness with details described in the SAP.

Change in NYHA class (improved, no change, or worsened) from baseline will be analyzed using a longitudinal proportional odds model. Missing data will be imputed based on the reason of missingness as described in the SAP.

9.4.4. Tertiary/Exploratory Endpoint(s)

The analyses for exploratory endpoints will be described in the SAP. Statistical tests will be performed at the two-sided significance level of 0.05. There will be no multiplicity adjustment for any analysis of exploratory variables unless specified otherwise. Missing values will not be explicitly imputed unless specified otherwise.

9.4.5. Other Safety Analyses

Safety will be assessed by summarizing and analyzing AEs, special safety topics, laboratory analytes, and vital signs. All safety analyses will be made on the Safety Population. Unless specified otherwise, all data obtained during study period from Safety Population, regardless of adherence to study drug, will be used for safety analyses. The details for safety analysis will be described in the SAP.

Adverse events will be coded from the actual term using MedDRA and reported with preferred terms and system organ class.

9.4.5.1. Evaluation of Immunogenicity

The frequency and percentage of participants with preexisting ADAs and with treatment-emergent ADAs to tirzepatide will be tabulated. Treatment-emergent ADAs are defined as those with a titer 2-fold (1 dilution) greater than the minimum required dilution of the ADA assay if no ADAs were detected at baseline (treatment-induced ADA), or those with a 4-fold (2 dilutions) increase in titer compared with baseline if ADAs were detected at baseline (treatment-boosted ADA). The details of analyses for immunogenicity will be specified in SAP.

9.4.6. Subgroup Analyses

Subgroup variables to be evaluated for the primary efficacy endpoint may include demography (for example, race, ethnicity), baseline disease characteristics (for example, diagnosed T2DM) and others. Subgroup analyses may also be performed for selected key secondary efficacy endpoints. Details for the subgroup analyses will be provided in the SAP.

9.5. Interim Analyses

Based on the projected enrollment, approximately 4 interim analyses of safety will be conducted. The first interim analysis is planned to occur when approximately 20% of the anticipated number of participants are randomized or 6 months after the first participant is randomized, whichever occurs later, followed by subsequent reviews approximately every 6 months throughout the study.

The DMC is authorized to evaluate unblinded interim analyses. Study sites will receive information about interim results only if they need to know for the safety of their participants.

Unblinding details are specified in a separate unblinding plan document.

The DMC charter will describe the planned interim analyses in detail.

9.6. Data Monitoring Committee

An independent DMC with members all external to the sponsor will be used to monitor participant safety in an unblinded fashion. For details on the DMC, refer to the DMC charter.

10. Supporting Documentation and Operational Considerations

10.1. Appendix 1: Regulatory, Ethical, and Study Oversight Considerations

10.1.1. Regulatory and Ethical Considerations

- This study will be conducted in accordance with the protocol and with the following:
  - Consensus ethical principles derived from international guidelines including the Declaration of Helsinki and CIOMS International Ethical Guidelines
  - Applicable ICH GCP guidelines
  - International Organization for Standardization (ISO) 14155
  - Applicable laws and regulations
- The protocol, protocol amendments, ICF, IB, and other relevant documents (for example, advertisements) must be submitted to an IRB/IEC by the investigator and reviewed and approved by the IRB/IEC before the study is initiated.
- Any amendments to the protocol will require IRB/IEC approval before implementation of changes made to the study design, except for changes necessary to eliminate an immediate hazard to study participants.
- Protocols and any substantial amendments to the protocol will require health authority approval prior to initiation except for changes necessary to eliminate an immediate hazard to study participants.
- The investigator will be responsible for the following:
  - Providing written summaries of the status of the study to the IRB/IEC annually or more frequently in accordance with the requirements, policies, and procedures established by the IRB/IEC
  - Notifying the IRB/IEC of SAEs or other significant safety findings as required by IRB/IEC procedures
  - Providing oversight of study conduct for participants under their responsibility and adherence to requirements of 21 CFR, ICH guidelines, the IRB/IEC, European regulation 536/2014 for clinical studies (if applicable), and all other applicable local regulations
- Investigator sites are compensated for participation in the study as detailed in the clinical trial agreement.

10.1.2. Financial Disclosure

Investigators and sub-investigators will provide the sponsor with sufficient, accurate financial information as requested to allow the sponsor to submit complete and accurate financial certification or disclosure statements to the appropriate regulatory authorities. Investigators are

responsible for providing information on financial interests during the course of the study and for 1 year after completion of the study.

10.1.3. Informed Consent Process

The investigator or his/her representative will explain the nature of the study, including the risks and benefits, to the participant or his/her legally authorized representative and answer all questions regarding the study.

Participants must be informed that their participation is voluntary. Participants or their legally authorized representative will be required to sign a statement of informed consent that meets the requirements of 21 CFR 50, local regulations, ICH guidelines, HIPAA requirements, where applicable, and the IRB/IEC or study center.

The medical record must include a statement that written informed consent was obtained before the participant was entered in the study and the date the written consent was obtained. The authorized person obtaining the informed consent must also sign the ICF.

Participants must be re-consented to the most current version of the ICF(s) during their participation in the study.

A copy of the ICF(s) must be provided to the participant or the participant's legally authorized representative and is kept on file.

Participants who are rescreened are required to sign a new ICF.

10.1.4. Data Protection

Participants will be assigned a unique identifier by the sponsor. Any participant records, datasets, or tissue samples that are transferred to the sponsor will contain the identifier only; participant names or any information which would make the participant identifiable will not be transferred.

The participant must be informed that the participant's personal, study-related data will be used by the sponsor in accordance with local data protection law. The level of disclosure must also be explained to the participant who will be required to give consent for his/her data to be used as described in the informed consent.

The participant must be informed that their medical records may be examined by Clinical Quality Assurance auditors or other authorized personnel appointed by the sponsor, by appropriate IRB/IEC members, and by inspectors from regulatory authorities.

The sponsor has processes in place to ensure data protection, information security, and data integrity. These processes include appropriate contingency plan(s) for appropriate and timely response in the event of a data security breach.

10.1.5. Dissemination of Clinical Study Data

Report Preparation

An investigator will sign the final CSR for this study, indicating agreement that, to the best of his or her knowledge, the report accurately describes the conduct and results of the study.

Public Access to Reports and Data

Reports

The sponsor will disclose a summary of study information, including tabular study results, on publicly available websites where required by local law or regulation.

Data

The sponsor provides access to all individual participant data collected during the trial, after anonymization, with the exception of PK, immunogenicity, or genetic data. Data are available to request 6 months after the indication studied has been approved in the United States and after primary publication acceptance, whichever is later. No expiration date of data requests is currently set once they are made available. Access is provided after a proposal has been approved by an independent review committee identified for this purpose and after receipt of a signed data sharing agreement. Data and documents, including the study protocol, SAP, CSR, and blank or annotated CRFs, will be provided in a secure data sharing environment for up to 2 years per proposal. For details on submitting a request, see the instructions provided at www.clinicalstudydatarequest.com.

Publications/Publication Policy

The publication policy is described in Section 10.1.9.

10.1.6. Data Quality Assurance

All participant data relating to the study will be recorded on printed or electronic CRF unless transmitted to the sponsor or designee electronically (for example, laboratory data or an electronic source, such as eCOA). The investigator is responsible for verifying that data entries are accurate and correct by physically or electronically signing the CRF.

The investigator must maintain accurate documentation (source data) that supports the information entered in the CRF.

The investigator must permit study-related monitoring, audits, IRB/IEC review, and regulatory agency inspections and provide direct access to source data documents.

Monitoring details describing strategy (for example, risk-based initiatives in operations and quality such as risk management and mitigation strategies and analytical risk-based monitoring), methods, responsibilities, and requirements, including handling of noncompliance issues and monitoring techniques, are provided in the Monitoring Plan.

The sponsor or designee is responsible for the data management of this study including quality checking of the data.

The sponsor assumes accountability for actions delegated to other individuals (for example, contract research organizations).

Study monitors will perform ongoing source data verification to confirm that data transcribed into the CRF by authorized site personnel are accurate, complete, and verifiable from source documents; that the safety and rights of participants are being protected; and that the study is being conducted in accordance with the currently approved protocol and any other study agreements, ICH GCP, and all applicable regulatory requirements.

Records and documents, including signed ICFs, pertaining to the conduct of this study must be retained by the investigator for the time period outlined in the clinical trial agreement unless local regulations or institutional policies require a longer retention period. No records may be destroyed during the retention period without the written approval of the sponsor. No records may be transferred to another location or party without written notification to the sponsor.

In addition, the sponsor or its representatives will periodically check a sample of the participant data recorded against source documents at the study site. The study may be audited by the sponsor or its representatives, and/or regulatory agencies at any time. Investigators will be given notice before an audit occurs.

Data Capture System

The investigator is responsible for ensuring the accuracy, completeness, legibility, and timeliness of the data reported to the sponsor.

An EDC system will be used in this study for the collection of CRF data. The investigator maintains a separate source for the data entered by the investigator or designee into the sponsor-provided EDC system. The investigator is responsible for the identification of any data to be considered source and for the confirmation that data reported are accurate and complete by signing the CRF.

Additionally, eCOA data (patient-reported outcomes instruments) will be directly recorded by the participant, into a device (for example, hand-held smart phone or tablet). The eCOA data will serve as the source documentation, and the investigator does not maintain a separate, written or electronic record of these data.

Data collected via the sponsor-provided data capture systems will be stored at third parties. The investigator will have continuous access to the data during the study and until decommissioning of the data capture system(s). Prior to decommissioning, the investigator will receive an archival copy of pertinent data for retention.

Data managed by a central vendor, such as laboratory test data, will be stored electronically in the central vendor's database system, and reports (as applicable) will be provided to the investigator for review and retention. Data will subsequently be transferred from the central vendor to the sponsor data warehouse.

Data from complaint forms submitted to the sponsor will be encoded and stored in the global product complaint management system.

10.1.7. Source Documents

Source documents provide evidence for the existence of the participant and substantiate the integrity of the data collected. Source documents are filed at the investigator's site.

Data reported on the CRF or entered in the eCRF that are transcribed from source documents must be consistent with the source documents, or the discrepancies must be explained. The investigator may need to request previous medical records or transfer records, depending on the study. Also, current medical records must be available.

Definition of what constitutes source data can be found in Section 10.1.6.

10.1.8. Study and Site Start and Closure

The study start date is the date on which the clinical study will be open for recruitment of participants.

The first act of recruitment is the first site open and will be the study start date.

The sponsor designee reserves the right to close the study site or terminate the study at any time for any reason at the sole discretion of the sponsor. Study sites will be closed upon study completion. A study site is considered closed when all required documents and study supplies have been collected and a study-site closure visit has been performed.

The investigator may initiate study-site closure at any time, provided there is reasonable cause and sufficient notice is given in advance of the intended termination.

Reasons for the early closure of a study site by the sponsor or investigator may include but are not limited to:

- Failure of the investigator to comply with the protocol, the requirements of the IRB/IEC or local health authorities, the sponsor's procedures, or GCP guidelines
- Inadequate recruitment of participants by the investigator
- Discontinuation of further study drug development.

If the study is prematurely terminated or suspended, the sponsor shall promptly inform the investigators, the IECs/IRBs, the regulatory authorities, and any contract research organization(s) used in the study of the reason for termination or suspension, as specified by the applicable regulatory requirements. The investigator shall promptly inform the participant and assures appropriate participant therapy and/or follow-up.

10.1.9. Publication Policy

In accordance with the sponsor's publication policy, the results of this study will be submitted for publication by a peer-reviewed journal.

10.1.10. Long-Term Sample Retention

Sample retention enables use of new technologies, response to regulatory questions, and investigation of variable response that may not be observed until later in the development of the intervention or after the intervention becomes commercially available.

This table describes the retention period for potential sample types.

Sample Type	Custodian	Retention Period After Last Participant Visit
Genetics sample	Sponsor or designee	up to 15 years
Exploratory biomarker sample	Sponsor or designee	up to15 years
Immunogenicity (antidrug antibody) sample	Sponsor or designee	up to 15 years
Pharmacokinetic sample	Sponsor or designee	up to 1 years

10.1.11. Investigator Information

Researchers with appropriate education, training, and experience, as determined by the Sponsor, will participate as investigators in this clinical trial

10.2. Appendix 2: Clinical Laboratory Tests

- Clinical laboratory testing will be performed according to the SoA (Section 1.3).
- Central and local laboratories will be used. The table below describes when the local or central laboratory will be used
- In circumstances where the sponsor approves local laboratory testing in lieu of central laboratory testing (in the table below), the local laboratory must be qualified in accordance with applicable local regulations.
- Protocol-specific requirements for inclusion or exclusion of participants are detailed in Section 5 of the protocol.
- Additional tests may be performed at any time during the study as determined necessary by the investigator or required by local regulations.
- Pregnancy testing will be performed according to the SoA.
- Investigators must document their review of the laboratory safety results. Laboratory results that will not be reported to investigative sites or other blinded personnel are noted in the table below.

Refer to Section 10.7 for recommended laboratory testing for hypersensitivity events.

Clinical Laboratory Tests	Comments
**Hematology**	Assayed by Lilly-designated laboratory.
Hemoglobin	
Hematocrit	
Erythrocyte count (RBCs [red blood cells])	
Mean cell volume	
Mean cell hemoglobin concentration	
Leukocytes (WBCs [white blood cells])	
Differential	
Neutrophils, segmented	
Lymphocytes	
Monocytes	
Eosinophils	
Basophils	
Platelets	
**Clinical Chemistry**	Assayed by Lilly-designated laboratory.
Sodium	
Potassium	
Chloride	
Bicarbonate	

Clinical Laboratory Tests	Comments
Total bilirubin	
Direct bilirubin	
Alkaline phosphatase (ALP)	
Alanine aminotransferase (ALT)	
Aspartate aminotransferase (AST)	
Gamma-glutamyl transferase (GGT)	
Blood urea nitrogen (BUN)	
Creatinine	
Creatine kinase (CK)	
Uric acid	
Total protein	
Albumin	
Calcium	
Phosphorus	
Glucose	
**Lipids**	
Total cholesterol	
Direct LDL-C	
HDL-C	
VLDL-C	
Triglycerides	
**Pancreas (Exocrine)**	Assayed by Lilly-designated laboratory.
Pancreatic amylase	
Lipase	
**Special Chemistry**	Assayed by Lilly-designated laboratory.
Hemoglobin A1c (HbA1c)	
Calcitonin	
Cystatin C	
N-terminal pro b-type natriuretic peptide (NT-proBNP)	
Cardiac troponin T (cTnT)	
C-reactive protein, high-sensitivity (hsCRP)	
Thyroid stimulating hormone	
**Urine Chemistry**	Assayed by Lilly-designated laboratory.
Albumin	
Creatinine	
**Calculation**	
eGFR (calculated by CKD-EPI equation)	Will be calculated by the Lilly-designated laboratory at all visits.
Urine albumin, creatinine, UACR	
**Hormones (female)**	
Urine Pregnancy	Local laboratory
Serum Pregnancy	Assayed by Lilly-designated laboratory.

Clinical Laboratory Tests	Comments
Follicle Stimulating Hormone (FSH)	Assayed by Lilly-designated laboratory.
**Pharmacokinetic Samples**	• Assayed by Lilly-designated laboratory.<br>• Results will not be provided to the investigative sites.<br>• In the event of systemic drug hypersensitivity reactions (immediate or nonimmediate), additional blood samples will be collected including ADA, PK, and exploratory biomarker samples. PK samples for immunogenicity must be taken prior to drug administration.
**Genetics sample**<br>Whole blood (EDTA)	• Assayed by Lilly-designated laboratory.<br>• Results will not be provided to the investigative sites.
**Exploratory Biomarker Samples**	• Assayed by Lilly-designated laboratory.<br>• Results will not be provided to the investigative sites.
Serum	
EDTA Plasma	
P800 Plasma	
**Immunogenicity Samples**	
Anti-tirzepatide antibodies<br>Anti-tirzepatide neutralizing antibodies	• Assayed by Lilly-designated laboratory.<br>• Results will not be provided to the investigative sites.<br>• In the event of systemic drug hypersensitivity reactions (immediate or nonimmediate), additional blood samples will be collected including ADA, PK, and exploratory biomarker samples. PK samples for immunogenicity must be taken prior to drug administration.

Abbreviations: ADA = antidrug antibody; CKD-EPI = Chronic Kidney Disease-Epidemiology Collaboration; EDTA = ethylenediaminetetraacetic acid; eGFR = estimated glomerular filtration rate; HDL-C = high-density lipoprotein cholesterol; LDL-C = low-density lipoprotein cholesterol; Lilly = Eli Lilly and company; PK = pharmacokinetic; UACR = urine albumin/creatinine ratio; VLDL-C = very low-density lipoprotein cholesterol.

10.3. Appendix 3: Adverse Events: Definitions and Procedures for Recording, Evaluating, Follow-up, and Reporting

The definitions and procedures detailed in this appendix are in accordance with ISO 14155.

Both the investigator and the sponsor will comply with all local medical device reporting requirements.

The detection and documentation procedures described in this protocol apply to all sponsor medical devices provided for use in the study. See Section 6.1.1 for the list of sponsor medical devices).

10.3.1. Definition of AE

AE Definition
• An AE is any untoward medical occurrence in a participant administered a pharmaceutical product and which does not necessarily have a causal relationship with the study drug. An AE can therefore be any unfavourable and unintended sign (including an abnormal laboratory finding), symptom, or disease (new or exacerbated) temporally associated with the use of a medicinal (investigational) product, whether or not related to the medicinal (investigational) product.<br>• An AE is any untoward medical occurrence, unintended disease or injury, or untoward clinical signs (including abnormal laboratory finding) in study participants, users, or other persons, whether or not related to the investigational medical device. This definition includes events related to the investigational medical device or comparator and events related to the procedures involved except for events in users or other persons, which only include events related to investigational devices.
**Events Meeting the AE Definition**
• Any abnormal laboratory test results (hematology, clinical chemistry, or urinalysis) or other safety assessments (for example, ECG, radiological scans, vital signs measurements), including those that worsen from baseline, considered clinically significant in the medical and scientific judgment of the investigator (that is, not related to progression of underlying disease).<br>• Exacerbation of a chronic or intermittent preexisting condition including either an increase in frequency and/or intensity of the condition.<br>• New conditions detected or diagnosed after study drug administration even though they may have been present before the start of the study.<br>• Signs, symptoms, or the clinical sequelae of a suspected drug-drug interaction.<br>• Signs, symptoms, or the clinical sequelae of a suspected overdose of either study drug or a concomitant medication. Overdose per se will not be reported as an AE/SAE unless it is an intentional overdose taken with possible suicidal/self-harming intent. Such an overdose should be reported regardless of sequelae.<br>• "Lack of efficacy" or "failure of expected pharmacological action" per se will not be reported as an AE or SAE. Such instances will be captured in the efficacy assessments.

However, the signs, symptoms, and/or clinical sequelae resulting from lack of efficacy will be reported as an AE or SAE if they fulfill the definition of an AE or SAE.
**Events NOT Meeting the AE Definition**
• Any clinically significant abnormal laboratory findings or other abnormal safety assessments which are associated with the underlying disease, unless judged by the investigator to be more severe than expected for the participant's condition.<br>• The disease/disorder being studied or expected progression, signs, or symptoms of the disease/disorder being studied, unless more severe than expected for the participant's condition.<br>• Medical or surgical procedure (for example, endoscopy, appendectomy): the condition that leads to the procedure is the AE.<br>• Situations in which an untoward medical occurrence did not occur (social and/or convenience admission to a hospital).<br>• Anticipated day-to-day fluctuations of preexisting disease(s) or condition(s) present or detected at the start of the study that do not worsen.

10.3.2. Definition of SAE

If an event is not an AE per the definition above, then it cannot be an SAE even if serious conditions are met (for example, hospitalization for signs/symptoms of the disease under study, death due to progression of disease).

**SAE is defined as any untoward medical occurrence that, at any dose:**
**a. Results in death**
**b. Is life-threatening**<br>The term 'life-threatening' in the definition of 'serious' refers to an event in which the participant was at risk of death at the time of the event. It does not refer to an event, which hypothetically might have caused death, if it were more severe.
**c. Requires inpatient hospitalization or prolongation of existing hospitalization**<br>• In general, hospitalization signifies that the participant has been admitted to the hospital for observation and/or treatment that would not have been appropriate in the physician's office or outpatient setting. Complications that occur during hospitalization are AEs. If a complication prolongs hospitalization or fulfills any other serious criteria, the event is serious. When in doubt as to whether "hospitalization" occurred or was necessary, the AE should be considered serious.<br>• Hospitalization for elective treatment of a preexisting condition that did not worsen from baseline is not considered an AE.
**d. Results in persistent disability/incapacity**

- The term disability means a substantial disruption of a person's ability to conduct normal life functions.
- This definition is not intended to include experiences of relatively minor medical significance such as uncomplicated headache, nausea, vomiting, diarrhea, influenza, and accidental trauma (for example, sprained ankle) which may interfere with or prevent everyday life functions but do not constitute a substantial disruption.

e. Is a congenital anomaly/birth defect

- Abnormal pregnancy outcomes (for example, spontaneous abortion, fetal death, stillbirth, congenital anomalies, ectopic pregnancy) are considered SAEs.

f. Other situations:

- Medical or scientific judgment should be exercised in deciding whether SAE reporting is appropriate in other situations such as important medical events that may not be immediately life-threatening or result in death or hospitalization but may jeopardize the participant or may require medical or surgical intervention to prevent one of the other outcomes listed in the above definition. These events should usually be considered serious.
- Examples of such events include invasive or malignant cancers; intensive treatment in an emergency room or at home for allergic bronchospasm, blood dyscrasias, or convulsions that do not result in hospitalization; or development of drug dependency or drug abuse.

g. Resulted in medical or surgical intervention to prevent life-threatening illness or injury or permanent impairment to a body structure or a body function.

Definition of Serious Adverse Device Effect (SADE)

An SADE is defined as an adverse device effect that has resulted in any of the consequences characteristic of a serious adverse event.

Definition of Unanticipated Adverse Device Effect (UADE)

A UADE is a serious adverse effect on health or safety or any life-threatening problem or death caused by or associated with a device, if that effect, problem, or death was not previously identified in nature, severity, or degree of incidence in the investigational plan or application (including a supplementary plan or application) or any other unanticipated serious problem associated with a device that relates to the rights, safety, or welfare of the participant.

10.3.3. Definition of Product Complaints

Product Complaint
• A product complaint is any written, electronic, or oral communication that alleges deficiencies related to the identity, quality, durability, reliability, safety, effectiveness, or performance of a study drug. When the ability to use the study drug safely is impacted, the following are also product complaints:<br>o Deficiencies in labeling information, and<br>o Use errors for device or drug-device combination products due to ergonomic design elements of the product.<br>• Product complaints related to study drugs used in clinical trials are collected in order to ensure the safety of participants, monitor quality, and to facilitate process and product improvements.<br>• Investigators will instruct participants to contact the site as soon as possible if he or she has a product complaint or problem with the study drug so that the situation can be assessed.<br>• An event may meet the definition of both a product complaint and an AE/SAE. In such cases, it should be reported as both a product complaint and as an AE/SAE.

10.3.4. Recording and Follow-Up of AE and/or SAE and Product Complaints

AE, SAE, and Product Complaint Recording
When an AE/SAE/product complaint occurs, it is the responsibility of the investigator to review all documentation (for example, hospital progress notes, laboratory reports, and diagnostics reports) related to the event.<br><br>The investigator will then record all relevant AE/SAE/product complaint information in the participant's medical records, in accordance with the investigator's normal clinical practice. AE/SAE information is reported on the appropriate eCRF page and product complaint information is reported on the Product Complaint Form.<br><br>Note: An event may meet the definition of both a product complaint and an AE/SAE. In such cases, it should be reported as both a product complaint and as an AE/SAE.<br><br>It is **not** acceptable for the investigator to send photocopies of the participant's medical records to sponsor or designee in lieu of completion of the eCRF page for AE/SAE and the Product Complaint Form for product complaints.<br><br>There may be instances when copies of medical records for certain cases are requested by sponsor or designee. In this case, all participant identifiers, with the exception of the participant number, will be redacted on the copies of the medical records before submission to sponsor or designee.<br><br>The investigator will attempt to establish a diagnosis of the event based on signs, symptoms, and/or other clinical information. Whenever possible, the diagnosis (not the individual signs/symptoms) will be documented as the AE/SAE.

Assessment of Intensity
The investigator will make an assessment of intensity for each AE and SAE reported during the study and assign it to one of the following categories:<br>• Mild: A type of adverse event that is usually transient and may require only minimal treatment or therapeutic intervention. The event does not generally interfere with usual activities of daily living.<br>• Moderate: A type of adverse event that is usually alleviated with additional specific therapeutic intervention. The event interferes with usual activities of daily living, causing discomfort but poses no significant or permanent risk of harm to the research participant.<br>• Severe: A type of adverse event that interrupts usual activities of daily living, or significantly affects clinical status, or may require intensive therapeutic intervention. An AE that is assessed as severe should not be confused with an SAE. Severe is a category utilized for rating the intensity of an event; and both AEs and SAEs can be assessed as severe.<br>An event is defined as 'serious' when it meets at least 1 of the predefined outcomes as described in the definition of an SAE, NOT when it is rated as severe.
**Assessment of Causality**
• The investigator is obligated to assess the relationship between study drug and each occurrence of each AE/SAE.<br>• A "reasonable possibility" of a relationship conveys that there are facts, evidence, and/or arguments to suggest a causal relationship, rather than a relationship cannot be ruled out.<br>• The investigator will use clinical judgment to determine the relationship.<br>• Alternative causes, such as underlying disease(s), concomitant therapy, and other risk factors, as well as the temporal relationship of the event to study drug administration will be considered and investigated.<br>• The investigator will also consult the IB in his/her assessment.<br>• For each AE/SAE, the investigator **must** document in the medical notes that he/she has reviewed the AE/SAE and has provided an assessment of causality.<br>• There may be situations in which an SAE has occurred, and the investigator has minimal information to include in the initial report to sponsor or designee. However, it is very important that the investigator always make an assessment of causality for every event before the initial transmission of the SAE data to sponsor or designee.<br>• The investigator may change his/her opinion of causality in light of follow-up information and send an SAE follow-up report with the updated causality assessment.<br>• The causality assessment is one of the criteria used when determining regulatory reporting requirements.

Follow-up of AEs and SAEs
• The investigator is obligated to perform or arrange for the conduct of supplemental measurements and/or evaluations as medically indicated or as requested by sponsor or designee to elucidate the nature and/or causality of the AE or SAE as fully as possible. This may include additional laboratory tests or investigations, histopathological examinations, or consultation with other health care professionals.

10.3.5. Reporting of SAEs

SAE Reporting via an Electronic Data Collection Tool
• The primary mechanism for reporting an SAE will be the electronic data collection tool.<br>• If the electronic system is unavailable, then the site will use the paper SAE data collection tool (see next section) in order to report the event within 24 hours.<br>• The site will enter the SAE data into the electronic system as soon as it becomes available.<br>• After the study is completed at a given site, the electronic data collection tool will be taken offline to prevent the entry of new data or changes to existing data.<br>• If a site receives a report of a new SAE from a study participant or receives updated data on a previously reported SAE after the electronic data collection tool has been taken off-line, then the site can report this information on a paper SAE form (see next section) or to the sponsor by telephone.<br>• Contacts for SAE reporting can be found in the study training.

10.3.6. Regulatory Reporting Requirements

SAE Regulatory Reporting
• Prompt notification by the investigator to the sponsor of an SAE is essential so that legal obligations and ethical responsibilities towards the safety of participants and the safety of a study drug under clinical investigation are met.<br>• The sponsor has a legal responsibility to notify both the local regulatory authority and other regulatory agencies about the safety of a study drug under clinical investigation. The sponsor will comply with country-specific regulatory requirements relating to safety reporting to the regulatory authority, Institutional Review Boards (IRB)/Independent Ethics Committees (IEC), and investigators.<br>• An investigator who receives an investigator safety report describing an SAE or other specific safety information (for example, summary or listing of SAEs) from the sponsor will review and then file it along with the IB and will notify the IRB/IEC, if appropriate according to local requirements.<br>• As required by local regulations, the investigator will report to their IRB/IEC any UADE (unanticipated problem that resulted in an SAE), or any product complaint that could have led to an SAE had precautions not been taken.

10.4. Appendix 4: Contraceptive Guidance and Collection of Pregnancy Information

10.4.1. Male participants:

Men, regardless of their fertility status, with nonpregnant WOCBP partners must agree to either remain abstinent (if this is their preferred and usual lifestyle) or use condoms, as well as 1 additional highly effective (<1% failure rate) method of contraception (such as combination oral contraceptives, implanted contraceptives, or intrauterine devices) or effective method of contraception (such as diaphragms with spermicide or cervical sponges) for the duration of the study and until their plasma concentrations are below the level that could result in a relevant potential exposure to a possible fetus, predicted to be 90 days plus 5 half-lives following the last dose of study drug, which is approximately 4 months after the last injection.

a) Men and their partners may choose to use a double-barrier method of contraception. (Barrier protection methods without concomitant use of a spermicide are not an effective or acceptable method of contraception. Thus, each barrier method must include use of a spermicide. It should be noted, however, that the use of male and female condoms as a double-barrier method is not considered acceptable due to the high failure rate when these barrier methods are combined.)
b) Periodic abstinence (for example, calendar, ovulation, symptothermal, or postovulation methods), declaration of abstinence just for the duration of a trial, and withdrawal are not acceptable methods of contraception.

Men with pregnant partners should use condoms during intercourse for the duration of the study and until the end of the estimated, relevant potential exposure in WOCBP (4 months).

Men should refrain from sperm donation for the duration of the study and until their plasma concentrations are below the level that could result in a relevant potential exposure to a possible fetus, predicted to be 90 days plus 5 half-lives following the last dose of study drug, which is approximately 4 months.

Men who are in exclusively same-sex relationships (as their preferred and usual lifestyle) are not required to use contraception.

10.4.2. Female participants:

Women of childbearing potential who are abstinent (if this is complete abstinence, as their preferred and usual lifestyle) or in a same-sex relationship (as part of their preferred and usual lifestyle) must agree to either remain abstinent or stay in a same-sex relationship without sexual relationships with males. Periodic abstinence (for example, calendar, ovulation, symptothermal, or postovulation methods), declaration of abstinence just for the duration of a trial, and withdrawal are not acceptable methods of contraception.

Otherwise, WOCBP participating must agree to use 2 forms of effective contraception, where at least 1 form is highly effective (<than 1% failure rate), for the entirety of the study. Contraception must continue following completion of study drug administration for the entirety of the study and for 2 months after the last injection.

a) WOCBP participating must test negative for pregnancy prior to initiation of treatment as indicated by a negative serum pregnancy test at the screening visit followed by a negative urine pregnancy test within 24 hours prior to exposure.

b) Two forms of effective contraception, where at least 1 form is highly effective (such as combination oral contraceptives, implanted contraceptives, or intrauterine devices) will be used for the duration of the trial and for 2 months after the last injection. Effective contraception (such as male or female condoms with spermicide, diaphragms with spermicide, or cervical sponges) may be used as the second therapy. Barrier protection methods without concomitant use of a spermicide are not a reliable or acceptable method. Thus, each barrier method must include use of a spermicide (that is, condom with spermicide, diaphragm with spermicide, or female condom with spermicide). It should be noted that the use of male and female condoms as a double-barrier method is not considered acceptable due to the high failure rate when these methods are combined.

c) Not be breastfeeding.

Women not of childbearing potential may participate and include those who are:

a) Infertile due to surgical sterilization, or

b) Postmenopausal.

Definitions:

Woman of Childbearing Potential (WOCBP)

A woman is considered fertile following menarche and until becoming postmenopausal unless permanently sterile (see below).

If fertility is unclear (for example, amenorrhea in adolescents or athletes) and a menstrual cycle cannot be confirmed before first dose of study drug, additional evaluation should be considered.

Women in the following categories are not considered WOCBP

1. Premenarchal
2. Premenopausal female with 1 of the following:
   - Documented hysterectomy
   - Documented bilateral salpingectomy
   - Documented bilateral oophorectomy

For individuals with permanent infertility due to an alternate medical cause other than the above, (for example, mullerian agenesis, androgen insensitivity), investigator discretion should be applied to determining study entry.

Note: Determination can come from the site personnel’s review of the participant’s medical records, medical examination, or medical history interview.

3. Postmenopausal female is defined as follows:
   a. A woman at any age at least 6 weeks post-surgical bilateral oophorectomy with or without hysterectomy, confirmed by operative note
   b. A woman at least 40 years of age and up to 55 years old with an intact uterus, not on hormone therapy*, who has had cessation of menses for at least 12 consecutive

months without an alternative medical cause, AND with a follicle-stimulating hormone >40 mIU/mL; or

c. A woman 55 or older not on hormone therapy, who has had at least 12 months of spontaneous amenorrhea, or
d. A woman at least 55 years of age with a diagnosis of menopause prior to starting hormone replacement therapy

* Women should not be taking medications during amenorrhea such as oral contraceptives, hormones, gonadotropin-releasing hormone, anti-estrogens, selective estrogen receptor modulators (SERMs), or chemotherapy that could induce transient amenorrhea.

Contraception Guidance:

Highly Effective Methods of Contraception:

- Combined oral contraceptive pill and mini pill
- NuvaRing®
- Implantable contraceptives
- Injectable contraceptives (such as Depo-Provera®)
- Intrauterine device (such as Mirena® and ParaGard®)
- Contraceptive patch – ONLY women <198 pounds or 90 kg
- Total abstinence (if this is their preferred and usual lifestyle) or in a same-sex relationship with no sexual relationship with males (as part of their preferred and usual lifestyle).

Note: periodic abstinence (for example, calendar, ovulation, symptothermal, postovulation methods), declaration of abstinence just for the duration of a trial, and withdrawal are not acceptable methods of contraception.

Note: Implantable contraceptives and injectable contraceptives (such as Depo-Provera) are only permitted if started prior to screening. Participants should not start these methods of contraception after being enrolled in the study.

- Vasectomy - for men in clinical trials

Effective Methods of Contraception (must use combination of 2 methods):

- Male condom with spermicide
- Female condom with spermicide
- Diaphragm with spermicide
- Cervical sponge
- Cervical cap with spermicide

Collection of Pregnancy Information

Male participants with partners who become pregnant

- The investigator will attempt to collect pregnancy information on any male participant's female partner who becomes pregnant while the male participant is in this study.
- After obtaining the necessary signed informed consent from the pregnant female partner directly, the investigator will record pregnancy information on the appropriate form and

submit it to the sponsor within 24 hours of learning of the partner’s pregnancy. The female partner will also be followed to determine the outcome of the pregnancy. Information on the status of the mother and child will be forwarded to the sponsor. Generally, the follow-up will be no longer than 6 to 8 weeks following the estimated delivery date. Any termination of the pregnancy will be reported regardless of fetal status (presence or absence of anomalies) or indication for the procedure.

Female Participants who become pregnant

- The investigator will collect pregnancy information on any female participant who becomes pregnant while participating in this study. The initial information will be recorded on the appropriate form and submitted to the sponsor within 24 hours of learning of a participant's pregnancy.
- The participant will be followed to determine the outcome of the pregnancy. The investigator will collect follow-up information on the participant and the neonate, and the information will be forwarded to the sponsor. Generally, follow-up will not be required for longer than 6 to 8 weeks beyond the estimated delivery date. Any termination of pregnancy will be reported, regardless of fetal status (presence or absence of anomalies) or indication for the procedure.
- While pregnancy itself is not considered to be an AE or SAE, any pregnancy complication or elective termination of a pregnancy for medical reasons will be reported as an AE or SAE.
- A spontaneous abortion (occurring at <20 weeks gestational age) or still birth (occurring at >20 weeks gestational age) is always considered to be an SAE and will be reported as such.
- Any poststudy, pregnancy-related SAE considered reasonably related to the study drug by the investigator will be reported to the sponsor as described in protocol Section 8.3.1. While the investigator is not obligated to actively seek this information in former study participants, he or she may learn of an SAE through spontaneous reporting.
- Any female participant who becomes pregnant while participating in the study will discontinue study drug and be withdrawn from the study.

10.5. Appendix 5: Adverse Events of Special Interest: Definitions and Procedures for Recording, Evaluating, Follow-Up, and Reporting.

10.5.1. Special Safety Topics

10.5.1.1. Hypoglycemia

Upon ICF signing, all participants will be educated about signs and symptoms of hypoglycemia, how to treat hypoglycemia, and how to collect appropriate information for each episode of hypoglycemia.

Hypoglycemia may be identified by spontaneous reporting of symptoms from participants (whether confirmed or unconfirmed by simultaneous glucose values) or by BG samples collected during study visits.

All participants with T2DM and who develop diabetes during the study will be provided with glucometers.

Participants with T2DM will be provided a diary to record relevant information (for example, glucose values, symptoms).

All hypoglycemic episodes are to be recorded on a specific eCRF and should not be otherwise recorded as AEs unless the event meets severe criteria. If a hypoglycemic event meets severe criteria (see definition below), it should be recorded as serious on the AE eCRFs, and reported to the sponsor as an SAE. To avoid duplicate reporting, all consecutive BG values <70 mg/dL (<3.9 mmol/L) occurring within a 1-hour period may be considered to be a single hypoglycemic event (Weinberg et al. 2010; Danne et al. 2013).

Investigators should use the following definitions and criteria when diagnosing and categorizing an episode considered to be related to hypoglycemia (the BG values in this section refer to values determined by a laboratory or International Federation of Clinical Chemistry and Laboratory Medicine blood-equivalent glucose meters and strips) in accordance with the 2020 American Diabetes Association position statement on glycemic targets (ADA 2020):

Glucose Alert Value (Level 1):

- Documented symptomatic hypoglycemia is defined as any time a participant feels that he or she is experiencing symptoms and/or signs associated with hypoglycemia and has a BG level of <70 mg/dL (<3.9 mmol/L).
- Documented asymptomatic hypoglycemia is defined as any event not accompanied by typical symptoms of hypoglycemia, but with a measured BG <70 mg/dL (<3.9 mmol/L).
- Documented unspecified hypoglycemia is defined as any event with no information about symptoms of hypoglycemia available, but with a measured BG <70 mg/dL (<3.9 mmol/L).

Clinically Significant Hypoglycemia (Level 2):

- Documented symptomatic hypoglycemia is defined as any time a participant feels that he or she is experiencing symptoms and/or signs associated with hypoglycemia and has a BG level of <54 mg/dL (<3.0 mmol/L).

- Documented asymptomatic hypoglycemia is defined as any event not accompanied by typical symptoms of hypoglycemia but with a measured BG <54 mg/dL (<3.0 mmol/L).
- Documented unspecified hypoglycemia is defined as any event with no information about symptoms of hypoglycemia available but with a measured BG <54 mg/dL (<3.0 mmol/L).

Severe Hypoglycemia (Level 3):

- Severe hypoglycemia is defined as an episode with severe cognitive impairment requiring the assistance of another person to actively administer carbohydrate, glucagon, or other resuscitative actions. These episodes may be associated with sufficient neuroglycopenia to induce seizure or coma. Blood glucose measurements may not be available during such an event, but neurological recovery attributable to the restoration of BG to normal is considered sufficient evidence that the event was induced by a low BG concentration.

Nocturnal Hypoglycemia:

Nocturnal hypoglycemia is a hypoglycemia event (including severe hypoglycemia) that occurs at night, presumably during sleep.

10.5.1.2. Pancreatitis

Acute pancreatitis is defined as an AE of interest in all trials with tirzepatide, including this trial.

Acute pancreatitis is an acute inflammatory process of the pancreas that may also involve peripancreatic tissues and/or remote organ systems (Banks and Freeman 2006). The diagnosis of acute pancreatitis requires 2 of the following 3 features:

- abdominal pain, characteristic of acute pancreatitis (generally located in the epigastrium and radiates to the back in approximately half the cases) (Banks and Freeman 2006; Koizumi et al. 2006); the pain is often associated with nausea and vomiting
- serum amylase (total and/or pancreatic) and/or lipase ≥3X ULN, and
- characteristic findings of acute pancreatitis on CT scan or MRI.

If acute pancreatitis is suspected, appropriate laboratory tests (including levels of pancreatic amylase and lipase) should be obtained via the central laboratory (and locally, if needed).

Imaging studies, such as abdominal CT scan with or without contrast, MRI, or gallbladder ultrasound, should be performed. Abdominal ultrasound may be used as an alternative method only if CT and MRI cannot be performed. If laboratory values and/or abdominal imaging support the diagnosis of acute pancreatitis, the participant must discontinue therapy with tirzepatide but will continue in the study. A review of the participant's concomitant medications should be conducted to assess any potential causal relationship with pancreatitis.

Each AE of pancreatitis must be reported. If typical signs and/or symptoms of pancreatitis are present and confirmed by laboratory values (lipase or amylase [total and/or pancreatic]) and imaging studies, the event must be reported as an SAE. For a potential case that does not meet all of these criteria, it is up to the investigator to determine the seriousness of the case (AE or SAE) and the relatedness of the event to study drug(s).

Each participant will have measurements of p-amylase and lipase (assessed at the central laboratory) as shown on the SoA (Section 1.3) to assess the effects of the investigational doses of tirzepatide on pancreatic enzyme levels. Serial measurements of pancreatic enzymes have limited clinical value for predicting episodes of acute pancreatitis in asymptomatic participants (Nauck et al. 2017; Steinberg et al. 2017a, 2017b). Thus, further diagnostic follow-up of cases of asymptomatic pancreatic hyperenzymemia (lipase and/or pancreatic amylase ≥3X ULN) is not mandated but may be performed based on the investigator's clinical judgment and assessment of the participant's overall clinical condition. Only cases of pancreatic hyperenzymemia that undergo additional diagnostic follow-up and/or are accompanied by symptoms suggestive of pancreatitis will be submitted for adjudication.

All suspected cases of acute or chronic pancreatitis will be adjudicated by an independent clinical endpoint committee. In addition, AEs of severe or serious abdominal pain of unknown etiology will also be submitted to the adjudication committee to assess for possible pancreatitis or other pancreatic disease. Relevant data from participants with acute or chronic pancreatitis and those with severe or serious abdominal pain will be entered into a specifically designed eCRF page. The adjudication committee representative will enter the results of adjudication in a corresponding eCRF page.

10.5.1.3. Thyroid Malignancies and C-Cell Hyperplasia

Individuals with personal or family history of MTC and/or MEN-2 will be excluded from the study. Participants who are diagnosed with MTC and/or MEN-2 during the study will have study drug stopped and should continue follow-up with an endocrinologist.

The assessment of thyroid safety during the trial will include reporting of any case of thyroid malignancy (including MTC and papillary carcinoma) and measurements of calcitonin. This data will be captured in specific eCRFs. The purpose of calcitonin measurements is to assess the potential of tirzepatide to affect thyroid C-cell function, which may indicate development of C-cell hyperplasia and neoplasms.

10.5.1.4. Calcitonin Measurements

If an increased calcitonin value (see definitions below) is observed in a participant who has been administered a medication that is known to increase serum calcitonin, then this medication should be stopped, and calcitonin levels should be measured after an appropriate washout period.

For participants who require additional endocrine assessment because of increased calcitonin concentration as defined in this section, data from the follow-up assessment will be collected in the specific section of the eCRF.

Calcitonin Measurements in Participants with eGFR ≥60 mL/min/1.73m^2

A significant increase in calcitonin for participants with eGFR ≥60 mL/min is defined below. If a participant's laboratory results meet these criteria, these clinically significant laboratory results should be recorded as an AE.

- calcitonin value ≥20 ng/L and <35 ng/L AND ≥50% increase from the screening value.
  - These participants will be asked to repeat the measurement within 1 month. If this repeat value is increasing (≥10% increase), study drug should be stopped, and the

participant encouraged to undergo additional endocrine assessment and longer-term follow-up by an endocrinologist to exclude any serious adverse effect on the thyroid.

- calcitonin value ≥35 ng/L AND ≥50% over the screening value.
    - In these participants, study drug should be stopped, and the participant recommended to immediately undergo additional endocrine assessments and longer-term follow-up by an endocrinologist.

Calcitonin Measurement in Participants with eGFR <60 mL/min/1.73m^2

A significant increase in calcitonin for participants with eGFR <60 mL/min/1.73m^2 is defined as a calcitonin value ≥35 ng/L AND ≥50% over the screening value. If a participant's labs meet these criteria, these clinically significant labs should be recorded as an AE.

In these participants, study drug should be discontinued (after first confirming the value), and the participant recommended to immediately undergo additional endocrine assessments and longer-term follow-up by an endocrinologist to exclude any serious adverse effect on the thyroid.

10.5.1.5. Major Adverse Cardiovascular Events

Deaths and nonfatal CV AEs will be adjudicated by a committee of physicians external to the sponsor with cardiology expertise. This committee will be blinded to treatment assignment. The nonfatal CV AEs to be adjudicated include:

- myocardial infarction
- hospitalization for unstable angina
- coronary interventions (such as coronary artery bypass graft or percutaneous coronary intervention), and
- cerebrovascular events, including cerebrovascular accident (stroke) and transient ischemic attack.

10.5.1.6. Supraventricular Arrhythmias and Cardiac Conduction Disorders

Participants who develop any event from these groups of disorders should undergo an ECG, which will be retained at the site as a source document. Additional diagnostic tests to determine exact diagnosis should be performed, as needed. The specific diagnosis will be recorded as an AE. Events that meet criteria for serious conditions as described in Section 10.3.2 must be reported as SAEs. If a clinically significant finding is identified by ECG (including, but not limited to, AF or changes from baseline in corrected QT interval), the investigator or qualified designee will determine if any change in study participant management is needed. This review of the ECG printed at the time of collection must be documented. Any new clinically relevant finding should be reported as an AE.

10.5.1.7. Hypersensitivity Events

All allergic or hypersensitivity reactions will be reported by the investigator as either AEs, or if any serious criterion is met, as SAEs.

In the event of suspected drug hypersensitivity reactions (immediate or nonimmediate) in subjects who experience moderate-to-severe reactions as assessed by the investigator, unscheduled blood samples will be collected as outlined in Appendix 7 (Section 10.7).

Additional data, such as type of reaction and treatment received, will be collected on any AEs or SAEs that the investigator deems related to study drug via the eCRF created for this purpose.

Study drug should be temporarily interrupted in any individual suspected of having a severe or serious allergic reaction to study drug. Study drug may be restarted when/if it is safe to do so, in the opinion of the investigator.

10.5.1.8. Injection Site Reactions

Injection site reactions will be collected on the eCRF separate from the hypersensitivity reaction eCRF. At the time of AE occurrence, samples will be collected for measurement of tirzepatide ADA and tirzepatide concentration.

10.5.1.9. Antidrug Antibodies

The occurrence of ADA formation will be assessed as outlined in Section 8.9.

10.5.1.10. Hepatobiliary Disorders

All events of treatment-emergent biliary colic, cholecystitis, or other suspected events related to gallbladder disease should be evaluated and additional diagnostic tests performed, as needed. In cases of elevated liver markers, hepatic monitoring should be initiated as outlined in Appendix 8 (Section 10.8).

10.5.1.11. Severe Gastrointestinal Adverse Events

Tirzepatide may cause severe GI AEs such as nausea, vomiting, and diarrhea. Information about severe GI AEs as well as antiemetic/antidiarrheal use will be collected in the eCRF/AE form. For detailed information concerning the management of GI AEs, please refer to Section 6.5.

10.5.1.12. Acute Renal Events

Renal safety will be assessed based on repeated renal function assessment as well as assessment of AEs suggestive of acute or worsening of chronic renal failure. Gastrointestinal AEs have been reported with tirzepatide, including nausea, diarrhea, and vomiting. This is consistent with other GLP-1 RAs (Aroda and Ratner 2011). The events may lead to dehydration, which could cause a deterioration in renal function, including acute renal failure.

Participants should be advised to notify investigators in case of severe nausea, frequent vomiting, or symptoms of dehydration.

10.6. Appendix 6: Genetics

Use/Analysis of DNA

- Genetic variation may impact a participant's response to study drug, susceptibility to, severity, and progression of disease. Variable response to study drug may be due to genetic determinants that impact drug absorption, distribution, metabolism, and excretion; mechanism of action of the drug; disease etiology; and/or molecular subtype of the disease being treated. Therefore, where local regulations and IRB/IEC allow, a blood sample will be collected for DNA analysis from consenting participants.
- DNA samples will be used for research related to tirzepatide or HF and related diseases. They may also be used to develop tests/assays including diagnostic tests related to tirzepatide or HF. Genetic research may consist of the analysis of one or more candidate genes, the analysis of genetic markers throughout the genome, or analysis of the entire genome (as appropriate).
- DNA sample analysis may be performed on pharmacogenetic variants thought to play a role in T2DM or CV disease to evaluate their association with observed clinical outcomes to tirzepatide in this study. In the event the observation of a study drug response, the samples may be genotyped, and analysis may be performed to evaluate a genetic association with response to tirzepatide. These investigations may be limited to a focused, candidate-gene study or, if appropriate, genome-wide association studies may be performed to identify regions of the genome associated with the variability observed in drug response. Samples may be used for investigations related to the disease, drug, or class of drugs under study in the context of this clinical program; however, samples may not be used for broad, exploratory, unspecified disease or population genetic analysis. Additional analyses may be conducted if it is hypothesized that this may help further understand the clinical data.
- The samples may be analyzed as part of a multi-study assessment of genetic factors involved in the response to tirzepatide or study drugs of this class to understand the study disease or related conditions.
- The results of genetic analyses may be reported in the CSR or in a separate study summary.
- The sponsor will store the DNA samples in a secure storage space with adequate measures to protect confidentiality.
- The samples will be retained while research on tirzepatide continues but no longer than 15 years or another period as per local requirements (see Section 10.1.10).

10.7. Appendix 7: Recommended Laboratory Testing for Hypersensitivity Events

Laboratory testing should be performed at the time of a systemic hypersensitivity event. The management of the AE may warrant lab testing beyond that described below and should be performed as clinically indicated. Laboratory assessments should be performed if the participant experiences generalized urticaria or if anaphylaxis is suspected.

- Collect the sample after the participant has been stabilized and within 1 to 2 hours of the event; however, samples may be obtained as late as 12 hours after the event as analytes can remain altered for an extended period of time. Record the time at which the sample was collected.
- Obtain a follow-up sample at the next regularly scheduled visit or after approximately 4 weeks, whichever is later.

Clinical Laboratory Tests for Hypersensitivity Events

**Hypersensitivity Tests**	**Notes**<br>Selected test may be obtained in the event of anaphylaxis or systemic allergic/hypersensitivity reactions.
Tirzepatide antidrug antibodies (immunogenicity/ADA)	Assayed by Lilly-designated laboratory.<br>Results will not be provided to the investigative sites.
Tirzepatide concentrations (PK)	Assayed by Lilly-designated laboratory.<br>Results will not be provided to the investigative sites.
Tryptase	Assayed by Lilly-designated laboratory.<br>Results will not be provided to the investigative sites.<br>Urine N-methylhistamine testing is performed in addition to tryptase testing. Collect the first void urine sample following the event. Collect a follow-up urine sample after approximately 4 weeks.<br>**Note:** If a tryptase sample is obtained more than 2 hours after the event (that is, within 2-12 hours), or is not obtained because more than 12 hours have lapsed since the event, collect a urine sample for N-methylhistamine testing.
N-methylhistamine	Will be performed if validated assay is available.<br>Assayed by Lilly-designated laboratory.<br>Results will not be provided to the investigative sites.
Drug-specific IgE	Will be performed if a validated assay is available.<br>Assayed by Lilly-designated laboratory.<br>Results will not be provided to the investigative sites.
Basophil activation test	Will be performed if a validated assay is available.<br>Assayed by Lilly-designated laboratory.<br>Results will not be provided to the investigative sites.<br>**NOTE:** The basophil activation test is an in vitro, cell-based assay that only requires a serum sample. It is a surrogate assay for drug specific-IgE but is not specific for IgE.
Complement (C3, C3a and C5a)	Assayed by Lilly-designated laboratory.<br>Results will not be provided to the investigative sites.
Cytokine panel: IL-6, IL-1β, IL-10	Assayed by Lilly-designated laboratory.<br>Results will not be provided to the investigative sites.

Abbreviations: ADA = antidrug antibody; IgE = immunoglobulin E; IL = interleukin; PK = pharmacokinetic.

10.8. Appendix 8: Liver Safety: Suggested Actions and Follow-Up Assessments

Hepatic Evaluation Testing

See Section 8.2.5.1 for guidance on appropriate test selection.

The Lilly-designated central laboratory must complete the analysis of all selected testing except for microbiology testing.

Local testing may be performed in addition to central testing when necessary for immediate participant management.

Results will be reported if a validated test or calculation is available.

**Hematology**	**Clinical Chemistry**
Hemoglobin	Total bilirubin
Hematocrit	Direct bilirubin
Erythrocytes (red blood cells [RBCs])	Alkaline phosphatase (ALP)
Leukocytes (white blood cells [WBCs])	Alanine aminotransferase (ALT)
Differential:	Aspartate aminotransferase (AST)
Neutrophils, segmented	Gamma-glutamyl transferase (GGT)
Lymphocytes	Creatine kinase (CK)
Monocytes	**Other Chemistry**
Basophils	Acetaminophen
Eosinophils	Acetaminophen protein adducts
Platelets	Alkaline phosphatase isoenzymes
Cell morphology (RBC and WBC)	Ceruloplasmin
**Coagulation**	Copper
	Ethyl alcohol (EtOH)
Prothrombin time, INR (PT-INR)	Haptoglobin
**Serology**	Immunoglobulin IgA (quantitative)
Hepatitis A virus (HAV) testing:	Immunoglobulin IgG (quantitative)
HAV total antibody	Immunoglobulin IgM (quantitative)
HAV IgM antibody	Phosphatidylethanol (PEth)
Hepatis B virus (HBV) testing:	**Urine Chemistry**
Hepatitis B surface antigen (HBsAg)	Drug screen
Hepatitis B surface antibody (anti-HBs)	Ethyl glucuronide (EtG)
Hepatitis B core total antibody (anti-HBc)	**Other Serology**
Hepatitis B core IgM antibody	Antinuclear antibody (ANA)
Hepatitis B core IgG antibody	Anti-smooth muscle antibody (ASMA)[a]

HBV DNA[b]	Anti-actin antibody[c]
Hepatis C virus (HCV) testing:	Epstein-Barr virus (EBV) testing:
HCV antibody	EBV antibody
HCV RNA[b]	EBV DNA[b]
Hepatitis D virus (HDV) testing:	Cytomegalovirus (CMV) testing:
HDV antibody	CMV antibody
Hepatitis E virus (HEV) testing:	CMV DNA[b]
HEV IgG antibody	Herpes simplex virus (HSV) testing:
HEV IgM antibody	HSV (Type 1 and 2) antibody
HEV RNA[b]	HSV (Type 1 and 2) DNA[b]
**Microbiology**[d]	Liver kidney microsomal type 1 (LKM-1) antibody
Culture:	
Blood	
Urine	

Abbreviations: Ig = immunoglobulin; INR = international normalized ratio; PT = prothrombin time.

[a] Not required if anti-actin antibody is tested.

[b] Reflex/confirmation dependent on regulatory requirements, testing availability, or both.

[c] Not required if anti-smooth muscle antibody (ASMA) is tested.

[d] Assayed ONLY by investigator-designated local laboratory; no central testing available.

10.9. Appendix 9: Medical Device Adverse Events (AEs), Adverse Device Effects (ADEs), Serious Adverse Events (SAEs) and Device Deficiencies: Definition and Procedures for Recording, Evaluating, Follow-Up, and Reporting

Refer to Section 10.3 for definitions and procedures for recording, evaluating, follow-up, and reporting of all events.

10.10. Appendix 10: Six-Minute Walk Test Screening Procedures

10.10.1. Screening Procedures and Flow Diagram

The flow diagram below details the participant flow and eligibility with the 6MWT.

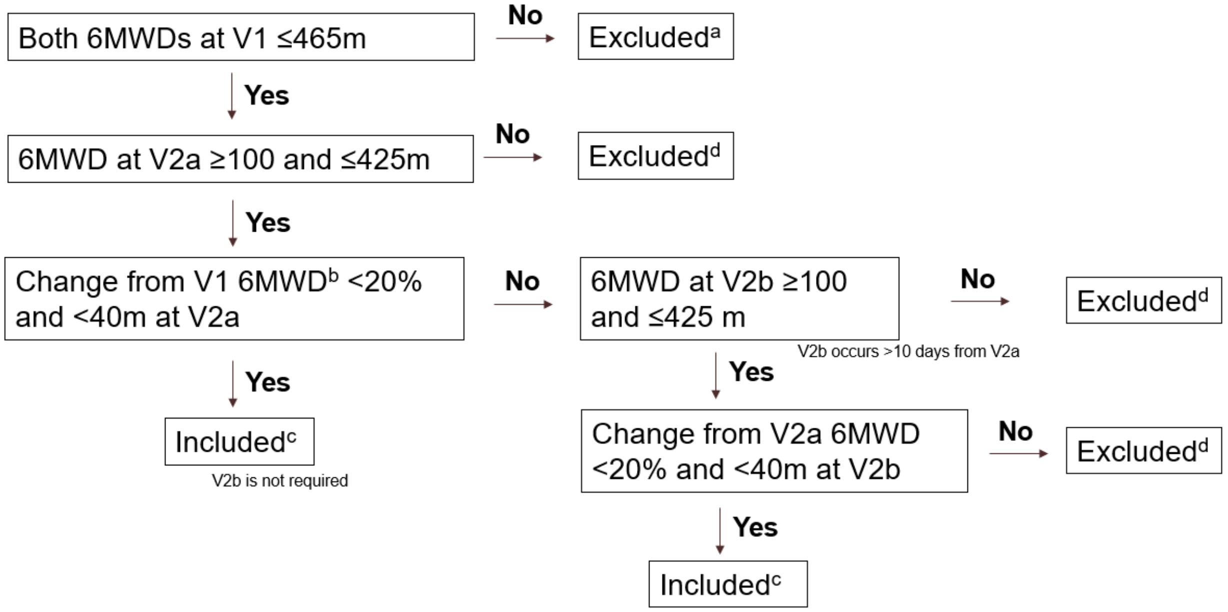

Abbreviations: 6MWD = 6-minute walk test distance; 6MWT = 6-minute walk test; V1 = Visit 1; V2a = Visit 2a; V2b = Visit 2b.

a Rescreening is not allowed

b Use the higher value of the two Visit 1 6MWD as a reference for Visit 2a

c Continue with other Visit 2 assessments according to the SoA

d Participants excluded on 6MWT may be re-screened after a minimum of 2 weeks

10.11. Appendix 11: New York Heart Association Classification of Heart Failure

Class	Symptomatology
I	No symptoms. Ordinary physical activity such as walking and climbing stairs does not cause fatigue or dyspnea.
II	Symptoms with ordinary physical activity. Walking or climbing stairs rapidly, walking uphill, walking or stair climbing after meals, in cold weather, in wind or when under emotional stress causes undue fatigue or dyspnea.
III	Symptoms with less than ordinary physical activity. Walking 1-2 blocks on the level and climbing more than one flight of stairs in normal conditions causes undue fatigue or dyspnea.
IV	Symptoms at rest. Inability to carry on any physical activity without fatigue or dyspnea.

10.12. Appendix 12: Abbreviations

Term	Definition
**6MWD**	6-minute walk test distance
**6MWT**	6-minute walk test
**ADA**	antidrug antibody
**AE**	adverse event
**AESI**	adverse events of special interest
**AF**	atrial fibrillation
**ALP**	alkaline phosphatase
**ALT**	alanine aminotransferase
**AST**	aspartate aminotransferase
**blinding/masking**	A single-blind study is one in which the investigator and/or his staff are aware of the treatment but the participant is not, or vice versa, or when the sponsor is aware of the treatment but the investigator and/his staff and the participant are not.<br>A double-blind study is one in which neither the participant nor any of the investigator or sponsor staff who are involved in the treatment or clinical evaluation of the subjects are aware of the treatment received.
**BG**	blood glucose
**BMI**	body mass index
**CEC**	clinical endpoint committee
**CFR**	Code of Federal Regulations
**CIOMS**	Council for International Organizations of Medical Sciences
**complaint**	A complaint is any written, electronic, or oral communication that alleges deficiencies related to the identity, quality, purity, durability, reliability, safety or effectiveness, or performance of a drug or drug delivery system.
**compliance**	Adherence to all study-related, good clinical practice (GCP), and applicable regulatory requirements.
**CONSORT**	Consolidated Standards of Reporting Trials
**CRF**	case report form
**CRP**	clinical research physician: Individual responsible for the medical conduct of the study. Responsibilities of the CRP may be performed by a physician, clinical research scientist, global safety physician or other medical officer.

Term	Definition
**CSR**	clinical study report
**CT**	computed tomography
**CV**	cardiovascular
**D Bil**	direct bilirubin
**DMC**	data monitoring committee
**Device Deficiencies**	Equivalent to product complaint
**ECG**	electrocardiogram
**eCOA**	electronic clinical outcome assessment
**eCRF**	electronic case report form
**EDC**	electronic data capture
**eGFR**	estimated glomerular filtration rate
**enroll**	The act of assigning a participant to a treatment. Participants who are enrolled in the study are those who have been assigned to a treatment.
**enter**	Participants entered into a study are those who sign the informed consent form directly or through their legally acceptable representatives.
**ERB**	ethical review board
**ET**	early termination
**EV**	extended visit
**GCP**	good clinical practice
**GGT**	gamma-glutamyl transferase
**GI**	gastrointestinal
**GIP**	glucose-dependent insulinotropic polypeptide
**GLP-1**	glucagon-like peptide-1
**HbA1c**	hemoglobin A1c
**HF**	heart failure
**HFpEF**	heart failure with preserved ejection fraction
**HIPAA**	Health Insurance Portability and Accountability Act

Term	Definition
**IB**	Investigator's Brochure
**ICF**	informed consent form
**ICH**	International Council for Harmonisation
**IEC**	independent ethics committee
**informed consent**	A process by which a participant voluntarily confirms his or her willingness to participate in a particular study, after having been informed of all aspects of the study that are relevant to the participant's decision to participate. Informed consent is documented by means of a written, signed and dated informed consent form.
**INR**	international normalized ratio
**interim analysis**	An interim analysis is an analysis of clinical study data, separated into treatment groups, that is conducted before the final reporting database is created/locked.
**IRB**	institutional review board
**ISO**	International Organization for Standardization
**ITT**	intention to treat: The principle that asserts that the effect of a treatment policy can be best assessed by evaluating on the basis of the intention to treat a participant (that is, the planned treatment regimen) rather than the actual treatment given. It has the consequence that participant allocated to a treatment group should be followed up, assessed, and analyzed as members of that group irrespective of their compliance to the planned course of treatment.
**IV**	intravenous
**IWRS**	interactive web-response system
**KCCQ**	Kansas City Cardiomyopathy Questionnaire
**KCCQ-CSS**	Kansas City Cardiomyopathy Questionnaire – Clinical Summary Scale
**LA**	left atrial
**LAV**	left atrial volume
**LVDEP**	left ventricular end-diastolic pressure
**LVEF**	left ventricular ejection fraction
**MedDRA**	Medical Dictionary for Regulatory Activities
**MEN-2**	multiple endocrine neoplasia type 2
**MMRM**	mixed model repeated measures
**MRI**	magnetic resonance imaging

Term	Definition
**MTC**	medullary thyroid cancer
**MTD**	maximum tolerated dose
**NT-proBNP**	N-terminal pro b-type natriuretic peptide
**NYHA**	New York Heart Association
**participant**	Equivalent to CDISC term "subject": an individual who participates in a clinical trial, either as recipient of an investigational medicinal product or as a control
**PC**	product complaint
**PCWP**	pulmonary capillary wedge pressure
**PK**	pharmacokinetics
**PT**	prothrombin time
**QTc**	corrected QT interval
**QW**	weekly
**RA**	receptor agonist
**SADE**	serious adverse device effect
**SAE**	serious adverse event
**SAP**	statistical analysis plan
**SBP**	systolic blood pressure
**SC**	subcutaneous
**screen**	The act of determining if an individual meets minimum requirements to become part of a pool of potential candidates for participation in a clinical study.
**SoA**	schedule of activities
**study intervention**	for this study, study intervention may be interpreted/synonymous with study drug
**SUSAR**	suspected unexpected serious adverse reaction
**T1DM**	type 1 diabetes mellitus
**T2DM**	type 2 diabetes mellitus
**TBL**	total bilirubin

Term	Definition
**TEAE**	Treatment-emergent adverse event: An untoward medical occurrence that emerges during a defined treatment period, having been absent pretreatment, or worsens relative to the pretreatment state, and does not necessarily have to have a causal relationship with this treatment.
**UADE**	unanticipated adverse device effect
**ULN**	upper limit of normal
**WOCBP**	women of childbearing potential

11. References

[ADA] American Diabetes Association. Glycemic targets: standards of medical care in diabetes – 2020. *Diabetes Care*. 2020;43(suppl 1):S66-S76. https://doi.org/10.2337/dc20-S006

Aminian A, Zajichek A, Arterburn DE, et al. Association of metabolic surgery with major adverse cardiovascular outcomes in patients with type 2 diabetes and obesity. *JAMA*. 2019;322(13):1271-1282. https://doi.org/10.1001/jama.2019.14231

Aroda VR, Ratner R. The safety and tolerability of GLP-1 receptor agonists in the treatment of type 2 diabetes: a review. *Diabetes Metab Res Rev*. 2011;27(6):528-542. https://doi.org/10.1002/dmrr.1202

Banks PA, Freeman ML.; Practice Parameters Committee of the American College of Gastroenterology. Practice guidelines in acute pancreatitis. *Am J Gastroenterol*. 2006;101(10):2379-2400. https://doi.org/10.1111/j.1572-0241.2006.00856.x

Danne T, Philotheou A, Goldman D, et al. A randomized trial comparing the rate of hypoglycemia – assessed using continuous glucose monitoring – in 125 preschool children with type 1 diabetes treated with insulin glargine or NPH insulin (the PRESCHOOL study). *Pediatr Diabetes*. 2013;14(8):593-601. https://doi.org/10.1111/pedi.12051

Davies MJ, Bergenstal R, Bode B, et al. Efficacy of liraglutide for weight loss among patients with type 2 diabetes: The SCALE Diabetes Randomized Clinical Trial. *JAMA*. 2015;314(7):687-699. https://doi.org/10.1001/jama.2015.9676 Erratum in: JAMA. 2016 Jan 5;315(1):90. PMID: 26284720.

Davies MJ, D'Alessio DA, Fradkin J, et al. Management of hyperglycemia in type 2 diabetes, 2018. A consensus report by the American Diabetes Association (ADA) and the European Association for the Study of Diabetes (EASD). *Diabetes Care*. 2018;41(12):2669-2701. https://doi.org/10.2337/dci18-0033

[EMA] European Medicines Agency. Guideline on clinical evaluation of medicinal products used in weight management. EMA/CHMP/311805/2014. Published June 23, 2016. Accessed December 23, 2020. https://www.ema.europa.eu/en/documents/scientific-guideline/guideline-clinical-evaluation-medicinal-products-used-weight-management-revision-1_en.pdf

EuroQol Research Foundation. EQ-5D-5L User Guide, Version 3.0. Updated September 2019. Accessed March 13, 2020. https://euroqol.org/publications/user-guides

[FDA] United States Food and Drug Administration. Guidance for industry: developing products for weight management. Published February 2007. Accessed December 23, 2020. https://www.fda.gov/media/71252/download

Ferreira JP, Jhund PS, Duarte K, et al. Use of the win ratio in cardiovascular trials. *JACC Heart Fail*. 2020;8(6):441-450. https://doi.org/10.1016/j.jchf.2020.02.010

Finkelstein DM, Schoenfeld DA. Combining mortality and longitudinal measures in clinical trials. *Stat Med*. 1999;18(11):1341-1354. https://doi.org/10.1002/(sici)1097-0258(19990615)18:11<1341::aid-sim129>3.0.co;2-7

Frias JP, Nauck MA, Van J, et al. Efficacy and safety of LY3298176, a novel dual GIP and GLP-1 receptor agonist, in patients with type 2 diabetes: a randomised, placebo-controlled and active comparator-controlled phase 2 trial. *Lancet*. 2018;392(10160):2180-2193. https://doi.org/10.1016/s0140-6736(18)32260-8

Green CP, Porter CB, Bresnahan DR, Spertus JA. Development and evaluation of the Kansas City Cardiomyopathy Questionnaire: a new health status measure for heart failure. *J Am Coll Cardiol*. 2000;35(5):1245-1255. https://doi.org/10.1016/S0735-1097(00)00531-3

Herring LY, Stevinson C, Davies MJ, et al. Changes in physical activity behaviour and physical function after bariatric surgery: a systematic review and meta-analysis. *Obes Rev*. 2016;17(3):250-261. https://doi.org/10.1111/obr.12361

Jamaly S, Carlsson L, Peltonen M, et al. Surgical obesity treatment and the risk of heart failure. *Eur Heart J*. 2019;40(26):2131-2138. https://doi.org/10.1093/eurheartj/ehz295

Joseph SM, Novak E, Arnold SV, et al. Comparable performance of the Kansas City Cardiomyopathy Questionnaire in patients with heart failure with preserved and reduced ejection fraction. *Circ Heart Fail*. 2013;6(6):1139-1146. https://doi.org/10.1161/CIRCHEARTFAILURE.113.000359

Kitzman DW, Brubaker P, Morgan T, et al. Effect of caloric restriction or aerobic exercise training on peak oxygen consumption and quality of life in obese older patients with heart failure with preserved ejection fraction: a randomized clinical trial. *JAMA*. 2016;315(1):36-46. https://doi.org/10.1001/jama.2015.17346

Kitzman DW, Shah SJ. The HFpEF obesity phenotype: the elephant in the room. *J Am Coll Cardiol*. 2016;68(2):200-203. https://doi.org/10.1016/j.jacc.2016.05.019

Koizumi M, Takada T, Kawarada Y, et al. JPN Guidelines for the management of acute pancreatitis: diagnostic criteria for acute pancreatitis. *J Hepatobiliary Pancreat Surg*. 2006;13(1):25-32. https://doi.org/10.1007/s00534-005-1048-2

Lingvay I, Desouza CV, Lalic KS, et al. A 26-week randomized controlled trial of semaglutide once daily versus liraglutide and placebo in patients with type 2 diabetes suboptimally controlled on diet and exercise with or without metformin. *Diabetes Care*. 2018;41(9):1926-1937. https://doi.org/10.2337/dc17-2381

Mikhalkova D, Holman SR, Jiang H, et al. Bariatric surgery-induced cardiac and lipidomic changes in obesity-related heart failure with preserved ejection fraction. *Obesity (Silver Spring)*. 2018;26(2):284-290. https://doi.org/10.1002/oby.22038

Miller WL, Borlaug BA. Impact of obesity on volume status in patients with ambulatory chronic heart failure. *J Card Fail*. 2020;26(2):112-117. https://doi.org/10.1016/j.cardfail.2019.09.010

Nauck MA, Frossard JL, Barkin JS, et al. Assessment of pancreas safety in the development program of once-weekly GLP-1 receptor agonist dulaglutide. *Diabetes Care*. 2017;40(5):647-654. https://doi.org/10.2337/dc16-0984

Packer M. Epicardial adipose tissue may mediate deleterious effects of obesity and inflammation on the myocardium. *J Am Coll Cardiol*. 2018;71(20):2360-2372. https://doi.org/10.1016/j.jacc.2018.03.509

Pi-Sunyer X, Astrup A, Fujioka K, et al; SCALE Obesity and Prediabetes NN8022-1839 Study Group. A randomized, controlled trial of 3.0 mg of liraglutide in weight management. *N Engl J Med*. 2015;373(1):11-22. https://doi.org/10.1056/NEJMoa1411892

Pocock SJ, Ariti CA, Collier TJ, Wang D. The win ratio: a new approach to the analysis of composite endpoints in clinical trials based on clinical priorities. *Eur Heart J*. 2012;33(2):176-182. https://doi.org/10.1093/eurheartj/ehr352

Rayner JJ, Peterzan MA, Watson WD, et al. Myocardial energetics in obesity: enhanced ATP delivery through creatine kinase with blunted stress response. *Circulation*. 2020;141(14):1152-1163. https://doi.org/10.1161/CIRCULATIONAHA.119.042770

Reddy YN, Rikhi A, Obokata M, et al. Quality of life in heart failure with preserved ejection fraction: importance of obesity, functional capacity, and physical inactivity. *Eur J Heart Fail*. 2020;22(6):1009-1018. https://doi.org/10.1002/ejhf.1788

Regev A, Palmer M, Avigan MI, et al. Consensus: guidelines: best practices for detection, assessment and management of suspected acute drug-induced liver injury during clinical trials in patients with nonalcoholic steatohepatitis. *Aliment Pharmacol Ther*. 2019;49(6):702-713.

Rubin DB. *Multiple Imputation for Nonresponse in Surveys*. John Wiley & Sons, Inc.; 1987.

Shimada YJ, Tsugawa Y, Brown DFM, Hasegawa K. Bariatric surgery and emergency department visits and hospitalizations for heart failure exacerbation: population-based, self-controlled series. *J Am Coll Cardiol*. 2016;67(8):895-903. https://doi.org/10.1016/j.jacc.2015.12.016

Shoemaker MJ, Curtis AB, Vangsnes E, Dickinson MG. Clinically meaningful change estimates for the six-minute walk test and daily activity in individuals with chronic heart failure. *Cardiopulm Phys Ther J*. 2013;24(3):21-29.

Solomon SD, Dobson J, Pocock S, et al.; Candesartan in Heart Failure: Assessment of Reduction in Mortality and Morbidity (CHARM) Investigators. *Circulation*. 2007;116(13):1482-1487. https://doi.org/10.1161/CIRCULATIONAHA.107.696906

Spertus JA, Jones PG, Sandhu AT, Arnold SV. Interpreting the Kansas City Cardiomyopathy Questionnaire in clinical trials and clinical care: JACC state-of-the-art review. *J Am Coll Cardiol*. 2020;76(20):2379-2390. https://doi.org/10.1016/j.jacc.2020.09.542

Steinberg WM, Buse JB, Ghorbani MLM, et al.; LEADER Steering Committee; LEADER Trial Investigators. Amylase, lipase, and acute pancreatitis in people with type 2 diabetes treated with liraglutide: results from the LEADER randomized trial. *Diabetes Care*. 2017a;40(7):966-972. https://doi.org/10.2337/dc16-2747

Steinberg WM, Rosenstock J, Wadden TA, et al. Impact of liraglutide on amylase, lipase, and acute pancreatitis in participants with overweight/obesity and normoglycemia, prediabetes, or type 2 diabetes: secondary analyses of pooled data from the SCALE clinical development program. *Diabetes Care*. 2017b;40(7):839-848. https://doi.org/10.2337/dc16-2684

Sundström J, Bruze G, Ottosson J, et al. Weight loss and heart failure: a nationwide study of gastric bypass surgery versus intensive lifestyle treatment. *Circulation*. 2017;135(17):1577-1585. https://doi.org/10.1161/CIRCULATIONAHA.116.025629

Täger T, Hanholz W, Cebola R, et al. Minimal important difference for 6-minute walk test distances among patients with chronic heart failure. *Int J Cardiol*. 2014;176(1):94-98. https://doi.org/10.1016/j.ijcard.2014.06.035

van der Meer P, Gaggin HK, Dec GW. ACC/AHA versus ESC guidelines on heart failure: JACC guideline comparison. *J Am Coll Cardiol*. 2019;73(21):2756-2768. https://doi.org/10.1016/j.jacc.2019.03.478

Weinberg ME, Bacchetti P, Rushakoff RJ. Frequently repeated glucose measurements overestimate the incidence of inpatient hypoglycemia and severe hyperglycemia. *J Diabetes Sci Technol.* 2010;4(3):577-582. https://doi.org/10.1177/193229681000400311

Wilson JM, Nikooienejad A, Robins DA, et al. The dual glucose-dependent insulinotropic peptide and glucagon-like peptide-1 receptor agonist, tirzepatide, improves lipoprotein biomarkers associated with insulin resistance and cardiovascular risk in patients with type 2 diabetes [published online August 16, 2020]. *Diab Obes and Metab*. https://doi.org/10.1111/dom.14174

Leo Document ID = 7364f020-3ee4-475e-80f0-c92242325670

Approver: PPD

Approval Date & Time: 15-Dec-2021 13:51:04 GMT

Signature meaning: Approved

Approver: PPD

Approval Date & Time: 15-Dec-2021 13:57:21 GMT

Signature meaning: Approved

Title Page

Confidential Information

The information contained in this document is confidential and is intended for the use of clinical investigators. It is the property of Eli Lilly and Company or its subsidiaries and should not be copied by or distributed to persons not involved in the clinical investigation of tirzepatide (LY3298176), unless such persons are bound by a confidentiality agreement with Eli Lilly and Company or its subsidiaries.

Note to Regulatory Authorities: This document may contain protected personal data and/or commercially confidential information exempt from public disclosure. Eli Lilly and Company requests consultation regarding release/redaction prior to any public release. In the United States, this document is subject to Freedom of Information Act (FOIA) Exemption 4 and may not be reproduced or otherwise disseminated without the written approval of Eli Lilly and Company or its subsidiaries.

Protocol Title: A Randomized, Double-Blind, Placebo-Controlled, Phase 3 Study Comparing the Efficacy and Safety of Tirzepatide versus Placebo in Patients with Heart Failure with Preserved Ejection Fraction and Obesity (SUMMIT)

Protocol Number: I8F-MC-GPID

Amendment Number: b

Compound: LY3298176

Study Phase: 3

Short Title: Tirzepatide vs Placebo in Obesity-related HFpEF

Sponsor Name: Eli Lilly and Company

Legal Registered Address: Indianapolis, Indiana, USA 46285

Manufacturer: Eli Lilly and Company

Regulatory Agency Identifier Number

IND: 147352

Approval Date: Protocol Amendment (b) Electronically Signed and Approved by Lilly on date provided below.

Approval Date: 21-Jan-2022 GMT

Medical Monitor Name and Contact Information will be provided separately.

Protocol Amendment Summary of Changes Table

DOCUMENT HISTORY	
**Document**	**Date**
Amendment (a)	*15-Dec-2021*
Original Protocol	*13-Jan-2021*

Amendment a

Overall Rationale for the Amendment:

The purpose of this protocol amendment is to incorporate feedback received from the FDA on exclusion criterion and concomitant therapy.

Section # and Name	Description of Change	Brief Rationale
Section 5.1 Inclusion Criteria	Criterion #7: removed "and/or" wording; added "or" between ARNI and SGLT2is	For clarification
Section 5.2 Exclusion Criteria	Criterion #13: for NYHA Class, added word "or" in sentence to ensure participants with NYHA Class I are excluded from the study<br>Criterion #20: for AF, changed "and" to "or" between "Visit 1" and "Visit 2"	For clarification
Section 6.5 Concomitant Therapy	Removed sentence on use of SGLT2i for treatment of T2DM	Per FDA feedback
Section 7.1.1 Permanent Discontinuation from Study Drug	A sentence inadvertently added and then was removed	For clarification

Table of Contents

1. Protocol Summary 7
1.1. Synopsis 7
1.2. Schema 10
1.3. Schedule of Activities (SoA) 11

2. Introduction 28
2.1. Study Rationale 28
2.2. Background 28
2.3. Benefit/Risk Assessment 29

3. Objectives and Endpoints 30

4. Study Design 33
4.1. Overall Design 33
4.2. Scientific Rationale for Study Design 35
4.2.1. Patient Input into Design 36
4.3. Justification for Dose 36
4.4. End of Study Definition 36

5. Study Population 38
5.1. Inclusion Criteria 38
5.2. Exclusion Criteria 40
5.3. Lifestyle Considerations 42
5.4. Screen Failures 42

6. Study Intervention 44
6.1. Study Interventions Administered 44
6.1.1. Medical Devices 45
6.2. Preparation/Handling/Storage/Accountability 45
6.3. Measures to Minimize Bias: Randomization and Blinding 46
6.4. Study Intervention Compliance 46
6.5. Concomitant Therapy 47
6.6. Dose Modification 49
6.6.1. Temporary Interruption 50
6.6.2. Restarting Study Drug after Interruption 50
6.7. Intervention after the End of the Study 51

7. Discontinuation of Study Intervention and Participant Discontinuation/Withdrawal 52
7.1. Discontinuation of Study Intervention 52
7.1.1. Permanent Discontinuation from Study Drug 52
7.2. Participant Discontinuation/Withdrawal from the Study 53
7.2.1. Inadvertently Enrolled Participants 55
7.3. Lost to Follow up 55

8. Study Assessments and Procedures 56
8.1. Efficacy Assessments 56
8.1.1. Primary Efficacy Assessment 56

8.1.2. Additional Secondary Efficacy Assessments 57
8.1.3. Exploratory Efficacy Assessments 58
8.2. Safety Assessments 59
8.2.1. Physical Examinations 59
8.2.2. Vital Signs 59
8.2.3. Electrocardiograms 59
8.2.4. Clinical Safety Laboratory Assessments 60
8.2.5. Safety Monitoring 60
8.3. Adverse Events, Serious Adverse Events, and Product Complaints 63
8.3.1. Timing and Mechanism for Collecting Events 64
8.3.2. Primary, Secondary, and Additional Study Endpoint Reporting 66
8.3.3. Adverse Events of Special Interest 66
8.4. Treatment of Overdose 67
8.5. Pharmacokinetics 67
8.6. Pharmacodynamics 67
8.7. Genetics 68
8.8. Biomarkers 68
8.9. Immunogenicity Assessments 68
8.10. Medical Resource Utilization and Health Economics 69

9. Statistical Considerations 70
9.1. Statistical Hypotheses 70
9.2. Sample Size Determination 70
9.3. Populations for Analyses 71
9.4. Statistical Analyses 71
9.4.1. General Considerations 71
9.4.2. Primary Endpoint(s) 72
9.4.3. Key Secondary Endpoint(s) 73
9.4.4. Tertiary/Exploratory Endpoint(s) 74
9.4.5. Other Safety Analyses 74
9.4.6. Subgroup Analyses 74
9.5. Interim Analyses 74
9.6. Data Monitoring Committee 75

10. Supporting Documentation and Operational Considerations 76
10.1. Appendix 1: Regulatory, Ethical, and Study Oversight Considerations 76
10.1.1. Regulatory and Ethical Considerations 76
10.1.2. Financial Disclosure 76
10.1.3. Informed Consent Process 77
10.1.4. Data Protection 77
10.1.5. Dissemination of Clinical Study Data 77
10.1.6. Data Quality Assurance 78
10.1.7. Source Documents 79
10.1.8. Study and Site Start and Closure 80
10.1.9. Publication Policy 80
10.1.10. Long-Term Sample Retention 80

10.1.11. Investigator Information 81
10.2. Appendix 2: Clinical Laboratory Tests..... 81
10.3. Appendix 3: Adverse Events: Definitions and Procedures for Recording, Evaluating, Follow-up, and Reporting..... 84
10.3.1. Definition of AE 84
10.3.2. Definition of SAE..... 85
10.3.3. Definition of Product Complaints 87
10.3.4. Recording and Follow-Up of AE and/or SAE and Product Complaints 87
10.3.5. Reporting of SAEs..... 89
10.3.6. Regulatory Reporting Requirements 89
10.4. Appendix 4: Contraceptive Guidance and Collection of Pregnancy Information 90
10.4.1. Male participants: 90
10.4.2. Female participants:..... 90
10.5. Appendix 5: Adverse Events of Special Interest: Definitions and Procedures for Recording, Evaluating, Follow-Up, and Reporting..... 94
10.5.1. Special Safety Topics 94
10.6. Appendix 6: Genetics 99
10.7. Appendix 7: Recommended Laboratory Testing for Hypersensitivity Events 100
10.8. Appendix 8: Liver Safety: Suggested Actions and Follow-Up Assessments 102
10.9. Appendix 9: Medical Device Adverse Events (AEs), Adverse Device Effects (ADEs), Serious Adverse Events (SAEs) and Device Deficiencies: Definition and Procedures for Recording, Evaluating, Follow-Up, and Reporting..... 104
10.10. Appendix 10: Six-Minute Walk Test Screening Procedures 105
10.10.1. Screening Procedures and Flow Diagram..... 105
10.11. Appendix 11: New York Heart Association Classification of Heart Failure..... 106
10.12. Appendix 12: Abbreviations 107
11. References 112

1. Protocol Summary

1.1. Synopsis

Protocol Title: A Randomized, Double-Blind, Placebo-Controlled, Phase 3 Study Comparing the Efficacy and Safety of Tirzepatide versus Placebo in Patients with Heart Failure with Preserved Ejection Fraction and Obesity (SUMMIT)

Short Title: Tirzepatide vs Placebo in Obesity-related HFpEF

Rationale:

Heart failure with preserved ejection fraction is a heterogenous clinical syndrome resulting from various pathophysiological processes. Among the broad spectrum of HFpEF clinical presentation, obesity-related HFpEF displays a distinct phenotype where increased visceral and ectopic adiposity as well as volume expansion plays a causal role (Kitzman and Shah 2016; Packer 2018; Miller and Borlaug 2020). Given tirzepatide's anti-inflammatory and antifibrotic effects and a reduction in circulating plasma volume as a consequence of the treatment of obesity, tirzepatide may provide clinical benefit to patients with HFpEF and BMI ≥30 kg/m^2.

Study I8F-MC-GPID, also known as SUMMIT, is a Phase 3, randomized, multicenter, international, placebo-controlled, double-blinded, parallel-arm study. This study will evaluate the effect of SC QW injection of tirzepatide, MTD up to 15 mg, on the risk of death, hospitalization or emergency care due to HF, exercise capacity, and health status in participants with HFpEF and BMI ≥30 kg/m^2.

Objectives and Endpoints

Objectives	Endpoints
Primary	
To demonstrate that a maximally tolerated tirzepatide dose up to 15 mg administered subcutaneously once weekly (SC QW) is superior to placebo based on the hierarchal composite endpoint in participants with HFpEF and BMI ≥30 kg/m^2.	A hierarchical composite of the following:<br>1. Time to all-cause mortality through the end of the treatment period<br>2. Occurrence of heart failure (HF) events through end of the treatment period, where HF events include HF hospitalization OR urgent HF visit (adjudicated)<br>o number of HF events<br>o time to first HF events<br>3. Change from baseline in the 6-minute walk test distance (6MWD) category at Week 52

	4. Change from baseline in the Kansas City Cardiomyopathy Questionnaire (KCCQ) Clinical Summary Scale (CSS) category at Week 52<br><br>The categories for change from baseline in the 6MWD are: 1) ≥30% worsening; 2) ≥20% and <30% worsening; 3) ≥10% and <20% worsening; 4) No change (<10% change); 5) ≥10% and <20% improvement; 6) ≥20% and <30% improvement; and 7) ≥30% improvement.<br><br>The categories for change from baseline in the KCCQ-CSS are: 1) ≥10-point worsening; 2) ≥5- but <10-point worsening; 3) No change (<5-point change); 4) ≥5- but <10-point improvement; and 5) ≥10-point improvement.
To demonstrate that a maximally tolerated tirzepatide dose up to 15 mg administered SC QW is superior to placebo to improve exercise capacity in participants with HFpEF and BMI ≥30 kg/m^2.	Change from baseline to Week 52 in exercise capacity as measured 6MWD
Key Secondary (multiplicity controlled)	
Long-term weight loss	Percent change from baseline to Week 52 in body weight loss
Patient-reported symptoms and physical limitations	Change from baseline to Week 52 in the KCCQ CSS
Exercise capacity	Change from baseline to Week 24 in 6MWD
New York Heart Association (NYHA) Class	Proportion of participants with NYHA Class change at Week 52

Abbreviations: BMI = body mass index; HFpEF = heart failure with preserved ejection fraction.

Overall Design

Study GPID is a randomized, outpatient, multicenter, international, placebo-controlled, double-blind, parallel-arm, Phase 3 study with 2 study periods. The study is designed to evaluate the efficacy and safety of SC QW tirzepatide, MTD up to 15 mg, compared to placebo, in participants with HFpEF and obesity.

Two intervention groups will be studied:

- Tirzepatide, MTD up to 15 mg, SC QW
- Placebo

The starting dose of tirzepatide is 2.5 mg QW, which is to be escalated at 4-week intervals to a maximum of 15 mg QW or to the highest maintenance dose tolerated by the participant (see Section 6).

The maximum duration of participation depends on when the last participant completes 52 weeks of treatment.

Disclosure Statement: This is a parallel-treatment study with 2 intervention groups that is double blinded.

Number of Participants:

Approximately 700 participants will be randomly assigned to study drug with approximately 350 participants per intervention group.

Intervention Groups and Duration:

The study will compare treatment with tirzepatide and treatment with placebo. Assignment to tirzepatide or placebo groups will be randomly allocated in a 1:1 ratio.

The starting dose of tirzepatide 2.5 mg QW is to be escalated to 15 mg QW or the highest maintenance dose tolerated by the participant (5 mg, 10 mg, QW).

Study participation for each participant is sectioned into the following study periods:

- Study Period 1: screening period, approximately 6 weeks and not more than 12 weeks
- Study Period 2: treatment period, at least 52 weeks

The study will continue until approximately 52 weeks after the last participant is randomized. The maximum duration of an individual's participation is estimated to be 120 weeks and will depend on duration of study enrollment.

Data Monitoring Committee: Yes

1.2. Schema

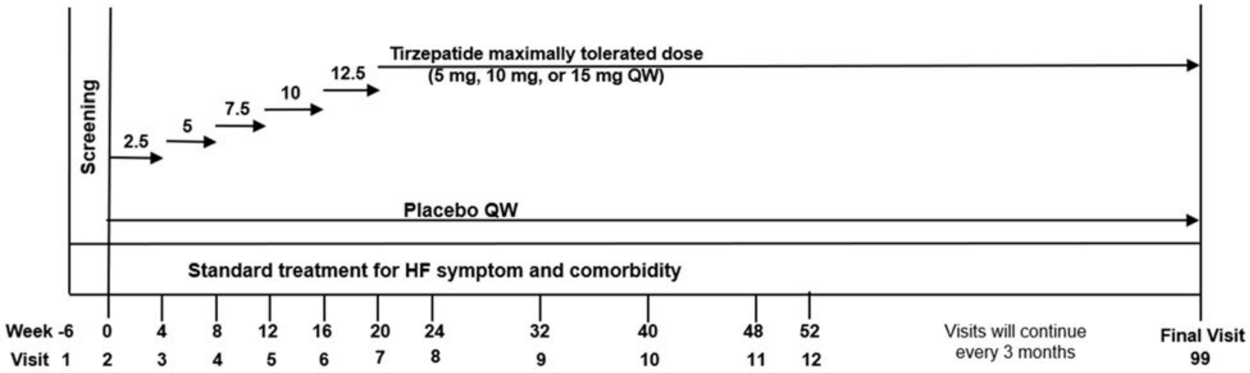

Abbreviations: HF = heart failure; QW = weekly.

Note: Screening procedures may take longer or shorter than 6 weeks but no more than 12 weeks.

1.3. Schedule of Activities (SoA)

Visit 1 and 2 procedures may be conducted over more than 1 day each as long as all activities are completed within the allowable visit interval tolerance for each visit.

For early terminations (ET) from the study that occur before the final visit (V99) in treatment period, see the activities listed for ET in this table.

Study I8F-MC-GPID	Study Period I Screening	Study Period II - Treatment period																		
														Unscheduled Visit (UV)[a]						
Visit number	1	2	3	4	5	6	7	8	9	10	11	12	EVa, EVb, EVc (13, 14, 15, 17, 18, 19, etc.)	EVd (16, 20, etc.)	UV	Dosing UV	Phone Follow-Up Dosing UV	ET	99	Comment
Weeks from randomization	-6	0	4	8	12	16	20	24	32	40	48	52	(+3, 6, 9, 15, 18, 21 months)	(+12, 24 months)				—	Final Visit	See footnote b
Visit interval tolerance (days)			±3	±3	±3	±3	±3	±3	±7	±7	±7	±7	±15	±15				—		
Telephone Visit									X		X		X				X			See footnote c
Informed consent	X																			
Inclusion and exclusion criteria, review and confirm	X	X																		
Demographics	X																			
Preexisting conditions and medical history, including relevant surgical history	X																			

Study I8F-MC-GPID	Study Period I Screening	Study Period II - Treatment period																		
														Unscheduled Visit (UV)[a]						
Visit number	1	2	3	4	5	6	7	8	9	10	11	12	EVa, EVb, EVc (13, 14, 15, 17, 18, 19, etc.)	EVd (16, 20, etc.)	UV	Dosing UV	Phone Follow-Up Dosing UV	ET	99	Comment
Weeks from randomization	-6	0	4	8	12	16	20	24	32	40	48	52	(+3, 6, 9, 15, 18, 21 months)	(+12, 24 months)				—	Final Visit	See footnote b
Visit interval tolerance (days)			±3	±3	±3	±3	±3	±3	±7	±7	±7	±7	±15	±15				—		
Telephone Visit									X		X		X				X			See footnote c
Prespecified medical history (indication and history of interest)	X																			Includes HF history, hospitalization for HF, CVD, MI, atrial fibrillation, stroke, CV risk (T2DM, HTN, dyslipidemia, metabolic syndrome)
Prior treatments for HFpEF	X																			
Substance use (alcohol, tobacco use)	X																			
Concomitant medications	X	X	X	X	X	X	X	X	X	X	X	X	X	X	X	X	X	X	X	
Adverse events (AEs)	X	X	X	X	X	X	X	X	X	X	X	X	X	X	X	X	X	X	X	Including Product Complaints
**Physical Evaluation**																				
Height	X																			

Study I8F-MC-GPID	Study Period I Screening	Study Period II - Treatment period																	Comment
														Unscheduled Visit (UV)[a]					
Visit number	1	2	3	4	5	6	7	8	9	10	11	12	EVa, EVb, EVc (13, 14, 15, 17, 18, 19, etc.)	EVd (16, 20, etc.)	UV	Dosing UV	Phone Follow-Up Dosing UV	ET	99	Comment
Weeks from randomization	-6	0	4	8	12	16	20	24	32	40	48	52	(+3, 6, 9, 15, 18, 21 months)	(+12, 24 months)				—	Final Visit	See footnote b
Visit interval tolerance (days)			±3	±3	±3	±3	±3	±3	±7	±7	±7	±7	±15	±15				—		
Telephone Visit									X		X		X				X			See footnote c
Weight	X	X	X	X	X	X	X	X		X		X		X	X	X		X	X	
Waist circumference	X	X						X				X		X				X	X	
Vital Signs	X	X	X	X	X	X	X	X		X		X		X	X	X		X	X	Include 2 Sitting BP and HR. HR to be performed by apical auscultation. Vital signs should be collected prior to the first 6MWT of the day and before ECG.
Physical examination	X											X		X				X		
Symptom-directed physical examination		X	X	X	X	X	X	X		X		X		X	X	X			X	As indicated based on participant status and standard of care, including dyspnea, orthopnea, paroxysmal nocturnal dyspnea (PND), edema, jugular venous distension (JVD), rales

Study I8F-MC-GPID	Study Period I Screening	Study Period II - Treatment period																		
														Unscheduled Visit (UV)[a]						
Visit number	1	2	3	4	5	6	7	8	9	10	11	12	EVa, EVb, EVc (13, 14, 15, 17, 18, 19, etc.)	EVd (16, 20, etc.)	UV	Dosing UV	Phone Follow-Up Dosing UV	ET	99	Comment
Weeks from randomization	-6	0	4	8	12	16	20	24	32	40	48	52	(+3, 6, 9, 15, 18, 21 months)	(+12, 24 months)				—	Final Visit	See footnote b
Visit interval tolerance (days)			±3	±3	±3	±3	±3	±3	±7	±7	±7	±7	±15	±15				—		
Telephone Visit									X		X		X				X			See footnote c
NYHA class assessment	X	X						X				X		X				X[d]		NYHA class assessment must be performed by an independent assessor. [d]Perform at ET only if participant early terminates at or prior to Week 52.
HF events		X	X	X	X	X	X	X	X	X	X	X	X	X	X	X		X	X	
Evaluation of injection site reactions		X	X	X	X	X	X	X		X		X		X	X	X		X	X	
Single-read 12-lead ECG	X	X						X				X						X[d]		Collect locally. Report atrial fibrillation or other abnormalities on the eCRF. Optional ECG is allowable if indicated. [d]Perform at ET only if participant early terminates at or prior to Week 52.

Study I8F-MC-GPID	Study Period I Screening	Study Period II - Treatment period																	
														Unscheduled Visit (UV)[a]					
Visit number	1	2	3	4	5	6	7	8	9	10	11	12	EVa, EVb, EVc (13, 14, 15, 17, 18, 19, etc.)	EVd (16, 20, etc.)	UV	Dosing UV	Phone Follow-Up Dosing UV	ET	99	Comment
Weeks from randomization	-6	0	4	8	12	16	20	24	32	40	48	52	(+3, 6, 9, 15, 18, 21 months)	(+12, 24 months)				—	Final Visit	See footnote b
Visit interval tolerance (days)			±3	±3	±3	±3	±3	±3	±7	±7	±7	±7	±15	±15				—		
Telephone Visit									X		X		X				X			See footnote c
Echocardiography	X																			For those required to complete the ECHO examination
Dilated fundoscopic examination	X																			Perform for participants with T2DM who have not had an evaluable dilated fundoscopic examination in the last 12 months. See exclusion criterion 25 (Section 5.2)<br>Follow-up dilated fundoscopic examination should be performed when clinically indicated by any AE suspected of worsening retinopathy.

Study I8F-MC-GPID	Study Period I Screening	Study Period II - Treatment period																		
														Unscheduled Visit (UV)[a]						
Visit number	1	2	3	4	5	6	7	8	9	10	11	12	EVa, EVb, EVc (13, 14, 15, 17, 18, 19, etc.)	EVd (16, 20, etc.)	UV	Dosing UV	Phone Follow-Up Dosing UV	ET	99	Comment
Weeks from randomization	-6	0	4	8	12	16	20	24	32	40	48	52	(+3, 6, 9, 15, 18, 21 months)	(+12, 24 months)				—	Final Visit	See footnote b
Visit interval tolerance (days)			±3	±3	±3	±3	±3	±3	±7	±7	±7	±7	±15	±15				—		
Telephone Visit									X		X		X				X			See footnote c
6MWT	X[e]	X[f]						X				X						X[d]		Ensure that participant completes the associated Borg Questionnaire prior to and after the 6MWT. [e]Two 6MWTs conducted at screening visit. [f]See Section 10.10.1 to determine if the 6MWT needs to be repeated for Visit 2. [d]Perform at ET only if participant early terminates at or prior to Week 52.
**Participant Education**																				
Diary instruction		X																		
**Participant Diary**																				

Study I8F-MC-GPID	Study Period I Screening	Study Period II - Treatment period																		
														Unscheduled Visit (UV)[a]						
Visit number	1	2	3	4	5	6	7	8	9	10	11	12	EVa, EVb, EVc (13, 14, 15, 17, 18, 19, etc.)	EVd (16, 20, etc.)	UV	Dosing UV	Phone Follow-Up Dosing UV	ET	99	Comment
Weeks from randomization	-6	0	4	8	12	16	20	24	32	40	48	52	(+3, 6, 9, 15, 18, 21 months)	(+12, 24 months)				—	Final Visit	See footnote b
Visit interval tolerance (days)			±3	±3	±3	±3	±3	±3	±7	±7	±7	±7	±15	±15				—		
Telephone Visit									X		X		X				X			See footnote c
Participant diary dispensed		X	X	X	X	X	X	X		X		X		X						
Diary compliance check/Assess study drug compliance[g]			X	X	X	X	X	X	X	X	X	X	X	X				X	X	
Diary return			X	X	X	X	X	X		X		X		X				X	X	
**Patient-Reported Outcomes (PROs) (Electronic[h]**																				[h]Perform PRO at ET only if participant early terminates at or prior to Week 52.
Kansas City Cardiomyopathy Questionnaire (KCCQ)	X	X						X				X						X		
EQ-5D-5L		X						X				X						X		

Study I8F-MC-GPID	Study Period I Screening	Study Period II - Treatment period																		
														Unscheduled Visit (UV)[a]						
Visit number	1	2	3	4	5	6	7	8	9	10	11	12	EVa, EVb, EVc (13, 14, 15, 17, 18, 19, etc.)	EVd (16, 20, etc.)	UV	Dosing UV	Phone Follow-Up Dosing UV	ET	99	Comment
Weeks from randomization	-6	0	4	8	12	16	20	24	32	40	48	52	(+3, 6, 9, 15, 18, 21 months)	(+12, 24 months)				—	Final Visit	See footnote b
Visit interval tolerance (days)			±3	±3	±3	±3	±3	±3	±7	±7	±7	±7	±15	±15				—		
Telephone Visit									X		X		X				X			See footnote c
Patient Global Impression of Status – Overall (PGIS-Overall)	X	X						X				X						X		
Patient Global Impression of Status – Physical Function (PGIS-Physical Function)	X	X						X				X						X		
Patient Global Impression of Status – Symptom Severity (PGIS-Symptom Severity)	X	X						X				X						X		
**Laboratory Tests and Sample Collections**																				
Hematology	X	X			X			X				X		X	X[i]	X[i]		X	X	See footnote i
Hemoglobin A1c (HbA1c)	X	X	X	X	X	X	X	X		X		X		X				X	X	

Study I8F-MC-GPID	Study Period I Screening	Study Period II - Treatment period																		
														Unscheduled Visit (UV)[a]						
Visit number	1	2	3	4	5	6	7	8	9	10	11	12	EVa, EVb, EVc (13, 14, 15, 17, 18, 19, etc.)	EVd (16, 20, etc.)	UV	Dosing UV	Phone Follow-Up Dosing UV	ET	99	Comment
Weeks from randomization	-6	0	4	8	12	16	20	24	32	40	48	52	(+3, 6, 9, 15, 18, 21 months)	(+12, 24 months)				—	Final Visit	See footnote b
Visit interval tolerance (days)			±3	±3	±3	±3	±3	±3	±7	±7	±7	±7	±15	±15				—		
Telephone Visit									X		X		X				X			See footnote c
Clinical chemistry (with glucose)	X	X[j]			X			X				X		X	X[i]	X[i]		X	X	[j]Required at Visit 2 randomization if screening labs are older than 28 days for chemistry, lipid, and pancreatic lipase/amylase[i]
Lipid panel	X	X[j]			X			X				X		X	X[i]	X[di]		X	X	[j]Required at Visit 2 randomization if screening labs are older than 28 days for chemistry, lipid, and pancreatic lipase/amylase
Thyroid-stimulating hormone (TSH)	X																			
Serum pregnancy	X	X[k]																		For women of childbearing potential only. See Appendix 4 (Section 10.4). [k]Collect serum pregnancy at Visit 2 only if Visit 1 serum was ≥28 days prior.

Study I8F-MC-GPID	Study Period I Screening	Study Period II - Treatment period																		
															Unscheduled Visit (UV)[a]					
Visit number	1	2	3	4	5	6	7	8	9	10	11	12	EVa, EVb, EVc (13, 14, 15, 17, 18, 19, etc.)	EVd (16, 20, etc.)	UV	Dosing UV	Phone Follow-Up Dosing UV	ET	99	Comment
Weeks from randomization	-6	0	4	8	12	16	20	24	32	40	48	52	(+3, 6, 9, 15, 18, 21 months)	(+12, 24 months)				—	Final Visit	See footnote b
Visit interval tolerance (days)			±3	±3	±3	±3	±3	±3	±7	±7	±7	±7	±15	±15				—		
Telephone Visit									X		X		X				X			See footnote c
Urine pregnancy (local)		X	X	X	X	X	X	X		X		X		X				X	X	A local urine pregnancy test must be performed at Visit 2 after patient eligibility has been confirmed with the result available prior to randomization and first injection of study drug(s) for WOCBP only. Additional local urine pregnancy tests may be performed at the investigator's discretion during the study. If required per local regulations and/or

Study I8F-MC-GPID	Study Period I Screening	Study Period II - Treatment period																		
														Unscheduled Visit (UV)[a]						
Visit number	1	2	3	4	5	6	7	8	9	10	11	12	EVa, EVb, EVc (13, 14, 15, 17, 18, 19, etc.)	EVd (16, 20, etc.)	UV	Dosing UV	Phone Follow-Up Dosing UV	ET	99	Comment
Weeks from randomization	-6	0	4	8	12	16	20	24	32	40	48	52	(+3, 6, 9, 15, 18, 21 months)	(+12, 24 months)				—	Final Visit	See footnote b
Visit interval tolerance (days)			±3	±3	±3	±3	±3	±3	±7	±7	±7	±7	±15	±15				—		
Telephone Visit									X		X		X				X			See footnote c
																				institutional guidelines, pregnancy testing can also occur at other times during the study treatment period. See Appendix 4 (Section 10.4).
Follicle-stimulating hormone (FSH)	X																			Collect FSH only in women whose menopausal status needs to be determined. For participants known to be either premenopausal or postmenopausal, these tests do not need to be collected
NT-proBNP	X	X			X			X				X						X[d]		[d]Perform at ET only if participant early terminates at or prior to Week 52.

Study I8F-MC-GPID	Study Period I Screening	Study Period II - Treatment period																			
														Unscheduled Visit (UV)[a]							
Visit number	1	2	3	4	5	6	7	8	9	10	11	12	EVa, EVb, EVc (13, 14, 15, 17, 18, 19, etc.)	EVd (16, 20, etc.)	UV	Dosing UV	Phone Follow-Up Dosing UV	ET	99	Comment
Weeks from randomization	-6	0	4	8	12	16	20	24	32	40	48	52	(+3, 6, 9, 15, 18, 21 months)	(+12, 24 months)				—	Final Visit	See footnote b
Visit interval tolerance (days)			±3	±3	±3	±3	±3	±3	±7	±7	±7	±7	±15	±15				—		
Telephone Visit									X		X		X				X			See footnote c
Cardiac troponin T (cTnT)		X			X			X				X						X[d]		[d]Perform at ET only if participant early terminates at or prior to Week 52.
Calcitonin	X							X				X		X				X	X	
Cystatin C		X			X			X				X						X[d]		[d]Perform at ET only if participant early terminates at or prior to Week 52.
C-reactive protein, high-sensitivity (hs-CRP)		X			X			X				X						X[d]		[d]Perform at ET only if participant early terminates at or prior to Week 52.
Pancreatic amylase	X	X[j]			X			X		X		X		X				X	X	[j]Required at Visit 2 randomization if screening labs are older than 28 days for chemistry, lipid, and pancreatic lipase/amylase

Study I8F-MC-GPID	Study Period I Screening	Study Period II - Treatment period																		
														Unscheduled Visit (UV)[a]						
Visit number	1	2	3	4	5	6	7	8	9	10	11	12	EVa, EVb, EVc (13, 14, 15, 17, 18, 19, etc.)	EVd (16, 20, etc.)	UV	Dosing UV	Phone Follow-Up Dosing UV	ET	99	Comment
Weeks from randomization	-6	0	4	8	12	16	20	24	32	40	48	52	(+3, 6, 9, 15, 18, 21 months)	(+12, 24 months)				—	Final Visit	See footnote b
Visit interval tolerance (days)			±3	±3	±3	±3	±3	±3	±7	±7	±7	±7	±15	±15				—		
Telephone Visit									X		X		X				X			See footnote c
Lipase	X	X[j]			X			X		X		X		X				X	X	[j]Required at Visit 2 randomization if screening labs are older than 28 days for chemistry, lipid, and pancreatic lipase/amylase
eGFR (CKD-EPI)	X	X[l]			X			X				X		X				X	X	The CKD-EPI equation will be used by the central lab to estimate and report eGFR. [l]Only collect calculated eGFR with Clinical Chemistry sample if screening labs are older than 28 days.
Urinary albumin/creatinine ratio (UACR)	X							X				X		X				X	X	

Study I8F-MC-GPID	Study Period I Screening	Study Period II - Treatment period																		
														Unscheduled Visit (UV)[a]						
Visit number	1	2	3	4	5	6	7	8	9	10	11	12	EVa, EVb, EVc (13, 14, 15, 17, 18, 19, etc.)	EVd (16, 20, etc.)	UV	Dosing UV	Phone Follow-Up Dosing UV	ET	99	Comment
Weeks from randomization	-6	0	4	8	12	16	20	24	32	40	48	52	(+3, 6, 9, 15, 18, 21 months)	(+12, 24 months)				—	Final Visit	See footnote b
Visit interval tolerance (days)			±3	±3	±3	±3	±3	±3	±7	±7	±7	±7	±15	±15				—		
Telephone Visit									X		X		X				X			See footnote c
Pharmacokinetic (PK) samples		X	X		X			X				X		X				X	X	PK samples should be taken prior to dose administration at the visit and at the same time as immunogenicity samples.
Immunogenicity samples		X	X		X			X				X		X				X	X	
**Stored Samples**																				
Genetics sample		X																		
Exploratory biomarker samples		X			X			X				X								
**Randomization and Dosing**																				
Randomization		X																		
Dispense study drug		X	X	X	X	X	X	X		X		X	X	X		X				

Study I8F-MC-GPID	Study Period I Screening	Study Period II - Treatment period																		
														Unscheduled Visit (UV)[a]						
Visit number	1	2	3	4	5	6	7	8	9	10	11	12	EVa, EVb, EVc (13, 14, 15, 17, 18, 19, etc.)	EVd (16, 20, etc.)	UV	Dosing UV	Phone Follow-Up Dosing UV	ET	99	Comment
Weeks from randomization	-6	0	4	8	12	16	20	24	32	40	48	52	(+3, 6, 9, 15, 18, 21 months)	(+12, 24 months)				—	Final Visit	See footnote b
Visit interval tolerance (days)			±3	±3	±3	±3	±3	±3	±7	±7	±7	±7	±15	±15				—		
Telephone Visit									X		X		X				X			See footnote c
Injection training with autoinjector demonstration device		X																		
Observe participant administer study drug		X	X	X	X	X	X	X		X		X		X		X				Participants should administer the first dose of study drug at Visit 2 after study procedures and randomization have been completed. Review technique with the participant at each in clinic visit.<br>Sites should coach and oversee if participants self-administer study drug at a scheduled visit.

Study I8F-MC-GPID	Study Period I Screening	Study Period II - Treatment period																		
														Unscheduled Visit (UV)[a]						
Visit number	1	2	3	4	5	6	7	8	9	10	11	12	EVa, EVb, EVc (13, 14, 15, 17, 18, 19, etc.)	EVd (16, 20, etc.)	UV	Dosing UV	Phone Follow-Up Dosing UV	ET	99	Comment
Weeks from randomization	-6	0	4	8	12	16	20	24	32	40	48	52	(+3, 6, 9, 15, 18, 21 months)	(+12, 24 months)				—	Final Visit	See footnote b
Visit interval tolerance (days)			±3	±3	±3	±3	±3	±3	±7	±7	±7	±7	±15	±15				—		
Telephone Visit									X		X		X				X			See footnote c
Dispense ancillary supplies to participant		X	X	X	X	X	X	X		X				X						
Participant returns study drugs and injection supplies			X	X	X	X	X	X		X		X		X				X	X	

Abbreviations: 6MWT = 6-minute walk test; BP = blood pressure; CKD-EPI = Chronic Kidney Disease Epidemiology Collaboration; CVD = cardiovascular disease; ECG = electrocardiogram; ECHO = echocardiography; eCRF = electronic case report form; eGFR = estimated glomerular filtration rate; EQ-5D-5L = 5-Level European Quality of Life Questionnaire; ET = Early Termination; EV = Extended Visit; HF = heart failure; HFpEF = heart failure with preserved ejection fraction; HR = heart rate; HTN = hypertension; MI = myocardial infarction; NT-proBNP = N-terminal pro b-type natriuretic peptide; NYHA = New York Heart Association; T2DM = type 2 diabetes mellitus; WOCBP = women of childbearing potential.

[a] There are 3 types of Unscheduled Visits (UV): These visits can occur during the dose-escalation period and maintenance period.

- Unscheduled Visits (UV) – Unscheduled Visit per investigator discretion. All activities with a check mark in UV visit are required.
- Dosing Unscheduled Visit (UV) – these visits will include dose re-escalation. The study drug must be restarted when it is safe to do so and only while at the clinic visit. The subsequent unscheduled dosing visits will be scheduled every 4 weeks (±7 days) until maximum tolerated dose is achieved.
- Phone Follow-up Dosing Unscheduled Visit (UV) – these visits are optional phone visits.

[b] At least 52 weeks (Visit 12) of treatment are planned. Additional visits after Visit 12 will occur every 3 months. The visits occurring after Visit 12 will follow the Extended Maintenance Visit schedule in sequence (EVa, EVb, EVc, and EVd) then repeat.

[c] Telephone visits can become office visits. Site documentation will serve as the source for telephone visits. Additional, optional telephone visits may be conducted at investigator discretion.

[g] This includes glucose monitoring for participants with T2D, weekly study drug injection.

[i] Unscheduled Visits: laboratory tests may be drawn according to investigator discretion. If only re-test labs required, site is not required to complete an unscheduled visit and associated procedures.

2. Introduction

2.1. Study Rationale

Heart failure with preserved ejection fraction is a heterogenous clinical syndrome resulting from various pathophysiological processes. Among the broad spectrum of HFpEF clinical presentation, obesity-related HFpEF displays a distinct phenotype where increased visceral and ectopic adiposity as well as volume expansion plays a causal role (Kitzman and Shah 2016; Packer 2018; Miller and Borlaug 2020). Given tirzepatide's potential to decrease inflammation and fibrosis and a to reduce circulating plasma volume as a consequence of the treatment of obesity, tirzepatide may provide clinical benefit to patients with HFpEF and BMI ≥30 kg/m^2.

Study I8F-MC-GPID, also known as SUMMIT, is a Phase 3, randomized, multicenter, international, placebo-controlled, double-blinded, parallel-arm study. This study will evaluate the effect of SC QW injection of tirzepatide, MTD up to 15 mg, on the risk of death, hospitalization or emergency care due to HF, exercise capacity, and health status in participants with HFpEF and BMI ≥30 kg/m^2.

2.2. Background

There is a significant unmet need in treatment of patients with HFpEF. Tirzepatide, a GIP and GLP-1 dual agonist, has the potential to provide benefit to patients with HFpEF and BMI ≥30 kg/m^2 with not only improvements in exercise capacity and symptoms but also a reduction in HF events and/or increased survival. Supporting a causal association between obesity and HFpEF, bariatric surgery improved NYHA class, patient-reported outcomes, and echo parameters (LV wall thickness, LV relaxation) in patients with HFpEF and obesity (Mikhalkova et al. 2018). A meta-analysis of bariatric surgery also showed improvement in functional capacity 6 to 12 months after surgery in patients with obesity (Herring et al. 2016). In patients with HFpEF and obesity, diet-induced weight loss (Δ=−7 kg, 20 weeks) significantly improved symptoms (KCCQ) and exercise capacity (6MWD and peak oxygen uptake) (Kitzman et al. 2016). Furthermore, weight reduction is proven to be effective in reducing HF risk and HF hospitalizations. A large observational study has demonstrated a 62% decrease (over 8 years) of HF incidence after bariatric surgery in patients with T2DM (Aminian et al. 2019). The reduction of HF risk after bariatric surgery has been consistently demonstrated in broader patient populations and considered to be mediated by weight loss with a hazard ratio for a 10-kg weight loss being 0.77 (Sundström et al. 2017; Jamaly et al. 2019). Moreover, a self-controlled case study showed a 29% (0 to 12 months) and a 43% (13 to 24 months) risk reduction of HF events in patients with HFpEF after bariatric surgery (Shimada et al. 2016).

It has been demonstrated that tirzepatide can provide significant body weight loss and improvement of lipid and glucose metabolism in patients with T2DM (Frias et al. 2018; Wilson et al. 2020). It is known that the body weight reduction with GLP-1 RAs in patients without T2DM is higher than in patients with T2DM (Davies et al. 2015; Pi-Sunyer et al. 2015; Lingvay et al. 2018). If Study GPID is assumed to include 40% to 50% of patients with T2DM, the mean placebo-adjusted body weight percent reduction that tirzepatide can provide in 52 weeks in this study is estimated to be 15% to 16%. This is based on tirzepatide clinical data and the

understanding of body weight loss differences between patients with and without T2DM treated with GLP-1 RAs. Thus, the predicted body weight loss with treatment with tirzepatide is close to that shown with bariatric surgery. In addition, tirzepatide may provide benefit to patients with HFpEF and obesity by virtue of cardiometabolic improvements (Wilson et al. 2020). Given the wide distribution of GIP receptor in the adipose tissue, GIP is thought to be actively involved in lipid and glucose metabolism.

Therefore, it will be meaningful to assess HF clinical outcomes in addition to the functional and symptomatic endpoints in this study, thereby facilitating the understanding of the clinical benefit of tirzepatide treatment in patients with HFpEF and obesity.

2.3. Benefit/Risk Assessment

More detailed information about the known and expected benefits and risks and reasonably expected AEs of tirzepatide may be found in the Investigator's Brochure.

3. Objectives and Endpoints

Objectives	Endpoints
Primary	
To demonstrate that a maximally tolerated tirzepatide dose up to 15 mg administered subcutaneously once weekly (SC QW) is superior to placebo based on the hierarchal composite endpoint in participants with HFpEF and BMI ≥30 kg/m^2.	A hierarchical composite of the following:<br>1. Time to all-cause mortality through the end of the treatment period<br>2. Occurrence of heart failure (HF) events through end of the treatment period, where HF events include HF hospitalization OR urgent HF visit (adjudicated)<br>o number of HF events<br>o time to first HF event<br>3. Change from baseline in the 6-minute walk test distance (6MWD) category at Week 52<br>4. Change from baseline in the Kansas City Cardiomyopathy Questionnaire (KCCQ) Clinical Summary Score (CSS) category at Week 52<br>The categories for change from baseline in the 6MWD are: 1) ≥30% worsening; 2) ≥20% and <30% worsening; 3) ≥10% and <20% worsening; 4) No change (<10% change); 5) ≥10% and <20% improvement; 6) ≥20% and <30% improvement; and 7) ≥30% improvement.<br>The categories for change from baseline in the KCCQ-CSS are: 1) ≥10-point worsening; 2) ≥5- but <10-point worsening; 3) No change (<5-point change); 4) ≥5- but <10-point improvement; and 5) ≥10-point improvement.
To demonstrate that a maximally tolerated tirzepatide dose up to 15 mg administered SC QW is superior to placebo to improve exercise capacity in participants with HFpEF and BMI ≥30 kg/m^2.	Change from baseline to Week 52 in exercise capacity as measured by 6MWD

Key Secondary (multiplicity controlled)	
Long-term weight loss	Percent change from baseline to Week 52 in body weight loss
Patient-reported symptoms and physical limitations	Change from baseline to Week 52 in the KCCQ CSS
Exercise capacity	Change from baseline to Week 24 in 6MWD
New York Heart Association (NYHA) Class	Proportion of participants with NYHA Class change at Week 52
Other Secondary	
Clinical outcome events of HF	• Time to first occurrence of HF events or CV death<br>• Time to first occurrence of HF events or all-cause death<br>• Time to first occurrence of HF events<br>• Time to recurrent events of HF events and CV death<br>• Time to recurrent events of HF events
Exploratory	
HF medication use	Change in the HF concomitant medication net use
Atrial fibrillation	Proportion of participants with atrial fibrillation
Waist circumference	Change from baseline (centimeters)
Patient-reported health-related quality of life	Change from baseline in KCCQ:<br>• Total Symptom Score (TSS)<br>• Overall Summary Score (OSS)
Patient-reported health status	Change from baseline in EQ-5D-5L:<br>o Index Score<br>o VAS Score

Patient-reported global health status	Proportion of participants with improvements in global health status from baseline as assessed by the PGIS-Overall
Patient-reported global impression of physical function	Proportion of participants with improvements in physical function from baseline as assessed by the PGIS-Physical Function
Patient-reported global symptom severity	Proportion of participants with improvements in symptom severity from baseline as assessed by the PGIS-Symptom Severity
Evaluation of prespecified biomarkers	• NT-proBNP<br>• cTnT<br>• hs-CRP

Abbreviations: BMI = body mass index; cTnT = cardiac troponin T; HFpEF = heart failure with preserved ejection fraction; hsCRP = C-reactive protein, high-sensitivity; NT-proBNP = N-terminal pro b-type natriuretic peptide; PGIS = Patient-Reported Global Impression of Status; VAS = visual analog scale.

4. Study Design

4.1. Overall Design

Study GPID is a randomized, outpatient, multicenter, international, placebo-controlled, double-blinded, parallel-arm, Phase 3 study with 2 study periods. The study is designed to evaluate the efficacy and safety of SC QW tirzepatide, MTD up to 15 mg, compared to placebo, in participants with HFpEF and obesity.

Two intervention groups will be studied:

- Tirzepatide MTD up to 15 mg SC QW
- Placebo

The study will compare treatment with tirzepatide and treatment with placebo. Assignment to tirzepatide or placebo groups will be randomly allocated in a 1:1 ratio

The starting dose of tirzepatide or placebo is 2.5 mg QW, which is to be escalated at 4-week intervals to a maximum of 15 mg QW or to the highest maintenance dose tolerated by the participant (see Section 6).

The study will consist of 2 periods:

- Study Period 1: screening period, lasting no more than 12 weeks.
- Study Period 2: treatment period, with a 20-week escalation followed by at least a 32-week maintenance period

The study will continue until approximately 52 weeks after the last participant is randomized. The maximum duration of an individual's participation is estimated to be 120 weeks and will depend on duration of study enrollment.

Participant Visit Scheme

Study participants will undergo screening assessments and procedures, randomization, and double-blinded treatment with tirzepatide or placebo. Assessments and procedures to be conducted in each treatment period are described in the SoA (Section 1.3) and in Study Assessments and Procedures (Section 8).

<u>Screening</u>

Screening procedures will be performed at Visit 1. Visit 1 procedures may be conducted over more than 1 day as long as all activities are completed within the allowable visit tolerance for each visit. The duration of Visit 1 is anticipated to be <6 weeks but may be longer. Visit 1 should no more than 12 weeks from date of informed consent.

At Visit 1, two 6MWTs will be conducted. The investigator must ensure that the participant is recovered from completion of the first 6MWT prior to conducting the second 6MWT (at least 1 hour between each test). The screening 6MWTs may be conducted over more than 1 day.

<u>Randomization</u>

At Visit 2, prior to randomization, the participant needs to complete the 6MWT. Visit 2 may need to be conducted over 2 in-clinic visits (considered Visit 2a and Visit 2b) if a repeat 6MWT is necessary to assess participant eligibility. If the participant is required to return to repeat the 6MWT, the remaining procedures should be conducted at the second in-clinic visit for randomization (Visit 2b), more than 10 days from Visit 2a. See Section 10.10.1 for details on when a participant must return for a second Visit 2 6MWT.

Participants will be randomized and receive blinded study drug at the end of Visit 2 after all screening procedures are completed. The participant must not receive study drug until all eligibility criteria, including the 6MWT, are met.

Treatment

Starting from randomization, the participant receives study drug and procedures are conducted as described in the SoA (Section 1.3). Every effort should be made by the investigator to maintain participants on study drug.

Study drug dose will be escalated as illustrated in the study schema (Section 1.2). Dose escalation will continue until the participant reaches the maintenance dose of either 5 mg, 10 mg, or 15 mg. Participants who do not tolerate the first dose escalation (that is, from 2.5 mg to 5 mg [or placebo equivalent]) will need to discontinue from study drug.

Participants begin treatment with either tirzepatide or placebo starting at 2.5 mg given as a subcutaneous (SC) injection every week (QW). The dose level is increased by 2.5 mg increments approximately every 4 weeks for the first 20 weeks according to the participant's tolerability, reaching a MTD up to 15 mg. Once a MTD has been achieved, participants will continue at this MTD until study end, treatment discontinuation, or study discontinuation. Dose modifications will be permitted during the study under the circumstances specified in Section 6.6.

During the study treatment period, if an unscheduled visit or telephone visit is deemed necessary to support participant compliance, this is allowable at the discretion of the investigator site personnel. Optional telephone visits can be performed approximately 2 weeks after starting study drug and after each dose increase.

Participants will continue into the extended maintenance period with the same treatment assignment starting with Visit 13. Extended visits (EV) continue until criteria for study discontinuation is met or study ends (see Study Closeout and Final Visit below).

Study Closeout and Final Visit

The study will continue until approximately 52 weeks after the final participant is randomized. A study duration of approximately 30 months is planned.

Approximately 3 months prior to the anticipated end of study, a study closeout will be declared. During the study closeout, a final visit (Visit 99) will be planned for each participant, with the exception of those who have died or prematurely discontinued from the study (Section 7.2). Study procedures for the final visit will be performed as outlined in the SoA (Section 1.3).

All unused study drug (unused single-dose pens) must be returned for compliance and final drug accountability. The sharp items container should also be returned to site or disposed of per local regulations.

4.2. Scientific Rationale for Study Design

Study GPID is a Phase 3 study designed to examine the efficacy and safety of SC QW tirzepatide MTD compared with placebo in participants with HFpEF and BMI ≥30 kg/m^2.

A placebo comparator was selected for this trial in accordance with regulatory guidance (FDA 2007; EMA 2016). Inclusion of a placebo comparator in Study GPID will allow for a direct assessment of the safety and efficacy of tirzepatide in participants with HFpEF and obesity.

Additionally, there is currently no approved therapy to be used as an active comparator in this population.

An endpoint assessment at 52 weeks of treatment is considered appropriate to assess the improvement of symptom and functional capacity. An extended maintenance treatment period increases the opportunity to evaluate HF events and outcomes.

The parallel-group design for treatment comparison was chosen to avoid any interaction between treatments that may interfere with the interpretation of the trial outcome. To minimize potential confounding effect of changes to concomitant medications, participants will be permitted to use the stable dose of concomitant medications that they require during the study. Medications that may interfere with the assessment of efficacy and safety characteristics of the study drug will not be allowed (see Section 6.5).

The primary endpoints were selected to assess the clinical benefit that tirzepatide could provide to patients in a holistic manner.

Assessment of HF events is relevant to HFpEF, which is characterized by a high frequency of recurrent HF hospitalizations. Moreover, hospitalization events reflect disease progression and high subsequent risk and predisposition, both of readmission and death (Solomon et al. 2007).

The conventional reporting of HF outcomes uses CV death and hospitalization for HF as a composite endpoint. Limitations of this approach include handling of each endpoint with equal importance irrespective of clinical significance, and ignorance of symptomatic and functional endpoints that are also relevant to patients from a clinical standpoint. To overcome this problem, the Win ratio approach was selected using 4 clinically meaningful endpoints as a hierarchical composite (Pocock et al. 2012; Ferreira 2020). This approach has several advantages over the conventional outcome assessment: 1) it prioritizes the more significant component of the composite (for example, death); 2) more of major component events are included in the analysis; and 3) it allows holistic evaluation of drug effects in a complex disorder such as HF.

Obesity is associated with cardiometabolic derangement and depleted myocardial energetics. During exercise in those with obesity, the heart does not increase adenosine triphosphate, leading to cardiac function impairment and exercise intolerance. It has been shown that weight loss reverses the energetic changes (Rayner et al. 2020). In patients with HFpEF, due to increased filling pressures, functional capacity is severely impaired, and patients can develop symptoms with light exercise. As a result, the ability to perform activities of daily living is deteriorated. Obesity is one of the main attributes to worsen quality of life in patients with HFpEF (Reddy et al. 2020). Therefore, 6MWD and KCCQ are meaningful endpoints to evaluate the clinical benefit of tirzepatide in patients with HFpEF and obesity.

For the primary composite endpoint, participants will be classified based on 6MWD categories of change from baseline with 10% incremental for improvement or worsening and KCCQ-CSS categories of change from baseline with 5- or 10-point improvements or worsening. It was estimated that the minimal important difference in 6MWD is approximately 30 to 37 meters in individuals with chronic HF. Relative to the mean 6MWD in these study populations (approximately 350 meters and 480 meters), the 30 to 37 meters is approximately 8% to 9% (Shoemaker et al. 2013; Täger et al. 2014).

For KCCQ-CSS, the categories were specified based on the evidence in the literature suggesting that 5- and 10-point changes in KCCQ summary scores represent small and moderate-to-large clinical changes, respectively. Each 5-point improvement in KCCQ scores has been demonstrated to be associated with improvements in CV mortality and all-cause hospitalization rates, while each 5-point decrement in KCCQ scores was associated with increased risk of CV death and the combined endpoint of CV death and hospitalization, in a linear fashion (Spertus et al. 2020).

4.2.1. Patient Input into Design

The sponsor involved patients in the design of this study by engaging patients in virtual collaborative events. The insights gained from these events were used to ensure that the study design is supportive of the well-being of the study participants and that the study procedures can be implemented effectively at the investigative sites.

4.3. Justification for Dose

Tirzepatide doses of up to 15 mg administered SC QW will be evaluated in this study.

Participants may be treated with lower maintenance doses of 5 mg or 10 mg if they do not achieve full dose escalation to 15 mg and/or do not tolerate 15 mg.

These doses and associated escalation schemes were selected based on assessment of safety, efficacy (glycemic and weight loss benefit), and GI tolerability data followed by exposure response modeling of data in participants with T2DM in Phase 1 and Phase 2 studies. Dosing algorithms starting at a low dose of 2.5 mg accompanied by dose escalation of 2.5 mg increments every 4 weeks should permit time for development of tolerance to GI events and are predicted to minimize GI tolerability concerns.

The dose selection of tirzepatide is based on the findings of the Phase 2 study results. Tirzepatide doses of 5 mg, 10 mg, and 15 mg QW have been tested and compared with dulaglutide 1.5 mg QW or placebo in a Phase 2 study (Frias et al. 2018). While all 3 doses of tirzepatide significantly improved the glycemic control versus dulaglutide, the largest difference was observed in the 15-mg tirzepatide treatment group. Moreover, the reduction in body weight on tirzepatide was also dose-dependent and greatest in the 15 mg QW treatment group.

4.4. End of Study Definition

The end of the study is defined as the date of the last visit or last scheduled procedure shown in the SoA for the last participant in the trial globally.

The criteria used to determine if a participant has completed the study will be described in the SAP.

5. Study Population

Prospective approval of protocol deviations to recruitment and enrollment criteria, also known as protocol waivers or exemptions, is not permitted.

5.1. Inclusion Criteria

Participants are eligible to be included in the study only if all of the following criteria apply:

Age

1. Participant must be at least 40 years of age inclusive, at the time of signing the ICF.

Type of Participant and Disease Characteristics

2. 6MWD ≤465 meters at both Visit 1 tests, between ≥100 meters and ≤425 meters at Visit 2 and change from the preceding qualifying 6MWD is <20% and <40 meters. See Section 10.10.1 for the flow diagram of the below qualifiers.
   - *If* Visit 2a 6MWD is both between 100 and 425 meters and…
     - <20% AND <40 meter change from the higher of the two 6MWDs conducted at Visit 1, *then* participant meets this inclusion criterion.
     - ≥20% OR ≥40 meter change from the higher of the two 6MWDs at Visit 1, *then* participant must attend Visit 2b. If at Visit 2b, the 6MWD is between 100 and 425 meters and <20% AND <40 meter change from preceding (Visit 2a) 6MWD, then participant meets this inclusion criterion.
3. Chronic HF (NYHA Class II-IV) diagnosed for at least 3 months before Visit 1
4. LVEF ≥50% demonstrated by echocardiogram performed at Visit 1 or within 6 months of Visit 1
5. At least 1 of the following to document evidence of HF:
   - Elevated NT-proBNP >200 pg/mL for participants without atrial AF or >600 pg/mL for participants with AF, as analyzed at the central laboratory at Visit 1
     OR
   - Evidence of structural heart disease:
     - LA enlargement (any of the following: LAV index ≥29 mL/m^2, or LAV >58 mL in male participants and >52 mL in female participants, or LA area >20 cm^2, or LA diameter >40 mm in male and >38 mm in female participants) determined by echocardiogram at Visit 1 or within 6 months of Visit 1

     OR
   - Evidence of elevated filling pressure:
     - At rest (PCWP ≥15 mmHg or LVEDP ≥15 mmHg) or with exercise (PCWP ≥25 mmHg) (based on historical record, not associated with hospitalization for decompensation of HF, within 2 years of Visit 1), or
     - E/e' ratio >15 (septal) or >13 (average of septal and lateral) determined by echocardiogram at Visit 1 or within 6 months of Visit 1

Note: Supporting medical documentation is required in all instances

6. Either one of:
   - eGFR <70 mL/min/1.73 m^2 at Visit 1, OR
   - HF decompensation within 12 months of Visit 1, defined as hospitalization for HF requiring IV diuretic treatment or urgent HF visit requiring IV diuretic treatment
     Note: Supporting medical documentation is required in all instances
7. Stable dose of all concomitant HF medications (these may include beta blockers, ACEis, ARBs, MRAs, ARNI, and/or SGLT2is), except for oral diuretics, for at least 4 weeks prior to Visit 1 and throughout the screening period
8. If treated with oral diuretics, dose must be stable for at least 2 weeks prior to Visit 1 and throughout the screening period; volume control must be optimally achieved in the opinion of the investigator
9. KCCQ-CSS ≤80 at Visit 1

Weight

10. BMI ≥30.0 kg/m^2 at Visit 1

Sex

11. At the time of signing the ICF:
    a. **Male participants**: Male participants with partners of childbearing potential should be willing to use reliable contraceptive methods for the duration of the trial and for 4 months thereafter (see Appendix 4 [Section 10.4]).
    b. Female participants:
    - Female participants not of childbearing potential may participate and include those who are infertile due to surgical sterilization and/or postmenopausal. Please refer to Appendix 4 (Section 10.4) for definitions.
    - Female participants of childbearing potential (not surgically sterilized and between menarche and 1-year postmenopausal) must:
      - test negative for pregnancy at Visit 1 based on a serum pregnancy test followed by a negative urine pregnancy test within 24 hours prior to exposure and agree to use 2 forms of effective contraception, if sexually active, where at least 1 form is highly effective, for the duration of the trial and for 2 months after the last injection, and
      - not be breastfeeding.

Contraceptive use by men or women of childbearing potential should be consistent with local regulations regarding the methods of contraception for those participating in clinical trials. See Appendix 4 (Section 10.4) for guidance.

Informed Consent

12. Capable of giving signed informed consent as described in Appendix 1 (Section 10.1), which includes compliance with the requirements and restrictions listed in the ICF and in this protocol.

5.2. Exclusion Criteria

Participants are excluded from the study if any of the following criteria apply:

Medical Conditions

13. Myocardial infarction, coronary artery bypass graft surgery, or other major CV surgery/intervention, stroke or transient ischemic attack in past 90 days, or unstable angina pectoris in past 30 days, prior to Visit 1 or during screening; Have NYHA Class I heart failure at either Visit 1 or Visit 2

14. Dominant contribution of noncardiac causes to exercise impairment or symptoms
    - Lung disease: pulmonary arterial hypertension, chronic thromboembolic pulmonary hypertension (CTEPH), or severe pulmonary disease including (COPD)
    - Other medical conditions: severe anemia (hemoglobin level <9 g/dL) at Visit 1, untreated thyroid disease or TSH >4.78 mU/L at Visit 1, or significant musculoskeletal disease
    - Orthopedic conditions that limit the ability to walk, such as severe arthritis in the leg, knee, hip injuries, hemiplegia, or amputation with artificial limb without stable prosthesis function for the past 3 months
    - Any condition that in the opinion of the investigator would interfere with the assessment of 6MWT

15. LVEF <40% by local echocardiography, MRI or other modalities documented any time within 2 years of Visit 1
16. Acute decompensated HF (exacerbation of HF) requiring IV diuretics, IV inotropes, or IV vasodilators, or left ventricular assist device (LVAD) within 4 weeks prior to Visit 1, and/or during the screening period until randomization
17. Impaired renal function, defined as eGFR <15 mL/min/1.73 m^2 (CKD-EPI) or requiring dialysis at Visit 1
18. Any one of the following:
    - Systolic blood pressure (SBP) ≥180 mmHg at Visit 1
    - SBP >160 mmHg both at Visit 1 and at Visit 2
    - Have symptomatic hypotension or SBP <100 mmHg at Visit 1 or Visit 2
19. Resting heart rate (sinus rhythm) ≥100 bpm at either Visit 1 or Visit 2
20. Atrial fibrillation or atrial flutter with a resting heart rate >110 bpm documented by ECG at either Visit 1 or Visit 2
21. Cardiac amyloidosis or cardiomyopathy based on accumulation disease (for example, haemochromatosis, Fabry disease), muscular dystrophy, cardiomyopathy with reversible causes (for example, stress cardiomyopathy), hypertrophic cardiomyopathy, Chagas cardiomyopathy or known pericardial constriction, or any severe (obstructive or regurgitant) valvular heart disease likely to lead to surgery during the study period
22. Completed prior surgical treatment for obesity or had liposuction or abdominoplasty within 1 year prior to Visit 1. Participants who plan to have surgical treatment for obesity or liposuction or abdominoplasty during the duration of the study are excluded.

23. Participation in a structured exercise training program in the 1 month prior to Visit 1 or planning to start a program during the study
24. Have T1DM
25. For participants with T2DM:
- Have uncontrolled diabetes requiring immediate therapy (such as diabetic ketoacidosis) at Visit 1 or Visit 2, in the judgement of the physician
- Have had 1 or more events of severe hypoglycemia and/or 1 or more events of hypoglycemia unawareness within 6 months prior to Visit 1 (see Section 10.5.1.1 for definition of hypoglycemia)
- Have HbA1c ≥9.5% (80 mmol/mol) at Visit 1, as analyzed at the central laboratory
- Have a history of proliferative diabetic retinopathy or diabetic maculopathy. Patients with severe nonproliferative diabetic retinopathy that requires acute treatment are also excluded.
- Treated with premix or prandial insulins or intensified insulin regimens (multiple daily injection with basal and prandial insulins or insulin pump therapy) at Visit 1

26. History of acute or chronic pancreatitis or at high risk for acute pancreatitis (for example, serum triglyceride level >500 mg/dL [5.65 mmol/L])
27. Have acute or chronic hepatitis, signs and symptoms of any other liver disease other than nonalcoholic fatty liver disease, or any of the following, as determined by the central laboratory during Visit 1:
- ALT or AST levels >2.5X the ULN for the reference range.

Note: Participants with nonalcoholic fatty liver disease are eligible to participate in this trial if their ALT level is ≤3.0X the ULN for the reference range.

28. Have a calcitonin level at Visit 1 of:
    - ≥20 ng/L, if eGFR is ≥60 mL/min/1.73 m^2
    - ≥35 ng/L, if eGFR is <60 mL/min/1.73 m^2
29. Have a family or personal history of medullary thyroid carcinoma (MTC) or Multiple Endocrine Neoplasia (MEN) Syndrome type 2
30. Have a history of an active or untreated malignancy or are in remission from a clinically significant malignancy (other than basal- or squamous-cell skin cancer, in situ carcinoma of the cervix, or in situ prostate cancer) for less than 5 years
31. Have a history of any other condition (such as known drug or alcohol abuse, diagnosed eating disorder, or other psychiatric disorder) that, in the opinion of the investigator, may preclude the participant from following and completing the protocol
32. Have a known clinically significant gastric emptying abnormality (for example, severe diabetic gastroparesis or gastric outlet obstruction) or chronically take drugs that directly affect GI motility

Prior/Concomitant Therapy

33. Treatment with any incretin, GLP-1 RA, or pramlintide in the 3 months prior to Visit 1
34. Discontinuation of any incretin, GLP-1 RA, or pramlintide due to intolerability any time prior to Visit 1

35. Have any other condition not listed in this section (for example, hypersensitivity or intolerance) that is a contraindication to GLP-1 RA
36. Implantable cardioverter defibrillator (ICD) implantation within 1 month prior to Visit 1 or planned implantation during the course of the study
37. Currently implanted left ventricular assist device (LVAD)
38. Cardiac resynchronization therapy (CRT) implanted within 6 months prior to Visit 1 or planned implantation during the course of the trial
39. Current use of medication associated with weight gain or weight loss, except when on stable dose for at least 3 months prior to Visit 1, and expected to be stable during the study period

Prior/Concurrent Clinical Study Experience

40. Have participated within the last 6 months in a clinical study involving an investigational product

Other Exclusions

41. Investigator site personnel directly affiliated with this study and/or their immediate families. Immediate family is defined as a spouse, parent, child, or sibling, whether biological or legally adopted
42. Lilly employees

5.3. Lifestyle Considerations

Study participants should be instructed not to donate blood or blood products during the study and for 8 weeks following the study.

5.4. Screen Failures

Screen failures are defined as participants who consent to participate in the clinical study but are not subsequently randomly assigned to study drug. A minimal set of screen failure information is required to ensure transparent reporting of screen failure participants to meet the CONSORT publishing requirements and to respond to queries from regulatory authorities. Minimal information includes demography, screen failure details, eligibility criteria, and any SAE.

Individuals who do not meet certain criteria for participation in this study (screen failure) may be rescreened once. Additionally, individuals who do not complete screening within 12 weeks from ICF date should be screen failed and may be rescreened. Rescreened participants should sign a new ICF and be assigned a new participant number. The interval between rescreenings should be at least 2 weeks. For participants who may have screen failed due to HbA1c criterion not met, the time to permit rescreening is at least 8 weeks. If, in the opinion of the investigator, an ineligible laboratory test result is the result of an error or extenuating circumstance, then that parameter can be retested once without the participant having to be rescreened. For rescreened participants, a repeat echocardiogram is not permitted.

Participants may be rescreened for the following reasons:

- Have become eligible to enroll in the study as the result of a protocol amendment

- Status has changed such that the eligibility criterion that caused the participant to screen fail would not cause the participant to screen fail again
- Completed screening and met all inclusion and exclusion requirements but are unable to be enrolled due to extenuating circumstances (such as severe weather, death in family, or child illness)

6. Study Intervention

Study drug is defined as any investigational intervention(s), marketed product(s), placebo, or medical device(s) intended to be administered to/used by a study participant according to the study protocol. For this study, 'study intervention' and 'study drug' are equivalent.

6.1. Study Interventions Administered

**Intervention Name**	Placebo	Tirzepatide (LY3298176)
**Type**	Drug (placebo)	Drug
**Dose Formulation**	Single-dose pen	Single-dose pen
**Unit Dose Strengths**	Not applicable	2.5 mg, 5 mg, 7.5 mg, 10 mg, 12.5 mg, 15 mg
**Dosage Levels**	Not applicable	15 mg QW<br>(or maximum tolerated dose of 5 mg QW or 10 mg QW)
**Route of Administration**	Subcutaneous	Subcutaneous
**Use**	Placebo	Experimental
**IMP and NIMP**	IMP	IMP
**Sourcing**	Provided centrally by the sponsor and dispensed via IWRS	
**Packaging and Labeling**	Study drug will be provided in autoinjectors (single-dose pens), packaged in cartons to be dispensed.<br>Clinical study materials will be labeled according to country regulatory requirements.	

Abbreviations: QW = weekly; IMP = investigational medicinal product; IWRS = interactive web-response system; NIMP = non-investigational medicinal product.

The following table shows the randomized study drugs for the entire study.

Treatment Group	Treatment Period Interval					
	Weeks 0 to 3	Weeks 4 to 7	Weeks 8 to 11	Weeks 12 to 15	Weeks 16 to 19	Weeks 20 to End of Treatment Period
Tirzepatide	2.5 mg	5 mg	7.5 mg	10 mg	12.5 mg	15 mg or MTD
Placebo	→					

Abbreviation: MTD = maximum tolerated dose.

There are no restrictions on the time of day each weekly dose of study drug is given, but it is advisable to administer the SC injections on the same day and same time each week. The actual date, time, and injection site location of all dose administrations will be recorded in the diary by the participant. If a dose of study drug is missed, the participant should take it as soon as possible, unless it is within 72 hours of the next dose, in which case that dose should be skipped, and the next dose should be taken at the appropriate time. The day of weekly administration can be changed if necessary, as long as the last dose was administered 72 or more hours previously.

All participants will inject study drug subcutaneously in the abdomen or thigh using the injection supplies provided; a caregiver may also administer the injection in the participant's upper arm. The injection site location of all dose administrations will be recorded by the participant. A new autoinjector will be used for each injection. If study drug is to always be injected in the same body region, participants should be advised to rotate injection sites each week.

6.1.1. Medical Devices

The combination product provided for use in the study is a tirzepatide or matching placebo autoinjector.

6.2. Preparation/Handling/Storage/Accountability

- The investigator or designee must confirm appropriate storage conditions have been maintained during transit for all study drug received and any discrepancies are reported and resolved before use of the study drug.
- Only participants enrolled in the study may receive study drug. Only study personnel may supply study drug.
- All study drug must be stored in a secure, environmentally controlled, and monitored (manual or automated) area in accordance with the labeled storage conditions with access limited to the investigator and authorized study personnel.
- The investigator, institution, or the head of the medical institution (where applicable) is responsible for study drug (includes study drug and autoinjector or single-dose pen) accountability, reconciliation, and record maintenance (that is, receipt, reconciliation, and final disposition of records).
- Study site staff must regularly assess whether the participant is correctly administering the assigned study drug and storing study drug according to the provided instructions.

Further guidance and information for the final disposition of unused study drugs are provided in the study training materials.

The investigator or designee is responsible for the following:

- Explaining the correct use of the study drug to the participant
- Verifying that instructions are followed properly
- Maintaining accurate records of study drug dispensing and collection as well as records of interruptions in study drug administration
- Instructing the participant to discard all used autoinjectors for study drug in a closeable, puncture-resistant container and dispose according to local regulations, and
- Considering dose adjustment of antihyperglycemic medications (see Section 6.5) at Visit 2 from first administration of study drug.

6.3. Measures to Minimize Bias: Randomization and Blinding

This is a double-blind study.

Participants who meet all criteria for enrollment will be randomized to one of the study treatment groups at the end of Visit 2. Assignment to treatment groups will be determined by a computer-generated, random sequence using an IWRS. Participants will be randomized in a 1:1 ratio to receive tirzepatide or placebo. The randomization will be stratified by HF decompensation (hospitalization for HF requiring IV diuretic treatment or urgent HF visit requiring IV diuretic treatment) within 12 months of screening (Y/N), diagnosed T2DM (Y/N), and BMI ≥35 kg/m^2 (Y/N).

Before the study is initiated, the log in information and directions for the IWRS will be provided to each site.

Study drug will be dispensed at the study visits shown in the SoA.

Returned unused study drug should not be re-dispensed to the participants.

The IWRS will be programmed with blind-breaking instructions. In case of an emergency, the investigator has the sole responsibility for determining if unblinding of a participant's drug assignment is warranted. Participant safety must always be the first consideration in making such a determination. If a participant's drug assignment is unblinded, the sponsor must be notified immediately after breaking the blind. The date and reason that the blind was broken must be recorded in the source documentation and CRF, as applicable.

If an investigator, site personnel performing assessments, or participant is unblinded, the participant must be discontinued from the study. In cases where there are ethical reasons to have the participant remain in the study, the investigator must obtain specific approval from a sponsor CRP for the participant to continue in the study.

6.4. Study Intervention Compliance

Participant compliance with study drug and adherence to study visits and procedures will be vitally important to meet the study objectives. This will be emphasized through comprehensive site training and consistently monitoring participant retention throughout the duration of the study.

Study drug compliance will be determined by the following:

- Study drug administration data will be recorded by the participants in the participant study diary and reviewed by the investigator at each study visit.
- The participants will be instructed to return any unused study drug and/or empty cartons at the next visit to the study site for the purpose of performing drug accountability.

Treatment compliance for each visit interval is defined as taking at least 75% of the required doses of study drug. Similarly, a participant will be considered significantly noncompliant if he or she is judged by the investigator to have intentionally or repeatedly taken more than the prescribed amount of medication (more than 125%).

In addition to the assessment of a participant's compliance with the study drug administration, other aspects of compliance with the study drug will be assessed at each visit based on the participant's adherence to the visit schedule, completion of study diaries, and any other parameters the investigator considers necessary.

Participants considered to be poorly compliant with their medication and/or the study procedures will receive additional training and instruction, as required, and will be reminded of the importance of complying with the protocol.

6.5. Concomitant Therapy

Any medication or vaccine (including over-the-counter or prescription medicines, vitamins, and/or herbal supplements) or other specific categories of interest that the participant is receiving at the time of enrollment or receives during the study must be recorded along with:

- Reason for use
- Dates of administration including start and end dates
- Dosage information including dose and frequency for concomitant therapy of special interest

The sponsor should be contacted if there are any questions regarding concomitant or prior therapy.

Initial doses of tirzepatide delay gastric emptying and have the potential to transiently impact the rate of absorption of concomitantly administered oral medicinal products. Tirzepatide should be used with caution in participants receiving oral medicinal products that require rapid GI absorption following the initial doses of tirzepatide, as exposure to oral medications may be increased.

Prevention of Hypoglycemia

Similar to GLP-1 RAs, tirzepatide does not generally cause hypoglycemia, but it is recommended to decrease the dose of concomitant sulfonylurea or insulin to reduce the risk of hypoglycemic episodes in patients with T2DM. For participants with T2DM, specific, individually tailored adjustments of the respective antihyperglycemic medications should be considered during the entire study. At Visit 2, with the initiation of study drug, the dose adjustments to the following concomitant glucose lowering medications are recommended.

Sulfonylureas: Sulfonylurea dose is recommended to be reduced at least 50% or discontinued, especially if the participant is receiving a low dose at randomization.

Insulins: For participants on basal insulin and with screening HbA1c ≤8.5%, the daily dose is recommended to be reduced by at least 20%.

During the dose escalation period, consider adjusting the total daily dose of insulin, if required for controlling worsening hyperglycemia and its acute complications.

During the maintenance period, further insulin dose reduction for the prevention of hypoglycemia is to be considered at the investigator's discretion.

Standard of Care for T2DM

The standard of care for diabetes may be adjusted at the discretion of the investigator as clinically indicated in accordance with local standard of care and professional society guidelines.

Participants will be permitted to use concomitant medications that they require during the study, except certain medications that may interfere with the assessment of efficacy and safety characteristics of the study drug. Prohibited medications include all GLP-1 RAs and pramlintide. Discontinuation of dipeptidyl peptidase 4 inhibitors at randomization is recommended in line with guidelines. Similarly, the use of dipeptidyl peptidase 4 inhibitor therapies during the study is also discouraged (Davies et al. 2018).

Regarding the use of insulin, participants can be included if treated with basal insulin only; and are allowed to use prandial insulin if needed during the study to attain optimal glucose control.

Hyperglycemia Rescue

Other medications for glycemic control for participants with T2DM meeting severe, persistent hyperglycemia criteria for rescue may be added during the study at the investigator's discretion.

Rescue therapy with glucose-lowering agents, including basal and prandial insulins, may be medically indicated in situations after randomization due to severe, persistent hyperglycemia or early discontinuation of study drug.

Hyperglycemia rescue criteria will be determined from values recorded in T2DM participant diaries. If a diary value equal to or greater than the glycemic threshold for rescue (see definitions below) is recorded, that value should be confirmed by a repeat fasting glucose text within 48 hours (for example, local laboratory). Intensification of T2DM therapy should be initiated if confirmed fasting glucose values are:

- ≥15.0 mmol/L (270 mg/dL) from baseline to Week 6 over at least a 2-week period (at least 2 consecutive values) after randomization
- ≥13.3 mmol/L (240 mg/dL) from Week 6 to Week 12 over at least a 2-week period (at least 2 consecutive values)
- ≥11.1 mmol/L (200 mg/dL) from Week 12 to end of trial over at least a 2-week period (at least 2 consecutive values)

In addition, if HbA1c is >9.0% at Week 12 or >8.0% at Week 24 or later in the study, glucose-lowering therapy should be adjusted to improve glycemic control as outlined above. In the event a participant's HbA1c values are less than these thresholds but are higher than what the

investigator feels comfortable leaving untreated, glucose-lowering medication can be adjusted. In addition, if participants develop symptoms of hyperglycemia (for example, polyuria and polydipsia), the investigator should implement measures to determine glycemic control and adjust as necessary. For participants newly diagnosed with T2DM during the trial, appropriate glucose-lowering therapy should be initiated per standard of care.

Standard of Care for Heart Failure

Anticipated treatment for heart failure should be decided prior to randomization.

Both American College of Cardiology/American Heart Association and European Society of Cardiology guidelines recommend symptom management with diuretic agents in patients with excess volume, as well as aggressive risk factor management for comorbidities for the treatment of HFpEF (van der Meer JACC 2019). Optimization of volume status and proactive adjustment of diuretic doses will help control symptoms and volume overload.

Participants should remain on stable doses of medications to treat heart failure condition and comorbidities such as hypertension. With the exception of diuretics, dose modification or alteration of such background therapies should be avoided unless all other measures fail to improve the participant's condition. However, if the participant's condition warrants a change in any of these medications, it will be allowed at the discretion of the investigator.

Management of Participants with Gastrointestinal Symptoms

In the Phase 2 program, the most commonly reported TEAEs for participants receiving tirzepatide were nausea, vomiting, and diarrhea. To mitigate GI symptoms and manage participants with intolerable GI AEs, the investigator should:

- Advise participants to eat smaller meals, for example, splitting 3 daily meals into 4, or more smaller meals, and to stop eating when they feel full. Also, participants may be informed that lower-fat meals could be better tolerated.
- Prescribe symptomatic medication (for example, anti-emetic or antidiarrheal medication) per local country availability and individual participant needs. Use of symptomatic medication should be captured as concomitant medication in the eCRF.
- Temporarily interrupt study drug. See Section 6.6.1. The data related to temporary interruption of study drug should be documented in source documents and entered on the eCRF.
- After the interruption, follow the guidance for restarting study drug (Section 6.6.2).

If intolerable GI symptoms or events persist despite the above measures, other dose modifications (see Section 6.6) may be considered.

6.6. Dose Modification

Interventions to optimize study drug tolerance and adherence may be employed throughout the study and include, but are not limited to, brief temporary interruptions and use of additional medications to manage symptoms.

Dose modifications, including temporary interruption and de-escalation may occur to manage issues with tolerability. It is preferred to attempt a temporary dose interruption (at any time) (Section 6.6.1) to manage tolerability issues. After a temporary dose interruption, participants may resume study drug at the same dose, re-escalate to the prior dose level (if re-escalation is desired or required), or resume at a lower MTD dose level, as tolerated. Guidance for resuming study drug after a temporary dose interruption should be followed (Section 6.6.2).

<u>**Unwarranted excessive weight reduction**</u>: **Dose interruption is preferred over de-escalation for slowing unwarranted excessive weight loss; however, the method used is at the investigator's discretion.** After the dose escalation period and at the investigator's discretion, when excessive weight reduction is not warranted due to safety concerns, the investigator may choose to adjust the study drug dose without first attempting a temporary dose interruption. The participant's study drug dose will be permanently reduced to 5 mg, in a blinded fashion for the remainder of the study and the dose cannot be re-escalated. A dose adjustment for unwarranted excessive weight reduction is completed through the IWRS. Participants on 5 mg will have blinded study drug temporarily interrupted (Section 6.6.1).

Dose reductions for unwarranted excessive weight loss may occur at scheduled and unscheduled visits.

6.6.1. Temporary Interruption

Temporary study drug interruption should be utilized to manage tolerability issues. After randomization, the investigator may interrupt study drug, for example, due to an AE (such as nausea vomiting, or excessive unwarranted weight loss) or a clinically significant laboratory value. Guidance for resuming study drug after a temporary dose interruption should be followed (Section 6.6.2). If study drug interruption is due to an AE, the event is to be followed and documented.

The interruption of study drug is not equivalent to discontinuation from study treatment. Any interruption of study drug does not affect the study visit structure/schedule per the SoA (Section 1.3). Even if study drug is interrupted, study procedures should continue during the dose interruption. Every effort should be made by the investigator to maintain participants in the study and to restart study drug promptly after any interruption, as soon as it is safe to do so (see Section 6.6.2. for restarting study drug). Dose interruptions are managed through the IWRS. The data related to interruption of study drug will be documented in source documents and entered on the eCRF, however participant noncompliance should not be recorded as interruption of study drug on the eCRF.

Participants who have a temporary interruption of the study drug will continue participating in the trial according to the protocol to collect all planned efficacy and safety measurements (Section 1.3).

6.6.2. Restarting Study Drug after Interruption

In certain situations, the investigator may need to temporarily interrupt study drug. Every effort should be made by the investigator to maintain participants on study drug and to restart study drug after any temporary interruption, as soon as it is safe to do so. Distribution of study drug at the correct dose will be per IWRS instructions.

If study drug interruption is…	then…
1 or 2 consecutive doses	Participant restarts study drug at last administered dose, per escalation schedule. If the participant has reached maintenance dose level, the study drug dose level will restart at the same prior achieved maintenance dose level.
3 or more consecutive doses	Participant restarts study drug (at 5 mg, managed by IWRS) and repeats dose escalation scheme until maintenance dose is reached. If maintenance dose has previously been established, the dose escalation cannot not exceed the prior achieved maintenance dose level.
Due to an AE	The event is to be documented and followed according to the procedures in Section 8.3.
Due to intolerable persistent GI AE	Participants should be treated as suggested in Section 6.5.

Abbreviations: AE = adverse event; GI = gastrointestinal; IWRS = interactive web response service.

Investigators should inform the sponsor that study drug has been temporarily interrupted.

Participants are not required to re-escalate to the prior maintenance dose level if a tolerability issue recurs during dose re-escalation.

If this attempted dose re-escalation to the prior maintenance dose level is not tolerated, the dose should be reduced to the next lower 5 mg incremental dose that was tolerated (for example, 5 mg or 10 mg). The participant will remain at that dose level for the duration of the study.

For participants receiving 5 mg maintenance dose, no dose de-escalation is permitted. Only dose interruption is permitted to manage tolerability issues (Section 6.6.2). This can be performed through IWRS.

In the event that a participant has a temporary interruption that requires extending the escalation beyond Visit 8, unscheduled visits are allowed in the IWRS to facilitate a 4-week dispensing schedule to complete the escalation. Unscheduled visits either in the clinic or by telephone may be conducted to provide support and guidance to participants as needed.

6.7. Intervention after the End of the Study

Tirzepatide will not be made available to participants after conclusion of the study. Due to the double-blind study drug assignment, it is not known if a participant received active study drug or placebo. Participants will not be unblinded until study end and the final analyses are complete.

7. Discontinuation of Study Intervention and Participant Discontinuation/Withdrawal

7.1. Discontinuation of Study Intervention

7.1.1. Permanent Discontinuation from Study Drug

Before permanently discontinuing study drug, attempts to maintain the participant should be documented (see Dose Modification Section 6.6 and Temporary Dose Interruption, Section 6.6.2). Contact the sponsor before study treatment discontinuation occurs to discuss potential options to maintain the participant on study drug until final study ends. It is the goal for participants to remain on study drug treatment until study ends. It may be necessary for a participant to permanently discontinue study drug. If study drug is definitively discontinued, the participant will remain in the study and attend all scheduled visits to be evaluated for safety and efficacy and any further evaluations as described in the SoA.

Possible reasons leading to permanent discontinuation of study drug:

- **Participant decision**
    - The participant or the participant's designee (for example, legal guardian) requests to discontinue study drug
- **Investigator Decision**
    - The investigator decides that the participant should be discontinued from study drug
- **Discontinuation due to a hepatic event or liver test abnormality**
  Participants who are interrupted/discontinued from study drug due to a hepatic event or liver test abnormality should have additional hepatic safety data collected via CRF.

  Interruption/discontinuation of the study drug for abnormal liver test results should be considered by the investigator when a participant meets one of the following conditions after consultation with the sponsor-designated medical monitor. These criteria differ based on the baseline liver test findings and are summarized below (Regev et al. 2019). If study drug is interrupted, it can be restarted only if another etiology is identified and liver enzymes return to baseline.

  For participants with baseline ALT <1.5X ULN and normal baseline bilirubin:

    - ALT or AST >8X ULN
    - ALT or AST >5X ULN for more than 2 weeks
    - ALT or AST >3X ULN and TBL >2X ULN or INR >1.5
    - ALT or AST >5X ULN with the appearance of fatigue, nausea, vomiting, right upper-quadrant pain or tenderness, fever, rash, and/or eosinophilia (>5%)
    - ALP >3X ULN
    - ALP >2.5X ULN and TBL >2X ULN

    - ALP >2.5X ULN with the appearance of fatigue, nausea, vomiting, right quadrant pain or tenderness, fever, rash, and/or eosinophilia (>5%)

  For participants with baseline ALT ≥1.5X ULN and normal baseline bilirubin:

  - ALT >5X baseline or ≥500 U/L (whichever occurs first)
  - ALT >2X baseline or ≥300 U/L (whichever occurs first) and TBL >2X ULN
  - ALT >3X baseline or ≥300 U/L (whichever occurs first) with the appearance of fatigue, nausea, vomiting, right upper-quadrant pain or tenderness, fever, rash, and/or eosinophilia (>5%)
  - ALP >3X ULN
  - ALP >2.5X ULN and TBL >2X ULN
  - ALP >2.5X ULN with the appearance of fatigue, nausea, vomiting, right quadrant pain or tenderness, fever, rash, and/or eosinophilia (>5%)

- In addition, participants will be permanently discontinued from the study drug in the following circumstances:
  - acute or chronic pancreatitis (see Section 10.5.1.2)
  - if a participant is diagnosed with thyroid C-cell hyperplasia, MEN-2, or MTC, or pancreatic cancer after randomization
  - if any other TEAE, SAE, or clinically significant laboratory value for which the investigator believes that permanent study drug discontinuation is the appropriate measure to be taken
  - if a participant is diagnosed with T1DM
  - if a female participant becomes pregnant
  - if an investigator, site personnel performing assessments, or participant is unblinded, or
  - if the investigator, after consultation with the sponsor-designated medical resource, determines that a clinically significant systemic hypersensitivity reaction has occurred. A clinically significant systemic hypersensitivity reaction is one that occurs after administration of the study drug (for example, drug-related symptomatic bronchospasm, allergy-related edema/angioedema, or hypotension) and requires parenteral medication, does not respond to symptomatic medication, results in clinical sequelae, or is an anaphylactic reaction.
  - in the opinion of the investigator, the participant should permanently discontinue the study intervention for safety reasons

7.2. Participant Discontinuation/Withdrawal from the Study

In order to minimize the amount of missing data and to enable assessment of study objectives as planned in the study protocol, every attempt will be made to keep participants in the study, irrespective of the following:

- compliance to study drug
- adherence to visit schedule

- missing assessments
- study drug discontinuation due to AE (Section 7)
- development of comorbidities, and
- development of clinical outcomes.

The circumstances listed above are ***not*** valid reasons for participant discontinuation from the study.

Participant will be discontinued from study (early termination, ET) in the following circumstances:

- enrollment in any other clinical study involving a study drug or enrollment in any other type of medical research judged not to be scientifically or medically compatible with this study
- participation in the study needs to be stopped for medical, safety, regulatory, or other reasons consistent with applicable laws, regulations, and GCP, and
- if the participant becomes pregnant.

A participant may withdraw from the study:

- at any time at his/her own request, or
- at the request of his/her designee (for example, legal guardian).
- at the discretion of the investigator for safety, behavioral, compliance, or administrative reasons
- if enrolled in any other clinical study involving an investigational product, or enrolled in any other type of medical research judged not to be scientifically or medically compatible with this study
- if the participant, for any reason, requires treatment with a therapeutic agent that is prohibited by the protocol and has been demonstrated to be effective for treatment of the study indication. In this case, discontinuation from the study occurs prior to introduction of the new agent.

Study discontinuation is expected to be rare and participants should be provided with options for alternative follow-up methods and/or end of study vital status/endpoint ascertainment. At the time of discontinuing from the study, an ET visit should be conducted, as shown in the SoA. If the participant refuses to have an ET visit in the clinic, efforts should be made to collect data via telephone. See the SoA for data to be collected at the time of study discontinuation. If the participant has not already discontinued the study intervention, the participant will be permanently discontinued from the study intervention at the time of the decision to discontinue the study.

If the participant withdraws consent for disclosure of future information, the sponsor may retain and continue to use any data collected before such a withdrawal of consent. If a participant withdraws from the study, he/she may request destruction of any samples taken and not tested, and the investigator must document this in the site study records.

7.2.1. Inadvertently Enrolled Participants

If the sponsor or investigator identifies a participant who did not meet enrollment criteria and was inadvertently enrolled, then the participant should remain in the study and be discontinued from study drug when continued treatment would not be medically appropriate If the investigator and the sponsor CRP agree it is medically appropriate to continue the study drug, the investigator must obtain documented approval from the sponsor CRP to allow the inadvertently enrolled participant to continue study drug. Safety follow-up should be performed as outlined in Section 8.2 (Safety Assessments) and Section 8.3 (Adverse Events and Serious Adverse Events) of the protocol.

7.3. Lost to Follow up

A participant will be considered lost to follow-up if he or she repeatedly fails to return for scheduled visits and is unable to be contacted by the study site (≥2 consecutive visits). Site personnel or designee are expected to make diligent attempts to contact participants who fail to return for a scheduled visit or were otherwise unable to be followed up by the site.

Site personnel, or an independent third party, will attempt to collect the vital status of the participant within legal and ethical boundaries for all participants randomized, including those who did not get study drug. Public sources may be searched for vital status information. If vital status is determined to be deceased, this will be documented and the participant will not be considered lost to follow-up. Sponsor personnel will not be involved in any attempts to collect vital status information.

Discontinuation of specific sites or of the study as a whole are handled as part of Appendix 1 (Section 10.1)

8. Study Assessments and Procedures

Study procedures and their timing are summarized in the SoA.

Immediate safety concerns should be discussed with the sponsor immediately upon occurrence or awareness to determine if the participant should continue or discontinue study drug.

Adherence to the study design requirements, including those specified in the SoA, is essential and required for study conduct.

All screening evaluations must be completed and reviewed to confirm that potential participants meet all eligibility criteria. The investigator will maintain a screening log to record details of all participants screened and to confirm eligibility or record reasons for screening failure, as applicable.

Repeat or unscheduled samples may be taken for safety reasons or for technical issues with the samples.

Other visit methods (i.e. remote, telephone) may be considered at the discretion of the investigator if a participant is unable to come to their scheduled on-site visit. Alternative options to visit procedures must be considered with prior consultation and written approval of the sponsor.

8.1. Efficacy Assessments

8.1.1. Primary Efficacy Assessment

The primary efficacy endpoints are

- the hierarchal composite of time to all-cause mortality, occurrence of HF events through the end of the treatment period, the change from baseline in 6MWD, and the change from baseline in KCCQ-CSS at 52 weeks, and
- change from baseline in 6MWD at 52 weeks.

The primary efficacy assessments include

- time to all-cause mortality
- occurrence of HF events
- 6MWT, and
- KCCQ.

An independent CEC will adjudicate death and HF events. The CEC charter will contain the final detailed event definitions for all adjudicated events.

8.1.1.1. Time to All-Cause Mortality

All-cause mortality will be recorded by the investigator at the time of death.

8.1.1.2. Occurrence of Heart Failure Events

HF events will be recorded when the investigator becomes aware of any

- hospitalization for HF, or
- urgent HF visit.

8.1.1.3. Six-Minute Walk Test

Participants will perform an exercise capacity assessment using the 6MWT. Testing of the 6MWT should be performed as directed in the SoA (Section 1.3). The 6MWT is to be performed indoors on a straight, flat, hard surface that is at least 30 meters in length.

The 6MWTs at Visits 1 and 2 will be performed to assess participant eligibility. The Visit 1 tests will also serve as the training test to familiarize participants with the procedure. Additional details can be found in Section 10.10.1.

Prior to and at the end of each 6MWT, participants will be asked to rate their breathing discomfort and overall fatigue using the Borg Scale, separately, at each timepoint.

8.1.1.4. Kansas City Cardiomyopathy Questionnaire

The KCCQ is a 23-item, participant self-administered questionnaire that assesses impacts of HF "over the past 2 weeks" on the following 7 domains (Green et al. 2000; Joseph et al. 2013):

- Physical Limitation (6 items)
- Symptom Stability (1 item)
- Symptom Frequency (4 items)
- Symptom Burden (3 items)
- Self-Efficacy (2 items)
- Quality of Life (3 items), and
- Social Limitation (4 items).

Each of the 23 individual items are answered on Likert scales of varying lengths (5-point, 6-point, or 7-point scales). Domain scores are obtained by averaging the associated individual items and transforming the score to a 0 to 100 range. Higher scores indicate better health status. Summary scores are obtained by combining select domain scores:

- Total Symptom Score: mean of the Symptom Frequency and Symptom Burden scores
- Clinical Summary Score: mean of the Physical Limitation and Total Symptom scores, and
- Overall Summary Score: mean of the Physical Limitation, Total Symptom, Quality of Life, and Social Limitation scores.

The Clinical Summary Score will be used for the primary and key secondary endpoints.

8.1.2. Additional Secondary Efficacy Assessments

Body weight will be assessed as described in Section 8.2.1.

The NYHA classification will be assessed and recorded at the time points indicated in the SoA (Section 1.3) by an independent, blinded assessor. The NYHA classification is provided in Appendix 11 (Section 10.11).

8.1.3. Exploratory Efficacy Assessments

8.1.3.1. Patient-Reported Outcomes Assessments

The self-reported questionnaires will be translated into the native language of the region, linguistically validated, and administered according to the SoA (Section 1.3). At these visits, the questionnaires should be completed before the participant has discussed their medical condition or progress in the study with the investigator and/or site staff.

8.1.3.1.1. Patient Global Impression of Status – Overall

Study participants will be asked to complete a Patient Global Impression of Status – Overall item specifically developed for this study. This is a participant-rated assessment of their overall health "in the past 2 weeks" and is rated on a 5-point scale ranging from "1-Excellent" to "5-Poor."

8.1.3.1.2. Patient Global Impression of Status – Physical Function

Study participants will be asked to complete a Patient Global Impression of Status – Physical Function item specifically developed for this study. This is a participant-rated assessment of the overall impact of HF symptoms on their ability to perform physical activities "in the past 2 weeks" and is rated on a 5-point scale ranging from "1- Not impacted" to "5- Extremely impacted, cannot perform physical activities."

8.1.3.1.3. Patient Global Impression of Status – Symptom Severity

Study participants will be asked to complete a Patient Global Impression of Status – Symptom Severity item specifically developed for this study. This is a participant-rated assessment of the overall severity of their HF symptoms "in the past 2 weeks" and is rated on a 5-point scale ranging from "1- No symptoms" to "5- Very severe."

8.1.3.1.4. EQ-5D-5L

Generic health-related quality of life will be assessed using the EQ-5D-5L (EuroQoL Research Foundation 2019). The EQ-5D-5L is a standardized 5-item instrument for use as a measure of health outcome. It provides a simple descriptive profile and a single index value for health status that can be used in the clinical and economic evaluation of health care as well as population health surveys. The EQ-5D-5L comprises 5 dimensions of health (mobility, self-care, usual activities, pain/discomfort, and anxiety/depression). The 5L version, introduced in 2005, scores each dimension at 5 levels (no problems, slight problems, moderate problems, severe problems, and unable to perform/extreme problems), for a total of 3125 possible health states. In addition to the health profile, a single health state index value can be derived based on a formula that attaches weights to each of the levels in each dimension. This index value ranges between less than 0 (where 0 is a health state equivalent to death; negative values are valued as worse than dead) to 1 (perfect health). In addition, the EQ Visual Analog Scale records the respondent's self-rated health status on a vertical graduated (0 to 100) visual analog scale. In conjunction with the health state data, it provides a composite picture of the respondent's health status.

The EQ-5D-5L is used worldwide and is available in more than 170 languages. Details on the instrument, scoring, organizing, and presenting the data collected can be found in the EQ-5D-5L User Guide (EuroQoL Research Foundation 2019).

8.2. Safety Assessments

Planned time points for all safety assessments are provided in the SoA (Section 1.3).

8.2.1. Physical Examinations

- A complete physical examination will include, at a minimum, assessments of the CV, respiratory, GI and neurological systems, as well as a thyroid examination.
    - Body weight, waist circumference, and height should be measured. All weights for a given participant should be measured in a consistent manner using a calibrated scale (mechanical or digital scales are acceptable), using the same scale whenever possible, and after the participant has emptied their bladder. Participants should be lightly clothed but not wearing shoes while their weight is measured.
- Symptom-directed physical examinations will be conducted as described in the SoA.
    - Investigators should pay special attention to clinical signs and symptoms related to HF as well as related to previous serious illnesses. Particular interest would include dyspnea, orthopnea, paroxysmal nocturnal dyspnea, edema, jugular venous distension, and rales.

The physical examination should be performed before the first 6MWT, if more than one 6MWTs are done.

8.2.2. Vital Signs

For each participant, vital signs measurements should be conducted according to the SoA (Section 1.3). An apical heart rate should be assessed during the collection of vital signs. The vital signs collection associated with the 6MWT should be separate and may be performed using automated equipment.

Any clinically significant findings from vital signs measurements that result in a diagnosis and that occur after the participant receives the first dose of study drug should be reported to the sponsor or its designee as an AE via the eCRF.

8.2.3. Electrocardiograms

Single 12-lead ECGs will be obtained locally as outlined in the SoA (see Section 1.3).

All ECGs should be recorded after the participant has been supine for 5 minutes in a quiet room.

The ECGs must be interpreted by a qualified physician (the investigator or designee) at the site as soon after the time of ECG collection as possible, and ideally while the participant is still present, for immediate participant management, if needed. The investigator (or qualified designee) is responsible for determining if any change in participant management is needed, and must document his/her review of the ECG printed at the time of evaluation. If a clinically relevant abnormality is observed on the participant's ECG, then the investigator should assess the participant for symptoms (such as palpitations, near syncope, syncope, or chest pain). The investigator must report the presence of AF on the eCRF.

The original ECG must be retained at the investigative site.

The investigator or qualified designee's interpretation will prevail for immediate participant management purposes.

8.2.4. Clinical Safety Laboratory Assessments

With the exception of laboratory test results that may unblind the study, the sponsor or its designee will provide the investigator with the results of laboratory tests analyzed by a central vendor, if a central vendor is used for the clinical trial.

See Appendix 2 (Section 10.2) for the list of clinical laboratory tests to be performed. The SoA describes the timing and frequency.

The investigator must review the laboratory results, document this review, and report any clinically relevant changes occurring during the study as an AE. The laboratory results must be retained with source documents unless a Source Document Agreement or comparable document cites an electronic location that accommodates the expected retention duration. Clinically significant abnormal laboratory findings are those that are not associated with the underlying disease, unless judged by the investigator to be more severe than expected for the participant's condition.

All laboratory tests with values considered clinically significantly abnormal during participation in the study should be repeated until the values return to normal or baseline levels or are no longer considered clinically significant by the investigator or medical monitor.

- If such values do not return to normal/baseline levels within a period of time judged reasonable by the investigator, the etiology should be identified and the sponsor notified.
- All protocol-required laboratory assessments, as defined in Appendix 2 (Section 10.2), must be conducted in accordance with the SoA, standard collection requirements, and laboratory manual.

If laboratory values from non-protocol specified laboratory assessments performed at an investigator-designated local laboratory require a change in participant management or are considered clinically significant by the investigator (for example, SAE or AE or dose modification), then report the information as an AE.

All urine pregnancy tests will be performed locally according to the SoA. Guidance for contraception and definitions are defined in Appendix 4 (Section 10.4).

8.2.5. Safety Monitoring

The sponsor will periodically review evolving aggregate safety data within the study by appropriate methods. The study team will review safety reports in a blinded fashion according to the schedule provided in the Trial-Level Safety Review Plan. The sponsor will also review SAEs within time frames mandated by company procedures. The Sponsor CRP will, as appropriate, consult with the functionally independent Global Patient Safety therapeutic area physician or clinical scientist.

8.2.5.1. Hepatic Safety Monitoring

Close Hepatic Monitoring

Laboratory tests (Appendix 8 [Section 10.8]), including ALT, AST, ALP, TBL, D Bil, GGT, and creatine kinase, should be repeated within 48 to 72 hours to confirm the abnormality and to determine if it is increasing or decreasing, if one or more of these conditions occur:

If a participant with baseline results of …	develops the following elevations:
ALT or AST <1.5X ULN	ALT or AST ≥3X ULN
ALP <1.5X ULN	ALP ≥2X ULN
TBL <1.5X ULN	TBL ≥2X ULN (except for participants with Gilbert's syndrome)
ALT or AST ≥1.5X ULN	ALT or AST ≥2X baseline
ALP ≥1.5X ULN	ALP ≥2X baseline
TBL ≥1.5x ULN	TBL ≥1.5X baseline (except for participants with Gilbert's syndrome)

Abbreviations: ALP = alkaline phosphatase; ALT = alanine aminotransferase; AST = aspartate aminotransferase; TBL = total bilirubin; ULN = upper limit of normal.

If the abnormality persists or worsens, clinical and laboratory monitoring and evaluation for possible causes of abnormal liver tests should be initiated by the investigator in consultation with the sponsor-designated medical monitor. At a minimum, this evaluation should include physical examination and a thorough medical history, including symptoms, recent illnesses (for example, HF, systemic infection, hypotension, or seizures), recent travel, history of concomitant medications (including over-the-counter), herbal and dietary supplements, history of alcohol use, and other substance abuse.

Initially, monitoring of symptoms and hepatic biochemical tests should be done at a frequency of 1 to 3 times weekly, based on the participant's clinical condition and hepatic biochemical tests. Subsequently, the frequency of monitoring may be lowered to once every 1 to 2 weeks, if the participant's clinical condition and laboratory results stabilize. Monitoring of ALT, AST, ALP, and TBL should continue until levels normalize or return to approximate baseline levels.

Comprehensive Hepatic Evaluation

A comprehensive evaluation should be performed to search for possible causes of liver injury if one or more of these conditions occur:

If a participant with baseline results of …	develops the following elevations:
ALT or AST <1.5X ULN	ALT or AST ≥3X ULN with hepatic signs/symptoms* OR<br>ALT or AST ≥5X ULN
ALP <1.5X ULN	ALP ≥3X ULN

TBL <1.5X ULN	TBL ≥2X ULN (except for participants with Gilbert's syndrome)
ALT or AST ≥1.5X ULN	ALT or AST ≥2X baseline with hepatic signs/symptoms* OR<br>ALT or AST ≥3X baseline
ALP ≥1.5X ULN	ALP ≥2X baseline
TBL ≥1.5X ULN	TBL ≥2X baseline (except for participants with Gilbert's syndrome)

Abbreviations: ALP = alkaline phosphatase; ALT = alanine aminotransferase; AST = aspartate aminotransferase; TBL = total bilirubin; ULN = upper limit of normal.

* Hepatic signs/symptoms are severe fatigue, nausea, vomiting, right upper quadrant abdominal pain, jaundice, fever, rash, and/or eosinophilia >5%.

At a minimum, this evaluation should include physical examination and a thorough medical history, as outlined above, as well as tests for PT-INR; tests for viral hepatitis A, B, C, or E; tests for autoimmune hepatitis; and an abdominal imaging study (for example, ultrasound or CT scan).

Based on the participant's history and initial results, further testing should be considered in consultation with the sponsor-designated medical monitor, including tests for hepatitis D virus (HDV), cytomegalovirus (CMV), Epstein-Barr virus (EBV), acetaminophen levels, acetaminophen protein adducts, urine toxicology screen, Wilson's disease, blood alcohol levels, urinary ethyl glucuronide, and blood phosphatidylethanol (see Appendix 8 [Section 10.8]). Based on the circumstances and the investigator's assessment of the participant's clinical condition, the investigator should consider referring the participant for a hepatologist or gastroenterologist consultation, magnetic resonance cholangiopancreatography (MRCP), endoscopic retrograde cholangiopancreatography (ERCP), cardiac echocardiogram, or a liver biopsy.

Additional hepatic data collection (hepatic safety CRF) in study participants who have abnormal liver tests during the study

Additional hepatic safety data collection in hepatic safety CRFs should be performed in study participants who meet 1 or more of the following 5 conditions:

1. Elevation of serum ALT to ≥5X ULN on 2 or more consecutive blood tests (if baseline ALT <1.5X ULN)
    - o In participants with baseline ALT ≥1.5X ULN, the threshold is ALT ≥3X baseline on 2 or more consecutive tests
2. Elevated TBL to ≥2X ULN (if baseline TBL <1.5X ULN) (except for cases of known Gilbert's syndrome)
    - o In participants with baseline TBL ≥1.5X ULN, the threshold should be TBL ≥2X baseline
3. Elevation of serum ALP to ≥2X ULN on 2 or more consecutive blood tests (if baseline ALP <1.5X ULN)

  - In participants with baseline ALP ≥1.5X ULN, the threshold is ALP ≥2X baseline on 2 or more consecutive blood tests

4. Hepatic event considered to be an SAE
5. Discontinuation of study drug due to a hepatic event

Note: the interval between the two consecutive blood tests should be at least 2 days.

8.3. Adverse Events, Serious Adverse Events, and Product Complaints

The definitions of the following events can be found in Appendix 3 (Section 10.3):

- Adverse events (AEs)
- Serious adverse events (SAEs)
- Product complaints (PCs)

These events will be reported by the participant (or, when appropriate, by a caregiver, surrogate, or the participant's legally authorized representative).

The investigator and any qualified designees are responsible for detecting, documenting, and recording events that meet these definitions and remain responsible for following up events that are serious, considered related to the study drug or study procedures, or that caused the participant to discontinue the study drug or study (see Section 7).

Care will be taken not to introduce bias when detecting events. Open-ended and non-leading verbal questioning of the participant is the preferred method to inquire about event occurrences.

After the initial report, the investigator is required to proactively follow each participant at subsequent visits/contacts. All SAEs will be followed until resolution, stabilization, the event is otherwise explained, or the participant is lost to follow-up (as defined in Section 7.3).

For product complaints, the investigator is responsible for ensuring that follow-up includes any supplemental investigations as indicated to elucidate the nature and/or causality. Further information on follow-up procedures is provided in Appendix 3 (Section 10.3).

Suspected Unexpected Serious Adverse Reactions

Suspected unexpected serious adverse reactions (SUSARs) are serious events that are not listed in the IB and that the investigator identifies as related to study drug or procedure. United States 21 CFR 312.32, European Union Clinical Trial Directive 2001/20/EC, and the associated detailed guidance or national regulatory requirements in participating countries require the reporting of SUSARs. The sponsor has procedures that will be followed for the identification, recording, and expedited reporting of SUSARs that are consistent with global regulations and the associated detailed guidance.

8.3.1. Timing and Mechanism for Collecting Events

This table describes the timing, deadlines, and mechanism for collecting events.

Event	Collection Start	Collection Stop	Timing for Reporting to Sponsor or Designee	Mechanism for Reporting	Back-up Method of Reporting
**Adverse Event**					
AE	Signing of the informed consent form (ICF)	Participation in study has ended	As soon as possible upon site awareness	AE eCRF	N/A
**Serious Adverse Event**					
SAE and SAE updates – prior to start of study drug **and** deemed reasonably possibly related with study procedures	Signing of the informed consent form (ICF)	Start of study drug	Within 24 hours of awareness	SAE eCRF	SAE paper form
SAE* and SAE updates – after start of study drug	Start of study drug	Participation in study has ended	Within 24 hours of awareness	SAE eCRF	SAE paper form

**Event**	**Collection Start**	**Collection Stop**	**Timing for Reporting to Sponsor or Designee**	**Mechanism for Reporting**	**Back-up Method of Reporting**
SAE* – after participant's study participation has ended **and** the investigator becomes aware	After participant's study participation has ended	N/A	Promptly	SAE paper form	N/A
**Pregnancy**					
Pregnancy in female participants and female partners of male participants	After the start of study drug	Four months after the last injection for female partners of male participants and 2 months after the last injection for female participants	Within 24 hours of learning of the pregnancy	SAE eCRF	SAE paper form
**Product Complaints**					
PC associated with an SAE or might have led to an SAE	Start of study drug	End of study drug	Within 24 hours of awareness	Product Complaint form	N/A
PC not associated with an SAE	Start of study drug	End of study drug	Within 1 business day of awareness	Product Complaint form	N/A

Event	Collection Start	Collection Stop	Timing for Reporting to Sponsor or Designee	Mechanism for Reporting	Back-up Method of Reporting
Updated PC information	—	—	As soon as possible upon site awareness	Originally completed Product Complaint form with all changes signed and dated by the investigator	N/A
PC (if investigator becomes aware)	Participation in study has ended	N/A	Promptly	Product Complaint form	N/A

Abbreviations: eCRF = electronic case report form; N/A = not applicable; PC = product complaint; SAE = serious adverse event.

*Serious adverse events, including death, caused by disease progression as described in Section 8.3.2 should not be reported unless the investigator deems them to be possibly related to study drug.

8.3.2. Primary, Secondary, and Additional Study Endpoint Reporting

The following investigator-reported events are considered potential endpoints and must be reported first as an AE on the AE eCRF (with the appropriate designation for seriousness). They must then be reported as an endpoint on the eCRF with all required source documents provided for adjudication to the CEC. These potential endpoints (even if they meet criteria for a serious event) are not to be reported on the SAE eCRF unless considered as possibly related to study drug, the drug delivery system, or study procedure. Potential endpoints that are serious and considered as possibly related to study drug, the drug delivery system, or study procedure must also be reported as an SAE using the SAE eCRF:

- all-cause mortality (death), and
- hospitalization for HF or an urgent HF visit.

In the case where 1 of the above endpoint events is reported but does not meet a prespecified event definition detailed in the CEC charter, as reviewed by the independent CEC, no further action will be taken by the study site.

8.3.3. Adverse Events of Special Interest

The following are AESI and will be adjudicated by an external adjudication committee. This committee will be blinded to treatment assignment.

- pancreatitis

- major adverse CV events (see Section 10.5.1.5), and
- deaths

The following are additional AESI for this program that will not be adjudicated by an external committee:

- hepatobiliary disorders
- severe hypoglycemia
- thyroid malignancies and C-cell hyperplasia
- supraventricular arrhythmias and cardiac conduction disorders
- allergic/hypersensitivity reactions, including injection site reactions and ADA formation
- severe GI AEs, and
- acute renal events.

Sites should collect additional details and data regarding AESI, as instructed on the applicable eCRFs, and detailed in Section 10.5.

The details on the definition of AESI will be provided in SAP.

8.4. Treatment of Overdose

Considering the mechanism of action of tirzepatide, potential overdose effects can be GI disorders and hypoglycemia. In the event of overdose, appropriate supportive treatment should be initiated according to the participant's clinical signs and symptoms. In the event of overdose, refer to the IB for tirzepatide.

8.5. Pharmacokinetics

Pharmacokinetic samples will be collected from all participants in this study.

Tirzepatide plasma concentrations will be determined from blood samples obtained from participants receiving tirzepatide treatment. Blood samples collected from participants assigned to the placebo arm will not be included in the bioanalysis of drug concentrations.

Blood samples for PK assessment will be collected prior to the dose administration and at the same time as the planned immunogenicity samples (that is, at Week 0 and then at Weeks 4, 12, 24, and 52 per the Study Schedule or additionally at follow-up and ET (reference SoA).

Samples will be analyzed at a laboratory approved by the sponsor and stored at a facility designated by the sponsor.

Concentrations of tirzepatide will be assayed using a validated liquid chromatography mass spectrometry method. Bioanalytical samples collected to measure tirzepatide concentrations will be retained for a maximum of 1 year following last participant visit for the study (Section 10.1.10). During this time, samples remaining after the bioanalyses may be used for exploratory analyses such as metabolism work, protein binding, and/or bioanalytical method cross-validation.

8.6. Pharmacodynamics

Pharmacodynamic parameters will not be evaluated in this study.

8.7. Genetics

A whole blood sample will be collected for pharmacogenetic analysis where local regulations allow.

See Appendix 2, Clinical Laboratory Tests (Section 10.2), and Section 1.3 (SoA) for sample collection information.

See Section 10.6 for genetic research, custody, and sample retention information.

8.8. Biomarkers

Biomarker research is performed to address questions of relevance to drug disposition, target engagement, pharmacodynamics, mechanism of action, variability of participant response (including safety), and clinical outcome. Sample collection is incorporated into clinical studies to enable examination of these questions through measurement of biomolecules including DNA, RNA, proteins, lipids, and other cellular elements.

Serum and plasma for exploratory biomarker research will be collected at the time specified in the SoA (Section 1.3) where local regulations allow.

All samples will be coded with the participant number. These samples and any data generated can be linked back to the participant only by the investigator site personnel.

Samples will be stored and analysis may be performed on biomarkers thought to play a role in disease processes, mechanism of action of tirzepatide, pathways associated with HFpEF, and/or research methods validating diagnostic tools or assay(s) related to HFpEF and associated diseases. Biomarkers may be evaluated to determine their association with observed clinical responses to tirzepatide and the disease state.

Samples will be retained at a facility selected by the sponsor or its designee for the duration detailed in Section 10.1.10, or for a shorter period if local regulations and ERBs impose shorter time limits. This retention period enables use of new technologies, response to regulatory questions, and investigation of variable response that may not be observed until later in the development of tirzepatide or after tirzepatide becomes commercially available.

8.9. Immunogenicity Assessments

At the visits and times specified in the SoA (Section 1.3), venous blood samples will be collected to determine antibody production against tirzepatide. Antibodies may be further characterized for cross-reactive binding to endogenous counterparts (native GIP and GLP-1) and their ability to neutralize the activity of tirzepatide and endogenous counterparts. To interpret the results of immunogenicity, a venous blood sample will be collected at the same time points to determine the plasma concentrations of tirzepatide. All samples for immunogenicity should be taken predose when applicable and possible.

Samples will be retained for a maximum of 15 years after the last participant visit, or for a shorter period if local regulations and ERBs allow, at a facility selected by the sponsor. The duration allows the sponsor to respond to future regulatory requests related to tirzepatide. Any samples remaining after 15 years will be destroyed.

8.10. Medical Resource Utilization and Health Economics

Not applicable.

9. Statistical Considerations

9.1. Statistical Hypotheses

Two primary hypotheses will be tested in this study:

- Tirzepatide MTD is superior to placebo for the hierarchical composite endpoint of all-cause mortality, occurrence of adjudicated HF events (including HF hospitalization or urgent HF visit) through the end of the treatment period, the change from baseline in 6MWD, and the change from baseline in KCCQ-CSS at Week 52.
- Tirzepatide MTD is superior to placebo for the change from baseline in 6MWD at Week 52.

Key secondary hypotheses (all under multiplicity control) are that tirzepatide MTD is superior to placebo with regards to

- percent change from baseline in body weight at Week 52
- change from baseline in KCCQ-CSS at Week 52
- change from baseline in 6MWD at Week 24, and
- proportion of participants with NYHA class change at Week 52.

All primary and key secondary hypotheses will be tested with the overall family-wise type I error rate at a 2-sided alpha level of 0.05 through the multiplicity control approach based on the graphical multiple testing procedure. For the 2 primary hypotheses, the hierarchical composite endpoint will be tested at a 2-sided alpha level of 0.04, and change in 6MWD will be tested at a 2-sided alpha level of 0.01 in parallel for statistical significance. If either is significant, the corresponding assigned alpha will be propagated to test the other primary efficacy endpoint at a 2-side alpha level of 0.05. The detailed graphical testing scheme will be outlined in the SAP.

9.2. Sample Size Determination

Approximately 700 participants will be randomized to either tirzepatide or placebo in a 1:1 ratio (approximately 350 participants per treatment arm), and the statistical powers are evaluated for each of the 2 primary efficacy endpoints at a 2-sided significance level of 0.01 to ensure that the sample size and power are sufficient for registration purposes. This sample size will provide

- approximately 85% power or higher to demonstrate superiority of tirzepatide MTD to placebo for the hierarchical composite endpoint using Finkelstein-Schoenfeld method (Finkelstein and Schoenfeld 1999)
- approximately 95% power for the change from baseline in 6MWD using Wilcoxon rank sum test, and
- more than 95% power to demonstrate superiority for at least one of above.

The sample size and power are estimated through simulations under the following assumptions:

Variable	Placebo	Tirzepatide MTD	Treatment Difference
All-cause mortality (n per 100 yr)	5	5 or 4.75	0 or -0.25 (0 or 5% RRR)
First HF events (n per 100 yr)	10	8 or 7	-2 or -3 (20% or 30% RRR)
6MWD – change from baseline (mean±SD)	0±75	30±85	30
KCCQ-CSS – change from baseline (mean±SD)	5±19	10±19	5

Abbreviations: 6MWD = 6-minute walk test distance; HF = heart failure; KCCQ-CSS = Kansas City Cardiomyopathy Questionnaire – Clinical Summary Scale; MTD = maximum tolerated dose; n = number; RRR = relative risk reduction; SD = standard deviation.

In addition, the following imputation rules for 6MWD and KCCQ-CSS at Week 52 are also built into the simulations: worst case imputation (assign 0 steps or a score of 0) for missing due to death (assume 5% death rate) and placebo imputation for 10% participants who discontinue study treatment due to AE.

9.3. Populations for Analyses

The following populations are defined:

Analysis Population	Description
Entered	All participants who sign the informed consent form
Randomized/Intent-to-Treat (ITT) Population	All participants assigned to treatment, regardless of whether they take any doses of study treatment, or if they took the correct treatment. Participants will be analyzed according to the treatment group to which they were assigned.
Safety Population	All participants in ITT population who take at least 1 dose of study treatment. Participants will be analyzed according to the treatment group to which they were assigned.

9.4. Statistical Analyses

9.4.1. General Considerations

Statistical analysis of this study will be the responsibility of sponsor or its designee.

Unless specified otherwise, all tests of treatment effects will be conducted at a 2-sided alpha level of 0.05 and all confidence intervals will be given at a 2-sided 95% level. Efficacy will be assessed using ITT Population and safety will be assessed using the Safety Population.

Any change to the data analysis methods described in the protocol will require an amendment only if it changes a principal feature of the protocol. Any other change to the data analysis methods described in the protocol, and the justification for making the change, will be described in the SAP and the CSR. Additional exploratory analyses of the data will be conducted as deemed appropriate.

The SAP will be completed prior to first unblinding and any subsequent amendments will be documented, with final amendments finalized prior to final database lock. The SAP will include a more technical and detailed description of the statistical analyses described in this section. This section is a summary of the planned statistical analyses of the most important endpoints including primary and key secondary endpoints.

9.4.2. Primary Endpoint(s)

The primary estimand for primary endpoints is to assess the treatment difference between tirzepatide and placebo relative to the efficacy measures for all randomized participants, and treatment policy strategy will be used to handle all intercurrent events, which is, all the observed values for the variable of interest are used regardless of whether the intercurrent event occurs. The endpoint and population-level summary for the estimand is described in Section 9.4.2.1 and Section 9.4.2.2 for each primary endpoint.

9.4.2.1. The Hierarchical Composite Endpoint

The analysis of the primary hierarchical composite endpoint will be performed with the Finkelstein-Schoenfeld method, and the win ratio (Pocock et al. 2012) will be reported as the measure of treatment effect. The population-level summary of win ratio will be calculated as number of pairs of tirzepatide-treated participant "wins" divided by number of pairs of placebo-treated participant "wins."

The Finkelstein-Schoenfeld method is based on the principle that each participant is compared with every other participant within each stratum in a pairwise manner that proceeds in a hierarchical fashion. Participants will be stratified according to HF decompensation within 12 months of screening (Y/N), diagnosed T2DM (Y/N), and baseline BMI ≥35 kg/m^2 (Y/N), yielding 8 stratification pools. For the primary composite endpoint, each pairwise comparison will proceed in the following order and a winner has:

- A delayed first occurrence of all-cause death;
- If the pair cannot be differentiated based on mortality, a winner has fewer HF events;
- If the pair cannot be differentiated by number of HF events, a winner has delayed time to the occurrence of first HF event;
- If the pair still cannot be differentiated, a winner has a more favorable category for change from baseline in 6MWD at Week 52;
- If the pair still cannot be differentiated, a winner has a more favorable category for change from baseline in KCCQ-CSS at Week 52;

- Otherwise the pair will be recorded as tied.

The categories for change from baseline in 6MWD are: 1) ≥30% worsening; 2) ≥20% and <30% worsening; 3) ≥10% and <20% worsening; 4) No change (less than 10% change); 5) ≥10% and <20% improvement; 6) ≥20% and <30% improvement; and 7) ≥30% improvement.

The categories for change from baseline in KCCQ-CSS are: 1) ≥10-point worsening; 2) ≥5- but <10-point worsening; 3) No change (<5-point change); 4) ≥5- but <10-point improvement; 5) ≥10-point improvement.

In the pairwise comparison for all-cause mortality and HF events, the censoring for death and HF events will be handled based on the win ratio method (Pocock et al. 2012). When 2 participants have different follow-up times, the shorter follow-up time will be used to compare the clinical outcome measure. Only adjudicated and confirmed endpoint events are included in the primary analysis.

The last measurement prior to randomization for 6MWD and KCCQ-CSS will be used as baseline. Missing 6MWD and KCCQ-CSS values at Week 52 will be imputed through multiple imputations based on the reason of missingness with details described in the SAP; the statistical inference over multiple imputations will be guided by the method proposed by Rubin (1987).

9.4.2.2. Change from Baseline in 6-Minute Walk Test Distance

For the primary endpoint of change from baseline to Week 52 in 6MWD, a stratified Wilcoxon (Van Elteren) test will be used as the primary analysis method, controlling for the stratification factors of HF decompensation within 12 months of screening (Y/N), diagnosed T2DM (Y/N), and baseline BMI ≥35 kg/m^2 (Y/N). Population-level summary of Hodges-Lehmann estimate for the median difference and corresponding confidence interval will be reported.

Missing 6MWD at Week 52 will be imputed through multiple imputations based on the reason of missingness with details described in the SAP. The statistical inference over multiple imputations will be guided by the method proposed by Rubin (1987).

9.4.3. Key Secondary Endpoint(s)

Analyses for the key secondary endpoints will also be guided by the treatment policy strategy.

Change from baseline in KCCQ-CSS at Week 52 and change from baseline in 6MWD at Week 24 will be analyzed using the same nonparametric approach as described in Section 9.4.2.2.

Percent change from baseline in body weight will be analyzed using an MMRM analysis. The MMRM model will include the categorical effect of treatment, time, treatment-by-time interaction, stratification factors, and the continuous covariate of baseline body weight value. Missing data will be imputed through multiple imputations based on the reason of missingness with details described in the SAP.

Change in NYHA class (improved, no change, or worsened) from baseline will be analyzed using a longitudinal proportional odds model. Missing data will be imputed based on the reason of missingness as described in the SAP.

9.4.4. Tertiary/Exploratory Endpoint(s)

The analyses for exploratory endpoints will be described in the SAP. Statistical tests will be performed at the two-sided significance level of 0.05. There will be no multiplicity adjustment for any analysis of exploratory variables unless specified otherwise. Missing values will not be explicitly imputed unless specified otherwise.

9.4.5. Other Safety Analyses

Safety will be assessed by summarizing and analyzing AEs, special safety topics, laboratory analytes, and vital signs. All safety analyses will be made on the Safety Population. Unless specified otherwise, all data obtained during study period from Safety Population, regardless of adherence to study drug, will be used for safety analyses. The details for safety analysis will be described in the SAP.

Adverse events will be coded from the actual term using MedDRA and reported with preferred terms and system organ class.

9.4.5.1. Evaluation of Immunogenicity

The frequency and percentage of participants with preexisting ADAs and with treatment-emergent ADAs to tirzepatide will be tabulated. Treatment-emergent ADAs are defined as those with a titer 2-fold (1 dilution) greater than the minimum required dilution of the ADA assay if no ADAs were detected at baseline (treatment-induced ADA), or those with a 4-fold (2 dilutions) increase in titer compared with baseline if ADAs were detected at baseline (treatment-boosted ADA). The details of analyses for immunogenicity will be specified in SAP.

9.4.6. Subgroup Analyses

Subgroup variables to be evaluated for the primary efficacy endpoint may include demography (for example, race, ethnicity), baseline disease characteristics (for example, diagnosed T2DM) and others. Subgroup analyses may also be performed for selected key secondary efficacy endpoints. Details for the subgroup analyses will be provided in the SAP.

9.5. Interim Analyses

Based on the projected enrollment, approximately 4 interim analyses of safety will be conducted. The first interim analysis is planned to occur when approximately 20% of the anticipated number of participants are randomized or 6 months after the first participant is randomized, whichever occurs later, followed by subsequent reviews approximately every 6 months throughout the study.

The DMC is authorized to evaluate unblinded interim analyses. Study sites will receive information about interim results only if they need to know for the safety of their participants.

Unblinding details are specified in a separate unblinding plan document.

The DMC charter will describe the planned interim analyses in detail.

9.6. Data Monitoring Committee

An independent DMC with members all external to the sponsor will be used to monitor participant safety in an unblinded fashion. For details on the DMC, refer to the DMC charter.

10. Supporting Documentation and Operational Considerations

10.1. Appendix 1: Regulatory, Ethical, and Study Oversight Considerations

10.1.1. Regulatory and Ethical Considerations

- This study will be conducted in accordance with the protocol and with the following:
  - Consensus ethical principles derived from international guidelines including the Declaration of Helsinki and CIOMS International Ethical Guidelines
  - Applicable ICH GCP guidelines
  - International Organization for Standardization (ISO) 14155
  - Applicable laws and regulations
- The protocol, protocol amendments, ICF, IB, and other relevant documents (for example, advertisements) must be submitted to an IRB/IEC by the investigator and reviewed and approved by the IRB/IEC before the study is initiated.
- Any amendments to the protocol will require IRB/IEC approval before implementation of changes made to the study design, except for changes necessary to eliminate an immediate hazard to study participants.
- Protocols and any substantial amendments to the protocol will require health authority approval prior to initiation except for changes necessary to eliminate an immediate hazard to study participants.
- The investigator will be responsible for the following:
  - Providing written summaries of the status of the study to the IRB/IEC annually or more frequently in accordance with the requirements, policies, and procedures established by the IRB/IEC
  - Notifying the IRB/IEC of SAEs or other significant safety findings as required by IRB/IEC procedures
  - Providing oversight of study conduct for participants under their responsibility and adherence to requirements of 21 CFR, ICH guidelines, the IRB/IEC, European regulation 536/2014 for clinical studies (if applicable), and all other applicable local regulations
- Investigator sites are compensated for participation in the study as detailed in the clinical trial agreement.

10.1.2. Financial Disclosure

Investigators and sub-investigators will provide the sponsor with sufficient, accurate financial information as requested to allow the sponsor to submit complete and accurate financial

certification or disclosure statements to the appropriate regulatory authorities. Investigators are responsible for providing information on financial interests during the course of the study and for 1 year after completion of the study.

10.1.3. Informed Consent Process

The investigator or his/her representative will explain the nature of the study, including the risks and benefits, to the participant or his/her legally authorized representative and answer all questions regarding the study.

Participants must be informed that their participation is voluntary. Participants or their legally authorized representative will be required to sign a statement of informed consent that meets the requirements of 21 CFR 50, local regulations, ICH guidelines, HIPAA requirements, where applicable, and the IRB/IEC or study center.

The medical record must include a statement that written informed consent was obtained before the participant was entered in the study and the date the written consent was obtained. The authorized person obtaining the informed consent must also sign the ICF.

Participants must be re-consented to the most current version of the ICF(s) during their participation in the study.

A copy of the ICF(s) must be provided to the participant or the participant's legally authorized representative and is kept on file.

Participants who are rescreened are required to sign a new ICF.

10.1.4. Data Protection

Participants will be assigned a unique identifier by the sponsor. Any participant records, datasets, or tissue samples that are transferred to the sponsor will contain the identifier only; participant names or any information which would make the participant identifiable will not be transferred.

The participant must be informed that the participant's personal, study-related data will be used by the sponsor in accordance with local data protection law. The level of disclosure must also be explained to the participant who will be required to give consent for his/her data to be used as described in the informed consent.

The participant must be informed that their medical records may be examined by Clinical Quality Assurance auditors or other authorized personnel appointed by the sponsor, by appropriate IRB/IEC members, and by inspectors from regulatory authorities.

The sponsor has processes in place to ensure data protection, information security, and data integrity. These processes include appropriate contingency plan(s) for appropriate and timely response in the event of a data security breach.

10.1.5. Dissemination of Clinical Study Data

Report Preparation

An investigator will sign the final CSR for this study, indicating agreement that, to the best of his or her knowledge, the report accurately describes the conduct and results of the study.

Public Access to Reports and Data

Reports

The sponsor will disclose a summary of study information, including tabular study results, on publicly available websites where required by local law or regulation.

Data

The sponsor provides access to all individual participant data collected during the trial, after anonymization, with the exception of PK, immunogenicity, or genetic data. Data are available to request 6 months after the indication studied has been approved in the United States and after primary publication acceptance, whichever is later. No expiration date of data requests is currently set once they are made available. Access is provided after a proposal has been approved by an independent review committee identified for this purpose and after receipt of a signed data sharing agreement. Data and documents, including the study protocol, SAP, CSR, and blank or annotated CRFs, will be provided in a secure data sharing environment for up to 2 years per proposal. For details on submitting a request, see the instructions provided at www.clinicalstudydatarequest.com.

Publications/Publication Policy

The publication policy is described in Section 10.1.9.

10.1.6. Data Quality Assurance

All participant data relating to the study will be recorded on printed or electronic CRF unless transmitted to the sponsor or designee electronically (for example, laboratory data or an electronic source, such as eCOA). The investigator is responsible for verifying that data entries are accurate and correct by physically or electronically signing the CRF.

The investigator must maintain accurate documentation (source data) that supports the information entered in the CRF.

The investigator must permit study-related monitoring, audits, IRB/IEC review, and regulatory agency inspections and provide direct access to source data documents.

Monitoring details describing strategy (for example, risk-based initiatives in operations and quality such as risk management and mitigation strategies and analytical risk-based monitoring), methods, responsibilities, and requirements, including handling of noncompliance issues and monitoring techniques, are provided in the Monitoring Plan.

The sponsor or designee is responsible for the data management of this study including quality checking of the data.

The sponsor assumes accountability for actions delegated to other individuals (for example, contract research organizations).

Study monitors will perform ongoing source data verification to confirm that data transcribed into the CRF by authorized site personnel are accurate, complete, and verifiable from source documents; that the safety and rights of participants are being protected; and that the study is being conducted in accordance with the currently approved protocol and any other study agreements, ICH GCP, and all applicable regulatory requirements.

Records and documents, including signed ICFs, pertaining to the conduct of this study must be retained by the investigator for the time period outlined in the clinical trial agreement unless local regulations or institutional policies require a longer retention period. No records may be destroyed during the retention period without the written approval of the sponsor. No records may be transferred to another location or party without written notification to the sponsor.

In addition, the sponsor or its representatives will periodically check a sample of the participant data recorded against source documents at the study site. The study may be audited by the sponsor or its representatives, and/or regulatory agencies at any time. Investigators will be given notice before an audit occurs.

Data Capture System

The investigator is responsible for ensuring the accuracy, completeness, legibility, and timeliness of the data reported to the sponsor.

An EDC system will be used in this study for the collection of CRF data. The investigator maintains a separate source for the data entered by the investigator or designee into the sponsor-provided EDC system. The investigator is responsible for the identification of any data to be considered source and for the confirmation that data reported are accurate and complete by signing the CRF.

Additionally, eCOA data (patient-reported outcomes instruments) will be directly recorded by the participant, into a device (for example, hand-held smart phone or tablet). The eCOA data will serve as the source documentation, and the investigator does not maintain a separate, written or electronic record of these data.

Data collected via the sponsor-provided data capture systems will be stored at third parties. The investigator will have continuous access to the data during the study and until decommissioning of the data capture system(s). Prior to decommissioning, the investigator will receive an archival copy of pertinent data for retention.

Data managed by a central vendor, such as laboratory test data, will be stored electronically in the central vendor's database system, and reports (as applicable) will be provided to the investigator for review and retention. Data will subsequently be transferred from the central vendor to the sponsor data warehouse.

Data from complaint forms submitted to the sponsor will be encoded and stored in the global product complaint management system.

10.1.7. Source Documents

Source documents provide evidence for the existence of the participant and substantiate the integrity of the data collected. Source documents are filed at the investigator's site.

Data reported on the CRF or entered in the eCRF that are transcribed from source documents must be consistent with the source documents, or the discrepancies must be explained. The investigator may need to request previous medical records or transfer records, depending on the study. Also, current medical records must be available.

Definition of what constitutes source data can be found in Section 10.1.6.

10.1.8. Study and Site Start and Closure

The study start date is the date on which the clinical study will be open for recruitment of participants.

The first act of recruitment is the first site open and will be the study start date.

The sponsor designee reserves the right to close the study site or terminate the study at any time for any reason at the sole discretion of the sponsor. Study sites will be closed upon study completion. A study site is considered closed when all required documents and study supplies have been collected and a study-site closure visit has been performed.

The investigator may initiate study-site closure at any time, provided there is reasonable cause and sufficient notice is given in advance of the intended termination.

Reasons for the early closure of a study site by the sponsor or investigator may include but are not limited to:

- Failure of the investigator to comply with the protocol, the requirements of the IRB/IEC or local health authorities, the sponsor's procedures, or GCP guidelines
- Inadequate recruitment of participants by the investigator
- Discontinuation of further study drug development.

If the study is prematurely terminated or suspended, the sponsor shall promptly inform the investigators, the IECs/IRBs, the regulatory authorities, and any contract research organization(s) used in the study of the reason for termination or suspension, as specified by the applicable regulatory requirements. The investigator shall promptly inform the participant and assures appropriate participant therapy and/or follow-up.

10.1.9. Publication Policy

In accordance with the sponsor's publication policy, the results of this study will be submitted for publication by a peer-reviewed journal.

10.1.10. Long-Term Sample Retention

Sample retention enables use of new technologies, response to regulatory questions, and investigation of variable response that may not be observed until later in the development of the intervention or after the intervention becomes commercially available.

This table describes the retention period for potential sample types.

Sample Type	Custodian	Retention Period After Last Participant Visit
Genetics sample	Sponsor or designee	up to 15 years
Exploratory biomarker sample	Sponsor or designee	up to 15 years
Immunogenicity (antidrug antibody) sample	Sponsor or designee	up to 15 years
Pharmacokinetic sample	Sponsor or designee	up to 1 years

10.1.11. Investigator Information

Researchers with appropriate education, training, and experience, as determined by the Sponsor, will participate as investigators in this clinical trial

10.2. Appendix 2: Clinical Laboratory Tests

- Clinical laboratory testing will be performed according to the SoA (Section 1.3).
- Central and local laboratories will be used. The table below describes when the local or central laboratory will be used
- In circumstances where the sponsor approves local laboratory testing in lieu of central laboratory testing (in the table below), the local laboratory must be qualified in accordance with applicable local regulations.
- Protocol-specific requirements for inclusion or exclusion of participants are detailed in Section 5 of the protocol.
- Additional tests may be performed at any time during the study as determined necessary by the investigator or required by local regulations.
- Pregnancy testing will be performed according to the SoA.
- Investigators must document their review of the laboratory safety results. Laboratory results that will not be reported to investigative sites or other blinded personnel are noted in the table below.

Refer to Section 10.7 for recommended laboratory testing for hypersensitivity events.

Clinical Laboratory Tests	Comments
**Hematology**	Assayed by Lilly-designated laboratory.
Hemoglobin	
Hematocrit	
Erythrocyte count (RBCs [red blood cells])	
Mean cell volume	
Mean cell hemoglobin concentration	
Leukocytes (WBCs [white blood cells])	
Differential	
Neutrophils, segmented	
Lymphocytes	
Monocytes	
Eosinophils	
Basophils	
Platelets	
**Clinical Chemistry**	Assayed by Lilly-designated laboratory.
Sodium	
Potassium	
Chloride	
Bicarbonate	

Clinical Laboratory Tests	Comments
Total bilirubin	
Direct bilirubin	
Alkaline phosphatase (ALP)	
Alanine aminotransferase (ALT)	
Aspartate aminotransferase (AST)	
Gamma-glutamyl transferase (GGT)	
Blood urea nitrogen (BUN)	
Creatinine	
Creatine kinase (CK)	
Uric acid	
Total protein	
Albumin	
Calcium	
Phosphorus	
Glucose	
**Lipids**	
Total cholesterol	
Direct LDL-C	
HDL-C	
VLDL-C	
Triglycerides	
**Pancreas (Exocrine)**	Assayed by Lilly-designated laboratory.
Pancreatic amylase	
Lipase	
**Special Chemistry**	Assayed by Lilly-designated laboratory.
Hemoglobin A1c (HbA1c)	
Calcitonin	
Cystatin C	
N-terminal pro b-type natriuretic peptide (NT-proBNP)	
Cardiac troponin T (cTnT)	
C-reactive protein, high-sensitivity (hsCRP)	
Thyroid stimulating hormone	
**Urine Chemistry**	Assayed by Lilly-designated laboratory.
Albumin	
Creatinine	
**Calculation**	
eGFR (calculated by CKD-EPI equation)	Will be calculated by the Lilly-designated laboratory at all visits.
Urine albumin, creatinine, UACR	
**Hormones (female)**	
Urine Pregnancy	Local laboratory
Serum Pregnancy	Assayed by Lilly-designated laboratory.

Clinical Laboratory Tests	Comments
Follicle Stimulating Hormone (FSH)	Assayed by Lilly-designated laboratory.
**Pharmacokinetic Samples**	• Assayed by Lilly-designated laboratory.<br>• Results will not be provided to the investigative sites.<br>• In the event of systemic drug hypersensitivity reactions (immediate or nonimmediate), additional blood samples will be collected including ADA, PK, and exploratory biomarker samples. PK samples for immunogenicity must be taken prior to drug administration.
**Genetics sample**<br>Whole blood (EDTA)	• Assayed by Lilly-designated laboratory.<br>• Results will not be provided to the investigative sites.
**Exploratory Biomarker Samples**	• Assayed by Lilly-designated laboratory.<br>• Results will not be provided to the investigative sites.
Serum	
EDTA Plasma	
P800 Plasma	
**Immunogenicity Samples**	
Anti-tirzepatide antibodies<br>Anti-tirzepatide neutralizing antibodies	• Assayed by Lilly-designated laboratory.<br>• Results will not be provided to the investigative sites.<br>• In the event of systemic drug hypersensitivity reactions (immediate or nonimmediate), additional blood samples will be collected including ADA, PK, and exploratory biomarker samples. PK samples for immunogenicity must be taken prior to drug administration.

Abbreviations: ADA = antidrug antibody; CKD-EPI = Chronic Kidney Disease-Epidemiology Collaboration; EDTA = ethylenediaminetetraacetic acid; eGFR = estimated glomerular filtration rate; HDL-C = high-density lipoprotein cholesterol; LDL-C = low-density lipoprotein cholesterol; Lilly = Eli Lilly and company; PK = pharmacokinetic; UACR = urine albumin/creatinine ratio; VLDL-C = very low-density lipoprotein cholesterol.

10.3. Appendix 3: Adverse Events: Definitions and Procedures for Recording, Evaluating, Follow-up, and Reporting

The definitions and procedures detailed in this appendix are in accordance with ISO 14155.

Both the investigator and the sponsor will comply with all local medical device reporting requirements.

The detection and documentation procedures described in this protocol apply to all sponsor medical devices provided for use in the study. See Section 6.1.1 for the list of sponsor medical devices).

10.3.1. Definition of AE

**AE Definition**
• An AE is any untoward medical occurrence in a participant administered a pharmaceutical product and which does not necessarily have a causal relationship with the study drug. An AE can therefore be any unfavourable and unintended sign (including an abnormal laboratory finding), symptom, or disease (new or exacerbated) temporally associated with the use of a medicinal (investigational) product, whether or not related to the medicinal (investigational) product.<br>• An AE is any untoward medical occurrence, unintended disease or injury, or untoward clinical signs (including abnormal laboratory finding) in study participants, users, or other persons, whether or not related to the investigational medical device. This definition includes events related to the investigational medical device or comparator and events related to the procedures involved except for events in users or other persons, which only include events related to investigational devices.
**Events Meeting the AE Definition**
• Any abnormal laboratory test results (hematology, clinical chemistry, or urinalysis) or other safety assessments (for example, ECG, radiological scans, vital signs measurements), including those that worsen from baseline, considered clinically significant in the medical and scientific judgment of the investigator (that is, not related to progression of underlying disease).<br>• Exacerbation of a chronic or intermittent preexisting condition including either an increase in frequency and/or intensity of the condition.<br>• New conditions detected or diagnosed after study drug administration even though they may have been present before the start of the study.<br>• Signs, symptoms, or the clinical sequelae of a suspected drug-drug interaction.<br>• Signs, symptoms, or the clinical sequelae of a suspected overdose of either study drug or a concomitant medication. Overdose per se will not be reported as an AE/SAE unless it is an intentional overdose taken with possible suicidal/self-harming intent. Such an overdose should be reported regardless of sequelae.<br>• “Lack of efficacy” or “failure of expected pharmacological action” per se will not be reported as an AE or SAE. Such instances will be captured in the efficacy assessments.

However, the signs, symptoms, and/or clinical sequelae resulting from lack of efficacy will be reported as an AE or SAE if they fulfill the definition of an AE or SAE.
**Events NOT Meeting the AE Definition**
• Any clinically significant abnormal laboratory findings or other abnormal safety assessments which are associated with the underlying disease, unless judged by the investigator to be more severe than expected for the participant's condition.<br>• The disease/disorder being studied or expected progression, signs, or symptoms of the disease/disorder being studied, unless more severe than expected for the participant's condition.<br>• Medical or surgical procedure (for example, endoscopy, appendectomy): the condition that leads to the procedure is the AE.<br>• Situations in which an untoward medical occurrence did not occur (social and/or convenience admission to a hospital).<br>• Anticipated day-to-day fluctuations of preexisting disease(s) or condition(s) present or detected at the start of the study that do not worsen.

10.3.2. Definition of SAE

If an event is not an AE per the definition above, then it cannot be an SAE even if serious conditions are met (for example, hospitalization for signs/symptoms of the disease under study, death due to progression of disease).

**SAE is defined as any untoward medical occurrence that, at any dose:**
**a. Results in death**
**b. Is life-threatening**<br>The term 'life-threatening' in the definition of 'serious' refers to an event in which the participant was at risk of death at the time of the event. It does not refer to an event, which hypothetically might have caused death, if it were more severe.
**c. Requires inpatient hospitalization or prolongation of existing hospitalization**<br>• In general, hospitalization signifies that the participant has been admitted to the hospital for observation and/or treatment that would not have been appropriate in the physician's office or outpatient setting. Complications that occur during hospitalization are AEs. If a complication prolongs hospitalization or fulfills any other serious criteria, the event is serious. When in doubt as to whether "hospitalization" occurred or was necessary, the AE should be considered serious.<br>• Hospitalization for elective treatment of a preexisting condition that did not worsen from baseline is not considered an AE.
**d. Results in persistent disability/incapacity**

• The term disability means a substantial disruption of a person's ability to conduct normal life functions.<br>• This definition is not intended to include experiences of relatively minor medical significance such as uncomplicated headache, nausea, vomiting, diarrhea, influenza, and accidental trauma (for example, sprained ankle) which may interfere with or prevent everyday life functions but do not constitute a substantial disruption.
**e. Is a congenital anomaly/birth defect**<br>• Abnormal pregnancy outcomes (for example, spontaneous abortion, fetal death, stillbirth, congenital anomalies, ectopic pregnancy) are considered SAEs.
**f. Other situations:**<br>• Medical or scientific judgment should be exercised in deciding whether SAE reporting is appropriate in other situations such as important medical events that may not be immediately life-threatening or result in death or hospitalization but may jeopardize the participant or may require medical or surgical intervention to prevent one of the other outcomes listed in the above definition. These events should usually be considered serious.<br>• Examples of such events include invasive or malignant cancers; intensive treatment in an emergency room or at home for allergic bronchospasm, blood dyscrasias, or convulsions that do not result in hospitalization; or development of drug dependency or drug abuse.
**g.** Resulted in medical or surgical intervention to prevent life-threatening illness or injury or permanent impairment to a body structure or a body function.
**Definition of Serious Adverse Device Effect (SADE)**<br>An SADE is defined as an adverse device effect that has resulted in any of the consequences characteristic of a serious adverse event.<br>**Definition of Unanticipated Adverse Device Effect (UADE)**<br>A UADE is a serious adverse effect on health or safety or any life-threatening problem or death caused by or associated with a device, if that effect, problem, or death was not previously identified in nature, severity, or degree of incidence in the investigational plan or application (including a supplementary plan or application) or any other unanticipated serious problem associated with a device that relates to the rights, safety, or welfare of the participant.

10.3.3. Definition of Product Complaints

Product Complaint
• A product complaint is any written, electronic, or oral communication that alleges deficiencies related to the identity, quality, durability, reliability, safety, effectiveness, or performance of a study drug. When the ability to use the study drug safely is impacted, the following are also product complaints:<br>o Deficiencies in labeling information, and<br>o Use errors for device or drug-device combination products due to ergonomic design elements of the product.<br>• Product complaints related to study drugs used in clinical trials are collected in order to ensure the safety of participants, monitor quality, and to facilitate process and product improvements.<br>• Investigators will instruct participants to contact the site as soon as possible if he or she has a product complaint or problem with the study drug so that the situation can be assessed.<br>• An event may meet the definition of both a product complaint and an AE/SAE. In such cases, it should be reported as both a product complaint and as an AE/SAE.

10.3.4. Recording and Follow-Up of AE and/or SAE and Product Complaints

AE, SAE, and Product Complaint Recording
When an AE/SAE/product complaint occurs, it is the responsibility of the investigator to review all documentation (for example, hospital progress notes, laboratory reports, and diagnostics reports) related to the event.<br>The investigator will then record all relevant AE/SAE/product complaint information in the participant's medical records, in accordance with the investigator's normal clinical practice. AE/SAE information is reported on the appropriate eCRF page and product complaint information is reported on the Product Complaint Form.<br>Note: An event may meet the definition of both a product complaint and an AE/SAE. In such cases, it should be reported as both a product complaint and as an AE/SAE.<br>It is **not** acceptable for the investigator to send photocopies of the participant's medical records to sponsor or designee in lieu of completion of the eCRF page for AE/SAE and the Product Complaint Form for product complaints.<br>There may be instances when copies of medical records for certain cases are requested by sponsor or designee. In this case, all participant identifiers, with the exception of the participant number, will be redacted on the copies of the medical records before submission to sponsor or designee.<br>The investigator will attempt to establish a diagnosis of the event based on signs, symptoms, and/or other clinical information. Whenever possible, the diagnosis (not the individual signs/symptoms) will be documented as the AE/SAE.

Assessment of Intensity
The investigator will make an assessment of intensity for each AE and SAE reported during the study and assign it to one of the following categories:<br>• Mild: A type of adverse event that is usually transient and may require only minimal treatment or therapeutic intervention. The event does not generally interfere with usual activities of daily living.<br>• Moderate: A type of adverse event that is usually alleviated with additional specific therapeutic intervention. The event interferes with usual activities of daily living, causing discomfort but poses no significant or permanent risk of harm to the research participant.<br>• Severe: A type of adverse event that interrupts usual activities of daily living, or significantly affects clinical status, or may require intensive therapeutic intervention. An AE that is assessed as severe should not be confused with an SAE. Severe is a category utilized for rating the intensity of an event; and both AEs and SAEs can be assessed as severe.<br>An event is defined as 'serious' when it meets at least 1 of the predefined outcomes as described in the definition of an SAE, NOT when it is rated as severe.
**Assessment of Causality**
• The investigator is obligated to assess the relationship between study drug and each occurrence of each AE/SAE.<br>• A "reasonable possibility" of a relationship conveys that there are facts, evidence, and/or arguments to suggest a causal relationship, rather than a relationship cannot be ruled out.<br>• The investigator will use clinical judgment to determine the relationship.<br>• Alternative causes, such as underlying disease(s), concomitant therapy, and other risk factors, as well as the temporal relationship of the event to study drug administration will be considered and investigated.<br>• The investigator will also consult the IB in his/her assessment.<br>• For each AE/SAE, the investigator **must** document in the medical notes that he/she has reviewed the AE/SAE and has provided an assessment of causality.<br>• There may be situations in which an SAE has occurred, and the investigator has minimal information to include in the initial report to sponsor or designee. However, it is very important that the investigator always make an assessment of causality for every event before the initial transmission of the SAE data to sponsor or designee.<br>• The investigator may change his/her opinion of causality in light of follow-up information and send an SAE follow-up report with the updated causality assessment.<br>• The causality assessment is one of the criteria used when determining regulatory reporting requirements.

Follow-up of AEs and SAEs
• The investigator is obligated to perform or arrange for the conduct of supplemental measurements and/or evaluations as medically indicated or as requested by sponsor or designee to elucidate the nature and/or causality of the AE or SAE as fully as possible. This may include additional laboratory tests or investigations, histopathological examinations, or consultation with other health care professionals.

10.3.5. Reporting of SAEs

SAE Reporting via an Electronic Data Collection Tool
• The primary mechanism for reporting an SAE will be the electronic data collection tool.<br>• If the electronic system is unavailable, then the site will use the paper SAE data collection tool (see next section) in order to report the event within 24 hours.<br>• The site will enter the SAE data into the electronic system as soon as it becomes available.<br>• After the study is completed at a given site, the electronic data collection tool will be taken offline to prevent the entry of new data or changes to existing data.<br>• If a site receives a report of a new SAE from a study participant or receives updated data on a previously reported SAE after the electronic data collection tool has been taken off-line, then the site can report this information on a paper SAE form (see next section) or to the sponsor by telephone.<br>• Contacts for SAE reporting can be found in the study training.

10.3.6. Regulatory Reporting Requirements

SAE Regulatory Reporting
• Prompt notification by the investigator to the sponsor of an SAE is essential so that legal obligations and ethical responsibilities towards the safety of participants and the safety of a study drug under clinical investigation are met.<br>• The sponsor has a legal responsibility to notify both the local regulatory authority and other regulatory agencies about the safety of a study drug under clinical investigation. The sponsor will comply with country-specific regulatory requirements relating to safety reporting to the regulatory authority, Institutional Review Boards (IRB)/Independent Ethics Committees (IEC), and investigators.<br>• An investigator who receives an investigator safety report describing an SAE or other specific safety information (for example, summary or listing of SAEs) from the sponsor will review and then file it along with the IB and will notify the IRB/IEC, if appropriate according to local requirements.<br>• As required by local regulations, the investigator will report to their IRB/IEC any UADE (unanticipated problem that resulted in an SAE), or any product complaint that could have led to an SAE had precautions not been taken.

10.4. Appendix 4: Contraceptive Guidance and Collection of Pregnancy Information

10.4.1. Male participants:

Men, regardless of their fertility status, with nonpregnant WOCBP partners must agree to either remain abstinent (if this is their preferred and usual lifestyle) or use condoms, as well as 1 additional highly effective (<1% failure rate) method of contraception (such as combination oral contraceptives, implanted contraceptives, or intrauterine devices) or effective method of contraception (such as diaphragms with spermicide or cervical sponges) for the duration of the study and until their plasma concentrations are below the level that could result in a relevant potential exposure to a possible fetus, predicted to be 90 days plus 5 half-lives following the last dose of study drug, which is approximately 4 months after the last injection.

a) Men and their partners may choose to use a double-barrier method of contraception. (Barrier protection methods without concomitant use of a spermicide are not an effective or acceptable method of contraception. Thus, each barrier method must include use of a spermicide. It should be noted, however, that the use of male and female condoms as a double-barrier method is not considered acceptable due to the high failure rate when these barrier methods are combined.)

b) Periodic abstinence (for example, calendar, ovulation, symptothermal, or postovulation methods), declaration of abstinence just for the duration of a trial, and withdrawal are not acceptable methods of contraception.

Men with pregnant partners should use condoms during intercourse for the duration of the study and until the end of the estimated, relevant potential exposure in WOCBP (4 months).

Men should refrain from sperm donation for the duration of the study and until their plasma concentrations are below the level that could result in a relevant potential exposure to a possible fetus, predicted to be 90 days plus 5 half-lives following the last dose of study drug, which is approximately 4 months.

Men who are in exclusively same-sex relationships (as their preferred and usual lifestyle) are not required to use contraception.

10.4.2. Female participants:

Women of childbearing potential who are abstinent (if this is complete abstinence, as their preferred and usual lifestyle) or in a same-sex relationship (as part of their preferred and usual lifestyle) must agree to either remain abstinent or stay in a same-sex relationship without sexual relationships with males. Periodic abstinence (for example, calendar, ovulation, symptothermal, or postovulation methods), declaration of abstinence just for the duration of a trial, and withdrawal are not acceptable methods of contraception.

Otherwise, WOCBP participating must agree to use 2 forms of effective contraception, where at least 1 form is highly effective (<than 1% failure rate), for the entirety of the study. Contraception must continue following completion of study drug administration for the entirety of the study and for 2 months after the last injection.

a) WOCBP participating must test negative for pregnancy prior to initiation of treatment as indicated by a negative serum pregnancy test at the screening visit followed by a negative urine pregnancy test within 24 hours prior to exposure.

b) Two forms of effective contraception, where at least 1 form is highly effective (such as combination oral contraceptives, implanted contraceptives, or intrauterine devices) will be used for the duration of the trial and for 2 months after the last injection. Effective contraception (such as male or female condoms with spermicide, diaphragms with spermicide, or cervical sponges) may be used as the second therapy. Barrier protection methods without concomitant use of a spermicide are not a reliable or acceptable method. Thus, each barrier method must include use of a spermicide (that is, condom with spermicide, diaphragm with spermicide, or female condom with spermicide). It should be noted that the use of male and female condoms as a double-barrier method is not considered acceptable due to the high failure rate when these methods are combined.

c) Not be breastfeeding.

Women not of childbearing potential may participate and include those who are:

a) Infertile due to surgical sterilization, or

b) Postmenopausal.

Definitions:

Woman of Childbearing Potential (WOCBP)

A woman is considered fertile following menarche and until becoming postmenopausal unless permanently sterile (see below).

If fertility is unclear (for example, amenorrhea in adolescents or athletes) and a menstrual cycle cannot be confirmed before first dose of study drug, additional evaluation should be considered.

Women in the following categories are not considered WOCBP

1. Premenarchal
2. Premenopausal female with 1 of the following:
   - Documented hysterectomy
   - Documented bilateral salpingectomy
   - Documented bilateral oophorectomy

For individuals with permanent infertility due to an alternate medical cause other than the above, (for example, mullerian agenesis, androgen insensitivity), investigator discretion should be applied to determining study entry.

Note: Determination can come from the site personnel's review of the participant's medical records, medical examination, or medical history interview.

3. Postmenopausal female is defined as follows:
   a. A woman at any age at least 6 weeks post-surgical bilateral oophorectomy with or without hysterectomy, confirmed by operative note
   b. A woman at least 40 years of age and up to 55 years old with an intact uterus, not on hormone therapy*, who has had cessation of menses for at least 12 consecutive

months without an alternative medical cause, AND with a follicle-stimulating hormone >40 mIU/mL; or

c. A woman 55 or older not on hormone therapy, who has had at least 12 months of spontaneous amenorrhea, or
d. A woman at least 55 years of age with a diagnosis of menopause prior to starting hormone replacement therapy

* Women should not be taking medications during amenorrhea such as oral contraceptives, hormones, gonadotropin-releasing hormone, anti-estrogens, selective estrogen receptor modulators (SERMs), or chemotherapy that could induce transient amenorrhea.

Contraception Guidance:

Highly Effective Methods of Contraception:

- Combined oral contraceptive pill and mini pill
- NuvaRing®
- Implantable contraceptives
- Injectable contraceptives (such as Depo-Provera®)
- Intrauterine device (such as Mirena® and ParaGard®)
- Contraceptive patch – ONLY women <198 pounds or 90 kg
- Total abstinence (if this is their preferred and usual lifestyle) or in a same-sex relationship with no sexual relationship with males (as part of their preferred and usual lifestyle).

Note: periodic abstinence (for example, calendar, ovulation, symptothermal, postovulation methods), declaration of abstinence just for the duration of a trial, and withdrawal are not acceptable methods of contraception.

Note: Implantable contraceptives and injectable contraceptives (such as Depo-Provera) are only permitted if started prior to screening. Participants should not start these methods of contraception after being enrolled in the study.

- Vasectomy - for men in clinical trials

Effective Methods of Contraception (must use combination of 2 methods):

- Male condom with spermicide
- Female condom with spermicide
- Diaphragm with spermicide
- Cervical sponge
- Cervical cap with spermicide

Collection of Pregnancy Information

Male participants with partners who become pregnant

- The investigator will attempt to collect pregnancy information on any male participant's female partner who becomes pregnant while the male participant is in this study.
- After obtaining the necessary signed informed consent from the pregnant female partner directly, the investigator will record pregnancy information on the appropriate form and

submit it to the sponsor within 24 hours of learning of the partner's pregnancy. The female partner will also be followed to determine the outcome of the pregnancy. Information on the status of the mother and child will be forwarded to the sponsor. Generally, the follow-up will be no longer than 6 to 8 weeks following the estimated delivery date. Any termination of the pregnancy will be reported regardless of fetal status (presence or absence of anomalies) or indication for the procedure.

Female Participants who become pregnant

- The investigator will collect pregnancy information on any female participant who becomes pregnant while participating in this study. The initial information will be recorded on the appropriate form and submitted to the sponsor within 24 hours of learning of a participant's pregnancy.
- The participant will be followed to determine the outcome of the pregnancy. The investigator will collect follow-up information on the participant and the neonate, and the information will be forwarded to the sponsor. Generally, follow-up will not be required for longer than 6 to 8 weeks beyond the estimated delivery date. Any termination of pregnancy will be reported, regardless of fetal status (presence or absence of anomalies) or indication for the procedure.
- While pregnancy itself is not considered to be an AE or SAE, any pregnancy complication or elective termination of a pregnancy for medical reasons will be reported as an AE or SAE.
- A spontaneous abortion (occurring at <20 weeks gestational age) or still birth (occurring at >20 weeks gestational age) is always considered to be an SAE and will be reported as such.
- Any poststudy, pregnancy-related SAE considered reasonably related to the study drug by the investigator will be reported to the sponsor as described in protocol Section 8.3.1. While the investigator is not obligated to actively seek this information in former study participants, he or she may learn of an SAE through spontaneous reporting.
- Any female participant who becomes pregnant while participating in the study will discontinue study drug and be withdrawn from the study.

10.5. Appendix 5: Adverse Events of Special Interest: Definitions and Procedures for Recording, Evaluating, Follow-Up, and Reporting.

10.5.1. Special Safety Topics

10.5.1.1. Hypoglycemia

Upon ICF signing, all participants will be educated about signs and symptoms of hypoglycemia, how to treat hypoglycemia, and how to collect appropriate information for each episode of hypoglycemia.

Hypoglycemia may be identified by spontaneous reporting of symptoms from participants (whether confirmed or unconfirmed by simultaneous glucose values) or by BG samples collected during study visits.

All participants with T2DM and who develop diabetes during the study will be provided with glucometers.

Participants with T2DM will be provided a diary to record relevant information (for example, glucose values, symptoms).

All hypoglycemic episodes are to be recorded on a specific eCRF and should not be otherwise recorded as AEs unless the event meets severe criteria. If a hypoglycemic event meets severe criteria (see definition below), it should be recorded as serious on the AE eCRFs, and reported to the sponsor as an SAE. To avoid duplicate reporting, all consecutive BG values <70 mg/dL (<3.9 mmol/L) occurring within a 1-hour period may be considered to be a single hypoglycemic event (Weinberg et al. 2010; Danne et al. 2013).

Investigators should use the following definitions and criteria when diagnosing and categorizing an episode considered to be related to hypoglycemia (the BG values in this section refer to values determined by a laboratory or International Federation of Clinical Chemistry and Laboratory Medicine blood-equivalent glucose meters and strips) in accordance with the 2020 American Diabetes Association position statement on glycemic targets (ADA 2020):

Glucose Alert Value (Level 1):

- Documented symptomatic hypoglycemia is defined as any time a participant feels that he or she is experiencing symptoms and/or signs associated with hypoglycemia and has a BG level of <70 mg/dL (<3.9 mmol/L).
- Documented asymptomatic hypoglycemia is defined as any event not accompanied by typical symptoms of hypoglycemia, but with a measured BG <70 mg/dL (<3.9 mmol/L).
- Documented unspecified hypoglycemia is defined as any event with no information about symptoms of hypoglycemia available, but with a measured BG <70 mg/dL (<3.9 mmol/L).

Clinically Significant Hypoglycemia (Level 2):

- Documented symptomatic hypoglycemia is defined as any time a participant feels that he or she is experiencing symptoms and/or signs associated with hypoglycemia and has a BG level of <54 mg/dL (<3.0 mmol/L).

- Documented asymptomatic hypoglycemia is defined as any event not accompanied by typical symptoms of hypoglycemia but with a measured BG <54 mg/dL (<3.0 mmol/L).
- Documented unspecified hypoglycemia is defined as any event with no information about symptoms of hypoglycemia available but with a measured BG <54 mg/dL (<3.0 mmol/L).

Severe Hypoglycemia (Level 3):

- Severe hypoglycemia is defined as an episode with severe cognitive impairment requiring the assistance of another person to actively administer carbohydrate, glucagon, or other resuscitative actions. These episodes may be associated with sufficient neuroglycopenia to induce seizure or coma. Blood glucose measurements may not be available during such an event, but neurological recovery attributable to the restoration of BG to normal is considered sufficient evidence that the event was induced by a low BG concentration.

Nocturnal Hypoglycemia:

Nocturnal hypoglycemia is a hypoglycemia event (including severe hypoglycemia) that occurs at night, presumably during sleep.

10.5.1.2. Pancreatitis

Acute pancreatitis is defined as an AE of interest in all trials with tirzepatide, including this trial.

Acute pancreatitis is an acute inflammatory process of the pancreas that may also involve peripancreatic tissues and/or remote organ systems (Banks and Freeman 2006). The diagnosis of acute pancreatitis requires 2 of the following 3 features:

- abdominal pain, characteristic of acute pancreatitis (generally located in the epigastrium and radiates to the back in approximately half the cases) (Banks and Freeman 2006; Koizumi et al. 2006); the pain is often associated with nausea and vomiting
- serum amylase (total and/or pancreatic) and/or lipase ≥3X ULN, and
- characteristic findings of acute pancreatitis on CT scan or MRI.

If acute pancreatitis is suspected, appropriate laboratory tests (including levels of pancreatic amylase and lipase) should be obtained via the central laboratory (and locally, if needed).

Imaging studies, such as abdominal CT scan with or without contrast, MRI, or gallbladder ultrasound, should be performed. Abdominal ultrasound may be used as an alternative method only if CT and MRI cannot be performed. If laboratory values and/or abdominal imaging support the diagnosis of acute pancreatitis, the participant must discontinue therapy with tirzepatide but will continue in the study. A review of the participant's concomitant medications should be conducted to assess any potential causal relationship with pancreatitis.

Each AE of pancreatitis must be reported. If typical signs and/or symptoms of pancreatitis are present and confirmed by laboratory values (lipase or amylase [total and/or pancreatic]) and imaging studies, the event must be reported as an SAE. For a potential case that does not meet all of these criteria, it is up to the investigator to determine the seriousness of the case (AE or SAE) and the relatedness of the event to study drug(s).

Each participant will have measurements of p-amylase and lipase (assessed at the central laboratory) as shown on the SoA (Section 1.3) to assess the effects of the investigational doses of tirzepatide on pancreatic enzyme levels. Serial measurements of pancreatic enzymes have limited clinical value for predicting episodes of acute pancreatitis in asymptomatic participants (Nauck et al. 2017; Steinberg et al. 2017a, 2017b). Thus, further diagnostic follow-up of cases of asymptomatic pancreatic hyperenzymemia (lipase and/or pancreatic amylase ≥3X ULN) is not mandated but may be performed based on the investigator's clinical judgment and assessment of the participant's overall clinical condition. Only cases of pancreatic hyperenzymemia that undergo additional diagnostic follow-up and/or are accompanied by symptoms suggestive of pancreatitis will be submitted for adjudication.

All suspected cases of acute or chronic pancreatitis will be adjudicated by an independent clinical endpoint committee. In addition, AEs of severe or serious abdominal pain of unknown etiology will also be submitted to the adjudication committee to assess for possible pancreatitis or other pancreatic disease. Relevant data from participants with acute or chronic pancreatitis and those with severe or serious abdominal pain will be entered into a specifically designed eCRF page. The adjudication committee representative will enter the results of adjudication in a corresponding eCRF page.

10.5.1.3. Thyroid Malignancies and C-Cell Hyperplasia

Individuals with personal or family history of MTC and/or MEN-2 will be excluded from the study. Participants who are diagnosed with MTC and/or MEN-2 during the study will have study drug stopped and should continue follow-up with an endocrinologist.

The assessment of thyroid safety during the trial will include reporting of any case of thyroid malignancy (including MTC and papillary carcinoma) and measurements of calcitonin. This data will be captured in specific eCRFs. The purpose of calcitonin measurements is to assess the potential of tirzepatide to affect thyroid C-cell function, which may indicate development of C-cell hyperplasia and neoplasms.

10.5.1.4. Calcitonin Measurements

If an increased calcitonin value (see definitions below) is observed in a participant who has been administered a medication that is known to increase serum calcitonin, then this medication should be stopped, and calcitonin levels should be measured after an appropriate washout period.

For participants who require additional endocrine assessment because of increased calcitonin concentration as defined in this section, data from the follow-up assessment will be collected in the specific section of the eCRF.

Calcitonin Measurements in Participants with eGFR ≥60 mL/min/1.73m^2

A significant increase in calcitonin for participants with eGFR ≥60 mL/min is defined below. If a participant's laboratory results meet these criteria, these clinically significant laboratory results should be recorded as an AE.

- calcitonin value ≥20 ng/L and <35 ng/L AND ≥50% increase from the screening value.
  - These participants will be asked to repeat the measurement within 1 month. If this repeat value is increasing (≥10% increase), study drug should be stopped, and the

participant encouraged to undergo additional endocrine assessment and longer-term follow-up by an endocrinologist to exclude any serious adverse effect on the thyroid.

- calcitonin value ≥35 ng/L AND ≥50% over the screening value.
    - In these participants, study drug should be stopped, and the participant recommended to immediately undergo additional endocrine assessments and longer-term follow-up by an endocrinologist.

Calcitonin Measurement in Participants with eGFR <60 mL/min/1.73 m^2

A significant increase in calcitonin for participants with eGFR <60 mL/min/1.73m^2 is defined as a calcitonin value ≥35 ng/L AND ≥50% over the screening value. If a participant's labs meet these criteria, these clinically significant labs should be recorded as an AE.

In these participants, study drug should be discontinued (after first confirming the value), and the participant recommended to immediately undergo additional endocrine assessments and longer-term follow-up by an endocrinologist to exclude any serious adverse effect on the thyroid.

10.5.1.5. Major Adverse Cardiovascular Events

Deaths and nonfatal CV AEs will be adjudicated by a committee of physicians external to the sponsor with cardiology expertise. This committee will be blinded to treatment assignment. The nonfatal CV AEs to be adjudicated include:

- myocardial infarction
- hospitalization for unstable angina
- coronary interventions (such as coronary artery bypass graft or percutaneous coronary intervention), and
- cerebrovascular events, including cerebrovascular accident (stroke) and transient ischemic attack.

10.5.1.6. Supraventricular Arrhythmias and Cardiac Conduction Disorders

Participants who develop any event from these groups of disorders should undergo an ECG, which will be retained at the site as a source document. Additional diagnostic tests to determine exact diagnosis should be performed, as needed. The specific diagnosis will be recorded as an AE. Events that meet criteria for serious conditions as described in Section 10.3.2 must be reported as SAEs. If a clinically significant finding is identified by ECG (including, but not limited to, AF or changes from baseline in corrected QT interval), the investigator or qualified designee will determine if any change in study participant management is needed. This review of the ECG printed at the time of collection must be documented. Any new clinically relevant finding should be reported as an AE.

10.5.1.7. Hypersensitivity Events

All allergic or hypersensitivity reactions will be reported by the investigator as either AEs, or if any serious criterion is met, as SAEs.

In the event of suspected drug hypersensitivity reactions (immediate or nonimmediate) in subjects who experience moderate-to-severe reactions as assessed by the investigator, unscheduled blood samples will be collected as outlined in Appendix 7 (Section 10.7).

Additional data, such as type of reaction and treatment received, will be collected on any AEs or SAEs that the investigator deems related to study drug via the eCRF created for this purpose.

Study drug should be temporarily interrupted in any individual suspected of having a severe or serious allergic reaction to study drug. Study drug may be restarted when/if it is safe to do so, in the opinion of the investigator.

10.5.1.8. Injection Site Reactions

Injection site reactions will be collected on the eCRF separate from the hypersensitivity reaction eCRF. At the time of AE occurrence, samples will be collected for measurement of tirzepatide ADA and tirzepatide concentration.

10.5.1.9. Antidrug Antibodies

The occurrence of ADA formation will be assessed as outlined in Section 8.9.

10.5.1.10. Hepatobiliary Disorders

All events of treatment-emergent biliary colic, cholecystitis, or other suspected events related to gallbladder disease should be evaluated and additional diagnostic tests performed, as needed. In cases of elevated liver markers, hepatic monitoring should be initiated as outlined in Appendix 8 (Section 10.8).

10.5.1.11. Severe Gastrointestinal Adverse Events

Tirzepatide may cause severe GI AEs such as nausea, vomiting, and diarrhea. Information about severe GI AEs as well as antiemetic/antidiarrheal use will be collected in the eCRF/AE form. For detailed information concerning the management of GI AEs, please refer to Section 6.5.

10.5.1.12. Acute Renal Events

Renal safety will be assessed based on repeated renal function assessment as well as assessment of AEs suggestive of acute or worsening of chronic renal failure. Gastrointestinal AEs have been reported with tirzepatide, including nausea, diarrhea, and vomiting. This is consistent with other GLP-1 RAs (Aroda and Ratner 2011). The events may lead to dehydration, which could cause a deterioration in renal function, including acute renal failure.

Participants should be advised to notify investigators in case of severe nausea, frequent vomiting, or symptoms of dehydration.

10.6. Appendix 6: Genetics

Use/Analysis of DNA

- Genetic variation may impact a participant's response to study drug, susceptibility to, severity, and progression of disease. Variable response to study drug may be due to genetic determinants that impact drug absorption, distribution, metabolism, and excretion; mechanism of action of the drug; disease etiology; and/or molecular subtype of the disease being treated. Therefore, where local regulations and IRB/IEC allow, a blood sample will be collected for DNA analysis from consenting participants.
- DNA samples will be used for research related to tirzepatide or HF and related diseases. They may also be used to develop tests/assays including diagnostic tests related to tirzepatide or HF. Genetic research may consist of the analysis of one or more candidate genes, the analysis of genetic markers throughout the genome, or analysis of the entire genome (as appropriate).
- DNA sample analysis may be performed on pharmacogenetic variants thought to play a role in T2DM or CV disease to evaluate their association with observed clinical outcomes to tirzepatide in this study. In the event the observation of a study drug response, the samples may be genotyped, and analysis may be performed to evaluate a genetic association with response to tirzepatide. These investigations may be limited to a focused, candidate-gene study or, if appropriate, genome-wide association studies may be performed to identify regions of the genome associated with the variability observed in drug response. Samples may be used for investigations related to the disease, drug, or class of drugs under study in the context of this clinical program; however, samples may not be used for broad, exploratory, unspecified disease or population genetic analysis. Additional analyses may be conducted if it is hypothesized that this may help further understand the clinical data.
- The samples may be analyzed as part of a multi-study assessment of genetic factors involved in the response to tirzepatide or study drugs of this class to understand the study disease or related conditions.
- The results of genetic analyses may be reported in the CSR or in a separate study summary.
- The sponsor will store the DNA samples in a secure storage space with adequate measures to protect confidentiality.
- The samples will be retained while research on tirzepatide continues but no longer than 15 years or another period as per local requirements (see Section 10.1.10).

10.7. Appendix 7: Recommended Laboratory Testing for Hypersensitivity Events

Laboratory testing should be performed at the time of a systemic hypersensitivity event. The management of the AE may warrant lab testing beyond that described below and should be performed as clinically indicated. Laboratory assessments should be performed if the participant experiences generalized urticaria or if anaphylaxis is suspected.

- Collect the sample after the participant has been stabilized and within 1 to 2 hours of the event; however, samples may be obtained as late as 12 hours after the event as analytes can remain altered for an extended period of time. Record the time at which the sample was collected.
- Obtain a follow-up sample at the next regularly scheduled visit or after approximately 4 weeks, whichever is later.

Clinical Laboratory Tests for Hypersensitivity Events

**Hypersensitivity Tests**	**Notes**<br>Selected test may be obtained in the event of anaphylaxis or systemic allergic/hypersensitivity reactions.
Tirzepatide antidrug antibodies (immunogenicity/ADA)	Assayed by Lilly-designated laboratory.<br>Results will not be provided to the investigative sites.
Tirzepatide concentrations (PK)	Assayed by Lilly-designated laboratory.<br>Results will not be provided to the investigative sites.
Tryptase	Assayed by Lilly-designated laboratory.<br>Results will not be provided to the investigative sites.<br>Urine N-methylhistamine testing is performed in addition to tryptase testing. Collect the first void urine sample following the event. Collect a follow-up urine sample after approximately 4 weeks.<br>**Note:** If a tryptase sample is obtained more than 2 hours after the event (that is, within 2-12 hours), or is not obtained because more than 12 hours have lapsed since the event, collect a urine sample for N-methylhistamine testing.
N-methylhistamine	Will be performed if validated assay is available.<br>Assayed by Lilly-designated laboratory.<br>Results will not be provided to the investigative sites.
Drug-specific IgE	Will be performed if a validated assay is available.<br>Assayed by Lilly-designated laboratory.<br>Results will not be provided to the investigative sites.
Basophil activation test	Will be performed if a validated assay is available.<br>Assayed by Lilly-designated laboratory.<br>Results will not be provided to the investigative sites.<br>**NOTE:** The basophil activation test is an in vitro, cell-based assay that only requires a serum sample. It is a surrogate assay for drug specific-IgE but is not specific for IgE.
Complement (C3, C3a and C5a)	Assayed by Lilly-designated laboratory.<br>Results will not be provided to the investigative sites.
Cytokine panel: IL-6, IL-1β, IL-10	Assayed by Lilly-designated laboratory.<br>Results will not be provided to the investigative sites.

Abbreviations: ADA = antidrug antibody; IgE = immunoglobulin E; IL = interleukin; PK = pharmacokinetic.

10.8. Appendix 8: Liver Safety: Suggested Actions and Follow-Up Assessments

Hepatic Evaluation Testing

See Section 8.2.5.1 for guidance on appropriate test selection.

The Lilly-designated central laboratory must complete the analysis of all selected testing except for microbiology testing.

Local testing may be performed in addition to central testing when necessary for immediate participant management.

Results will be reported if a validated test or calculation is available.

**Hematology**	**Clinical Chemistry**
Hemoglobin	Total bilirubin
Hematocrit	Direct bilirubin
Erythrocytes (red blood cells [RBCs])	Alkaline phosphatase (ALP)
Leukocytes (white blood cells [WBCs])	Alanine aminotransferase (ALT)
Differential:	Aspartate aminotransferase (AST)
Neutrophils, segmented	Gamma-glutamyl transferase (GGT)
Lymphocytes	Creatine kinase (CK)
Monocytes	**Other Chemistry**
Basophils	Acetaminophen
Eosinophils	Acetaminophen protein adducts
Platelets	Alkaline phosphatase isoenzymes
Cell morphology (RBC and WBC)	Ceruloplasmin
**Coagulation**	Copper
	Ethyl alcohol (EtOH)
Prothrombin time, INR (PT-INR)	Haptoglobin
**Serology**	Immunoglobulin IgA (quantitative)
Hepatitis A virus (HAV) testing:	Immunoglobulin IgG (quantitative)
HAV total antibody	Immunoglobulin IgM (quantitative)
HAV IgM antibody	Phosphatidylethanol (PEth)
Hepatis B virus (HBV) testing:	**Urine Chemistry**
Hepatitis B surface antigen (HBsAg)	Drug screen
Hepatitis B surface antibody (anti-HBs)	Ethyl glucuronide (EtG)
Hepatitis B core total antibody (anti-HBc)	**Other Serology**
Hepatitis B core IgM antibody	Antinuclear antibody (ANA)
Hepatitis B core IgG antibody	Anti-smooth muscle antibody (ASMA)[a]

HBV DNA[b]	Anti-actin antibody[c]
Hepatis C virus (HCV) testing:	Epstein-Barr virus (EBV) testing:
HCV antibody	EBV antibody
HCV RNA[b]	EBV DNA[b]
Hepatitis D virus (HDV) testing:	Cytomegalovirus (CMV) testing:
HDV antibody	CMV antibody
Hepatitis E virus (HEV) testing:	CMV DNA[b]
HEV IgG antibody	Herpes simplex virus (HSV) testing:
HEV IgM antibody	HSV (Type 1 and 2) antibody
HEV RNA[b]	HSV (Type 1 and 2) DNA[b]
**Microbiology**[d]	Liver kidney microsomal type 1 (LKM-1) antibody
Culture:	
Blood	
Urine	

Abbreviations: Ig = immunoglobulin; INR = international normalized ratio; PT = prothrombin time.

[a] Not required if anti-actin antibody is tested.

[b] Reflex/confirmation dependent on regulatory requirements, testing availability, or both.

[c] Not required if anti-smooth muscle antibody (ASMA) is tested.

[d] Assayed ONLY by investigator-designated local laboratory; no central testing available.

10.9. Appendix 9: Medical Device Adverse Events (AEs), Adverse Device Effects (ADEs), Serious Adverse Events (SAEs) and Device Deficiencies: Definition and Procedures for Recording, Evaluating, Follow-Up, and Reporting

Refer to Section 10.3 for definitions and procedures for recording, evaluating, follow-up, and reporting of all events.

10.10. Appendix 10: Six-Minute Walk Test Screening Procedures

10.10.1. Screening Procedures and Flow Diagram

The flow diagram below details the participant flow and eligibility with the 6MWT.

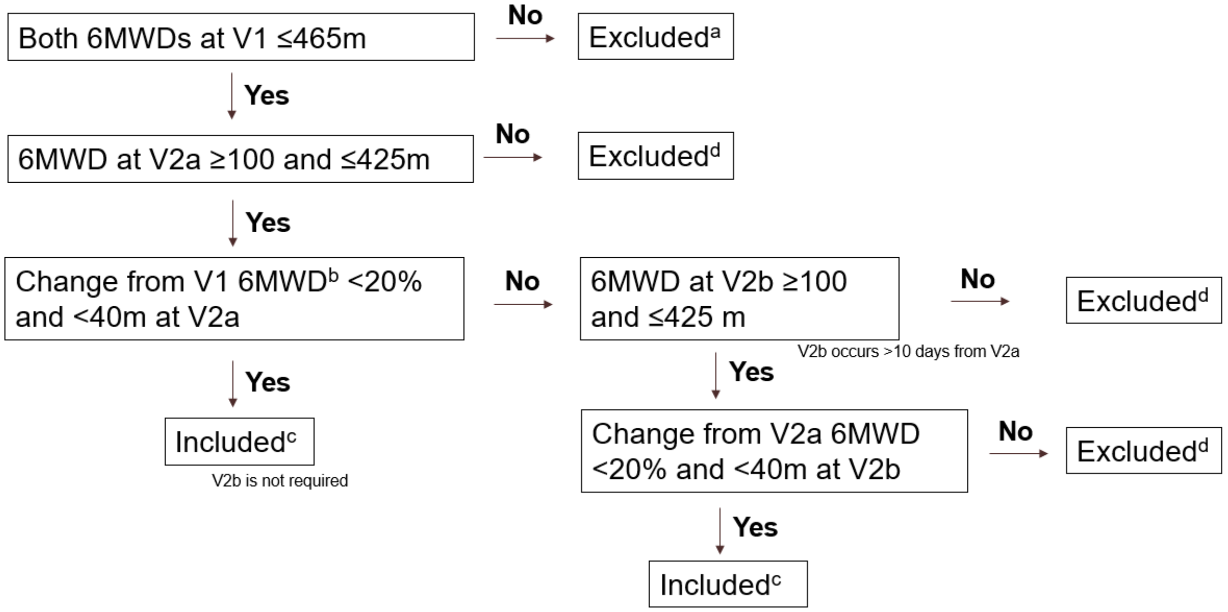

Abbreviations: 6MWD = 6-minute walk test distance; 6MWT = 6-minute walk test; V1 = Visit 1; V2a = Visit 2a; V2b = Visit 2b.

[a] Rescreening is not allowed

[b] Use the higher value of the two Visit 1 6MWD as a reference for Visit 2a

[c] Continue with other Visit 2 assessments according to the SoA

[d] Participants excluded on 6MWT may be re-screened after a minimum of 2 weeks

10.11. Appendix 11: New York Heart Association Classification of Heart Failure

Class	Symptomatology
**I**	No symptoms. Ordinary physical activity such as walking and climbing stairs does not cause fatigue or dyspnea.
**II**	Symptoms with ordinary physical activity. Walking or climbing stairs rapidly, walking uphill, walking or stair climbing after meals, in cold weather, in wind or when under emotional stress causes undue fatigue or dyspnea.
**III**	Symptoms with less than ordinary physical activity. Walking 1-2 blocks on the level and climbing more than one flight of stairs in normal conditions causes undue fatigue or dyspnea.
**IV**	Symptoms at rest. Inability to carry on any physical activity without fatigue or dyspnea.

10.12. Appendix 12: Abbreviations

Term	Definition
**6MWD**	6-minute walk test distance
**6MWT**	6-minute walk test
**ADA**	antidrug antibody
**AE**	adverse event
**AESI**	adverse events of special interest
**AF**	atrial fibrillation
**ALP**	alkaline phosphatase
**ALT**	alanine aminotransferase
**AST**	aspartate aminotransferase
**blinding/masking**	A single-blind study is one in which the investigator and/or his staff are aware of the treatment but the participant is not, or vice versa, or when the sponsor is aware of the treatment but the investigator and/his staff and the participant are not.<br>A double-blind study is one in which neither the participant nor any of the investigator or sponsor staff who are involved in the treatment or clinical evaluation of the subjects are aware of the treatment received.
**BG**	blood glucose
**BMI**	body mass index
**CEC**	clinical endpoint committee
**CFR**	Code of Federal Regulations
**CIOMS**	Council for International Organizations of Medical Sciences
**complaint**	A complaint is any written, electronic, or oral communication that alleges deficiencies related to the identity, quality, purity, durability, reliability, safety or effectiveness, or performance of a drug or drug delivery system.
**compliance**	Adherence to all study-related, good clinical practice (GCP), and applicable regulatory requirements.
**CONSORT**	Consolidated Standards of Reporting Trials
**CRF**	case report form
**CRP**	clinical research physician: Individual responsible for the medical conduct of the study. Responsibilities of the CRP may be performed by a physician, clinical research scientist, global safety physician or other medical officer.

Term	Definition
**CSR**	clinical study report
**CT**	computed tomography
**CV**	cardiovascular
**D Bil**	direct bilirubin
**DMC**	data monitoring committee
**Device Deficiencies**	Equivalent to product complaint
**ECG**	electrocardiogram
**eCOA**	electronic clinical outcome assessment
**eCRF**	electronic case report form
**EDC**	electronic data capture
**eGFR**	estimated glomerular filtration rate
**enroll**	The act of assigning a participant to a treatment. Participants who are enrolled in the study are those who have been assigned to a treatment.
**enter**	Participants entered into a study are those who sign the informed consent form directly or through their legally acceptable representatives.
**ERB**	ethical review board
**ET**	early termination
**EV**	extended visit
**GCP**	good clinical practice
**GGT**	gamma-glutamyl transferase
**GI**	gastrointestinal
**GIP**	glucose-dependent insulinotropic polypeptide
**GLP-1**	glucagon-like peptide-1
**HbA1c**	hemoglobin A1c
**HF**	heart failure
**HFpEF**	heart failure with preserved ejection fraction
**HIPAA**	Health Insurance Portability and Accountability Act

Term	Definition
**IB**	Investigator's Brochure
**ICF**	informed consent form
**ICH**	International Council for Harmonisation
**IEC**	independent ethics committee
**informed consent**	A process by which a participant voluntarily confirms his or her willingness to participate in a particular study, after having been informed of all aspects of the study that are relevant to the participant's decision to participate. Informed consent is documented by means of a written, signed and dated informed consent form.
**INR**	international normalized ratio
**interim analysis**	An interim analysis is an analysis of clinical study data, separated into treatment groups, that is conducted before the final reporting database is created/locked.
**IRB**	institutional review board
**ISO**	International Organization for Standardization
**ITT**	intention to treat: The principle that asserts that the effect of a treatment policy can be best assessed by evaluating on the basis of the intention to treat a participant (that is, the planned treatment regimen) rather than the actual treatment given. It has the consequence that participant allocated to a treatment group should be followed up, assessed, and analyzed as members of that group irrespective of their compliance to the planned course of treatment.
**IV**	intravenous
**IWRS**	interactive web-response system
**KCCQ**	Kansas City Cardiomyopathy Questionnaire
**KCCQ-CSS**	Kansas City Cardiomyopathy Questionnaire – Clinical Summary Scale
**LA**	left atrial
**LAV**	left atrial volume
**LVDEP**	left ventricular end-diastolic pressure
**LVEF**	left ventricular ejection fraction
**MedDRA**	Medical Dictionary for Regulatory Activities
**MEN-2**	multiple endocrine neoplasia type 2
**MMRM**	mixed model repeated measures
**MRI**	magnetic resonance imaging

Term	Definition
**MTC**	medullary thyroid cancer
**MTD**	maximum tolerated dose
**NT-proBNP**	N-terminal pro b-type natriuretic peptide
**NYHA**	New York Heart Association
**participant**	Equivalent to CDISC term “subject”: an individual who participates in a clinical trial, either as recipient of an investigational medicinal product or as a control
**PC**	product complaint
**PCWP**	pulmonary capillary wedge pressure
**PK**	pharmacokinetics
**PT**	prothrombin time
**QTc**	corrected QT interval
**QW**	weekly
**RA**	receptor agonist
**SADE**	serious adverse device effect
**SAE**	serious adverse event
**SAP**	statistical analysis plan
**SBP**	systolic blood pressure
**SC**	subcutaneous
**screen**	The act of determining if an individual meets minimum requirements to become part of a pool of potential candidates for participation in a clinical study.
**SoA**	schedule of activities
**study intervention**	for this study, study intervention may be interpreted/synonymous with study drug
**SUSAR**	suspected unexpected serious adverse reaction
**T1DM**	type 1 diabetes mellitus
**T2DM**	type 2 diabetes mellitus
**TBL**	total bilirubin

Term	Definition
**TEAE**	Treatment-emergent adverse event: An untoward medical occurrence that emerges during a defined treatment period, having been absent pretreatment, or worsens relative to the pretreatment state, and does not necessarily have to have a causal relationship with this treatment.
**UADE**	unanticipated adverse device effect
**ULN**	upper limit of normal
**WOCBP**	women of childbearing potential

11. References

[ADA] American Diabetes Association. Glycemic targets: standards of medical care in diabetes – 2020. *Diabetes Care*. 2020;43(suppl 1):S66-S76. https://doi.org/10.2337/dc20-S006

Aminian A, Zajichek A, Arterburn DE, et al. Association of metabolic surgery with major adverse cardiovascular outcomes in patients with type 2 diabetes and obesity. *JAMA*. 2019;322(13):1271-1282. https://doi.org/10.1001/jama.2019.14231

Aroda VR, Ratner R. The safety and tolerability of GLP-1 receptor agonists in the treatment of type 2 diabetes: a review. *Diabetes Metab Res Rev*. 2011;27(6):528-542. https://doi.org/10.1002/dmrr.1202

Banks PA, Freeman ML.; Practice Parameters Committee of the American College of Gastroenterology. Practice guidelines in acute pancreatitis. *Am J Gastroenterol*. 2006;101(10):2379-2400. https://doi.org/10.1111/j.1572-0241.2006.00856.x

Danne T, Philotheou A, Goldman D, et al. A randomized trial comparing the rate of hypoglycemia – assessed using continuous glucose monitoring – in 125 preschool children with type 1 diabetes treated with insulin glargine or NPH insulin (the PRESCHOOL study). *Pediatr Diabetes*. 2013;14(8):593-601. https://doi.org/10.1111/pedi.12051

Davies MJ, Bergenstal R, Bode B, et al. Efficacy of liraglutide for weight loss among patients with type 2 diabetes: The SCALE Diabetes Randomized Clinical Trial. *JAMA*. 2015;314(7):687-699. https://doi.org/10.1001/jama.2015.9676 Erratum in: JAMA. 2016 Jan 5;315(1):90. PMID: 26284720.

Davies MJ, D'Alessio DA, Fradkin J, et al. Management of hyperglycemia in type 2 diabetes, 2018. A consensus report by the American Diabetes Association (ADA) and the European Association for the Study of Diabetes (EASD). *Diabetes Care*. 2018;41(12):2669-2701. https://doi.org/10.2337/dci18-0033

[EMA] European Medicines Agency. Guideline on clinical evaluation of medicinal products used in weight management. EMA/CHMP/311805/2014. Published June 23, 2016. Accessed December 23, 2020. https://www.ema.europa.eu/en/documents/scientific-guideline/guideline-clinical-evaluation-medicinal-products-used-weight-management-revision-1_en.pdf

EuroQol Research Foundation. EQ-5D-5L User Guide, Version 3.0. Updated September 2019. Accessed March 13, 2020. https://euroqol.org/publications/user-guides

[FDA] United States Food and Drug Administration. Guidance for industry: developing products for weight management. Published February 2007. Accessed December 23, 2020. https://www.fda.gov/media/71252/download

Ferreira JP, Jhund PS, Duarte K, et al. Use of the win ratio in cardiovascular trials. *JACC Heart Fail*. 2020;8(6):441-450. https://doi.org/10.1016/j.jchf.2020.02.010

Finkelstein DM, Schoenfeld DA. Combining mortality and longitudinal measures in clinical trials. *Stat Med*. 1999;18(11):1341-1354. https://doi.org/10.1002/(sici)1097-0258(19990615)18:11<1341::aid-sim129>3.0.co;2-7

Frias JP, Nauck MA, Van J, et al. Efficacy and safety of LY3298176, a novel dual GIP and GLP-1 receptor agonist, in patients with type 2 diabetes: a randomised, placebo-controlled and active comparator-controlled phase 2 trial. *Lancet*. 2018;392(10160):2180-2193. https://doi.org/10.1016/s0140-6736(18)32260-8

Green CP, Porter CB, Bresnahan DR, Spertus JA. Development and evaluation of the Kansas City Cardiomyopathy Questionnaire: a new health status measure for heart failure. *J Am Coll Cardiol*. 2000;35(5):1245-1255. https://doi.org/10.1016/S0735-1097(00)00531-3

Herring LY, Stevinson C, Davies MJ, et al. Changes in physical activity behaviour and physical function after bariatric surgery: a systematic review and meta-analysis. *Obes Rev*. 2016;17(3):250-261. https://doi.org/10.1111/obr.12361

Jamaly S, Carlsson L, Peltonen M, et al. Surgical obesity treatment and the risk of heart failure. *Eur Heart J*. 2019;40(26):2131-2138. https://doi.org/10.1093/eurheartj/ehz295

Joseph SM, Novak E, Arnold SV, et al. Comparable performance of the Kansas City Cardiomyopathy Questionnaire in patients with heart failure with preserved and reduced ejection fraction. *Circ Heart Fail*. 2013;6(6):1139-1146. https://doi.org/10.1161/CIRCHEARTFAILURE.113.000359

Kitzman DW, Brubaker P, Morgan T, et al. Effect of caloric restriction or aerobic exercise training on peak oxygen consumption and quality of life in obese older patients with heart failure with preserved ejection fraction: a randomized clinical trial. *JAMA*. 2016;315(1):36-46. https://doi.org/10.1001/jama.2015.17346

Kitzman DW, Shah SJ. The HFpEF obesity phenotype: the elephant in the room. *J Am Coll Cardiol*. 2016;68(2):200-203. https://doi.org/10.1016/j.jacc.2016.05.019

Koizumi M, Takada T, Kawarada Y, et al. JPN Guidelines for the management of acute pancreatitis: diagnostic criteria for acute pancreatitis. *J Hepatobiliary Pancreat Surg*. 2006;13(1):25-32. https://doi.org/10.1007/s00534-005-1048-2

Lingvay I, Desouza CV, Lalic KS, et al. A 26-week randomized controlled trial of semaglutide once daily versus liraglutide and placebo in patients with type 2 diabetes suboptimally controlled on diet and exercise with or without metformin. *Diabetes Care*. 2018;41(9):1926-1937. https://doi.org/10.2337/dc17-2381

Mikhalkova D, Holman SR, Jiang H, et al. Bariatric surgery-induced cardiac and lipidomic changes in obesity-related heart failure with preserved ejection fraction. *Obesity (Silver Spring)*. 2018;26(2):284-290. https://doi.org/10.1002/oby.22038

Miller WL, Borlaug BA. Impact of obesity on volume status in patients with ambulatory chronic heart failure. *J Card Fail*. 2020;26(2):112-117. https://doi.org/10.1016/j.cardfail.2019.09.010

Nauck MA, Frossard JL, Barkin JS, et al. Assessment of pancreas safety in the development program of once-weekly GLP-1 receptor agonist dulaglutide. *Diabetes Care*. 2017;40(5):647-654. https://doi.org/10.2337/dc16-0984

Packer M. Epicardial adipose tissue may mediate deleterious effects of obesity and inflammation on the myocardium. *J Am Coll Cardiol*. 2018;71(20):2360-2372. https://doi.org/10.1016/j.jacc.2018.03.509

Pi-Sunyer X, Astrup A, Fujioka K, et al; SCALE Obesity and Prediabetes NN8022-1839 Study Group. A randomized, controlled trial of 3.0 mg of liraglutide in weight management. *N Engl J Med*. 2015;373(1):11-22. https://doi.org/10.1056/NEJMoa1411892

Pocock SJ, Ariti CA, Collier TJ, Wang D. The win ratio: a new approach to the analysis of composite endpoints in clinical trials based on clinical priorities. *Eur Heart J*. 2012;33(2):176-182. https://doi.org/10.1093/eurheartj/ehr352

Rayner JJ, Peterzan MA, Watson WD, et al. Myocardial energetics in obesity: enhanced ATP delivery through creatine kinase with blunted stress response. *Circulation*. 2020;141(14):1152-1163. https://doi.org/10.1161/CIRCULATIONAHA.119.042770

Reddy YN, Rikhi A, Obokata M, et al. Quality of life in heart failure with preserved ejection fraction: importance of obesity, functional capacity, and physical inactivity. *Eur J Heart Fail*. 2020;22(6):1009-1018. https://doi.org/10.1002/ejhf.1788

Regev A, Palmer M, Avigan MI, et al. Consensus: guidelines: best practices for detection, assessment and management of suspected acute drug-induced liver injury during clinical trials in patients with nonalcoholic steatohepatitis. *Aliment Pharmacol Ther*. 2019;49(6):702-713.

Rubin DB. *Multiple Imputation for Nonresponse in Surveys*. John Wiley & Sons, Inc.; 1987.

Shimada YJ, Tsugawa Y, Brown DFM, Hasegawa K. Bariatric surgery and emergency department visits and hospitalizations for heart failure exacerbation: population-based, self-controlled series. *J Am Coll Cardiol*. 2016;67(8):895-903. https://doi.org/10.1016/j.jacc.2015.12.016

Shoemaker MJ, Curtis AB, Vangsnes E, Dickinson MG. Clinically meaningful change estimates for the six-minute walk test and daily activity in individuals with chronic heart failure. *Cardiopulm Phys Ther J*. 2013;24(3):21-29.

Solomon SD, Dobson J, Pocock S, et al.; Candesartan in Heart Failure: Assessment of Reduction in Mortality and Morbidity (CHARM) Investigators. *Circulation*. 2007;116(13):1482-1487. https://doi.org/10.1161/CIRCULATIONAHA.107.696906

Spertus JA, Jones PG, Sandhu AT, Arnold SV. Interpreting the Kansas City Cardiomyopathy Questionnaire in clinical trials and clinical care: JACC state-of-the-art review. *J Am Coll Cardiol*. 2020;76(20):2379-2390. https://doi.org/10.1016/j.jacc.2020.09.542

Steinberg WM, Buse JB, Ghorbani MLM, et al.; LEADER Steering Committee; LEADER Trial Investigators. Amylase, lipase, and acute pancreatitis in people with type 2 diabetes treated with liraglutide: results from the LEADER randomized trial. *Diabetes Care*. 2017a;40(7):966-972. https://doi.org/10.2337/dc16-2747

Steinberg WM, Rosenstock J, Wadden TA, et al. Impact of liraglutide on amylase, lipase, and acute pancreatitis in participants with overweight/obesity and normoglycemia, prediabetes, or type 2 diabetes: secondary analyses of pooled data from the SCALE clinical development program. *Diabetes Care*. 2017b;40(7):839-848. https://doi.org/10.2337/dc16-2684

Sundström J, Bruze G, Ottosson J, et al. Weight loss and heart failure: a nationwide study of gastric bypass surgery versus intensive lifestyle treatment. *Circulation*. 2017;135(17):1577-1585. https://doi.org/10.1161/CIRCULATIONAHA.116.025629

Täger T, Hanholz W, Cebola R, et al. Minimal important difference for 6-minute walk test distances among patients with chronic heart failure. *Int J Cardiol*. 2014;176(1):94-98. https://doi.org/10.1016/j.ijcard.2014.06.035

van der Meer P, Gaggin HK, Dec GW. ACC/AHA versus ESC guidelines on heart failure: JACC guideline comparison. *J Am Coll Cardiol*. 2019;73(21):2756-2768. https://doi.org/10.1016/j.jacc.2019.03.478

Weinberg ME, Bacchetti P, Rushakoff RJ. Frequently repeated glucose measurements overestimate the incidence of inpatient hypoglycemia and severe hyperglycemia. *J Diabetes Sci Technol.* 2010;4(3):577-582. https://doi.org/10.1177/193229681000400311

Wilson JM, Nikooienejad A, Robins DA, et al. The dual glucose-dependent insulinotropic peptide and glucagon-like peptide-1 receptor agonist, tirzepatide, improves lipoprotein biomarkers associated with insulin resistance and cardiovascular risk in patients with type 2 diabetes [published online August 16, 2020]. *Diab Obes and Metab*. https://doi.org/10.1111/dom.14174

Leo Document ID = f627040c-7fc7-4380-a51e-2965dac81d36

Approver: PPD

Approval Date & Time: 21-Jan-2022 13:08:17 GMT

Signature meaning: Approved

Approver: PPD

Approval Date & Time: 21-Jan-2022 13:14:00 GMT

Signature meaning: Approved

Title Page

Confidential Information

The information contained in this document is confidential and is intended for the use of clinical investigators. It is the property of Eli Lilly and Company or its subsidiaries and should not be copied by or distributed to persons not involved in the clinical investigation of tirzepatide (LY3298176), unless such persons are bound by a confidentiality agreement with Eli Lilly and Company or its subsidiaries.

Note to Regulatory Authorities: This document may contain protected personal data and/or commercially confidential information exempt from public disclosure. Eli Lilly and Company requests consultation regarding release/redaction prior to any public release. In the United States, this document is subject to Freedom of Information Act (FOIA) Exemption 4 and may not be reproduced or otherwise disseminated without the written approval of Eli Lilly and Company or its subsidiaries.

Protocol Title: A Randomized, Double-Blind, Placebo-Controlled, Phase 3 Study Comparing the Efficacy and Safety of Tirzepatide versus Placebo in Patients with Heart Failure with Preserved Ejection Fraction and Obesity (SUMMIT)

Protocol Number: I8F-MC-GPID

Amendment Number: c

Compound: LY3298176

Study Phase: 3

Short Title: Tirzepatide vs Placebo in Obesity-related HFpEF

Sponsor Name: Eli Lilly and Company

Legal Registered Address: Indianapolis, Indiana, USA 46285

Manufacturer: Eli Lilly and Company

Regulatory Agency Identifier Number

IND: 147352

Approval Date: Protocol Amendment (c) Electronically Signed and Approved by Lilly on date provided below.

Document ID: VV-CLIN-120976

Medical Monitor Name and Contact Information will be provided separately.

Protocol Amendment Summary of Changes Table

DOCUMENT HISTORY	
**Document**	**Date**
Amendment (b)	*21-Jan-2022*
Amendment (a)	*15-Dec-2021*
Original Protocol	*13-Jan-2021*

Amendment c

This amendment is considered to be substantial.

The amendment is considered to be substantial because of the change in design and methodology, and revision to the primary endpoints, which impacts the scientific value of the study.

Overall Rationale for the Amendment:

The purpose of this protocol amendment is to revise the dual primary endpoints.

Given the significant weight loss, and associated cardiometabolic improvements, achieved with tirzepatide, assessment of CV death and HF events, in addition to KCCQ score as a primary endpoint, offers the unique opportunity to evaluate tirzepatide for the benefit of patients with HFpEF and obesity. The dual primary endpoints described in GPID Protocol Amendment (b) may not be able to capture the full potential benefit offered by tirzepatide in this patient population.

Additionally, this amendment broadens the heart failure endpoint definition based on growing evidence that supports outpatient intensification of oral diuretics indicating worsening of HF. This change results in a new definition of HF event, defined as worsening symptoms or signs of HF, which are treated either as an inpatient (by hospitalization, treated by oral or IV diuretic intensification) or as an outpatient (by IV or oral diuretic intensification).

**Section # and Name**	**Description of Change**	**Brief Rationale**
1.1. Synopsis<br>3. Objectives and Endpoints	Revised primary endpoints to<br>• Change from baseline to Week 52 in the KCCQ-CSS<br>• Occurrence of the composite endpoint of CV death and/or HF events over time	Assessment of CV death and HF events, in addition to change from baseline to Week 52 in the KCCQ-CSS score as a primary endpoint, offers the unique opportunity to evaluate tirzepatide for the benefit of patients with HFpEF and obesity.<br>The hierarchical components using the win ratio were removed from alpha-adjusted endpoints as they present significant complexity in analytics and interpretation
	Key secondary endpoints revised to<br>• change from baseline to Week ~~24~~52 in 6MWD<br>• percent change from baseline to week 52 in body weight<br>• Change from baseline to Week 52 in hsCRP<br>~~• NYHA class change at week 52~~<br>~~• Change from baseline to week 52 in the KCCQ-CSS~~	Revisions aligned with change in primary endpoint. hsCRP added as key secondary as it is considered a clinically significant biomarker in HF population
	Removed "The maximum duration of participation depends on when the last participant completes 52 weeks of treatment" from Overall Design	This was duplicated text in same section
	EVa, EVb, EVc weeks from randomization updated to	Provides clarity of when the extended visits occur based

1.3. Schedule of Activities (SoA)	"(+3, 6, 9, 15, 18, 21 months from Visit 12)"	from Visit 12 and not from randomization
	EVd weeks from randomization updated to "(+12, 24 months from Visit 12)"	Provides clarity of when the extended visits occur based from Visit 12 and not from randomization
	"Including product complaints" removed from adverse events (AEs) row	Clarification; product complaints are not AEs
	Footnote "c" added sentence noting that if a participant requires a telephone visit, they must still pick up study drug from the site	Clarification
	Footnotes "d," "e," "f," "h," "j," "k," and "l" text in comment column of table moved to footnote section below SoA	Editorial
2.1. Study Rationale	Revised study objectives summary statement relative to updated endpoints	Alignment with updated endpoints
2.2. Background	Section revised and updated to provide additional background information and references	Alignment with updated endpoints
3. Objectives and Endpoints	Hierarchical composite assessed by win ratio moved to other secondary endpoint	Alignment with updated endpoints
	NYHA class, exercise capacity (6MWD at Week 24) moved from Key Secondary endpoints to Other Secondary endpoints	Exercise capacity analyzed as other secondary and NYHA class moved as part of the revised endpoint strategy

	Change from baseline to Week 24 in KCCQ-CSS added to other secondary endpoints<br>Proportion of participants attaining KCCQ-CSS meaningful within-patient change (MWPC) threshold at Week 52 added to other secondary endpoints	MWPC analysis added to support KCCQ-CSS
	Exploratory endpoint "HF medication use" integrated into primary endpoint CV death and/or HF event	Heart failure event definition expanded to include oral diuretic augmentation; therefore, a separate HF medication use is no longer needed
	Exploratory endpoint "Evaluation of prespecified biomarkers" hsCRP; moved to key secondary endpoint	Alignment with updated endpoints
	Added exploratory endpoints:<br>• Change from baseline to week 52 in waist to height ratio<br>• eGFR slope	Alignment with updated endpoints
4.1. Overall Design	Study Closeout and Final Visit: study duration revised as the duration of the trial depends on the last patient visit	Provides guidance to sites regarding final visit scheduling and expectations
	Study Closeout and Final Visit: indicated sponsor will notify sites of the study closeout based on the visit date of the last patient randomized	Provides guidance to sites regarding final visit scheduling and expectations

	Study Closeout and Final Visit: added statement that participants who have completed 52 weeks of the study are expected to complete V99 during the 3-month period prior to study close; no investigational product will be dispensed at the final visit	Provides guidance to sites regarding final visit scheduling and expectations
	Study Closeout and Final Visit: added "Any participant who has discontinued the study prior to completing 52 weeks of study duration is expected to complete an early termination visit per the SoA."	Provides guidance to sites of when to schedule final visit for participants
4.2. Scientific Rationale for Study Design	Revised section to provide rationale regarding updated primary endpoints	Alignment with revised endpoints
4.4. End of Study Definition	Clarified to indicate that the end of the study will occur approximately 52 weeks after the last participant has been randomized	Clarification
6.4. Study Intervention Compliance	Compliance revised as follows:<br>Treatment compliance ~~for each visit interval~~ is defined as taking at least 75% of the required doses of study drug	Clarification
6.5. Concomitant Therapy	Dosage information for concomitant therapy of special interest updated to include drugs for diabetes, obesity diuretics, and cardiovascular drugs	Clarification

6.6.1. Temporary Interruption	Indicated that for cases where increased ALT, AST, or ALP occur, close hepatic monitoring must be initiated	Language regarding hepatic safety streamlined for clarity and consistency
6.6.2. Restarting Study Drug after Interruption	Added “During re-escalation after a temporary dose interruption, participants should be followed every 4 weeks until either a new lower maintenance dose level or prior maintenance dose level is reached.”	Clarification
	Clarified that if an unscheduled visit occurs in the same week or date of a regular scheduled visit per the SoA, the site should complete all procedures included for the regular scheduled visit	Clarification
7.1.1. Permanent Discontinuation from Study Drug	Revised permanent discontinuation to indicate that participants who permanently discontinue the study drug will remain in the trial	Clarification
	Revised discontinuation due to hepatic event to reference appropriate section	Clarification
	Added bariatric surgery to permanent discontinuation circumstances	Bariatric surgery may pose a safety risk to subject taking IP
	Added GLP-1RA to permanent discontinuation circumstances	Added as co-administration of GLP-1RA and study drug may have safety implications to the participant
7.2. Participant Discontinuation/Withdrawal from the Study	Added instruction for sites regarding participants unwilling/unable to return for follow-up visits	Clarification

7.3. Lost to Follow up	Revised section to include updated instructions to sites regarding determination of participant vital status	Clarification to guide sites to continue attempts to reach participants until end of study
8.1.1. Primary Efficacy Assessment	Updated primary efficacy assessment to KCCQ-CSS and CV death and/or HF event	Alignment with revised primary endpoints
	Hierarchical composite assessed by win ratio moved to other secondary endpoint	Alignment with updated endpoints
	6MWT moved to Section 8.1.2	Alignment with revised primary endpoints
	Removed Time to All-Cause Mortality	Alignment with revised primary endpoints
8.1.1.2. Kansas City Cardiomyopathy Questionnaire	Addition of information regarding KCCQ collection at V8, V12, and ET	Clarification
8.1.1.2. Definition of Heart Failure Events	Revised definition of HF events to include outpatient intensification of oral diuretics due to HF events	Alignment with revised primary endpoints
8.1.2. Secondary Efficacy Assessments	Revised section header to "~~Additional~~ Secondary Efficacy Assessments"	Clarification
	Addition of information regarding 6MWT collection at V8, V12, and ET	Clarification
	Revised secondary endpoint "Body Weight" to include hsCRP; revised section subheader: "Body Weight and hsCRP"	hsCRP assessment added to align with change to key secondary endpoints
8.1.3. Exploratory Efficacy Assessments	Moved NYHA Classification from Secondary Efficacy	Alignment with revised primary and secondary endpoints

	Assessments to Exploratory Efficacy Assessments	
8.2.5.1. Hepatic Safety Monitoring, Evaluation, and Criteria for Study Drug Interruption or Discontinuation	Section header updated, addition of tables for hepatic safety monitoring which indicate when to initiate close hepatic monitoring, comprehensive evaluation, or interrupt or discontinue study drug	Updated to current Lilly procedures. No major changes
8.2.5.2. Close Hepatic Monitoring	Separate section header created, removed table, addition of CBC with differential to laboratory tests	Clarification
8.2.5.3. Comprehensive Hepatic Evaluation	Separate section header created, removed table	Clarification
8.2.5.4. Study Drug Interruption or Discontinuation due to a Hepatic Event	Section added; additional instruction provided regarding study drug interruption or discontinuation	Clarification
8.3.1. Timing and Mechanism for Collecting Events	Pregnancy reporting mechanism and back-up mechanism updated to pregnancy paper form eCRF and pregnancy paper form respectively	Correction
8.3.2. Primary, Secondary, and Additional Study Endpoint Reporting	Revised “hospitalization for HF or an urgent HF visit” to “HF events”	Alignment with revised primary and secondary endpoints
8.4. Treatment of Overdose	Removed reference to IB	Template update
	Definition of overdose updated to injection of study drug more than 1 time in 72 hours	Correction and clarification

	Revised required investigator action in the event of an overdose	Correction and clarification
9.1. Statistical Hypotheses	Revised primary hypotheses to<br>• tirzepatide MTD is superior to placebo for the change from baseline in KCCQ-CSS at Week 52<br>• tirzepatide MTD is superior to placebo for the occurrence of the composite endpoint of CV death and/or HF events over time	Alignment with revised primary endpoints
	Revised key secondary hypotheses to that tirzepatide MTD is superior to placebo with regards to:<br>• change from baseline in 6MWD at Week ~~24~~52<br>• percent change from baseline in body weight at Week 52<br>• change from baseline in hsCRP at Week 52	Alignment with revised endpoints
	Revised statistical methods related to updated primary and key secondary endpoints	Alignment with revised primary and key secondary endpoints
9.2. Sample Size Determination	Updated sample size justification description as related to revised primary endpoints	Alignment with revised primary endpoints
9.4.2. Primary Endpoint(s)	Revised statistical methods related to primary endpoints; removed hierarchical	Alignment with revised primary endpoints

	composite endpoint and change in 6MWD, added KCCQ-CSS (9.4.2.1) and HF outcomes (9.4.2.2)	
9.4.3. Key Secondary Endpoint(s)	Revised statistical methods related to key secondary endpoints:<br>• KCCQ-CSS endpoint moved to primary endpoint<br>• 6MWD revised from 24 weeks to 52 weeks<br>• Analysis method of percent change in body weight revised from MMRM to ANCOVA<br>• NYHA class removed<br>• hsCRP endpoint added (ANCOVA analysis)	Alignment with revised key secondary endpoints and correct analysis method appropriate for the estimand
9.4.4. Tertiary/Exploratory Endpoint(s)	"Other secondary" removed from section	Alignment with revised secondary endpoints
10.4.2. Female participants	Revised contraception requirement from 2 months after the last administration of study drug to 4 weeks	Update to align with Investigator's Brochure
Throughout the protocol	Minor editorial corrections, minor clarifying changes	Minor editorial changes, therefore not described; clarification

Table of Contents

1. Protocol Summary 16
1.1. Synopsis 16
1.2. Schema 18
1.3. Schedule of Activities (SoA) 19

2. Introduction 42
2.1. Study Rationale 42
2.2. Background 42
2.3. Benefit/Risk Assessment 43

3. Objectives and Endpoints 44

4. Study Design 47
4.1. Overall Design 47
4.2. Scientific Rationale for Study Design 49
4.2.1. Patient Input into Design 50
4.3. Justification for Dose 50
4.4. End of Study Definition 50

5. Study Population 51
5.1. Inclusion Criteria 51
5.2. Exclusion Criteria 53
5.3. Lifestyle Considerations 55
5.4. Screen Failures 55

6. Study Intervention 57
6.1. Study Interventions Administered 57
6.1.1. Medical Devices 58
6.2. Preparation/Handling/Storage/Accountability 58
6.3. Measures to Minimize Bias: Randomization and Blinding 59
6.4. Study Intervention Compliance 59
6.5. Concomitant Therapy 60
6.6. Dose Modification 63
6.6.1. Temporary Interruption 63
6.6.2. Restarting Study Drug after Interruption 64
6.7. Intervention after the End of the Study 65

7. Discontinuation of Study Intervention and Participant Discontinuation/Withdrawal 66
7.1. Discontinuation of Study Intervention 66
7.1.1. Permanent Discontinuation from Study Drug 66
7.2. Participant Discontinuation/Withdrawal from the Study 67
7.2.1. Inadvertently Enrolled Participants 68
7.3. Lost to Follow up 68

8. Study Assessments and Procedures 70
8.1. Efficacy Assessments 70
8.1.1. Primary Efficacy Assessment 70

8.1.2. Secondary Efficacy Assessments 71
8.1.3. Exploratory Efficacy Assessments 72
8.2. Safety Assessments 73
8.2.1. Physical Examinations 73
8.2.2. Vital Signs 73
8.2.3. Electrocardiograms 74
8.2.4. Clinical Safety Laboratory Assessments 74
8.2.5. Safety Monitoring 75
8.3. Adverse Events, Serious Adverse Events, and Product Complaints 78
8.3.1. Timing and Mechanism for Collecting Events 79
8.3.2. Primary, Secondary, and Additional Study Endpoint Reporting 81
8.3.3. Adverse Events of Special Interest 82
8.4. Treatment of Overdose 82
8.5. Pharmacokinetics 82
8.6. Pharmacodynamics 83
8.7. Genetics 83
8.8. Biomarkers 83
8.9. Immunogenicity Assessments 84
8.10. Medical Resource Utilization and Health Economics 84

9. Statistical Considerations 85
9.1. Statistical Hypotheses 85
9.2. Sample Size Determination 85
9.3. Populations for Analyses 86
9.4. Statistical Analyses 86
9.4.1. General Considerations 86
9.4.2. Primary Endpoint(s) 86
9.4.3. Key Secondary Endpoint(s) 87
9.4.4. Tertiary/Exploratory Endpoint(s) 88
9.4.5. Other Safety Analyses 88
9.4.6. Subgroup Analyses 88
9.5. Interim Analyses 88
9.6. Data Monitoring Committee 89

10. Supporting Documentation and Operational Considerations 90
10.1. Appendix 1: Regulatory, Ethical, and Study Oversight Considerations 90
10.1.1. Regulatory and Ethical Considerations 90
10.1.2. Financial Disclosure 90
10.1.3. Informed Consent Process 91
10.1.4. Data Protection 91
10.1.5. Dissemination of Clinical Study Data 91
10.1.6. Data Quality Assurance 92
10.1.7. Source Documents 93
10.1.8. Study and Site Start and Closure 94
10.1.9. Publication Policy 94
10.1.10. Long-Term Sample Retention 94

10.1.11. Investigator Information 95
10.2. Appendix 2: Clinical Laboratory Tests 95
10.3. Appendix 3: Adverse Events: Definitions and Procedures for Recording, Evaluating, Follow-up, and Reporting 98
10.3.1. Definition of AE 98
10.3.2. Definition of SAE 99
10.3.3. Definition of Product Complaints 101
10.3.4. Recording and Follow-Up of AE and/or SAE and Product Complaints 101
10.3.5. Reporting of SAEs 103
10.3.6. Regulatory Reporting Requirements 103
10.4. Appendix 4: Contraceptive Guidance and Collection of Pregnancy Information 104
10.4.1. Male participants: 104
10.4.2. Female participants: 104
10.5. Appendix 5: Adverse Events of Special Interest: Definitions and Procedures for Recording, Evaluating, Follow-Up, and Reporting. 108
10.5.1. Special Safety Topics 108
10.6. Appendix 6: Genetics 113
10.7. Appendix 7: Recommended Laboratory Testing for Hypersensitivity Events 114
10.8. Appendix 8: Liver Safety: Suggested Actions and Follow-Up Assessments 116
10.9. Appendix 9: Medical Device Adverse Events (AEs), Adverse Device Effects (ADEs), Serious Adverse Events (SAEs) and Device Deficiencies: Definition and Procedures for Recording, Evaluating, Follow-Up, and Reporting 118
10.10. Appendix 10: Six-Minute Walk Test Screening Procedures 119
10.10.1. Screening Procedures and Flow Diagram 119
10.11. Appendix 11: New York Heart Association Classification of Heart Failure 120
10.12. Appendix 12: Abbreviations 121
10.13. Appendix 13: Protocol Amendment History 126
11. References 131

1. Protocol Summary

1.1. Synopsis

Protocol Title: A Randomized, Double-Blind, Placebo-Controlled, Phase 3 Study Comparing the Efficacy and Safety of Tirzepatide versus Placebo in Patients with Heart Failure with Preserved Ejection Fraction and Obesity (SUMMIT)

Short Title: Tirzepatide vs Placebo in Obesity-related HFpEF

Rationale:

Heart failure with preserved ejection fraction is a heterogenous clinical syndrome resulting from various pathophysiological processes. Among the broad spectrum of HFpEF clinical presentation, obesity-related HFpEF displays a distinct phenotype where increased visceral and ectopic adiposity as well as volume expansion plays a causal role (Kitzman and Shah 2016; Packer 2018; Miller and Borlaug 2020). Given tirzepatide's anti-inflammatory and antifibrotic effects and a reduction in circulating plasma volume as a consequence of the treatment of obesity, tirzepatide may provide clinical benefit to patients with HFpEF and BMI ≥30 kg/m^2.

Study I8F-MC-GPID, also known as SUMMIT, is a Phase 3, randomized, multicenter, international, placebo-controlled, double-blinded, parallel-arm study. This study will evaluate the effect of SC QW injection of tirzepatide, MTD up to 15 mg, on the health status, risk of death, HF events, and exercise capacity in participants with HFpEF and BMI ≥30 kg/m^2.

Objectives and Endpoints

Objectives	Endpoints
Primary	
To demonstrate that a maximally tolerated tirzepatide dose up to 15 mg administered SC QW is superior to placebo to improve patient-reported symptoms and physical limitations in participants with HFpEF and obesity	Change from baseline to Week 52 in the KCCQ-CSS
To demonstrate that a maximally tolerated tirzepatide dose up to 15 mg administered SC QW is superior to placebo based on the composite HF outcome endpoint in participants with HFpEF and obesity	Occurrence of the composite endpoint of CV death and/or HF events over time
Key Secondary (multiplicity controlled)	
Exercise capacity	Change from baseline to Week 52 in 6MWD

Long-term weight loss	Percent change from baseline to Week 52 in body weight
Evaluation of change in inflammation	Change from baseline to Week 52 in hsCRP

Abbreviations: 6MWD = 6-minute walk distance; CV = cardiovascular; HF = heart failure; HFpEF = heart failure with preserved ejection fraction; KCCQ-CSS = Kansas City Cardiomyopathy Questionnaire – Clinical Summary Score; QW = once weekly; SC = subcutaneous.

Overall Design

Study GPID is a randomized, outpatient, multicenter, international, placebo-controlled, double-blind, parallel-arm, Phase 3 study with 2 study periods. The study is designed to evaluate the efficacy and safety of SC QW tirzepatide, MTD up to 15 mg, compared to placebo, in participants with HFpEF and obesity.

Two intervention groups will be studied:

- Tirzepatide, MTD up to 15 mg, SC QW
- Placebo

The starting dose of tirzepatide is 2.5 mg QW, which is to be escalated at 4-week intervals to a maximum of 15 mg QW or to the highest maintenance dose tolerated by the participant (see Section 6).

Disclosure Statement: This is a parallel-treatment study with 2 intervention groups that is double blinded.

Number of Participants:

Approximately 700 participants will be randomly assigned to study drug with approximately 350 participants per intervention group.

Intervention Groups and Duration:

The study will compare treatment with tirzepatide and treatment with placebo. Assignment to tirzepatide or placebo groups will be randomly allocated in a 1:1 ratio.

The starting dose of tirzepatide 2.5 mg QW is to be escalated to 15 mg QW or the highest maintenance dose tolerated by the participant (5 mg, 10 mg, QW).

Study participation for each participant is sectioned into the following study periods:

- Study Period 1: screening period, approximately 6 weeks and not more than 12 weeks
- Study Period 2: treatment period, at least 52 weeks

The study will continue until approximately 52 weeks after the last participant is randomized. The maximum duration of an individual's participation is estimated to be 120 weeks and will depend on duration of study enrollment.

Data Monitoring Committee: Yes

1.2. Schema

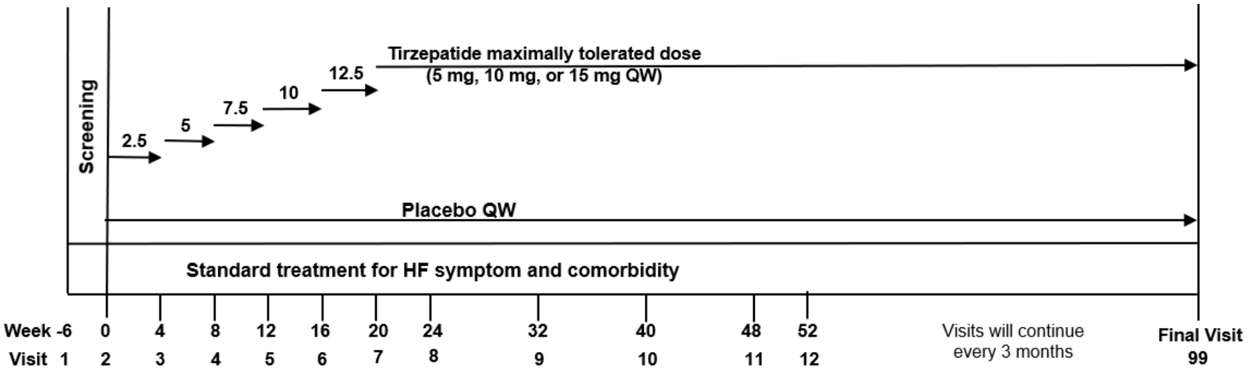

Abbreviations: HF = heart failure; QW = weekly.

Note: Screening procedures may take longer or shorter than 6 weeks but no more than 12 weeks.

1.3. Schedule of Activities (SoA)

Visit 1 and 2 procedures may be conducted over more than 1 day each as long as all activities are completed within the allowable visit interval tolerance for each visit.

For early terminations (ET) from the study that occur before the final visit (V99) in treatment period, see the activities listed for ET in this table.

Study I8F-MC-GPID	Study Period I Screening	Study Period II - Treatment period																		
														Unscheduled Visit (UV)[a]						
Visit number	1	2	3	4	5	6	7	8	9	10	11	12	EVa, EVb, EVc (13, 14, 15, 17, 18, 19, etc.)	EVd (16, 20, etc.)	UV	Dosing UV	Phone Follow-Up Dosing UV	ET	99	Comment
Weeks from randomization	-6	0	4	8	12	16	20	24	32	40	48	52	(+3, 6, 9, 15, 18, 21 months from Visit 12)	(+12, 24 months from Visit 12)				—	Final Visit	See footnote b
Visit interval tolerance (days)			±3	±3	±3	±3	±3	±3	±7	±7	±7	±7	±15	±15				—		
Telephone Visit									X		X		X				X			See footnote c
Informed consent	X																			
Inclusion and exclusion criteria, review and confirm	X	X																		

Study I8F-MC-GPID	Study Period I Screening	Study Period II - Treatment period																			
														Unscheduled Visit (UV)[a]						
Visit number	1	2	3	4	5	6	7	8	9	10	11	12	EVa, EVb, EVc (13, 14, 15, 17, 18, 19, etc.)	EVd (16, 20, etc.)	UV	Dosing UV	Phone Follow-Up Dosing UV	ET	99	Comment
Weeks from randomization	-6	0	4	8	12	16	20	24	32	40	48	52	(+3, 6, 9, 15, 18, 21 months from Visit 12)	(+12, 24 months from Visit 12)				—	Final Visit	See footnote b
Visit interval tolerance (days)			±3	±3	±3	±3	±3	±3	±7	±7	±7	±7	±15	±15				—		
Telephone Visit									X		X		X				X			See footnote c
Demographics	X																			
Preexisting conditions and medical history, including relevant surgical history	X																			

Study I8F-MC-GPID	Study Period I Screening	Study Period II - Treatment period																		
														Unscheduled Visit (UV)[a]						
Visit number	1	2	3	4	5	6	7	8	9	10	11	12	EVa, EVb, EVc (13, 14, 15, 17, 18, 19, etc.)	EVd (16, 20, etc.)	UV	Dosing UV	Phone Follow-Up Dosing UV	ET	99	Comment
Weeks from randomization	-6	0	4	8	12	16	20	24	32	40	48	52	(+3, 6, 9, 15, 18, 21 months from Visit 12)	(+12, 24 months from Visit 12)				—	Final Visit	See footnote b
Visit interval tolerance (days)			±3	±3	±3	±3	±3	±3	±7	±7	±7	±7	±15	±15				—		
Telephone Visit									X		X		X				X			See footnote c
Prespecified medical history (indication and history of interest)	X																			Includes HF history, hospitalization for HF, CVD, MI, atrial fibrillation, stroke, CV risk (T2DM, HTN, dyslipidemia, metabolic syndrome)
Prior treatments for HFpEF	X																			
Substance use (alcohol, tobacco use)	X																			

Study I8F-MC-GPID	Study Period I Screening	Study Period II - Treatment period																		
														Unscheduled Visit (UV)[a]						
Visit number	1	2	3	4	5	6	7	8	9	10	11	12	EVa, EVb, EVc (13, 14, 15, 17, 18, 19, etc.)	EVd (16, 20, etc.)	UV	Dosing UV	Phone Follow-Up Dosing UV	ET	99	Comment
Weeks from randomization	-6	0	4	8	12	16	20	24	32	40	48	52	(+3, 6, 9, 15, 18, 21 months from Visit 12)	(+12, 24 months from Visit 12)				—	Final Visit	See footnote b
Visit interval tolerance (days)			±3	±3	±3	±3	±3	±3	±7	±7	±7	±7	±15	±15				—		
Telephone Visit									X		X		X				X			See footnote c
Concomitant medications	X	X	X	X	X	X	X	X	X	X	X	X	X	X	X	X	X	X	X	
Adverse events (AEs)	X	X	X	X	X	X	X	X	X	X	X	X	X	X	X	X	X	X	X	
**Physical Evaluation**																				
Height	X																			
Weight	X	X	X	X	X	X	X	X		X		X		X	X	X		X	X	
Waist circumference	X	X						X				X		X				X	X	

Study I8F-MC-GPID	Study Period I Screening	Study Period II - Treatment period																		
														Unscheduled Visit (UV)[a]						
Visit number	1	2	3	4	5	6	7	8	9	10	11	12	EVa, EVb, EVc (13, 14, 15, 17, 18, 19, etc.)	EVd (16, 20, etc.)	UV	Dosing UV	Phone Follow-Up Dosing UV	ET	99	Comment
Weeks from randomization	-6	0	4	8	12	16	20	24	32	40	48	52	(+3, 6, 9, 15, 18, 21 months from Visit 12)	(+12, 24 months from Visit 12)				—	Final Visit	See footnote b
Visit interval tolerance (days)			±3	±3	±3	±3	±3	±3	±7	±7	±7	±7	±15	±15				—		
Telephone Visit									X		X		X				X			See footnote c
Vital Signs	X	X	X	X	X	X	X	X		X		X		X	X	X		X	X	Include 2 Sitting BP and HR. HR to be performed by apical auscultation. Vital signs should be collected prior to the first 6MWT of the day and before ECG.
Physical examination	X											X		X				X		

Study I8F-MC-GPID	Study Period I Screening	Study Period II - Treatment period																		
														Unscheduled Visit (UV)[a]						
Visit number	1	2	3	4	5	6	7	8	9	10	11	12	EVa, EVb, EVc (13, 14, 15, 17, 18, 19, etc.)	EVd (16, 20, etc.)	UV	Dosing UV	Phone Follow-Up Dosing UV	ET	99	Comment
Weeks from randomization	-6	0	4	8	12	16	20	24	32	40	48	52	(+3, 6, 9, 15, 18, 21 months from Visit 12)	(+12, 24 months from Visit 12)				—	Final Visit	See footnote b
Visit interval tolerance (days)			±3	±3	±3	±3	±3	±3	±7	±7	±7	±7	±15	±15				—		
Telephone Visit									X		X		X				X			See footnote c
Symptom-directed physical examination		X	X	X	X	X	X	X		X		X		X	X	X			X	As indicated based on participant status and standard of care, including dyspnea, orthopnea, paroxysmal nocturnal dyspnea (PND), edema, jugular venous distension (JVD), rales

Study I8F-MC-GPID	Study Period I Screening	Study Period II - Treatment period																		
														Unscheduled Visit (UV)[a]						
Visit number	1	2	3	4	5	6	7	8	9	10	11	12	EVa, EVb, EVc (13, 14, 15, 17, 18, 19, etc.)	EVd (16, 20, etc.)	UV	Dosing UV	Phone Follow-Up Dosing UV	ET	99	Comment
Weeks from randomization	-6	0	4	8	12	16	20	24	32	40	48	52	(+3, 6, 9, 15, 18, 21 months from Visit 12)	(+12, 24 months from Visit 12)				—	Final Visit	See footnote b
Visit interval tolerance (days)			±3	±3	±3	±3	±3	±3	±7	±7	±7	±7	±15	±15				—		
Telephone Visit									X		X		X				X			See footnote c
NYHA class assessment	X	X						X				X		X				X[d]		NYHA class assessment must be performed by an independent assessor. See footnote d.
HF events		X	X	X	X	X	X	X	X	X	X	X	X	X	X	X		X	X	
Evaluation of injection site reactions		X	X	X	X	X	X	X		X		X		X	X	X		X	X	

Study I8F-MC-GPID	Study Period I Screening	Study Period II - Treatment period																		
														Unscheduled Visit (UV)[a]						
Visit number	1	2	3	4	5	6	7	8	9	10	11	12	EVa, EVb, EVc (13, 14, 15, 17, 18, 19, etc.)	EVd (16, 20, etc.)	UV	Dosing UV	Phone Follow-Up Dosing UV	ET	99	Comment
Weeks from randomization	-6	0	4	8	12	16	20	24	32	40	48	52	(+3, 6, 9, 15, 18, 21 months from Visit 12)	(+12, 24 months from Visit 12)				—	Final Visit	See footnote b
Visit interval tolerance (days)			±3	±3	±3	±3	±3	±3	±7	±7	±7	±7	±15	±15				—		
Telephone Visit									X		X		X				X			See footnote c
Single-read 12-lead ECG	X	X						X				X						X[d]		Collect locally. Report atrial fibrillation or other abnormalities on the eCRF. Optional ECG is allowable if indicated. See footnote d
Echocardiography	X																			For those required to complete the ECHO examination

Study I8F-MC-GPID	Study Period I Screening	Study Period II - Treatment period																		
														Unscheduled Visit (UV)[a]						
Visit number	1	2	3	4	5	6	7	8	9	10	11	12	EVa, EVb, EVc (13, 14, 15, 17, 18, 19, etc.)	EVd (16, 20, etc.)	UV	Dosing UV	Phone Follow-Up Dosing UV	ET	99	Comment
Weeks from randomization	-6	0	4	8	12	16	20	24	32	40	48	52	(+3, 6, 9, 15, 18, 21 months from Visit 12)	(+12, 24 months from Visit 12)				—	Final Visit	See footnote b
Visit interval tolerance (days)			±3	±3	±3	±3	±3	±3	±7	±7	±7	±7	±15	±15				—		
Telephone Visit									X		X		X				X			See footnote c
Dilated fundoscopic examination	X																			Perform for participants with T2DM who have not had an evaluable dilated fundoscopic examination in the last 12 months. See exclusion criterion 25 (Section 5.2)

Study I8F-MC-GPID	Study Period I Screening	Study Period II - Treatment period																		
														Unscheduled Visit (UV)[a]						
Visit number	1	2	3	4	5	6	7	8	9	10	11	12	EVa, EVb, EVc (13, 14, 15, 17, 18, 19, etc.)	EVd (16, 20, etc.)	UV	Dosing UV	Phone Follow-Up Dosing UV	ET	99	Comment
Weeks from randomization	-6	0	4	8	12	16	20	24	32	40	48	52	(+3, 6, 9, 15, 18, 21 months from Visit 12)	(+12, 24 months from Visit 12)				—	Final Visit	See footnote b
Visit interval tolerance (days)			±3	±3	±3	±3	±3	±3	±7	±7	±7	±7	±15	±15				—		
Telephone Visit									X		X		X				X			See footnote c
																				Follow-up dilated fundoscopic examination should be performed when clinically indicated by any AE suspected of worsening retinopathy.
6MWT	X[e]	X[f]						X				X						X[d]		Ensure that participant completes the associated Borg Questionnaire prior to and after the 6MWT. See footnotes e, f, and d
**Participant Education**																				

Study I8F-MC-GPID	Study Period I Screening	Study Period II - Treatment period																		
															Unscheduled Visit (UV)[a]					
Visit number	1	2	3	4	5	6	7	8	9	10	11	12	EVa, EVb, EVc (13, 14, 15, 17, 18, 19, etc.)	EVd (16, 20, etc.)	UV	Dosing UV	Phone Follow-Up Dosing UV	ET	99	Comment
Weeks from randomization	-6	0	4	8	12	16	20	24	32	40	48	52	(+3, 6, 9, 15, 18, 21 months from Visit 12)	(+12, 24 months from Visit 12)				—	Final Visit	See footnote b
Visit interval tolerance (days)			±3	±3	±3	±3	±3	±3	±7	±7	±7	±7	±15	±15				—		
Telephone Visit									X		X		X				X			See footnote c
Diary instruction		X																		
**Participant Diary**																				
Participant diary dispensed		X	X	X	X	X	X	X		X		X		X						
Diary compliance check/Assess study drug compliance[g]			X	X	X	X	X	X	X	X	X	X	X	X				X	X	
Diary return			X	X	X	X	X	X		X		X		X				X	X	
**Patient-Reported Outcomes (PROs) (Electronic[h])**																				See footnote h

Study I8F-MC-GPID	Study Period I Screening	Study Period II - Treatment period																		
														Unscheduled Visit (UV)[a]						
Visit number	1	2	3	4	5	6	7	8	9	10	11	12	EVa, EVb, EVc (13, 14, 15, 17, 18, 19, etc.)	EVd (16, 20, etc.)	UV	Dosing UV	Phone Follow-Up Dosing UV	ET	99	Comment
Weeks from randomization	-6	0	4	8	12	16	20	24	32	40	48	52	(+3, 6, 9, 15, 18, 21 months from Visit 12)	(+12, 24 months from Visit 12)				—	Final Visit	See footnote b
Visit interval tolerance (days)			±3	±3	±3	±3	±3	±3	±7	±7	±7	±7	±15	±15				—		
Telephone Visit									X		X		X				X			See footnote c
Kansas City Cardiomyopathy Questionnaire (KCCQ)	X	X						X				X						X		
EQ-5D-5L		X						X				X						X		
Patient Global Impression of Status – Overall (PGIS-Overall)	X	X						X				X						X		

Study I8F-MC-GPID	Study Period I Screening	Study Period II - Treatment period																		
														Unscheduled Visit (UV)[a]						
Visit number	1	2	3	4	5	6	7	8	9	10	11	12	EVa, EVb, EVc (13, 14, 15, 17, 18, 19, etc.)	EVd (16, 20, etc.)	UV	Dosing UV	Phone Follow-Up Dosing UV	ET	99	Comment
Weeks from randomization	-6	0	4	8	12	16	20	24	32	40	48	52	(+3, 6, 9, 15, 18, 21 months from Visit 12)	(+12, 24 months from Visit 12)				—	Final Visit	See footnote b
Visit interval tolerance (days)			±3	±3	±3	±3	±3	±3	±7	±7	±7	±7	±15	±15				—		
Telephone Visit									X		X		X				X			See footnote c
Patient Global Impression of Status – Physical Function (PGIS-Physical Function)	X	X						X				X						X		
Patient Global Impression of Status – Symptom Severity (PGIS-Symptom Severity)	X	X						X				X						X		
**Laboratory Tests and Sample Collections**																				

Study I8F-MC-GPID	Study Period I Screening	Study Period II - Treatment period																		
														Unscheduled Visit (UV)[a]						
Visit number	1	2	3	4	5	6	7	8	9	10	11	12	EVa, EVb, EVc (13, 14, 15, 17, 18, 19, etc.)	EVd (16, 20, etc.)	UV	Dosing UV	Phone Follow-Up Dosing UV	ET	99	Comment
Weeks from randomization	-6	0	4	8	12	16	20	24	32	40	48	52	(+3, 6, 9, 15, 18, 21 months from Visit 12)	(+12, 24 months from Visit 12)				—	Final Visit	See footnote b
Visit interval tolerance (days)			±3	±3	±3	±3	±3	±3	±7	±7	±7	±7	±15	±15				—		
Telephone Visit									X		X		X				X			See footnote c
Hematology	X	X			X			X				X		X	X[i]	X[i]		X	X	See footnote i
Hemoglobin A1c (HbA1c)	X	X	X	X	X	X	X	X		X		X		X				X	X	
Clinical chemistry (with glucose)	X	X[j]			X			X				X		X	X[i]	X[i]		X	X	See footnote j
Lipid panel	X	X[j]			X			X				X		X	X[i]	X[di]		X	X	See footnote j
Thyroid-stimulating hormone (TSH)	X																			

Study I8F-MC-GPID	Study Period I Screening	Study Period II - Treatment period																		
														Unscheduled Visit (UV)[a]						
Visit number	1	2	3	4	5	6	7	8	9	10	11	12	EVa, EVb, EVc (13, 14, 15, 17, 18, 19, etc.)	EVd (16, 20, etc.)	UV	Dosing UV	Phone Follow-Up Dosing UV	ET	99	Comment
Weeks from randomization	-6	0	4	8	12	16	20	24	32	40	48	52	(+3, 6, 9, 15, 18, 21 months from Visit 12)	(+12, 24 months from Visit 12)				—	Final Visit	See footnote b
Visit interval tolerance (days)			±3	±3	±3	±3	±3	±3	±7	±7	±7	±7	±15	±15				—		
Telephone Visit									X		X		X				X			See footnote c
Serum pregnancy	X	X[k]																		For women of childbearing potential only. See Appendix 4 (Section 10.4). See footnote k

Urine pregnancy (local)		X	X	X	X	X	X	X		X		X		X				X	X	A local urine pregnancy test must be performed at Visit 2 after patient eligibility has been confirmed with the result available prior to randomization and first injection of study drug(s) for WOCBP only. Additional local urine pregnancy tests may be performed at the investigator's discretion during the study. If required per local regulations and/or institutional guidelines, pregnancy testing can also occur at other times during the study treatment period. See Appendix 4 (Section 10.4).
Follicle-stimulating hormone (FSH)	X																			Collect FSH only in women whose menopausal status needs to be determined. For participants known to be either premenopausal or postmenopausal, these tests do not need to be collected
NT-proBNP	X	X			X			X				X						X d		See footnote d

Study I8F-MC-GPID	Study Period I Screening	Study Period II - Treatment period																		
														Unscheduled Visit (UV)[a]						
Visit number	1	2	3	4	5	6	7	8	9	10	11	12	EVa, EVb, EVc (13, 14, 15, 17, 18, 19, etc.)	EVd (16, 20, etc.)	UV	Dosing UV	Phone Follow-Up Dosing UV	ET	99	Comment
Weeks from randomization	-6	0	4	8	12	16	20	24	32	40	48	52	(+3, 6, 9, 15, 18, 21 months from Visit 12)	(+12, 24 months from Visit 12)				—	Final Visit	See footnote b
Visit interval tolerance (days)			±3	±3	±3	±3	±3	±3	±7	±7	±7	±7	±15	±15				—		
Telephone Visit									X		X		X				X			See footnote c
Cardiac troponin T (cTnT)		X			X			X				X						X[d]		See footnote d
Calcitonin	X							X				X		X				X	X	
Cystatin C		X			X			X				X						X[d]		See footnote d
C-reactive protein, high-sensitivity (hsCRP)		X			X			X				X						X[d]		See footnote d
Pancreatic amylase	X	X[j]			X			X		X		X		X				X	X	See footnote j

Study I8F-MC-GPID	Study Period I Screening	Study Period II - Treatment period																		
														Unscheduled Visit (UV)[a]						
Visit number	1	2	3	4	5	6	7	8	9	10	11	12	EVa, EVb, EVc (13, 14, 15, 17, 18, 19, etc.)	EVd (16, 20, etc.)	UV	Dosing UV	Phone Follow-Up Dosing UV	ET	99	Comment
Weeks from randomization	-6	0	4	8	12	16	20	24	32	40	48	52	(+3, 6, 9, 15, 18, 21 months from Visit 12)	(+12, 24 months from Visit 12)				—	Final Visit	See footnote b
Visit interval tolerance (days)			±3	±3	±3	±3	±3	±3	±7	±7	±7	±7	±15	±15				—		
Telephone Visit									X		X		X				X			See footnote c
Lipase	X	X[j]			X			X		X		X		X				X	X	See footnote j
eGFR (CKD-EPI)	X	X[l]			X			X				X		X				X	X	The CKD-EPI equation will be used by the central lab to estimate and report eGFR. See footnote l
Urinary albumin/creatinine ratio (UACR)	X							X				X		X				X	X	

Study I8F-MC-GPID	Study Period I Screening	Study Period II - Treatment period																		
														Unscheduled Visit (UV)[a]						
Visit number	1	2	3	4	5	6	7	8	9	10	11	12	EVa, EVb, EVc (13, 14, 15, 17, 18, 19, etc.)	EVd (16, 20, etc.)	UV	Dosing UV	Phone Follow-Up Dosing UV	ET	99	Comment
Weeks from randomization	-6	0	4	8	12	16	20	24	32	40	48	52	(+3, 6, 9, 15, 18, 21 months from Visit 12)	(+12, 24 months from Visit 12)				—	Final Visit	See footnote b
Visit interval tolerance (days)			±3	±3	±3	±3	±3	±3	±7	±7	±7	±7	±15	±15				—		
Telephone Visit									X		X		X				X			See footnote c
Pharmacokinetic (PK) samples		X	X		X			X				X		X				X	X	PK samples should be taken prior to dose administration at the visit and at the same time as immunogenicity samples.
Immunogenicity samples		X	X		X			X				X		X				X	X	
**Stored Samples**																				
Genetics sample		X																		
Exploratory biomarker samples		X			X			X				X								

Study I8F-MC-GPID	Study Period I Screening	Study Period II - Treatment period																		
														Unscheduled Visit (UV)[a]						
Visit number	1	2	3	4	5	6	7	8	9	10	11	12	EVa, EVb, EVc (13, 14, 15, 17, 18, 19, etc.)	EVd (16, 20, etc.)	UV	Dosing UV	Phone Follow-Up Dosing UV	ET	99	Comment
Weeks from randomization	-6	0	4	8	12	16	20	24	32	40	48	52	(+3, 6, 9, 15, 18, 21 months from Visit 12)	(+12, 24 months from Visit 12)				—	Final Visit	See footnote b
Visit interval tolerance (days)			±3	±3	±3	±3	±3	±3	±7	±7	±7	±7	±15	±15				—		
Telephone Visit									X		X		X				X			See footnote c
**Randomization and Dosing**																				
Randomization		X																		
Dispense study drug		X	X	X	X	X	X	X		X		X	X	X		X				
Injection training with autoinjector demonstration device		X																		

Study I8F-MC-GPID	Study Period I Screening	Study Period II - Treatment period																		
															Unscheduled Visit (UV)[a]					
Visit number	1	2	3	4	5	6	7	8	9	10	11	12	EVa, EVb, EVc (13, 14, 15, 17, 18, 19, etc.)	EVd (16, 20, etc.)	UV	Dosing UV	Phone Follow-Up Dosing UV	ET	99	Comment
Weeks from randomization	-6	0	4	8	12	16	20	24	32	40	48	52	(+3, 6, 9, 15, 18, 21 months from Visit 12)	(+12, 24 months from Visit 12)				—	Final Visit	See footnote b
Visit interval tolerance (days)			±3	±3	±3	±3	±3	±3	±7	±7	±7	±7	±15	±15				—		
Telephone Visit									X		X		X				X			See footnote c
Observe participant administer study drug		X	X	X	X	X	X	X		X		X		X		X				Participants should administer the first dose of study drug at Visit 2 after study procedures and randomization have been completed. Review technique with the participant at each in clinic visit.

Study I8F-MC-GPID	Study Period I Screening	Study Period II - Treatment period																		
														Unscheduled Visit (UV)[a]						
Visit number	1	2	3	4	5	6	7	8	9	10	11	12	EVa, EVb, EVc (13, 14, 15, 17, 18, 19, etc.)	EVd (16, 20, etc.)	UV	Dosing UV	Phone Follow-Up Dosing UV	ET	99	Comment
Weeks from randomization	-6	0	4	8	12	16	20	24	32	40	48	52	(+3, 6, 9, 15, 18, 21 months from Visit 12)	(+12, 24 months from Visit 12)				—	Final Visit	See footnote b
Visit interval tolerance (days)			±3	±3	±3	±3	±3	±3	±7	±7	±7	±7	±15	±15				—		
Telephone Visit									X		X		X				X			See footnote c
																				Sites should coach and oversee if participants self-administer study drug at a scheduled visit.
Dispense ancillary supplies to participant		X	X	X	X	X	X	X		X				X						
Participant returns study drugs and injection supplies			X	X	X	X	X	X		X		X		X				X	X	

Abbreviations: 6MWT = 6-minute walk test; BP = blood pressure; CKD-EPI = Chronic Kidney Disease Epidemiology Collaboration; CVD = cardiovascular disease; ECG = electrocardiogram; ECHO = echocardiography; eCRF = electronic case report form; eGFR = estimated glomerular filtration rate; EQ-5D-5L = 5-Level European Quality of Life Questionnaire; ET = Early Termination; EV = Extended Visit; HF = heart failure; HFpEF = heart failure with preserved ejection fraction; HR = heart rate; HTN = hypertension; MI = myocardial infarction; NT-proBNP = N-terminal pro b-type natriuretic peptide; NYHA = New York Heart Association; T2DM = type 2 diabetes mellitus; WOCBP = women of childbearing potential.

[a] There are 3 types of Unscheduled Visits (UV): These visits can occur during the dose-escalation period and maintenance period.

- Unscheduled Visits (UV) – Unscheduled Visit per investigator discretion. All activities with a check mark in UV visit are required.
- Dosing Unscheduled Visit (UV) – these visits will include dose re-escalation. The study drug must be restarted when it is safe to do so and only while at the clinic visit. The subsequent unscheduled dosing visits will be scheduled every 4 weeks (±7 days) until maximum tolerated dose is achieved.
- Phone Follow-up Dosing Unscheduled Visit (UV) – these visits are optional phone visits.

[b] At least 52 weeks (Visit 12) of treatment are planned. Additional visits after Visit 12 will occur every 3 months. The visits occurring after Visit 12 will follow the Extended Maintenance Visit schedule in sequence (EVa, EVb, EVc, and EVd) then repeat. If the final study visit is combined, site should only use the visit 12 lab kit; please refer to Study Closeout and Final Visit in Section 4.1.

[c] Telephone visits can become office visits. Site documentation will serve as the source for telephone visits. Additional, optional telephone visits may be conducted at investigator discretion. If patient requires study drug at a telephone visit, the participant will need to come to the clinic to pick up study drug in addition to telephone visit.

[d] Perform at ET only if participant early terminates at or prior to Week 52.

[e] Two 6MWTs conducted at screening visit.

[f] See Section 10.10.1 to determine if the 6MWT needs to be repeated for Visit 2.

[g] This includes glucose monitoring for participants with T2D, weekly study drug injection.

[h] Perform PRO at ET only if participant early terminates at or prior to Week 52.

[i] Unscheduled Visits: laboratory tests may be drawn according to investigator discretion. If only re-test labs required, site is not required to complete an unscheduled visit and associated procedures.

[j] Required at Visit 2 randomization if screening labs are older than 28 days for chemistry, lipid, and pancreatic lipase/amylase.

[k] Collect serum pregnancy at Visit 2 only if Visit 1 serum was ≥28 days prior.

[l] Only collect calculated eGFR with Clinical Chemistry sample if screening labs are older than 28 days.

2. Introduction

2.1. Study Rationale

Heart failure with preserved ejection fraction is a heterogenous clinical syndrome resulting from various pathophysiological processes. Among the broad spectrum of HFpEF clinical presentation, obesity-related HFpEF displays a distinct phenotype where increased visceral and ectopic adiposity as well as volume expansion plays a causal role (Kitzman and Shah 2016; Packer 2018; Miller and Borlaug 2020). Given tirzepatide's potential to decrease inflammation and fibrosis and a to reduce circulating plasma volume as a consequence of the treatment of obesity, tirzepatide may provide clinical benefit to patients with HFpEF and BMI ≥30 kg/m^2.

Study I8F-MC-GPID, also known as SUMMIT, is a Phase 3, randomized, multicenter, international, placebo-controlled, double-blinded, parallel-arm study. This study will evaluate the effect of SC QW injection of tirzepatide, MTD up to 15 mg, on the health status, risk of death, HF events, and exercise capacity in participants with HFpEF and BMI ≥30 kg/m^2.

2.2. Background

Obesity is one of the main attributes to worsen quality of life in patients with HFpEF (Reddy et al. 2020). There is a significant unmet need in treatment of patients with HFpEF. Tirzepatide, a GIP and GLP-1 dual agonist, has the potential to provide benefit to patients with HFpEF and BMI ≥30 kg/m^2. Tirzepatide may improve symptoms and exercise capacity and may also a reduce in HF events and/or increase survival. Supporting a causal association between obesity and HFpEF, bariatric surgery improved NYHA class, patient-reported outcomes, and echo parameters (LV wall thickness, LV relaxation) in patients with HFpEF and obesity (Mikhalkova et al. 2018). A meta-analysis of bariatric surgery also showed improvement in functional capacity 6 to 12 months after surgery in patients with obesity (Herring et al. 2016). In patients with HFpEF and obesity, diet-induced weight loss (Δ=−7 kg, 20 weeks) significantly improved symptoms (KCCQ) and exercise capacity (6MWD and peak oxygen uptake) (Kitzman et al. 2016). Furthermore, weight reduction is proven to be effective in reducing HF risk and HF hospitalizations. A large observational study has demonstrated a 62% decrease (over 8 years) of HF incidence after bariatric surgery in patients with T2DM (Aminian et al. 2019). The reduction of HF risk after bariatric surgery has been consistently demonstrated in broader patient populations and considered to be mediated by weight loss with a hazard ratio for a 10-kg weight loss being 0.77 (Sundström et al. 2017; Jamaly et al. 2019). Moreover, a self-controlled case study showed a 29% (0 to 12 months) and a 43% (13 to 24 months) risk reduction of HF events in patients with HFpEF after bariatric surgery (Shimada et al. 2016). Finally, in a recently published placebo-controlled study (STEP-HFpEF), semaglutide reduced body weight by 10.7% and improved KCCQ-CSS by 7.8 points at 52 weeks, both with $p<0.001$ (Kosiborod et al. 2023; Borlaug et al. 2023). In addition, superiority on the hierarchical composite endpoint (death, HF events, differences in the change in KCCQ-CSS, and 6MWD) was achieved, including the proportion of patients who had improved KCCQ score by at least 15 points in the semaglutide group. Semaglutide also improved 6MWD, with between-group difference of 20.3 meters. Most intriguingly, adjudicated events of hospitalization for heart failure or an urgent visit occurred in

12 patients in the placebo group, but only in 1 patient in the semaglutide group (HR 0.08; 95% CI 0.00 to 0.42)

It has been demonstrated that tirzepatide can provide significant body weight loss and improvement of lipid and glucose metabolism in patients with and without T2DM (Frias et al. 2018; Wilson et al. 2020; Jastreboff et al. 2022). It is known that the body weight reduction with GLP-1 RAs in patients without T2DM is higher than in patients with T2DM (Davies et al. 2015; Pi-Sunyer et al. 2015; Lingvay et al. 2018). If Study GPID is assumed to include 40% to 50% of patients with T2DM, the mean placebo-adjusted body weight percent reduction that tirzepatide can provide in 52 weeks in this study is estimated to be 15% to 16%. This is based on tirzepatide clinical data and the understanding of body weight loss differences between patients with and without T2DM treated with GLP-1 RAs. Thus, the predicted body weight loss with treatment with tirzepatide is close to that shown with bariatric surgery.

Tirzepatide may provide benefit to patients with HFpEF and obesity by virtue of cardiometabolic improvements (Wilson et al. 2020). Given the wide distribution of GIP receptor in the adipose tissue, GIP is thought to be actively involved in lipid and glucose metabolism. As suggested by the results of emerging data (Kosiborod et al. 2023) and the significant weight loss achieved with tirzepatide, the effect of tirzepatide on HF events in an obesity-related HFpEF population are expected to be robust. Therefore, it will be meaningful to assess the impact of tirzepatide not only on functional and symptomatic endpoints but also on the reduction in the risk of HF events in SUMMIT, thereby facilitating the holistic understanding of the clinical impact of tirzepatide treatment in patients with HFpEF and obesity.

2.3. Benefit/Risk Assessment

More detailed information about the known and expected benefits and risks and reasonably expected AEs of tirzepatide may be found in the Investigator’s Brochure.

3. Objectives and Endpoints

Objectives	Endpoints
Primary	
To demonstrate that a maximally tolerated tirzepatide dose up to 15 mg administered SC QW is superior to placebo to improve patient-reported symptoms and physical limitations in participants with HFpEF and obesity	Change from baseline to Week 52 in the KCCQ-CSS
To demonstrate that a maximally tolerated tirzepatide dose up to 15 mg administered SC QW is superior to placebo based on the composite HF outcome endpoint in participants with HFpEF and obesity	Occurrence of the composite endpoint of CV death and/or HF events over time
Key Secondary (multiplicity controlled)	
Exercise capacity	Change from baseline to Week 52 in 6MWD
Long-term weight loss	Percent change from baseline to Week 52 in body weight
Evaluation of change in inflammation	Change from baseline to Week 52 in hsCRP
Other Secondary	
Hierarchical composite assessed by win ratio	A hierarchical composite of the following:<br>1. Time to all-cause mortality through the end of the study<br>2. Occurrence of heart failure (HF) events through end of the study, where HF events are defined as worsening heart failure with intensification of diuretics (oral or IV) during a hospitalization, urgent care visit or outpatient visit (adjudicated)<br>• number of HF events<br>• time to first HF events<br>3. Change from baseline in KCCQ-CSS category at Week 52

	4. Change from baseline in the 6-minute walk test distance (6MWD) category at Week 52<br><br>The categories for change from baseline in the KCCQ-CSS are:<br><br>1. ≥10-point worsening<br>2. ≥5- but <10-point worsening<br>3. No change (<5-point change)<br>4. ≥5- but <10-point improvement<br>5. ≥10- but <15-point improvement<br>6. ≥15-point improvement<br><br>The categories for change from baseline in the 6MWD are:<br><br>1. ≥30% worsening<br>2. ≥20% and <30% worsening<br>3. ≥10% and <20% worsening<br>4. No change (<10% change)<br>5. ≥10% and <20% improvement<br>6. ≥20% and <30% improvement<br>7. ≥30% improvement.
Clinical outcome events of HF	• Time to all-cause death<br>• Time to first occurrence of HF events or all-cause death<br>• Time to recurrent events of HF events and all-cause death<br>• Time to first occurrence of HF events<br>• Time to recurrent events of HF events
New York Heart Association (NYHA) Class	Proportion of participants with NYHA Class change at Week 52
Exercise capacity	Change from baseline to Week 24 in 6MWD
Patient-reported symptoms and physical limitations	• Change from baseline to Week 24 in KCCQ-CSS<br>• Proportion of participants attaining KCCQ-CSS meaningful within-patient change (MWPC) threshold at Week 52
Exploratory	

Atrial fibrillation	Proportion of participants with atrial fibrillation
Waist circumference	Change from baseline (centimeters)
Patient-reported health-related quality of life	Change from baseline in KCCQ:<br>• Total Symptom Score (TSS)<br>• Overall Summary Score (OSS)
Patient-reported health status	Change from baseline in EQ-5D-5L:<br>o Index Score<br>o VAS Score
Patient-reported global health status	Proportion of participants with improvements in global health status from baseline as assessed by the PGIS-Overall
Patient-reported global impression of physical function	Proportion of participants with improvements in physical function from baseline as assessed by the PGIS-Physical Function
Patient-reported global symptom severity	Proportion of participants with improvements in symptom severity from baseline as assessed by the PGIS-Symptom Severity
Evaluation of prespecified biomarkers	• NT-proBNP<br>• cTnT
Waist to height ratio	Change from baseline to Week 52 in waist to height ratio
Kidney function	eGFR slope

Abbreviations: 6MWD = 6-minute walk distance; BMI = body mass index; CV = cardiovascular; hsCRP = high-sensitivity C-reactive protein; HF = heart failure; HFE(s) = heart failure event(s); HFpEF = heart failure with preserved ejection fraction; KCCQ-CSS = Kansas City Cardiomyopathy Questionnaire – Clinical Summary Score; NYHA = New York Heart Association; QW = once weekly; SC = subcutaneous.

4. Study Design

4.1. Overall Design

Study GPID is a randomized, outpatient, multicenter, international, placebo-controlled, double-blinded, parallel-arm, Phase 3 study with 2 study periods. The study is designed to evaluate the efficacy and safety of SC QW tirzepatide, MTD up to 15 mg, compared to placebo, in participants with HFpEF and obesity.

Two intervention groups will be studied:

- Tirzepatide MTD up to 15 mg SC QW
- Placebo

The study will compare treatment with tirzepatide and treatment with placebo. Assignment to tirzepatide or placebo groups will be randomly allocated in a 1:1 ratio.

The starting dose of tirzepatide or placebo is 2.5 mg QW, which is to be escalated at 4-week intervals to a maximum of 15 mg QW or to the highest maintenance dose tolerated by the participant (see Section 6).

The study will consist of 2 periods:

- Study Period 1: screening period, lasting no more than 12 weeks.
- Study Period 2: treatment period, with a 20-week escalation followed by at least a 32-week maintenance period.

The study will continue until approximately 52 weeks after the last participant is randomized. The maximum duration of an individual's participation is estimated to be 120 weeks and will depend on duration of study enrollment.

Participant Visit Scheme

Study participants will undergo screening assessments and procedures, randomization, and double-blinded treatment with tirzepatide or placebo. Assessments and procedures to be conducted in each treatment period are described in the SoA (Section 1.3) and in Study Assessments and Procedures (Section 8).

<u>Screening</u>

Screening procedures will be performed at Visit 1. Visit 1 procedures may be conducted over more than 1 day as long as all activities are completed within the allowable visit tolerance for each visit. The duration of Visit 1 is anticipated to be <6 weeks but may be longer. Visit 1 should no more than 12 weeks from date of informed consent.

At Visit 1, two 6MWTs will be conducted. The investigator must ensure that the participant is recovered from completion of the first 6MWT prior to conducting the second 6MWT (at least 1 hour between each test). The screening 6MWTs may be conducted over more than 1 day.

Randomization

At Visit 2, prior to randomization, the participant needs to complete the 6MWT. Visit 2 may need to be conducted over 2 in-clinic visits (considered Visit 2a and Visit 2b) if a repeat 6MWT is necessary to assess participant eligibility. If the participant is required to return to repeat the 6MWT, the remaining procedures should be conducted at the second in-clinic visit for randomization (Visit 2b), more than 10 days from Visit 2a. See Section 10.10.1 for details on when a participant must return for a second Visit 2 6MWT.

Participants will be randomized and receive blinded study drug at the end of Visit 2 after all screening procedures are completed. The participant must not receive study drug until all eligibility criteria, including the 6MWT, are met.

Treatment

Starting from randomization, the participant receives study drug and procedures are conducted as described in the SoA (Section 1.3). Every effort should be made by the investigator to maintain participants on study drug.

Study drug dose will be escalated as illustrated in the study schema (Section 1.2). Dose escalation will continue until the participant reaches the maintenance dose of either 5 mg, 10 mg, or 15 mg. Participants who do not tolerate the first dose escalation (that is, from 2.5 mg to 5 mg [or placebo equivalent]) will need to discontinue from study drug.

Participants begin treatment with either tirzepatide or placebo starting at 2.5 mg given as a subcutaneous (SC) injection every week (QW). The dose level is increased by 2.5 mg increments approximately every 4 weeks for the first 20 weeks according to the participant's tolerability, reaching a MTD up to 15 mg. Once a MTD has been achieved, participants will continue at this MTD until study end, treatment discontinuation, or study discontinuation. Dose modifications will be permitted during the study under the circumstances specified in Section 6.6.

During the study treatment period, if an unscheduled visit or telephone visit is deemed necessary to support participant compliance, this is allowable at the discretion of the investigator site personnel. Optional telephone visits can be performed approximately 2 weeks after starting study drug and after each dose increase.

Participants will continue into the extended maintenance period with the same treatment assignment starting with Visit 13. Extended visits (EV) continue until criteria for study discontinuation is met or study ends (see Study Closeout and Final Visit below).

Study Closeout and Final Visit

The study will continue until approximately 52 weeks after the final participant is randomized The final study visit (Visit 99) for the study is based from the date of when the last patient for the trial was randomized.

Approximately 3 months prior to the anticipated end of study, the sponsor will notify sites of upcoming study closeout based on projected last patient visit date of the last patient randomized to the study. During the study closeout, a final visit (Visit 99) will be planned for each participant, with the exception of those who have died or prematurely discontinued from the study (Section 7.2). Any participant that has completed at least 52 weeks of study duration should be scheduled to complete the final visit (Visit 99) during this 3-month period prior to the

study final visit. No additional investigational product will be dispensed at this final visit. Study procedures for the final visit will be performed as outlined in the SoA (Section 1.3). Subjects completing 52 weeks of treatment during the last 3 months of the study will have a combined Visit 12 (52-week treatment visit) and Visit 99 (final visit) on the same day. During this combined visit, sites should only use the Visit 12 lab kit for blood draws and processing.

All unused study drug (unused single-dose pens) must be returned for compliance and final drug accountability. The sharp items container should also be returned to site or disposed of per local regulations.

Any participant who has discontinued the study prior to completing 52 weeks of study duration is expected to complete an early termination visit per the SoA.

4.2. Scientific Rationale for Study Design

Study GPID is a Phase 3 study designed to examine the efficacy and safety of SC QW tirzepatide MTD compared with placebo in participants with HFpEF and BMI ≥30 kg/m^2.

A placebo comparator was selected for this trial in accordance with regulatory guidance (FDA 2007; EMA 2016). Inclusion of a placebo comparator in Study GPID will allow for a direct assessment of the safety and efficacy of tirzepatide in participants with HFpEF and obesity.

Additionally, there is currently no approved therapy to be used as an active comparator in this population.

An endpoint assessment at 52 weeks of treatment is considered appropriate to assess the improvement of symptom and functional capacity. An extended maintenance treatment period increases the opportunity to evaluate HF events and outcomes.

The parallel-group design for treatment comparison was chosen to avoid any interaction between treatments that may interfere with the interpretation of the trial outcome. To minimize potential confounding effect of changes to concomitant medications, participants will be permitted to use the stable dose of concomitant medications that they require during the study. Medications that may interfere with the assessment of efficacy and safety characteristics of the study drug will not be allowed (see Section 6.5).

Assessment of HF events is relevant to HFpEF, which is characterized by a high frequency of recurrent HF hospitalizations. Moreover, hospitalization events reflect disease progression and high subsequent risk and predisposition, both of readmission and death (Solomon et al. 2007). Recent studies have shown that outpatient oral diuretic intensification in ambulatory care carries similar risk as an urgent HF visit and is independently associated with subsequent cardiovascular events, including death (Chatur et al. 2023; Ferreira et al. 2022; Madelaire et al. 2020).

Obesity is associated with important degrees of exercise intolerance and a markedly impaired quality of life and health status (Reddy et al. 2020). In patients with HFpEF, due to increased filling pressures, functional capacity is severely impaired, and patients can develop symptoms with light exercise. As a result, the ability to perform activities of daily living is deteriorated. Therefore, KCCQ is a meaningful endpoint to evaluate the clinical benefit of tirzepatide in patients with HFpEF and obesity.

Given the significant weight loss, and associated cardiometabolic improvements, achieved with tirzepatide, assessment of CV death and HF events, in addition to KCCQ-CSS as a primary endpoint, offers the unique opportunity to evaluate tirzepatide for the benefit of HFpEF patients with obesity.

4.2.1. Patient Input into Design

The sponsor involved patients in the design of this study by engaging patients in virtual collaborative events. The insights gained from these events were used to ensure that the study design is supportive of the well-being of the study participants and that the study procedures can be implemented effectively at the investigative sites.

4.3. Justification for Dose

Tirzepatide doses of up to 15 mg administered SC QW will be evaluated in this study.

Participants may be treated with lower maintenance doses of 5 mg or 10 mg if they do not achieve full dose escalation to 15 mg and/or do not tolerate 15 mg.

These doses and associated escalation schemes were selected based on assessment of safety, efficacy (glycemic and weight loss benefit), and GI tolerability data followed by exposure response modeling of data in participants with T2DM in Phase 1 and Phase 2 studies. Dosing algorithms starting at a low dose of 2.5 mg accompanied by dose escalation of 2.5 mg increments every 4 weeks should permit time for development of tolerance to GI events and are predicted to minimize GI tolerability concerns.

The dose selection of tirzepatide is based on the findings of the Phase 2 study results. Tirzepatide doses of 5 mg, 10 mg, and 15 mg QW have been tested and compared with dulaglutide 1.5 mg QW or placebo in a Phase 2 study (Frias et al. 2018). While all 3 doses of tirzepatide significantly improved the glycemic control versus dulaglutide, the largest difference was observed in the 15-mg tirzepatide treatment group. Moreover, the reduction in body weight with tirzepatide was also dose-dependent and greatest in the 15 mg QW treatment group.

4.4. End of Study Definition

The end of study will occur approximately 52 weeks after the last participant has been randomized to the study globally. The study end will occur based on this definition and is not impacted by the status of the last randomized participant.

The criteria used to determine if a participant has completed the study will be described in the SAP.

5. Study Population

Prospective approval of protocol deviations to recruitment and enrollment criteria, also known as protocol waivers or exemptions, is not permitted.

5.1. Inclusion Criteria

Participants are eligible to be included in the study only if all of the following criteria apply:

Age

1. Participant must be at least 40 years of age inclusive, at the time of signing the ICF.

Type of Participant and Disease Characteristics

2. 6MWD ≤465 meters at both Visit 1 tests, between ≥100 meters and ≤425 meters at Visit 2 and change from the preceding qualifying 6MWD is <20% and <40 meters. See Section 10.10.1 for the flow diagram of the below qualifiers.
   - *If* Visit 2a 6MWD is both between 100 and 425 meters and…
     - <20% AND <40 meter change from the higher of the two 6MWDs conducted at Visit 1, *then* participant meets this inclusion criterion.
     - ≥20% OR ≥40 meter change from the higher of the two 6MWDs at Visit 1, *then* participant must attend Visit 2b. If at Visit 2b, the 6MWD is between 100 and 425 meters and <20% AND <40 meter change from preceding (Visit 2a) 6MWD, then participant meets this inclusion criterion.
3. Chronic HF (NYHA Class II-IV) diagnosed for at least 3 months before Visit 1.
4. LVEF ≥50% demonstrated by echocardiogram performed at Visit 1 or within 6 months of Visit 1.
5. At least 1 of the following to document evidence of HF:
   - Elevated NT-proBNP >200 pg/mL for participants without atrial AF or >600 pg/mL for participants with AF, as analyzed at the central laboratory at Visit 1
     OR
   - Evidence of structural heart disease:
     - LA enlargement (any of the following: LAV index ≥29 mL/m^2, or LAV >58 mL in male participants and >52 mL in female participants, or LA area >20 cm^2, or LA diameter >40 mm in male and >38 mm in female participants) determined by echocardiogram at Visit 1 or within 6 months of Visit 1

     OR
   - Evidence of elevated filling pressure:
     - At rest (PCWP ≥15 mmHg or LVEDP ≥15 mmHg) or with exercise (PCWP ≥25 mmHg) (based on historical record, not associated with hospitalization for decompensation of HF, within 2 years of Visit 1), or
     - E/e' ratio >15 (septal) or >13 (average of septal and lateral) determined by echocardiogram at Visit 1 or within 6 months of Visit 1

Note: Supporting medical documentation is required in all instances.

6. Either one of:
   - eGFR <70 mL/min/1.73 m^2 at Visit 1, OR
   - HF decompensation within 12 months of Visit 1, defined as hospitalization for HF requiring IV diuretic treatment or urgent HF visit requiring IV diuretic treatment
     Note: Supporting medical documentation is required in all instances.
7. Stable dose of all concomitant HF medications (these may include beta blockers, ACEis, ARBs, MRAs, ARNI, and/or SGLT2is), except for oral diuretics, for at least 4 weeks prior to Visit 1 and throughout the screening period.
8. If treated with oral diuretics, dose must be stable for at least 2 weeks prior to Visit 1 and throughout the screening period; volume control must be optimally achieved in the opinion of the investigator.
9. KCCQ-CSS ≤80 at Visit 1.

Weight

10. BMI ≥30.0 kg/m^2 at Visit 1.

Sex

11. At the time of signing the ICF:
   a. **Male participants**: Male participants with partners of childbearing potential should be willing to use reliable contraceptive methods for the duration of the trial and for 4 months thereafter (see Appendix 4 [Section 10.4]).
   b. Female participants:
   - Female participants not of childbearing potential may participate and include those who are infertile due to surgical sterilization and/or postmenopausal. Please refer to Appendix 4 (Section 10.4) for definitions.
   - Female participants of childbearing potential (not surgically sterilized and between menarche and 1-year postmenopausal) must:
     - test negative for pregnancy at Visit 1 based on a serum pregnancy test followed by a negative urine pregnancy test within 24 hours prior to exposure and agree to use 2 forms of effective contraception, if sexually active, where at least 1 form is highly effective, for the duration of the trial and for 2 months after the last injection, and
     - not be breastfeeding.

Contraceptive use by men or women of childbearing potential should be consistent with local regulations regarding the methods of contraception for those participating in clinical trials. See Appendix 4 (Section 10.4) for guidance.

Informed Consent

12. Capable of giving signed informed consent as described in Appendix 1 (Section 10.1), which includes compliance with the requirements and restrictions listed in the ICF and in this protocol.

5.2. Exclusion Criteria

Participants are excluded from the study if any of the following criteria apply:

Medical Conditions

13. Myocardial infarction, coronary artery bypass graft surgery, or other major CV surgery/intervention, stroke or transient ischemic attack in past 90 days, or unstable angina pectoris in past 30 days, prior to Visit 1 or during screening; Have NYHA Class I heart failure at either Visit 1 or Visit 2.

14. Dominant contribution of noncardiac causes to exercise impairment or symptoms
    - Lung disease: pulmonary arterial hypertension, chronic thromboembolic pulmonary hypertension (CTEPH), or severe pulmonary disease including (COPD)
    - Other medical conditions: severe anemia (hemoglobin level <9 g/dL) at Visit 1, untreated thyroid disease or TSH >4.78 mU/L at Visit 1, or significant musculoskeletal disease
    - Orthopedic conditions that limit the ability to walk, such as severe arthritis in the leg, knee, hip injuries, hemiplegia, or amputation with artificial limb without stable prosthesis function for the past 3 months
    - Any condition that in the opinion of the investigator would interfere with the assessment of 6MWT

15. LVEF <40% by local echocardiography, MRI or other modalities documented any time within 2 years of Visit 1.
16. Acute decompensated HF (exacerbation of HF) requiring IV diuretics, IV inotropes, or IV vasodilators, or left ventricular assist device (LVAD) within 4 weeks prior to Visit 1, and/or during the screening period until randomization.
17. Impaired renal function, defined as eGFR <15 mL/min/1.73 m^2 (CKD-EPI) or requiring dialysis at Visit 1.
18. Any one of the following:
    - Systolic blood pressure (SBP) ≥180 mmHg at Visit 1
    - SBP >160 mmHg both at Visit 1 and at Visit 2
    - Have symptomatic hypotension or SBP <100 mmHg at Visit 1 or Visit 2
19. Resting heart rate (sinus rhythm) ≥100 bpm at either Visit 1 or Visit 2.
20. Atrial fibrillation or atrial flutter with a resting heart rate >110 bpm documented by ECG at either Visit 1 or Visit 2.
21. Cardiac amyloidosis or cardiomyopathy based on accumulation disease (for example, haemochromatosis, Fabry disease), muscular dystrophy, cardiomyopathy with reversible causes (for example, stress cardiomyopathy), hypertrophic cardiomyopathy, Chagas cardiomyopathy or known pericardial constriction, or any severe (obstructive or regurgitant) valvular heart disease likely to lead to surgery during the study period.
22. Completed prior surgical treatment for obesity or had liposuction or abdominoplasty within 1 year prior to Visit 1. Participants who plan to have surgical treatment for obesity or liposuction or abdominoplasty during the duration of the study are excluded.

23. Participation in a structured exercise training program in the 1 month prior to Visit 1 or planning to start a program during the study.
24. Have T1DM.
25. For participants with T2DM:
- Have uncontrolled diabetes requiring immediate therapy (such as diabetic ketoacidosis) at Visit 1 or Visit 2, in the judgement of the physician
- Have had 1 or more events of severe hypoglycemia and/or 1 or more events of hypoglycemia unawareness within 6 months prior to Visit 1 (see Section 10.5.1.1 for definition of hypoglycemia)
- Have HbA1c ≥9.5% (80 mmol/mol) at Visit 1, as analyzed at the central laboratory
- Have a history of proliferative diabetic retinopathy or diabetic maculopathy. Patients with severe nonproliferative diabetic retinopathy that requires acute treatment are also excluded.
- Treated with premix or prandial insulins or intensified insulin regimens (multiple daily injection with basal and prandial insulins or insulin pump therapy) at Visit 1.

26. History of acute or chronic pancreatitis or at high risk for acute pancreatitis (for example, serum triglyceride level >500 mg/dL [5.65 mmol/L]).
27. Have acute or chronic hepatitis, signs and symptoms of any other liver disease other than nonalcoholic fatty liver disease, or any of the following, as determined by the central laboratory during Visit 1:
- ALT or AST levels >2.5X the ULN for the reference range.

Note: Participants with nonalcoholic fatty liver disease are eligible to participate in this trial if their ALT level is ≤3.0X the ULN for the reference range.

28. Have a calcitonin level at Visit 1 of:
    - ≥20 ng/L, if eGFR is ≥60 mL/min/1.73 m^2
    - ≥35 ng/L, if eGFR is <60 mL/min/1.73 m^2
29. Have a family or personal history of medullary thyroid carcinoma (MTC) or Multiple Endocrine Neoplasia (MEN) Syndrome type 2.
30. Have a history of an active or untreated malignancy or are in remission from a clinically significant malignancy (other than basal- or squamous-cell skin cancer, in situ carcinoma of the cervix, or in situ prostate cancer) for less than 5 years.
31. Have a history of any other condition (such as known drug or alcohol abuse, diagnosed eating disorder, or other psychiatric disorder) that, in the opinion of the investigator, may preclude the participant from following and completing the protocol.
32. Have a known clinically significant gastric emptying abnormality (for example, severe diabetic gastroparesis or gastric outlet obstruction) or chronically take drugs that directly affect GI motility.

Prior/Concomitant Therapy

33. Treatment with any incretin, GLP-1 RA, or pramlintide in the 3 months prior to Visit 1.

34. Discontinuation of any incretin, GLP-1 RA, or pramlintide due to intolerability any time prior to Visit 1.
35. Have any other condition not listed in this section (for example, hypersensitivity or intolerance) that is a contraindication to GLP-1 RA.
36. Implantable cardioverter defibrillator (ICD) implantation within 1 month prior to Visit 1 or planned implantation during the course of the study.
37. Currently implanted left ventricular assist device (LVAD).
38. Cardiac resynchronization therapy (CRT) implanted within 6 months prior to Visit 1 or planned implantation during the course of the trial.
39. Current use of medication associated with weight gain or weight loss, except when on stable dose for at least 3 months prior to Visit 1, and expected to be stable during the study period.

Prior/Concurrent Clinical Study Experience

40. Have participated within the last 6 months in a clinical study involving an investigational product.

Other Exclusions

41. Investigator site personnel directly affiliated with this study and/or their immediate families. Immediate family is defined as a spouse, parent, child, or sibling, whether biological or legally adopted.
42. Lilly employees.

5.3. Lifestyle Considerations

Study participants should be instructed not to donate blood or blood products during the study and for 8 weeks following the study.

5.4. Screen Failures

Screen failures are defined as participants who consent to participate in the clinical study but are not subsequently randomly assigned to study drug. A minimal set of screen failure information is required to ensure transparent reporting of screen failure participants to meet the CONSORT publishing requirements and to respond to queries from regulatory authorities. Minimal information includes demography, screen failure details, eligibility criteria, and any SAE.

Individuals who do not meet certain criteria for participation in this study (screen failure) may be rescreened once. Additionally, individuals who do not complete screening within 12 weeks from ICF date should be screen failed and may be rescreened. Rescreened participants should sign a new ICF and be assigned a new participant number. The interval between rescreenings should be at least 2 weeks. For participants who may have screen failed due to HbA1c criterion not met, the time to permit rescreening is at least 8 weeks. If, in the opinion of the investigator, an ineligible laboratory test result is the result of an error or extenuating circumstance, then that parameter can be retested once without the participant having to be rescreened. For rescreened participants, a repeat echocardiogram is not permitted.

Participants may be rescreened for the following reasons:

- Have become eligible to enroll in the study as the result of a protocol amendment
- Status has changed such that the eligibility criterion that caused the participant to screen fail would not cause the participant to screen fail again
- Completed screening and met all inclusion and exclusion requirements but are unable to be enrolled due to extenuating circumstances (such as severe weather, death in family, or child illness)

6. Study Intervention

Study drug is defined as any investigational intervention(s), marketed product(s), placebo, or medical device(s) intended to be administered to/used by a study participant according to the study protocol. For this study, 'study intervention' and 'study drug' are equivalent.

6.1. Study Interventions Administered

**Intervention Name**	Placebo	Tirzepatide (LY3298176)
**Type**	Drug (placebo)	Drug
**Dose Formulation**	Single-dose pen	Single-dose pen
**Unit Dose Strengths**	Not applicable	2.5 mg, 5 mg, 7.5 mg, 10 mg, 12.5 mg, 15 mg
**Dosage Levels**	Not applicable	15 mg QW<br>(or maximum tolerated dose of 5 mg QW or 10 mg QW)
**Route of Administration**	Subcutaneous	Subcutaneous
**Use**	Placebo	Experimental
**IMP and NIMP**	IMP	IMP
**Sourcing**	Provided centrally by the sponsor and dispensed via IWRS	
**Packaging and Labeling**	Study drug will be provided in autoinjectors (single-dose pens), packaged in cartons to be dispensed.<br>Clinical study materials will be labeled according to country regulatory requirements.	

Abbreviations: QW = weekly; IMP = investigational medicinal product; IWRS = interactive web-response system; NIMP = non-investigational medicinal product.

The following table shows the randomized study drugs for the entire study.

Treatment Group	Treatment Period Interval					
	Weeks 0 to 3	Weeks 4 to 7	Weeks 8 to 11	Weeks 12 to 15	Weeks 16 to 19	Weeks 20 to End of Treatment Period
Tirzepatide	2.5 mg	5 mg	7.5 mg	10 mg	12.5 mg	15 mg or MTD
Placebo	→					

Abbreviation: MTD = maximum tolerated dose.

There are no restrictions on the time of day each weekly dose of study drug is given, but it is advisable to administer the SC injections on the same day and same time each week. The actual date, time, and injection site location of all dose administrations will be recorded in the diary by the participant. If a dose of study drug is missed, the participant should take it as soon as possible, unless it is within 72 hours of the next dose, in which case that dose should be skipped, and the next dose should be taken at the appropriate time. The day of weekly administration can be changed if necessary, as long as the last dose was administered 72 or more hours previously.

All participants will inject study drug subcutaneously in the abdomen or thigh using the injection supplies provided; a caregiver may also administer the injection in the participant's upper arm. The injection site location of all dose administrations will be recorded by the participant. A new autoinjector will be used for each injection. If study drug is to always be injected in the same body region, participants should be advised to rotate injection sites each week.

6.1.1. Medical Devices

The combination product provided for use in the study is a tirzepatide or matching placebo autoinjector.

6.2. Preparation/Handling/Storage/Accountability

- The investigator or designee must confirm appropriate storage conditions have been maintained during transit for all study drug received and any discrepancies are reported and resolved before use of the study drug.
- Only participants enrolled in the study may receive study drug. Only study personnel may supply study drug.
- All study drug must be stored in a secure, environmentally controlled, and monitored (manual or automated) area in accordance with the labeled storage conditions with access limited to the investigator and authorized study personnel.
- The investigator, institution, or the head of the medical institution (where applicable) is responsible for study drug (includes study drug and autoinjector or single-dose pen) accountability, reconciliation, and record maintenance (that is, receipt, reconciliation, and final disposition of records).
- Study site staff must regularly assess whether the participant is correctly administering the assigned study drug and storing study drug according to the provided instructions.

Further guidance and information for the final disposition of unused study drugs are provided in the study training materials.

The investigator or designee is responsible for the following:

- Explaining the correct use of the study drug to the participant
- Verifying that instructions are followed properly
- Maintaining accurate records of study drug dispensing and collection as well as records of interruptions in study drug administration
- Instructing the participant to discard all used autoinjectors for study drug in a closeable, puncture-resistant container and dispose according to local regulations, and
- Considering dose adjustment of antihyperglycemic medications (see Section 6.5) at Visit 2 from first administration of study drug.

6.3. Measures to Minimize Bias: Randomization and Blinding

This is a double-blind study.

Participants who meet all criteria for enrollment will be randomized to one of the study treatment groups at the end of Visit 2. Assignment to treatment groups will be determined by a computer-generated, random sequence using an IWRS. Participants will be randomized in a 1:1 ratio to receive tirzepatide or placebo. The randomization will be stratified by HF decompensation (hospitalization for HF requiring IV diuretic treatment or urgent HF visit requiring IV diuretic treatment) within 12 months of screening (Y/N), diagnosed T2DM (Y/N), and BMI ≥35 kg/m^2 (Y/N).

Before the study is initiated, the log in information and directions for the IWRS will be provided to each site.

Study drug will be dispensed at the study visits shown in the SoA.

Returned unused study drug should not be re-dispensed to the participants.

The IWRS will be programmed with blind-breaking instructions. In case of an emergency, the investigator has the sole responsibility for determining if unblinding of a participant's drug assignment is warranted. Participant safety must always be the first consideration in making such a determination. If a participant's drug assignment is unblinded, the sponsor must be notified immediately after breaking the blind. The date and reason that the blind was broken must be recorded in the source documentation and CRF, as applicable.

If an investigator, site personnel performing assessments, or participant is unblinded, the participant must be discontinued from the study. In cases where there are ethical reasons to have the participant remain in the study, the investigator must obtain specific approval from a sponsor CRP for the participant to continue in the study.

6.4. Study Intervention Compliance

Participant compliance with study drug and adherence to study visits and procedures will be vitally important to meet the study objectives. This will be emphasized through comprehensive site training and consistently monitoring participant retention throughout the duration of the study.

Study drug compliance will be determined by the following:

- Study drug administration data will be recorded by the participants in the participant study diary and reviewed by the investigator at each study visit.
- The participants will be instructed to return any unused study drug and/or empty cartons at the next visit to the study site for the purpose of performing drug accountability.

Treatment compliance is defined as taking at least 75% of the required doses of study drug. Similarly, a participant will be considered significantly noncompliant if he or she is judged by the investigator to have intentionally or repeatedly taken more than the prescribed amount of medication (more than 125%).

In addition to the assessment of a participant's compliance with the study drug administration, other aspects of compliance with the study drug will be assessed at each visit based on the participant's adherence to the visit schedule, completion of study diaries, and any other parameters the investigator considers necessary.

Participants considered to be poorly compliant with their medication and/or the study procedures will receive additional training and instruction, as required, and will be reminded of the importance of complying with the protocol.

6.5. Concomitant Therapy

Any medication or vaccine (including over-the-counter or prescription medicines, vitamins, and/or herbal supplements) or other specific categories of interest that the participant is receiving at the time of enrollment or receives during the study must be recorded along with:

- Reason for use
- Dates of administration including start and end dates
- Dosage information including dose and frequency for concomitant therapy of special interest (drugs used for diabetes, diuretics, drugs used for obesity, and cardiovascular drugs)

The sponsor should be contacted if there are any questions regarding concomitant or prior therapy.

Initial doses of tirzepatide delay gastric emptying and have the potential to transiently impact the rate of absorption of concomitantly administered oral medicinal products. Tirzepatide should be used with caution in participants receiving oral medicinal products that require rapid GI absorption following the initial doses of tirzepatide, as exposure to oral medications may be increased.

Prevention of Hypoglycemia

Similar to GLP-1 RAs, tirzepatide does not generally cause hypoglycemia, but it is recommended to decrease the dose of concomitant sulfonylurea or insulin to reduce the risk of hypoglycemic episodes in patients with T2DM. For participants with T2DM, specific, individually tailored adjustments of the respective antihyperglycemic medications should be considered during the entire study.

At Visit 2, with the initiation of study drug, the dose adjustments to the following concomitant glucose lowering medications are recommended.

Sulfonylureas: Sulfonylurea dose is recommended to be reduced at least 50% or discontinued, especially if the participant is receiving a low dose at randomization.

Insulins: For participants on basal insulin and with screening HbA1c ≤8.5%, the daily dose is recommended to be reduced by at least 20%.

During the dose escalation period, consider adjusting the total daily dose of insulin, if required for controlling worsening hyperglycemia and its acute complications.

During the maintenance period, further insulin dose reduction for the prevention of hypoglycemia is to be considered at the investigator's discretion.

Standard of Care for T2DM

The standard of care for diabetes may be adjusted at the discretion of the investigator as clinically indicated in accordance with local standard of care and professional society guidelines.

Participants will be permitted to use concomitant medications that they require during the study, except certain medications that may interfere with the assessment of efficacy and safety characteristics of the study drug. Prohibited medications include all GLP-1 RAs and pramlintide. Discontinuation of dipeptidyl peptidase 4 inhibitors at randomization is recommended in line with guidelines. Similarly, the use of dipeptidyl peptidase 4 inhibitor therapies during the study is also discouraged (Davies et al. 2018).

Regarding the use of insulin, participants can be included if treated with basal insulin only; and are allowed to use prandial insulin if needed during the study to attain optimal glucose control.

Hyperglycemia Rescue

Other medications for glycemic control for participants with T2DM meeting severe, persistent hyperglycemia criteria for rescue may be added during the study at the investigator's discretion.

Rescue therapy with glucose-lowering agents, including basal and prandial insulins, may be medically indicated in situations after randomization due to severe, persistent hyperglycemia or early discontinuation of study drug.

Hyperglycemia rescue criteria will be determined from values recorded in T2DM participant diaries. If a diary value equal to or greater than the glycemic threshold for rescue (see definitions below) is recorded, that value should be confirmed by a repeat fasting glucose text within 48 hours (for example, local laboratory). Intensification of T2DM therapy should be initiated if confirmed fasting glucose values are:

- ≥15.0 mmol/L (270 mg/dL) from baseline to Week 6 over at least a 2-week period (at least 2 consecutive values) after randomization
- ≥13.3 mmol/L (240 mg/dL) from Week 6 to Week 12 over at least a 2-week period (at least 2 consecutive values)
- ≥11.1 mmol/L (200 mg/dL) from Week 12 to end of trial over at least a 2-week period (at least 2 consecutive values)

In addition, if HbA1c is >9.0% at Week 12 or >8.0% at Week 24 or later in the study, glucose-lowering therapy should be adjusted to improve glycemic control as outlined above. In the event a participant's HbA1c values are less than these thresholds but are higher than what the investigator feels comfortable leaving untreated, glucose-lowering medication can be adjusted. In addition, if participants develop symptoms of hyperglycemia (for example, polyuria and polydipsia), the investigator should implement measures to determine glycemic control and adjust as necessary. For participants newly diagnosed with T2DM during the trial, appropriate glucose-lowering therapy should be initiated per standard of care.

Standard of Care for Heart Failure

Anticipated treatment for heart failure should be decided prior to randomization.

Both American College of Cardiology/American Heart Association and European Society of Cardiology guidelines recommend symptom management with diuretic agents in patients with excess volume, as well as aggressive risk factor management for comorbidities for the treatment of HFpEF (van der Meer JACC 2019). Optimization of volume status and proactive adjustment of diuretic doses will help control symptoms and volume overload.

Participants should remain on stable doses of medications to treat heart failure condition and comorbidities such as hypertension. With the exception of diuretics, dose modification or alteration of such background therapies should be avoided unless all other measures fail to improve the participant's condition. However, if the participant's condition warrants a change in any of these medications, it will be allowed at the discretion of the investigator.

Management of Participants with Gastrointestinal Symptoms

In the Phase 2 program, the most commonly reported TEAEs for participants receiving tirzepatide were nausea, vomiting, and diarrhea. To mitigate GI symptoms and manage participants with intolerable GI AEs, the investigator should:

- Advise participants to eat smaller meals, for example, splitting 3 daily meals into 4, or more smaller meals, and to stop eating when they feel full. Also, participants may be informed that lower-fat meals could be better tolerated.
- Prescribe symptomatic medication (for example, anti-emetic or antidiarrheal medication) per local country availability and individual participant needs. Use of symptomatic medication should be captured as concomitant medication in the eCRF.
- Temporarily interrupt study drug. See Section 6.6.1. The data related to temporary interruption of study drug should be documented in source documents and entered on the eCRF.
- After the interruption, follow the guidance for restarting study drug (Section 6.6.2).

If intolerable GI symptoms or events persist despite the above measures, other dose modifications (see Section 6.6) may be considered.

6.6. Dose Modification

Interventions to optimize study drug tolerance and adherence may be employed throughout the study and include, but are not limited to, brief temporary interruptions and use of additional medications to manage symptoms.

Dose modifications, including temporary interruption and de-escalation may occur to manage issues with tolerability. It is preferred to attempt a temporary dose interruption (at any time) (Section 6.6.1) to manage tolerability issues. After a temporary dose interruption, participants may resume study drug at the same dose, re-escalate to the prior dose level (if re-escalation is desired or required), or resume at a lower MTD dose level, as tolerated. Guidance for resuming study drug after a temporary dose interruption should be followed (Section 6.6.2).

Unwarranted excessive weight reduction: **Dose interruption is preferred over de-escalation for slowing unwarranted excessive weight loss; however, the method used is at the investigator's discretion.** After the dose escalation period and at the investigator's discretion, when excessive weight reduction is not warranted due to safety concerns, the investigator may choose to adjust the study drug dose without first attempting a temporary dose interruption. The participant's study drug dose will be permanently reduced to 5 mg, in a blinded fashion for the remainder of the study and the dose cannot be re-escalated. A dose adjustment for unwarranted excessive weight reduction is completed through the IWRS. Participants on 5 mg will have blinded study drug temporarily interrupted (Section 6.6.1).

Dose reductions for unwarranted excessive weight loss may occur at scheduled and unscheduled visits.

6.6.1. Temporary Interruption

Temporary study drug interruption should be utilized to manage tolerability issues. After randomization, the investigator may interrupt study drug, for example, due to an AE (such as nausea vomiting, excessive unwarranted weight loss or a clinically significant laboratory value). Guidance for resuming study drug after a temporary dose interruption should be followed (Section 6.6.2). If study drug interruption is due to an AE, the event is to be followed and documented.

For cases where increased ALT, AST, or ALP occurs, study drug may be interrupted (Section 6.6.2), and close hepatic monitoring must be initiated (Section 8.2.5.1). The interruption of study drug is not equivalent to discontinuation from study treatment. Any interruption of study drug does not affect the study visit structure/schedule per the SoA (Section 1.3). Even if study drug is interrupted, study procedures should continue during the dose interruption. Every effort should be made by the investigator to maintain participants in the study and to restart study drug promptly after any interruption, as soon as it is safe to do so (see Section 6.6.2. for restarting study drug). Dose interruptions are managed through the IWRS. The data related to interruption of study drug will be documented in source documents and entered on the eCRF, however participant noncompliance should not be recorded as interruption of study drug on the eCRF.

Participants who have a temporary interruption of the study drug will continue participating in the trial according to the protocol to collect all planned efficacy and safety measurements (Section 1.3).

6.6.2. Restarting Study Drug after Interruption

In certain situations, the investigator may need to temporarily interrupt study drug. Every effort should be made by the investigator to maintain participants on study drug and to restart study drug after any temporary interruption, as soon as it is safe to do so. Distribution of study drug at the correct dose will be per IWRS instructions.

If study drug interruption is…	then…
1 or 2 consecutive doses	Participant restarts study drug at last administered dose, per escalation schedule. If the participant has reached maintenance dose level, the study drug dose level will restart at the same prior achieved maintenance dose level.
3 or more consecutive doses	Participant restarts study drug (at 5 mg, managed by IWRS) and repeats dose escalation scheme until maintenance dose is reached. If maintenance dose has previously been established, the dose escalation cannot not exceed the prior achieved maintenance dose level.
Due to an AE	The event is to be documented and followed according to the procedures in Section 8.3.
Due to intolerable persistent GI AE	Participants should be treated as suggested in Section 6.5.

Abbreviations: AE = adverse event; GI = gastrointestinal; IWRS = interactive web response service.

Investigators should inform the sponsor that study drug has been temporarily interrupted.

Participants are not required to re-escalate to the prior maintenance dose level if a tolerability issue recurs during dose re-escalation.

If this attempted dose re-escalation to the prior maintenance dose level is not tolerated, the dose should be reduced to the next lower 5 mg incremental dose that was tolerated (for example, 5 mg or 10 mg). The participant will remain at that dose level for the duration of the study. During re-escalation after a temporary dose interruption, participants should be followed every 4 weeks until either a new lower maintenance dose level or prior maintenance dose level is reached.

For participants receiving 5 mg maintenance dose, no dose de-escalation is permitted. Only dose interruption is permitted to manage tolerability issues (Section 6.6.2). This can be performed through IWRS.

In the event that a participant has a temporary interruption that requires extending the escalation beyond Visit 8, unscheduled visits are allowed in the IWRS to facilitate a 4-week dispensing schedule to complete the escalation.

If an unscheduled visit occurs in the same week or date of a regular scheduled visit per the SoA, the site should complete all procedures included for the regular scheduled visit. Unscheduled

visits either in the clinic or by telephone may be conducted to provide support and guidance to participants as needed.

6.7. Intervention after the End of the Study

Tirzepatide will not be made available to participants after conclusion of the study. Due to the double-blind study drug assignment, it is not known if a participant received active study drug or placebo. Participants will not be unblinded until study end and the final analyses are complete.

7. Discontinuation of Study Intervention and Participant Discontinuation/Withdrawal

7.1. Discontinuation of Study Intervention

7.1.1. Permanent Discontinuation from Study Drug

Before permanently discontinuing study drug, attempts to maintain the participant should be documented (see Dose Modification, Section 6.6 and Temporary Dose Interruption, Section 6.6.1). Contact the sponsor before study treatment discontinuation occurs to discuss potential options to maintain the participant on study drug until final study ends. It is the goal for participants to remain on study drug treatment until study ends.

Permanent discontinuation of study drug will not automatically lead to discontinuation from the study. If study drug is permanently discontinued, the participant will remain in the study and attend all scheduled visits to be evaluated for safety and efficacy as described in the SoA.

Possible reasons leading to permanent discontinuation of study drug:

- **Participant decision**
    - The participant or the participant's designee (for example, legal guardian) requests to discontinue study drug
- **Investigator Decision**
    - The investigator decides that the participant should be discontinued from study drug
- **Discontinuation due to a hepatic event or liver test abnormality**
  Please refer to Section 8.2.5.1 for liver chemistry stopping criteria and Section 8.2.5.4 for study drug interruption or discontinuation due to an hepatic event. Participants who experience a hepatic event or liver test abnormality should have additional hepatic safety data collected via CRF (see Section 8.2.5.1 for details). Resumption of the study drug can be considered only in consultation with the Lilly-designated medical monitor and only if the liver test results return to baseline and if a self-limited non-drug etiology is identified.

- In addition, participants will be permanently discontinued from the study drug in the following circumstances:
    - acute or chronic pancreatitis (see Section 10.5.1.2)
    - if a participant is diagnosed with thyroid C-cell hyperplasia, MEN-2, or MTC, or pancreatic cancer after randomization
    - if any other TEAE, SAE, or clinically significant laboratory value for which the investigator believes that permanent study drug discontinuation is the appropriate measure to be taken
    - if a participant is diagnosed with T1DM
    - if a female participant becomes pregnant
    - if an investigator, site personnel performing assessments, or participant is unblinded

- if the investigator, after consultation with the sponsor-designated medical resource, determines that a clinically significant systemic hypersensitivity reaction has occurred. A clinically significant systemic hypersensitivity reaction is one that occurs after administration of the study drug (for example, drug-related symptomatic bronchospasm, allergy-related edema/angioedema, or hypotension) and requires parenteral medication, does not respond to symptomatic medication, results in clinical sequelae, or is an anaphylactic reaction.
- if the participant undergoes any bariatric surgery during the study
- if the participant begins treatment with a GLP-1RA during the study,
- in the opinion of the investigator, the participant should permanently discontinue the study intervention for safety reasons.

7.2. Participant Discontinuation/Withdrawal from the Study

In order to minimize the amount of missing data and to enable assessment of study objectives as planned in the study protocol, every attempt will be made to keep participants in the study, irrespective of the following:

- compliance to study drug
- adherence to visit schedule
- missing assessments
- study drug discontinuation due to AE (Section 7)
- development of comorbidities, and
- development of clinical outcomes.

The circumstances listed above are ***not*** valid reasons for participant discontinuation from the study.

Participant will be discontinued from study in the following circumstances:

- enrollment in any other clinical study involving a study drug or enrollment in any other type of medical research judged not to be scientifically or medically compatible with this study
- participation in the study needs to be stopped for medical, safety, regulatory, or other reasons consistent with applicable laws, regulations, and GCP, and
- if the participant becomes pregnant.

A participant may discontinue from the study:

- at any time at his/her own request
- at the request of his/her designee (for example, legal guardian)
- at the discretion of the investigator for safety, behavioral, compliance, or administrative reasons
- if enrolled in any other clinical study involving an investigational product, or enrolled in any other type of medical research judged not to be scientifically or medically compatible with this study, or

- if the participant, for any reason, requires treatment with a therapeutic agent that is prohibited by the protocol and has been demonstrated to be effective for treatment of the study indication. In this case, discontinuation from the study occurs prior to introduction of the new agent.

Study discontinuation is expected to be rare and participants should be provided with options for alternative follow-up methods and/or end of study vital status/endpoint ascertainment.

If the participant is unwilling or unable to return for all scheduled follow-up visits in person, the site will attempt to collect as much follow-up information as possible via telephone or other virtual methods of direct patient contact with the patient. Sites are expected to conduct these alternative visit methods according to the visit interval outlined in the Schedule of Activities.

An ET visit should be conducted, as shown in the SoA. If the participant refuses to have an ET visit in the clinic, efforts should be made to collect data via telephone. See the SoA for data to be collected at the time of ET visit. If the participant has not already discontinued the study intervention, the participant will be permanently discontinued from the study intervention at the time of the decision to discontinue the study.

If the participant withdraws consent for disclosure of future information, the sponsor may retain and continue to use any data collected before such a withdrawal of consent. If a participant withdraws from the study, he/she may request destruction of any samples taken and not tested, and the investigator must document this in the site study records.

7.2.1. Inadvertently Enrolled Participants

If the sponsor or investigator identifies a participant who did not meet enrollment criteria and was inadvertently enrolled, then the participant should remain in the study and be discontinued from study drug when continued treatment would not be medically appropriate. If the investigator and the sponsor CRP agree it is medically appropriate to continue the study drug, the investigator must obtain documented approval from the sponsor Chief Medical Officer (CMO) to allow the inadvertently enrolled participant to continue study drug. Safety follow-up should be performed as outlined in Section 8.2 (Safety Assessments) and Section 8.3 (Adverse Events and Serious Adverse Events) of the protocol.

7.3. Lost to Follow up

A participant will be considered high risk for lost to follow-up if he or she repeatedly fails to return for scheduled visits. Site personnel or designee are expected to make diligent attempts to contact participants who fail to return for a scheduled visit or were otherwise unable to be followed up by the site. If a participant refuses all means of completing study visits, contact with the patient's family or the patient's primary physician or medical record review during the study and at the end of the study to ascertain vital status and record safety and efficacy endpoints is required.

The disposition of lost to follow-up will be documented in the eCRF at the time of study end. Site personnel should continue to enter missed visits into both IWRS and eCRF. A patient will be considered as lost to follow-up only if the patient is unable to be contacted by the study site during the study close-out period, and no data on vital status (alive or dead) is available through accepted methods for ascertainment, including query of public databases, contact with patient's

family or designee/personal contact (e.g., friend), family doctor, or attempt to determine vital status and endpoints via other means (if not prohibited by local laws) (e.g., national registries/databases, medical records, voter records, and third-party patient locator services).

If vital status is determined, this will be documented and the patient will not be considered lost to follow-up. If no final visit is available, and vital status is not determined during the study close-out period, this will be documented and the patient will be considered lost to follow-up.

Note: If the investigator site personnel are unable to contact the patient, they may give the patient's name and last known contact information to a patient locator service to try to find current information, if not prohibited by local laws and regulations. The patient locator service will **not** contact the patient directly and any new information they find will be shared with investigator site personnel.

Sponsor personnel will not be involved in any attempts to collect vital status information.

Discontinuation of specific sites or of the study as a whole are handled as part of Appendix 1 (Section 10.1)

8. Study Assessments and Procedures

Study procedures and their timing are summarized in the SoA.

Immediate safety concerns should be discussed with the sponsor immediately upon occurrence or awareness to determine if the participant should continue or discontinue study drug.

Adherence to the study design requirements, including those specified in the SoA, is essential and required for study conduct.

All screening evaluations must be completed and reviewed to confirm that potential participants meet all eligibility criteria. The investigator will maintain a screening log to record details of all participants screened and to confirm eligibility or record reasons for screening failure, as applicable.

Repeat or unscheduled samples may be taken for safety reasons or for technical issues with the samples.

Other visit methods (i.e. remote, telephone) may be considered at the discretion of the investigator if a participant is unable to come to their scheduled on-site visit. Alternative options to visit procedures must be considered with prior consultation and written approval of the sponsor.

8.1. Efficacy Assessments

8.1.1. Primary Efficacy Assessment

The primary efficacy endpoints are

- change from baseline to Week 52 in KCCQ-CSS.
- occurrence of the composite endpoint of CV death and/or HF events over time, and

Death and heart failure events will be recorded by the investigator at the time of event discovery. An independent CEC will adjudicate death (CV, non-CV, unknown cause) and HF events. The CEC charter will contain the final detailed event definitions for all adjudicated events.

8.1.1.1. Kansas City Cardiomyopathy Questionnaire

The KCCQ is a 23-item, participant self-administered questionnaire that assesses impacts of HF "over the past 2 weeks" on the following 7 domains (Green et al. 2000; Joseph et al. 2013):

- Physical Limitation (6 items)
- Symptom Stability (1 item)
- Symptom Frequency (4 items)
- Symptom Burden (3 items)
- Self-Efficacy (2 items)
- Quality of Life (3 items), and
- Social Limitation (4 items).

Each of the 23 individual items are answered on Likert scales of varying lengths (5-point, 6-point, or 7-point scales). Domain scores are obtained by averaging the associated individual

items and transforming the score to a 0 to 100 range. Higher scores indicate better health status. Summary scores are obtained by combining select domain scores:

- Total Symptom Score: mean of the Symptom Frequency and Symptom Burden scores
- Clinical Summary Score: mean of the Physical Limitation and Total Symptom scores, and
- Overall Summary Score: mean of the Physical Limitation, Total Symptom, Quality of Life, and Social Limitation scores.

The Clinical Summary Score will be used for the primary and key secondary endpoints.

KCCQ collections are required per the SoA at Visits 8 and 12 for all participants in the study regardless study drug status (on or off study drug).

If Visit 12 is missed or not performed within the SoA window of the Visit 12 KCCQ (52 ± 7 days from Visit 2), it is requested that the KCCQ be performed within 30 days of missed Visit 12 date. If a participant discontinues study before reaching Visit 12, a KCCQ must be conducted at the early termination visit.

The KCCQ and all other self-reported questionnaires will be translated into the native language of the region, linguistically validated, and administered according to the SoA (Section 1.3). At these visits, the questionnaires should be completed before the participant has discussed their medical condition or progress in the study with the investigator and/or site staff.

8.1.1.2. Definition of Heart Failure Events

The heart failure event definition within the protocol includes worsening symptoms or signs of HF, which are meaningful to the participant and require intensification of treatment characterized by one or more of the following: hospitalization for heart failure regardless of duration or treatment received; use of intravenous drug, usually an intravenous diuretic, but may include intravenous vasodilators or positive inotropic drugs; or augmentation or increase in oral diuretic therapy.

8.1.2. Secondary Efficacy Assessments

8.1.2.1. Six-Minute Walk Test

Participants will perform an exercise capacity assessment using the 6MWT. Testing of the 6MWT should be performed as directed in the SoA (Section 1.3). The 6MWT is to be performed indoors on a straight, flat, hard surface that is at least 30 meters in length.

The 6MWTs at Visits 1 and 2 will be performed to assess participant eligibility. The Visit 1 tests will also serve as the training test to familiarize participants with the procedure. Additional details can be found in Section 10.10.1.

Prior to and at the end of each 6MWT, participants will be asked to rate their breathing discomfort and overall fatigue using the Borg Scale, separately, at each timepoint.

6MWT are required per the SoA at Visits 8 and 12 for all participants in the study regardless study drug status (on or off study drug).

If Visit 12 is missed or not performed within the SoA window of the Visit 12 6MWT (52 ± 7 days from Visit 2), it is requested that the 6MWT be performed within 30 days of missed Visit 12 date.

If a participant discontinues study before reaching Visit 12, a 6MWT must be conducted at the early termination visit.

8.1.2.2. Body Weight and hsCRP

Body weight will be assessed as described in Section 8.2.1. hsCRP will be assessed as described in Section 8.2.4.

8.1.3. Exploratory Efficacy Assessments

8.1.3.1. Patient Global Impression of Status

Three patient global impression items will be assessed and are described below.

8.1.3.1.1. Patient Global Impression of Status – Overall

Study participants will be asked to complete a Patient Global Impression of Status – Overall item specifically developed for this study. This is a participant-rated assessment of their overall health "in the past 2 weeks" and is rated on a 5-point scale ranging from "1-Excellent" to "5-Poor."

8.1.3.1.2. Patient Global Impression of Status – Physical Function

Study participants will be asked to complete a Patient Global Impression of Status – Physical Function item specifically developed for this study. This is a participant-rated assessment of the overall impact of HF symptoms on their ability to perform physical activities "in the past 2 weeks" and is rated on a 5-point scale ranging from "1- Not impacted" to "5- Extremely impacted, cannot perform physical activities."

8.1.3.1.3. Patient Global Impression of Status – Symptom Severity

Study participants will be asked to complete a Patient Global Impression of Status – Symptom Severity item specifically developed for this study. This is a participant-rated assessment of the overall severity of their HF symptoms "in the past 2 weeks" and is rated on a 5-point scale ranging from "1- No symptoms" to "5- Very severe."

8.1.3.2. EQ-5D-5L

Generic health-related quality of life will be assessed using the EQ-5D-5L (EuroQoL Research Foundation 2019). The EQ-5D-5L is a standardized 5-item instrument for use as a measure of health outcome. It provides a simple descriptive profile and a single index value for health status that can be used in the clinical and economic evaluation of health care as well as population health surveys. The EQ-5D-5L comprises 5 dimensions of health (mobility, self-care, usual activities, pain/discomfort, and anxiety/depression). The 5L version, introduced in 2005, scores each dimension at 5 levels (no problems, slight problems, moderate problems, severe problems, and unable to perform/extreme problems), for a total of 3125 possible health states. In addition to the health profile, a single health state index value can be derived based on a formula that attaches weights to each of the levels in each dimension. This index value ranges between less than 0 (where 0 is a health state equivalent to death; negative values are valued as worse than

dead) to 1 (perfect health). In addition, the EQ Visual Analog Scale records the respondent's self-rated health status on a vertical graduated (0 to 100) visual analog scale. In conjunction with the health state data, it provides a composite picture of the respondent's health status.

The EQ-5D-5L is used worldwide and is available in more than 170 languages. Details on the instrument, scoring, organizing, and presenting the data collected can be found in the EQ-5D-5L User Guide (EuroQoL Research Foundation 2019).

8.1.3.3. NYHA Classification

The NYHA classification will be assessed and recorded at the time points indicated in the SoA (Section 1.3) by an independent, blinded assessor. The NYHA classification is provided in Appendix 11 (Section 10.11).

8.2. Safety Assessments

Planned time points for all safety assessments are provided in the SoA (Section 1.3).

8.2.1. Physical Examinations

- A complete physical examination will include, at a minimum, assessments of the CV, respiratory, GI and neurological systems, as well as a thyroid examination.
    - Body weight, waist circumference, and height should be measured. All weights for a given participant should be measured in a consistent manner using a calibrated scale (mechanical or digital scales are acceptable), using the same scale whenever possible, and after the participant has emptied their bladder. Participants should be lightly clothed but not wearing shoes while their weight is measured.
- Symptom-directed physical examinations will be conducted as described in the SoA.
    - Investigators should pay special attention to clinical signs and symptoms related to HF as well as related to previous serious illnesses. Particular interest would include dyspnea, orthopnea, paroxysmal nocturnal dyspnea, edema, jugular venous distension, and rales.

The physical examination should be performed before the first 6MWT, if more than one 6MWTs are done.

8.2.2. Vital Signs

For each participant, vital signs measurements should be conducted according to the SoA (Section 1.3). An apical heart rate should be assessed during the collection of vital signs. The vital signs collection associated with the 6MWT should be separate and may be performed using automated equipment.

Any clinically significant findings from vital signs measurements that result in a diagnosis and that occur after the participant receives the first dose of study drug should be reported to the sponsor or its designee as an AE via the eCRF.

8.2.3. Electrocardiograms

Single 12-lead ECGs will be obtained locally as outlined in the SoA (see Section 1.3).

All ECGs should be recorded after the participant has been supine for 5 minutes in a quiet room.

The ECGs must be interpreted by a qualified physician (the investigator or designee) at the site as soon after the time of ECG collection as possible, and ideally while the participant is still present, for immediate participant management, if needed. The investigator (or qualified designee) is responsible for determining if any change in participant management is needed, and must document his/her review of the ECG printed at the time of evaluation. If a clinically relevant abnormality is observed on the participant's ECG, then the investigator should assess the participant for symptoms (such as palpitations, near syncope, syncope, or chest pain). The investigator must report the presence of AF on the eCRF.

The original ECG must be retained at the investigative site.

The investigator or qualified designee's interpretation will prevail for immediate participant management purposes.

8.2.4. Clinical Safety Laboratory Assessments

With the exception of laboratory test results that may unblind the study, the sponsor or its designee will provide the investigator with the results of laboratory tests analyzed by a central vendor, if a central vendor is used for the clinical trial.

See Appendix 2 (Section 10.2) for the list of clinical laboratory tests to be performed. The SoA describes the timing and frequency.

The investigator must review the laboratory results, document this review, and report any clinically relevant changes occurring during the study as an AE. The laboratory results must be retained with source documents unless a Source Document Agreement or comparable document cites an electronic location that accommodates the expected retention duration. Clinically significant abnormal laboratory findings are those that are not associated with the underlying disease, unless judged by the investigator to be more severe than expected for the participant's condition.

All laboratory tests with values considered clinically significantly abnormal during participation in the study should be repeated until the values return to normal or baseline levels or are no longer considered clinically significant by the investigator or medical monitor.

- If such values do not return to normal/baseline levels within a period of time judged reasonable by the investigator, the etiology should be identified and the sponsor notified.
- All protocol-required laboratory assessments, as defined in Appendix 2 (Section 10.2), must be conducted in accordance with the SoA, standard collection requirements, and laboratory manual.

If laboratory values from non-protocol specified laboratory assessments performed at an investigator-designated local laboratory require a change in participant management or are considered clinically significant by the investigator (for example, SAE or AE or dose modification), then report the information as an AE.

All urine pregnancy tests will be performed locally according to the SoA. Guidance for contraception and definitions are defined in Appendix 4 (Section 10.4).

8.2.5. Safety Monitoring

The sponsor will periodically review evolving aggregate safety data within the study by appropriate methods. The study team will review safety reports in a blinded fashion according to the schedule provided in the Trial-Level Safety Review Plan. The sponsor will also review SAEs within time frames mandated by company procedures. The Sponsor CRP will, as appropriate, consult with the functionally independent Global Patient Safety therapeutic area physician or clinical scientist.

8.2.5.1. Hepatic Safety Monitoring, Evaluation, and Criteria for Study Drug Interruption or Discontinuation

The following tables summarize actions to take based on abnormal hepatic laboratory or clinical changes.

Participants with normal or near-normal baseline (ALT, AST, or ALP <1.5x ULN)

If this laboratory value is observed…	Then…		
	Initiate or continue close hepatic monitoring	Initiate comprehensive evaluation	Interrupt or discontinue study drug
ALT or AST ≥3x ULN	X		
ALP ≥2x ULN	X		
TBL ≥2x ULN[b]	X		
ALT or AST ≥5x ULN	X	X	
ALP ≥2.5x ULN	X	X	
ALT or AST ≥3x ULN with hepatic signs or symptoms[a]	X	X	X
ALT or AST ≥5x ULN for more than 2 weeks	X	X	X
ALT or AST ≥8x ULN	X	X	X
ALT or AST ≥3x ULN and TBL ≥2x ULN[b] or INR≥ 1.5	X	X	X
ALP ≥3x ULN	X	X	X
ALP ≥2.5x ULN and TBL ≥2x ULN[b]	X	X	X
ALP ≥2.5x ULN with hepatic signs or symptoms[a]	X	X	X

[a] Examples of hepatic signs or symptoms: severe fatigue, nausea, vomiting, right upper quadrant abdominal pain, fever, rash, and/or eosinophilia >5%.
[b] In participants with Gilbert's syndrome, the threshold for TBL may be higher.

Participants with elevated baseline (ALT, AST, or ALP ≥ 1.5x ULN)

If this laboratory value is observed…	Then…		
	**Initiate or continue close hepatic monitoring**	**Initiate comprehensive evaluation**	**Interrupt or discontinue study drug**
ALT or AST ≥2x baseline	X		
ALP ≥2x baseline	X		
TBL ≥2x ULN[b]	X		
ALT or AST ≥3x baseline or ≥250 U/L (whichever occurs first)	X	X	
ALP ≥2.5x baseline	X	X	
ALT or AST ≥2x baseline or ≥250 U/L (whichever occurs first) with hepatic signs or symptoms[a]	X	X	X
ALT or AST ≥3x baseline or ≥250 U/L (whichever occurs first) for more than 2 weeks	X	X	X
ALT or AST ≥4x baseline or ≥400 U/L (whichever occurs first)	X	X	X
ALT or AST ≥2x baseline or ≥250 U/L (whichever occurs first) and TBL ≥2x ULN[b] or INR ≥1.5	X	X	X
ALP ≥3x baseline	X	X	X
ALP ≥2.5x baseline and TBL ≥2x ULN[b]	X	X	X
ALP ≥2.5x baseline with hepatic signs or symptoms[a]	X	X	X

[a] Examples of hepatic signs or symptoms: severe fatigue, nausea, vomiting, right upper quadrant abdominal pain, fever, rash, and/or eosinophilia >5%.
[b] In participants with Gilbert's syndrome, the threshold for TBL may be higher.

Additional hepatic data collection (hepatic safety CRF) in study participants who have abnormal liver tests during the study

Additional hepatic safety data collection in hepatic safety CRFs should be performed in study participants who meet 1 or more of the following 5 conditions:

1. Elevation of serum ALT to ≥5X ULN on 2 or more consecutive blood tests (if baseline ALT <1.5X ULN)
    - In participants with baseline ALT ≥1.5X ULN, the threshold is ALT ≥3X baseline on 2 or more consecutive tests
2. Elevated TBL to ≥2X ULN (if baseline TBL <1.5X ULN) (except for cases of known Gilbert's syndrome)
    - In participants with baseline TBL ≥1.5X ULN, the threshold should be TBL ≥2X baseline
3. Elevation of serum ALP to ≥2X ULN on 2 or more consecutive blood tests (if baseline ALP <1.5X ULN)

   - In participants with baseline ALP ≥1.5X ULN, the threshold is ALP ≥2X baseline on 2 or more consecutive blood tests

4. Hepatic event considered to be an SAE
5. Discontinuation of study drug due to a hepatic event

Note: the interval between the two consecutive blood tests should be at least 2 days.

8.2.5.2. Close Hepatic Monitoring

Close hepatic monitoring should include these actions:

- Laboratory tests (Appendix 8 [Section 10.8]), including ALT, AST, ALP, TBL, D. Bil, GGT, CK, and CBC with differential, should be checked within 48 to 72 hours of the detection of elevated liver tests to confirm the abnormality and to determine if it is increasing or decreasing.
- If the abnormality persists, clinical and laboratory monitoring should continue at a frequency of 2-3 times weekly until levels normalize or return to approximate baseline values.
- In addition to lab tests, basic evaluation for possible causes of abnormal liver tests should be initiated by the investigator in consultation with the Lilly-designated medical monitor. At a minimum, this evaluation should include physical examination and a thorough medical history, including current symptoms, recent illnesses (for example, heart failure, systemic infection, hypotension, or seizures), recent travel, concomitant medications (including over-the-counter), herbal and dietary supplements, history of alcohol drinking and other substance abuse.

8.2.5.3. Comprehensive Hepatic Evaluation

Comprehensive hepatic evaluation should include these actions:

- At a minimum, comprehensive hepatic evaluation should include physical examination and a thorough medical history, as outlined above, as well as tests for PT-INR; tests for viral hepatitis A, B, C, and E; tests for autoimmune hepatitis; and an abdominal imaging study, for example, ultrasound or CT scan.
- Based on the participant’s history and initial results, further testing should be considered in consultation with the Lilly-designated medical monitor, including tests for hepatitis D virus (HDV), cytomegalovirus (CMV), Epstein-Barr virus (EBV), acetaminophen levels, acetaminophen protein adducts, urine toxicology screen, Wilson’s disease, blood alcohol levels, urinary ethyl glucuronide, and blood phosphatidylethanol.
- Based on the circumstances and the investigator’s assessment of the participant’s clinical condition, the investigator should consider referring the participant for a hepatologist or gastroenterologist consultation, and additional tests including magnetic resonance cholangiopancreatography (MRCP), endoscopic retrograde cholangiopancreatography (ERCP), cardiac echocardiogram, or a liver biopsy.

- Clinical and laboratory monitoring should continue at a frequency of 1-2 times weekly until levels normalize or return to approximate baseline values.
- All the medical information and test results related to the hepatic monitoring and comprehensive hepatic evaluation should be collected and recorded in a hepatic safety case report form (CRF).

8.2.5.4. Study Drug Interruption or Discontinuation due to a Hepatic Event

Interruption or discontinuation of study drug should include these actions:

- While the participant is not receiving the study drug, clinical and laboratory monitoring should continue at a frequency of 1 to 2 times weekly until liver tests normalize or return to approximate baseline values.
- If the hepatic event continues past the anticipated end of the study (that is, data lock), the investigator should consult with the Lilly-designated medical monitor to determine the need for further data collection beyond the end date of the study (that is, data lock date).
- All the medical information and test results related to the close hepatic monitoring and comprehensive hepatic evaluation should be collected and recorded in a hepatic safety case report form (CRF).
- Resumption of the study drug after interruption for a hepatic reason can be considered only in consultation with the Lilly-designated medical monitor and only if the liver test results returned to near baseline and if a self-limited non-study-drug etiology is identified. Otherwise, the study drug should be permanently discontinued.

8.3. Adverse Events, Serious Adverse Events, and Product Complaints

The definitions of the following events can be found in Appendix 3 (Section 10.3):

- Adverse events (AEs)
- Serious adverse events (SAEs)
- Product complaints (PCs)

These events will be reported by the participant (or, when appropriate, by a caregiver, surrogate, or the participant's legally authorized representative).

The investigator and any qualified designees are responsible for detecting, documenting, and recording events that meet these definitions and remain responsible for following up events that are serious, considered related to the study drug or study procedures, or that caused the participant to discontinue the study drug or study (see Section 7).

Care will be taken not to introduce bias when detecting events. Open-ended and non-leading verbal questioning of the participant is the preferred method to inquire about event occurrences.

After the initial report, the investigator is required to proactively follow each participant at subsequent visits/contacts. All SAEs will be followed until resolution, stabilization, the event is otherwise explained, or the participant is lost to follow-up (as defined in Section 7.3).

For product complaints, the investigator is responsible for ensuring that follow-up includes any supplemental investigations as indicated to elucidate the nature and/or causality. Further information on follow-up procedures is provided in Appendix 3 (Section 10.3).

Suspected Unexpected Serious Adverse Reactions

Suspected unexpected serious adverse reactions (SUSARs) are serious events that are not listed in the IB and that the investigator identifies as related to study drug or procedure. United States 21 CFR 312.32, European Union Clinical Trial Directive 2001/20/EC, and the associated detailed guidance or national regulatory requirements in participating countries require the reporting of SUSARs. The sponsor has procedures that will be followed for the identification, recording, and expedited reporting of SUSARs that are consistent with global regulations and the associated detailed guidance.

8.3.1. Timing and Mechanism for Collecting Events

This table describes the timing, deadlines, and mechanism for collecting events.

Event	Collection Start	Collection Stop	Timing for Reporting to Sponsor or Designee	Mechanism for Reporting	Back-up Method of Reporting
**Adverse Event**					
AE	Signing of the informed consent form (ICF)	Participation in study has ended	As soon as possible upon site awareness	AE eCRF	N/A
**Serious Adverse Event**					
SAE and SAE updates – prior to start of study drug **and** deemed reasonably possibly related with study procedures	Signing of the informed consent form (ICF)	Start of study drug	Within 24 hours of awareness	SAE eCRF	SAE paper form

Event	Collection Start	Collection Stop	Timing for Reporting to Sponsor or Designee	Mechanism for Reporting	Back-up Method of Reporting
SAE* and SAE updates – after start of study drug	Start of study drug	Participation in study has ended	Within 24 hours of awareness	SAE eCRF	SAE paper form
SAE* – after participant's study participation has ended **and** the investigator becomes aware	After participant's study participation has ended	N/A	Promptly	SAE paper form	N/A
**Pregnancy**					
Pregnancy in female participants and female partners of male participants	After the start of study drug	Four months after the last injection for female partners of male participants and 2 months after the last injection for female participants	Within 24 hours of learning of the pregnancy	Pregnancy paper form eCRF	Pregnancy paper form
**Product Complaints**					
PC associated with an SAE or might have led to an SAE	Start of study drug	End of study drug	Within 24 hours of awareness	Product Complaint form	N/A
PC not associated with an SAE	Start of study drug	End of study drug	Within 1 business day of awareness	Product Complaint form	N/A

Event	Collection Start	Collection Stop	Timing for Reporting to Sponsor or Designee	Mechanism for Reporting	Back-up Method of Reporting
Updated PC information	—	—	As soon as possible upon site awareness	Originally completed Product Complaint form with all changes signed and dated by the investigator	N/A
PC (if investigator becomes aware)	Participation in study has ended	N/A	Promptly	Product Complaint form	N/A

Abbreviations: eCRF = electronic case report form; N/A = not applicable; PC = product complaint; SAE = serious adverse event.

*Serious adverse events, including death, caused by disease progression as described in Section 8.3.2 should not be reported unless the investigator deems them to be possibly related to study drug.

8.3.2. Primary, Secondary, and Additional Study Endpoint Reporting

The following investigator-reported events are considered potential endpoints and must be reported first as an AE on the AE eCRF (with the appropriate designation for seriousness). They must then be reported as an endpoint on the eCRF with all required source documents provided for adjudication to the CEC. These potential endpoints (even if they meet criteria for a serious event) are not to be reported on the SAE eCRF unless considered as possibly related to study drug, the drug delivery system, or study procedure. Potential endpoints that are serious and considered as possibly related to study drug, the drug delivery system, or study procedure must also be reported as an SAE using the SAE eCRF:

- all-cause mortality (death), and
- HF events.

In the case where 1 of the above endpoint events is reported but does not meet a prespecified event definition detailed in the CEC charter, as reviewed by the independent CEC, no further action will be taken by the study site.

8.3.3. Adverse Events of Special Interest

The following are AESI and will be adjudicated by an external adjudication committee. This committee will be blinded to treatment assignment.

- pancreatitis
- major adverse CV events (see Section 10.5.1.5), and
- deaths

The following are additional AESI for this program that will not be adjudicated by an external committee:

- hepatobiliary disorders
- severe hypoglycemia
- thyroid malignancies and C-cell hyperplasia
- supraventricular arrhythmias and cardiac conduction disorders
- allergic/hypersensitivity reactions, including injection site reactions and ADA formation
- severe GI AEs, and
- acute renal events.

Sites should collect additional details and data regarding AESI, as instructed on the applicable eCRFs, and detailed in Section 10.5.

The details on the definition of AESI will be provided in SAP.

8.4. Treatment of Overdose

Considering the mechanism of action of tirzepatide, potential overdose effects can be GI disorders and hypoglycemia. In the event of overdose, appropriate supportive treatment should be initiated according to the participant's clinical signs and symptoms.
Study drug overdose (defined as injection of study drug more than 1 time within 72 hours) will be reported as an AE.

In the event of an overdose, the investigator should

- contact the medical monitor immediately
- evaluate the participant to determine, in consultation with the medical monitor, whether study intervention should be interrupted or whether the dose should be reduced, and
- closely monitor the participant for any AE/SAE and laboratory abnormalities as medically appropriate.

8.5. Pharmacokinetics

Pharmacokinetic samples will be collected from all participants in this study.

Tirzepatide plasma concentrations will be determined from blood samples obtained from participants receiving tirzepatide treatment. Blood samples collected from participants assigned to the placebo arm will not be included in the bioanalysis of drug concentrations.

Blood samples for PK assessment will be collected prior to the dose administration and at the same time as the planned immunogenicity samples (that is, at Week 0 and then at Weeks 4, 12, 24, and 52 per the Study Schedule or additionally at follow-up and ET (reference SoA).

Samples will be analyzed at a laboratory approved by the sponsor and stored at a facility designated by the sponsor.

Concentrations of tirzepatide will be assayed using a validated liquid chromatography mass spectrometry method. Bioanalytical samples collected to measure tirzepatide concentrations will be retained for a maximum of 1 year following last participant visit for the study (Section 10.1.10). During this time, samples remaining after the bioanalyses may be used for exploratory analyses such as metabolism work, protein binding, and/or bioanalytical method cross-validation.

8.6. Pharmacodynamics

Pharmacodynamic parameters will not be evaluated in this study.

8.7. Genetics

A whole blood sample will be collected for pharmacogenetic analysis where local regulations allow.

See Appendix 2, Clinical Laboratory Tests (Section 10.2), and Section 1.3 (SoA) for sample collection information.

See Section 10.6 for genetic research, custody, and sample retention information.

8.8. Biomarkers

Biomarker research is performed to address questions of relevance to drug disposition, target engagement, pharmacodynamics, mechanism of action, variability of participant response (including safety), and clinical outcome. Sample collection is incorporated into clinical studies to enable examination of these questions through measurement of biomolecules including DNA, RNA, proteins, lipids, and other cellular elements.

Serum and plasma for exploratory biomarker research will be collected at the time specified in the SoA (Section 1.3) where local regulations allow.

All samples will be coded with the participant number. These samples and any data generated can be linked back to the participant only by the investigator site personnel.

Samples will be stored and analysis may be performed on biomarkers thought to play a role in disease processes, mechanism of action of tirzepatide, pathways associated with HFpEF, and/or research methods validating diagnostic tools or assay(s) related to HFpEF and associated diseases. Biomarkers may be evaluated to determine their association with observed clinical responses to tirzepatide and the disease state.

Samples will be retained at a facility selected by the sponsor or its designee for the duration detailed in Section 10.1.10, or for a shorter period if local regulations and ERBs impose shorter time limits. This retention period enables use of new technologies, response to regulatory questions, and investigation of variable response that may not be observed until later in the development of tirzepatide or after tirzepatide becomes commercially available.

8.9. Immunogenicity Assessments

At the visits and times specified in the SoA (Section 1.3), venous blood samples will be collected to determine antibody production against tirzepatide. Antibodies may be further characterized for cross-reactive binding to endogenous counterparts (native GIP and GLP-1) and their ability to neutralize the activity of tirzepatide and endogenous counterparts. To interpret the results of immunogenicity, a venous blood sample will be collected at the same time points to determine the plasma concentrations of tirzepatide. All samples for immunogenicity should be taken predose when applicable and possible.

Samples will be retained for a maximum of 15 years after the last participant visit, or for a shorter period if local regulations and ERBs allow, at a facility selected by the sponsor. The duration allows the sponsor to respond to future regulatory requests related to tirzepatide. Any samples remaining after 15 years will be destroyed.

8.10. Medical Resource Utilization and Health Economics

Not applicable.

9. Statistical Considerations

9.1. Statistical Hypotheses

Two primary hypotheses will be tested in this study:

- Tirzepatide MTD is superior to placebo for the change from baseline to Week 52 in KCCQ-CSS.
- Tirzepatide MTD is superior to placebo for the occurrence of the composite endpoint of CV death and/or HF events over time.

Key secondary hypotheses (all under multiplicity control) are that tirzepatide MTD is superior to placebo with regards to

- change from baseline to Week 52 in 6MWD
- percent change from baseline to Week 52 in body weight, and
- change from baseline to Week 52 in hsCRP.

All primary and key secondary hypotheses will be tested with the overall family-wise type I error rate at a 2-sided alpha level of 0.05 through the multiplicity control approach based on the graphical multiple testing procedure. For the primary hypotheses, the HF outcome will be tested at a 2-sided alpha level of 0.04, and change in KCCQ-CSS will be tested at a 2-sided alpha level of 0.01 in parallel for statistical significance. If significant, the respective alpha of the primary endpoints will be propagated to test the key secondary endpoints. If any of the primary endpoints is not significant, then the appropriate alpha after the key secondary endpoints testing will be recycled to that primary endpoint. The detailed graphical testing scheme will be outlined in the SAP.

9.2. Sample Size Determination

A sample size of 700 participants (350 in each treatment group) will provide roughly 80% power for the change from baseline to Week 52 in KCCQ-CSS using Wilcoxon rank sum test under the assumptions that the change from baseline to Week 52 in KCCQ-CSS follows normal distribution with mean of 5 and standard deviation of 19 in placebo and mean of 10 and standard deviation of 19 in tirzepatide group at a 2-sided alpha of 0.01 significance level. The expected events at the end of the study will provide roughly 80% power to demonstrate the superiority of tirzepatide MTD to placebo in occurrence of the composite endpoint of CV death and/or HF events at a 2-sided alpha of 0.04 significance level under the treatment effect estimate assumption of 0.5. The study power is calculated using nQuery Version 9.1.

9.3. Populations for Analyses

The following populations are defined:

Analysis Population	Description
Entered	All participants who sign the informed consent form
Randomized/Intent-to-Treat (ITT) Population	All participants assigned to treatment, regardless of whether they take any doses of study treatment, or if they took the correct treatment. Participants will be analyzed according to the treatment group to which they were assigned.
Safety Population	All participants in ITT population who take at least 1 dose of study treatment. Participants will be analyzed according to the treatment group to which they were assigned.

9.4. Statistical Analyses

9.4.1. General Considerations

Statistical analysis of this study will be the responsibility of sponsor or its designee.

Unless specified otherwise, all tests of treatment effects will be conducted at a 2-sided alpha level of 0.05 and all confidence intervals will be given at a 2-sided 95% level. Efficacy will be assessed using ITT Population and safety will be assessed using the Safety Population.

Any change to the data analysis methods described in the protocol will require an amendment only if it changes a principal feature of the protocol. Any other change to the data analysis methods described in the protocol, and the justification for making the change, will be described in the SAP and the CSR. Additional exploratory analyses of the data will be conducted as deemed appropriate.

The SAP will be completed prior to first unblinding and any subsequent amendments will be documented, with final amendments finalized prior to final database lock. The SAP will include a more technical and detailed description of the statistical analyses described in this section. This section is a summary of the planned statistical analyses of the most important endpoints including primary and key secondary endpoints.

9.4.2. Primary Endpoint(s)

The primary estimand for primary endpoints is to assess the treatment difference between tirzepatide and placebo relative to the efficacy measures for all randomized participants, and treatment policy strategy will be used to handle all intercurrent events, which is, all the observed values for the variable of interest are used regardless of whether the intercurrent event occurs. The endpoint and population-level summary for the estimand is described in Section 9.4.2.1 and Section 9.4.2.2 for each primary endpoint.

9.4.2.1. Change from Baseline in KCCQ-CSS

For the primary endpoint of change from baseline to Week 52 in KCCQ-CSS, a stratified Wilcoxon (Van Elteren) test will be used as the primary analysis method, controlling for the stratification factors of HF decompensation within 12 months of screening (Y/N), diagnosed T2DM (Y/N), and baseline BMI ≥35 kg/m^2 (Y/N). Population-level summary of Hodges-Lehmann estimate for the median difference and corresponding confidence interval will be reported.

The last measurement prior to randomization KCCQ-CSS will be used as baseline. Missing KCCQ-CSS at Week 52 will be imputed through multiple imputations based on the reason of missingness with details described in the SAP. The statistical inference over multiple imputations will be guided by the method proposed by Rubin (1987).

9.4.2.2. Occurrence of CV Death and/or HF Event over time

The primary analysis model will include fixed factors of treatment and the stratification factors of HF decompensation within 12 months of screening (Y/N), diagnosed T2D (Y/N), and baseline BMI ≥35 kg/m^2 (Y/N). The analysis model with a full list of covariates will be specified in the SAP. The censoring date for a participant is the date of participant's end of follow-up. The missing data due to censoring will be implicitly handled by the model, assuming censoring is independent of the outcome. The treatment effect estimate, with its 95% CI and p-value, will be provided using the primary analysis model.

The estimated cumulative event curve over time will be provided. Counts and proportions of participants who experience a primary endpoint event will be calculated as well as counts of primary endpoint events. The total person-years of follow-up, the incidence rate per 100 person-years of follow-up, and the absolute risk difference will be provided.

9.4.3. Key Secondary Endpoint(s)

Analyses for the key secondary endpoints will also be guided by the treatment policy strategy.

Change from baseline in 6MWD at Week 52 will be analyzed using the same nonparametric approach as described in Section 9.4.2.1.

Percent change from baseline in body weight will be analyzed using an analysis of covariance (ANCOVA) analysis. The ANCOVA model will include the categorical effect of treatment, stratification factors, and the continuous covariate of baseline body weight value. Missing data will be imputed through multiple imputations based on the reason of missingness with details described in the SAP.

Change from baseline in hsCRP will be analyzed using an ANCOVA model. The ANCOVA model will include the categorical effect of treatment, stratification factors, and the continuous covariate of baseline hsCRP value. The ANCOVA model will be based on the log-transformed values of hsCRP. Missing data will be imputed through multiple imputations based on the reason of missingness with details described in the SAP.

9.4.4. Tertiary/Exploratory Endpoint(s)

The analyses for exploratory endpoints will be described in the SAP. Statistical tests will be performed at the two-sided significance level of 0.05. There will be no multiplicity adjustment for any analysis of exploratory variables unless specified otherwise. Missing values will not be explicitly imputed unless specified otherwise.

9.4.5. Other Safety Analyses

Safety will be assessed by summarizing and analyzing AEs, special safety topics, laboratory analytes, and vital signs. All safety analyses will be made on the Safety Population. Unless specified otherwise, all data obtained during study period from Safety Population, regardless of adherence to study drug, will be used for safety analyses. The details for safety analysis will be described in the SAP.

Adverse events will be coded from the actual term using MedDRA and reported with preferred terms and system organ class.

9.4.5.1. Evaluation of Immunogenicity

The frequency and percentage of participants with preexisting ADAs and with treatment-emergent ADAs to tirzepatide will be tabulated. Treatment-emergent ADAs are defined as those with a titer 2-fold (1 dilution) greater than the minimum required dilution of the ADA assay if no ADAs were detected at baseline (treatment-induced ADA), or those with a 4-fold (2 dilutions) increase in titer compared with baseline if ADAs were detected at baseline (treatment-boosted ADA). The details of analyses for immunogenicity will be specified in SAP.

9.4.6. Subgroup Analyses

Subgroup variables to be evaluated for the primary efficacy endpoint may include demography (for example, race, ethnicity), baseline disease characteristics (for example, diagnosed T2DM) and others. Subgroup analyses may also be performed for selected secondary efficacy endpoints. Details for the subgroup analyses will be provided in the SAP.

9.5. Interim Analyses

Based on the projected enrollment, approximately 4 interim analyses of safety will be conducted. The first interim analysis is planned to occur when approximately 20% of the anticipated number of participants are randomized or 6 months after the first participant is randomized, whichever occurs later, followed by subsequent reviews approximately every 6 months throughout the study.

The DMC is authorized to evaluate unblinded interim analyses. Study sites will receive information about interim results only if they need to know for the safety of their participants.

Unblinding details are specified in a separate unblinding plan document.

The DMC charter will describe the planned interim analyses in detail.

9.6. Data Monitoring Committee

An independent DMC with members all external to the sponsor will be used to monitor participant safety in an unblinded fashion. For details on the DMC, refer to the DMC charter.

10. Supporting Documentation and Operational Considerations

10.1. Appendix 1: Regulatory, Ethical, and Study Oversight Considerations

10.1.1. Regulatory and Ethical Considerations

- This study will be conducted in accordance with the protocol and with the following:
  - Consensus ethical principles derived from international guidelines, including the Declaration of Helsinki and CIOMS International Ethical Guidelines
  - Applicable ICH GCP guidelines
  - International Organization for Standardization (ISO) 14155
  - Applicable laws and regulations
- The protocol, protocol amendments, ICF, IB, and other relevant documents (for example, advertisements) must be submitted to an IRB/IEC by the investigator and reviewed and approved by the IRB/IEC before the study is initiated.
- Any amendments to the protocol will require IRB/IEC approval before implementation of changes made to the study design, except for changes necessary to eliminate an immediate hazard to study participants.
- Protocols and any substantial amendments to the protocol will require health authority approval prior to initiation except for changes necessary to eliminate an immediate hazard to study participants.
- The investigator will be responsible for the following:
  - Providing written summaries of the status of the study to the IRB/IEC annually or more frequently in accordance with the requirements, policies, and procedures established by the IRB/IEC
  - Notifying the IRB/IEC of SAEs or other significant safety findings as required by IRB/IEC procedures
  - Providing oversight of study conduct for participants under their responsibility and adherence to requirements of 21 CFR, ICH guidelines, the IRB/IEC, European regulation 536/2014 for clinical studies (if applicable), and all other applicable local regulations
- Investigator sites are compensated for participation in the study as detailed in the clinical trial agreement.

10.1.2. Financial Disclosure

Investigators and sub-investigators will provide the sponsor with sufficient, accurate financial information as requested to allow the sponsor to submit complete and accurate financial certification or disclosure statements to the appropriate regulatory authorities. Investigators are

responsible for providing information on financial interests during the course of the study and for 1 year after completion of the study.

10.1.3. Informed Consent Process

The investigator or his/her representative will explain the nature of the study, including the risks and benefits, to the participant or his/her legally authorized representative and answer all questions regarding the study.

Participants must be informed that their participation is voluntary. Participants or their legally authorized representative will be required to sign a statement of informed consent that meets the requirements of 21 CFR 50, local regulations, ICH guidelines, HIPAA requirements, where applicable, and the IRB/IEC or study center.

The medical record must include a statement that written informed consent was obtained before the participant was entered in the study and the date the written consent was obtained. The authorized person obtaining the informed consent must also sign the ICF.

Participants must be re-consented to the most current version of the ICF(s) during their participation in the study.

A copy of the ICF(s) must be provided to the participant or the participant’s legally authorized representative and is kept on file.

Participants who are rescreened are required to sign a new ICF.

10.1.4. Data Protection

Participants will be assigned a unique identifier by the sponsor. Any participant records, datasets, or tissue samples that are transferred to the sponsor will contain the identifier only; participant names or any information which would make the participant identifiable will not be transferred.

The participant must be informed that the participant’s personal, study-related data will be used by the sponsor in accordance with local data protection law. The level of disclosure must also be explained to the participant who will be required to give consent for his/her data to be used as described in the informed consent.

The participant must be informed that their medical records may be examined by Clinical Quality Assurance auditors or other authorized personnel appointed by the sponsor, by appropriate IRB/IEC members, and by inspectors from regulatory authorities.

The sponsor has processes in place to ensure data protection, information security, and data integrity. These processes include appropriate contingency plan(s) for appropriate and timely response in the event of a data security breach.

10.1.5. Dissemination of Clinical Study Data

Report Preparation

An investigator will sign the final CSR for this study, indicating agreement that, to the best of his or her knowledge, the report accurately describes the conduct and results of the study.

Public Access to Reports and Data

Reports

The sponsor will disclose a summary of study information, including tabular study results, on publicly available websites where required by local law or regulation.

Data

The sponsor provides access to all individual participant data collected during the trial, after anonymization, with the exception of PK, immunogenicity, or genetic data. Data are available to request 6 months after the indication studied has been approved in the United States and after primary publication acceptance, whichever is later. No expiration date of data requests is currently set once they are made available. Access is provided after a proposal has been approved by an independent review committee identified for this purpose and after receipt of a signed data sharing agreement. Data and documents, including the study protocol, SAP, CSR, and blank or annotated CRFs, will be provided in a secure data sharing environment for up to 2 years per proposal. For details on submitting a request, see the instructions provided at www.clinicalstudydatarequest.com.

Publications/Publication Policy

The publication policy is described in Section 10.1.9.

10.1.6. Data Quality Assurance

All participant data relating to the study will be recorded on printed or electronic CRF unless transmitted to the sponsor or designee electronically (for example, laboratory data or an electronic source, such as eCOA). The investigator is responsible for verifying that data entries are accurate and correct by physically or electronically signing the CRF.

The investigator must maintain accurate documentation (source data) that supports the information entered in the CRF.

The investigator must permit study-related monitoring, audits, IRB/IEC review, and regulatory agency inspections and provide direct access to source data documents.

Monitoring details describing strategy (for example, risk-based initiatives in operations and quality such as risk management and mitigation strategies and analytical risk-based monitoring), methods, responsibilities, and requirements, including handling of noncompliance issues and monitoring techniques, are provided in the Monitoring Plan.

The sponsor or designee is responsible for the data management of this study including quality checking of the data.

The sponsor assumes accountability for actions delegated to other individuals (for example, contract research organizations).

Study monitors will perform ongoing source data verification to confirm that data transcribed into the CRF by authorized site personnel are accurate, complete, and verifiable from source documents; that the safety and rights of participants are being protected; and that the study is being conducted in accordance with the currently approved protocol and any other study agreements, ICH GCP, and all applicable regulatory requirements.

Records and documents, including signed ICFs, pertaining to the conduct of this study must be retained by the investigator for the time period outlined in the clinical trial agreement unless local regulations or institutional policies require a longer retention period. No records may be destroyed during the retention period without the written approval of the sponsor. No records may be transferred to another location or party without written notification to the sponsor.

In addition, the sponsor or its representatives will periodically check a sample of the participant data recorded against source documents at the study site. The study may be audited by the sponsor or its representatives, and/or regulatory agencies at any time. Investigators will be given notice before an audit occurs.

Data Capture System

The investigator is responsible for ensuring the accuracy, completeness, legibility, and timeliness of the data reported to the sponsor.

An EDC system will be used in this study for the collection of CRF data. The investigator maintains a separate source for the data entered by the investigator or designee into the sponsor-provided EDC system. The investigator is responsible for the identification of any data to be considered source and for the confirmation that data reported are accurate and complete by signing the CRF.

Additionally, eCOA data (patient-reported outcomes instruments) will be directly recorded by the participant, into a device (for example, hand-held smart phone or tablet). The eCOA data will serve as the source documentation, and the investigator does not maintain a separate, written or electronic record of these data.

Data collected via the sponsor-provided data capture systems will be stored at third parties. The investigator will have continuous access to the data during the study and until decommissioning of the data capture system(s). Prior to decommissioning, the investigator will receive an archival copy of pertinent data for retention.

Data managed by a central vendor, such as laboratory test data, will be stored electronically in the central vendor's database system, and reports (as applicable) will be provided to the investigator for review and retention. Data will subsequently be transferred from the central vendor to the sponsor data warehouse.

Data from complaint forms submitted to the sponsor will be encoded and stored in the global product complaint management system.

10.1.7. Source Documents

Source documents provide evidence for the existence of the participant and substantiate the integrity of the data collected. Source documents are filed at the investigator's site.

Data reported on the CRF or entered in the eCRF that are transcribed from source documents must be consistent with the source documents, or the discrepancies must be explained. The investigator may need to request previous medical records or transfer records, depending on the study. Also, current medical records must be available.

Definition of what constitutes source data can be found in Section 10.1.6.

10.1.8. Study and Site Start and Closure

The study start date is the date on which the clinical study will be open for recruitment of participants.

The first act of recruitment is the first site open and will be the study start date.

The sponsor designee reserves the right to close the study site or terminate the study at any time for any reason at the sole discretion of the sponsor. Study sites will be closed upon study completion. A study site is considered closed when all required documents and study supplies have been collected and a study-site closure visit has been performed.

The investigator may initiate study-site closure at any time, provided there is reasonable cause and sufficient notice is given in advance of the intended termination.

Reasons for the early closure of a study site by the sponsor or investigator may include but are not limited to:

- Failure of the investigator to comply with the protocol, the requirements of the IRB/IEC or local health authorities, the sponsor's procedures, or GCP guidelines
- Inadequate recruitment of participants by the investigator
- Discontinuation of further study drug development.

If the study is prematurely terminated or suspended, the sponsor shall promptly inform the investigators, the IECs/IRBs, the regulatory authorities, and any contract research organization(s) used in the study of the reason for termination or suspension, as specified by the applicable regulatory requirements. The investigator shall promptly inform the participant and assures appropriate participant therapy and/or follow-up.

10.1.9. Publication Policy

In accordance with the sponsor's publication policy, the results of this study will be submitted for publication by a peer-reviewed journal.

10.1.10. Long-Term Sample Retention

Sample retention enables use of new technologies, response to regulatory questions, and investigation of variable response that may not be observed until later in the development of the intervention or after the intervention becomes commercially available.

This table describes the retention period for potential sample types.

Sample Type	Custodian	Retention Period After Last Participant Visit
Genetics sample	Sponsor or designee	up to 15 years
Exploratory biomarker sample	Sponsor or designee	up to 15 years
Immunogenicity (antidrug antibody) sample	Sponsor or designee	up to 15 years
Pharmacokinetic sample	Sponsor or designee	up to 1 years

10.1.11. Investigator Information

Researchers with appropriate education, training, and experience, as determined by the Sponsor, will participate as investigators in this clinical trial

10.2. Appendix 2: Clinical Laboratory Tests

- Clinical laboratory testing will be performed according to the SoA (Section 1.3).
- Central and local laboratories will be used. The table below describes when the local or central laboratory will be used
- In circumstances where the sponsor approves local laboratory testing in lieu of central laboratory testing (in the table below), the local laboratory must be qualified in accordance with applicable local regulations.
- Protocol-specific requirements for inclusion or exclusion of participants are detailed in Section 5 of the protocol.
- Additional tests may be performed at any time during the study as determined necessary by the investigator or required by local regulations.
- Pregnancy testing will be performed according to the SoA.
- Investigators must document their review of the laboratory safety results. Laboratory results that will not be reported to investigative sites or other blinded personnel are noted in the table below.

Refer to Section 10.7 for recommended laboratory testing for hypersensitivity events.

Clinical Laboratory Tests	Comments
**Hematology**	Assayed by Lilly-designated laboratory.
Hemoglobin	
Hematocrit	
Erythrocyte count (RBCs [red blood cells])	
Mean cell volume	
Mean cell hemoglobin concentration	
Leukocytes (WBCs [white blood cells])	
Differential	
Neutrophils, segmented	
Lymphocytes	
Monocytes	
Eosinophils	
Basophils	
Platelets	
**Clinical Chemistry**	Assayed by Lilly-designated laboratory.
Sodium	
Potassium	
Chloride	
Bicarbonate	

Clinical Laboratory Tests	Comments
Total bilirubin	
Direct bilirubin	
Alkaline phosphatase (ALP)	
Alanine aminotransferase (ALT)	
Aspartate aminotransferase (AST)	
Gamma-glutamyl transferase (GGT)	
Blood urea nitrogen (BUN)	
Creatinine	
Creatine kinase (CK)	
Uric acid	
Total protein	
Albumin	
Calcium	
Phosphorus	
Glucose	
**Lipids**	
Total cholesterol	
Direct LDL-C	
HDL-C	
VLDL-C	
Triglycerides	
**Pancreas (Exocrine)**	Assayed by Lilly-designated laboratory.
Pancreatic amylase	
Lipase	
**Special Chemistry**	Assayed by Lilly-designated laboratory.
Hemoglobin A1c (HbA1c)	
Calcitonin	
Cystatin C	
N-terminal pro b-type natriuretic peptide (NT-proBNP)	
Cardiac troponin T (cTnT)	
C-reactive protein, high-sensitivity (hsCRP)	
Thyroid stimulating hormone	
**Urine Chemistry**	Assayed by Lilly-designated laboratory.
Albumin	
Creatinine	
**Calculation**	
eGFR (calculated by CKD-EPI equation)	Will be calculated by the Lilly-designated laboratory at all visits.
Urine albumin, creatinine, UACR	
**Hormones (female)**	
Urine Pregnancy	Local laboratory
Serum Pregnancy	Assayed by Lilly-designated laboratory.

Clinical Laboratory Tests	Comments
Follicle Stimulating Hormone (FSH)	Assayed by Lilly-designated laboratory.
**Pharmacokinetic Samples**	• Assayed by Lilly-designated laboratory.<br>• Results will not be provided to the investigative sites.<br>• In the event of systemic drug hypersensitivity reactions (immediate or nonimmediate), additional blood samples will be collected including ADA, PK, and exploratory biomarker samples. PK samples for immunogenicity must be taken prior to drug administration.
**Genetics sample**<br>Whole blood (EDTA)	• Assayed by Lilly-designated laboratory.<br>• Results will not be provided to the investigative sites.
**Exploratory Biomarker Samples**	• Assayed by Lilly-designated laboratory.<br>• Results will not be provided to the investigative sites.
Serum	
EDTA Plasma	
P800 Plasma	
**Immunogenicity Samples**	
Anti-tirzepatide antibodies<br>Anti-tirzepatide neutralizing antibodies	• Assayed by Lilly-designated laboratory.<br>• Results will not be provided to the investigative sites.<br>• In the event of systemic drug hypersensitivity reactions (immediate or nonimmediate), additional blood samples will be collected including ADA, PK, and exploratory biomarker samples. PK samples for immunogenicity must be taken prior to drug administration.

Abbreviations: ADA = antidrug antibody; CKD-EPI = Chronic Kidney Disease-Epidemiology Collaboration; EDTA = ethylenediaminetetraacetic acid; eGFR = estimated glomerular filtration rate; HDL-C = high-density lipoprotein cholesterol; LDL-C = low-density lipoprotein cholesterol; Lilly = Eli Lilly and company; PK = pharmacokinetic; UACR = urine albumin/creatinine ratio; VLDL-C = very low-density lipoprotein cholesterol.

10.3. Appendix 3: Adverse Events: Definitions and Procedures for Recording, Evaluating, Follow-up, and Reporting

The definitions and procedures detailed in this appendix are in accordance with ISO 14155.

Both the investigator and the sponsor will comply with all local medical device reporting requirements.

The detection and documentation procedures described in this protocol apply to all sponsor medical devices provided for use in the study. See Section 6.1.1 for the list of sponsor medical devices).

10.3.1. Definition of AE

AE Definition
• An AE is any untoward medical occurrence in a participant administered a pharmaceutical product and which does not necessarily have a causal relationship with the study drug. An AE can therefore be any unfavorable and unintended sign (including an abnormal laboratory finding), symptom, or disease (new or exacerbated) temporally associated with the use of a medicinal (investigational) product, whether or not related to the medicinal (investigational) product.<br>• An AE is any untoward medical occurrence, unintended disease or injury, or untoward clinical signs (including abnormal laboratory finding) in study participants, users, or other persons, whether or not related to the investigational medical device. This definition includes events related to the investigational medical device or comparator and events related to the procedures involved except for events in users or other persons, which only include events related to investigational devices.
**Events Meeting the AE Definition**
• Any abnormal laboratory test results (hematology, clinical chemistry, or urinalysis) or other safety assessments (for example, ECG, radiological scans, vital signs measurements), including those that worsen from baseline, considered clinically significant in the medical and scientific judgment of the investigator (that is, not related to progression of underlying disease).<br>• Exacerbation of a chronic or intermittent preexisting condition including either an increase in frequency and/or intensity of the condition.<br>• New conditions detected or diagnosed after study drug administration even though they may have been present before the start of the study.<br>• Signs, symptoms, or the clinical sequelae of a suspected drug-drug interaction.<br>• Signs, symptoms, or the clinical sequelae of a suspected overdose of either study drug or a concomitant medication. Overdose per se will not be reported as an AE/SAE unless it is an intentional overdose taken with possible suicidal/self-harming intent. Such an overdose should be reported regardless of sequelae.<br>• "Lack of efficacy" or "failure of expected pharmacological action" per se will not be reported as an AE or SAE. Such instances will be captured in the efficacy assessments.

However, the signs, symptoms, and/or clinical sequelae resulting from lack of efficacy will be reported as an AE or SAE if they fulfill the definition of an AE or SAE.
**Events NOT Meeting the AE Definition**
• Any clinically significant abnormal laboratory findings or other abnormal safety assessments which are associated with the underlying disease, unless judged by the investigator to be more severe than expected for the participant's condition.<br>• The disease/disorder being studied or expected progression, signs, or symptoms of the disease/disorder being studied, unless more severe than expected for the participant's condition.<br>• Medical or surgical procedure (for example, endoscopy, appendectomy): the condition that leads to the procedure is the AE.<br>• Situations in which an untoward medical occurrence did not occur (social and/or convenience admission to a hospital).<br>• Anticipated day-to-day fluctuations of preexisting disease(s) or condition(s) present or detected at the start of the study that do not worsen.

10.3.2. Definition of SAE

If an event is not an AE per the definition above, then it cannot be an SAE even if serious conditions are met (for example, hospitalization for signs/symptoms of the disease under study, death due to progression of disease).

**SAE is defined as any untoward medical occurrence that, at any dose:**
**a. Results in death**
**b. Is life-threatening**<br>The term "life-threatening" in the definition of "serious" refers to an event in which the participant was at risk of death at the time of the event. It does not refer to an event, which hypothetically might have caused death, if it were more severe.
**c. Requires inpatient hospitalization or prolongation of existing hospitalization**<br>• In general, hospitalization signifies that the participant has been admitted to the hospital for observation and/or treatment that would not have been appropriate in the physician's office or outpatient setting. Complications that occur during hospitalization are AEs. If a complication prolongs hospitalization or fulfills any other serious criteria, the event is serious. When in doubt as to whether "hospitalization" occurred or was necessary, the AE should be considered serious.<br>• Hospitalization for elective treatment of a preexisting condition that did not worsen from baseline is not considered an AE.

d. Results in persistent disability/incapacity

- The term disability means a substantial disruption of a person's ability to conduct normal life functions.
- This definition is not intended to include experiences of relatively minor medical significance such as uncomplicated headache, nausea, vomiting, diarrhea, influenza, and accidental trauma (for example, sprained ankle) which may interfere with or prevent everyday life functions but do not constitute a substantial disruption.

e. Is a congenital anomaly/birth defect

- Abnormal pregnancy outcomes (for example, spontaneous abortion, fetal death, stillbirth, congenital anomalies, ectopic pregnancy) are considered SAEs.

f. Other situations:

- Medical or scientific judgment should be exercised in deciding whether SAE reporting is appropriate in other situations such as important medical events that may not be immediately life-threatening or result in death or hospitalization but may jeopardize the participant or may require medical or surgical intervention to prevent one of the other outcomes listed in the above definition. These events should usually be considered serious.
- Examples of such events include invasive or malignant cancers; intensive treatment in an emergency room or at home for allergic bronchospasm, blood dyscrasias, or convulsions that do not result in hospitalization; or development of drug dependency or drug abuse.

g. Resulted in medical or surgical intervention to prevent life-threatening illness or injury or permanent impairment to a body structure or a body function.

Definition of Serious Adverse Device Effect (SADE)

An SADE is defined as an adverse device effect that has resulted in any of the consequences characteristic of a serious adverse event.

Definition of Unanticipated Adverse Device Effect (UADE)

An UADE is a serious adverse effect on health or safety or any life-threatening problem or death caused by or associated with a device, if that effect, problem, or death was not previously identified in nature, severity, or degree of incidence in the investigational plan or application (including a supplementary plan or application) or any other unanticipated serious problem associated with a device that relates to the rights, safety, or welfare of the participant.

10.3.3. Definition of Product Complaints

Product Complaint
• A product complaint is any written, electronic, or oral communication that alleges deficiencies related to the identity, quality, durability, reliability, safety, effectiveness, or performance of a study drug. When the ability to use the study drug safely is impacted, the following are also product complaints:<br>o Deficiencies in labeling information, and<br>o Use errors for device or drug-device combination products due to ergonomic design elements of the product.<br>• Product complaints related to study drugs used in clinical trials are collected in order to ensure the safety of participants, monitor quality, and to facilitate process and product improvements.<br>• Investigators will instruct participants to contact the site as soon as possible if he or she has a product complaint or problem with the study drug so that the situation can be assessed.<br>• An event may meet the definition of both a product complaint and an AE/SAE. In such cases, it should be reported as both a product complaint and as an AE/SAE.

10.3.4. Recording and Follow-Up of AE and/or SAE and Product Complaints

AE, SAE, and Product Complaint Recording
When an AE/SAE/product complaint occurs, it is the responsibility of the investigator to review all documentation (for example, hospital progress notes, laboratory reports, and diagnostics reports) related to the event.<br>The investigator will then record all relevant AE/SAE/product complaint information in the participant's medical records, in accordance with the investigator's normal clinical practice. AE/SAE information is reported on the appropriate eCRF page and product complaint information is reported on the Product Complaint Form.<br>Note: An event may meet the definition of both a product complaint and an AE/SAE. In such cases, it should be reported as both a product complaint and as an AE/SAE.<br>It is **not** acceptable for the investigator to send photocopies of the participant's medical records to sponsor or designee in lieu of completion of the eCRF page for AE/SAE and the Product Complaint Form for product complaints.<br>There may be instances when copies of medical records for certain cases are requested by sponsor or designee. In this case, all participant identifiers, with the exception of the participant number, will be redacted on the copies of the medical records before submission to sponsor or designee.<br>The investigator will attempt to establish a diagnosis of the event based on signs, symptoms, and/or other clinical information. Whenever possible, the diagnosis (not the individual signs/symptoms) will be documented as the AE/SAE.

**Assessment of Intensity**
The investigator will make an assessment of intensity for each AE and SAE reported during the study and assign it to one of the following categories:<br>• Mild: A type of adverse event that is usually transient and may require only minimal treatment or therapeutic intervention. The event does not generally interfere with usual activities of daily living.<br>• Moderate: A type of adverse event that is usually alleviated with additional specific therapeutic intervention. The event interferes with usual activities of daily living, causing discomfort but poses no significant or permanent risk of harm to the research participant.<br>• Severe: A type of adverse event that interrupts usual activities of daily living, or significantly affects clinical status, or may require intensive therapeutic intervention. An AE that is assessed as severe should not be confused with an SAE. Severe is a category utilized for rating the intensity of an event; and both AEs and SAEs can be assessed as severe.<br>An event is defined as 'serious' when it meets at least 1 of the predefined outcomes as described in the definition of an SAE, NOT when it is rated as severe.
**Assessment of Causality**
• The investigator is obligated to assess the relationship between study drug and each occurrence of each AE/SAE.<br>• A "reasonable possibility" of a relationship conveys that there are facts, evidence, and/or arguments to suggest a causal relationship, rather than a relationship cannot be ruled out.<br>• The investigator will use clinical judgment to determine the relationship.<br>• Alternative causes, such as underlying disease(s), concomitant therapy, and other risk factors, as well as the temporal relationship of the event to study drug administration will be considered and investigated.<br>• The investigator will also consult the IB in his/her assessment.<br>• For each AE/SAE, the investigator **must** document in the medical notes that he/she has reviewed the AE/SAE and has provided an assessment of causality.<br>• There may be situations in which an SAE has occurred, and the investigator has minimal information to include in the initial report to sponsor or designee. However, it is very important that the investigator always make an assessment of causality for every event before the initial transmission of the SAE data to sponsor or designee.<br>• The investigator may change his/her opinion of causality in light of follow-up information and send an SAE follow-up report with the updated causality assessment.<br>• The causality assessment is one of the criteria used when determining regulatory reporting requirements.

Follow-up of AEs and SAEs
• The investigator is obligated to perform or arrange for the conduct of supplemental measurements and/or evaluations as medically indicated or as requested by sponsor or designee to elucidate the nature and/or causality of the AE or SAE as fully as possible. This may include additional laboratory tests or investigations, histopathological examinations, or consultation with other health care professionals.

10.3.5. Reporting of SAEs

SAE Reporting via an Electronic Data Collection Tool
• The primary mechanism for reporting an SAE will be the electronic data collection tool.<br>• If the electronic system is unavailable, then the site will use the paper SAE data collection tool (see next section) in order to report the event within 24 hours.<br>• The site will enter the SAE data into the electronic system as soon as it becomes available.<br>• After the study is completed at a given site, the electronic data collection tool will be taken offline to prevent the entry of new data or changes to existing data.<br>• If a site receives a report of a new SAE from a study participant or receives updated data on a previously reported SAE after the electronic data collection tool has been taken off-line, then the site can report this information on a paper SAE form (see next section) or to the sponsor by telephone.<br>• Contacts for SAE reporting can be found in the study training.

10.3.6. Regulatory Reporting Requirements

SAE Regulatory Reporting
• Prompt notification by the investigator to the sponsor of an SAE is essential so that legal obligations and ethical responsibilities towards the safety of participants and the safety of a study drug under clinical investigation are met.<br>• The sponsor has a legal responsibility to notify both the local regulatory authority and other regulatory agencies about the safety of a study drug under clinical investigation. The sponsor will comply with country-specific regulatory requirements relating to safety reporting to the regulatory authority, Institutional Review Boards (IRB)/Independent Ethics Committees (IEC), and investigators.<br>• An investigator who receives an investigator safety report describing an SAE or other specific safety information (for example, summary or listing of SAEs) from the sponsor will review and then file it along with the IB and will notify the IRB/IEC, if appropriate according to local requirements.<br>• As required by local regulations, the investigator will report to their IRB/IEC any UADE (unanticipated problem that resulted in an SAE), or any product complaint that could have led to an SAE had precautions not been taken.

10.4. Appendix 4: Contraceptive Guidance and Collection of Pregnancy Information

10.4.1. Male participants:

Men, regardless of their fertility status, with nonpregnant WOCBP partners must agree to either remain abstinent (if this is their preferred and usual lifestyle) or use condoms, as well as 1 additional highly effective (<1% failure rate) method of contraception (such as combination oral contraceptives, implanted contraceptives, or intrauterine devices) or effective method of contraception (such as diaphragms with spermicide or cervical sponges) for the duration of the study and until their plasma concentrations are below the level that could result in a relevant potential exposure to a possible fetus, predicted to be 90 days plus 5 half-lives following the last dose of study drug, which is approximately 4 months after the last injection.

a) Men and their partners may choose to use a double-barrier method of contraception. (Barrier protection methods without concomitant use of a spermicide are not an effective or acceptable method of contraception. Thus, each barrier method must include use of a spermicide. It should be noted, however, that the use of male and female condoms as a double-barrier method is not considered acceptable due to the high failure rate when these barrier methods are combined.)

b) Periodic abstinence (for example, calendar, ovulation, symptothermal, or postovulation methods), declaration of abstinence just for the duration of a trial, and withdrawal are not acceptable methods of contraception.

Men with pregnant partners should use condoms during intercourse for the duration of the study and until the end of the estimated, relevant potential exposure in WOCBP (4 months).

Men should refrain from sperm donation for the duration of the study and until their plasma concentrations are below the level that could result in a relevant potential exposure to a possible fetus, predicted to be 90 days plus 5 half-lives following the last dose of study drug, which is approximately 4 months.

Men who are in exclusively same-sex relationships (as their preferred and usual lifestyle) are not required to use contraception.

10.4.2. Female participants:

Women of childbearing potential who are abstinent (if this is complete abstinence, as their preferred and usual lifestyle) or in a same-sex relationship (as part of their preferred and usual lifestyle) must agree to either remain abstinent or stay in a same-sex relationship without sexual relationships with males. Periodic abstinence (for example, calendar, ovulation, symptothermal, or postovulation methods), declaration of abstinence just for the duration of a trial, and withdrawal are not acceptable methods of contraception.

Otherwise, WOCBP participating must agree to use 2 forms of effective contraception, where at least 1 form is highly effective (<than 1% failure rate), for the entirety of the study. Contraception must continue following completion of study drug administration for the entirety of the study and for 4 weeks after the last injection.

a) WOCBP participating must test negative for pregnancy prior to initiation of treatment as indicated by a negative serum pregnancy test at the screening visit followed by a negative urine pregnancy test within 24 hours prior to exposure.

b) Two forms of effective contraception, where at least 1 form is highly effective (such as combination oral contraceptives, implanted contraceptives, or intrauterine devices) will be used for the duration of the trial and for 2 months after the last injection. Effective contraception (such as male or female condoms with spermicide, diaphragms with spermicide, or cervical sponges) may be used as the second therapy. Barrier protection methods without concomitant use of a spermicide are not a reliable or acceptable method. Thus, each barrier method must include use of a spermicide (that is, condom with spermicide, diaphragm with spermicide, or female condom with spermicide). It should be noted that the use of male and female condoms as a double-barrier method is not considered acceptable due to the high failure rate when these methods are combined.

c) Not be breastfeeding.

Women not of childbearing potential may participate and include those who are:

a) Infertile due to surgical sterilization, or

b) Postmenopausal.

Definitions:

Woman of Childbearing Potential (WOCBP)

A woman is considered fertile following menarche and until becoming postmenopausal unless permanently sterile (see below).

If fertility is unclear (for example, amenorrhea in adolescents or athletes) and a menstrual cycle cannot be confirmed before first dose of study drug, additional evaluation should be considered.

Women in the following categories are not considered WOCBP

1. Premenarchal
2. Premenopausal female with 1 of the following:
    - Documented hysterectomy
    - Documented bilateral salpingectomy
    - Documented bilateral oophorectomy

For individuals with permanent infertility due to an alternate medical cause other than the above, (for example, mullerian agenesis, androgen insensitivity), investigator discretion should be applied to determining study entry.

Note: Determination can come from the site personnel’s review of the participant’s medical records, medical examination, or medical history interview.

3. Postmenopausal female is defined as follows:
    a. A woman at any age at least 6 weeks post-surgical bilateral oophorectomy with or without hysterectomy, confirmed by operative note
    b. A woman at least 40 years of age and up to 55 years old with an intact uterus, not on hormone therapy*, who has had cessation of menses for at least 12 consecutive

months without an alternative medical cause, AND with a follicle-stimulating hormone >40 mIU/mL; or

c. A woman 55 or older not on hormone therapy, who has had at least 12 months of spontaneous amenorrhea, or
d. A woman at least 55 years of age with a diagnosis of menopause prior to starting hormone replacement therapy.

* Women should not be taking medications during amenorrhea such as oral contraceptives, hormones, gonadotropin-releasing hormone, anti-estrogens, selective estrogen receptor modulators (SERMs), or chemotherapy that could induce transient amenorrhea.

Contraception Guidance:

Highly Effective Methods of Contraception:

- Combined oral contraceptive pill and mini pill
- NuvaRing®
- Implantable contraceptives
- Injectable contraceptives (such as Depo-Provera®)
- Intrauterine device (such as Mirena® and ParaGard®)
- Contraceptive patch – ONLY women <198 pounds or 90 kg
- Total abstinence (if this is their preferred and usual lifestyle) or in a same-sex relationship with no sexual relationship with males (as part of their preferred and usual lifestyle).

Note: periodic abstinence (for example, calendar, ovulation, symptothermal, postovulation methods), declaration of abstinence just for the duration of a trial, and withdrawal are not acceptable methods of contraception.

Note: Implantable contraceptives and injectable contraceptives (such as Depo-Provera) are only permitted if started prior to screening. Participants should not start these methods of contraception after being enrolled in the study.

- Vasectomy - for men in clinical trials

Effective Methods of Contraception (must use combination of 2 methods):

- Male condom with spermicide
- Female condom with spermicide
- Diaphragm with spermicide
- Cervical sponge
- Cervical cap with spermicide

Collection of Pregnancy Information

Male participants with partners who become pregnant

- The investigator will attempt to collect pregnancy information on any male participant's female partner who becomes pregnant while the male participant is in this study.
- After obtaining the necessary signed informed consent from the pregnant female partner directly, the investigator will record pregnancy information on the appropriate form and

submit it to the sponsor within 24 hours of learning of the partner's pregnancy. The female partner will also be followed to determine the outcome of the pregnancy. Information on the status of the mother and child will be forwarded to the sponsor. Generally, the follow-up will be no longer than 6 to 8 weeks following the estimated delivery date. Any termination of the pregnancy will be reported regardless of fetal status (presence or absence of anomalies) or indication for the procedure.

Female participants who become pregnant

- The investigator will collect pregnancy information on any female participant who becomes pregnant while participating in this study. The initial information will be recorded on the appropriate form and submitted to the sponsor within 24 hours of learning of a participant's pregnancy.
- The participant will be followed to determine the outcome of the pregnancy. The investigator will collect follow-up information on the participant and the neonate, and the information will be forwarded to the sponsor. Generally, follow-up will not be required for longer than 6 to 8 weeks beyond the estimated delivery date. Any termination of pregnancy will be reported, regardless of fetal status (presence or absence of anomalies) or indication for the procedure.
- While pregnancy itself is not considered to be an AE or SAE, any pregnancy complication or elective termination of a pregnancy for medical reasons will be reported as an AE or SAE.
- A spontaneous abortion (occurring at <20 weeks gestational age) or still birth (occurring at >20 weeks gestational age) is always considered to be an SAE and will be reported as such.
- Any poststudy, pregnancy-related SAE considered reasonably related to the study drug by the investigator will be reported to the sponsor as described in protocol Section 8.3.1. While the investigator is not obligated to actively seek this information in former study participants, he or she may learn of an SAE through spontaneous reporting.
- Any female participant who becomes pregnant while participating in the study will discontinue study drug and be withdrawn from the study.

10.5. Appendix 5: Adverse Events of Special Interest: Definitions and Procedures for Recording, Evaluating, Follow-Up, and Reporting.

10.5.1. Special Safety Topics

10.5.1.1. Hypoglycemia

Upon ICF signing, all participants will be educated about signs and symptoms of hypoglycemia, how to treat hypoglycemia, and how to collect appropriate information for each episode of hypoglycemia.

Hypoglycemia may be identified by spontaneous reporting of symptoms from participants (whether confirmed or unconfirmed by simultaneous glucose values) or by BG samples collected during study visits.

All participants with T2DM and who develop diabetes during the study will be provided with glucometers.

Participants with T2DM will be provided a diary to record relevant information (for example, glucose values, symptoms).

All hypoglycemic episodes are to be recorded on a specific eCRF and should not be otherwise recorded as AEs unless the event meets severe criteria. If a hypoglycemic event meets severe criteria (see definition below), it should be recorded as serious on the AE eCRFs, and reported to the sponsor as an SAE. To avoid duplicate reporting, all consecutive BG values <70 mg/dL (<3.9 mmol/L) occurring within a 1-hour period may be considered to be a single hypoglycemic event (Weinberg et al. 2010; Danne et al. 2013).

Investigators should use the following definitions and criteria when diagnosing and categorizing an episode considered to be related to hypoglycemia (the BG values in this section refer to values determined by a laboratory or International Federation of Clinical Chemistry and Laboratory Medicine blood-equivalent glucose meters and strips) in accordance with the 2020 American Diabetes Association position statement on glycemic targets (ADA 2020):

Glucose Alert Value (Level 1):

- Documented symptomatic hypoglycemia is defined as any time a participant feels that he or she is experiencing symptoms and/or signs associated with hypoglycemia and has a BG level of <70 mg/dL (<3.9 mmol/L).
- Documented asymptomatic hypoglycemia is defined as any event not accompanied by typical symptoms of hypoglycemia, but with a measured BG <70 mg/dL (<3.9 mmol/L).
- Documented unspecified hypoglycemia is defined as any event with no information about symptoms of hypoglycemia available, but with a measured BG <70 mg/dL (<3.9 mmol/L).

Clinically Significant Hypoglycemia (Level 2):

- Documented symptomatic hypoglycemia is defined as any time a participant feels that he or she is experiencing symptoms and/or signs associated with hypoglycemia and has a BG level of <54 mg/dL (<3.0 mmol/L).

- Documented asymptomatic hypoglycemia is defined as any event not accompanied by typical symptoms of hypoglycemia but with a measured BG <54 mg/dL (<3.0 mmol/L).
- Documented unspecified hypoglycemia is defined as any event with no information about symptoms of hypoglycemia available but with a measured BG <54 mg/dL (<3.0 mmol/L).

Severe Hypoglycemia (Level 3):

- Severe hypoglycemia is defined as an episode with severe cognitive impairment requiring the assistance of another person to actively administer carbohydrate, glucagon, or other resuscitative actions. These episodes may be associated with sufficient neuroglycopenia to induce seizure or coma. Blood glucose measurements may not be available during such an event, but neurological recovery attributable to the restoration of BG to normal is considered sufficient evidence that the event was induced by a low BG concentration.

Nocturnal Hypoglycemia:

Nocturnal hypoglycemia is a hypoglycemia event (including severe hypoglycemia) that occurs at night, presumably during sleep.

10.5.1.2. Pancreatitis

Acute pancreatitis is defined as an AE of interest in all trials with tirzepatide, including this trial.

Acute pancreatitis is an acute inflammatory process of the pancreas that may also involve peripancreatic tissues and/or remote organ systems (Banks and Freeman 2006). The diagnosis of acute pancreatitis requires 2 of the following 3 features:

- abdominal pain, characteristic of acute pancreatitis (generally located in the epigastrium and radiates to the back in approximately half the cases) (Banks and Freeman 2006; Koizumi et al. 2006); the pain is often associated with nausea and vomiting
- serum amylase (total and/or pancreatic) and/or lipase ≥3X ULN, and
- characteristic findings of acute pancreatitis on CT scan or MRI.

If acute pancreatitis is suspected, appropriate laboratory tests (including levels of pancreatic amylase and lipase) should be obtained via the central laboratory (and locally, if needed).

Imaging studies, such as abdominal CT scan with or without contrast, MRI, or gallbladder ultrasound, should be performed. Abdominal ultrasound may be used as an alternative method only if CT and MRI cannot be performed. If laboratory values and/or abdominal imaging support the diagnosis of acute pancreatitis, the participant must discontinue therapy with tirzepatide but will continue in the study. A review of the participant's concomitant medications should be conducted to assess any potential causal relationship with pancreatitis.

Each AE of pancreatitis must be reported. If typical signs and/or symptoms of pancreatitis are present and confirmed by laboratory values (lipase or amylase [total and/or pancreatic]) and imaging studies, the event must be reported as an SAE. For a potential case that does not meet all of these criteria, it is up to the investigator to determine the seriousness of the case (AE or SAE) and the relatedness of the event to study drug(s).

Each participant will have measurements of p-amylase and lipase (assessed at the central laboratory) as shown on the SoA (Section 1.3) to assess the effects of the investigational doses of tirzepatide on pancreatic enzyme levels. Serial measurements of pancreatic enzymes have limited clinical value for predicting episodes of acute pancreatitis in asymptomatic participants (Nauck et al. 2017; Steinberg et al. 2017a, 2017b). Thus, further diagnostic follow-up of cases of asymptomatic pancreatic hyperenzymemia (lipase and/or pancreatic amylase ≥3X ULN) is not mandated but may be performed based on the investigator's clinical judgment and assessment of the participant's overall clinical condition. Only cases of pancreatic hyperenzymemia that undergo additional diagnostic follow-up and/or are accompanied by symptoms suggestive of pancreatitis will be submitted for adjudication.

All suspected cases of acute or chronic pancreatitis will be adjudicated by an independent clinical endpoint committee. In addition, AEs of severe or serious abdominal pain of unknown etiology will also be submitted to the adjudication committee to assess for possible pancreatitis or other pancreatic disease. Relevant data from participants with acute or chronic pancreatitis and those with severe or serious abdominal pain will be entered into a specifically designed eCRF page. The adjudication committee representative will enter the results of adjudication in a corresponding eCRF page.

10.5.1.3. Thyroid Malignancies and C-Cell Hyperplasia

Individuals with personal or family history of MTC and/or MEN-2 will be excluded from the study. Participants who are diagnosed with MTC and/or MEN-2 during the study will have study drug stopped and should continue follow-up with an endocrinologist.

The assessment of thyroid safety during the trial will include reporting of any case of thyroid malignancy (including MTC and papillary carcinoma) and measurements of calcitonin. This data will be captured in specific eCRFs. The purpose of calcitonin measurements is to assess the potential of tirzepatide to affect thyroid C-cell function, which may indicate development of C-cell hyperplasia and neoplasms.

10.5.1.4. Calcitonin Measurements

If an increased calcitonin value (see definitions below) is observed in a participant who has been administered a medication that is known to increase serum calcitonin, then this medication should be stopped, and calcitonin levels should be measured after an appropriate washout period.

For participants who require additional endocrine assessment because of increased calcitonin concentration as defined in this section, data from the follow-up assessment will be collected in the specific section of the eCRF.

Calcitonin Measurements in Participants with eGFR ≥60 mL/min/1.73 m^2

A significant increase in calcitonin for participants with eGFR ≥60 mL/min is defined below. If a participant's laboratory results meet these criteria, these clinically significant laboratory results should be recorded as an AE.

- calcitonin value ≥20 ng/L and <35 ng/L AND ≥50% increase from the screening value.
  - These participants will be asked to repeat the measurement within 1 month. If this repeat value is increasing (≥10% increase), study drug should be stopped, and the

participant encouraged to undergo additional endocrine assessment and longer-term follow-up by an endocrinologist to exclude any serious adverse effect on the thyroid.

- calcitonin value ≥35 ng/L AND ≥50% over the screening value.
    - In these participants, study drug should be stopped, and the participant recommended to immediately undergo additional endocrine assessments and longer-term follow-up by an endocrinologist.

Calcitonin Measurement in Participants with eGFR <60 mL/min/1.73 m^2

A significant increase in calcitonin for participants with eGFR <60 mL/min/1.73 m^2 is defined as a calcitonin value ≥35 ng/L AND ≥50% over the screening value. If a participant's labs meet these criteria, these clinically significant labs should be recorded as an AE.

In these participants, study drug should be discontinued (after first confirming the value), and the participant recommended to immediately undergo additional endocrine assessments and longer-term follow-up by an endocrinologist to exclude any serious adverse effect on the thyroid.

10.5.1.5. Major Adverse Cardiovascular Events

Deaths and nonfatal CV AEs will be adjudicated by a committee of physicians external to the sponsor with cardiology expertise. This committee will be blinded to treatment assignment. The nonfatal CV AEs to be adjudicated include:

- myocardial infarction
- hospitalization for unstable angina
- coronary interventions (such as coronary artery bypass graft or percutaneous coronary intervention), and
- cerebrovascular events, including cerebrovascular accident (stroke) and transient ischemic attack.

10.5.1.6. Supraventricular Arrhythmias and Cardiac Conduction Disorders

Participants who develop any event from these groups of disorders should undergo an ECG, which will be retained at the site as a source document. Additional diagnostic tests to determine exact diagnosis should be performed, as needed. The specific diagnosis will be recorded as an AE. Events that meet criteria for serious conditions as described in Section 10.3.2 must be reported as SAEs. If a clinically significant finding is identified by ECG (including, but not limited to, AF or changes from baseline in corrected QT interval), the investigator or qualified designee will determine if any change in study participant management is needed. This review of the ECG printed at the time of collection must be documented. Any new clinically relevant finding should be reported as an AE.

10.5.1.7. Hypersensitivity Events

All allergic or hypersensitivity reactions will be reported by the investigator as either AEs, or if any serious criterion is met, as SAEs.

In the event of suspected drug hypersensitivity reactions (immediate or nonimmediate) in subjects who experience moderate-to-severe reactions as assessed by the investigator, unscheduled blood samples will be collected as outlined in Appendix 7 (Section 10.7).

Additional data, such as type of reaction and treatment received, will be collected on any AEs or SAEs that the investigator deems related to study drug via the eCRF created for this purpose.

Study drug should be temporarily interrupted in any individual suspected of having a severe or serious allergic reaction to study drug. Study drug may be restarted when/if it is safe to do so, in the opinion of the investigator.

10.5.1.8. Injection Site Reactions

Injection site reactions will be collected on the eCRF separate from the hypersensitivity reaction eCRF. At the time of AE occurrence, samples will be collected for measurement of tirzepatide ADA and tirzepatide concentration.

10.5.1.9. Antidrug Antibodies

The occurrence of ADA formation will be assessed as outlined in Section 8.9.

10.5.1.10. Hepatobiliary Disorders

All events of treatment-emergent biliary colic, cholecystitis, or other suspected events related to gallbladder disease should be evaluated and additional diagnostic tests performed, as needed. In cases of elevated liver markers, hepatic monitoring should be initiated as outlined in Appendix 8 (Section 10.8).

10.5.1.11. Severe Gastrointestinal Adverse Events

Tirzepatide may cause severe GI AEs such as nausea, vomiting, and diarrhea. Information about severe GI AEs as well as antiemetic/antidiarrheal use will be collected in the eCRF/AE form. For detailed information concerning the management of GI AEs, please refer to Section 6.5.

10.5.1.12. Acute Renal Events

Renal safety will be assessed based on repeated renal function assessment as well as assessment of AEs suggestive of acute or worsening of chronic renal failure. Gastrointestinal AEs have been reported with tirzepatide, including nausea, diarrhea, and vomiting. This is consistent with other GLP-1 RAs (Aroda and Ratner 2011). The events may lead to dehydration, which could cause a deterioration in renal function, including acute renal failure.

Participants should be advised to notify investigators in case of severe nausea, frequent vomiting, or symptoms of dehydration.

10.6. Appendix 6: Genetics

Use/Analysis of DNA

- Genetic variation may impact a participant's response to study drug, susceptibility to, severity, and progression of disease. Variable response to study drug may be due to genetic determinants that impact drug absorption, distribution, metabolism, and excretion; mechanism of action of the drug; disease etiology; and/or molecular subtype of the disease being treated. Therefore, where local regulations and IRB/IEC allow, a blood sample will be collected for DNA analysis from consenting participants.
- DNA samples will be used for research related to tirzepatide or HF and related diseases. They may also be used to develop tests/assays including diagnostic tests related to tirzepatide or HF. Genetic research may consist of the analysis of one or more candidate genes, the analysis of genetic markers throughout the genome, or analysis of the entire genome (as appropriate).
- DNA sample analysis may be performed on pharmacogenetic variants thought to play a role in T2DM or CV disease to evaluate their association with observed clinical outcomes to tirzepatide in this study. In the event the observation of a study drug response, the samples may be genotyped, and analysis may be performed to evaluate a genetic association with response to tirzepatide. These investigations may be limited to a focused, candidate-gene study or, if appropriate, genome-wide association studies may be performed to identify regions of the genome associated with the variability observed in drug response. Samples may be used for investigations related to the disease, drug, or class of drugs under study in the context of this clinical program; however, samples may not be used for broad, exploratory, unspecified disease or population genetic analysis. Additional analyses may be conducted if it is hypothesized that this may help further understand the clinical data.
- The samples may be analyzed as part of a multi-study assessment of genetic factors involved in the response to tirzepatide or study drugs of this class to understand the study disease or related conditions.
- The results of genetic analyses may be reported in the CSR or in a separate study summary.
- The sponsor will store the DNA samples in a secure storage space with adequate measures to protect confidentiality.
- The samples will be retained while research on tirzepatide continues but no longer than 15 years or another period as per local requirements (see Section 10.1.10).

10.7. Appendix 7: Recommended Laboratory Testing for Hypersensitivity Events

Laboratory testing should be performed at the time of a systemic hypersensitivity event. The management of the AE may warrant lab testing beyond that described below and should be performed as clinically indicated. Laboratory assessments should be performed if the participant experiences generalized urticaria or if anaphylaxis is suspected.

- Collect the sample after the participant has been stabilized and within 1 to 2 hours of the event; however, samples may be obtained as late as 12 hours after the event as analytes can remain altered for an extended period of time. Record the time at which the sample was collected.
- Obtain a follow-up sample at the next regularly scheduled visit or after approximately 4 weeks, whichever is later.

Clinical Laboratory Tests for Hypersensitivity Events

**Hypersensitivity Tests**	**Notes**<br>Selected test may be obtained in the event of anaphylaxis or systemic allergic/hypersensitivity reactions.
Tirzepatide antidrug antibodies (immunogenicity/ADA)	Assayed by Lilly-designated laboratory.<br>Results will not be provided to the investigative sites.
Tirzepatide concentrations (PK)	Assayed by Lilly-designated laboratory.<br>Results will not be provided to the investigative sites.
Tryptase	Assayed by Lilly-designated laboratory.<br>Results will not be provided to the investigative sites.<br>Urine N-methylhistamine testing is performed in addition to tryptase testing. Collect the first void urine sample following the event. Collect a follow-up urine sample after approximately 4 weeks.<br>**Note:** If a tryptase sample is obtained more than 2 hours after the event (that is, within 2-12 hours), or is not obtained because more than 12 hours have lapsed since the event, collect a urine sample for N-methylhistamine testing.
N-methylhistamine	Will be performed if validated assay is available.<br>Assayed by Lilly-designated laboratory.<br>Results will not be provided to the investigative sites.
Drug-specific IgE	Will be performed if a validated assay is available.<br>Assayed by Lilly-designated laboratory.<br>Results will not be provided to the investigative sites.
Basophil activation test	Will be performed if a validated assay is available.<br>Assayed by Lilly-designated laboratory.<br>Results will not be provided to the investigative sites.<br>**NOTE:** The basophil activation test is an in vitro, cell-based assay that only requires a serum sample. It is a surrogate assay for drug specific-IgE but is not specific for IgE.
Complement (C3, C3a and C5a)	Assayed by Lilly-designated laboratory.<br>Results will not be provided to the investigative sites.
Cytokine panel: IL-6, IL-1β, IL-10	Assayed by Lilly-designated laboratory.<br>Results will not be provided to the investigative sites.

Abbreviations: ADA = antidrug antibody; IgE = immunoglobulin E; IL = interleukin; PK = pharmacokinetic.

10.8. Appendix 8: Liver Safety: Suggested Actions and Follow-Up Assessments

Hepatic Evaluation Testing

See Section 8.2.5.1 for guidance on appropriate test selection.

The Lilly-designated central laboratory must complete the analysis of all selected testing except for microbiology testing.

Local testing may be performed in addition to central testing when necessary for immediate participant management.

Results will be reported if a validated test or calculation is available.

**Hematology**	**Clinical Chemistry**
Hemoglobin	Total bilirubin
Hematocrit	Direct bilirubin
Erythrocytes (red blood cells [RBCs])	Alkaline phosphatase (ALP)
Leukocytes (white blood cells [WBCs])	Alanine aminotransferase (ALT)
Differential:	Aspartate aminotransferase (AST)
Neutrophils, segmented	Gamma-glutamyl transferase (GGT)
Lymphocytes	Creatine kinase (CK)
Monocytes	**Other Chemistry**
Basophils	Acetaminophen
Eosinophils	Acetaminophen protein adducts
Platelets	Alkaline phosphatase isoenzymes
Cell morphology (RBC and WBC)	Ceruloplasmin
**Coagulation**	Copper
	Ethyl alcohol (EtOH)
Prothrombin time, INR (PT-INR)	Haptoglobin
**Serology**	Immunoglobulin IgA (quantitative)
Hepatitis A virus (HAV) testing:	Immunoglobulin IgG (quantitative)
HAV total antibody	Immunoglobulin IgM (quantitative)
HAV IgM antibody	Phosphatidylethanol (PEth)
Hepatis B virus (HBV) testing:	**Urine Chemistry**
Hepatitis B surface antigen (HBsAg)	Drug screen
Hepatitis B surface antibody (anti-HBs)	Ethyl glucuronide (EtG)
Hepatitis B core total antibody (anti-HBc)	**Other Serology**
Hepatitis B core IgM antibody	Antinuclear antibody (ANA)
Hepatitis B core IgG antibody	Anti-smooth muscle antibody (ASMA)[a]

HBV DNA[b]	Anti-actin antibody[c]
Hepatis C virus (HCV) testing:	Epstein-Barr virus (EBV) testing:
HCV antibody	EBV antibody
HCV RNA[b]	EBV DNA[b]
Hepatitis D virus (HDV) testing:	Cytomegalovirus (CMV) testing:
HDV antibody	CMV antibody
Hepatitis E virus (HEV) testing:	CMV DNA[b]
HEV IgG antibody	Herpes simplex virus (HSV) testing:
HEV IgM antibody	HSV (Type 1 and 2) antibody
HEV RNA[b]	HSV (Type 1 and 2) DNA[b]
**Microbiology**[d]	Liver kidney microsomal type 1 (LKM-1) antibody
Culture:	
Blood	
Urine	

Abbreviations: Ig = immunoglobulin; INR = international normalized ratio; PT = prothrombin time.

[a] Not required if anti-actin antibody is tested.

[b] Reflex/confirmation dependent on regulatory requirements, testing availability, or both.

[c] Not required if anti-smooth muscle antibody (ASMA) is tested.

[d] Assayed ONLY by investigator-designated local laboratory; no central testing available.

10.9. Appendix 9: Medical Device Adverse Events (AEs), Adverse Device Effects (ADEs), Serious Adverse Events (SAEs) and Device Deficiencies: Definition and Procedures for Recording, Evaluating, Follow-Up, and Reporting

Refer to Section 10.3 for definitions and procedures for recording, evaluating, follow-up, and reporting of all events.

10.10. Appendix 10: Six-Minute Walk Test Screening Procedures

10.10.1. Screening Procedures and Flow Diagram

The flow diagram below details the participant flow and eligibility with the 6MWT.

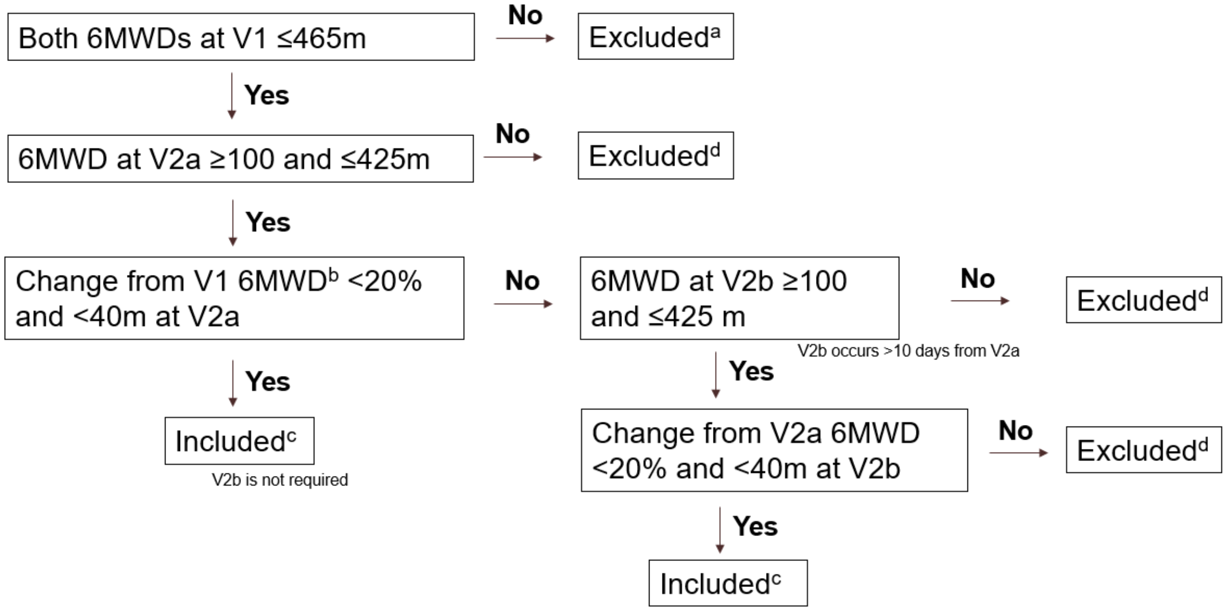

Abbreviations: 6MWD = 6-minute walk test distance; 6MWT = 6-minute walk test; V1 = Visit 1; V2a = Visit 2a; V2b = Visit 2b.

a Rescreening is not allowed

b Use the higher value of the two Visit 1 6MWD as a reference for Visit 2a.

c Continue with other Visit 2 assessments according to the SoA.

d Participants excluded on 6MWT may be re-screened after a minimum of 2 weeks.

10.11. Appendix 11: New York Heart Association Classification of Heart Failure

Class	Symptomatology
**I**	No symptoms. Ordinary physical activity such as walking and climbing stairs does not cause fatigue or dyspnea.
**II**	Symptoms with ordinary physical activity. Walking or climbing stairs rapidly, walking uphill, walking or stair climbing after meals, in cold weather, in wind or when under emotional stress causes undue fatigue or dyspnea.
**III**	Symptoms with less than ordinary physical activity. Walking 1-2 blocks on the level and climbing more than one flight of stairs in normal conditions causes undue fatigue or dyspnea.
**IV**	Symptoms at rest. Inability to carry on any physical activity without fatigue or dyspnea.

10.12. Appendix 12: Abbreviations

Term	Definition
**6MWD**	6-minute walk test distance
**6MWT**	6-minute walk test
**ADA**	antidrug antibody
**AE**	adverse event
**AESI**	adverse events of special interest
**AF**	atrial fibrillation
**ALP**	alkaline phosphatase
**ALT**	alanine aminotransferase
**AST**	aspartate aminotransferase
**blinding/masking**	A single-blind study is one in which the investigator and/or his staff are aware of the treatment but the participant is not, or vice versa, or when the sponsor is aware of the treatment but the investigator and/his staff and the participant are not.<br>A double-blind study is one in which neither the participant nor any of the investigator or sponsor staff who are involved in the treatment or clinical evaluation of the subjects are aware of the treatment received.
**BG**	blood glucose
**BMI**	body mass index
**CEC**	clinical endpoint committee
**CFR**	Code of Federal Regulations
**CIOMS**	Council for International Organizations of Medical Sciences
**complaint**	A complaint is any written, electronic, or oral communication that alleges deficiencies related to the identity, quality, purity, durability, reliability, safety or effectiveness, or performance of a drug or drug delivery system.
**compliance**	Adherence to all study-related, good clinical practice (GCP), and applicable regulatory requirements.
**CONSORT**	Consolidated Standards of Reporting Trials
**CRF**	case report form
**CRP**	clinical research physician: Individual responsible for the medical conduct of the study. Responsibilities of the CRP may be performed by a physician, clinical research scientist, global safety physician or other medical officer.

Term	Definition
**CSR**	clinical study report
**CT**	computed tomography
**CV**	cardiovascular
**D Bil**	direct bilirubin
**DMC**	data monitoring committee
**Device Deficiencies**	Equivalent to product complaint
**ECG**	electrocardiogram
**eCOA**	electronic clinical outcome assessment
**eCRF**	electronic case report form
**EDC**	electronic data capture
**eGFR**	estimated glomerular filtration rate
**enroll**	The act of assigning a participant to a treatment. Participants who are enrolled in the study are those who have been assigned to a treatment.
**enter**	Participants entered into a study are those who sign the informed consent form directly or through their legally acceptable representatives.
**ERB**	ethical review board
**ET**	early termination
**EV**	extended visit
**GCP**	good clinical practice
**GGT**	gamma-glutamyl transferase
**GI**	gastrointestinal
**GIP**	glucose-dependent insulinotropic polypeptide
**GLP-1**	glucagon-like peptide-1
**HbA1c**	hemoglobin A1c
**HF**	heart failure
**HFpEF**	heart failure with preserved ejection fraction
**HIPAA**	Health Insurance Portability and Accountability Act

Term	Definition
**IB**	Investigator's Brochure
**ICF**	informed consent form
**ICH**	International Council for Harmonisation
**IEC**	independent ethics committee
**informed consent**	A process by which a participant voluntarily confirms his or her willingness to participate in a particular study, after having been informed of all aspects of the study that are relevant to the participant's decision to participate. Informed consent is documented by means of a written, signed and dated informed consent form.
**INR**	international normalized ratio
**interim analysis**	An interim analysis is an analysis of clinical study data, separated into treatment groups, that is conducted before the final reporting database is created/locked.
**IRB**	institutional review board
**ISO**	International Organization for Standardization
**ITT**	intention to treat: The principle that asserts that the effect of a treatment policy can be best assessed by evaluating on the basis of the intention to treat a participant (that is, the planned treatment regimen) rather than the actual treatment given. It has the consequence that participant allocated to a treatment group should be followed up, assessed, and analyzed as members of that group irrespective of their compliance to the planned course of treatment.
**IV**	intravenous
**IWRS**	interactive web-response system
**KCCQ**	Kansas City Cardiomyopathy Questionnaire
**KCCQ-CSS**	Kansas City Cardiomyopathy Questionnaire – Clinical Summary Scale
**LA**	left atrial
**LAV**	left atrial volume
**LVDEP**	left ventricular end-diastolic pressure
**LVEF**	left ventricular ejection fraction
**MedDRA**	Medical Dictionary for Regulatory Activities
**MEN-2**	multiple endocrine neoplasia type 2
**MMRM**	mixed model repeated measures
**MRI**	magnetic resonance imaging

Term	Definition
**MTC**	medullary thyroid cancer
**MTD**	maximum tolerated dose
**NT-proBNP**	N-terminal pro b-type natriuretic peptide
**NYHA**	New York Heart Association
**participant**	Equivalent to CDISC term "subject": an individual who participates in a clinical trial, either as recipient of an investigational medicinal product or as a control
**PC**	product complaint
**PCWP**	pulmonary capillary wedge pressure
**PK**	pharmacokinetics
**PT**	prothrombin time
**QTc**	corrected QT interval
**QW**	weekly
**RA**	receptor agonist
**SADE**	serious adverse device effect
**SAE**	serious adverse event
**SAP**	statistical analysis plan
**SBP**	systolic blood pressure
**SC**	subcutaneous
**screen**	The act of determining if an individual meets minimum requirements to become part of a pool of potential candidates for participation in a clinical study.
**SoA**	schedule of activities
**study intervention**	for this study, study intervention may be interpreted/synonymous with study drug
**SUSAR**	suspected unexpected serious adverse reaction
**T1DM**	type 1 diabetes mellitus
**T2DM**	type 2 diabetes mellitus
**TBL**	total bilirubin

Term	Definition
**TEAE**	Treatment-emergent adverse event: An untoward medical occurrence that emerges during a defined treatment period, having been absent pretreatment, or worsens relative to the pretreatment state, and does not necessarily have to have a causal relationship with this treatment.
**UADE**	unanticipated adverse device effect
**ULN**	upper limit of normal
**WOCBP**	women of childbearing potential

10.13. Appendix 13: Protocol Amendment History

The Protocol Amendment Summary of Changes Table for the current amendment is located directly before the Table of Contents (TOC).

Amendment b: 21 January 2022

Overall Rationale for the Amendment:

The purpose of this protocol amendment is to incorporate feedback received from the FDA on exclusion criterion and concomitant therapy.

Section # and Name	Description of Change	Brief Rationale
Section 5.1 Inclusion Criteria	Criterion #7: removed "and/or" wording; added "or" between ARNI and SGLT2is	For clarification
Section 5.2 Exclusion Criteria	Criterion #13: for NYHA Class, added word "or" in sentence to ensure participants with NYHA Class I are excluded from the study<br>Criterion #20: for AF, changed "and" to "or" between "Visit 1" and "Visit 2"	For clarification
Section 6.5 Concomitant Therapy	Removed sentence on use of SGLT2i for treatment of T2DM	Per FDA feedback
Section 7.1.1 Permanent Discontinuation from Study Drug	A sentence inadvertently added and then was removed	For clarification

Amendment a: 15 December 2021

Overall Rationale for the Amendment:

The purpose of this protocol amendment is to clarify dosing information to allow for more participant retention and clarity on the dosing regimen, add additional unscheduled visits, and make inclusion/exclusion criteria updates.

Section # and Name	Description of Change	Brief Rationale
Section 1.1 Synopsis	• In Intervention Groups and Duration, changed 15 mg to QW<br>• Added wording to Study Period 1 to specify timing	• To Clarify dose<br>• For Clarification in timing
Section 1.2 Schema	Adjusted wording in Note for screening procedures timing	To Clarify screening period timing
Section 1.3 Schedule of Activities (SoA)	• Added 3 columns to Unscheduled Visits (UV): UV, Dosing UV, and Phone follow up Dosing UV, added Xs for specific tests<br>• Footnote “a” added in regard to Unscheduled Visits; subsequent footnotes adjusted<br>• For Telephone Visit, X added to Phone Follow-Up Dosing UV	• To specify UV<br>• To explain UV; for accuracy<br>• For Accuracy
Section 3 Objectives and Endpoints	• Added Other Secondary title & moved Incidence HF events and CV Death from Exploratory<br>• Added timing to first occurrence and recurrent events for HF events and CV death<br>• The Endpoint wording for the exploratory Objective Atrial fibrillation is updated	• Change in Endpoints<br>• To specify timings<br>• Correction
Section 4.1 Overall Design	• For Study Period 1, timing period adjusted<br>• For Screening, sentence added to clarify time period for V1<br>• For Treatment, dose escalation described; paragraphs added to specify that participant be	• Correction<br>• Clarification for timing for duration of V1<br>• New information added to clarify dose escalation,

	maintained on study drug; some sentences regarding dose and discontinuation removed	maintenance on study drug; To reduce redundancy
Section 5.1 Inclusion Criteria	• For Inclusion Criterion #7 added additional HF medications	• Clarification for Concomitant Medication use
Section 5.2 Exclusion Criteria	• For Exclusion Criterion #13, NYHA Class information added<br>• For Exclusion Criterion #15 changed wording to include MRI or other local modalities<br>• For Exclusion Criterion #21 added sentence on participants with Chagas disease<br>• For Exclusion Criterion #25 removed sentence regarding fundoscopic examination and clarified definition of nonproliferative diabetic retinopathy	• Additional information<br>• For Clarification<br>• For Clarification<br>• Correction and Clarification
Section 5.4 Screen Failures	Added upper limit of screening period to clarify screen failure	For Clarification
Section 6.4 Study Intervention Compliance	Wording added to clarify participant compliance	For Clarification
Section 6.5 Concomitant Therapy	• Added information on ARNI, SGLT2i, and GLP-1/GIPR use in the study<br>• Changed Hyperglycemia Rescue at Week 26 to Week 24<br>• Wording added in Standard of Care for Heart Failure<br>• Wording changed to Clarify dosing modification for GI Symptoms	• Clarification of Concomitant Medication Use<br>• Correction<br>• For Clarification<br>• For Clarification
Section 6.6 Dose Modification	Information changed to clarify dose modification	For Clarification

Section 6.7 Intervention after the End of the Study	Wording added in regard to unblinding information	For Clarification
Section 7.1.1 Permanent Discontinuation from Study Drug	• Wording added to clarify permanent discontinuation of study drug<br>• Wording changed to specific criteria when participant has Pancreatic Cancer	• For Clarification<br>• For Clarification
Section 7.1.2 Temporary Interruption	Wording changed to clarify that study interruption, is not study discontinuation; Moved to Section 6.6.1	For Clarification
Section 7.1.3 Restarting Study Drug after Interruption	Added wording on Maintenance dose levels; Moved to Section 6.6.2	For Clarification
Section 7.2 Participant Discontinuation/Withdrawal from the Study	Wording changed to clarify participant retention in the study	For Clarification
Section 7.2.1 Inadvertently Enrolled Participants	Wording Changed to clarify medical appropriateness of treatment	For Clarification
Section 8 Study Assessments and Procedures	Information added regarding remote visits	For Clarification
Section 8.1.1.3 Six-Minute Walk Test	Clarified wording on Borg Scale	For Clarification
Section 8.2.3 Electrocardiograms	Clarified on use of ECG machine type	For Clarification
Section 8.2.4 Clinical Safety Laboratory Assessments	Urine pregnancy wording added	For Clarification
Section 10.1.1 Regulatory and Ethical Considerations	Added bullet point, International Organization for Standardization (ISO) 14155 and wording on substantiality of amendments	To align with current Harmonized Protocol Template v10

Section 10.1.11 Investigator Information	Added Investigator Information Section	To align with current Harmonized Protocol Template v10
Section 10.2 Appendix 2: Clinical Laboratory Tests	Removed Lactate dehydrogenase	Error Correction
Section 10.5.1.5 Major Adverse Cardiovascular Events	Removed hospitalization for HF	This is an efficacy point
Section 10.10 Appendix 10: Six Minute Walk Test Screening Procedures	Flow Diagram corrected for 6MWD	Error Correction

11. References

[ADA] American Diabetes Association. Glycemic targets: standards of medical care in diabetes – 2020. *Diabetes Care*. 2020;43(suppl 1):S66-S76. https://doi.org/10.2337/dc20-S006

Aminian A, Zajichek A, Arterburn DE, et al. Association of metabolic surgery with major adverse cardiovascular outcomes in patients with type 2 diabetes and obesity. *JAMA*. 2019;322(13):1271-1282. https://doi.org/10.1001/jama.2019.14231

Aroda VR, Ratner R. The safety and tolerability of GLP-1 receptor agonists in the treatment of type 2 diabetes: a review. *Diabetes Metab Res Rev*. 2011;27(6):528-542. https://doi.org/10.1002/dmrr.1202

Banks PA, Freeman ML.; Practice Parameters Committee of the American College of Gastroenterology. Practice guidelines in acute pancreatitis. *Am J Gastroenterol*. 2006;101(10):2379-2400. https://doi.org/10.1111/j.1572-0241.2006.00856.x

Chatur S, Vaduganathan M, Claggett BL, et al. Outpatient worsening among patients with mildly reduced and preserved ejection fraction heart failure in the DELIVER Trial. *Circulation*. 2023;148(22):1735-1745. https://doi.org/10.1161/CIRCULATIONAHA.123.066506

Danne T, Philotheou A, Goldman D, et al. A randomized trial comparing the rate of hypoglycemia – assessed using continuous glucose monitoring – in 125 preschool children with type 1 diabetes treated with insulin glargine or NPH insulin (the PRESCHOOL study). *Pediatr Diabetes*. 2013;14(8):593-601. https://doi.org/10.1111/pedi.12051

Davies MJ, Bergenstal R, Bode B, et al. Efficacy of liraglutide for weight loss among patients with type 2 diabetes: The SCALE Diabetes Randomized Clinical Trial. *JAMA*. 2015;314(7):687-699. https://doi.org/10.1001/jama.2015.9676 Erratum in: JAMA. 2016 Jan 5;315(1):90. PMID: 26284720

Davies MJ, D'Alessio DA, Fradkin J, et al. Management of hyperglycemia in type 2 diabetes, 2018. A consensus report by the American Diabetes Association (ADA) and the European Association for the Study of Diabetes (EASD). *Diabetes Care*. 2018;41(12):2669-2701. https://doi.org/10.2337/dci18-0033

[EMA] European Medicines Agency. Guideline on clinical evaluation of medicinal products used in weight management. EMA/CHMP/311805/2014. Published June 23, 2016. Accessed December 23, 2020. https://www.ema.europa.eu/en/documents/scientific-guideline/guideline-clinical-evaluation-medicinal-products-used-weight-management-revision-1_en.pdf

EuroQol Research Foundation. EQ-5D-5L User Guide, Version 3.0. Updated September 2019. Accessed March 13, 2020. https://euroqol.org/publications/user-guides

[FDA] United States Food and Drug Administration. Guidance for industry: developing products for weight management. Published February 2007. Accessed December 23, 2020. https://www.fda.gov/media/71252/download

Ferreira JP, Liu J, Claggett BL, et al. Outpatient diuretic intensification as endpoint in heart failure with preserved ejection fraction trials: an analysis from TOPCAT. *Eur J Heart Fail*. 2022;24(2):378-384. https://doi.org/10.1002/ejhf.2376Frias JP, Nauck MA, Van J, et al. Efficacy and safety of LY3298176, a novel dual GIP and GLP-1 receptor agonist, in patients

with type 2 diabetes: a randomised, placebo-controlled and active comparator-controlled phase 2 trial. *Lancet*. 2018;392(10160):2180-2193. https://doi.org/10.1016/s0140-6736(18)32260-8

Green CP, Porter CB, Bresnahan DR, Spertus JA. Development and evaluation of the Kansas City Cardiomyopathy Questionnaire: a new health status measure for heart failure. *J Am Coll Cardiol*. 2000;35(5):1245-1255. https://doi.org/10.1016/S0735-1097(00)00531-3

Herring LY, Stevinson C, Davies MJ, et al. Changes in physical activity behaviour and physical function after bariatric surgery: a systematic review and meta-analysis. *Obes Rev*. 2016;17(3):250-261. https://doi.org/10.1111/obr.12361

Jamaly S, Carlsson L, Peltonen M, et al. Surgical obesity treatment and the risk of heart failure. *Eur Heart J*. 2019;40(26):2131-2138. https://doi.org/10.1093/eurheartj/ehz295

Jastreboff AM, Aronne LJ, Ahmad NN, et al. Tirzepatide once weekly for the treatment of obesity. *N Engl J Med*. 2022;387(3):205-216. https://doi.org/10.1056/NEJMoa2206038

Joseph SM, Novak E, Arnold SV, et al. Comparable performance of the Kansas City Cardiomyopathy Questionnaire in patients with heart failure with preserved and reduced ejection fraction. *Circ Heart Fail*. 2013;6(6):1139-1146. https://doi.org/10.1161/CIRCHEARTFAILURE.113.000359

Kosiborod MN, Bhatt AS, Claggett BL, et al. Effect of dapagliflozin on health status in patients with preserved or mildly reduced ejection fraction. *J Am Coll Cardiol*. 2023;81(5):460-473. https://doi.org/10.1016/j.jacc.2022.11.006

Kitzman DW, Brubaker P, Morgan T, et al. Effect of caloric restriction or aerobic exercise training on peak oxygen consumption and quality of life in obese older patients with heart failure with preserved ejection fraction: a randomized clinical trial. *JAMA*. 2016;315(1):36-46. https://doi.org/10.1001/jama.2015.17346

Kitzman DW, Shah SJ. The HFpEF obesity phenotype: the elephant in the room. *J Am Coll Cardiol*. 2016;68(2):200-203. https://doi.org/10.1016/j.jacc.2016.05.019

Koizumi M, Takada T, Kawarada Y, et al. JPN Guidelines for the management of acute pancreatitis: diagnostic criteria for acute pancreatitis. *J Hepatobiliary Pancreat Surg*. 2006;13(1):25-32. https://doi.org/10.1007/s00534-005-1048-2

Lingvay I, Desouza CV, Lalic KS, et al. A 26-week randomized controlled trial of semaglutide once daily versus liraglutide and placebo in patients with type 2 diabetes suboptimally controlled on diet and exercise with or without metformin. *Diabetes Care*. 2018;41(9):1926-1937. https://doi.org/10.2337/dc17-2381

Madelaire C, Gustafsson F, Stevenson LW. One-year mortality after intensification of outpatient diuretic therapy. *J Am Heart Assoc*. 2020;9(14):e016010. https://doi.org/10.1161/jaha.119.016010

Mikhalkova D, Holman SR, Jiang H, et al. Bariatric surgery-induced cardiac and lipidomic changes in obesity-related heart failure with preserved ejection fraction. *Obesity (Silver Spring)*. 2018;26(2):284-290. https://doi.org/10.1002/oby.22038

Miller WL, Borlaug BA. Impact of obesity on volume status in patients with ambulatory chronic heart failure. *J Card Fail*. 2020;26(2):112-117. https://doi.org/10.1016/j.cardfail.2019.09.010

Nauck MA, Frossard JL, Barkin JS, et al. Assessment of pancreas safety in the development program of once-weekly GLP-1 receptor agonist dulaglutide. *Diabetes Care*. 2017;40(5):647-654. https://doi.org/10.2337/dc16-0984

Packer M. Epicardial adipose tissue may mediate deleterious effects of obesity and inflammation on the myocardium. *J Am Coll Cardiol*. 2018;71(20):2360-2372. https://doi.org/10.1016/j.jacc.2018.03.509

Pi-Sunyer X, Astrup A, Fujioka K, et al; SCALE Obesity and Prediabetes NN8022-1839 Study Group. A randomized, controlled trial of 3.0 mg of liraglutide in weight management. *N Engl J Med*. 2015;373(1):11-22. https://doi.org/10.1056/NEJMoa1411892

Rayner JJ, Peterzan MA, Watson WD, et al. Myocardial energetics in obesity: enhanced ATP delivery through creatine kinase with blunted stress response. *Circulation*. 2020;141(14):1152-1163. https://doi.org/10.1161/CIRCULATIONAHA.119.042770

Reddy YN, Rikhi A, Obokata M, et al. Quality of life in heart failure with preserved ejection fraction: importance of obesity, functional capacity, and physical inactivity. *Eur J Heart Fail*. 2020;22(6):1009-1018. https://doi.org/10.1002/ejhf.1788

Regev A, Palmer M, Avigan MI, et al. Consensus: guidelines: best practices for detection, assessment and management of suspected acute drug-induced liver injury during clinical trials in patients with nonalcoholic steatohepatitis. *Aliment Pharmacol Ther*. 2019;49(6):702-713.

Rubin DB. *Multiple Imputation for Nonresponse in Surveys*. John Wiley & Sons, Inc.; 1987.

Shimada YJ, Tsugawa Y, Brown DFM, Hasegawa K. Bariatric surgery and emergency department visits and hospitalizations for heart failure exacerbation: population-based, self-controlled series. *J Am Coll Cardiol*. 2016;67(8):895-903. https://doi.org/10.1016/j.jacc.2015.12.016

Shoemaker MJ, Curtis AB, Vangsnes E, Dickinson MG. Clinically meaningful change estimates for the six-minute walk test and daily activity in individuals with chronic heart failure. *Cardiopulm Phys Ther J*. 2013;24(3):21-29.

Solomon SD, Dobson J, Pocock S, et al.; Candesartan in Heart Failure: Assessment of Reduction in Mortality and Morbidity (CHARM) Investigators. *Circulation*. 2007;116(13):1482-1487. https://doi.org/10.1161/CIRCULATIONAHA.107.696906

Spertus JA, Jones PG, Sandhu AT, Arnold SV. Interpreting the Kansas City Cardiomyopathy Questionnaire in clinical trials and clinical care: JACC state-of-the-art review. *J Am Coll Cardiol*. 2020;76(20):2379-2390. https://doi.org/10.1016/j.jacc.2020.09.542

Steinberg WM, Buse JB, Ghorbani MLM, et al.; LEADER Steering Committee; LEADER Trial Investigators. Amylase, lipase, and acute pancreatitis in people with type 2 diabetes treated with liraglutide: results from the LEADER randomized trial. *Diabetes Care*. 2017a;40(7):966-972. https://doi.org/10.2337/dc16-2747

Steinberg WM, Rosenstock J, Wadden TA, et al. Impact of liraglutide on amylase, lipase, and acute pancreatitis in participants with overweight/obesity and normoglycemia, prediabetes, or type 2 diabetes: secondary analyses of pooled data from the SCALE clinical development program. *Diabetes Care*. 2017b;40(7):839-848. https://doi.org/10.2337/dc16-2684

Sundström J, Bruze G, Ottosson J, et al. Weight loss and heart failure: a nationwide study of gastric bypass surgery versus intensive lifestyle treatment. *Circulation*. 2017;135(17):1577-1585. https://doi.org/10.1161/CIRCULATIONAHA.116.025629

Täger T, Hanholz W, Cebola R, et al. Minimal important difference for 6-minute walk test distances among patients with chronic heart failure. *Int J Cardiol*. 2014;176(1):94-98. https://doi.org/10.1016/j.ijcard.2014.06.035

van der Meer P, Gaggin HK, Dec GW. ACC/AHA versus ESC guidelines on heart failure: JACC guideline comparison. *J Am Coll Cardiol*. 2019;73(21):2756-2768. https://doi.org/10.1016/j.jacc.2019.03.478

Weinberg ME, Bacchetti P, Rushakoff RJ. Frequently repeated glucose measurements overestimate the incidence of inpatient hypoglycemia and severe hyperglycemia. *J Diabetes Sci Technol.* 2010;4(3):577-582. https://doi.org/10.1177/193229681000400311

Wilson JM, Nikooienejad A, Robins DA, et al. The dual glucose-dependent insulinotropic peptide and glucagon-like peptide-1 receptor agonist, tirzepatide, improves lipoprotein biomarkers associated with insulin resistance and cardiovascular risk in patients with type 2 diabetes [published online August 16, 2020]. *Diab Obes and Metab*. https://doi.org/10.1111/dom.14174

Signature Page for VV-CLIN-120976 v1.0

Approval	PPD [illegible]<br>[illegible] 23:44 GMT+0000

Approval	PPD [illegible]<br>[illegible]24 19:09:49 GMT+0000

Signature Page for VV-CLIN-120976 v1.0

1. Statistical Analysis Plan I8F-MC-GPID: A Randomized, Double-Blind, Placebo-Controlled, Phase 3 Study Comparing the Efficacy and Safety of Tirzepatide versus Placebo in Patients with Heart Failure with Preserved Ejection Fraction and Obesity (SUMMIT)

Confidential Information

The information contained in this document is confidential and the information contained within it may not be reproduced or otherwise disseminated without the approval of Eli Lilly and Company or its subsidiaries.

Note to Regulatory Authorities: this document may contain protected personal data and/or commercially confidential information exempt from public disclosure. Eli Lilly and Company requests consultation regarding release/redaction prior to any public release. In the United States, this document is subject to Freedom of Information Act (FOIA) Exemption 4 and may not be reproduced or otherwise disseminated without the written approval of Eli Lilly and Company or its subsidiaries.

LY3298176 for Obesity-related HFpEF

A randomized, outpatient, multi-center, international, placebo-controlled, double-blinded parallel arm Phase 3 study to evaluate the efficacy and safety of QW tirzepatide, maximum tolerated dose (MTD) up to 15 mg, compared to placebo, in participants with heart failure with preserved ejection fraction and obesity.

Eli Lilly and Company
Indianapolis, Indiana USA 46285
Protocol I8F-MC-GPID
Phase 3

Approval Date: 22-Nov-2021 GMT

2. Table of Contents

Section **Page**

1. Statistical Analysis Plan I8F-MC-GPID: A Randomized, Double-Blind, Placebo-Controlled, Phase 3 Study Comparing the Efficacy and Safety of Tirzepatide versus Placebo in Patients with Heart Failure with Preserved Ejection Fraction and Obesity (SUMMIT) 1
2. Table of Contents 2
3. Revision History 8
4. Study Objectives 9
4.1. Primary Objective 9
4.2. Key Secondary Objectives 9
5. Study Design 10
6. A Priori Statistical Methods 11
6.1. Populations for Analyses 11
6.2. General Considerations 11
6.3. Adjustments for Covariates 12
6.4. Handling of Dropouts or Missing Data 12
6.5. Multicenter Studies 13
6.6. Multiple Comparisons/Multiplicity 13
6.7. Patient Disposition 13
6.8. Historical Illnesses and Preexisting Conditions 13
6.9. Patient Characteristics 13
6.10. Concomitant Therapy 13
6.11. Treatment Exposure and Compliance 14
6.11.1. Study and Study Treatment Exposure 14
6.11.2. Compliance to Study Treatment 14
6.12. Important Protocol Deviations 14
6.13. Efficacy Analyses 14
6.13.1. Primary Endpoints/Estimands Analysis 15
6.13.1.1. The Hierarchical Composite Endpoint 15
6.13.1.1.1. Estimand for the Endpoint 15
6.13.1.1.2. Main Analytical Approach 16
6.13.1.2. Change from Baseline in 6MWD 17
6.13.1.2.1. Estimand for the Endpoint 17
6.13.1.2.2. Main Analytical Approach 17
6.13.1.3. Methods for Missing Data Imputation 17
6.13.1.4. Sensitivity Analyses for Primary Endpoint 17

6.13.1.5. Supplementary Analyses 18
6.13.2. Key Secondary Endpoints/Estimands 18
6.13.2.1. Change from Baseline in KCCQ-CSS at Week 52 and 6MWD at Week 24 18
6.13.2.1.1. Estimand for the Endpoints 18
6.13.2.1.2. Main Analytical Approach 18
6.13.2.2. Percent Change from Baseline in Body Weight Loss at Week 52 19
6.13.2.2.1. Estimand for the Endpoint 19
6.13.2.2.2. Main Analytical Approach 19
6.13.2.3. Proportion of Participants with NYHA Class Change at Week 52 19
6.13.2.3.1. Estimand for the Endpoint 19
6.13.2.3.2. Main Analytical Approach 19
6.13.2.4. Sensitivity Analyses for Key Secondary Endpoints 20
6.13.2.5. Supplementary Analyses Related to Key Secondary Endpoints 20
6.13.3. Type I Error Rate Control Strategy for Primary and Key Secondary Efficacy Analyses 20
6.13.4. Exploratory Endpoints 21
6.14. Safety Analyses 22
6.14.1. Analysis of Adverse Events 22
6.14.1.1. Treatment Emergent Adverse Events 22
6.14.1.2. Common Adverse Events 23
6.14.1.3. Deaths 23
6.14.1.4. Other Serious Adverse Events 23
6.14.1.5. Other Significant Adverse Events 24
6.14.2. Patient Narratives 24
6.14.3. Special Safety Topics 24
6.14.3.1. Exocrine Pancreas Safety 24
6.14.3.1.1. Pancreatic Enzyme 24
6.14.3.1.2. Pancreatitis Events 24
6.14.3.2. Gastrointestinal Adverse Events 25
6.14.3.2.1. Nausea, Vomiting, and Diarrhea 25
6.14.3.2.2. Severe Gastrointestinal Events 25
6.14.3.3. Hepatobiliary Disorders 25
6.14.3.3.1. Hepatobiliary Events 25
6.14.3.3.2. Liver Enzymes 25
6.14.3.4. Severe Hypoglycemia 26

6.14.3.5. Immunogenicity....27
6.14.3.5.1. Definitions of Sample ADA Status....27
6.14.3.5.2. Definitions of Immunogenicity Assessment Periods....31
6.14.3.5.3. Definitions of Participant ADA Status....31
6.14.3.5.4. Analyses to be Performed....32
6.14.3.6. Hypersensitivity Reactions....32
6.14.3.6.1. Severe/Serious Hypersensitivity Reactions....33
6.14.3.7. Injection Site Reaction....33
6.14.3.7.1. Severe/Serious Injection Site Reactions....34
6.14.3.8. Major Adverse Cardiovascular Events....34
6.14.3.9. Renal Safety....35
6.14.3.9.1. Acute Renal Events....35
6.14.3.9.2. Dehydration....35
6.14.3.10. Thyroid Safety Monitoring....35
6.14.3.10.1. Calcitonin....35
6.14.3.10.2. C-Cell Hyperplasia and Thyroid Malignancies....36
6.14.3.11. Treatment-Emergent Supraventricular Arrhythmias and Cardiac Conduction Disorders....36
6.14.4. Vital Signs....36
6.14.5. Clinical Laboratory Evaluation....37
6.15. Subgroup Analyses....38
6.16. Interim Analyses and Data Monitor Committee....39
7. Unblinding Plan....40
8. References....41
9. Appendices....42

Table of Contents

Table **Page**

Table GPID.6.1. Description of Analysis Populations....................11

Table GPID.6.2. Baseline and Postbaseline Definitions for Safety Analyses....................11

Table GPID.6.3. Exploratory Efficacy Endpoints and Analyses....................21

Table GPID.6.4. Sample ADA Assay Results....................28

Table GPID.6.5. Sample Clinical ADA Interpretation Results....................29

Table GPID.6.6. *In Silico* Classification for Cross-Reactive NAb....................30

Table GPID.6.7. Adverse Events for Analysis with Immunogenicity Results....................32

Table GPID.6.8. Categorical Criteria for Abnormal Treatment-Emergent Blood Pressure and Heart Rate Measurements....................37

Table of Contents

Figure **Page**

Figure GPID.5.1. Illustration of study design for Clinical Protocol I8F-MC-GPID. 10

Figure GPID.6.1. Flowchart of immunogenicity multitiered testing approach. 28

Table of Contents

Appendix **Page**

Appendix 1. KCCQ – Scoring Instructions 43
Appendix 2. Searching Criteria For Special Safety Topics 48
Appendix 3. Cardiac Magnetic Resonance Imaging (MRI) Substudy 50

3. Revision History

This is the first version of the statistical analysis plan (SAP) for Study I8F-MC-GPID (GPID), which is approved prior to the first unblinding for an Independent Data Monitoring Committee review during the study.

4. Study Objectives

4.1. Primary Objective

To demonstrate that a maximally tolerated dose (MTD) of tirzepatide up to 15 mg administered subcutaneously once weekly (SC QW) is superior to placebo in participants with heart failure with preserved ejection fraction (HFpEF) and BMI ≥30 kg/m^2 based on:

- The hierarchical composite endpoint of
  - time to all-cause mortality through end of the treatment period
  - occurrence of adjudicated heart failure (HF) events through end of the treatment period, where HF events include HF hospitalization OR urgent HF visit
    - number of HF events
    - time to first HF event
  - change from baseline in 6-minute walk test distance (6MWD) category at Week 52
  - change from baseline in Kansas City Cardiomyopathy Questionnaire (KCCQ) Clinical Summary Score (CSS) category at Week 52
- Change from baseline to Week 52 in exercise capacity as measured by 6MWD

The effectiveness of tirzepatide will be demonstrated if one of the primary objectives is met. The details on type I error control are discussed in Section 6.13.3.

4.2. Key Secondary Objectives

The key secondary objectives are to demonstrate that tirzepatide MTD is superior to placebo with regards to

- percent change from baseline in body weight at Week 52;
- change from baseline in KCCQ-CSS at Week 52;
- change from baseline in 6MWD at Week 24;
- proportion of participants with NYHA Class change at Week 52

All the key secondary objectives are under multiplicity control. The details on type I error control are discussed in Section 6.13.3.

5. Study Design

Study GPID is a randomized, outpatient, multi-center, international, placebo-controlled, double-blinded parallel arm Phase 3 study with 2 study periods. The study is designed to evaluate the efficacy and safety of QW tirzepatide, maximum tolerated dose (MTD) up to 15 mg, compared to placebo, in participants with HFpEF and BMI ≥30 kg/m^2.

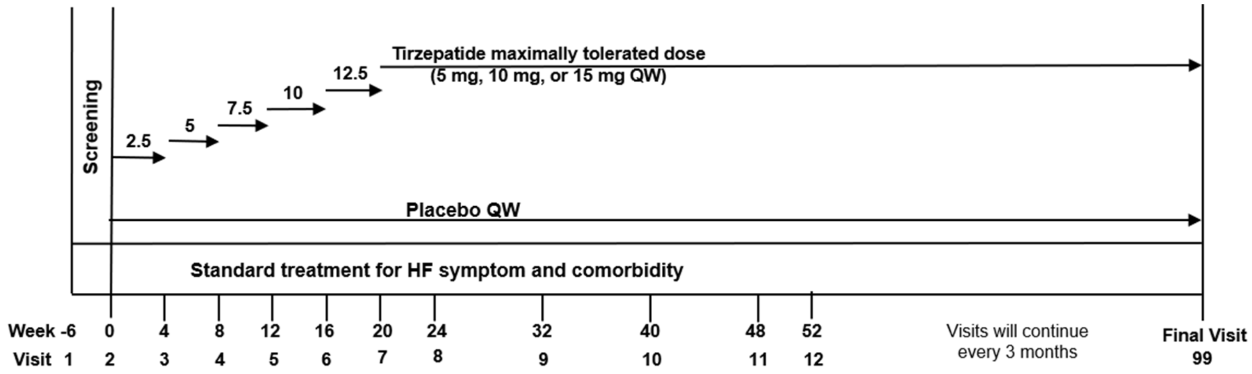

Note: Screening procedures may take longer or shorter than 6 weeks and variation in screening procedures will not be considered a protocol deviation.

Figure GPID.5.1. Illustration of study design for Clinical Protocol I8F-MC-GPID.

Two intervention groups will be studied:

- Tirzepatide MTD up to 15 mg SC QW
- Placebo

The study will compare treatment with tirzepatide and treatment with placebo. Assignment to tirzepatide or placebo groups will be randomly allocated in a 1:1 ratio.

The starting dose of tirzepatide is 2.5 mg QW, which is to be escalated at 4-week intervals to a maximum of 15 mg QW or to the highest maintenance dose tolerated by the participant.

The study will consist of 2 periods:

- Study Period 1: screening period, up to approximately 6 weeks
- Study Period 2: treatment period, with a 20-week escalation followed by at least a 32-week maintenance period

The study will continue until approximately 52 weeks after the last participant is randomized. The maximum duration of an individual's participation will depend on duration of study enrollment.

6. A Priori Statistical Methods

6.1. Populations for Analyses

The populations for analyses are defined in the following table (Table GPID.6.1).

Table GPID.6.1. Description of Analysis Populations

Analysis Population	Description
Entered	All participants who sign the informed consent form (ICF)
Randomized/Intent-to-Treat (ITT) Population	All participants assigned to treatment, regardless of whether they take any doses of study treatment, or if they took the correct treatment. Participants will be analyzed according to the treatment group to which they were assigned.
Safety Population	All participants in ITT population who take at least 1 dose of study treatment. Participants will be analyzed according to the treatment group to which they were assigned.

6.2. General Considerations

Statistical analysis of this study will be the responsibility of Eli Lilly and Company or its designee. Some analyses and summaries described in this analysis plan may not be conducted if not warranted by data (e.g., too few events to justify conducting an analysis). Additional analyses of the data may be conducted as deemed appropriate.

Statistical treatment comparisons will be performed between tirzepatide MTD and placebo. Unless otherwise specified, all tests of treatment effects will be conducted at a 2-sided alpha level of 0.05, and all confidence intervals (CIs) will be given at a 2-sided 95% level; efficacy will be assessed using ITT population, and baseline is defined as the last nonmissing observation collected prior to or at randomization for efficacy analyses; safety will be assessed using safety population, the definition of baseline and postbaseline for safety analyses are specified in Table GPID.6.2.

Table GPID.6.2. Baseline and Postbaseline Definitions for Safety Analyses

Analysis Type	Baseline	Postbaseline
1.1) Treatment-Emergent Adverse Events	The baseline period is defined as the start of screening and ends prior to the first dose of study treatment (typically at Week 0).	Starts after the first dose of study treatment and ends at the end of the study participation.
1.2) Treatment-Emergent Abnormal Labs[a] and Vital Signs	For labs, baseline period is defined as prior to or within 1 hour after the first dose time and will include all scheduled and unscheduled measurements.<br>For vital signs, baseline period is defined as measurements collected prior to or on the first dose day.	Postbaseline will be defined as after the baseline period through the end of the study participation. All scheduled and unscheduled measurements will be included.

Baseline and Postbaseline Definitions for Safety Analyses

Analysis Type	Baseline	Postbaseline
1.3) Change from Last Baseline to Week xx and to Last Postbaseline for Labs[a] and Vital Signs	The last scheduled and unscheduled non-missing assessment recorded during the baseline period defined above (1.2).	Postbaseline will be defined as above (1.2). Only scheduled visits will be included. The early termination (ET) visits are considered scheduled visits.

a Immunogenicity related analysis is specified in Section 6.14.3.5.

Summary descriptive statistics for continuous measures will include sample size, mean, standard deviation (SD), median, minimum, and maximum. Summary statistics for categorical measures (including categorized continuous measures) will include sample size, frequency, and percentages. Summary statistics for discrete count measures will include sample size, mean, SD, median, minimum, and maximum.

Unless otherwise specified, all observed data will be taken into account for analysis regardless of adherence to randomized treatment. Participants who discontinue study treatment early are asked to remain in the study and attend all scheduled visits for all data collection until end of study participation.

End of study participation for a participant will be the earliest of date of death, date of withdrawal from further participation in the study, or date of final visit. For participants considered to be lost-to-follow-up, end of study participation will be the date of lost-to-follow-up reported by the investigator. Participant data included in the database after the last date of study participation will be excluded from statistical analysis.

Not all analyses described in this SAP will necessarily be included in the Clinical Study Reports (CSRs). Any analysis described in this SAP and not provided in the CSR would be available upon request. Not all displays will necessarily be created as a "static" display. Some may be incorporated into interactive display tools instead of or in addition to a static display.

6.3. Adjustments for Covariates

The study is stratified by diagnosed T2DM (Yes/No), HF decompensation (including hospitalization for HF requiring IV diuretic treatment or urgent HF visit requiring IV diuretic treatment) within 12 months of screening (Yes/No), and BMI $\geq$ 35 kg/m^2 (Y/N). Unless otherwise specified, the stratification factors will be adjusted in efficacy analyses. The value for stratification factors will be obtained from the data collected or derived from the eCRF. In addition, the baseline value of the endpoint will be used as a covariate when appropriate.

6.4. Handling of Dropouts or Missing Data

For the primary and key secondary efficacy endpoint analyses subject to type 1 error rate control, missing data will be imputed based on the method described in Section 6.13.1.3 and 6.13.2.

For exploratory endpoints, missing values will not be explicitly imputed unless specified otherwise.

6.5. Multicenter Studies

There is no stratification by site or country for randomization. However, the country or regional effect may be examined for the primary endpoints through subgroup analysis.

6.6. Multiple Comparisons/Multiplicity

The type 1 error rate control strategy for primary and key secondary objectives is discussed in Section 6.13.3. There will be no multiplicity adjustments for evaluating exploratory objectives and safety assessments.

6.7. Patient Disposition

Summaries and a listing of study disposition and study drug disposition will be provided for all randomized participants. Comparison between treatment arms will be performed using Fisher's exact test.

6.8. Historical Illnesses and Preexisting Conditions

The count and percentages of participants with historical illnesses and preexisting conditions will be summarized by treatment group using the Medical Dictionary for Regulatory Activities (MedDRA) Preferred Term (PTs) nested within System Organ Class (SOC). The SOC will be in alphabetical order. Conditions (i.e., PTs) will be ordered by decreasing frequency in tirzepatide (TZP) MTD arm within SOC. This will be summarized for all randomized participants. Historical illnesses and preexisting conditions of special interest will also be summarized separately.

6.9. Patient Characteristics

A listing of participant demographics for all randomized participants will be provided. The demographic and baseline clinical characteristics will also be summarized by study treatment for all randomized participants. Baseline demographic and clinical characteristics of special interest include but are not limited to: age (years), sex (female, male), race, ethnicity, height (cm), weight (kg), BMI (kg/m^2), waist circumference (cm), age group (<65, ≥65; <75, ≥75), BMI group (<35, ≥35 and <40, ≥40 kg/m^2), country, vital signs, characterization of HFpEF and HFpEF-related comorbidities.

6.10. Concomitant Therapy

Concomitant medication will be summarized by treatment groups and displayed by decreasing frequency of WHODrug PTs in TZP MTD arm.

In addition, medications of interest (as defined below) will be summarized by treatment groups:

- Baseline use of
  - HF medications, by type/class
  - antiplatelet and anticoagulant medications, by type/class
  - antihyperglycemic medications, by type/class
  - lipid lowering therapy, by type/class

- o antihypertensive therapy other than HF medications, by type/class

6.11. Treatment Exposure and Compliance

6.11.1. Study and Study Treatment Exposure

Summary of duration of follow-up (defined as time in days from date of randomization to the date of the last study visit) will be provided by treatment group in ITT population; Summary of duration on study treatment (defined as time in days from date of first dose of study treatment to date of last dose of study treatment plus 7 days) will be provided by treatment group in safety population.

For the summary of duration on study treatment, the frequency and percentage of participants falling into the following categorical ranges will also be summarized by planned treatment group as well: >0 week, ≥4 weeks, ≥8 weeks, ≥12 weeks, ≥16 weeks, ≥20 weeks, ≥24 weeks, ≥32 weeks, ≥40 weeks, ≥48 weeks, ≥52 weeks, and every 12 weeks thereafter until the maximum duration of the treatment period.

No p-values will be reported in these summaries as they are intended to describe the study populations rather than test hypotheses.

6.11.2. Compliance to Study Treatment

Summary of prematurely discontinuing study treatment (including reason for discontinuation) will be provided by study treatment. A time-to-event analysis of premature study treatment discontinuation will also be conducted.

If data warrants, the counts and percentages of participants who follow the planned escalation scheme, have dose interruption, or have dose de-escalation will be summarized for each treatment group. In addition, the proportion of participants receiving 2.5 mg, 5 mg, 7.5 mg, 10 mg, 12.5 mg, or 15 mg may be presented by randomized treatment and visit during the dose escalation period.

Treatment compliance will be defined as taking at least 75% of the scheduled tirzepatide doses. Compliance at each treatment visit and over the whole treatment period will be calculated using the number of doses administered (regardless of the actual dose in mg administered) divided by the total number of doses expected to be administered ×100 at the specific visit or over the treatment period, respectively. Treatment compliance will be summarized descriptively at each treatment visit and over the treatment period by treatment using safety population.

6.12. Important Protocol Deviations

Important protocol deviations are identified in the Trial Issues Management Plan (TIMP). A listing and a summary of important protocol deviations by treatment will be provided.

6.13. Efficacy Analyses

The primary estimand for primary endpoints and key secondary endpoints is to assess the treatment difference between TZP and placebo relative to the efficacy measures for all

randomized participants, and treatment policy strategy will be used to handle intercurrent events (ICEs), meaning all the observed values for the variable of interest are used regardless of whether or not the intercurrent event occurs. The details of the primary estimand for each endpoint will be described in the following sections.

6.13.1. Primary Endpoints/Estimands Analysis

6.13.1.1. The Hierarchical Composite Endpoint

6.13.1.1.1. Estimand for the Endpoint

The primary estimand for the endpoint is defined as the win ratio of the TZP MTD vs placebo for the hierarchical composite endpoint. Specially, the estimand is described by the following attributes:

- Endpoint: A composite endpoint of all-cause mortality, occurrence of HF events through the end of the treatment period, the change from baseline in 6MWD, and the change from baseline in KCCQ-CSS at Week 52;
- Treatment condition of interest: tirzepatide MTD vs. placebo;
- Handling of intercurrent events: using treatment policy strategy to handle all intercurrent events;
- Population: all randomized participants;
- Population-level summary: win ratio (Pocock et al. 2012), calculated as number of pairs of tirzepatide-treated participant “wins” divided by number of pairs of placebo-treated participant “wins”. The winner is determined in each pair-wise comparison in the following order:
    - A delayed first occurrence of all-cause death;
    - If the pair cannot be differentiated based on mortality, a winner has fewer HF events;
    - If the pair cannot be differentiated by number of HF events, a winner has delayed time to the occurrence of first HF event;
    - If the pair still cannot be differentiated, a winner has a more favorable category for change from baseline in 6MWD at Week 52;
    - If the pair still cannot be differentiated, a winner has a more favorable category for change from baseline in KCCQ-CSS at Week 52;
    - Otherwise the pair will be recorded as tied.

The categories for change from baseline in 6MWD are: 1) ≥30% worsening; 2) ≥20% and <30% worsening; 3) ≥10% and <20% worsening; 4) No change (<10% change); 5) ≥10% and <20% improvement; 6) ≥20% and <30% improvement; 7) ≥30% improvement.

The categories for change from baseline in KCCQ-CSS are: 1) ≥10-point worsening; 2) ≥5- but <10-point worsening; 3) No change (<5-point change); 4) ≥5 but <10-point improvement; 5) ≥10-point improvement.

The KCCQ is a 23-item, participant self-administered questionnaire that assesses impacts of HF “over the past 2 weeks” on 7 domains (Green et al. 2000; Joseph et al. 2013). Each of the

23 individual items are answered on Likert scales of varying lengths (5-point, 6-point, or 7-point scales). Domain scores are obtained by averaging the associated individual items and transforming the score to a 0 to 100 range. Higher scores indicate better health status. KCCQ-CSS is a summary score that is derived by taking the mean of the Physical Limitation and Total Symptom scores. Detailed scoring instructions are provided in Appendix 1.

6.13.1.1.2. Main Analytical Approach

The analysis of the primary hierarchical composite endpoint will be performed with the Finkelstein-Schoenfeld (F-S) method (Finkelstein and Schoenfeld 1999), and the win ratio (Pocock et al. 2012) will be reported as the measure of treatment effect. The F-S method is based on the principle that each participant is compared with every other participant within each stratum in a pair-wise manner that proceeds in a hierarchical fashion. Participants will be stratified according to HF decompensation within 12 months of screening (Yes/No), diagnosed T2DM (Yes/No), and baseline BMI ≥ 35 kg/m^2 (Yes/No), yielding 8 stratification pools.

In the pair-wise comparison for all-cause death and HF events, the censoring for death and HF events will be handled based on the win ratio method (Pocock et al. 2012). When two participants have different follow-up times, the shorter follow-up time will be used to compare the clinical outcome measures. For example, if a participant with follow-up time of 55 weeks is compared with a participant with follow-up time of 60 weeks, the events occur after 55 weeks will not be counted in the winner determination for this pair of participants. Only adjudicated and confirmed endpoint events are included in the primary analysis. If an urgent HF visit is followed by a HF hospitalization within 7 days or vice versa, the urgent HF visit and the HF hospitalization will be considered as a single HF event; If a participant is hospitalized again due to HF within 14 days of discharge from a HF hospitalization, this will be considered as a single HF event.

The last measurement prior to randomization for 6MWD and KCCQ-CSS will be used as baseline. For missing 6MWD and KCCQ-CSS measurements at Week 52, multiple imputation will be utilized to impute the continuous values of the missing measurements (see Section 6.13.1.3 for details), and the imputed values will be categorized into the categories of change from baseline as defined in Section 6.13.1.1.1 for 6MWD and KCCQ-CSS for the pair-wise comparisons. The analysis for the primary hierarchical composite endpoint will be repeated using each dataset generated with multiple imputation, the results will be combined and final statistical inference over multiple imputations will be guided by the method proposed by Rubin (1987).

In addition, summary statistics of all observed data for each component will be reported by treatment arm, that will include number and percentage of participants with death, HFEs, rate of HFEs, and the number and percentage of participants that fall into each change category of 6MWD and KCCQ-CSS.

6.13.1.2. Change from Baseline in 6MWD

6.13.1.2.1. Estimand for the Endpoint

The estimand for the primary endpoint is described by the following attributes:

- Endpoint: Change from baseline to Week 52 in 6MWD;
- Treatment condition of interest: tirzepatide MTD vs. placebo;
- Handling of intercurrent events: using treatment policy strategy to handle all intercurrent events;
- Population: all randomized participants;
- Population-level summary: Hodges-Lehmann estimate of the median difference between treatment arms.

6.13.1.2.2. Main Analytical Approach

A stratified Wilcoxon test (van Elteren 1960) will be used as the main analysis method with controlling of the stratification factors of HF decompensation within 12 months of screening (Y/N), diagnosed T2DM (Y/N), and baseline BMI ≥ 35 kg/m^2 (Y/N). Hodges-Lehmann estimate for the median difference and 2-sided 99% and 95% confidence intervals will be reported.

Missing 6MWD measurements at Week 52 will be imputed through multiple imputations as specified in Section 6.13.1.3. The complete datasets generated through multiple imputations will be analyzed and Van Elteren test will be conducted for treatment comparison. The final statistical inference over multiple imputations will be guided by the method proposed by Rubin (1987).

6.13.1.3. Methods for Missing Data Imputation

The missing measurement for 6MWD and KCCQ-CSS at 52 weeks for the primary estimand will be imputed through multiple imputation based on the reason for missingness.

- For missing data due to exceptional circumstances, such as pandemics or natural disasters, the missing data is considered as missing at random (MAR) and multiple imputation will be performed using all nonmissing data from the same treatment arm.

- For missing measurements due to death, multiple imputation will be performed using the worst 15% observed data at 52 weeks from the same treatment group.
  For missing data due to all other ICEs or without ICE, retrieved dropout imputation will be applied, which will utilize observed data from participants in the same treatment group who had outcome measures at Week 52 after early discontinuation of study drug to impute the missing value. In case there are not enough retrieved dropouts to provide a reliable imputation model, reference to the placebo imputation will be used.

6.13.1.4. Sensitivity Analyses for Primary Endpoint

For the primary endpoint of change from baseline in 6MWD, a mixed-effect model for repeated measures (MMRM) analysis will be conducted to analyze the change from baseline in 6MWD. The analysis will be guided by treatment policy strategy. All values of the collected 6MWD data

at baseline, 24 weeks, and 52 weeks will be used in the MMRM analysis. The primary endpoint assessment will be the contrast between tirzepatide and placebo at Week 52.

The MMRM model will include treatment, time (Weeks 24 and 52), treatment-by-time interaction, stratification factors as fixed effects, and baseline value of the 6MWD as a covariate. Restricted maximum likelihood (REML) will be used to obtain model parameter estimates and the Kenward-Roger option will be used to estimate the denominator degrees of freedom. An unstructured covariance structure will be used to model the within-patient errors. If the analysis fails to converge, the following variance-covariance matrices will be used until convergence is achieved:

- heterogeneous Toeplitz
- heterogeneous first order autoregressive
- heterogeneous compound symmetry
- Toeplitz
- first order autoregressive, and
- compound symmetry.

In the MMRM analysis, the probability of missingness for any postbaseline 6MWD data that are not collected is assumed only to depend on the observed 6MWD values.

6.13.1.5. Supplementary Analyses

Supplementary analyses will be conducted to explore the clinically meaningful change in 6MWD and KCCQ through distribution-based methods and anchor-based methods. The details will be provided in the psychometric analysis plan (PAP) for the study.

6.13.2. Key Secondary Endpoints/Estimands

6.13.2.1. Change from Baseline in KCCQ-CSS at Week 52 and 6MWD at Week 24

6.13.2.1.1. Estimand for the Endpoints

The estimand is described by the following attributes:

- Endpoints:
  - Change from baseline to Week 52 in KCCQ-CSS;
  - Change from baseline to Week 24 in 6MWD;
- Treatment condition of interest: tirzepatide MTD vs. placebo;
- Handling of intercurrent events: using treatment policy strategy to handle all intercurrent events;
- Population: all randomized participants;
- Population-level summary: Hodges-Lehmann estimate of the median difference between treatment arms.

6.13.2.1.2. Main Analytical Approach

For the key secondary efficacy endpoints of change from baseline in KCCQ-CSS at Week 52 and change from baseline in 6MWD at Week 24, the same nonparametric approach as described

in Section 6.13.1.2.2 and the same missing data imputations as described in Section 6.13.1.3 will be utilized.

6.13.2.2. Percent Change from Baseline in Body Weight Loss at Week 52

6.13.2.2.1. Estimand for the Endpoint

- Endpoint: percent change from baseline to Week 52 in body weight
- Treatment condition of interest: tirzepatide MTD vs. placebo;
- Handling of intercurrent events: using treatment policy strategy to handle all intercurrent events;
- Population: all randomized participants;
- Population-level summary: difference of the least square means of the percent change between treatment arms.

6.13.2.2.2. Main Analytical Approach

For the percent body weight change from baseline, a mixed-effect model for repeated measures (MMRM) analysis similar to the MMRM model as described in Section 6.13.1.4 will be conducted. The response variable of MMRM will be the percent change in body weight from baseline values obtained at each scheduled postbaseline visit up to 52 weeks during the treatment period. The independent variables of the MMRM model will include the categorical effect of treatment, time, treatment-by-time interaction, stratification factors as fixed effects, and the continuous covariate of baseline body weight value as a covariate.

Missing body weight data at the scheduled postbaseline visits due to reasons other than exceptional circumstances will be imputed using retrieved dropout approach (as described in Section 6.13.1.3) through multiple imputation. The missing data due to exceptional circumstances will be implicitly imputed in the MMRM model. The final statistical inference over multiple imputations will be obtained using the method proposed by Rubin (1987).

6.13.2.3. Proportion of Participants with NYHA Class Change at Week 52

6.13.2.3.1. Estimand for the Endpoint

- Endpoints: proportion of participants with NYHA class change at Week 52
- Treatment condition of interest: tirzepatide MTD vs. placebo;
- Handling of intercurrent events: using treatment policy strategy to handle all intercurrent events other than death; For death, composite variable strategy will be utilized, specifically, for participants who die prior to NYHA measurements, the NYHA class change is considered as worsened.
- Population: all randomized participants;
- Population-level summary: odds ratio between treatment arms.

6.13.2.3.2. Main Analytical Approach

The categorical change in New York Heart Association Class (improved, no change, or worsened) from baseline will be analyzed using a longitudinal proportional odds model. The response variable of the analysis model will be the change in NYHA class from baseline

obtained at each scheduled postbaseline visit during treatment period (the visits at Week 24 and Week 52). The independent variables of the model will include the categorical effect of treatment, time, treatment-by-time interaction and the stratification factors, and baseline NYHA class as a covariate.

For missing NYHA change category data, as stated in Section 6.13.2.3.1, the category worsened is assigned for death; for missing data due to reasons other than exceptional circumstances at Week 24 and Week 52, the retrieved dropout approach as described in Section 6.13.1.3 will be used to impute the missing data through multiple imputation. The missing data due to exceptional circumstances will be implicitly imputed in the analysis model. The final statistical inference over multiple imputations will be obtained using the method proposed by Rubin (1987). Odds ratio and 95% CI relative to placebo will be reported for improved vs no change or worsened, and for improved or no change vs worsened.

6.13.2.4. Sensitivity Analyses for Key Secondary Endpoints

For the change from baseline in KCCQ-CSS, the MMRM analysis as described in Section 6.13.1.4 without utilizing multiple imputation as described in Section 6.13.1.3 will be conducted. For percent change from baseline in body weight, the MMRM analysis without utilizing multiple imputation as described in Section 6.13.2.2 will be conducted.

For the NYHA class change, the longitudinal proportional odds model as described in Section 6.13.2.3 will be repeated without missing data imputation.

6.13.2.5. Supplementary Analyses Related to Key Secondary Endpoints

To further evaluate the change from baseline to Week 52 in the body weight and in the KCCQ-CSS, a longitudinal logistic model will be utilized to compare the percentage of participants achieving ≥ 5%, ≥ 10% and ≥ 15% in body weight reduction respectively, and to compare the percentage of participants achieving ≥ 5-point, and ≥ 10-point improvement respectively in KCCQ-CSS. The independent variables for the model and variance-covariance structure to be used will be similar to the MMRM model as described in Section 6.13.1.4.

6.13.3. Type I Error Rate Control Strategy for Primary and Key Secondary Efficacy Analyses

All primary and key secondary hypotheses will be tested with the overall family-wise type I error rate at a 2-sided alpha level of 0.05 through the multiplicity control approach based on the graphical multiple testing procedure. For the 2 primary hypotheses, the hierarchical composite endpoint will be tested at a 2-sided alpha level of 0.04 and change in 6MWD will be tested at a 2-sided alpha level of 0.01 in parallel for statistical significance. If either is significant, the corresponding assigned alpha will be propagated to test the other primary efficacy endpoint at a 2-side alpha level of 0.05. If after this procedure both primary efficacy endpoints are significant, the alpha of 0.05 will be propagated to the key secondary efficacy endpoints. The detailed graphical testing scheme will be outlined in a later version of the SAP.

6.13.4. Exploratory Endpoints

Unless otherwise specified, exploratory efficacy analyses will be guided by treatment policy strategy using all randomized population. Missing data will not be imputed.

Table GPID.6.3. Exploratory Efficacy Endpoints and Analyses

Objectives	Endpoints	Analytical Approaches
HF medication use	Change in the HF concomitant medication net use:<br>• Intensification of diuretic use (defined as any new start and any dose increase)	The time from randomization to the first occurrence of any new start or dose increase of diuretics will be analyzed by a Cox proportional hazard model stratified by the study stratification factors with treatment as a fixed effect.
Clinical outcome events of HF	Time to first occurrence of<br>• HF events and CV death<br>• HF events and all-cause death<br>• HF events<br>Time to recurrent events of<br>• HF events and CV death<br>• HF events	The time from randomization to the first occurrence of any component of the composite endpoint will be analyzed by a Cox proportional hazard model stratified by the study stratification factors with treatment as a fixed effect.<br>Time to recurrent event analyses will be performed using a joint frailty model stratified by the study stratification factors with treatment as a fixed effect.<br>Investigator-reported and CEC-adjudicated events will be analyzed respectively.
Atrial fibrillation (AF)	Proportion of participants with AF	Logistic regression analysis will be conducted with AF status at 24 weeks and 52 weeks as the dependent variable and include treatment, stratification factors, and baseline AF status in the model.
Waist circumference	Change from baseline in waist circumference	MMRM analysis similar to the model described in Section 6.13.1.4 will be conducted.
Patient-reported health-related quality of life	Change from baseline in KCCQ:<br>• Total Symptom Score (TSS)<br>• Overall Summary Score (OSS)	MMRM analysis similar to the model described in Section 6.13.1.4 will be conducted.
Patient-reported health status	Change from baseline in EQ-5D-5L (measured at 24 weeks and 52 weeks):<br>• Index Score<br>• VAS Score	MMRM analysis similar to the model described in Section 6.13.1.4 will be conducted.
Patient-reported Impression of Status (PGIS)	Proportion of participants with improvements from baseline in:<br>• PGIS – Overall<br>• PGIS – Physical Function<br>• PGIS – Symptom Severity	Proportion of participants with improvements from baseline will be summarized. Shift analysis from baseline to Week 24 and to Week 52 will also be performed.

Exploratory Efficacy Endpoints and Analyses

Objectives	Endpoints	Analytical Approaches
Evaluation of prespecified biomarkers	Change from baseline in:<br>• NT-proBNP<br>• cTNT<br>• hs-CRP	MMRM analysis similar to the model described in Section 6.13.1.4 will be conducted. The data will be log-transformed for the analysis.

Abbreviations: CEC = clinical endpoint committee; cTNT = cardiac troponin T; CV = cardiovascular; EQ-5D-5L = EuroQOL 5 Dimension 5 Level scale; HF = heart failure; hs-CRP = high-sensitivity C-reactive protein; KCCQ = Kansas City Cardiomyopathy Questionnaire; NT-proBNP = N-terminal pro-B-type natriuretic peptide; VAS = visual analog scale.

6.14. Safety Analyses

Unless specified otherwise, safety analyses will be conducted in the safety population (Table GPID.6.1); all events that occur between the first dose date of study drug and the end date of study participation will be included, regardless of the adherence to study drug.

The statistical assessment of homogeneity of the distribution of categorical safety responses between tirzepatide MTD and placebo will be conducted using Fisher's exact test, unless specified otherwise.

The mean change from baseline differences among treatments at all scheduled visits will be assessed via an MMRM using REML. Unless specified otherwise, the MMRM analysis will contain measurements up to 52 weeks, and the model will include treatment group, stratification factors, visit and treatment-by-visit interaction as fixed effects, and baseline value of the safety parameter as a covariate. To model the covariance structure within participants, the unstructured covariance matrix will be used. If this model fails to converge, the covariance structures specified in Section 6.13.1.4 will be tested in order until met convergence. If the data does not warrant the MMRM model, then an ANCOVA model will be conducted.

For selected safety parameters, time-to-first-event analysis via the Cox-proportional hazards model may be conducted. Participants without the event will be censored at the end of study participation. For participants experiencing the event, the "time-to-first-event" will be the time (in days) from first dose to first occurrence of the event.

6.14.1. Analysis of Adverse Events

6.14.1.1. Treatment Emergent Adverse Events

A treatment-emergent adverse event (TEAE) is defined as an event that first occurred or worsened in severity after the first dose of study treatment. The MedDRA Lowest Level Term (LLT) will be used in the treatment-emergent derivation. The maximum severity for each LLT during the baseline period including ongoing medical history will be used as baseline severity. For events with a missing severity during the baseline period, it will be treated as "mild" in severity for determining treatment-emergence. Events with a missing severity during the postbaseline period will be treated as "severe" and treatment-emergence will be determined by comparing to baseline severity.

For events occurring on the day of taking study medication for the first time, the case report form (CRF)-collected information (e.g., treatment emergent flag, start time of study treatment and event) will be used to determine whether the event was pre- versus post-treatment if available. If the relevant information is not available, then the events will be counted as post-treatment.

Unless otherwise specified, the counts and percentages of participants with TEAEs will be summarized by treatment using MedDRA PT nested within System Organ Class (SOC). Statistical comparisons will be applied at both the SOC and PT levels. Events will be ordered by decreasing frequency in TZP arm within SOC. The SOC will be in alphabetical order. For events that are sex-specific, the denominator and computation of the percentage will include only participants from the given sex.

An overview of the number and percentage of participants who experienced a TEAE, serious adverse event (SAE), death, discontinued from study treatment or study due to an AE, or with a TEAE related to study treatment will be summarized by treatment.

The counts and percentages of patients with TEAEs by maximum severity will be summarized by treatment using MedDRA PT. For each participant and TEAE, the maximum severity for the MedDRA PT is the maximum postbaseline severity observed from all associated LLTs mapping to the MedDRA PT. The maximum severity will be determined based on the nonmissing severities. If all severities are missing for the defined postbaseline period of interest, it will show as missing in the table.

6.14.1.2. Common Adverse Events

The counts and percentages of participants with TEAEs, overall and common (common TEAEs occurred in ≥5% of participants before rounding), will be summarized by treatment using MedDRA PT. Events will be ordered by decreasing frequency in TZP arm.

6.14.1.3. Deaths

A listing of all deaths during the study will be provided. The listing will include participant identification including the treatment, site number, date of death, age at the time of enrollment, sex, associated AE group identification, time from last dose of study drug to death (if participant had discontinued study drug), and primary cause of death.

6.14.1.4. Other Serious Adverse Events

The counts and percentages of participants who experienced an SAE (including deaths and SAEs temporally associated or preceding deaths) during the postbaseline period will be summarized by treatment using MedDRA PT nested within SOC. Events will be ordered by decreasing frequency in TZP arm within SOC. The SOC will be in alphabetical order.

A listing of all SAEs will be provided. The listing will include treatment, participant identification including the site number, date of event, age at the time of enrollment, sex, AE group identification, MedDRA SOC and PT, severity, outcome, relationship to study drug, time from first dose of study drug to the event, and time from most recent dose to event (if participant discontinued study drug prior to the event), investigator-reported endpoint indicator, and clinical endpoint committee (CEC) -adjudicated endpoint indicator.

6.14.1.5. Other Significant Adverse Events

The counts and percentages of participants who discontinued from study treatment or study due to an AE during the postbaseline period may be summarized by treatment group using MedDRA PT nested within SOC. Events will be ordered by decreasing frequency in TZP arm within SOC.

6.14.2. Patient Narratives

Patient narratives will be provided for all participants who experience any of the following "notable" events:

- death
- serious adverse event,
- pregnancy, or
- permanent discontinuation of study treatment due to AEs.

Patient narratives (patient level data and summary paragraph) will be provided for participants in the randomized population with at least 1 notable event.

6.14.3. Special Safety Topics

For adverse events of special interest (AESI) or special safety topics, the counts and percentages of participants will be summarized by treatment and PT with decreasing frequency in TZP arm if the overall count is ≥10. Individual participant level data may be presented. Displays with individual participant level data may be created using various formats, such as a customized listing and/or a customized graphical participant profile. Adverse events of special interest (AESI) are defined in each section of special safety topics, where applicable.

6.14.3.1. Exocrine Pancreas Safety

6.14.3.1.1. Pancreatic Enzyme

Observed pancreatic enzyme data (p-amylase and lipase) will be summarized by treatment and nominal visit.

The counts and percentages of participants with maximum postbaseline pancreatic enzyme value exceeding the following thresholds will be provided by baseline pancreatic enzyme value (≤ upper limit of normal [ULN], > ULN), and postbaseline: ≤1x ULN, (>1 to ≤3) x ULN, (>3 to ≤5) x ULN, (>5 to ≤10) x ULN, >10x ULN.

An MMRM analysis will be used to analyze each pancreatic enzyme with a log transformed (postbaseline measure/baseline measure) response variable and treatment, nominal visit, treatment-by-nominal visit interaction as fixed effects.

6.14.3.1.2. Pancreatitis Events

Summaries of adjudicated and investigator-reported pancreatic events will be provided by treatment. Detailed searching criteria can be found in Appendix 2.

Treatment emergent adjudication confirmed pancreatitis will be considered as AESI. Listing of participants with adjudicated pancreatitis may be provided if deemed necessary.

6.14.3.2. Gastrointestinal Adverse Events

6.14.3.2.1. Nausea, Vomiting, and Diarrhea

Summaries and analyses for incidence and severity of nausea, vomiting (including "vomiting" and "vomiting projectile"), diarrhea (including "diarrhea" and "diarrhoea"), and 3 events combined, will be provided by each treatment group.

Summary of the prevalence over time for nausea, vomiting, and diarrhea will also be presented. Time to the onset of nausea, vomiting, and diarrhea will be plotted.

6.14.3.2.2. Severe Gastrointestinal Events

The PTs under the gastrointestinal (GI) SOC in MedDRA will be used to identify GI AEs, and only the PTs with serious/severe treatment-emergent cases will be considered as AESIs.

The counts and percentages of participants with severe/serious treatment-emergent GI events may be summarized by treatment, or a listing may be provided.

6.14.3.3. Hepatobiliary Disorders

6.14.3.3.1. Hepatobiliary Events

Severe/serious treatment-emergent hepatobiliary disorders will be considered as AESIs. The counts and percentages of participants with treatment-emergent potentially drug-related hepatobiliary events may be summarized by treatment using the MedDRA PTs. The detailed search criteria can be found in Appendix 2.

Events related to acute gallbladder disease may also be summarized or a listing may be provided. The search criteria can be found in Appendix 2.

6.14.3.3.2. Liver Enzymes

Common analyses for laboratory analyte measurements described in Section 6.14.1.5 are applicable for the liver enzyme related measurements. This section provides additional analyses for liver enzymes.

The counts and percentages of participants with the following elevations in hepatic laboratory tests at any time during the study will be summarized between treatment groups:

- The counts and percentages of participants with an alanine aminotransferase (ALT) measurement $\geq 3 \times$, $5 \times$, and $10 \times$ ULN will be summarized for all participants with a postbaseline value and for subsets based on the following levels of baseline value.
    - participants whose nonmissing maximum baseline value is $\leq 1 \times$ ULN,
    - participants whose maximum baseline is $>1 \times$ ULN,
    - participants whose baseline values are missing.
- The counts and percentages of participants with an aspartate aminotransferase (AST) measurement $\geq 3 \times$, $5 \times$, and $10 \times$ ULN during the study will be summarized for all patients with a postbaseline value and for subsets based on various levels of baseline, as described above for ALT.

- The counts and percentages of participants with a total bilirubin (TBL) measurement ≥2 × ULN during the study will be summarized for all participants with a postbaseline value, and for the following subsets based on the baseline values:
    - participants whose nonmissing maximum baseline value is ≤1 × ULN,
    - participants whose maximum baseline is >1 × ULN, but <2 × ULN,
    - participants whose maximum baseline value is ≥2 × ULN, and
    - participants whose baseline values are missing.
- The counts and percentages of participants with a serum alkaline phosphatase (ALP) measurement ≥2 × ULN during the study will be summarized for all participants with a postbaseline value, and the same subsets as described for TBL.

Maximum baseline will be the maximum nonmissing observation in the baseline period. The maximum value will be the maximum nonmissing value from the postbaseline period. Planned and unplanned measurements will be included.

6.14.3.4. Severe Hypoglycemia

The following categories in accordance with the 2019 American Diabetes Association position statement on glycemic targets (ADA 2019) will be defined in the database.

Glucose Alert Value (Level 1):

- **Documented symptomatic hypoglycemia** is defined as any time a patient feels that he/she is experiencing symptoms and/or signs associated with hypoglycemia, and has a blood glucose (BG) level of <70 mg/dL (<3.9 mmol/L).
- **Documented asymptomatic hypoglycemia** is defined as any event not accompanied by typical symptoms of hypoglycemia, but with a measured BG <70 mg/dL (<3.9 mmol/L).
- **Documented unspecified hypoglycemia** is defined as any event with no information about symptoms of hypoglycemia available, but with a measured BG <70 mg/dL (<3.9 mmol/L).

Documented Clinically Significant Hypoglycemia (Level 2):

- **Documented symptomatic hypoglycemia** is defined as any time a participant feels that he/she is experiencing symptoms and/or signs associated with hypoglycemia and has a BG level of <54 mg/dL (<3.0 mmol/L).
- **Documented asymptomatic hypoglycemia** is defined as any event not accompanied by typical symptoms of hypoglycemia but with a measured BG <54 mg/dL (<3.0 mmol/L).
- **Documented unspecified hypoglycemia** is defined as any event with no information about symptoms of hypoglycemia available but with a measured BG <54 mg/dL (<3.0 mmol/L).

Severe Hypoglycemia (Level 3):

Severe hypoglycemia is defined as an episode with severe cognitive impairment requiring the assistance of another person to actively administer carbohydrate, glucagon, or other resuscitative actions. These episodes may be associated with sufficient neuroglycopenia to induce seizure or coma. Plasma glucose (PG) measurements may not be available during such an event, but

neurological recovery attributable to the restoration of PG to normal is considered sufficient evidence that the event was induced by a low PG concentration.

Other hypoglycemia categories:

Nocturnal hypoglycemia is defined as any hypoglycemic event that occurs between bedtime and waking. If a hypoglycemic event meets the criteria of severe, the event would specifically be collected as an SAE. Serious hypoglycemia is defined by pharmacovigilance criteria and will also be captured with a SAE form.

To avoid duplicate reporting, all consecutive hypoglycemic events occurring within a 1-hour period will be considered to be a single hypoglycemic event.

Severe/serious hypoglycemia may be summarized by treatment group, or a listing may be provided.

6.14.3.5. Immunogenicity

6.14.3.5.1. Definitions of Sample ADA Status

At a high level, an individual sample is potentially examined multiple times, in a hierarchical procedure, to produce a sample anti-drug antibodies (ADA) assay result and potentially multiple cross-reactive antibodies assay results and multiple neutralizing antibodies (NAb) assay results.

The cut points used, the drug tolerance of each assay, and the possible values of titers are operating characteristics of the assay. Figure GPID.6.1 details a flow chart that reflects the multitiered testing approach.

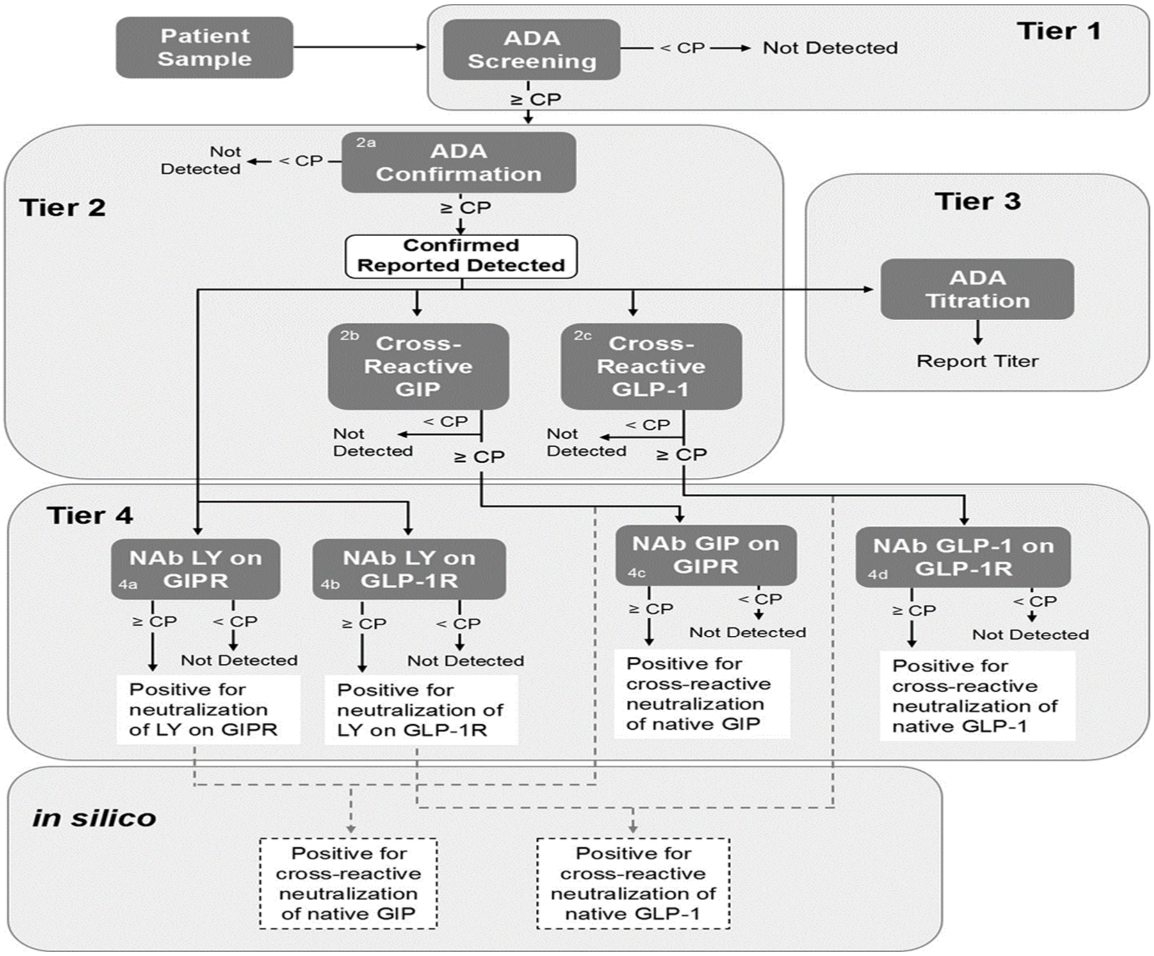

Abbreviations: ADA = anti-drug antibodies; CP = cut point; GIP = glucose-dependent insulinotropic polypeptide; GIPR = glucose-dependent insulinotropic polypeptide receptor; GLP-1 = glucagon-like peptide-1; GLP-1R = glucagon-like peptide-1 receptor; LY = LY3298176; NAb =neutralizing antibodies.

Figure GPID.6.1. Flowchart of immunogenicity multitiered testing approach.

Table GPID.6.4 outlines results as reported from Tier 2a of the multitiered testing approach. Tier 4 results are reported similarly.

Table GPID.6.4. Sample ADA Assay Results

Sample Laboratory Result	Explanation
Detected	ADA are detected and confirmed.
Not Detected	The raw result as reported from the laboratory indicates not detected. The clinical interpretation of such results depends on other factors (see Table GPID.6.5).
NO TEST, QNS, etc.	Sample exists but was unevaluable by the assay.

Abbreviations: ADA = anti-drug antibodies; QNS = quantity not sufficient.

It can be the case that the presence of high concentrations of tirzepatide will affect ADA immunoassays, and conversely high levels of ADA may affect the measurement of tirzepatide concentration. Thus, a tirzepatide drug concentration, assessed from a sample drawn at the same time as the ADA sample, plays a key role in clinical interpretation of a sample when the laboratory results is Not Detected (see Table GPID.6.5).

Table GPID.6.5. Sample Clinical ADA Interpretation Results

Sample Clinical Interpretation	Explanation
ADA Present	ADA assay result is Detected
ADA Not Present	ADA assay result is Not Detected and simultaneous drug concentration is at a level that has been demonstrated to not interfere in the ADA detection method (i.e., drug concentration is below the assay's drug tolerance level).<br><br>For participants receiving placebo, drug concentration is not assessed and is assumed to be below the assay's drug tolerance level.<br><br>If drug concentration was planned but is not available for a treatment-period sample, a Not Detected sample will be declared ADA Not present.
ADA Inconclusive	ADA assay result is Not Detected but drug concentration in the sample is at a level that can cause interference in the ADA detection method.
ADA Missing	ADA sample not drawn, QNS, not tested, etc., causing there to be no laboratory result reported or the result is reported as "no test."

Abbreviations: ADA = antidrug antibodies; QNS = quantity not sufficient.

All ADA present samples will be evaluated for cross-reactive GIP (Tier 2b), cross-reactive GLP-1 (Tier 2c), NAb LY (tirzepatide) on GIP-R (Tier 4a), and NAb LY (tirzepatide) on GLP-1R (Tier 4b). If cross-reactive GIP is detected, NAb GIP on GIP-R (Tier 4c) is evaluated. If cross-reactive GLP-1 is detected, NAb GLP-1 on GLP-1R (Tier 4d) is evaluated (Figure GPID.6.1).

Similar terminology to Table GPID.6.6 applies for each type of cross-reactive and NAb assay. Importantly, each of these are distinct assays and, in general, have different assay operating characteristics.

The following are considered inconclusive for the NAb result:

- NAb LY on GIP-R: if NAb result is not detected, and pharmacokinetic (PK) concentration is ≥ drug tolerance limit of the NAb LY on GIP-R assay
- NAb LY on GLP-1R: if NAb result is not detected, and PK concentration is ≥ drug tolerance limit of the NAb LY on GLP-1R assay
- NAb GIP on GIP-R: if NAb result is not detected, and PK concentration is ≥ drug tolerance limit of the NAb GIP on GIP-R assay
- NAb GLP-1 on GLP-1R: if NAb result is not detected, and PK concentration is ≥ drug tolerance limit of the NAb GLP-1 on GLP-1R assay

To mitigate inconclusive cross-reactive NAb interpretations against native GIP and GLP-1 due to potential tirzepatide concentrations greater than or equal to the drug tolerance limit of the NAb GIP on GIP-R (Tier 4c) and NAb GLP-1 on GLP-1R (Tier 4d) assays, an *in silico* method utilizing results from Tiers 2b and 2c, Tiers 4a and 4b, and tirzepatide concentrations is introduced. The *in silico* method is outlined in the following table:

Table GPID.6.6. *In Silico* Classification for Cross-Reactive NAb

***In Silico* Classification**	**Cross-Reactive ADA Result**	**NAb Result**	**Circulating Tirzepatide Level (ng/mL)**	***In Silico* Cross-Reactive NAb Interpretation**
Cross-Reactive Nab to nGIP	Tier 2b: "Not Detected"	Tier 4a "Not Detected"<br>Or<br>Tier 4a: "Detected" or N/A or Missing	Any Value or Missing	Not Present
	Tier 2b: "Detected"	Tier 4a: "Not Detected"	< drug tolerance limit of Tier 4a assay	Not Present
	Tier 2b: "Detected"	Tier 4a: "Not Detected"	≥ drug tolerance limit of Tier 4a assay	Inconclusive
	Tier 2b: "Detected"	Tier 4a: "Detected"	< drug tolerance limit of Tier 4a assay	Present
	Tier 2b: "Detected"	Tier 4a: "Detected"	≥ drug tolerance limit of Tier 4a assay	Present
Cross-Reactive NAb to nGLP-1	Tier 2c: "Not Detected"	Tier 4b "Not Detected"<br>Or<br>Tier 4b: "Detected" or N/A or Missing	Any Value or Missing	Not Present
	Tier 2c: "Detected"	Tier 4b: "Not Detected"	< drug tolerance limit of Tier 4b assay	Not Present
	Tier 2c: "Detected"	Tier 4b: "Not Detected"	≥ drug tolerance limit of Tier 4b assay	Inconclusive
	Tier 2c: "Detected"	Tier 4b: "Detected"	< drug tolerance limit of Tier 4b assay	Present
	Tier 2c: "Detected"	Tier 4b: "Detected"	≥ drug tolerance limit of Tier 4b assay	Present

Abbreviations: ADA = antidrug antibodies; NAb = neutralizing antibody; nGIP = native glucose-dependent insulinotropic polypeptide; nGLP-1 = native glucagon-like peptide-1; Tier 2b = cross-reactive ADA to nGIP; Tier 2c = cross-reactive ADA to nGLP-1; Tier 4a = NAb LY (tirzepatide) on GIPR; Tier 4b = NAb LY (tirzepatide) on GLP-1R.

Note: Only the drug tolerance limits of the Tier 4a and 4b assays are used for in silico classifications as they are lower than the drug tolerance limits of the Tier 2b and 2c assays, respectively.

6.14.3.5.2. Definitions of Immunogenicity Assessment Periods

Immunogenicity Baseline Observations: Baseline period for immunogenicity assessment for each participant includes all observations prior to first dose of study treatment. In instances where multiple baseline observations are collected, to determine participant ADA status the last nonmissing immunogenicity assessment prior to first administration of study drug is used to determine treatment-emergent status (see below).

Immunogenicity Postbaseline Period Observations: Postbaseline period observations for each participant includes all observations after the first administration of study drug.

6.14.3.5.3. Definitions of Participant ADA Status

Treatment-emergent (TE) ADA-evaluable participants: A participant with a nonmissing baseline ADA result and at least 1 nonmissing postbaseline ADA result.

TE ADA-unevaluable participant: any participant who does not meet the evaluable criteria.

Baseline ADA Present (preexisting antibody): ADA detected in a sample collected up to the first dose date and time.

Baseline ADA Not Present: ADA is not detected, and the corresponding PK concentration is missing or below the drug tolerance limit in a sample collected up to the first dose date and time.

Treatment-emergent ADA positive (TE ADA+) participant: An evaluable participant who had a:

- baseline status of ADA Not Present and at least 1 postbaseline status of ADA Present with titer ≥2 × minimum required dilution (MRD), where the MRD is the minimum required dilution of the ADA assay or
- baseline and postbaseline status of ADA Present, with the postbaseline titer being 2 dilutions (4-fold) greater than the baseline titer. That is, the participant has baseline (B) status of ADA Present, with titer 1:B, and at least 1 postbaseline (P) status of ADA Present, with titer 1:P, with P/B ≥ 4.

As shown in Figure GPID.6.1, a titer is expected when ADA assay result is Detected. On occasion, the corresponding assay cannot be performed, in which case a titer value will be imputed for the purpose of TE ADA determination. A baseline sample with detected ADA and no titer is imputed to be the MRD (1:10), and a postbaseline sample with ADA detected and no titer is imputed to be one dilution above the MRD (1:20).

TE ADA-Inconclusive participant: A TE ADA-evaluable participant is TE ADA Inconclusive if ≥20% of the participant's postbaseline samples, drawn pre-dose, are ADA Inconclusive and all remaining postbaseline samples are ADA Not Present.

TE ADA-negative (TE ADA-) participant: A TE ADA-evaluable participant is TE ADA- when the participant is not TE ADA+ and not TE ADA Inconclusive.

For each NAb assay, the following are defined:

NAb positive (NAb+) participant: A participant who is TE ADA+ and has a NAb positive sample in the postbaseline period.

NAb Inconclusive participant: A participant who is TE ADA+, is not NAb+, and all samples that have TE ADA+ titer have a NAb Inconclusive sample result.

NAb negative (NAb-) participant: A participant is neither NAb+ nor NAb Inconclusive.

Unless specified otherwise, the above-mentioned definitions of NAb are applicable to all NAb analyses, including cross-reactive NAb analyses, and cross-reactive antibodies.

6.14.3.5.4. Analyses to be Performed

The count and proportion of participants who are TE ADA+ will be tabulated by treatment group, where proportion are relative to the number of TE ADA-evaluable participants, as defined above. The tabulation will include the count and proportion of participants with ADA Present at baseline, and the count and proportion of TE ADA+ participants exhibiting each type of cross-reactive antibodies and NAb. This analysis will be performed for the planned treatment period.

The cross-reactive Nab will exclude Tier 4c and 4d results but include the *in silico* classification as cross-reactive NAb for summary.

A summary will be provided of the count and percentage of tirzepatide-treated participants experiencing specific TEAE (see Table GPID.6.7) by participant TE ADA status (TE ADA+, TE ADA-, TE ADA Inconclusive). The PT will be ordered by decreasing incidence in TE ADA+ status group.

Table GPID.6.7. Adverse Events for Analysis with Immunogenicity Results

TEAE category	Criteria
Hypersensitivity reactions	Anaphylaxis SMQ (narrow or algorithm)
	Hypersensitivity SMQ (narrow)
	Angioedema SMQ (narrow)
	Severe Cutaneous Adverse Reaction SMQ (narrow)
Injection site reactions	Injection site reaction HLT
	Infusion site reaction HLT
	Administration site reaction HLT

Abbreviations: HLT = high-level term; MedDRA = Medical Dictionary for Regulatory Activity; SMQ = standardized MedDRA query; TEAE = treatment-emergent adverse event.

Additional immunogenicity analyses as determined later may be presented. The relationship between antibody titers, the PK parameters, and pharmacodynamics (PD) response to tirzepatide may also be assessed.

Cases of TE ADA that are associated with TEAEs of either severe/serious hypersensitivity or severe/serious injection site reaction (ISR) will be classified as AESIs.

6.14.3.6. Hypersensitivity Reactions

Two main analyses are performed in support of assessment of potential immediate hypersensitivity, including anaphylaxis as well as potential nonimmediate hypersensitivity.

Time Period A, of potential immediate hypersensitivity includes all TEAEs occurring from start of study drug administration up to 24 hours after end of study drug administration. For events without the hypersensitivity eCRF, only date (no time) information are collected. Among these events without time information, the event occurred on the same date as the study drug injection date will be included in Time Period A.

Time Period B, of potential non-immediate hypersensitivity, includes all TEAEs occurring more than 24 hours after the end of study drug administration, but prior to subsequent drug administration.

Analyses for both time periods are based on the following:

- Narrow and algorithm terms in Anaphylactic reaction SMQ (20000021) (analysis for algorithm term only applicable for Time Period A)
- Narrow terms in Angioedema SMQ (20000024)
- Narrow terms in Severe cutaneous adverse reactions SMQ (20000020)
- Narrow terms in Hypersensitivity SMQ (20000214)

For the Anaphylactic reaction SMQ, each term is classified by scope (Narrow, Broad) and by category (A, B, C, D). All Narrow terms are category A, and all Broad terms are category B, C, or D. In addition to the usual Narrow and Broad searches, the SMQ defines an algorithm to further refine the cases of interest. For time period A analysis, the Anaphylactic reaction SMQ algorithm will be included. The algorithm is based upon events that occur within Time Period A. The counts and percentages of participants who experienced a TEAE for the following will be analyzed for each of the 2 time periods:

- Any narrow term from any one of the 4 SMQs indicated above (i.e., combined search across narrow of all 4 SMQs)
- Any narrow scope term within each SMQ, separately (i.e., narrow SMQ search). For Time Period A analysis, any term from Anaphylactic reaction SMQ algorithm.

Within query, individual PTs that satisfied the queries will be summarized. For Time Period A analysis, the Anaphylactic reaction SMQ algorithm will be summarized. Also, a single event may satisfy multiple SMQ, in which case the event contributes to every applicable SMQ.

6.14.3.6.1. Severe/Serious Hypersensitivity Reactions

The severe/serious cases of hypersensitivity will be considered as AESIs. Summary of severe/serious hypersensitivity reactions or listing may be provided.

6.14.3.7. Injection Site Reaction

Injection site reaction, incidence and rates, and related information reported via “Injection Site Reactions” eCRF will be summarized by treatment. Information to be summarized include location of the reaction, timing of reaction relative to study drug administration, and characteristics of the injection site reaction: erythema, induration, pain, pruritus and edema.

Patient-based analysis and event-based analysis may be provided if necessary. The patient-based analysis summarizes all injection-site reaction (ISR) questionnaire forms for an individual

patient with a single statistic, typically an extreme value. This analysis allows each patient to contribute only once for each parameter, at the expense of a focus on the most extreme events. By contrast, the event-based analysis summarizes all ISR questionnaire forms received, without regard to individual patients. This provides characteristics of ISR events as a proportion of all events for which questionnaire responses were provided, at the expense of some potential bias due to differential contribution of individual patients to the analysis.

The counts and percentages of participants with treatment emergent injection site reaction will be summarized by treatment using the MedDRA PTs. Detailed searching criteria can be found in Appendix 2.

The PT will be listed for summary in decreasing order of incidence for tirzepatide-treated participants.

6.14.3.7.1. Severe/Serious Injection Site Reactions

The severe/serious injection site reactions (e.g., abscess, cellulitis, erythema, hematomas/hemorrhage, exfoliation/necrosis, pain, subcutaneous nodules, swelling, induration, inflammation) will be considered as AESI.

The counts and percentage of participants with severe/serious ISRs may be summarized by treatment, or a listing of participants with treatment-emergent severe/serious ISRs may be provided.

6.14.3.8. Major Adverse Cardiovascular Events

Major adverse cardiovascular events (MACE) reported by investigators are adjudicated by an independent CEC in a blinded fashion.

The following positively adjudicated MACE will be considered as AESI:

- myocardial infarction (MI),
- hospitalization for unstable angina,
- coronary interventions (such as coronary artery bypass graft [CABG] or percutaneous coronary intervention [PCI]),
- cerebrovascular events, including cerebrovascular accident (stroke) and transient ischemic attack (TIA).

Cardiovascular death and hospitalization for heart failure are not considered as AESI since they are reported as efficacy endpoints in this study.

The counts and percentages of participants with adjudicated MACE may be summarized by treatment. In addition, MACE reported by investigator may also be summarized although a MACE reported by investigator is not considered as AESI.

A listing of participants reporting MACE events, either reported by investigator or identified by the CEC, may be provided.

6.14.3.9. Renal Safety

Laboratory measures related to renal safety will be analyzed as specified for laboratory measurements in Section 6.14.5.

In addition, two shift tables examining renal function will be created. A min-to-min shift table of estimated glomerular filtration rate (eGFR) estimated by the Chronic Kidney Disease Epidemiology Collaboration (CKD-EPI) equation with units ml/min/1.73m^2, using categories (<30, ≥30 to <45, ≥45 to <60, ≥60 to <90, and ≥90 mL/min/1.73m^2). A max-to-max shift table of urine albumin-to-creatinine ratio (UACR), using the categories UACR <30 mg/g, 30 mg/g ≤ UACR ≤300 mg/g, UACR >300 mg/g (respectively, these represent normal, microalbuminuria, and macroalbuminuria).

Mixed model repeated measure analyses as in Section 6.14 for eGFR and UACR will be provided. Log transformation will be performed for UACR.

6.14.3.9.1. Acute Renal Events

Because severe GI events may lead to dehydration, which could cause a deterioration in renal function including acute renal failure, dehydration events will be analyzed. Acute renal events associated with chronic renal failure exacerbation will also be captured.

Severe/serious renal events from the following SMQ search will be considered as AESI.

The counts and percentages of participants with acute renal events may be summarized by treatment if overall count >10 by using the MedDRA PTs contained in any of the following SMQs:

- Acute renal failure: Narrow terms in Acute renal failure SMQ (20000003) and
- Chronic kidney disease: Narrow terms in Chronic kidney disease SMQ (20000213).

In addition, a listing of participants with treatment-emergent acute renal events may be provided, if deemed necessary.

6.14.3.9.2. Dehydration

Dehydration events will be captured in the Narrow terms in Dehydration SMQ (20000232). Severe/serious dehydration events will be considered as AESI.

A listing of participants with treatment-emergent dehydration events may be provided.

6.14.3.10. Thyroid Safety Monitoring

6.14.3.10.1. Calcitonin

The purpose of calcitonin measurements is to assess the potential of tirzepatide to affect thyroid C-cell function, which may indicate development of C-cell hyperplasia and neoplasms.

Observed calcitonin data (a thyroid-specific laboratory assessment) will be summarized by treatment and nominal visit.

The counts and percentages of participants with a maximum postbaseline calcitonin value in the following thresholds will be provided by treatment and baseline calcitonin value (≤20 ng/L, >20 ng/L to ≤35 ng/L, >35 ng/L). Postbaseline categories are: ≤20 ng/L, >20 ng/L to ≤35 ng/L, >35 ng/L to ≤50 ng/L, >50 ng/L to ≤100 ng/L, and >100 ng/L.

6.14.3.10.2. C-Cell Hyperplasia and Thyroid Malignancies

Treatment-emergent thyroid malignancies and C-cell hyperplasia will be considered as AESI. Thyroid malignancies and C-cell hyperplasia will be identified using MedDRA High Level Term (HLT) for thyroid neoplasms and PT for thyroid C-cell hyperplasia.

The counts and percentages of participants with treatment-emergent thyroid C-cell hyperplasia and malignancies may be summarized or a listing of participants with treatment-emergent thyroid C-cell hyperplasia and neoplasms may be provided if deemed necessary.

6.14.3.11. Treatment-Emergent Supraventricular Arrhythmias and Cardiac Conduction Disorders

Severe/serious treatment-emergent supraventricular arrhythmias and cardiac conduction disorders will be considered as AESI. The cardiovascular events will include clinically relevant rhythm and conduction disorders.

The treatment-emergent supraventricular arrhythmias and cardiac conduction disorders events will be identified using the MedDRA PTs. Detailed searching criteria can be found in Appendix 2.

The counts and percentages of participants with treatment emergent supraventricular arrhythmias and cardiac conduction disorders may be summarized by treatment and PT nested within SMQ. The PT will be ordered with decreasing frequency in TZP arm within SMQ. A listing of participants with treatment-emergent supraventricular arrhythmias and cardiac conduction disorders may be provided if deemed necessary.

6.14.4. Vital Signs

Two sitting blood pressure and apical heart rate measurements are collected at each visit scheduled for vital sign collection. For the multiple records of an individual vital sign collected at the same visit, the average value will be used for data summaries and analyses. The vital signs collected in association with 6-minute walk test (6MWT) will be excluded from the vital signs analyses.

Descriptive summaries by treatment and by nominal scheduled visit will be provided for baseline and postbaseline values as well as change from baseline values.

An MMRM and/or an ANCOVA model as described in Section 6.14 might be conducted if necessary.

Counts and percentages of participants with treatment-emergent abnormal sitting SBP, sitting DBP, and heart rate will be presented by treatment for participants who have both baseline and at least 1 postbaseline result. A treatment-emergent high result is defined as a change from a value

less than or equal to the high limit at baseline to a value greater than the high limit at any time that meets the specified change criteria during the postbaseline period. A treatment-emergent low result is defined as a change from a value greater than or equal to the low limit at baseline to a value less than the low limit at any time that meets the specified change criteria during the postbaseline period. To assess decreases, change from the minimum value during the baseline period to the minimum value during the postbaseline period will be used. To assess increases, changes from the maximum value during the baseline period to the maximum value during the postbaseline period will be used. Both planned and unplanned measurements will be included in the analysis. The criteria for identifying participants with treatment-emergent vital signs abnormalities are listed in Table GPID.6.8.

Table GPID.6.8. Categorical Criteria for Abnormal Treatment-Emergent Blood Pressure and Heart Rate Measurements

Parameter	Low	High
Systolic BP (mm Hg) (Supine or sitting – forearm at heart level)	≤90 and decrease from baseline ≥20	≥140 and increase from baseline ≥20
Diastolic BP (mm Hg) (Supine or sitting – forearm at heart level)	≤50 and decrease from baseline ≥10	≥90 and increase from baseline ≥10
heart rate (bpm) (apical)	<50 and decrease from baseline ≥15	>100 and increase from baseline ≥15

Abbreviations: BP = blood pressure; bpm = beats per minute.

6.14.5. Clinical Laboratory Evaluation

All safety laboratory data will be reported in the International System of Units and conventional units. Limits from the performing lab will be used to define low (L) and high (H). Descriptive summaries by treatment and by nominal visit will be provided for the baseline and postbaseline values as well as the change from baseline values.

For selected laboratory analyte measurements collected quantitatively, observed and change from baseline values for each visit may be displayed in plots for participants who have both a baseline and at least 1 postbaseline planned measurement. Baseline will be the last nonmissing observation during baseline period. Unplanned measurements will be excluded from plots.

A shift table will be provided including unplanned measurements. The shift table will include the number and percentage of participants within each baseline category (low, normal, high, or missing) versus each postbaseline category (low, normal, high, or missing) by treatment. The proportion of participants shifted will be compared between treatments using Fisher's exact test.

For qualitative laboratory analytes, the number and percentage of participants with normal and abnormal values will be summarized by treatment.

A listing of abnormal findings will be created for laboratory analyte measurements, including qualitative measures. The listing will include participant identification, treatment group,

laboratory collection date, study day, analyte name, and analyte finding. Other variables may be added, as appropriate.

The MMRM model or ANCOVA (if MMRM model is not applicable) as described in Section 6.14 will be used for the analysis for the continuous measurements for selected lab tests with or without log-transformed (postbaseline measure/baseline measure) response variables. For measures analyzed using log-transformed values, the results will be presented with the scale back transforming to the original, related scale.

The summary of treatment-emergent abnormal, high, or low laboratory results any time will be provided.

6.15. Subgroup Analyses

The following subgroup variables will be considered for subgroup analyses if there are adequate number of patients in each subcategory:

- age group: ≤65, >65
- age group: ≤75, >75
- race: White, Black, Asian, Other
- sex: Male, Female
- ethnicity
- region: US, OUS
- baseline BMI (kg/m^2): <35, ≥35 and <40, ≥40 kg/m^2
- HF decompensation within 12 months of screening: Yes, No
- diagnosed T2DM at screening: Yes, No
- atrial fibrillation (AF) at baseline
- baseline eGFR (mL/min/1.73m2): <60, ≥60
- N-terminal pro-B-type natriuretic peptide (NT-proBNP) at baseline: ≤Median, >Median
- NYHA class: Class II, Class III or IV
- Baseline use of MRA: Yes, No
- Baseline use of ARNi: Yes, No
- Baseline use of SGLT2 inhibitors: Yes, No
- Baseline use of diuretics: Yes, No
- Baseline LVEF: ≤Median, >Median
- Baseline SBP: ≤Median, >Median

Subgroup analyses will be conducted for the primary efficacy endpoints. The primary analysis defined in Section 6.13.1.1.2 for the hierarchically composite endpoint and in Section 6.13.1.2.2 for the 6MWD endpoint will be conducted in each subcategory of the subgroup variable for the corresponding primary endpoint.

The subgroup analyses may also be performed for the key secondary efficacy endpoints using the primary analysis approaches defined in Section 6.13.2.

If the primary analysis approach is nonparametric (Section 6.13.2.1), the analysis will be conducted in each subcategory for the subgroup variable.

If the primary analysis approach is a longitudinal model, the same analysis model as defined in Section 6.13.2.2 or 6.13.2.3 for the corresponding endpoint will be conducted in each subcategory of the subgroup variable to obtain estimates of the treatment group difference. In addition, a full model with additional terms of subgroup, subgroup-by-treatment, subgroup-by-time, and subgroup-by-treatment-by-time interactions will be used to obtain interaction p-values.

6.16. Interim Analyses and Data Monitor Committee

The details for the interim analyses and data monitor committee (DMC) will be provided in the DMC charter.

7. Unblinding Plan

Details of the blinding and unblinding will be provided in Blinding and Unblinding Plan document for Study GPID.

8. References

[ADA] American Diabetes Association. 6. Glycemic targets: standards of medical care in diabetes-2019. Diabetes Care. 2019;42(suppl 1):S61-S70. https://doi.org/10.2337/dc19-S006

Finkelstein DM, Schoenfeld DA. Combining mortality and longitudinal measures in clinical trials. *Stat Med.* 1999;18:1341–1354. doi: 10.1002/(sici)1097-0258(19990615)18:11<1341::aid-sim129>3.0.co;2-7

Green CP, Porter CB, Bresnahan DR, Spertus JA. Development and evaluation of the Kansas City Cardiomyopathy Questionnaire: a new health status measure for heart failure. *J Am Coll Cardiol*. 2000;35(5):1245-1255. doi: 10.1016/s0735-1097(00)00531-3

Joseph SM, Novak E, Arnold SV, et al. Comparable performance of the Kansas City Cardiomyopathy Questionnaire in patients with heart failure with preserved and reduced ejection fraction. *Circ Heart Fail.* 2013;6(6):1139-1146. doi: 10.1161/CIRCHEARTFAILURE.113.000359

Pocock SJ, Ariti CA, Collier TJ, Wang D. The win ratio: a new approach to the analysis of composite endpoints in clinical trials based on clinical priorities. *Eur Heart J.* 2012;33(2):176-182. doi: 10.1093/eurheartj/ehr352

Rubin DB. *Multiple Imputation for Nonresponse in Surveys*. New York: John Wiley & Sons Inc.; 1987.

van Elteren, PH. On the combination of independent two sample tests of Wilcoxon. *Bull Inst Int Stats.* 1960;37 :351–361.

9. Appendices

Appendix 1. KCCQ – Scoring Instructions

The Kansas City Cardiomyopathy Questionnaire
Scoring Instructions

There are 10 summary scores within the KCCQ, which are calculated as follows:

1. Physical Limitation

- Code responses to each of Questions 1a-f as follows:

  Extremely limited = 1
  Quite a bit limited = 2
  Moderately limited = 3
  Slightly limited = 4
  Not at all limited = 5
  Limited for other reasons or did not do = <*missing value*>

- If at least three of Questions 1a-f are not missing, then compute

  Physical Limitation Score = 100*[(mean of Questions 1a-f actually answered) – 1]/4

  (see footnote at end of this document for explanation of meaning of "actually answered")

2. Symptom Stability

- Code the response to Question 2 as follows:

  Much worse = 1
  Slightly worse = 2
  Not changed = 3
  Slightly better = 4
  Much better = 5
  I've had no symptoms over the last 2 weeks = 3

- If Question 2 is not missing, then compute

  Symptom Stability Score = 100*[(Question 2) – 1]/4

3. Symptom Frequency

- Code responses to Questions 3, 5, 7 and 9 as follows:

  <u>Question 3</u>
  Every morning = 1
  3 or more times a week but not every day = 2
  1-2 times a week = 3
  Less than once a week = 4
  Never over the past 2 weeks = 5

3. Symptom Frequency (cont.)

Questions 5 and 7
All of the time = 1
Several times a day = 2
At least once a day = 3
3 or more times a week but not every day = 4
1-2 times a week = 5
Less than once a week = 6
Never over the past 2 weeks = 7

Question 9
Every night = 1
3 or more times a week but not every day = 2
1-2 times a week = 3
Less than once a week = 4
Never over the past 2 weeks = 5

- If at least two of Questions 3, 5, 7 and 9 are not missing, then compute:

S3 = [(Question 3) – 1]/4
S5 = [(Question 5) – 1]/6
S7 = [(Question 7) – 1]/6
S9 = [(Question 9) – 1]/4

Symptom Frequency Score = 100*(mean of S3, S5, S7 and S9)

4. Symptom Burden

- Code responses to each of Questions 4, 6 and 8 as follows:

Extremely bothersome = 1
Quite a bit bothersome = 2
Moderately bothersome = 3
Slightly bothersome = 4
Not at all bothersome = 5
I've had no swelling/fatigue/shortness of breath = 5

- If at least one of Questions 4, 6 and 8 is not missing, then compute

Symptom Burden Score = 100*[(mean of Questions 4, 6 and 8 actually answered) – 1]/4

5. Total Symptom Score

= mean of the following available summary scores:
Symptom Frequency Score
Symptom Burden Score

© Copyright 2000 – 2003 John Spertus MD MPH

6. Self-Efficacy

- Code responses to Questions 10 and 11 as follows:

  Question 10
  Not at all sure = 1
  Not very sure = 2
  Somewhat sure = 3
  Mostly sure = 4
  Completely sure = 5

  Question 11
  Do not understand at all = 1
  Do not understand very well = 2
  Somewhat understand = 3
  Mostly understand = 4
  Completely understand = 5

- If at least one of Questions 10 and 11 is not missing, then compute

  Self-Efficacy Score = 100*[(mean of Questions 10 and 11 actually answered) – 1]/4

7. Quality of Life

- Code responses to Questions 12, 13 and 14 as follows:

  Question 12
  It has extremely limited my enjoyment of life = 1
  It has limited my enjoyment of life quite a bit = 2
  It has moderately limited my enjoyment of life = 3
  It has slightly limited my enjoyment of life = 4
  It has not limited my enjoyment of life at all = 5

  Question 13
  Not at all satisfied = 1
  Mostly dissatisfied = 2
  Somewhat satisfied = 3
  Mostly satisfied = 4
  Completely satisfied = 5

  Question 14
  I felt that way all of the time = 1
  I felt that way most of the time = 2
  I occasionally felt that way = 3
  I rarely felt that way = 4
  I never felt that way = 5

© Copyright 2000 – 2003 John Spertus MD MPH

7. Quality of Life (cont.)

- If at least one of Questions 12, 13 and 14 is not missing, then compute

  Quality of Life Score = 100*[(mean of Questions 12, 13 and 14 actually answered) – 1]/4

8. Social Limitation

- Code responses to each of Questions 15a-d as follows:

  Severely limited = 1
  Limited quite a bit = 2
  Moderately limited = 3
  Slightly limited = 4
  Did not limit at all = 5
  Does not apply or did not do for other reasons = <*missing value*>

- If at least two of Questions 15a-d are not missing, then compute

  Social Limitation Score = 100*[(mean of Questions 15a-d actually answered) – 1]/4

9. Overall Summary Score

= mean of the following available summary scores:
Physical Limitation Score
Total Symptom Score
Quality of Life Score
Social Limitation Score

10. Clinical Summary Score

= mean of the following available summary scores:
Physical Limitation Score
Total Symptom Score

Note: references to "**means of questions actually answered**" imply the following.

- If there are *n* questions in a scale, and the subject must answer *m* to score the scale, but the subject answers only *n-i*, where *n-i* >= *m*, calculate the **mean of those questions** as
  (sum of the responses to those *n-i* questions) / (*n-i*)
  not
  (sum of the responses to those *n-i* questions) / *n*

If doing these calculations seems like too much trouble, consider using one of our tools – available at www.cvoutcomes.org:

- SAS or SPSS code
- Excel spreadsheets
- Web data services

© Copyright 2000 – 2003 John Spertus MD MPH

Appendix 2. Searching Criteria For Special Safety Topics

Pancreatitis Events

Determination of investigator-reported events will be through the "Acute pancreatitis" Standardized Medical Dictionary for Regulatory Activities (MedDRA) Query (SMQ) (20000022, narrow scope) and a "Chronic pancreatitis" Preferred Term (PT) search of the adverse event (AE) database, while adjudication-confirmed pancreatitis are found from adjudication forms.

Supraventricular Arrhythmias and Cardiac Conduction Disorders

Treatment-emergent supraventricular arrhythmias, arrhythmias and cardiac conduction disorders will be considered as an adverse event of special interest (AESI). The cardiovascular events will include clinically relevant rhythm and conduction disorders. The treatment-emergent supraventricular arrhythmias and cardiac conduction disorders events will be included using the MedDRA PT contained in any of the following SMQs:

1) Supraventricular Arrhythmias:
   - For symptoms: Arrhythmia related investigations, signs and symptoms SMQ (20000051), narrow and broad terms
   - For supraventricular arrhythmias: In Cardiac arrhythmia SMQ, under tachyarrhythmia sub SMQ
     - Supraventricular tachyarrhythmia SMQ (20000057), broad and narrow terms
     - Tachyarrhythmia terms, nonspecific SMQ (20000164), narrow terms only; and
     - Ventricular tachyarrhythmia SMQ (20000058), narrow terms only.
2) Cardiac Conduction Disorders
   - Conduction defects SMQ (20000056), narrow terms only; and
   - Cardiac conduction disorders High Level Term (HLT; 10000032), all PTs.

Injection Site Reactions

Treatment emergent injection site reaction will be summarized by treatment using the MedDRA PT in any of the following:

- HLT of Injection site reaction
- HLT of Administration site reaction
- HLT of Infusion Site Reactions

Acute Gallbladder Disease

All events of TEAE biliary colic, cholecystitis, or other suspected events related to gallbladder disease will be summarized by treatment groups by PT with decreasing frequency under following SMQs:

- Narrow PTs in Gallbladder related disorders SMQ (20000124)
- Narrow PTs in Biliary tract disorders SMQ (20000125), and
- Narrow PTs in Gallstone related disorders SMQ (20000127).

Hepatic Treatment-Emergent Adverse Events

Treatment-emergent potentially drug-related hepatic disorders will be summarized by treatment using the MedDRA PTs contained in any of the following SMQs:

- Broad and narrow terms in the Liver related investigations, signs and symptoms SMQ (20000008)
- Broad and narrow terms in the Cholestasis and jaundice of hepatic origin SMQ (20000009)
- Broad and narrow terms in the Hepatitis non-infections SMQ (20000010)
- Broad and narrow terms in the Hepatic failure, fibrosis and cirrhosis and other liver damage SMQ (20000013)
- Narrow terms in the Liver-related coagulation and bleeding disturbances SMQ (20000015)
- Narrow PTs in Gallbladder related disorders SMQ (20000124)
- Narrow PTs in Biliary tract disorders SMQ (20000125); and
- Narrow PTs in Gallstone related disorders SMQ (20000127).

Appendix 3. Cardiac Magnetic Resonance Imaging (MRI) Substudy

This section is applicable to the participants who are enrolled in the cardiac MRI addendum.

This addendum applies to a subset of participants (approximately 150 patients) enrolled at selected sites that have the technical capability of conducting cardiac MRI. MRI measurements at baseline will be performed at randomization or within 7 days after randomization (V2). The baseline MRI should be performed prior to the second dose of study treatment. The postbaseline MRI is collected at 52 weeks or at early discontinuation visit if the discontinuation occurs prior to 52 weeks.

MRI analyses will be guided by treatment policy strategy and conducted among all patients who are enrolled in the addendum, received at least 1 dose of study drug, and have at least 1 MRI measurement. The baseline MRI is defined as the measurement taken prior to the second dose of the study treatment. The measurement taken at early discontinuation visit will be carried forward and used as the measurement for Week 52. The patient demographics and baseline characteristics for the MRI substudy will be summarized.

Objectives	Endpoints	Analytical Approaches
Evaluation of cardiac function and structure by cardiac MRI	Changes from baseline to Week 52 for the following:<br>Structural and functional parameters<br>• Left ventricular mass and index (LVM and LVMI, respectively)<br>• Left ventricular end diastolic volume and index (LVEDV and LVEDVI, respectively)<br>• Left ventricular end systolic volume and index (LVESV and LVESVI, respectively)<br>• Left atrial volume and index (LAV and LAVI, respectively)<br>• Left ventricular ejection fraction (LVEF)<br>Feature tracking<br>• Left ventricular global longitudinal strain (LVGLS)<br>• Left ventricular global circumferential strain (LVGCS)<br>Adipose tissue volumes<br>• Epicardial fat volume<br>• Pericardial fat volume	Change from baseline to Week 52 for each parameter will be compared between treatment arms using an analysis of covariance (ANCOVA) approach. The model will include treatment, the stratification factors of diagnosed T2DM (Y/N) and baseline BMI $\geq$ 35 (Y/N), and the baseline value for the parameter.<br>Summary statistics for MRI parameters at baseline and at Week 52 will be provided. The treatment comparison at baseline will be performed using an ANOVA model.

Title Page

Protocol Title: A Randomized, Double-Blind, Placebo-Controlled, Phase 3 Study Comparing the Efficacy and Safety of Tirzepatide versus Placebo in Patients with Heart Failure with Preserved Ejection Fraction and Obesity (SUMMIT)

Protocol Number: I8F-MC-GPID

Compound Number: LY3298176

Short Title: A Statistical Analysis Plan for Tirzepatide in Participants with Heart Failure with Preserved Ejection Fraction and Obesity (SUMMIT)

Sponsor Name: Eli Lilly and Company

Legal Registered Address: Indianapolis, Indiana USA 46285

Regulatory Agency Identifier Number(s)

**Registry**	**ID**
IND	147352

Confidential Information

The information contained in this document is confidential and the information contained within it may not be reproduced or otherwise disseminated without the approval of Eli Lilly and Company or its subsidiaries

Note to Regulatory Authorities: This document may contain protected personal data and/or commercially confidential information exempt from public disclosure. Eli Lilly and Company requests consultation regarding release/redaction prior to any public release. In the United States, this document is subject to Freedom of Information Act (FOIA) Exemption 4 and may not be reproduced or otherwise disseminated without the written approval of Eli Lilly and Company or its subsidiaries.

Document ID: VV-CLIN-064990

Approved on 26 Jun 2024 GMT

Table of Contents

Title Page 1
Table of Contents 2
Version history 6
1. Study Objectives 11
1.1. Primary Objective 11
1.2. Key Secondary Objectives 11
2. Study Design 12
3. A Priori Statistical Methods 13
3.1. Populations for Analyses 13
3.2. General Considerations 13
3.3. Adjustments for Covariates 15
3.4. Handling of Dropouts or Missing Data 15
3.5. Multicenter Studies 15
3.6. Multiple Comparisons/Multiplicity 15
3.7. Patient Disposition 15
3.8. Historical Illnesses and Preexisting Conditions 15
3.9. Patient Characteristics 15
3.10. Concomitant Therapy 16
3.11. Treatment Exposure and Compliance 16
3.11.1. Study and Study Treatment Exposure 16
3.11.2. Compliance to Study Treatment 17
3.12. Important Protocol Deviations 17
3.13. Efficacy Analyses 18
3.13.1. Primary Endpoints/Estimands Analysis 18
3.13.2. Key Secondary Endpoints/Estimands 21
3.13.3. Type I Error Rate Control Strategy for Primary and Key Secondary Efficacy Analyses 23
3.13.4. Other Secondary 24
3.13.5. Exploratory Endpoints 26
3.14. Safety Analyses 28
3.14.1. Analysis of Adverse Events 28
3.14.2. Patient Narratives 30
3.14.3. Special Safety Topics 30
3.14.4. Vital Signs 44
3.14.5. Clinical Laboratory Evaluation 45
3.15. Subgroup Analyses 46

3.15.1. Primary Efficacy Endpoints 46
3.15.2. Safety in Special Groups and Situations 47
3.16. Interim Analyses and Data Monitoring Committee 48
4. Unblinding Plan 49
5. Supporting Documentation 50
5.1. Appendix 1: Kansas City Cardiomyopathy Questionnaire – Scoring Instructions 50
5.2. Appendix 2: Search Criteria for Special Safety Topics 61
5.3. Appendix 3: Cardiac Magnetic Resonance Imaging Substudy 64
5.4. Appendix 4: Statistical Analysis for China 65
6. References 66

Table of Contents

Table **Page**

Table GPID.3.1. Description of Analysis Populations....13

Table GPID.3.2. Baseline and Postbaseline Definitions for Safety Analyses....14

Table GPID.3.3. Definition of Time-to-Event for a Specific Event of Interest....14

Table GPID.3.4. Other Secondary....25

Table GPID.3.5. Exploratory Efficacy Endpoints and Analyses....27

Table GPID.3.6. Sample ADA Assay Results....36

Table GPID.3.7. Sample Clinical ADA Interpretation Results....36

Table GPID.3.8. *In Silico* Classification for Cross-Reactive NAb....37

Table GPID.3.9. Adverse Events for Analysis with Immunogenicity Results....39

Table GPID.3.10. Categorical Criteria for Abnormal Blood Pressure and Heart Rate Measurements....44

Table GPID.3.11. Categorical Criteria for Additional Analyses of Heart Rate....45

Table of Contents

Figure **Page**

Figure GPID.2.1. Illustration of study design for Clinical Protocol I8F-MC-GPID. 12
Figure GPID.3.1 Graphical testing scheme for Study GPID. 24
Figure GPID.3.2. Flowchart of immunogenicity multitiered testing approach. 35

Approved on 26 Jun 2024 GMT

Version history

This is the second version of the statistical analysis plan (SAP) for Study I8F-MC-GPID (GPID), which is based on Protocol (c) GPID, approved on 14 February 2024. SAP GPID version 1 was approved on 22 November 2021. See approval date for the current version of this SAP on Page 1.

Major Revision Summary for I8F-MC-GPID Statistical Analysis Plan Version 2

Section # and Name	Description of Change	Brief Rationale
Section 1.1 Primary Objective	Revised primary endpoints to<br>• change from baseline to Week 52 in the KCCQ-CSS, and<br>• occurrence of the composite endpoint of CV death and/or HF events over time.<br>Hierarchical composite assessed by win ratio moved to other secondary endpoint.<br>Change from baseline to Week 52 in 6MWD moved to Key Secondary endpoints.	To align with protocol (c).
Section 1.2 Key Secondary Objectives	Key secondary endpoints revised to<br>• change from baseline to Week 52 in 6MWD<br>• percent change from baseline to Week 52 in body weight, and<br>• change from baseline to Week 52 in hsCRP.<br>NYHA class, exercise capacity (6MWD at Week 24) moved from Key Secondary endpoints to Other Secondary endpoints.	To align with protocol (c)

Section # and Name	Description of Change	Brief Rationale
Section 3.2 General consideration	Change end of study participation to end of follow-up.<br><br>Add detail of time to event derivation.	To provide more details of the updated primary analysis and to align with the new primary endpoints.
Section 3.11.1 Study and Study Treatment Exposure	Included longer follow-up interval when summarizing duration.	To provide more details of exposure.
Section 3.11.2 Compliance to Study Treatment	Clarification of compliance criteria.	To align with protocol (c)
Section 3.13.1 Primary Endpoints/Estimands Analysis	Revised statistical methods related to primary endpoints.	To align with the revised primary endpoint and provide an appropriate analysis method for each endpoint/estimand.
Section 3.13.2 Key Secondary Endpoints/Estimands	Revised statistical methods related to key secondary endpoints.	To align with the revised primary endpoint and provide an appropriate analysis method for each endpoint/estimand.
Section 3.13.3 Type I Error Rate Control Strategy for Primary and Key Secondary Efficacy Analyses	Updated type I error rate control strategy	To provide details of the strategy.
Section 3.13.4 Other Secondary	Other Secondary endpoints added including:<br>• hierarchical composite assessed by win ratio<br>• clinical outcome events of HF<br>• NYHA Class<br>• proportion of participants attaining ≥5%, ≥10%, ≥15% and ≥20% in body weight reduction change at Week 52	To align with the revised primary endpoint and provide an appropriate analysis method for each endpoint/estimand.

Section # and Name	Description of Change	Brief Rationale
	- change from baseline to Week 24 in KCCQ-CSS added to other secondary endpoints - proportion of participants attaining KCCQ-CSS MWPC threshold at Week 52 added to other secondary endpoints - proportion of participants attaining ≥5 meters, ≥10 meters, and ≥15 points KCCQ-CSS change at Week 52 - change from baseline to Week 24 in 6MWD - proportion of participants attaining 6MWD meaningful within-patient change (MWPC) threshold at Week 52, and - proportion of participants attaining ≥10 meters, ≥20 meters, and ≥30 meters 6MWD change at Week 52.	
Section 3.13.5 Exploratory Endpoints	Exploratory endpoint "HF medication use" integrated into primary endpoint CV death and/or HF event.<br><br>Clinical outcome events of HF moved to other secondary.<br><br>Exploratory endpoint "Evaluation of prespecified	To align with the revised primary endpoint and provide an appropriate analysis method for each endpoint/estimand.

Approved on 26 Jun 2024 GMT

Section # and Name	Description of Change	Brief Rationale
	biomarkers" hsCRP; moved to key secondary endpoint.<br>Added exploratory endpoints:<br>• change from baseline to week 52 in waist to height ratio, and<br>• eGFR slope.	
Section 3.14.3 Special Safety Topics	Added safety topics.	To align with PSAP GPID version 4.
Section 3.14.3.5.2 Liver Enzymes	Updated categorization.	To align with the new Lilly hepatic analyses plan.
Section 3.15 Subgroup Analysis	Updated categorization of subgroup variables.<br>Removed subgroup of ARNi and age category of 75 and added new subgroups.<br>Added safety in special groups and situations section.	To be consistent with baseline characteristics and to provide additional subgroups analysis.<br>To align with PSAP GPID version 4 and provide submission level safety analyses details.
Section 5.1, Appendix 1	Added Kansas City Cardiomyopathy Questionnaire.	To provide details of the questionnaire.
Section 5.2, Appendix 2	Added further detailed search criteria for analysis of special safety topics.	To align with PSAP GPID version 4 and provide detailed descriptions of MedDRA search criteria.
Section 5.3, Appendix 3	Added additional endpoints of interest and analysis details.	To provide additional details of analysis for the cardiac MRI substudy.
Section 5.4, Appendix 4	Added statistical analysis for China.	To specify analyses to be performed for participants enrolled in mainland China and Taiwan.

Abbreviations: 6MWD = 6-minute walk test distance; ARNi = angiotensin receptor-neprilysin inhibitors; CV = cardiovascular; eGFR = estimated glomerular filtration rate; HF = heart failure; hsCRP = high-sensitivity C-reactive protein ; KCCQ-CSS = Kansas City Cardiomyopathy Questionnaire-Clinical Summary Score; MedDRA = Medical Dictionary for Regulatory Activities; MWPC = meaningful within-patient change; NYHA = New York Heart Association; PSAP = program statistical analysis plan.

1. Study Objectives

1.1. Primary Objective

To demonstrate that a maximally tolerated dose (MTD) of tirzepatide up to 15 mg administered subcutaneously (SC) once weekly (QW) is superior to placebo in participants with heart failure (HF) with preserved ejection fraction (HFpEF) and body mass index (BMI) ≥30 kg/m^2 based on:

- change from baseline in Kansas City Cardiomyopathy Questionnaire (KCCQ) Clinical Summary Score (CSS) at Week 52, and
- occurrence of the composite endpoint of cardiovascular (CV) death and/or heart failure (HF) events over time.

The effectiveness of tirzepatide will be demonstrated if either one or both the primary objectives is met. The details on type I error control are discussed in Section 3.13.3.

The HF event definition within Protocol GPID includes worsening symptoms or signs of HF, which are meaningful to the participant and require intensification of treatment characterized by one or more of the following: hospitalization for heart failure regardless of duration or treatment received; use of intravenous drug, usually an intravenous diuretic, but may include intravenous vasodilators or positive inotropic drugs; or augmentation or increase in oral diuretic therapy.

1.2. Key Secondary Objectives

The key secondary objectives are to demonstrate that tirzepatide MTD is superior to placebo with regards to

- change from baseline in 6-minute walk test distance (6MWD) at Week 52
- percent change from baseline in body weight at Week 52, and
- change from baseline in high-sensitivity C-reactive protein (hsCRP) at Week 52.

All the key secondary objectives are under multiplicity control. The details on type I error control are discussed in Section 3.13.3.

2. Study Design

Study GPID is a randomized, outpatient, multicenter, international, placebo-controlled, double-blinded, parallel arm Phase 3 study with 2 study periods. The study is designed to evaluate the efficacy and safety of QW tirzepatide MTD up to 15 mg compared to placebo in participants with HFpEF and BMI ≥30 kg/m^2. Figure GPID.2.1 illustrates the Study GPID design.

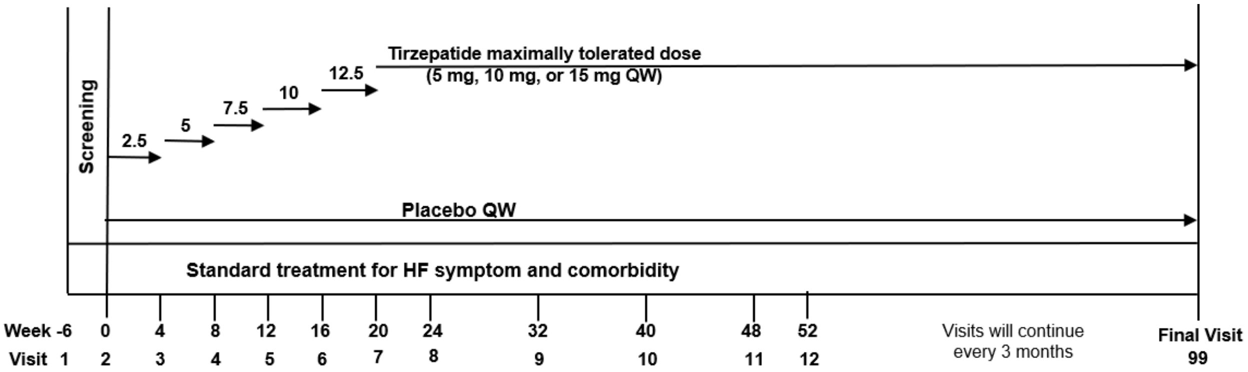

Note: Screening procedures may take longer or shorter than 6 weeks and variation in screening procedures will not be considered a protocol deviation.

Figure GPID.2.1. Illustration of study design for Clinical Protocol I8F-MC-GPID.

Two intervention groups will be studied:

- tirzepatide MTD up to 15 mg SC QW, and
- placebo.

Study GPID will compare treatment with tirzepatide and treatment with placebo. Assignment to tirzepatide or placebo groups will be randomly allocated in a 1:1 ratio.

The starting dose of tirzepatide is 2.5 mg QW, which will be escalated at 4-week intervals to a maximum of 15 mg QW, or to the highest maintenance dose tolerated by the participant.

The study will consist of 2 periods:

- Study Period 1: screening period, up to approximately 6 weeks, and
- Study Period 2: treatment period, with a 20-week escalation followed by at least a 32-week maintenance period.

Study GPID will continue until approximately 52 weeks after the last participant is randomized. The maximum duration of an individual’s participation will depend on the duration of study enrollment.

3. A Priori Statistical Methods

3.1. Populations for Analyses

The populations for analyses are defined in the following table (Table GPID.3.1).

Table GPID.3.1. Description of Analysis Populations

Analysis Population	Description
Entered	All participants who sign the ICF.
Randomized/ITT Population	All participants assigned to treatment, regardless of whether they take any doses of study treatment, or if they took the correct treatment. Participants will be analyzed according to the treatment group to which they were assigned.
Safety Population	All participants in the ITT population who take at least 1 dose of study treatment. Participants will be analyzed according to the treatment group to which they were assigned.

Abbreviations: ICF = informed consent form; ITT = intent-to-treat.

3.2. General Considerations

Statistical analysis of this study will be the responsibility of Eli Lilly and Company or its designee. Some analyses and summaries described in this analysis plan may not be conducted if not warranted by data (for example, too few events to justify conducting an analysis). Additional analyses of the data may be conducted as deemed appropriate.

Statistical treatment comparisons will be performed between tirzepatide MTD and placebo. Unless otherwise specified, all tests of treatment effects will be conducted at a 2-sided alpha level of 0.05, and all confidence intervals (CIs) will be given at a 2-sided 95% level. Efficacy will be assessed using the intent-to-treat (ITT) population. Baseline is defined as the last nonmissing observation collected prior to or at randomization for efficacy analyses. Safety will be assessed using safety population and the definition of baseline and postbaseline for safety analyses are specified in Table GPID.3.2.

Table GPID.3.2. Baseline and Postbaseline Definitions for Safety Analyses

Analysis Type	Baseline	Postbaseline
1.1) Treatment-Emergent Adverse Events	The baseline period is defined as the start of screening and ends prior to the first dose of study treatment (typically at Week 0).	Starts after the first dose of study treatment and ends at the end of follow-up. See Section 3.2 for the definition of a participant's end of follow-up.
1.2) Treatment-Emergent Abnormal Labs[a] and Vital Signs	Baseline will include all scheduled and unscheduled measurements during the baseline period (Visit 1 and Visit 2).	Postbaseline will be defined as measurements after Visit 2. All scheduled and unscheduled measurements will be included.
**Analysis Type**	**Baseline**	**Postbaseline**
1.3) Change from Last Baseline to Week xx and to Last Postbaseline for Labs[a] and Vital Signs	The last scheduled and unscheduled nonmissing assessment recorded during the baseline period defined above (1.2).	Postbaseline will be defined as above (1.2). Only scheduled visits will be included. The ET visits are considered scheduled visits.

Abbreviations: ET = early termination.

[a] Immunogenicity related analysis is specified in Section 3.14.3.8.

Summary descriptive statistics for continuous measures will include sample size, mean, standard deviation (SD), median, minimum, and maximum. Summary statistics for categorical measures (including categorized continuous measures) will include sample size, frequency, and percentages. Summary statistics for discrete count measures will include sample size, mean, SD, median, minimum, and maximum.

Unless otherwise specified, all observed data will be considered for analysis regardless of adherence to randomized treatment. The participant's end of follow-up will be the later of last contact date or study disposition date.

The definition of time-to-event for a specific event of interest is specified in Table GPID.3.3.

Table GPID.3.3. Definition of Time-to-Event for a Specific Event of Interest

If a participant:	then:
Experiences the event	time-to-event for a specific event of interest will be the number of days between the date of randomization and the onset date of the event plus 1 day.
Does not experience the event	the participant will be censored and the number of days between the date of randomization and the date of the participant's end of follow-up plus 1 day will be used for analysis.

Not all analyses described in SAP GPID will necessarily be included in the clinical study report (CSR). Any analysis described in this SAP and not provided in the CSR will be available upon request. Not all displays will necessarily be created as a "static" display. Some may be incorporated into interactive display tools instead of or in addition to a static display.

3.3. Adjustments for Covariates

The study is stratified by diagnosed type 2 diabetes mellitus (T2DM) (Y/N), HF decompensation (including hospitalization for HF requiring intravenous [IV] diuretic treatment or urgent HF visit requiring IV diuretic treatment) within 12 months of screening (Y/N), and baseline BMI group (<35, ≥35 kg/m^2). Unless otherwise specified, the stratification factors will be adjusted in the efficacy analyses. The value for stratification factors will be obtained from the data collected or derived from the electronic case report form (eCRF). In addition, the baseline value of the endpoint will be used as a covariate when appropriate.

3.4. Handling of Dropouts or Missing Data

For the primary and key secondary efficacy endpoint analyses subject to type I error rate control, missing data will be imputed based on the methods described in Section 3.13.1.3 and 3.13.2.

For all other endpoints, missing values will not be explicitly imputed unless specified otherwise.

3.5. Multicenter Studies

There is no stratification by site or country for randomization. However, the country or region effect may be examined for the primary endpoints through subgroup analysis.

3.6. Multiple Comparisons/Multiplicity

The type I error rate control strategy for primary and key secondary objectives is discussed in Section 3.13.3. There will be no multiplicity adjustments for evaluating other secondary and exploratory objectives and safety assessments.

3.7. Patient Disposition

Summaries and a listing of study disposition and study drug disposition will be provided for all randomized participants. Comparison between treatment arms will be performed using Fisher's exact test.

Summaries of study disposition will be provided for all entered but not randomized participants.

3.8. Historical Illnesses and Preexisting Conditions

The count and percentages of participants with historical illnesses and preexisting conditions will be summarized by treatment group using the Medical Dictionary for Regulatory Activities (MedDRA Version 27.0) preferred terms (PTs) nested within system organ class (SOC). The SOC will be in alphabetical order. Conditions (that is, PTs) will be ordered by decreasing frequency in tirzepatide MTD arm within SOC. This will be summarized for all randomized participants. Historical illnesses and preexisting conditions of special interest will also be summarized separately.

3.9. Patient Characteristics

A listing of participant demographics for all randomized participants will be provided. The demographic and baseline clinical characteristics will also be summarized by study treatment

group for all randomized participants. Baseline demographic and clinical characteristics of special interest include but are not limited to:

- age (years)
- sex (female, male)
- race
- ethnicity
- height (cm)
- weight (kg)
- BMI (kg/m^2)
- waist circumference (cm)
- age group (<65, ≥65; <75, ≥75; <65, ≥65 and <75, ≥75 and < 85, ≥85)
- BMI group (≥30 and <35, ≥35 and <40, ≥40 kg/ m^2)
- country
- vital signs, and
- characterization of HFpEF and HFpEF-related comorbidities.

3.10. Concomitant Therapy

Concomitant medication will be summarized by treatment groups and displayed by decreasing frequency of WHODrug (Version MAR24B3) PTs in tirzepatide MTD arm.

In addition, medications of interest (as defined below) will be summarized by treatment groups:

- Baseline use of
  - HF medications
  - antihypertensive therapy other than HF medications
  - antiplatelet and anticoagulant medications
  - antihyperglycemic medications, and
  - lipid lowering therapy.
- Changes to baseline medication post-randomization
  - HF medications
  - antihypertensive therapy other than HF medications
  - antihyperglycemic therapy, and
  - lipid lowering therapy.

3.11. Treatment Exposure and Compliance

3.11.1. Study and Study Treatment Exposure

A summary of duration on study follow-up (defined as time in days from date of randomization to the date of the end of follow-up plus 1 day) will be provided by treatment group in the ITT population.

A summary of duration on study treatment (defined as time in days from date of first dose of study treatment to date of last dose of study treatment plus 7 days) will be provided by treatment group in the safety population.

For the summary of duration on study and study treatment, the frequency and percentage of participants falling into the following categorical ranges will also be summarized by planned treatment group: >0 week, ≥4 weeks, ≥8 weeks, ≥12 weeks, ≥16 weeks, ≥20 weeks, ≥24 weeks, ≥32 weeks, ≥40 weeks, ≥48 weeks, ≥52 weeks, ≥65 weeks, ≥78 weeks, ≥91 weeks, ≥104 weeks, ≥117 weeks, ≥130 weeks, ≥143 weeks, ≥156 weeks.

In addition, the frequency and percentages of participants falling into the following exposure ranges for study and study treatment may be summarized by planned treatment group: >0 to <4 weeks, ≥4 to <8 weeks, ≥8 to <12 weeks, ≥12 to <16 weeks, ≥16 to <20 weeks, ≥20 to <24 weeks, ≥24 to <32 weeks, ≥32 to <40 weeks, ≥40 to <48 weeks, ≥48 to <52 weeks, ≥52 weeks to <65 weeks, ≥65 weeks to <78 weeks, ≥78 weeks to <91 weeks, ≥91 weeks to <104 weeks, ≥104 weeks to <117 weeks, ≥117 weeks to <130 weeks, ≥130 weeks to <143 weeks, ≥143 weeks to <156 weeks, ≥156 weeks.

No p-values will be reported in these summaries as they are intended to describe the study populations rather than test hypotheses.

3.11.2. Compliance to Study Treatment

A summary of prematurely discontinuing study treatment (including reason for discontinuation) will be provided by study treatment. A time-to-event analysis of premature study treatment discontinuation will also be conducted.

If the data warrants, the counts and percentages of participants who follow the planned escalation scheme, have dose interruption, or have dose modification, will be summarized for the tirzepatide-treated group. In addition, the proportion of participants receiving 2.5 mg, 5 mg, 7.5 mg, 10 mg, 12.5 mg, or 15 mg dose may be presented for the tirzepatide-treated group by visit during the dose escalation period.

Treatment compliance will be defined as taking at least 75% of the scheduled tirzepatide doses. Similarly, a participant will be considered significantly noncompliant if he or she is judged by the investigator to have intentionally or repeatedly taken more than the prescribed amount of medication (more than 125%).

Compliance over the whole treatment period will be calculated using the number of doses administered (regardless of the actual dose in mg administered) divided by the total number of doses expected to be administered ×100 over the treatment period. Total number of doses expected is defined as the number of weeks between the treatment end date and first dose date minus the number of dose interruptions due to adverse event (AE), investigator decision, or abnormal lab results if there are any. Treatment compliance will be summarized descriptively over the treatment period by treatment using the safety population.

3.12. Important Protocol Deviations

Important protocol deviations are defined in the Trial Issues Management Plan. A listing and a summary of important protocol deviations by treatment will be provided.

Approved on 26 Jun 2024 GMT

3.13. Efficacy Analyses

The primary estimand for primary endpoints and key secondary endpoints is to assess the treatment difference between tirzepatide and placebo relative to the efficacy measures for all randomized participants. The treatment policy strategy will be used to handle intercurrent events (ICEs), meaning all the observed values for the variable of interest are used regardless of whether or not the ICE occurs. The details of the primary estimand for each endpoint will be described in the following sections.

3.13.1. Primary Endpoints/Estimands Analysis

3.13.1.1. Change from Baseline in Kansas City Cardiomyopathy Questionnaire-Clinical Summary Score (KCCQ-CSS)

3.13.1.1.1. Estimand for the Endpoint

The estimand for the primary endpoint is described by the following attributes:

- Endpoint: change from baseline to Week 52 in KCCQ-CSS.
- Treatment condition of interest: tirzepatide MTD versus placebo.
- Handling of ICEs: using the treatment policy strategy to handle all ICEs.
- Population: all randomized participants.
- Population-level summary: the median difference between treatment arms.

The KCCQ is a 23-item, participant self-administered questionnaire that assesses impacts of HF “over the past 2 weeks” on 7 domains (Green et al. 2000; Joseph et al. 2013). Each of the 23 individual items are answered on Likert scales of varying length (5-point, 6-point, or 7-point scales). Domain scores are obtained by averaging the associated individual items and transforming the score to a 0 to 100 range. Higher scores indicate better health status. KCCQ-CSS is a summary score that is derived by taking the mean of the Physical Limitation and Total Symptom scores. Detailed scoring instructions are provided in Appendix 1, Section 5.1.

3.13.1.1.2. Main Analytical Approach

A stratified Wilcoxon test (van Elteren 1960) will be used as the main analysis method, controlling for the stratification factors of HF decompensation within 12 months of screening (Y/N), diagnosed T2DM (Y/N), and baseline BMI (<35, ≥35 kg/m^2). The Hodges-Lehmann estimate for the median difference and 2-sided 99% and 95% CIs will be reported.

Missing KCCQ-CSS measurements at Week 52 will be imputed through multiple imputation as specified in Section 3.13.1.3. The complete datasets generated through multiple imputation will be analyzed and a Van Elteren test will be conducted for treatment comparison. The final statistical inference over multiple imputation will be guided by the method proposed by Rubin (1987).

The empirical cumulative distribution function and histogram of the change from baseline to Week 52 in the KCCQ-CSS will be provided by treatment group.

3.13.1.2. Occurrence of Cardiovascular Death and/or Heart Failure Event over time

3.13.1.2.1. Time to first occurrence of Cardiovascular Death or Heart Failure Event

3.13.1.2.1.1. Estimand for the Endpoint

The estimand for the primary endpoint is described by the following attributes:

- Endpoint: time from randomization to the first occurrence of the clinical endpoint committee (CEC)-confirmed heart failure events or CV death.
- Treatment condition of interest: tirzepatide MTD versus placebo.
- Handling of ICEs: using treatment policy strategy to handle all ICEs.
- Population: all randomized participants.
- Population-level summary: hazard ratio.

3.13.1.2.1.2. Main Analytical Approach

The primary analysis will be a Cox proportional hazards model with treatment as a fixed effect adjusting for diagnosed T2DM (Y/N), baseline probability of HFpEF (<0.8, ≥0.8), and baseline N-terminal pro b-type natriuretic peptide (NTproBNP) (<200, ≥200 ng/L). The probability of HFpEF is derived from the HFpEF-ABA model (Reddy et al 2024). Participants who did not have an adjudicated primary endpoint event on or prior to the end of follow-up will be censored at the date of participant's end of follow-up. The missing data due to censoring will be implicitly handled by the Cox regression model, assuming censoring is independent of the outcome. The hazard ratio, with its CI and p-value, will be provided through the primary analysis model.

The Kaplan-Meier method will be used to estimate the cumulative event curve over time. Counts and proportions of participants who experience a primary endpoint event will be calculated. The total person-years of follow-up for the primary endpoint, the incidence rate per 100 person-years of follow-up for the primary endpoint, and the absolute risk difference (ARD) for the primary endpoint will be provided.

Person-years of follow-up for a specific event of interest are defined for each participant as the time-to-event divided by 365.25.

The incidence rate per 100 person-years of follow-up is defined by dividing the number of participants who developed the event during the study period by the event specific total person-years of follow-up (that is, time-to-event as defined above) multiplied by 100.

The ARD for an endpoint is defined as the difference in incidence rate per 100 person-years between the 2 treatment groups (placebo minus tirzepatide).

3.13.1.3. Methods for Missing Data Imputation

The missing measurement for KCCQ-CSS at 52 weeks for the primary estimand will be imputed through multiple imputation based on the reason for missingness.

- For missing measurements due to death, multiple imputation will be performed using the worst 15% observed data at 52 weeks from the same treatment group.
- For missing data due to all other ICEs or without ICE, retrieved dropout imputation will be applied, which will utilize observed data from participants in the same treatment group

who had outcome measures at Week 52 after early discontinuation of study drug to impute the missing value. In case there are not enough retrieved dropouts to provide a reliable imputation model, reference to the placebo imputation will be used.

3.13.1.4. Sensitivity Analyses for Kansas City Cardiomyopathy Questionnaire-Clinical Summary Score

For the primary endpoint of change from baseline in KCCQ-CSS, a mixed-effects model repeated measures (MMRM) analysis will be conducted to analyze the change from baseline in the KCCQ-CSS. The analysis will be guided by the treatment policy strategy. All values of the collected KCCQ-CSS data at baseline, 24 weeks, and 52 weeks will be used in the MMRM analysis. The primary endpoint assessment will be the contrast between tirzepatide and placebo at Week 52. The MMRM analysis will be repeated using data during the on-treatment period, which is considered as up to the last dose date plus 7 days.

The MMRM model will include treatment, time (Weeks 24 and 52), treatment-by-time interaction, stratification factors as fixed effects, and baseline value of the KCCQ-CSS as a covariate. Restricted maximum likelihood (REML) will be used to obtain model parameter estimates and the Kenward-Roger option will be used to estimate the denominator degrees of freedom. An unstructured covariance structure will be used to model the within-patient errors. If the analysis fails to converge, the following variance-covariance matrices will be used until convergence is achieved:

- heterogeneous Toeplitz
- heterogeneous first order autoregressive
- heterogeneous compound symmetry
- Toeplitz
- first order autoregressive, and
- compound symmetry.

In the MMRM analysis, the probability of missingness for any postbaseline KCCQ-CSS data that are not collected is assumed only to depend on the observed KCCQ-CSS values.

Change from baseline in the KCCQ-CSS will also be analyzed using an ANCOVA model. The ANCOVA model will include the categorical effect of treatment, stratification factors, and the continuous covariate of baseline KCCQ-CSS value. Missing KCCQ-CSS measurements at Week 52 will be imputed through multiple imputations as specified in Section 3.13.1.3.

3.13.1.5. Additional Analyses for Composite Endpoint of CV Death And HF Event

The contribution of each component of the primary composite endpoint (HF events and CV death) to the overall treatment effect will be examined. Methods similar to those described for the primary analysis will be used to separately analyze the time from randomization to the first occurrence of each component of the primary composite endpoint. The hazard ratio, with its CI and p-value, will be provided.

The CEC-confirmed total number of HF events (first and recurrent) and CV death will be analyzed by the semi-parametric proportional rates model (abbreviated as the LWYY model)

(Lin et al 2000) with treatment as a fixed effect adjusting for diagnosed T2DM (Y/N), baseline probability of HFpEF (<0.8, ≥0.8), and baseline NTproBNP (<200, ≥200 ng/L). A 30-day spacing rule will be applied when consider recurrent events. If an HF event is followed by another within 30 days, these HF events will be considered as a single HF event and the onset date will be the onset of the first HF event. If an HF event is followed by CV death within 30 days, only the CV death will be considered and the onset date will be the onset of the CV death. If an oral diuretic intensification is within 30 days of the date of randomization, it will not be considered as an endpoint. Participants who did not have an adjudicated primary endpoint event on or prior to end of follow-up will be censored at the date of participant's end of follow-up. The rate ratio with 95 confidence interval and p-value will be provided.

Non-parametric estimates of HF event rates over time, allowing for death as terminal event, will be provided (Ghosh and Lin 2000) with treatment as a fixed effect adjusting for diagnosed T2DM (Y/N), baseline probability of HFpEF (<0.8, ≥0.8), and baseline NTproBNP (<200, ≥200 ng/L). A similar spacing rule, as specified in Section 3.13.1.5, will be applied.

Analyses described in Section 3.13.1.2.1.2 for HF events and CV death will be repeated using the investigator's reported events.

3.13.2. Key Secondary Endpoints/Estimands

3.13.2.1. Change from Baseline in 6MWD at Week 52

3.13.2.1.1. Estimand for the Endpoints

The estimand is described by the following attributes:

- Endpoints: Change from baseline to Week 52 in 6MWD
- Treatment condition of interest: tirzepatide MTD vs. placebo
- Handling of intercurrent events: using treatment policy strategy to handle all intercurrent events
- Population: all randomized participants, and
- Population-level summary: median difference between treatment arms.

3.13.2.1.2. Main Analytical Approach

For the key secondary efficacy endpoints of change from baseline in 6MWD at Week 52, the same nonparametric approach as described in Section 3.13.1.1.2, and the same missing data imputation as described in Section 3.13.1.3, will be utilized.

3.13.2.2. Percent Change from Baseline in Body Weight Loss at Week 52

3.13.2.2.1. Estimand for the Endpoint

The estimand is described by the following attributes:

- Endpoint: percent change from baseline to Week 52 in body weight
- Treatment condition of interest: tirzepatide MTD versus. placebo
- Handling of ICEs: using treatment policy strategy to handle all ICEs
- Population: all randomized participants, and

- Population-level summary: the difference in means of the percent change between treatment arms.

3.13.2.2.2. Main Analytical Approach

The percent change from baseline in body weight will be analyzed using an analysis of covariance (ANCOVA) analysis. The ANCOVA model will include the categorical effect of treatment, stratification factors excluding baseline BMI group (<35, ≥35 kg/m^2), and the continuous covariate of baseline body weight value.

Missing body weight data at the scheduled postbaseline visits will be imputed using the retrieved dropout approach (as described in Section 3.13.1.3) through multiple imputation. The final statistical inference over multiple imputations will be obtained using the method proposed by Rubin (1987).

3.13.2.3. Change from Baseline in High-sensitivity C-reactive Protein at Week 52

3.13.2.3.1. Estimand for the Endpoint

- Endpoints: change from baseline to Week 52 in hsCRP.
- Treatment condition of interest: tirzepatide MTD versus placebo.
- Handling of ICEs: using the treatment policy strategy to handle all ICEs other than death.
- Population: all randomized participants.
- Population-level summary: the difference in mean change between treatment arms.

3.13.2.3.2. Main Analytical Approach

Change from baseline in hsCRP will be analyzed using an ANCOVA model. The ANCOVA model will include the categorical effect of treatment, stratification factors, and the continuous covariate of baseline hsCRP value. The ANCOVA model will be based on the log-transformed values of hsCRP.

Missing hsCRP at the scheduled postbaseline visits will be imputed using the retrieved dropout approach (as described in Section 3.13.1.3) through multiple imputation. The final statistical inference over multiple imputations will be obtained using the method proposed by Rubin (1987).

3.13.2.4. Sensitivity Analyses for Key Secondary Endpoints

For the change from baseline in 6MWD, a similar MMRM analysis as described in Section 3.13.1.4 will be conducted using data during the on-treatment period.

For percent change from baseline in body weight, a similar MMRM analysis as described in Section 3.13.1.4 will be conducted using data during the on-treatment period.

For the hsCRP change, a similar MMRM analysis as described in Section 3.13.1.4 will be conducted using data during the on-treatment period.

Change from baseline in the 6MWD will also be analyzed using an ANCOVA model. The ANCOVA model will include the categorical effect of treatment, stratification factors, and the

continuous covariate of the baseline 6MWD value. Missing 6MWD measurements at Week 52 will be imputed through multiple imputation as specified in Section 3.13.1.3.

3.13.3. Type I Error Rate Control Strategy for Primary and Key Secondary Efficacy Analyses

All primary and key secondary hypotheses will be tested with the overall family-wise type I error rate at a 2-sided alpha level of 0.05 through the multiplicity control approach based on the graphical multiple testing procedure. For the primary hypotheses, the HF outcome will be tested at a 2-sided alpha level of 0.04 and change in KCCQ-CSS will be tested at a 2-sided alpha level of 0.01 in parallel for statistical significance. If significant, the respective alpha of the primary endpoints will be propagated to test the key secondary endpoints. If any of the primary endpoints is not significant, then the appropriate alpha after the key secondary endpoints testing will be recycled to that primary endpoint.

Figure GPID.3.1 provides the details of the graphical multiple testing procedure.

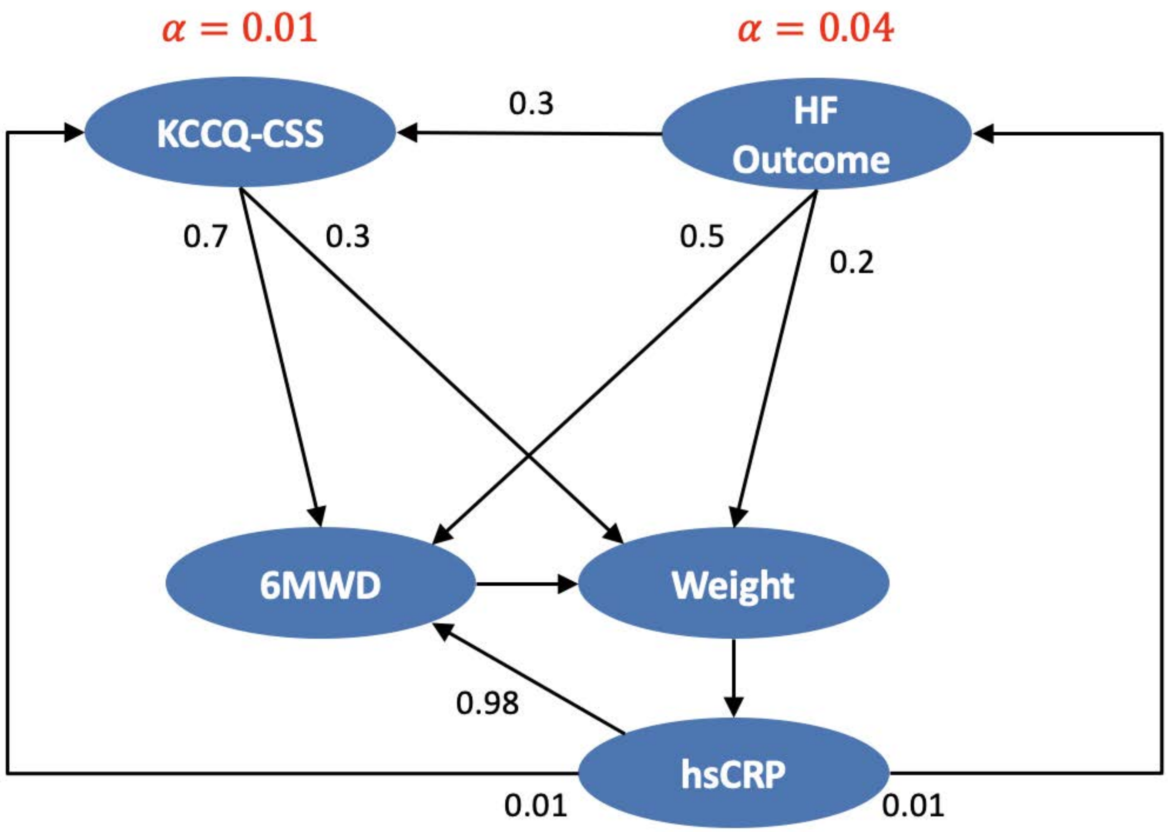

Abbreviations: 6MWD = 6-minute walk distance; HF = heart failure ; hsCRP = high-sensitivity C-reactive protein; KCCQ-CSS = Kansas City Cardiomyopathy Questionnaire Clinical Summary Score.

Figure GPID.3.1 Graphical testing scheme for Study GPID.

3.13.4. Other Secondary

Unless otherwise specified, other secondary analyses will be guided by the treatment policy strategy using all randomized population. Missing data will not be imputed unless otherwise specified. The other secondary analyses can be seen in Table GPID.3.4.

Table GPID.3.4. Other Secondary

Objectives	Endpoints	Analytical Approaches
Hierarchical composite	A hierarchical composite of the following:<br>• time to all-cause mortality through the end of follow-up<br>• occurrence of HF events through end of follow-up, where HF events are as defined in Section 1.1<br>• number of HF events<br>• time to first HF events<br>• change from baseline in KCCQ-CSS category at Week 52, and<br>• Change from baseline in the 6MWD category at Week 52.<br>The categories for change from baseline in the KCCQ-CSS are:<br>1. ≥10-point worsening<br>2. ≥5- but <10-point worsening<br>3. No change (<5-point change)<br>4. ≥5- but <10-point improvement<br>5. ≥10- but <15-point improvement, and<br>6. ≥15-point improvement.<br>The categories for change from baseline in the 6MWD are:<br>1. ≥30% worsening<br>2. ≥20% and <30% worsening<br>3. ≥10% and <20% worsening<br>4. no change (<10% change)<br>5. ≥10% and <20% improvement<br>6. ≥20% and <30% improvement, and<br>7. ≥30% improvement.	The win ratio (Pocock et al. 2012) will be reported as the measure of treatment effect based on the principle that each participant is compared with every other participant within each stratum in a pair-wise manner that proceeds in a hierarchical fashion.<br>Participants will be stratified according to HF decompensation within 12 months of screening (Y/N), diagnosed T2DM (Y/N), and baseline BMI group (<35, ≥35 kg/m^2), yielding 8 stratification pools. The variance of win ratio will be calculated by the asymptotic normal U statistic approach (Dong et al. 2018).<br>Missing KCCQ-CSS and 6MWD will be imputed as described in Section 3.13.1.3.
Clinical outcome events of HF	• Time to all-cause death<br>• Time to first occurrence of HF events or all-cause death<br>• Time to recurrent events of HF events and all-cause death<br>• Time to first occurrence of HF events<br>• Time to recurrent events of HF events	The time from randomization to the first occurrence of any component of the composite endpoint will be analyzed by a Cox proportional hazards model similar to the model described in Section 3.13.1.2.1.2.<br><br>Time to recurrent event analyses will be performed using a LWYY as specified in Section 3.13.1.5. Similar 30-day spacing rule will be applied as described in Section 3.13.1.5.

Objectives	Endpoints	Analytical Approaches
NYHA Class	Proportion of participants with NYHA Class improvement at Week 52.	Logistic regression analysis will be conducted including treatment, stratification factors, and baseline NYHA Class in the model.
Weight loss	Proportion of participants attaining ≥5%, ≥10%, ≥15% and ≥20% in body weight reduction change at Week 52.	Logistic regression analysis will be conducted including treatment, stratification factors excluding baseline BMI group (<35, ≥35 kg/m^2), and the continuous covariate of baseline body weight value in the model. Missing body weight measurements at Week 52 will be imputed using a retrieved dropout approach (as described in Section 3.13.2.2) through multiple imputation.
Exercise capacity	• Change from baseline to Week 24 in 6MWD<br>• Proportion of participants attaining 6MWD MWPC threshold at Week 52<br>• Proportion of participants attaining ≥10 meters, ≥20 meters, and ≥30 meters 6MWD change at Week 52	A similar nonparametric approach to the one described in Section 3.13.1.1.2 will be conducted.<br><br>Logistic regression analysis will be conducted including treatment, stratification factors, and baseline 6MWD in the model. The MWPC threshold at Week 52 is decided in analysis conducted separately guided by the PAP. Missing 6MWD measurements at Week 52 will be imputed through multiple imputation, as specified in Section 3.13.1.3.
Patient-reported symptoms and physical limitations	• Change from baseline to Week 24 in KCCQ-CSS<br>• Proportion of participants attaining KCCQ-CSS MWPC threshold at Week 52<br>• Proportion of participants attaining ≥5 meters, ≥10 meters, and ≥15 points KCCQ-CSS change at Week 52	A similar nonparametric approach to the one described in Section 3.13.1.1.2 will be conducted.<br><br>Logistic regression analysis will be conducted including treatment, stratification factors, and baseline 6MWD in the model. The MWPC threshold at Week 52 is decided in analysis conducted separately guided by the PAP. Missing KCCQ-CSS measurements at Week 52 will be imputed through multiple imputation as specified in Section 3.13.1.3.

Abbreviations: 6MWD = 6-minute walk test distance; BMI = body mass index; HF = heart failure; KCCQ-CSS = Kansas City Cardiomyopathy Questionnaire – Clinical Summary Score; LWYY = Lin-Wei-Yang-Ying; MWPC = meaningful within-patient change; NYHA = New York Heart Association; PAP = psychometric analysis plan; T2DM = type 2 diabetes mellitus.

Note: MWPC threshold at Week 52 for KCCQ-CSS is 20; MWPC threshold at Week 52 for 6MWD is 25 meters.

3.13.5. Exploratory Endpoints

Unless otherwise specified, exploratory efficacy analyses will be guided by treatment policy strategy using all randomized population. Missing data will not be imputed.

Table GPID.3.5. Exploratory Efficacy Endpoints and Analyses

Objectives	Endpoints	Analytical Approaches
Atrial fibrillation	• Proportion of participants with atrial fibrillation at Week 24 and at Week 52.<br>• Proportion of participants with atrial fibrillation at Week 24 and at Week 52 among those without atrial fibrillation at baseline.<br>• Proportion of participants without atrial fibrillation at Week 24 and at Week 52 among those with atrial fibrillation at baseline.	Fisher's exact test or logistic regression analysis including treatment, stratification factors, and baseline atrial fibrillation status will be conducted.
Waist circumference	Change from baseline in waist circumference	MMRM analysis similar to the model described in Section 3.13.1.4 will be conducted.
Patient-reported health-related quality of life	Change from baseline in KCCQ:<br>• Total Symptom Score (TSS)<br>• Overall Summary Score (OSS)	MMRM analysis similar to the model described in Section 3.13.1.4 will be conducted.
Patient-reported health status	Change from baseline in EQ-5D-5L (measured at 24 weeks and 52 weeks):<br>• Index Score, and<br>• VAS Score.	MMRM analysis similar to the model described in Section 3.13.1.4 will be conducted.
PGIS	Proportion of participants with improvements from baseline in:<br>• PGIS – Overall<br>• PGIS – Physical Function, and<br>• PGIS – Symptom Severity.	Proportion of participants with improvements from baseline will be summarized. Shift analysis from baseline to Week 24 and to Week 52 will also be performed.
Evaluation of prespecified biomarkers	Change from baseline in:<br>• NT-proBNP<br>• cTNT	MMRM analysis similar to the model described in Section 3.13.1.4 will be conducted. The data will be log-transformed for the analysis.
Waist to height ratio	Change from baseline to Week 52 in waist to height ratio	MMRM analysis similar to the model described in Section 3.13.1.4 will be conducted.
Kidney function	eGFR slope	The slope analysis will be constructed with eGFR as a dependent variable, including treatment group, stratification factors, baseline eGFR, time (as a continuous variable) and treatment-by-time interaction as fixed effects, and including subject as a random intercept and time as a random slope

Abbreviations: cTNT = cardiac troponin T; eGFR = estimated glomerular filtration rate; EQ-5D-5L = EuroQOL 5 Dimension 5 Level scale; KCCQ = Kansas City Cardiomyopathy Questionnaire; MMRM = mixed-effects model repeated measures; NT-proBNP = N-terminal pro-B-type natriuretic peptide; PGIS = Patient Global Impression of Severity; VAS = visual analog scale.
Note: Additional exploratory analysis may be conducted.

3.14. Safety Analyses

Unless specified otherwise, safety analyses will be conducted in the safety population (Table GPID.3.1). All events that occur between the first dose date of study drug and the end date of follow-up will be included in the safety analyses, regardless of the adherence to study drug.

The statistical assessment of homogeneity of the distribution of categorical safety responses between tirzepatide MTD and placebo will be conducted using Fisher's exact test, unless specified otherwise. Risk difference and its 95% CI will also be provided, where appropriate.

The mean change from baseline differences among treatments at all scheduled visits will be assessed via an MMRM analyses using REML. Unless specified otherwise, the MMRM analysis will contain measurements from planned visits up to 104 weeks, and the model will include treatment group, stratification factors, visit and treatment-by-visit interaction as fixed effects, and baseline value of the safety parameter as a covariate. To model the covariance structure within participants, the unstructured covariance matrix will be used. If this model fails to converge, the covariance structures specified in Section 3.13.1.4 will be tested in order until convergence has been met. If the data does not warrant the MMRM model, then an ANCOVA model will be conducted.

For selected safety parameters, a time-to-first-event analysis via the Cox-proportional hazards model may be conducted. Participants without the event will be censored at the end of follow-up.

3.14.1. Analysis of Adverse Events

3.14.1.1. Treatment-Emergent Adverse Events

A treatment-emergent AE (TEAE) is defined as an event that first occurred or worsened in severity after the first dose of study treatment. The MedDRA lowest level term (LLT) will be used in the treatment-emergent derivation. The maximum severity for each LLT during the baseline period, including ongoing medical history, will be used as baseline severity. For events with a missing severity during the baseline period, the event will be treated as mild in severity for determining treatment-emergence. Events with a missing severity during the postbaseline period will be treated as severe and treatment-emergence will be determined by comparing to baseline severity.

For events occurring on the day of taking study medication for the first time, the case report form (CRF)-collected information (for example, treatment emergent flag, start time of study treatment, and event) will be used to determine whether the event was pre- versus posttreatment if available. If the relevant information is not available, then the events will be counted as posttreatment.

Approved on 26 Jun 2024 GMT

Unless otherwise specified, the counts and percentages of participants with TEAEs will be summarized by treatment using the MedDRA PT nested within SOC. Statistical comparisons will be applied at both the SOC and PT levels. Events will be ordered by decreasing frequency in tirzepatide arm within SOC. The SOC will be in alphabetical order. For events that are sex-specific, the denominator and computation of the percentage will include only participants from the given sex.

An overview of the number and percentage of participants who experienced a TEAE, serious AE (SAE), death, or discontinued from study treatment or study due to an AE will be summarized by treatment.

The counts and percentages of patients with TEAEs by maximum severity will be summarized by treatment using MedDRA PT within SOC. For each participant and TEAE, the maximum severity for the MedDRA PT is the maximum postbaseline severity observed from all associated LLTs mapping to the MedDRA PT. The maximum severity will be determined based on the nonmissing severities. If all severities are missing for the defined postbaseline period of interest, it will show as missing in the table.

3.14.1.2. Common Adverse Events

The counts and percentages of participants with TEAEs, overall and common (common TEAEs occurred in ≥5% of participants before rounding), will be summarized by treatment using MedDRA PT. Events will be ordered by decreasing frequency in the tirzepatide arm.

3.14.1.3. Deaths

A listing of all deaths during Study GPID will be provided. The listing will include participant identification including the treatment, site number, date of death, age at the time of enrollment, sex, associated AE group identification, time from last dose of study drug to death (if participant had discontinued study drug), and primary cause of death.

3.14.1.4. Other Serious Adverse Events

The counts and percentages of participants who experienced an SAE (including deaths and SAEs temporally associated or preceding deaths) during the postbaseline period will be summarized by treatment using the MedDRA PT nested within SOC. Events will be ordered by decreasing frequency in the tirzepatide arm within SOC. The SOC will be in alphabetical order.

A listing of all SAEs will be provided. The listing will include treatment, participant identification including the site number, date of event, age at the time of enrollment, sex, AE group identification, MedDRA SOC and PT, severity, outcome, relationship to study drug, time from first dose of study drug to the event, and time from most recent dose to event (if the participant discontinued study drug prior to the event).

3.14.1.5. Other Significant Adverse Events

The counts and percentages of participants who discontinued from study treatment or study due to an AE during the postbaseline period may be summarized by treatment group using the MedDRA PT nested within the SOC. Events will be ordered by decreasing frequency in the

tirzepatide arm within SOC. Additionally, a Kaplan-Meier plot of time to study treatment discontinuation due to AEs will be presented.

3.14.2. Patient Narratives

Patient narratives will be provided for all participants who experience any of the following “notable” events:

- death
- protocol defined serious adverse event,
- pregnancy, or
- permanent discontinuation of study treatment due to AEs.

Patient narratives (patient level data and summary paragraph) will be provided for participants in the randomized population with at least 1 notable event.

3.14.3. Special Safety Topics

For AE(s) of special interest (AESI) or special safety topics, the counts and percentages of participants will be summarized by treatment and PT with decreasing frequency in the tirzepatide arm. Individual participant-level data may be presented. Displays with individual participant level data may be created using various formats, such as a customized listing and/or a customized graphical participant profile. AESI are defined in each section of the special safety topics, where applicable.

3.14.3.1. Amputation/Peripheral Revascularization

Amputation/peripheral revascularization will be considered as AESI. Participants with amputations/peripheral revascularization will be searched using the following MedDRA PTs and summarized:

- *Amputation*, and
- *Peripheral revascularization*.

A listing of participants with treatment-emergent (TE) events will be provided.

3.14.3.2. Diabetic Retinopathy Complications

Results of the baseline dilated fundoscopic exam will be summarized by treatment. Any TEAE suspected of worsening retinopathy triggers a follow-up dilated fundoscopic exam. A summary of TEAEs suspected of worsening retinopathy will be summarized by treatment and PT. Severe or serious AEs will be classified as AESI and a listing provided.

A complete list of PTs for inclusion in the above analyses is available in Appendix 2, Section 5.2.

3.14.3.3. Exocrine Pancreas Safety

3.14.3.3.1. Pancreatic Enzyme

Observed pancreatic enzyme data (p-amylase and lipase) will be summarized by treatment and nominal visit.

The counts and percentages of participants with maximum postbaseline pancreatic enzyme value exceeding the following thresholds will be provided by baseline pancreatic enzyme value (≤ upper limit of normal [ULN], > ULN), and postbaseline: ≤1 × ULN, (>1 to ≤3) × ULN, (> 3 to ≤5) × ULN, (>5 to ≤10) × ULN, >10 × ULN.

An MMRM analysis will be used to analyze each pancreatic enzyme with a log-transformed (postbaseline measure/baseline measure) response variable and treatment, nominal visit, and treatment-by-nominal visit interaction as fixed effects.

3.14.3.3.2. Pancreatitis Events

Investigator-reported events will be searched using the "*Acute pancreatitis*" Standardized MedDRA Query (SMQ) (20000022, narrow scope) and a "*Chronic pancreatitis*" PT in the AE database, while adjudication-confirmed pancreatitis are found from adjudication forms.

A summary of adjudicated pancreatic events will be provided by treatment.

TE adjudication confirmed pancreatitis will be considered as AESI. A listing of participants with investigator-reported and adjudicated pancreatitis will be provided.

3.14.3.4. Gastrointestinal Adverse Events

3.14.3.4.1. Nausea, Diarrhea, Constipation, and Vomiting

Summaries and analyses for incidence and severity of nausea, diarrhea (including "diarrhea", "diarrhoea" and "frequent bowel movements"), constipation (including "constipation", "infrequent bowel movement" and "faeces hard"), and vomiting (including "vomiting" and "vomiting projectile"), and nausea, vomiting and diarrhea combined, will be provided by each treatment group.

Summary of the prevalence over time for nausea, diarrhea, constipation, and vomiting will also be presented. Time to onset of nausea, diarrhea, constipation, and vomiting will be plotted.

3.14.3.4.2. Severe Gastrointestinal Events

The PTs under the *Gastrointestinal (GI) disorders* SOC in MedDRA will be used to identify GI AEs, and only PTs with serious/severe TE cases will be considered as AESI.

The counts and percentages of participants with severe/serious TE GI events will be summarized by treatment.

3.14.3.5. Hepatobiliary Disorders

3.14.3.5.1. Hepatobiliary Events

The counts and percentages of participants with TE hepatic events will be summarized by treatment using the MedDRA PTs. The detailed search criteria can be found in Appendix 2, Section 5.2.

TE events related to acute gallbladder disease will also be summarized. The search criteria can be found in Appendix 2, Section 5.2.

Approved on 26 Jun 2024 GMT

Severe/serious TE hepatic events and severe/serious TE acute gallbladder disease will be considered as AESI and summarized separately.

In addition, counts and percentages of participants with acute gallbladder disease by weight change category will be provided by treatment.

3.14.3.5.2. Liver Enzymes

Common analyses for laboratory analyte measurements described in Section 3.14.1.5 are applicable for the liver enzyme related measurements. This section provides additional analyses for liver enzymes.

The counts and percentages of participants with the following elevations in hepatic laboratory tests at any time during the study will be summarized between treatment groups:

- The counts and percentages of participants with an alanine transaminase (ALT) measurement ≥1× ULN, ≥3× ULN, ≥5× ULN, ≥10× ULN, and ≥20× ULN will be summarized for all participants with a postbaseline value and for subsets based on the following baseline values:
    - participants whose nonmissing maximum baseline value is ≤1× ULN
    - participants whose maximum baseline is >1× ULN, and
    - participants whose baseline values are missing.
- The counts and percentages of participants with an aspartate aminotransferase (AST) measurement ≥1× ULN, ≥3× ULN, ≥5× ULN, ≥10× ULN, and ≥20 × ULN during the treatment period will be summarized for all participants with a postbaseline value and for subsets based on various baseline levels, as described above for ALT.
- The counts and percentages of participants with a total bilirubin (TBL) measurement ≥2× ULN, ≥5× ULN, and ≥8× ULN will be summarized for all participants with a postbaseline value and for the following subsets based on the baseline values:
    - participants whose nonmissing maximum baseline value is ≤1× ULN
    - participants whose maximum baseline is >1 × ULN but <2 × ULN
    - participants whose maximum baseline value is ≥2× ULN, and
    - participants whose baseline values are missing.
- The counts and percentages of participants with a direct bilirubin (DBL) measurement ≥2× ULN AND ≥5× ULN will be summarized for all participants with a postbaseline value and for the same subsets as described for TBL.
- The counts and percentages of participants with a serum alkaline phosphatase (ALP) measurement ≥2× ULN and ≥3× ULN will be summarized for all participants with a postbaseline value and for the same subsets as described for TBL.
- The counts and percentages of participants with a gamma-glutamyltransferase (GGT) measurement ≥2× ULN will be summarized for all participants with a postbaseline value, if data available.

Maximum baseline will be the maximum nonmissing observation in the baseline period. The maximum value will be the maximum nonmissing value from the postbaseline period. Planned and unplanned measurements will be included.

Hepatocellular drug-induced liver injury screening plot will be created. The plot with maximum postbaseline transaminase (ALT or AST, whichever is higher, regardless of the time between the two maximum values) divided by ULN versus maximum postbaseline total bilirubin divided by ULN (y-axis) will be created that includes all participants from the safety populations. Each participant with at least 1 postbaseline ALT or AST and total bilirubin contributes 1 point to the plot. Dashed lines represent TBL and transaminase cut-offs of 2× ULN and 3× ULN, respectively. A potential Hy's law case is circled and is defined as having a maximum postbaseline TBL equal to or exceeding 2× ULN within 30 days after maximum postbaseline ALT or AST equal to or exceeding 3× ULN, without cholestasis (defined as ALP less than 2× ULN). The percentages of study participants falling in each of the three relevant quadrants of the plot (right upper, left upper, right lower) will be summarized in a table.

Cholestatic Drug-Induced Liver Injury Screening plot based on the maximum postbaseline TBL and ALP will be created, regardless of the time between the two maximum values. Dashed lines represent TBL and ALP cut-offs of 2× ULN and 3× ULN, respectively. A potential cholestatic liver injury case is circled and is defined as having a maximum postbaseline TBL equal to or exceeding 2× ULN within 30 days after maximum postbaseline ALP equal to or exceeding 3× ULN. The percentages of study participants falling in each of the three relevant quadrants of the plot (right upper, left upper, right lower) will be summarized in a table.

3.14.3.6. Severe Persistent Hyperglycemia Requiring Rescue Therapy

A summary of initiation of rescue therapy in response to severe, persistent hyperglycemia will be provided by treatment group.

3.14.3.7. Hypoglycemia

The following categories in accordance with the 2021 American Diabetes Association position statement on glycemic targets (ADA 2021) will be defined in the database.

Glucose Alert Value (Level 1):

- **Documented symptomatic hypoglycemia** is defined as any time a participant feels he/she is experiencing symptoms and/or signs associated with hypoglycemia, and has a blood glucose (BG) level of <70 mg/dL (<3.9 mmol/L) and ≥54 mg/dL (≥3.0 mmol/L).
- **Documented asymptomatic hypoglycemia** is defined as any event not accompanied by typical symptoms of hypoglycemia, but with a measured BG <70 mg/dL (<3.9 mmol/L) and ≥54 mg/dL (≥3.0 mmol/L).
- **Documented unspecified hypoglycemia** is defined as any event with no information about symptoms of hypoglycemia available, but with a measured BG <70 mg/dL (<3.9 mmol/L) and ≥54 mg/dL (≥3.0 mmol/L).

Documented Clinically Significant Hypoglycemia (Level 2):

- **Documented symptomatic hypoglycemia** is defined as any time a participant feels he/she is experiencing symptoms and/or signs associated with hypoglycemia and has a BG level of <54 mg/dL (<3.0 mmol/L).
- **Documented asymptomatic hypoglycemia** is defined as any event not accompanied by typical symptoms of hypoglycemia but with a measured BG <54 mg/dL (<3.0 mmol/L).
- **Documented unspecified hypoglycemia** is defined as any event with no information about symptoms of hypoglycemia available but with a measured BG <54 mg/dL (<3.0 mmol/L).

Severe Hypoglycemia (Level 3):

Severe hypoglycemia is defined as an episode with severe cognitive impairment requiring the assistance of another person to actively administer carbohydrate, glucagon, or other resuscitative actions. These episodes may be associated with sufficient neuroglycopenia to induce seizure or coma. Plasma glucose (PG) measurements may not be available during such an event, but neurological recovery attributable to the restoration of PG to normal is considered sufficient evidence that the event was induced by a low PG concentration.

Other hypoglycemia categories:

Nocturnal hypoglycemia is defined as any hypoglycemic event that occurs between bedtime and waking. If a hypoglycemic event meets the criteria of severe, the event would specifically be collected as an SAE. Serious hypoglycemia is defined by pharmacovigilance criteria and will also be captured with an SAE form.

To avoid duplicate reporting, all consecutive hypoglycemic events occurring within a 1-hour period will be considered as a single hypoglycemic event.

Hypoglycemia data will be censored at the time of receipt of new antihyperglycemic medication. This censoring will occur regardless of whether the reason for the alternative medication is rescue from severe, persistent hyperglycemia (with or without continuation of assigned study medication) or maintenance of glucose control in the event of cessation of assigned study medication.

Both the incidence (percent of participants experiencing ≥1 episode) and the rate (episodes/participant/year) of level 2 or level 3 hypoglycemia will be reported.

A listing of level 2 or 3 hypoglycemia will also be provided.

3.14.3.8. Immunogenicity

3.14.3.8.1. Definitions of Sample Anti-drug Antibody Status

At a high level, an individual sample is potentially examined multiple times, in a hierarchical procedure, to produce a sample anti-drug antibodies (ADA) assay result and potentially multiple cross-reactive antibodies assay results and multiple neutralizing antibodies (NAb) assay results.

The cut points used, the drug tolerance of each assay, and the possible values of titers are operating characteristics of the assay. Figure GPID.3.2 details a flow chart that reflects the multitiered testing approach.

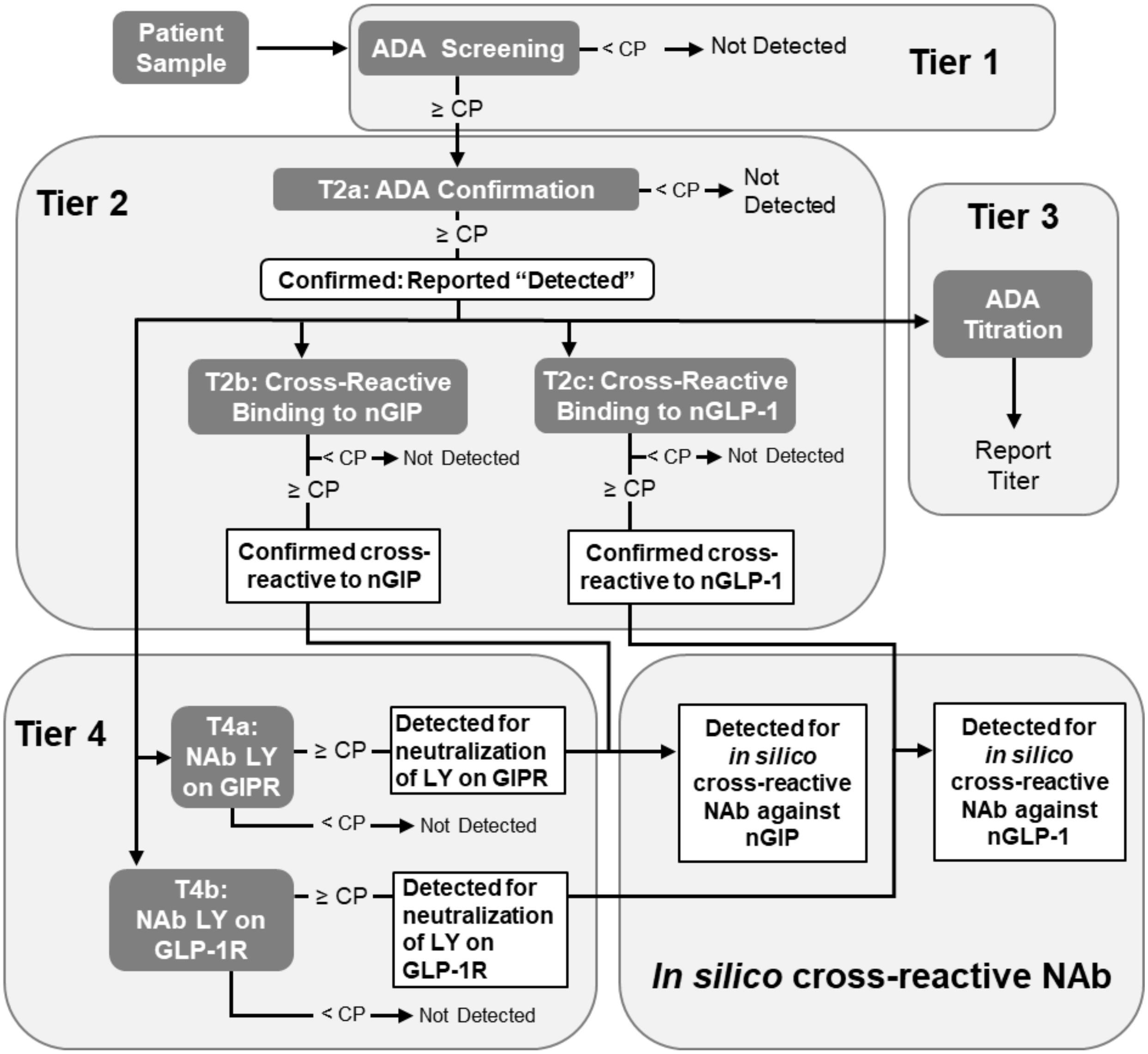

Abbreviations: ADA = antidrug antibodies; CP = cut point; GIPR = glucose-dependent insulinotropic polypeptide receptor; GLP-1R = glucagon-like peptide-1 receptor; LY = LY3298176 (tirzepatide); NAb = neutralizing antibodies; nGIP = native glucose-dependent insulinotropic polypeptide; nGLP-1 = native glucagon-like peptide-1; T2 = Tier 2; T4 = Tier 4.

Figure GPID.3.2. Flowchart of immunogenicity multitiered testing approach.

Table GPID.3.6 outlines results as reported from Tier 2a of the multitiered testing approach. Tier 4 results are reported similarly.

Table GPID.3.6. Sample ADA Assay Results

Sample Laboratory Result	Explanation
Detected	ADA are Detected and Confirmed.
Not Detected	The raw result as reported from the laboratory indicates Not Detected. The clinical interpretation of such results depends on other factors (see Table GPID.3.7).
NO TEST, QNS, etc.	Sample exists but was unevaluable by the assay.

Abbreviations: ADA = anti-drug antibodies; QNS = quantity not sufficient.

It may be the case that the presence of high concentrations of tirzepatide will affect ADA immunoassays, and conversely, that high levels of ADA may affect the measurement of tirzepatide concentrations. Thus, a tirzepatide drug concentration, assessed from a sample drawn at the same time as the ADA sample, plays a key role in the clinical interpretation of a sample when the laboratory results is Not Detected (see Table GPID.3.7).

Table GPID.3.7. Sample Clinical ADA Interpretation Results

Sample Clinical Interpretation	Explanation
ADA Present	ADA assay result is Detected.
ADA Not Present	ADA assay result is Not Detected, and simultaneous drug concentration is at a level that has been demonstrated to not interfere in the ADA detection method (that is, drug concentration is below the assay's drug tolerance level).<br>For participants receiving placebo, drug concentration is not assessed and is assumed to be below the assay's drug tolerance level.<br>If the drug concentration was planned but is not available for a treatment-period sample, a Not Detected sample will be declared ADA Not Present.
ADA Inconclusive	ADA assay result is Not Detected but the drug concentration in the sample is at a level that may cause interference in the ADA detection method.
ADA Missing	ADA sample not drawn, QNS, not tested, etc., causing there to be no laboratory result reported or the result is reported as "no test."

Abbreviations: ADA = antidrug antibodies; QNS = quantity not sufficient.

All ADA present samples will be evaluated for cross-reactive GIP (Tier 2b), cross-reactive GLP-1 (Tier 2c), NAb LY (tirzepatide) on GIP-R (Tier 4a), and NAb LY (tirzepatide) on GLP-1R (Tier 4b). If cross-reactive GIP is detected, NAb GIP on GIP-R (Tier 4c) is evaluated. If cross-reactive GLP-1 is detected, NAb GLP-1 on GLP-1R (Tier 4d) is evaluated (Figure GPID.3.2).

Similar terminology to Table GPID.3.8 applies for each type of cross-reactive and NAb assay. Importantly, each of these are distinct assays and, in general, have different assay operating characteristics.

The following are considered inconclusive for the NAb result:

- NAb LY on GIP-R: if the NAb result is not detected, and pharmacokinetic (PK) concentration is ≥ drug tolerance limit of the NAb LY on GIP-R assay
- NAb LY on GLP-1R: if the NAb result is not detected, and PK concentration is ≥ drug tolerance limit of the NAb LY on GLP-1R assay
- NAb GIP on GIP-R: if the NAb result is not detected, and PK concentration is ≥ drug tolerance limit of the NAb GIP on GIP-R assay
- NAb GLP-1 on GLP-1R: if the NAb result is not detected, and PK concentration is ≥ drug tolerance limit of the NAb GLP-1 on GLP-1R assay

To mitigate inconclusive cross-reactive NAb interpretations against native GIP and GLP-1 due to potential tirzepatide concentrations greater than or equal to the drug tolerance limit of the NAb GIP on GIP-R (Tier 4c) and NAb GLP-1 on GLP-1R (Tier 4d) assays, an *in silico* method utilizing results from Tiers 2b and 2c, Tiers 4a and 4b, and tirzepatide concentrations is introduced. The *in silico* method is outlined in Table GPID.3.8.

Table GPID.3.8. *In Silico* Classification for Cross-Reactive NAb

***In Silico* Classification**	**Cross-Reactive ADA Result**	**NAb Result**	**Circulating Tirzepatide Level (ng/mL)**	***In Silico* Cross-Reactive NAb Interpretation**
Cross-Reactive NAb to nGIP	Tier 2b: "Not Detected"	Tier 4a "Not Detected" Or Tier 4a: "Detected" or N/A or Missing	Any Value or Missing	Not Present
	Tier 2b: "Detected"	Tier 4a: "Not Detected"	< drug tolerance limit of Tier 4a assay	Not Present
	Tier 2b: "Detected"	Tier 4a: "Not Detected"	≥ drug tolerance limit of Tier 4a assay	Inconclusive
	Tier 2b: "Detected"	Tier 4a: "Detected"	< drug tolerance limit of Tier 4a assay	Present
	Tier 2b: "Detected"	Tier 4a: "Detected"	≥ drug tolerance limit of Tier 4a assay	Present
Cross-Reactive NAb to nGLP-1	Tier 2c: "Not Detected"	Tier 4b "Not Detected" Or Tier 4b: "Detected" or N/A or Missing	Any Value or Missing	Not Present
	Tier 2c: "Detected"	Tier 4b: "Not Detected"	< drug tolerance limit of Tier 4b assay	Not Present
	Tier 2c: "Detected"	Tier 4b: "Not Detected"	≥ drug tolerance limit of Tier 4b assay	Inconclusive
	Tier 2c: "Detected"	Tier 4b: "Detected"	< drug tolerance limit of Tier 4b assay	Present
	Tier 2c: "Detected"	Tier 4b: "Detected"	≥ drug tolerance limit of Tier 4b assay	Present

Abbreviations: ADA = antidrug antibodies; GIPR = glucose-dependent insulinotropic polypeptide receptor; GLP-1R = glucagon-like peptide-1 receptor; LY = tirzepatide; NAb = neutralizing antibody; nGIP = native glucose-dependent insulinotropic polypeptide; nGLP-1 = native glucagon-like peptide-1; Tier 2b = cross-reactive

ADA to nGIP; Tier 2c = cross-reactive ADA to nGLP-1; Tier 4a = NAb LY (tirzepatide) on GIPR; Tier 4b = NAb LY (tirzepatide) on GLP-1R.

Note: Only the drug tolerance limits of the Tier 4a and 4b assays are used for in silico classifications as they are lower than the drug tolerance limits of the Tier 2b and 2c assays, respectively.

3.14.3.8.2. Definitions of Immunogenicity Assessment Periods

Immunogenicity Baseline Observations: The baseline period for immunogenicity assessment for each participant includes all observations prior to the first dose of study treatment. In instances where multiple baseline observations are collected, the last nonmissing immunogenicity assessment prior to first administration of study drug is used to determine participant's ADA status and TE status (see below).

Immunogenicity Postbaseline Period Observations: Postbaseline period observations for each participant includes all observations after the first administration of study drug.

3.14.3.8.3. Definitions of Participant ADA Status

Treatment-emergent ADA (TE ADA)-evaluable participants: A participant with a nonmissing baseline ADA result and at least 1 nonmissing postbaseline ADA result.

TE ADA-unevaluable participant: any participant who does not meet the evaluable criteria.

Baseline ADA Present (preexisting antibody): ADA detected in a sample collected up to the first dose date and time.

Baseline ADA Not Present: ADA is not detected, and the corresponding PK concentration is missing or below the drug tolerance limit in a sample collected up to the first dose date and time.

TE ADA positive (TE ADA+) participant: an evaluable participant who had a:

- baseline status of ADA Not Present and at least 1 postbaseline status of ADA Present with titer ≥2× minimum required dilution (MRD), where the MRD is the minimum required dilution of the ADA assay, or
- baseline and postbaseline status of ADA Present, with the postbaseline titer being 2 dilutions (4-fold) greater than the baseline titer. That is, the participant has a baseline (B) status of ADA Present, with titer 1:B, and at least 1 postbaseline (P) status of ADA Present, with titer 1:P, with P/B ≥4.

As shown in Figure GPID.3.2, a titer is expected when the ADA assay result is Detected. On the occasion when the corresponding assay cannot be performed, in which case a titer value will be imputed for the purpose of TE ADA determination. A baseline sample with detected ADA and no titer is imputed to be the MRD (1:10), and a postbaseline sample with ADA detected and no titer is imputed to be one dilution above the MRD (1:20).

TE ADA-Inconclusive participant: a TE ADA-evaluable participant is TE ADA Inconclusive if ≥20% of the participant's postbaseline samples, drawn predose, are ADA Inconclusive and all remaining postbaseline samples are ADA Not Present.

TE ADA-negative (TE ADA-) participant: a TE ADA-evaluable participant is TE ADA- when the participant is not TE ADA+ and not TE ADA Inconclusive.

For each NAb assay, the following are defined:

NAb positive (NAb+) participant: a participant who is TE ADA+ and has a NAb positive sample in the postbaseline period.

NAb Inconclusive participant: a participant who is TE ADA+, is not NAb+, and all samples that have TE ADA+ titer have a NAb Inconclusive sample result.

NAb negative (NAb-) participant: a participant is neither NAb+ nor NAb Inconclusive.

Unless specified otherwise, these definitions of NAb are applicable to all NAb analyses, including cross-reactive NAb analyses, and cross-reactive antibodies.

3.14.3.8.4. Analyses to be Performed

The count and proportion of participants who are TE ADA+ will be tabulated by treatment group, where the proportion is relative to the number of TE ADA-evaluable participants, as defined above. The tabulation will include the count and proportion of participants with ADA Present at baseline, and the count and proportion of TE ADA+ participants exhibiting each type of cross-reactive antibodies and NAb. This analysis will be performed for the planned treatment period.

The cross-reactive NAb will exclude Tier 4c and 4d results but include the *in silico* classification as cross-reactive NAb for summary.

A summary will be provided of the count and percentage of tirzepatide-treated participants experiencing specific TEAE (see Table GPID.3.9) by participant TE ADA status (TE ADA+, TE ADA-, TE ADA Inconclusive). The PT will be ordered by decreasing incidence in TE ADA+ status group.

Table GPID.3.9. Adverse Events for Analysis with Immunogenicity Results

TEAE category	Criteria
Hypersensitivity reactions	Anaphylactic reaction SMQ (narrow or algorithm)
	Hypersensitivity SMQ (narrow)
	Angioedema SMQ (narrow)
	Severe Cutaneous Adverse Reaction SMQ (narrow)
	Vasculitis SMQ (narrow)
Injection site reactions	Injection site reaction HLT
	Infusion site reaction HLT
	Administration site reaction HLT

Abbreviations: HLT = high-level term; MedDRA = Medical Dictionary for Regulatory Activity; SMQ = standardized MedDRA query; TEAE = treatment-emergent adverse event.

Additional immunogenicity analyses as determined later may be presented. The relationship between antibody titers, PK parameters, and the pharmacodynamic response to tirzepatide may also be assessed.

3.14.3.9. Hypersensitivity Reactions

Two main analyses are performed in support of the assessment of potential immediate hypersensitivity, including anaphylaxis as well as potential nonimmediate hypersensitivity.

Time Period A, of potential immediate hypersensitivity, includes all TEAEs occurring from the start of study drug administration up to 24 hours after the end of study drug administration. For events without the hypersensitivity eCRF, only date (no time) information is collected. Among these events without time information, if the event occurred on the same date as the study drug injection date it will be included in Time Period A.

Time Period B, of potential nonimmediate hypersensitivity, includes all TEAEs occurring more than 24 hours after the end of study drug administration, but prior to subsequent drug administration.

Analyses for both time periods are based on the following:

- narrow and algorithm terms in *Anaphylactic reaction* SMQ (20000021) (analysis for algorithm term only applicable for Time Period A)
- narrow terms in *Angioedema* SMQ (20000024)
- narrow terms in *Severe cutaneous adverse reactions* SMQ (20000020)
- narrow terms in *Hypersensitivity* SMQ (20000214), and
- narrow terms in *Vasculitis* SMQ (20000174).

For the *Anaphylactic reaction* SMQ, each term is classified by scope (narrow, broad) and by category (A, B, C, D). All narrow terms are category A, and all broad terms are category B, C, or D. In addition to the usual narrow and broad searches, the SMQ defines an algorithm to further refine the cases of interest. For the Time Period A analysis, the *Anaphylactic reaction* SMQ algorithm will be included. The algorithm is based upon events that occur within Time Period A. The counts and percentages of participants who experienced a TEAE for the following will be analyzed for each of the 2 time periods:

- any narrow term from any one of the 4 SMQs indicated above (that is, combined search across narrow of all 4 SMQs)
- any narrow scope term within each SMQ, separately (that is, narrow SMQ search). For the Time Period A analysis, any term from Anaphylactic reaction SMQ algorithm.

Within each query, individual PTs that satisfied the queries will be summarized. For the Time Period A analysis, the *Anaphylactic reaction* SMQ algorithm will be summarized. Also, a single event may satisfy multiple SMQs, in which case the event contributes to every applicable SMQ.

3.14.3.9.1. Severe/Serious Hypersensitivity Reactions

The severe or serious cases of hypersensitivity will be considered as AESI. Summaries of severe/serious TE hypersensitivity reactions by treatment will be provided.

Approved on 26 Jun 2024 GMT

3.14.3.10. Injection Site Reactions

Injection site reactions, incidence, and rates, and related information reported via the Injection Site Reactions eCRF, will be summarized by treatment. Information to be summarized includes location of the reaction, timing of the reaction relative to study drug administration, and characteristics of the injection site reaction: erythema, induration, pain, pruritus, and edema.

Patient-based analysis and event-based analysis may be provided if necessary. The patient-based analysis summarizes all injection-site reaction (ISR) questionnaire forms for an individual participant with a single statistic, typically an extreme value. This analysis allows each participant to contribute only once for each parameter, at the expense of a focus on the most extreme events. By contrast, the event-based analysis summarizes all ISR questionnaire forms received, without regard to individual participants. This provides characteristics of ISR events as a proportion of all events for which questionnaire responses were provided, at the expense of some potential bias due to the differential contribution of individual patients to the analysis.

The counts and percentages of participants with TE ISRs will be summarized by treatment using MedDRA PTs. Detailed search criteria can be found in Appendix 2, Section 5.2.

Events will be ordered by decreasing frequency in the tirzepatide arm.

3.14.3.10.1. Severe/Serious Injection Site Reactions

The severe or serious ISRs (for example, abscess, cellulitis, erythema, hematomas/hemorrhage, exfoliation/necrosis, pain, subcutaneous nodules, swelling, induration, inflammation) will be considered as AESI.

The counts and percentage of participants with severe or serious TE ISRs will be summarized by treatment.

3.14.3.11. Major Adverse Cardiovascular Events

Major adverse cardiovascular events (MACE) reported by investigators are adjudicated by an independent CEC in a blinded fashion.

The following positively-adjudicated MACE will be considered as an AESI:

- myocardial infarction
- hospitalization for unstable angina
- coronary interventions (such as coronary artery bypass graft or percutaneous coronary intervention), and
- cerebrovascular events, including cerebrovascular accident (stroke) and transient ischemic attack.

Cardiovascular death and hospitalization for HF are not considered as AESI since they are reported as efficacy endpoints in Study GPID.

The counts and percentages of participants with adjudicated MACE may be summarized by treatment. In addition, MACE reported by investigator may also be summarized, although MACE reported by investigator is not considered as AESI.

A listing of participants reporting MACE, either reported by investigator or identified by the CEC, may be provided.

3.14.3.12. Major Depressive Disorder/Suicidal Ideation or Behavior

The severe or serious TE major depressive disorder/suicidal ideation or behavior will be captured as AESI. AEs will be searched using MedDRA PTs from SMQs narrow scope: 20000037 (*Suicide/self-injury*) and 20000167 (*Depression [excl suicide and self injury]*).

The counts and percentages of participants with TEAEs will be summarized by treatment group using MedDRA PT nested within SMQ. Events will be ordered by decreasing frequency in the tirzepatide arm nested within SMQ.

3.14.3.13. Renal Safety

Laboratory measures related to renal safety will be analyzed as specified for laboratory measurements in Section 3.14.5.

In addition, two shift tables examining renal function will be created. A minimum-to- minimum shift table of estimated glomerular filtration rate (eGFR) estimated by the Chronic Kidney Disease Epidemiology Collaboration (CKD-EPI) equation with unit as mL/min/1.73m^2, using categories (<30, ≥30 to <45, ≥45 to <60, ≥60 to <90, and ≥90 mL/min/1.73m^2). A maximum-to-maximum shift table of urine albumin-to-creatinine ratio (UACR), using the categories UACR <30 mg/g, 30 mg/g ≤ UACR ≤300 mg/g, UACR >300 mg/g (respectively, these represent normal, microalbuminuria, and macroalbuminuria).

MMRM analyses as described in Section 3.14 for eGFR and UACR will be provided. Log-transformation will be performed for UACR.

3.14.3.13.1. Acute Renal Events

As severe GI events may lead to dehydration, which could cause a deterioration in renal function including acute renal failure, dehydration events will be analyzed. Acute renal events associated with chronic renal failure exacerbation will also be captured.

The counts and percentages of participants with TE acute renal events will be summarized by treatment by using the MedDRA PTs contained in any of the following SMQs:

- Acute renal failure: narrow terms in *Acute renal failure* SMQ (20000003), and
- Chronic kidney disease: narrow terms in *Chronic kidney disease* SMQ (20000213).

Severe or serious renal events will be considered as AESI and will also be summarized by treatment

3.14.3.13.2. Dehydration

Dehydration events will be captured in the narrow terms in *Dehydration* SMQ (20000232).

The counts and percentages of participants with dehydration events will be summarized by treatment. Severe or serious TE dehydration will be considered as AESIs and summarized separately.

3.14.3.14. Thyroid Safety Monitoring

3.14.3.14.1. Calcitonin

The purpose of calcitonin measurements is to assess the potential of tirzepatide to affect thyroid C-cell function, which may indicate development of C-cell hyperplasia and neoplasms.

Observed calcitonin data (a thyroid-specific laboratory assessment) will be summarized by treatment and nominal visit.

The counts and percentages of participants with a maximum postbaseline calcitonin value in the following thresholds will be provided by treatment and baseline calcitonin categories (≤20 ng/L, >20 ng/L to ≤35 ng/L, >35 ng/L). Postbaseline categories are: ≤20 ng/L, >20 ng/L to ≤35 ng/L, >35 ng/L to ≤50 ng/L, >50 ng/L to ≤100 ng/L, and >100 ng/L.

3.14.3.14.2. C-Cell Hyperplasia and Thyroid Malignancies

TE thyroid malignancies and C-cell hyperplasia will be considered as AESI. Thyroid malignancies and C-cell hyperplasia will be identified using the MedDRA high-level term (HLT) for *Thyroid neoplasms malignant* and PT for *Thyroid C-cell hyperplasia*.

The counts and percentages of participants with TE thyroid C-cell hyperplasia and malignancies will be summarized.

3.14.3.15. Arrhythmias and Cardiac Conduction Disorders

Severe/serious TE arrhythmias and cardiac conduction disorders will be considered as AESI. The cardiovascular events will include clinically relevant rhythm and conduction disorders.

The TE arrhythmias and cardiac conduction disorders events will be identified using the MedDRA PTs. Detailed searching criteria can be found in Appendix 2, Section 5.2.

The counts and percentages of participants with treatment emergent arrhythmias and cardiac conduction disorders will be summarized by treatment and PT nested within SMQ. The PTs will be ordered with decreasing frequency in the tirzepatide arm within SMQ.

3.14.3.16. Malignancy

The malignancy events will be included using the MedDRA PTs contained in the *Malignant tumours* SMQ (20000194) narrow scope or *Tumours of unspecified malignancy* SMQ (20000195) narrow scope. Malignancy will be considered as an AESI.

The counts and percentages of participants with TE malignancy will be summarized by treatment.

3.14.3.17. Metabolic Acidosis

Severe/serious metabolic acidosis, including diabetic ketoacidosis, will be captured as AESI.

A listing of participants with TE events will be provided using the list of PTs provided in Appendix 2, Section 5.2.

Approved on 26 Jun 2024 GMT

3.14.3.18. Hypotension, Orthostatic Hypotension, and Syncope

The AE database will be searched using predefined PTs to identify events consistent with hypotension, orthostatic hypotension, and syncope.

The counts and percentages of participants with TE hypotension, orthostatic hypotension, and syncope will be summarized by treatment and PT. Detailed searching criteria can be found in Appendix 2, Section 5.2.

3.14.4. Vital Signs

Two sitting blood pressure and apical heart rate measurements are collected at each visit scheduled for vital sign collection. For the multiple records of an individual vital sign collected at the same visit, the average value will be used for data summaries and analyses. The vital signs collected in association with 6-minute walk test (6MWT) will be excluded from the vital signs analyses.

Descriptive summaries by treatment and by nominal scheduled visit will be provided for baseline and postbaseline values as well as change from baseline values.

An MMRM and/or an ANCOVA model as described in Section 3.14 may be conducted if necessary.

Counts and percentages of participants with abnormal sitting systolic blood pressure, sitting diastolic blood pressure, and heart rate will be presented by treatment for participants who have both baseline and at least 1 postbaseline result at any time during the entire study. To assess decreases, change from the minimum value during the baseline period to the minimum value during the postbaseline period will be used. To assess increases, changes from the maximum value during the baseline period to the maximum value during the postbaseline period will be used. Both planned and unplanned measurements will be included in the analysis. The criteria for identifying participants with vital signs abnormalities are listed in Table GPID.3.10.

Table GPID.3.10. Categorical Criteria for Abnormal Blood Pressure and Heart Rate Measurements

Parameter	Low	High
Systolic BP (mm Hg) (supine or sitting – forearm at heart level)	≤90 and decrease from baseline ≥20	≥140 and increase from baseline ≥20<br>≥129 and increase from baseline ≥20
Diastolic BP (mm Hg) (supine or sitting – forearm at heart level)	≤50 and decrease from baseline ≥10	≥90 and increase from baseline ≥10
Heart rate (bpm) (apical)	<50 and decrease from baseline ≥15	>100 and increase from baseline ≥15

Abbreviations: BP = blood pressure; bpm = beats per minute.

Approved on 26 Jun 2024 GMT

Additional analyses of heart rate

Counts and percentages of participants who have changes from baseline to postbaseline or postbaseline absolute heart rate will be summarized by treatment group for the categories listed in Table GPID.3.11.

Table GPID.3.11. Categorical Criteria for Additional Analyses of Heart Rate

Value at any postbaseline visit	>100 bpm,<br>>130 bpm
Value at 2 consecutive postbaseline visits	>100 bpm
Value at any 3 postbaseline visits	>100 bpm
Change from baseline to maximum at any visit	>20 bpm
Change from baseline at 2 consecutive visits	>20 bpm
Change from baseline at any 3 visits	>20 bpm

Abbreviation: bpm = beats per minute.

3.14.5. Clinical Laboratory Evaluation

All safety laboratory data will be reported using the International System of Units and conventional units. Limits from the performing lab will be used to define low (L) and high (H). Descriptive summaries by treatment and by nominal visit will be provided for the baseline and postbaseline values as well as the change from baseline values.

For selected laboratory analyte measurements collected quantitatively, observed and change from baseline values for each visit may be displayed in plots for participants who have both a baseline and at least 1 postbaseline planned measurement. Baseline will be defined as the last nonmissing observation during the baseline period. Unplanned measurements will be excluded from plots.

A shift table will be provided, including unplanned measurements. The shift table will include the number and percentage of participants with a change from baseline to postbaseline of normal or high to low, and normal or low to high. The proportion of participants shifted will be compared between treatments using Fisher's exact test.

A listing of abnormal findings will be created for laboratory analyte measurements, including qualitative measures. The listing will include participant identification, treatment group, laboratory collection date, study day, analyte name, and analyte finding. Other variables may be added, as appropriate.

The MMRM model or ANCOVA (if the MMRM model is not applicable), as described in Section 3.14, will be used for the analysis for the continuous measurements for selected lab tests with or without log-transformed (postbaseline measure/baseline measure) response variables. For measures analyzed using log-transformed values, the results will be presented with the scale back-transforming to the original, related scale.

3.15. Subgroup Analyses

3.15.1. Primary Efficacy Endpoints

The following subgroup variables will be considered for subgroup analyses if there are adequate number of participants in each subcategory:

- age group: <65, ≥65
- race: White, American Indian or Alaska Native, Black or African American, Asian, or Other
- sex: Male, Female
- ethnicity: Hispanic or Latino, Not Hispanic or Latino
- region: US, Central/South America, Asia, and Other
- baseline BMI (kg/m^2): <35, ≥35
- baseline BMI (kg/m^2): <35, ≥35 and <40, ≥40
- HF decompensation within 12 months of screening: Yes, No
- diagnosed T2DM at screening: Yes, No
- atrial fibrillation at baseline: Yes, No
- baseline eGFR (mL/min/1.73m^2): <60, ≥60
- N-terminal pro-B-type natriuretic peptide (NT-proBNP) at baseline: <200, ≥200
- NYHA class: Class II, Class III or IV
- baseline use of MRA: Yes, No
- baseline use of RAS inhibitors (ACE +ARB+ARNi): Yes, No
- baseline use of a beta blocker: Yes, No
- baseline use of sodium-glucose co-transporter 2 inhibitors: Yes, No
- baseline use of diuretics: Yes, No
- baseline left ventricular ejection fraction: <60, ≥60
- baseline systolic blood pressure: <130, ≥130
- baseline hsCRP: <2, ≥2
- baseline KCCQ-CSS: <Median, ≥Median
- coronary artery disease at baseline: Yes, No
- baseline heart rate: <70, ≥70
- baseline probability of HFpEF: <0.8, ≥0.8, and
- baseline waist-to-height ratio: <0.6, ≥0.6.

Subgroup analyses will be considered for the primary efficacy endpoints. An ANCOVA analysis similar to the model described in Section 3.13.1.4 will be considered for the KCCQ-CSS. A Cox proportional hazards model similar to the model described in Section 3.13.1.2 will be considered for the HF outcomes in each subcategory of the subgroup variable with more than 10 events for the corresponding primary endpoint. In addition, a full model with additional terms of subgroup and subgroup-by-treatment interactions will be used to obtain interaction p-values.

The subgroup analyses may also be performed for the key secondary efficacy endpoints using the primary analysis approaches defined in Section 3.13.2. The same analysis model for the corresponding endpoint will be conducted in each subcategory of the subgroup variable to obtain

Approved on 26 Jun 2024 GMT

estimates of the treatment group difference. In addition, a full model with additional terms of subgroup and subgroup-by-treatment interactions will be used to obtain interaction p-values.

3.15.2. Safety in Special Groups and Situations

3.15.2.1. Intrinsic Factors

A subgroup analysis will be presented for common TEAEs, for the purposes of presentation in the summary of clinical safety (SCS). The subgroups will be

- age group: <65, ≥65 years
- sex: female, male
- race: American Indian or Alaska Native, Asian, Black/African American, Native Hawaiian or Other Pacific Islander, White, and Other
- BMI: ≥30 and <35, ≥35 and <40, ≥40 kg/m^2
- T2DM: Yes, No, and
- eGFR: <60 mL/min/1.73m^2, ≥60 mL/min/1.73m^2.

The response variable will be each most common TEAE. The explanatory variables will be treatment, subgroup, and treatment-by-subgroup interaction. Within each subgroup category, odds ratios (treatment over placebo) and associated p-values will be provided.

An additional summary of AEs will be provided for the age groups of <65, ≥65 to <75, ≥75 to <85, and ≥85 years to meet expectations from the European Union (EMA 2014). A summary table will be created similarly to Table 12.12 in the PHUSE AE white paper (PHUSE 2017). Rows of the table include number of participants with at least 1 of the following:

- TEAE
- SAE (separate rows for fatal, hospitalization, life-threatening, disability, other)
- AEs leading to study intervention discontinuation
- Accidents and injuries (SMQ)
- Cardiac disorders (SOC)
- Infections and infestations (SOC)
- Nervous system disorders (SOC)
- Psychiatric disorders (SOC)
- Vascular disorders (SOC)
- Central nervous system vascular disorders (SMQ)
- Anticholinergic syndrome (PT),
- Fractures, or
- Hypotension, falls, fractures.

Approved on 26 Jun 2024 GMT

3.15.2.2. Extrinsic Factors

Subset analyses will be conducted for region and ethnicity for the SCS.

The following regions will be considered:

- United States
- Central/South America (Argentina, Brazil, Mexico)
- Asia (China, India, Taiwan), and
- Other (Israel, Russia).

3.16. Interim Analyses and Data Monitoring Committee

The details for the interim analyses and data monitoring committee (DMC) will be provided in the DMC charter.

4. Unblinding Plan

Details of the blinding and unblinding will be provided in the blinding and unblinding plan document for Study GPID.

5. Supporting Documentation

5.1. Appendix 1: Kansas City Cardiomyopathy Questionnaire - Scoring Instructions

The Kansas City Cardiomyopathy Questionnaire
Scoring Instructions

There are 10 summary scores within the KCCQ, which are calculated as follows:

1. **Physical Limitation**

   - Code responses to each of Questions 1a-f as follows:

     Extremely limited = 1
     Quite a bit limited = 2
     Moderately limited = 3
     Slightly limited = 4
     Not at all limited = 5
     Limited for other reasons or did not do = <*missing value*>

   - If at least three of Questions 1a-f are not missing, then compute

     Physical Limitation Score = 100*[(mean of Questions 1a-f actually answered) – 1]/4

     (see footnote at end of this document for explanation of meaning of "actually answered")

2. **Symptom Stability**

   - Code the response to Question 2 as follows:

     Much worse = 1
     Slightly worse = 2
     Not changed = 3
     Slightly better = 4
     Much better = 5
     I've had no symptoms over the last 2 weeks = 3

   - If Question 2 is not missing, then compute

     Symptom Stability Score = 100*[(Question 2) – 1]/4

3. **Symptom Frequency**

   - Code responses to Questions 3, 5, 7 and 9 as follows:

     <u>Question 3</u>
     Every morning = 1
     3 or more times a week but not every day = 2
     1-2 times a week = 3
     Less than once a week = 4
     Never over the past 2 weeks = 5

3. Symptom Frequency (cont.)

Questions 5 and 7
All of the time = 1
Several times a day = 2
At least once a day = 3
3 or more times a week but not every day = 4
1-2 times a week = 5
Less than once a week = 6
Never over the past 2 weeks = 7

Question 9
Every night = 1
3 or more times a week but not every day = 2
1-2 times a week = 3
Less than once a week = 4
Never over the past 2 weeks = 5

- If at least two of Questions 3, 5, 7 and 9 are not missing, then compute:

S3 = [(Question 3) – 1]/4
S5 = [(Question 5) – 1]/6
S7 = [(Question 7) – 1]/6
S9 = [(Question 9) – 1]/4

Symptom Frequency Score = 100*(mean of S3, S5, S7 and S9)

4. Symptom Burden

- Code responses to each of Questions 4, 6 and 8 as follows:

Extremely bothersome = 1
Quite a bit bothersome = 2
Moderately bothersome = 3
Slightly bothersome = 4
Not at all bothersome = 5
I've had no swelling/fatigue/shortness of breath = 5

- If at least one of Questions 4, 6 and 8 is not missing, then compute

Symptom Burden Score = 100*[(mean of Questions 4, 6 and 8 actually answered) – 1]/4

5. Total Symptom Score

= mean of the following available summary scores:
Symptom Frequency Score
Symptom Burden Score

© Copyright 2000 – 2003 John Spertus MD MPH

Approved on 26 Jun 2024 GMT

6. Self-Efficacy

- Code responses to Questions 10 and 11 as follows:

  Question 10
  Not at all sure = 1
  Not very sure = 2
  Somewhat sure = 3
  Mostly sure = 4
  Completely sure = 5

  Question 11
  Do not understand at all = 1
  Do not understand very well = 2
  Somewhat understand = 3
  Mostly understand = 4
  Completely understand = 5

- If at least one of Questions 10 and 11 is not missing, then compute

  Self-Efficacy Score = 100*[(mean of Questions 10 and 11 actually answered) – 1]/4

7. Quality of Life

- Code responses to Questions 12, 13 and 14 as follows:

  Question 12
  It has extremely limited my enjoyment of life = 1
  It has limited my enjoyment of life quite a bit = 2
  It has moderately limited my enjoyment of life = 3
  It has slightly limited my enjoyment of life = 4
  It has not limited my enjoyment of life at all = 5

  Question 13
  Not at all satisfied = 1
  Mostly dissatisfied = 2
  Somewhat satisfied = 3
  Mostly satisfied = 4
  Completely satisfied = 5

  Question 14
  I felt that way all of the time = 1
  I felt that way most of the time = 2
  I occasionally felt that way = 3
  I rarely felt that way = 4
  I never felt that way = 5

© Copyright 2000 – 2003 John Spertus MD MPH

7. Quality of Life (cont.)

- If at least one of Questions 12, 13 and 14 is not missing, then compute

  Quality of Life Score = 100*[(mean of Questions 12, 13 and 14 actually answered) – 1]/4

8. Social Limitation

- Code responses to each of Questions 15a-d as follows:

  Severely limited = 1
  Limited quite a bit = 2
  Moderately limited = 3
  Slightly limited = 4
  Did not limit at all = 5
  Does not apply or did not do for other reasons = *<missing value>*

- If at least two of Questions 15a-d are not missing, then compute

  Social Limitation Score = 100*[(mean of Questions 15a-d actually answered) – 1]/4

9. Overall Summary Score

= mean of the following available summary scores:
Physical Limitation Score
Total Symptom Score
Quality of Life Score
Social Limitation Score

10. Clinical Summary Score

= mean of the following available summary scores:
Physical Limitation Score
Total Symptom Score

Note: references to "**means of questions actually answered**" imply the following.

- If there are *n* questions in a scale, and the subject must answer *m* to score the scale, but the subject answers only *n-i*, where *n-i* >= *m*, calculate the **mean of those questions** as
  (sum of the responses to those *n-i* questions) / (*n-i*)
  not
  (sum of the responses to those *n-i* questions) / *n*

If doing these calculations seems like too much trouble, consider using one of our tools – available at www.cvoutcomes.org:

- SAS or SPSS code
- Excel spreadsheets
- Web data services

© Copyright 2000 – 2003 John Spertus MD MPH

Approved on 26 Jun 2024 GMT

<table>
<tr><th>Question No.</th><th>Domain</th><th>Question</th><th>Response</th></tr>
<tr><td>1a</td><td>Physical limitation</td><td>How much are you limited by heart failure (shortness of breath or fatigue) in dressing yourself over the past 2 weeks?</td><td rowspan="6">1: Extremely limited
2: Quite a bit limited
3: Moderately limited
4: Slightly limited
5: Not at all limited
0: Limited for other reasons or did not do the activity</td></tr>
<tr><td>1b</td><td>Physical limitation</td><td>How much are you limited by heart failure (shortness of breath or fatigue) in showering/bathing over the past 2 weeks?</td></tr>
<tr><td>1c</td><td>Physical limitation</td><td>How much are you limited by heart failure (shortness of breath or fatigue) in walking 1 block on level ground over the past 2 weeks?</td></tr>
<tr><td>1d</td><td>Physical limitation</td><td>How much are you limited by heart failure (shortness of breath or fatigue) in doing yard work, housework or carrying groceries over the past 2 weeks?</td></tr>
<tr><td>1e</td><td>Physical limitation</td><td>How much are you limited by heart failure (shortness of breath or fatigue) in climbing a flight of stairs without stopping over the past 2 weeks?</td></tr>
<tr><td>1f</td><td>Physical limitation</td><td>How much are you limited by heart failure (shortness of breath or fatigue) in hurrying or jogging over the past 2 weeks?</td></tr>
</table>

Question No.	Domain	Question	Response
2	Symptom Stability	Compared with 2 weeks ago, have your symptoms of heart failure (shortness of breath, fatigue, or ankle swelling) changed?	1: Much worse<br>2: Slightly worse<br>3: Not changed<br>4: Slightly better<br>5: Much better<br>3.0: I had no symptoms over the last 2 weeks
3	Symptom Frequency	Over the past 2 weeks, how many times did you have swelling in your feet, ankles or legs when you woke up in the morning?	1: Every morning<br>2: 3 or more times a week, but not every day<br>3: 1-2 times a week<br>4: Less than once a week<br>5: Never over the past 2 weeks
4	Symptom Burden	Over the past 2 weeks, how much has swelling in your feet, ankles or legs bothered you?	1: Extremely bothersome<br>2: Quite a bit bothersome<br>3: Moderately bothersome<br>4: Slightly bothersome<br>5: Not at all bothersome<br>5.0: I've had no swelling

Question No.	Domain	Question	Response
5	Symptom Frequency	Over the past 2 weeks, on average, how many times has fatigue limited your ability to do what you want?	1: All of the time,<br>2: Several times per day<br>3: At least once a day<br>4: 3 or more times per week but not every day<br>5: 1-2 times per week<br>6: Less than once a week<br>7: Never over the past 2 weeks
6	Symptom Burden	Over the past 2 weeks, how much has your fatigue bothered you?	1: Extremely bothersome<br>2: Quite a bit bothersome<br>3: Moderately bothersome<br>4: Slightly bothersome<br>5: Not at all bothersome<br>5.0: I’ve had no fatigue
7	Symptom Frequency	Over the past 2 weeks, on average, how many times has shortness of breath limited your ability to do what you wanted?	1: All of the time<br>2: Several times per day<br>3: At least once a day<br>4: 3 or more times per week but not every day<br>5: 1-2 times per week<br>6: Less than once a week<br>7: Never over the past 2 weeks

Question No.	Domain	Question	Response
8	Symptom Burden	Over the past 2 weeks, how much has your shortness of breath bothered you?	1: Extremely bothersome<br>2: Quite a bit bothersome<br>3: Moderately bothersome<br>4: Slightly bothersome<br>5: Not at all bothersome<br>5.0: I've had no shortness of breath
9	Symptom Frequency	Over the past 2 weeks, on average, how many times have you been forced to sleep sitting up in a chair or with at least 3 pillows to prop you up because of shortness of breath?	1: Every night<br>2: 3 or more times a week, but not every day<br>3: 1-2 times a week<br>4: Less than once a week<br>5: Never over the past 2 weeks
10	Self-efficacy	Heart failure symptoms can worsen for a number of reasons. How sure are you that you know what to do, or whom to call, if your heart failure gets worse?	1: Not at all sure<br>2: Not very sure<br>3: Somewhat sure<br>4: Mostly sure<br>5: Completely sure
11	Self-efficacy	How well do you understand what things you are able to do to keep your heart failure symptoms from getting worse?	1: Do not understand at all<br>2: Do not understand very well<br>3: Somewhat understand<br>4: Mostly understand<br>5: Completely understand

Approved on 26 Jun 2024 GMT

Question No.	Domain	Question	Response
12	Quality of Life	Over the past 2 weeks, how much has your heart failure limited your enjoyment of life?	1: It has extremely limited my enjoyment of life<br>2: It has limited my enjoyment of life quite a bit<br>3: It has moderately limited my enjoyment of life<br>4: It has slightly limited my enjoyment of life<br>5: It has not limited my enjoyment of life at all
13	Quality of Life	If you had to spend the rest of your life with your heart failure the way it is right now, how would you feel about this?	1: Not at all satisfied<br>2: Mostly dissatisfied<br>3: Somewhat satisfied<br>4: Mostly satisfied<br>5: Completely satisfied
14	Quality of Life	Over the past 2 weeks, how often have you felt discouraged or down in the dumps because of your heart failure?	1: I felt that way all of the time<br>2: I felt that way most of the time<br>3: I occasionally felt that way<br>4: I rarely felt that way<br>5: I never felt that way

Question No.	Domain	Question	Response
15a	Social Limitation	How much have your heart failure limited your participation in hobbies, recreational activities over the past 2 weeks?	1: Severely limited<br>2: Limited quite a bit<br>3: Moderately limited<br>4: Slightly limited<br>5: Did not limited at all<br>6: Does not apply or did not do for other reasons
15b	Social Limitation	How much have your heart failure limited your participation in working or doing household chores over the past 2 weeks?	
15c	Social Limitation	How much have your heart failure limited your participation in visiting family or friends out of your home over the past 2 weeks?	
15d	Social Limitation	How much have your heart failure limited your participation in intimate relationships with loved ones over the past 2 weeks?	

5.2. Appendix 2: Search Criteria for Special Safety Topics

Diabetic Retinopathy Complications

Diabetic retinopathy complications will be identified using the following MedDRA PTs:

Preferred Term	Code
Arteriosclerotic retinopathy	10063452
Blindness	10005169
Blindness transient	10005184
Blindness unilateral	10005186
Cystoid macular oedema	10058202
Diabetic blindness	10012646
Diabetic eye disease	10012661
Diabetic retinal oedema	10012688
Diabetic retinopathy	10012689
Diabetic uveitis	10012692
Exudative retinopathy	10015901
Eye laser surgery	10057105
Fundoscopy	10017519
Fundoscopy abnormal	10017520
Intra-ocular injection	10057098
Macular detachment	10075873
Macular oedema	10025415
Maculopathy	10025425
Non-proliferative retinopathy	10081568
Phacotrabeculectomy	10059276
Retinal collateral vessels	10077911
Retinal cryoablation	10074908
Retinal detachment	10038848
Retinal exudates	10038862
Retinal haemorrhage	10038867
Retinal laser coagulation	10038873
Retinal neovascularisation	10055666
Retinal oedema	10038886
Retinal operation	10062107
Retinal thickening	10077890
Retinal vascular disorder	10038901
Retinal vascular occlusion	10038903
Retinal vein occlusion	10038907
Retinopathy	10038923

Preferred Term	Code
Retinopathy haemorrhagic	10051447
Retinopathy hypertensive	10038926
Retinopathy proliferative	10038934
Sudden visual loss	10042441
Vision blurred	10047513
Visual acuity reduced	10047531
Visual acuity reduced transiently	10047532
Visual impairment	10047571
Vitrectomy	10047644

Arrhythmias and Cardiac Conduction Disorders

TE arrhythmias, arrhythmias, and cardiac conduction disorders will be considered as AESI. Cardiovascular events will include clinically relevant rhythm and conduction disorders. TE arrhythmias and cardiac conduction disorders events will be included using the MedDRA PT contained in any of the following SMQs:

1) Arrhythmias:
    - For symptoms: *Arrhythmia related investigations, signs and symptoms* SMQ (20000051), narrow and broad terms
    - For supraventricular arrhythmias: in *Cardiac arrhythmia* SMQ, under the tachyarrhythmia sub SMQ
        - *Supraventricular tachyarrhythmia* SMQ (20000057), broad and narrow terms
        - *Tachyarrhythmia terms, nonspecific* SMQ (20000164), narrow terms only, and
        - *Ventricular tachyarrhythmia* SMQ (20000058), narrow terms only.
2) Cardiac Conduction Disorders
    - *Conduction defects* SMQ (20000056), narrow terms only, and
    - *Cardiac conduction disorders* High Level Term (HLT; 10000032), all PTs.

Injection Site Reactions

TE ISRs will be summarized by treatment using the MedDRA PT in any of the following:

- HLT of *Injection site reaction*
- HLT of *Administration site reaction*
- HLT of *Infusion Site Reactions*

Acute Gallbladder Disease

All events of TEAE biliary colic, cholecystitis, or other suspected events related to gallbladder disease will be summarized by treatment groups by PT with decreasing frequency under following SMQs:

- narrow PTs in *Gallbladder related disorders* SMQ (20000124)
- narrow PTs in *Biliary tract disorders* SMQ (20000125), and
- narrow PTs in *Gallstone related disorders* SMQ (20000127).

Hepatic Treatment-Emergent Adverse Events

TE potentially drug-related hepatic disorders will be summarized by treatment using the MedDRA PTs contained in any of the following SMQs:

- broad and narrow terms in the *Liver related investigations, signs and symptoms* SMQ (20000008)
- broad and narrow terms in the *Cholestasis and jaundice of hepatic origin* SMQ (20000009)
- broad and narrow terms in the *Hepatitis non-infections* SMQ (20000010)
- broad and narrow terms in the *Hepatic failure, fibrosis and cirrhosis and other liver damage* SMQ (20000013), and
- narrow terms in the *Liver-related coagulation and bleeding disturbances* SMQ (20000015).

Metabolic Acidosis

Metabolic acidosis will be identified using the following MedDRA PTs:

Preferred Term	Code
Blood ketone body	10057593
Blood ketone body increased	10057594
Blood ketone body present	10057598
Diabetic ketoacidosis	10012671
Diabetic ketoacidotic hyperglycaemic coma	10012672
Diabetic ketosis	10012673
Euglycaemic diabetic ketoacidosis	10080061
Ketoacidosis	10023379
Ketonuria	10023388
Ketosis	10023391
Lactic acidosis	10023676
Urine ketone body	10059222
Urine ketone body present	10057597

Approved on 26 Jun 2024 GMT

Hypotension, Orthostatic Hypotension, and Syncope

Hypotension, orthostatic hypotension, and syncope will identified using the following MedDRA PTs:

Preferred Term	Code
Diastolic hypotension	10066077
Hypotension	10021097
Hypotensive crisis	10083659
Orthostatic hypotension	10031127
Blood pressure ambulatory decreased	10005731
Blood pressure decreased	10005734
Blood pressure diastolic decreased	10005737
Blood pressure orthostatic decreased	10053356
Blood pressure systolic decreased	10005758
Mean arterial pressure decreased	10026983
Blood pressure orthostatic	10053352
Dizziness	10013573
Presyncope	10036653
Syncope	10042772
Drop attacks	10013643
Loss of consciousness	10024855

5.3. Appendix 3: Cardiac Magnetic Resonance Imaging Substudy

This section is applicable to the participants who are enrolled in the cardiac magnetic resonance imaging (MRI) addendum.

This addendum applies to a subset of participants (approximately 150 participants) enrolled at selected sites that have the technical capability of conducting a cardiac MRI.

The participant demographics and baseline characteristics for the MRI substudy will be summarized for all participants enrolled in the MRI substudy.

Summaries of study disposition and study drug disposition will be provided for all participants enrolled in the MRI substudy.

MRI analyses will be guided by the treatment policy strategy and conducted for participants who are enrolled in the addendum, received at least 1 dose of study drug, and have MRI measurements at both baseline and postbaseline. The baseline MRI is defined as the MRI measurement taken prior or within 7 days of the second dose of the study treatment. The measurement for the Week 52 MRI will include a measurement taken either at 52 weeks, or within 105 days after 52 weeks, or at the early discontinuation visit if the discontinuation occurs prior to 52 weeks.

Objectives	Endpoints	Analytical Approaches
Evaluation of cardiac function and structure by cardiac MRI	Changes from baseline to Week 52 for the following:<br>Structural and functional parameters<br>• Left ventricular mass and index (LVM and LVMI, respectively)<br>• Left ventricular end diastolic volume and index (LVEDV and LVEDVI, respectively)<br>• Left ventricular end systolic volume and index (LVESV and LVESVI, respectively)<br>• Left atrial volume and index (LAV and LAVI, respectively)<br>• Left ventricular ejection fraction (LVEF)<br>• Left ventricular cardiac output (LVCO)<br>• Left ventricular stroke volume (LVSV)<br>Feature tracking<br>• Left ventricular global longitudinal strain (LVGLS)<br>• Left ventricular global circumferential strain (LVGCS)<br>• Left apical endocardial global longitudinal strain (LAEGLS)<br>• Left apical endocardial global circumferential strain (LAEGCS)<br>Adipose tissue volumes<br>• Epicardial fat volume<br>• Pericardial fat volume	Change from baseline to Week 52 for each parameter will be compared between treatment arms using an ANCOVA approach. The model will include treatment, the stratification factors of diagnosed T2DM (Y/N) and BMI group (<35, ≥35 kg/m^2), and the baseline value for the parameter.<br>Summary statistics for MRI parameters at baseline and at Week 52 will be provided. The treatment comparison at baseline will be performed using an ANOVA model.

Abbreviations: ANCOVA = analysis of covariance; ANOVA = analysis of variance; BMI= body mass index; MRI = magnetic resonance imaging; T2DM = type 2 diabetes mellitus.

5.4. Appendix 4: Statistical Analysis for China

Analyses will be performed for the following subpopulation:

- participants enrolled in China (mainland China and Taiwan).

The analysis methods for this subpopulation will be similar to those described for the main part SAP GPID. If there is not a sufficient number of participants in the subpopulation, summary statistics will be provided.

The analyses to be included will be documented in a separate list of analyses which should include disposition, demographics, and selected efficacy and safety endpoints.

6. References

[ADA] American Diabetes Association. 6. Glycemic targets: Standards of medical care in diabetes - 2021. *Diabetes Care.* 2021:44(suppl 1):S73-S84. https://doi.org/10.2337/dc21-S006

Dong G, Qiu J, Wang D. The stratified win ratio. *Journal of Biopharmaceutical Statistics*, 2018; 778-796. https://doi.org/10.1080/10543406.2017.1397007

Ghosh D, Lin DY. Nonparametric analysis of recurrent events and death. *Biometrics.* 2000; 56(2):554-562. https://doi.org/10.1111/j.0006-341x.2000.00554.x

Green CP, Porter CB, Bresnahan DR, Spertus JA. Development and evaluation of the Kansas City Cardiomyopathy Questionnaire: a new health status measure for heart failure. *J Am Coll Cardiol*. 2000;35(5):1245-1255. https://doi.org/10.1016/s0735-1097(00)00531-3

Joseph SM, Novak E, Arnold SV, et al. Comparable performance of the Kansas City Cardiomyopathy Questionnaire in patients with heart failure with preserved and reduced ejection fraction. *Circ Heart Fail.* 2013;6(6):1139-1146. https://doi.org/10.1161/CIRCHEARTFAILURE.113.000359

Lin DY, Wei LJ, Yang I, Ying Z. Semiparametric regression for the mean and rate functions of recurrent events. *Journal of the Royal Statistical Society, Series B.* 2000;62:711–730. https://doi.org/10.1111/1467-9868.00259

Pocock SJ, Ariti CA, Collier TJ, Wang D. The win ratio: a new approach to the analysis of composite endpoints in clinical trials based on clinical priorities. *Eur Heart J.* 2012;33(2):176-182. https://doi.org/10.1093/eurheartj/ehr352

PHUSE. Analysis and displays associated with adverse events: focus on adverse events in phase 2-4 clinical trials and integrated summary document. February 2017. Accessed June 24, 2024. https://phuse.s3.eu-central-1.amazonaws.com/Deliverables/Standard+Analyses+and+Code+Sharing/Analyses+and+Displays+Associated+with+Adverse+Events+Focus+on+Adverse+Events+in+Phase+2-4+Clinical+Trials+and+Integrated+Summary.pdf

Rubin DB. *Multiple Imputation for Nonresponse in Surveys*. New York: John Wiley & Sons Inc.; 1987.

Van Elteren, PH. On the combination of independent two sample tests of Wilcoxon. *Bull Inst Int Stats.* 1960;37 :351–361.

Reddy YNV, Carter RE, Sundaram V, Kaye DM, et al. An evidence-based screening tool for heart failure with preserved ejection fraction: The HFpEF-ABA score. *Nature Medicine*, 2024; in press

Approved on 26 Jun 2024 GMT

Signature Page for VV-CLIN-064990 v2.0

Approval	PPD<br>[redacted]24 12:10:19 GMT+0000

Signature Page for VV-CLIN-064990 v2.0

1. Type C Briefing Document

LY3298176 (tirzepatide)
IND [redacted]

Meeting Date: 23 January 2024

United States Food and Drug Administration
Office of Cardiology, Hematology, Endocrinology, and Nephrology
Division of Cardiology and Nephrology

Eli Lilly and Company
Lilly contact: PPD
Assoc. Director, PPD
Phone: PPD
e-mail: PPD

Confidential Information

This document contains trade secrets, or commercial or financial information, privileged and confidential, delivered in confidence and reliance that such information will not be released to the public without the express written consent of Eli Lilly and Company.

Note to Regulatory Authorities: This document may contain protected personal data and/or commercially confidential information exempt from public disclosure. Eli Lilly and Company requests consultation regarding release/redaction prior to any public release. In the United States, this document is subject to Freedom of Information Act (FOIA) Exemption 4 and may not be reproduced or otherwise disseminated without the written approval of Eli Lilly and Company or its subsidiaries.

Document ID: VV-REG-271498

Approved on 01 Dec 2023 GMT

2. Table of Contents

Section **Page**

1. Type C Briefing Document 1
2. Table of Contents 2
3. Introduction 7
3.1. Tirzepatide Description 7
3.2. Intended Indication 7
3.3. Obesity-Related HFpEF: a Specific HFpEF Subphenotype 7
3.4. Tirzepatide in Obesity-Related HFpEF 9
3.5. SUMMIT Study 10
3.6. Previous Regulatory Interaction with FDA 11
3.7. Objective of This Briefing Document 11
4. Meeting Objectives and Topics for Discussion 12
4.1. Meeting Objectives 12
4.2. Topics for Discussion 12
5. Revised Primary Endpoint Strategy for SUMMIT 14
5.1. Question 1: Proposed Revision of Primary Endpoints 14
5.1.1. Rationale for Worsening Heart Failure Events as One of the Dual Primary Endpoint 14
5.1.2. Rationale for KCCQ-CSS as the Other Primary Endpoint 15
5.2. Question 2: Proposed Definition of Heart Failure Events 17
5.2.1. Rationale 17
5.2.2. Previous Definition of HF Events 18
5.2.3. Revised Definition of Worsening HF Events 18
5.3. Question 3: Proposed Statistical Analysis for the Primary and Key Secondary Endpoints 19
5.3.1. Statistical Hypotheses 19
5.3.2. Estimand for Primary Analyses 19
5.3.3. Primary Endpoints and Analysis 20
5.3.4. Sample Size Justification and Power Calculation 21
6. References 22

List of Tables

Table		Page
Table 5.1.	Previous Primary and Key Secondary Objectives and Endpoints for the SUMMIT Study	15
Table 5.2.	Revised Primary and Key Secondary Objectives and Endpoints for SUMMIT	16

List of Figures

Figure **Page**

Figure 3.1. Chemical structure of tirzepatide with the standard single-letter amino acid code with the exception of residues aminoisobutyric acid 2, aminoisobutyric acid 13, and lysine 20, where the structures of these amino acid residues are depicted. 7

List of Appendices

Appendix **Page**

Appendix 1. Draft Protocol Amendment for SUMMIT..25

Abbreviations and Definitions

Term	Definition
**BMI**	body mass index
**CV**	cardiovascular
**ED**	emergency department
**GIP**	glucose-dependent insulinotropic polypeptide
**GLP-1**	glucagon-like peptide-1
**GPID**	I8F-MC-GPID
**IV**	intravenous
**HF**	heart failure
**HFpEF**	heart failure with preserved ejection fraction
**HR**	hazard ratio
**hsCRP**	high-sensitivity C-reactive protein
**KCCQ**	Kansas City Cardiomyopathy Questionnaire
**KCCQ-CSS**	Kansas City Cardiomyopathy Questionnaire – Clinical Summary Score
**MTD**	maximum tolerated dose
**6MWD**	6-minute walk distance
**T2D**	type 2 diabetes mellitus

Approved on 01 Dec 2023 GMT

3. Introduction

The briefing document provides information for a Type C meeting to receive feedback from FDA on proposed changes to the endpoints of the Phase 3 Study SUMMIT (I8F-MC-GPID [GPID]) for treatment of patients with HFpEF (left ventricular ejection fraction ≥50%) and BMI ≥30 kg/m^2.

3.1. Tirzepatide Description

Tirzepatide is a GIP and GLP-1 receptor agonist. It is an amino acid sequence including a C20 fatty diacid moiety that enables albumin binding and prolongs the half-life. Tirzepatide selectively binds to and activates both the GIP and GLP-1 receptors, the targets for native GIP and GLP-1.

Figure 3.1 illustrates the chemical structure of tirzepatide.

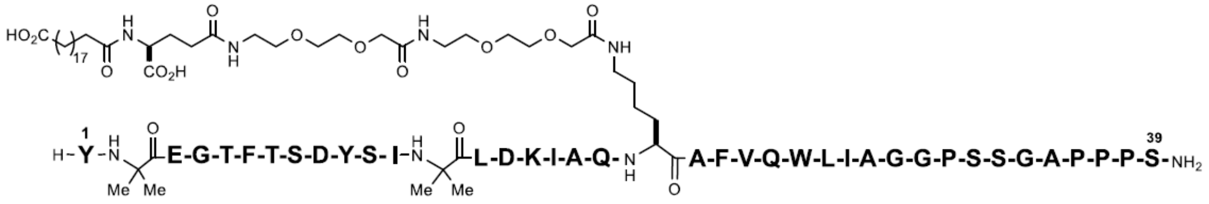

Figure 3.1. Chemical structure of tirzepatide with the standard single-letter amino acid code with the exception of residues aminoisobutyric acid 2, aminoisobutyric acid 13, and lysine 20, where the structures of these amino acid residues are depicted.

Tirzepatide is indicated

- as an adjunct to diet and exercise to improve glycemic control in adults with T2D, and
- as an adjunct to a reduced-calorie diet and increased physical activity for chronic weight management in adults with an initial BMI of 30 kg/m^2 or greater (obesity) or 27 kg/m^2 or greater (overweight) in the presence of at least 1 weight-related comorbid condition, for example, hypertension, dyslipidemia, T2D, obstructive sleep apnea, or CV disease.

3.2. Intended Indication

Tirzepatide is indicated to reduce the risk of HF-related events and to improve clinical symptoms and exercise capacity, in adults with chronic HFpEF, left ventricular ejection fraction ≥50% and BMI ≥30 kg/m^2.

3.3. Obesity-Related HFpEF: a Specific HFpEF Subphenotype

Currently, multiple classes of drugs are approved for HF and reduced ejection fraction, and some drugs such as sodium-glucose transport protein 2 inhibitors are approved for HF regardless of

ejection fraction. However, the major current challenge in the management of HF is the development of new treatments for HFpEF.

The major factor that drives the development of HFpEF is obesity, and visceral and ectopic adiposity. The accumulation of excess adipose tissue surrounding visceral organs results in a state of systemic inflammation, typically characterized by an increase in hsCRP. This systemic inflammatory state is transduced onto the myocardium by the expansion of epicardial adipose tissue. As a result, the myocardium suffers from inflammation and fibrosis, which limits its distensibility. Due to the concurrence of systemic inflammation and hypervolemia, obesity-related HFpEF is now considered a distinct subphenotype of HFpEF (Obokata et al. 2017; Borlaug et al. 2022).

Patients with obesity-related HFpEF have important degrees of exercise intolerance and a markedly impaired quality of life and health status (Reddy et al. 2020). Among patients with HFpEF, those who have obesity present the greatest impairment in functional capacity, and abdominal obesity is associated with increased morbidity and mortality (Tsujimoto et al. 2017; Chen et al. 2021). In addition, as the state of HFpEF progresses, patients are at risk of rapid worsening of symptoms and signs. They may require a physician visit, during which diuretics are typically added or intensified, either orally or intravenously. In patients with HF, baseline renal impairment and worsening renal function over time are frequently observed and relate to strongly impaired survival (Damman et al. 2014). If severe, these worsening HF events can result in hospitalization, and as the disease continues to advance, obesity-related HFpEF can result in CV death. The occurrence of outpatient or inpatient worsening HF is the most important identifier of subsequent cardiac demise, and thus they are commonly included in composite endpoints – along with CV death – in the evaluation of the efficacy of new treatments for HF.

In a study in HFpEF-patients that evaluated the effect of dapagliflozin versus placebo (DELIVER), the primary endpoint was the composite of CV death and worsening inpatient and outpatient HF events, defined as either an unplanned hospitalization due to HF or an urgent visit due to HF (Solomon et al. 2022). Post hoc analysis demonstrated that outpatient oral diuretic intensification carries risk of subsequent mortality similar to an urgent HF visit (Chatur et al. 2023).

The seminal importance of obesity in obesity-related HFpEF (thus distinguishing it as a distinct HFpEF subphenotype) is strongly supported by 3 important lines of evidence:

1. Weight loss produced by diet is accompanied by an improvement in functional capacity in patients with established obesity-related HFpEF in controlled clinical trials (Kitzman et al. 2016). In a large observational study, bariatric surgery was accompanied by 62% decrease in the incidence of HF in patients with T2D (Aminian et al. 2019). The reduction of HF risk after bariatric surgery is considered to be mediated by weight loss, with a HR for a 10-kg weight loss being 0.77 (Sundström et al. 2017). Bariatric surgery has been associated with a reduction in mortality in patients with established HF (Höskuldsdóttir et al. 2021).

2. In a recently published placebo-controlled study (STEP-HFpEF), semaglutide reduced body weight by 10.7% and improved KCCQ-CSS by 7.8 points at 52 weeks, both with $p<0.001$ (Kosiborod et al. 2023; Borlaug et al. 2023). In addition, superiority on the hierarchical composite endpoint (death, HF events, differences in the change in KCCQ-CSS, and 6MWD) was achieved, including the proportion of patients who had improved KCCQ score by at least 15 points in the semaglutide group. Semaglutide also improved 6MWD, with between-group difference of 20.3 meters. Most intriguingly, the composite of CV death or worsening HF events occurred in 12 patients in the placebo group, but only in 1 patient in the semaglutide group (HR 0.08; 95% CI 0.00 to 0.42).
3. In a recently published large-scale double-blind, placebo-controlled study of patients with obesity and a history of CV disease (SELECT), treatment with a GLP-1 receptor agonist, semaglutide, reduced the risk of CV death, myocardial infarction, stroke, and HF hospitalizations (Lincoff et al. 2023). In this study, 24% of the participants had HF at baseline. Hospitalization or urgent medical visit for HF occurred in 97 participants in the semaglutide group and 122 participants in the placebo group (HR 0.79; 95% CI 0.60 to 1.03). The effects of semaglutide were consistent across prespecified subgroups, but the effects of the drug in the subgroup of patients with obesity-related HFpEF have not yet been reported.

An awareness of the importance of treating obesity in patients with HFpEF is being incorporated in the 2023 American College of Cardiology Expert Consensus Decision Pathway (Kittleson et al. 2023).

Despite the importance of obesity in HFpEF, currently no approved treatment is available for obesity-related HFpEF.

3.4. Tirzepatide in Obesity-Related HFpEF

Tirzepatide has the potential to provide benefit to patients with HFpEF and BMI $\geq$30 kg/m^2.

It has been demonstrated that tirzepatide can provide significant body weight loss in patients with and without T2D. Importantly, the predicted body weight loss with tirzepatide is comparable to the range that can be achieved with bariatric surgery. Data from the SURMOUNT clinical development program demonstrated significant body weight reduction of 22.5% at 72 weeks in SURMOUNT-1 (non-T2D population) and 14.7% at 72 weeks in SURMOUNT-2 (T2D population) (Jastreboff et al. 2022; Garvey et al. 2023).

In a Phase 2 study (Wilson et al. 2022), tirzepatide dose-dependently decreased hsCRP from baseline levels in patients with T2D. This reduction was statistically significant in the tirzepatide 15-mg group (36.2%). Additionally, tirzepatide 15 mg significantly reduced hsCRP versus placebo.

A substudy of the SURPASS-3 study (Gastaldelli et al. 2022) analyzed changes in liver fat content and volumes of visceral adipose tissue and abdominal subcutaneous adipose tissue in response to tirzepatide or insulin degludec. Treatment with tirzepatide for 52 weeks resulted in

significantly greater reductions in liver fat content and in volumes of visceral adipose tissue and abdominal subcutaneous adipose tissue compared with insulin degludec.

A post hoc analysis of the SURPASS-4 study (Heerspink et al. 2022) demonstrated in participants with T2D and high CV risk that tirzepatide slowed the rate of estimated glomerular filtration rate decline and reduced urine albumin–creatinine ratio in clinically meaningful ways compared with insulin glargine.

Given the weight loss achieved with tirzepatide, the effect of tirzepatide on HF events in an obesity-related HFpEF population is expected to be robust. Therefore, it will be meaningful to assess the impact of tirzepatide not only on functional and symptomatic endpoints, but also on the reduction in the risk of worsening HF events.

3.5. SUMMIT Study

SUMMIT is a Phase 3, randomized, multicenter, international, placebo-controlled, double-blind, parallel-arm study. The study is designed to evaluate the effect of tirzepatide once-weekly MTD up to 15 mg on the risk of death, hospitalization, or urgent care visits due to HF, exercise capacity, and health status in participants with HFpEF and BMI ≥30 kg/m^2.

GPID Protocol Amendment (b) is the last approved version of the SUMMIT study protocol. The design of the SUMMIT study in GPID Protocol Amendment (b) specified dual primary endpoints:

- a hierarchical composite of ranked clinical events, with changes in 6MWD, being ranked higher than KCCQ score, and
- 6MWD at Week 52.

The alpha assigned to the hierarchical composite was 0.04, and the alpha assigned to the 6MWD was 0.01.

The SUMMIT study has finished enrollment. [redacted] (chaired by [redacted]) [redacted]

The SUMMIT study is distinguished from the STEP-HFpEF study in several important aspects:

In the SUMMIT study, HfpEF is defined as left ventricular ejection fraction 50% and requires participants to have an estimated glomerular filtration rate <70 mL/min/1.73 m^2 or HF decompensation history within 12 months. The study will continue until the last patient randomly assigned reaches the 52-week visit, extending the time frame of treatment and follow-up of a majority of participants in the study. Last participant visit or study completion is expected to occur in June 2024.

[redacted].

Based on new data presented since the initiation of the SUMMIT study in 2021 including the results of the STEP-HFpEF study, and the differences in the SUMMIT and STEP-HFpEF study design, the Steering Committee has recommended to Lilly that the primary endpoints of the SUMMIT study should be revised.

3.6. Previous Regulatory Interaction with FDA

[redacted]

3.7. Objective of This Briefing Document

The objective of this briefing document is to provide rationale and relevant information for the change of the primary endpoints of the SUMMIT study to:

- time to first occurrence of any component event of CV death or worsening HF event [redacted], and
- KCCQ-CSS at Week 52 [redacted].

To facilitate review, Lilly presents in this document a focused, brief overview of the rationale for the change and the new endpoints strategy, as well as a description of the planned statistical analyses and draft protocol amendment for the SUMMIT study.

4. Meeting Objectives and Topics for Discussion

4.1. Meeting Objectives

The key objectives of this Type C meeting are to gain FDA alignment and advice on

[redacted]

4.2. Topics for Discussion

Lilly proposes a revision of the dual primary endpoints for the SUMMIT study as follows:

- time to first occurrence of any component event of CV death or worsening HF event, and
- KCCQ-CSS at Week 52.

The dual primary endpoints as described in GPID Protocol Amendment (b) are

- a hierarchical composite endpoint of ranked clinical measures, and
- 6MWD at week 52.

Based on the results of the STEP-HFpEF study, and additional reasons described further in this section, the current dual primary endpoints are not likely to capture the most important measures of tirzepatide in patients with HFpEF and obesity.

The proposed approach may be able to demonstrate a more impactful measure of HF outcomes. The alternative hypothesis for the primary objectives is that tirzepatide is superior to placebo in reducing the risk of CV death or worsening HF events and in improving KCCQ-CSS.

Essentially, Lilly is proposing that the components of the current hierarchical composite endpoint be separated into individual dual primary endpoints, that is, instead of evaluating KCCQ-CSS and “worsening HF events” as part of a ranked composite, these 2 endpoints would be evaluated individually. CV death or worsening HF was part of the hierarchical composite endpoint, and this composite is now considered separately. KCCQ-CSS was part of the hierarchical composite endpoint, and it is now considered separately.

[redacted]

Table 5.1 describes the hierarchical composite included as primary endpoint in GPID Protocol Amendment (b) and Table 5.2 describes the revised hierarchical composite that will be assessed as key secondary endpoint.

Question 1:

CCI [redacted]

Question 2:

CCI [redacted]

Question 3:

CCI [redacted]

5. Revised Primary Endpoint Strategy for SUMMIT

5.1. Question 1: Proposed Revision of Primary Endpoints

[redacted]

It is Lilly's intention to change the primary dual endpoints of the SUMMIT study to time to first occurrence of any component event of CV death or worsening HF event [redacted]), and KCCQ-CSS at Week 52 [redacted]

With this new strategy, Lilly aims to provide additional clarity on clinical outcomes and functional benefit.

[redacted]

The hierarchical composite endpoint, as currently defined, presents significant complexity in analytics and interpretation, as it is composed of a combination of clinical endpoints measured over the entire duration of the study with symptomatic and functional endpoints measured after a fixed treatment duration. [redacted]

Given the significant weight loss, and associated cardiometabolic improvements, achieved with tirzepatide, assessment of CV death and worsening HF events, in addition to KCCQ score as a primary endpoint, offers the unique opportunity to evaluate tirzepatide for the benefit of patients with HFpEF and obesity. The dual primary endpoints described in GPID Protocol Amendment (b) may not be able to capture the full potential benefit offered by tirzepatide in this patient population.

5.1.1. Rationale for Worsening Heart Failure Events as One of the Dual Primary Endpoint

The most interesting finding in STEP-HFpEF is a decrease in the risk of the composite of CV death or worsening HF events (HR 0.08; 95% CI 0.00 to 0.42). This was based on only 13 events: 12 events in the placebo group, but only 1 event in the semaglutide group. Because of its larger sample size, longer duration of follow-up and higher (CV death and HF event) risk of participants enrolled, the SUMMIT study is anticipated to accumulate a significantly larger number of events. Therefore, Lilly proposes time to first occurrence of the composite of CV death or worsening HF event as one of the dual primary endpoints.

5.1.2. Rationale for KCCQ-CSS as the Other Primary Endpoint

It is Lilly's intention to include KCCQ-CSS at Week 52 as the other primary endpoint. In the STEP-HFpEF study, as compared with placebo, semaglutide improved KCCQ-CSS by 7.8 points ($p<0.001$).

The primary symptomatic efficacy endpoint in the STEP-HFpEF study was KCCQ-CSS, and the effect size of semaglutide on KCCQ-CSS was substantial, 7.8 points. This treatment effect is markedly larger than that observed in large-scale studies of drugs for HFpEF (PARAGON-HF, EMPEROR-Preserved, DELIVER), which have evaluated KCCQ score (Solomon et al. 2019; Anker et al. 2021; Solomon et al. 2022). The typical effect size in these studies was about 1 to 3 points. In a smaller HFpEF study that predominantly enrolled patients with high BMIs, the change in KCCQ-CSS was 5.8 (Nassif et al. 2021). The effect size on KCCQ-CSS observed in the STEP-HFpEF study was significantly larger. In addition, superiority on the hierarchical composite endpoint was achieved primarily driven by the proportion of patients who had improved KCCQ score by at least 15 points in the semaglutide group.

Table 5.1 and Table 5.2 show the previous (as described in GPID Protocol Amendment [b]) and revised primary and key secondary endpoints of the study, respectively.

Table 5.1. Previous Primary and Key Secondary Objectives and Endpoints for the SUMMIT Study

Objectives	Endpoints
**Primary**	
To demonstrate that a maximally tolerated tirzepatide dose up to 15 mg administered SC QW is superior to placebo based on the hierarchal composite endpoint in participants with HFpEF and BMI ≥30 kg/m^2, analyzed as a win ratio.	A hierarchical composite of the following:<br>1. Time to all-cause mortality through the end of the treatment period<br>2. Occurrence of HF events through end of the treatment period, where HF events include HF hospitalization OR urgent HF visit (adjudicated)<br>• number of HF events<br>• time to first HF event<br>3. Change from baseline in the 6MWD category at Week 52<br>4. Change from baseline in the KCCQ-CSS category at Week 52.<br>The categories for deciding a win on paired testing for the change from baseline in 6MWD are:<br>1. ≥30% worsening<br>2. ≥20% and <30% worsening<br>3. ≥10% and <20% worsening<br>4. No change (<10% change)<br>5. ≥10% and <20% improvement<br>6. ≥20% and <30% improvement, and<br>7. ≥30% improvement.<br>The categories for deciding a win on paired testing for change in baseline in the KCCQ-CSS are:<br>1. ≥10-point worsening

Objectives	Endpoints
	2. ≥5- but <10-point worsening<br>3. No change (<5-point change)<br>4. ≥5- but <10-point improvement, and<br>5. ≥10-point improvement.
To demonstrate that a maximally tolerated tirzepatide dose up to 15 mg administered SC QW is superior to placebo to improve exercise capacity in participants with HFpEF and BMI ≥30 kg/m^2.	Change from baseline to Week 52 in exercise capacity as measured by 6MWD
**Key Secondary (multiplicity controlled)**	
Long-term weight loss	Percent change from baseline to Week 52 in body weight loss
Patient-reported symptoms and physical limitations	Change from baseline to Week 52 in the KCCQ-CSS
Exercise capacity	Change from baseline to Week 24 in 6MWD
NYHA Class	Proportion of participants with NYHA Class change at Week 52

Abbreviations: 6MWD = 6-minute walk distance; BMI = body mass index; HF = heart failure; HfpEF = heart failure with preserved ejection fraction; KCCQ-CSS = Kansas City Cardiomyopathy Questionnaire – Clinical Summary Score; NYHA = New York Heart Association; QW = once weekly; SC = subcutaneous.

Table 5.2. Revised Primary and Key Secondary Objectives and Endpoints for SUMMIT

Objectives	Endpoints
**Primary**	
To demonstrate that a maximally tolerated tirzepatide dose up to 15 mg administered SC QW is superior to placebo based on the composite HF outcome endpoint in participants with HfpEF and obesity.	Time to first occurrence of any component event of CV death or worsening HF events during the study.
To demonstrate that a maximally tolerated tirzepatide dose up to 15 mg administered SC QW is superior to placebo to improve patient-reported symptoms and physical limitations in participants with HfpEF and obesity.	Change from baseline to Week 52 in the KCCQ-CSS

CCI

[redacted]

Objectives	Endpoints
	[redacted]
Exercise capacity	Change from baseline to Week 52 in 6MWD
Long-term weight loss	Percent change from baseline to Week 52 in body weight loss
Evaluation of biomarker hsCRP	Change from baseline to Week 52 in hsCRP

Abbreviations: 6MWD = 6-minute walk distance; BMI = body mass index; hsCRP = high-sensitivity C-reactive protein; HF = heart failure; HFE = heart failure event; HFpEF = heart failure with preserved ejection fraction; KCCQ-CSS = Kansas City Cardiomyopathy Questionnaire – Clinical Summary Score; NYHA = New York Heart Association; QW = once weekly; SC = subcutaneous.

5.2. Question 2: Proposed Definition of Heart Failure Events

[redacted]

It is Lilly's intention to [redacted]

5.2.1. Rationale

It is recognized that the administration of IV drugs as an outpatient can be administratively and technically challenging, and thus oral intensification is often the only possible therapeutic approach for many healthcare systems. Regardless of the physician choice, the critical element of a worsening HF event is the occurrence of worsening symptoms or signs of HF. [redacted]

[redacted]

The recent post hoc analysis of DELIVER (Chatur et al. 2023) has demonstrated that outpatient oral diuretic intensification in ambulatory care carries risk of subsequent mortality similar to an urgent HF visit, and is preceded by clinically significant declines in health status. These findings were consistent with the analyses of TOPCAT-Americas (Ferreira et al. 2022) and the Danish registry (Madelaire et al. 2020). Moreover, inclusion of outpatient worsening HF episodes in the analysis of the EMPEROR-Reduced study has been shown to provide a more comprehensive assessment of HF outcomes (Lam et al. 2021).

5.2.2. Previous Definition of HF Events

[redacted]

5.2.3. Revised Definition of Worsening HF Events

[redacted]

[redacted]

5.3. Question 3: Proposed Statistical Analysis for the Primary and Key Secondary Endpoints

[redacted]

5.3.1. Statistical Hypotheses

The alternative hypothesis for the primary objectives is that tirzepatide MTD is superior to placebo in reducing the risk of CV death or worsening HF event and in improving KCCQ-CSS in adults with HFpEF and BMI ≥30 kg/m^2.

All primary and key secondary endpoint analyses will utilize the graphical multiple testing procedure to control the overall family-wise type I error rate [redacted]

5.3.2. Estimand for Primary Analyses

The primary estimand for primary endpoints is the treatment difference between tirzepatide and placebo relative to the efficacy measures for all participants randomly assigned regardless of adherence to assigned treatment.

The missing measurements for KCCQ-CSS will be imputed through multiple imputation based on the reason for missingness:

- For missing data due to exceptional circumstances, such as pandemics or natural disasters, the missing data is considered as missing at random and multiple imputation will be performed using all nonmissing data from the same treatment arm.
- For missing measurements due to death, multiple imputation will be performed using the worst 15% observed data at 52 weeks from the same treatment group. For all other missing data, retrieved dropout imputation will be applied, which will utilize observed data from participants in the same treatment group who had outcome measures at Week 52 after early discontinuation of study drug to impute the missing value. In case

there are not enough retrieved dropouts to provide a reliable imputation model, reference to placebo imputation will be used.

5.3.3. Primary Endpoints and Analysis

The primary endpoint related to HF outcome will be the time from first dose (for dosed participants) or from randomization (for participants never dosed) to the first occurrence of CV death or worsening HF event during the study.

The primary endpoint related to KCCQ-CSS will be the change from baseline to Week 52 in KCCQ-CSS.

Primary analyses for HF outcome

The primary analysis on the primary hypotheses for HF outcome will be using a Cox proportional hazard model including fixed factors of treatment and the stratification factors of

CCI [redacted]

The complete list of covariates included in the analysis model will be specified in the statistical analysis plan. The censoring date for a participant is the date of last contact when the primary endpoint status is determined. The missing data due to censoring will be implicitly handled by the Cox regression model, assuming censoring is independent of the outcome. The HR, with its 95% CI and p-value, will be provided using the primary analysis model.

The Kaplan–Meier method will be used to estimate the cumulative event curve over time. Counts and proportions of participants who experience a primary endpoint event will be calculated. The total person-years of follow-up, the incidence rate per 100 person-years of follow-up, and the absolute risk difference will be provided.

Primary analyses for KCCQ-CSS

For the change from baseline to Week 52 in KCCQ-CSS, a stratified Wilcoxon (Van Elteren) test will be used as the analysis method, controlling for the stratification factors of

CCI [redacted]

Population-level summary of Hodges–Lehmann estimate for the median difference and 95% confidence interval will be reported.

Missing KCCQ-CSS at Week 52 will be imputed through multiple imputation as specified in Section 5.3.2 prior to ranking for the van Elteren test. The statistical inference over multiple imputation will be guided by the method proposed by Rubin (1987).

5.3.4. Sample Size Justification and Power Calculation

The study has 731 participants randomly assigned to tirzepatide MTD or placebo group in a 1:1 ratio. A sample size of 731 participants will provide over 80% power for the change from baseline to Week 52 in KCCQ-CSS using Wilcoxon rank sum test under the assumptions that the change from baseline to Week 52 in KCCQ-CSS has a mean of 5 and a standard deviation of 19 in placebo and a mean of 10 and a standard deviation of 19 in the tirzepatide group.

Approximately 70 events at the end of the study will provide roughly 80% power to demonstrate the superiority of tirzepatide MTD to placebo in time to first occurrence of CV death or worsening HF event at a 2-sided alpha of 0.04 significance level under the HR assumption of 0.5.

6. References

Aminian A, Zajichek A, Arterburn DE, et al. Association of metabolic surgery with major adverse cardiovascular outcomes in patients with type 2 diabetes and obesity. *JAMA*. 2019;322(13):1271-1282. https://doi.org/10.1001/jama.2019.14231

Anker SD, Butler J, Filippatos G, et al. Empagliflozin in heart failure with a preserved ejection fraction. *N Engl J Med*. 2021;385(16):1451-1461. https://doi.org/10.1056/NEJMoa2107038

Borlaug BA, Jensen MD, Kitzman DW, et al. Obesity and heart failure with preserved ejection fraction: new insights and pathophysiological targets. *Cardiovasc Res*. 2022;118(18):3434-3450. https://doi.org/10.1093/cvr/cvac120

Borlaug BA, Kitzman DW, Davies MJ, et al. Semaglutide in HFpEF across obesity class and by body weight reduction: a prespecified analysis of the STEP-HFpEF trial. *Nat Med*. 2023;29(9):2358-2365. https://doi.org/10.1038/s41591-023-02526-x

Chatur S, Vaduganathan M, Claggett B, et al. Dapagliflozin and diuretic utilization in heart failure with mildly reduced or preserved ejection fraction: the DELIVER trial. *Eur Heart J*. 2023;44(31):2930-2943. https://doi.org/10.1093/eurheartj/ehad283

Chen H, Chhor M, Rayner BS, et al. Evaluation of the diagnostic accuracy of current biomarkers in heart failure with preserved ejection fraction: a systematic review and meta-analysis. *Arch Cardiovasc Dis*. 2021;114(12):793-804. https://doi.org/10.1016/j.acvd.2021.10.007

Damman K, Valente MA, Voors AA, et al. Renal impairment, worsening renal function, and outcome in patients with heart failure: an updated meta-analysis. *Eur Heart J*. 2014;35(7):455-469. https://doi.org/10.1093/eurheartj/eht386

Ferreira JP, Liu J, Claggett BL, et al. Outpatient diuretic intensification as endpoint in heart failure with preserved ejection fraction trials: an analysis from TOPCAT. *Eur J Heart Fail*. 2022;24(2):378-384. https://doi.org/10.1002/ejhf.2376

Garvey WT, Frias JP, Jastreboff AM, et al. Tirzepatide once weekly for the treatment of obesity in people with type 2 diabetes (SURMOUNT-2): a double-blind, randomised, multicentre, placebo-controlled, phase 3 trial. *Lancet*. 2023;402(10402):613-626. https://doi.org/10.1016/S0140-6736(23)01200-X

Gastaldelli A, Cusi K, Fernández Landó L, et al. Effect of tirzepatide versus insulin degludec on liver fat content and abdominal adipose tissue in people with type 2 diabetes (SURPASS-3 MRI): a substudy of the randomised, open-label, parallel-group, phase 3 SURPASS-3 trial. *Lancet Diabetes Endocrinol*. 2022;10(6):393-406. https://doi.org/10.1016/S2213-8587(22)00070-5

Heerspink HJL, Sattar N, Pavo I, et al. Effects of tirzepatide versus insulin glargine on kidney outcomes in type 2 diabetes in the SURPASS-4 trial: post-hoc analysis of an open-label, randomised, phase 3 trial. *Lancet Diabetes Endocrinol*. 2022;10(11):774-785. https://doi.org/10.1016/S2213-8587(22)00243-1

Höskuldsdóttir G, Sattar N, Miftaraj M, et al. Potential effects of bariatric surgery on the incidence of heart failure and atrial fibrillation in patients with type 2 diabetes mellitus and obesity and on mortality in patients with preexisting heart failure: a nationwide, matched,

observational cohort study. *J Am Heart Assoc*. 2021;10(7):e019323. https://doi.org/10.1161/JAHA.120.019323

Jastreboff AM, Aronne LJ, Ahmad NN, et al. Tirzepatide once weekly for the treatment of obesity. *N Engl J Med*. 2022;387(3):205-216. https://doi.org/10.1056/NEJMoa2206038

Kittleson MM, Panjrath GS, Amancherla K, et al. 2023 ACC Expert Consensus Decision Pathway on Management of Heart Failure with Preserved Ejection Fraction: A Report of the American College of Cardiology Solution Set Oversight Committee. *J Am Coll Cardiol*. 2023;81(18):1835-1878. https://doi.org/10.1016/j.jacc.2023.03.393

Kitzman DW, Brubaker P, Morgan T, et al. Effect of caloric restriction or aerobic exercise training on peak oxygen consumption and quality of life in obese older patients with heart failure with preserved ejection fraction: a randomized clinical trial. *JAMA*. 2016;315(1):36-46. https://doi.org/10.1001/jama.2015.17346

Lam CSP, Ferreira JP, Pfarr E, et al. Regional and ethnic influences on the response to empagliflozin in patients with heart failure and a reduced ejection fraction: the EMPEROR-Reduced trial. *Eur Heart J*. 2021;42(43):4442-4451. https://doi.org/10.1093/eurheartj/ehab360

Lincoff AM, Brown-Frandsen K, Colhoun HM, et al. Semaglutide and cardiovascular outcomes in obesity without diabetes [published online Nov 11, 2023]. *N Engl J Med*. https://doi.org/10.1056/NEJMoa2307563

Madelaire C, Gustafsson F, Stevenson LW, et al. One-year mortality after intensification of outpatient diuretic therapy. *J Am Heart Assoc*. 2020;9(14):e016010. https://doi.org/10.1161/JAHA.119.016010

Nassif ME, Windsor SL, Borlaug BA, et al. The SGLT2 inhibitor dapagliflozin in heart failure with preserved ejection fraction: a multicenter randomized trial. *Nat Med*. 2021;27(11):1954-1960. https://doi.org/10.1038/s41591-021-01536-x

Obokata M, Reddy YNV, Pislaru SV, et al. Evidence supporting the existence of a distinct obese phenotype of heart failure with preserved ejection fraction. *Circulation*. 2017;136(1):6-19. https://doi.org/10.1161/CIRCULATIONAHA.116.026807

Reddy YNV, Rikhi A, Obokata M, et al. Quality of life in heart failure with preserved ejection fraction: importance of obesity, functional capacity, and physical inactivity. *Eur J Heart Fail*. 2020;22(6):1009-1018. https://doi.org/10.1002/ejhf.1788

Rubin DB. *Multiple Imputation for Nonresponse in Surveys*. John Wiley & Sons, Inc.; 1987.

Solomon SD, McMurray JJV, Anand IS, et al. Angiotensin-neprilysin inhibition in heart failure with preserved ejection fraction. *N Engl J Med*. 2019;381(17):1609-1620. https://doi.org/10.1056/NEJMoa1908655

Solomon SD, McMurray JJV, Claggett B, et al. Dapagliflozin in heart failure with mildly reduced or preserved ejection fraction. *N Engl J Med*. 2022;387(12):1089-1098. https://doi.org/10.1056/NEJMoa2206286

Sundström J, Bruze G, Ottosson J, et al. Weight loss and heart failure: a nationwide study of gastric bypass surgery versus intensive lifestyle treatment. *Circulation*. 2017;135(17):1577-1585. https://doi.org/10.1161/CIRCULATIONAHA.116.025629

Tsujimoto T, Kajio H. Abdominal obesity is associated with an increased risk of all-cause mortality in patients with HFpEF [published correction appears in *J Am Coll Cardiol*. 2018;71(9):1059]. *J Am Coll Cardiol*. 2017;70(22):2739-2749. https://doi.org/10.1016/j.jacc.2017.09.1111

Wilson JM, Lin Y, Luo MJ, et al. The dual glucose-dependent insulinotropic polypeptide and glucagon-like peptide-1 receptor agonist tirzepatide improves cardiovascular risk biomarkers in patients with type 2 diabetes: a post hoc analysis. *Diabetes Obes Metab*. 2022;24(1):148-153. https://doi.org/10.1111/dom.14553
