# Supplementary material for: Effects of tirzepatide on circulatory overload and end-organ damage in heart failure with preserved ejection fraction and obesity: a secondary analysis of the SUMMIT trial
Source: Nat Med. 2024 Nov 17;31(2):544–51. doi: 10.1038/s41591-024-03374-z (PMC11835708; doi:10.1038/s41591-024-03374-z)
Supplement: Supplementary file 2 — Reporting Summary [file 41591_2024_3374_MOESM2_ESM.pdf]

Corresponding author(s): Barry A Borlaug

Last updated by author(s): Oct 23, 2024

Reporting Summary

Nature Portfolio wishes to improve the reproducibility of the work that we publish. This form provides structure for consistency and transparency in reporting. For further information on Nature Portfolio policies, see our Editorial Policies and the Editorial Policy Checklist.

Statistics

For all statistical analyses, confirm that the following items are present in the figure legend, table legend, main text, or Methods section.

n/a	Confirmed	
☐	☒	The exact sample size (*n*) for each experimental group/condition, given as a discrete number and unit of measurement
☐	☒	A statement on whether measurements were taken from distinct samples or whether the same sample was measured repeatedly
☐	☒	The statistical test(s) used AND whether they are one- or two-sided<br>*Only common tests should be described solely by name; describe more complex techniques in the Methods section.*
☐	☒	A description of all covariates tested
☐	☒	A description of any assumptions or corrections, such as tests of normality and adjustment for multiple comparisons
☐	☒	A full description of the statistical parameters including central tendency (e.g. means) or other basic estimates (e.g. regression coefficient) AND variation (e.g. standard deviation) or associated estimates of uncertainty (e.g. confidence intervals)
☐	☒	For null hypothesis testing, the test statistic (e.g. F, t, r) with confidence intervals, effect sizes, degrees of freedom and P value noted<br>*Give P values as exact values whenever suitable.*
☒	☐	For Bayesian analysis, information on the choice of priors and Markov chain Monte Carlo settings
☒	☐	For hierarchical and complex designs, identification of the appropriate level for tests and full reporting of outcomes
☐	☒	Estimates of effect sizes (e.g. Cohen's d, Pearson's r), indicating how they were calculated

Our web collection on statistics for biologists contains articles on many of the points above.

Software and code

Policy information about availability of computer code

Data collection	No software was used for data collection.
Data analysis	No custom code used for analysis. All analyses were performed in SAS.

For manuscripts utilizing custom algorithms or software that are central to the research but not yet described in published literature, software must be made available to editors and reviewers. We strongly encourage code deposition in a community repository (e.g. GitHub). See the Nature Portfolio guidelines for submitting code & software for further information.

Data

Policy information about availability of data

All manuscripts must include a data availability statement. This statement should provide the following information, where applicable:

- Accession codes, unique identifiers, or web links for publicly available datasets
- A description of any restrictions on data availability
- For clinical datasets or third party data, please ensure that the statement adheres to our policy

Eli Lilly and Company provides access to all individual participant data collected during the trial, after anonymization, except for pharmacokinetic or genetic data. Data are available to request 6months after the indication studied has been approved in the USA and European Union and after primary publication acceptance, whichever is later. No expiration date of data requests is currently set once data have been made available. Access is provided after a proposal has been approved by an independent review committee identified for this purpose and after receipt of a signed data-sharing agreement. Data and documents, including the study

protocol, statistical analysis plan, clinical study report and blank or annotated case report forms, will be provided in a secure data-sharing environment. For details on submitting a request, see the instructions provided at www.vivli.org.

Research involving human participants, their data, or biological material

Policy information about studies with human participants or human data. See also policy information about sex, gender (identity/presentation), and sexual orientation and race, ethnicity and racism.

Reporting on sex and gender	The terms of sex and gender are used appropriately throughout the manuscript.
Reporting on race, ethnicity, or other socially relevant groupings	Data on race and ethnicity are provided.
Population characteristics	Detailed baseline characteristics are presented in Table 1.
Recruitment	Patients with HFpEF were recruited for cardiology and heart failure clinics an participating enrolling centers. There is no identifiable self-selection or other bias, as all patient potentially meeting the eligibility criteria were evaluated for potential enrollment.
Ethics oversight	Individual site IRBs across the 129 centers.

Note that full information on the approval of the study protocol must also be provided in the manuscript.

Field-specific reporting

Please select the one below that is the best fit for your research. If you are not sure, read the appropriate sections before making your selection.

☒ Life sciences ☐ Behavioural & social sciences ☐ Ecological, evolutionary & environmental sciences

For a reference copy of the document with all sections, see nature.com/documents/nr-reporting-summary-flat.pdf

Life sciences study design

All studies must disclose on these points even when the disclosure is negative.

Sample size	All available patients over the study period were included, and the trial was powered to detect an effect on the primary outcome of worsening heart failure event or cardiovascular death (primary endpoint for the overall trial).
Data exclusions	No data were excluded from this analysis
Replication	Replicate measurements were not obtained for clinical or laboratory data. However there was replicate confirmation for statistical analysis. Each statistical analysis was conducted independently by two analysts: a primary analyst and a validation analyst. The validation analysts independently verified the results, and all analyses yielded matching outcomes between the two analysts.
Randomization	This was a randomized trial of tirzepatide vs placebo.
Blinding	This was a double blind trial.

Reporting for specific materials, systems and methods

We require information from authors about some types of materials, experimental systems and methods used in many studies. Here, indicate whether each material, system or method listed is relevant to your study. If you are not sure if a list item applies to your research, read the appropriate section before selecting a response.

Materials & experimental systems

n/a	Involved in the study	
☒	☐	Antibodies
☒	☐	Eukaryotic cell lines
☒	☐	Palaeontology and archaeology
☒	☐	Animals and other organisms
☐	☒	Clinical data
☒	☐	Dual use research of concern
☒	☐	Plants

Methods

n/a	Involved in the study	
☒	☐	ChIP-seq
☒	☐	Flow cytometry
☒	☐	MRI-based neuroimaging

Clinical data

Policy information about clinical studies

All manuscripts should comply with the ICMJE guidelines for publication of clinical research and a completed CONSORT checklist must be included with all submissions.

Clinical trial registration	NCT04847557
Study protocol	Included as supplemental material, along with the statistical analysis plan.
Data collection	Data were collected in individual case report forms in a clinical or hospital setting. Between April 20, 2021 and June 30, 2023, 1494 patients were screened, and 731 patients were randomly assigned to receive tirzepatide (n=364) or placebo (n=367) at 129 centers in 9 countries. Data collection occurred between April 20, 2021 and July 2, 2024.
Outcomes	The primary endpoint of the SUMMIT trial was time to first worsening heart failure event or cardiovascular death, and the trial was powered for this endpoint. The outcomes for this secondary mechanistic analysis from SUMMIT were identified based upon pathophysiologic understanding of obesity-related HFpEF as variables that we hypothesized would change with tirzepatide treatment, including measures of blood pressure, estimated blood and plasma volume, kidney function, albuminuria, cardiac injury, inflammation, and cardiac distention (reflected by NT-proBNP). These variables were obtained based either upon physical examination (for blood pressure) or laboratory tests (from blood and urine samples).

Plants

Seed stocks	NA
Novel plant genotypes	NA
Authentication	NA
